# Catalytic asymmetric functionalization and dearomatization of thiophenes†

**DOI:** 10.1039/d4sc03530e

**Published:** 2024-08-02

**Authors:** Zhengxing Zhao, Yingxin Li, Shiqi Jia, Lei Peng, Zian Zhang, Fengdi Wu, Pengfei Wang, Wenling Qin, Yu Lan, Hailong Yan

**Affiliations:** a Chongqing Key Laboratory of Natural Product Synthesis and Drug Research, School of Pharmaceutical Sciences, Chongqing University Chongqing 401331 P. R. China yhl198151@cqu.edu.cn wenling.qin@cqu.edu.cn; b Green Catalysis Center, College of Chemistry, Zhengzhou University Zhengzhou 450001 P. R. China jiashiqi17@zzu.edu.cn; c Chongqing University FuLing Hospital, Chongqing University Chongqing 408000 P. R. China; d School of Chemistry and Chemical Engineering, Chongqing Key Laboratory of Theoretical and Computational Chemistry, Chongqing University Chongqing 400030 P. R. China

## Abstract

The asymmetric synthesis of thiophene-derived compounds, including catalytic asymmetric dearomatization of thiophene and atroposelective synthesis of benzothiophene derivatives, has rarely been reported. In this work, the asymmetric transformation of the thiophene motif is investigated. Through the rational design of substrates with a thiophene structure, by using the vinylidene *ortho*-quinone methide (VQM) intermediate as a versatile tool, axially chiral naphthyl-benzothiophene derivatives and thiophene-dearomatized chiral spiranes were obtained in high yields with excellent enantioselectivities.

## Abstract 2

In this study, we developed an efficient strategy for the asymmetric intramolecular reactions of VQMs and thiophenes, which proceed *via* two exclusive pathways, yielding axially chiral thiophene derivatives characterized by high stereoselectivity.

## Graphical abstract

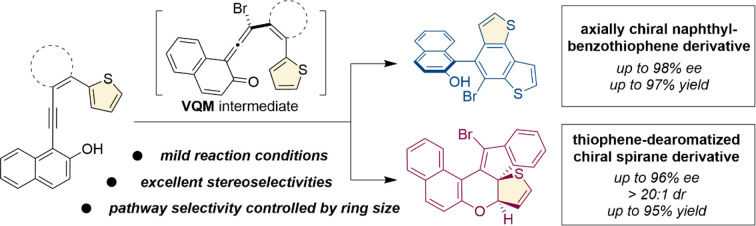

## Introduction

**Scheme 1:**
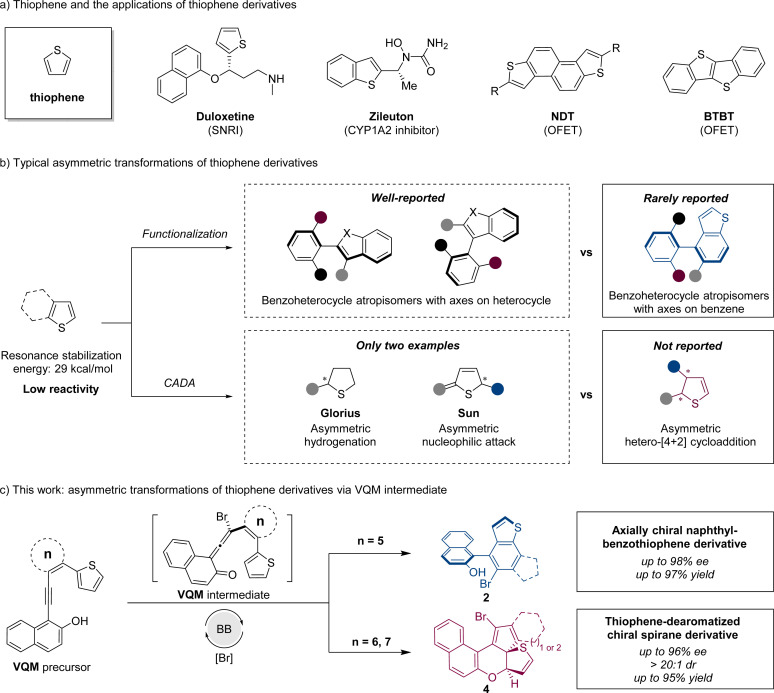
Research background and this work.

Thiophene, a common heterocyclic five-membered aromatic compound, plays a vital role in functional molecules due to its availability and unique properties. As versatile bioactive scaffolds and organic field-effect transistors, stereogenic compounds with a thiophene or benzothiophene nucleus moiety are well applied in pharmaceutical science^1^ and organic semiconducting materials^2^ (Scheme 1a). Due to the great application potential of thiophene-based chiral structures, it is significant to develop asymmetric synthetic strategies to access chiral thiophene derivatives.

Generally, the asymmetric transformation of thiophene involves two strategies: functionalization and catalytic asymmetric dearomatization (CADA) reaction (Scheme 1b). Through the functionalization based on the thiophene structure, a stereogenic axis can be constructed to furnish benzothiophene-derived atropisomers. As an important branch of biaryl atropisomers, axially chiral benzoheterocycles possess higher structural variability because of different heteroatoms and ring sizes,^3^ which sometimes allow some unique properties. Currently, the enantioselective synthesis of chiral benzoheterocycles has been extensively investigated, with the majority of methods emphasizing the construction of stereogenic axes through the high reactivity of the heterocycle. In contrast, there are relatively few reports addressing the construction of chiral axes utilizing the less reactive phenyl moieties, particularly at the 4- and 7-positions.^4^ The development of an efficient method of constructing axially chiral benzothiophenes with axes on the phenyl part is important for benzoheterocycle atropisomers. On the other hand, through the CADA strategy, an unsaturated thiophene-derived skeleton with a higher degree of functionality and structural diversity (sp^3^-content)^5^ can be obtained. Compared to other common heteroarenes like furan, pyrrole, and pyridine, thiophene has higher resonance stabilization energy.^6^ The high stability of thiophene leads to lower reactivities in transformations especially in dearomatizations. To the best of our knowledge, only two examples of thiophene stereoselective dearomatization were reported. The Glorius group reported a direct asymmetric hydrogenation of thiophene with a Ru–NHC catalytic system^7^ and Sun and coworkers reported the first organocatalytic approach with an intermolecular nucleophilic attack process (Scheme 1b).^8^ Developing new construction methods to access different chiral dearomatized thiophene structures is still a challenge.^9^

Vinylidene *ortho*-quinone methide (VQM), as a pluripotent intermediate, is deemed to be a powerful tool for the asymmetric modification and transformation of inert moieties.^10^ Investigations on VQM-based intramolecular reactions with aromatic structures have made some progress. Through intramolecular nucleophilic cyclization^11^ or hetero-[4 + 2] cycloaddition,^12^ interesting chiral products could be constructed in great yields and stereoselectivities. Inspired by reported reactions, we designed two types of substrates to further investigate the application of this synthetic strategy in the asymmetric transformation of inert thiophenes.

We report herein the intramolecular reaction between the thiophene moiety and VQM intermediate. The substrates were rationally designed based on thiophenes and VQM precursors. In these reactions, catalyzed by a chiral Brønsted base (BB), with the brominating reagent as the electrophile, a highly active VQM chiral intermediate could be formed *in situ* to activate inert thiophene structures. The ring size of the tether between VQM and thiophene led to two different pathways: 6π-electrocyclization and dearomatizative intramolecular hetero-[4 + 2] cycloaddition. Through the two pathways, benzothiophene-derived atropisomers and thiophene-dearomatized chiral spirane derivatives were obtained selectively with excellent yields and stereoselectivities (Scheme 1c).

## Results and discussion

At the outset of our study, we designed two types of substrates 1 and 3 with five-membered or larger rings as the tether between the thiophene moiety and VQM precursor. In our preliminary research, the two kinds of structures respectively give the benzothiophene-derived axially chiral biaryls and centrally chiral thiophene-dearomatized products as the only product. We firstly optimized the approach to access the axially chiral benzothiophene derivatives with substrate 1a as the template substrate (Table 1). The atroposelective intramolecular 6π-electrocyclization of 1a was carried out in the presence of 20 mol% catalyst under standard conditions (0.05 M, DCM, −40 °C, and with *N*-bromosuccinimide (NBS) as the brominating reagent). According to our previous results of VQM-mediated asymmetric transformation,^10^ we examined a series of chiral Brønsted base catalysts. With a quinine-derived amide cat-A as the catalyst, the reaction gave decent enantiomeric excess and excellent yield (entry 1, 93% yield, 72% ee). Other catalysts with different positions of substituents on the phenyl moiety (cat B, C, and D) or thiourea instead of amide structure (cat E and F) were also tested. In these cases, 2a was obtained in poorer enantioselectivities albeit with excellent yields (entries 2–6). These results showed that the quinine-derived amide catalyst with the trifluoromethyl substituent group on the *para*-position was essential for matching this substrate to control the stereoselectivity. With cat-A as the best catalyst in hand, the influence of the solvent on the reaction was subsequently examined. After some common solvents (entries 7–11) were screened, CHCl_3_ was found to be the best solvent and the reaction in CHCl_3_ ultimately furnished the product in excellent yield and enantioselectivity (94% yield, 95% ee). Further studies on the reaction temperature hinted that low temperatures were vital to enantioselectivity control. The optimal result was realized at −60 °C (99% yield, 97% ee) and both yield (85%) and ee (65%) significantly decreased at 0 °C (entries 12–14). After that, the concentrations of the reagents in the reaction system were examined. Increasing the reaction concentration (0.1 M) led to a slightly decreased yield. When the reaction concentration decreased to 0.033 M, the yield and enantioselectivity were almost unchanged (entries 15 and 16). Finally, we determined the conditions to give high yield and stereoselectivity and further investigated the influences of the reduction in catalyst loading on the reaction (entry 17). When the catalyst loading decreased to 10 mol%, the enantioselectivity was almost unchanged, and the yield decreased only slightly.

**Table 1:** Optimization of reaction conditions^a^

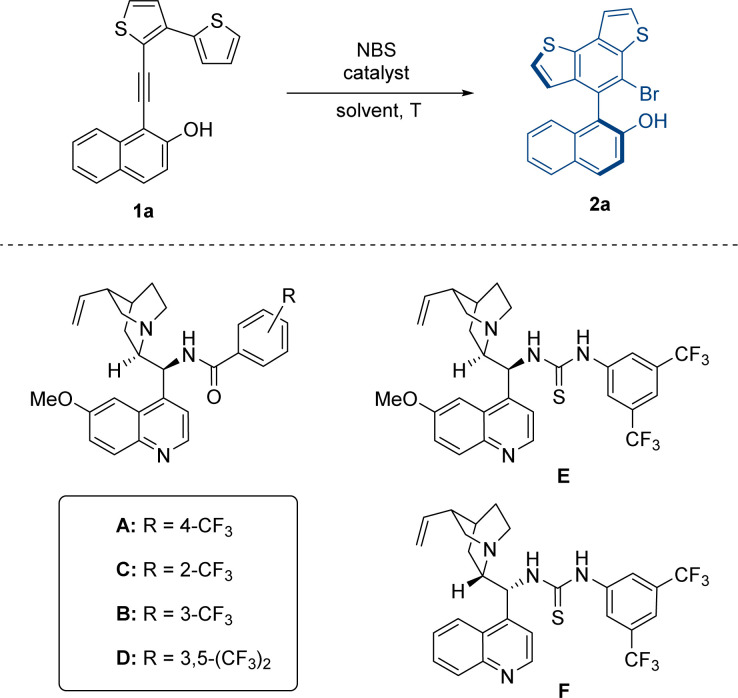					
Entry	Catalyst	Solvent	*T* (°C)	Yield^b^ (%)	ee^c^ (%)
1	A	DCM	−40	93	72
2	B	DCM	−40	91	48
3	C	DCM	−40	87	21
4	D	DCM	−40	82	63
5	E	DCM	−40	98	9
6	F	DCM	−40	97	−4
7	A	Toluene	−40	97	93
8	A	Acetone	−40	95	28
9	A	EtOAc	−40	96	78
10	A	THF	−40	95	26
11	A	CHCl_3_	−40	94	95
12	A	CHCl_3_	0	85	65
13	A	CHCl_3_	−20	93	84
14	A	CHCl_3_	−60	99	97
15^d^	A	CHCl_3_	−60	97	97
16^e^	A	CHCl_3_	−60	99	97
17^f^	A	CHCl_3_	−60	94	97

a Reaction conditions: 1a (0.05 mmol, 1.0 equiv.), catalyst (0.01 mmol, 20 mol%) in solvent (1 mL) at the corresponding temperature for 30 min. Then NBS (0.05 mmol, 1.0 equiv.) was added and stirred under the corresponding temperature for 3 h.

b Isolated yield.

c Enantiomeric excess (ee) determined by HPLC.

d Reaction in CHCl_3_ (0.5 mL).

e Reaction in CHCl_3_ (1.5 mL).

f Catalyst loading: 10 mol%.

Hence, with the optimal reaction conditions, other substrates were investigated so as to elucidate the universality of this atroposelective intramolecular 6π-electrocyclization (Table 2). At the beginning, substrates with substituents on the 6- or 7-position of 2-naphthols were examined. Substrates 1b–g, with different substituents such as bromo, *n*-propyl, cyclohexyl, methoxyl, and phenyl on the 6- or 7-position of the 2-naphthol moiety, were well tolerated and delivered the corresponding products 2b–g in excellent yields and enantioselectivities (79–93% yields, 96–99% ee). Then, we explored the effect of the substituent position of thiophene (ring A) on the reaction. The positions of alkynyl and ring B were exchanged so as to produce substrates 1h–k, which successfully delivered products 2h–k in excellent yields and enantioselectivities (89–94% yields, 91–98% ee). The influence of ring B on the reaction was further evaluated. When the substituted position of ring B was changed from 2-position to 3-position of thiophene or the thiophene of ring A or B was replaced with furan, the reaction also proceeded well to give high yields and enantioselectivity (2l–n, 76–97% yields, 93–97% ee). The configurational stability of 2l was investigated and this new axially chiral biaryl skeleton retained excellent thermal stability at 120 °C. The Δ*G*^‡^ was computed to be 37.7 kcal mol^−1^ and the *t*_1/2_ was calculated to be 10 905 h (see the ESI† for details). When ring B was replaced by benzothiophene or benzofuran (1o, 1p), the reaction still gave good results (2o, 2p, 88–90% yields, 90–98% ee). Products 2q–s with methyl-substituted ring A indicated that this reaction had good tolerance to the substituent groups on ring A. When five-membered ring B was replaced by phenyl or naphthyl (1t, 1u), the products were also obtained in good yields and excellent enantioselectivities. To further explore the reaction, the iodo-substituted instead of bromo-substituted product 2v was prepared with NIS as the iodinating reagent. The iodo-substituted VQM did not give good enantioselectivity. The result might be interpreted as follows. Due to the higher reactivity of the iodo cation, the fast formation speed of the iodo-substituted VQM intermediate impaired the chirality control process. We also made an attempt on the hydro-substituted product with cat-E as the catalyst without any other additives, but the reactivity of hydro-substituted VQM was too low to give any conversion. Finally, we examined the substrates with cyclopentene as ring A (1v, 1w). Substrates 1v and 1w underwent the same reaction and delivered products 2w and 2x in excellent yields and medium enantioselectivities (89–90% yields, 70–71% ee). The absolute configurations of 2a and 2l were determined to be a*S* by single-crystal X-ray crystallographic analysis and others were assigned by analogy.

**Table 2:** Substrate scope^a^

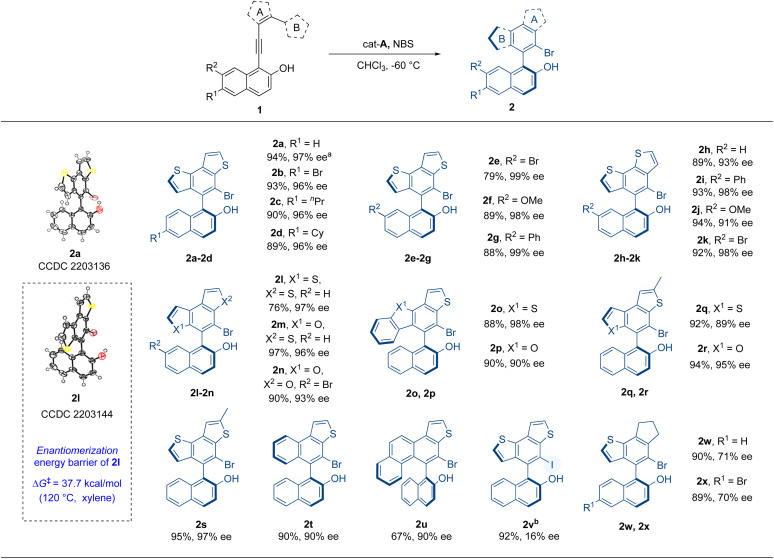

a Reaction conditions: 1 (0.05 mmol, 1.0 equiv.), cat-A (0.005 mmol, 10 mol%) in CHCl_3_ (1.0 mL) at −60 °C for 30 min, then NBS (0.05 mmol, 1.0 equiv.) was added at −60 °C, with stirring continued at −60 °C for 3 h.

b Reaction conditions for 2v: 1a (0.05 mmol, 1.0 equiv.), cat-A (0.005 mmol, 10 mol%) in CHCl_3_ (1.0 mL) at −60 °C for 30 min, then NIS (0.05 mmol, 1.0 equiv.) was added at −60 °C, with stirring continued at −60 °C for 3 h.

After establishing the process for atroposelective intramolecular 6π-electrocyclization, we intended to investigate the scope of thiophene-dearomatized products obtained from substrates 3 through an asymmetric intramolecular [4 + 2] hetero-Diels–Alder cycloaddition (Table 3). With a benzene ring as the linkage between the thiophene and VQM precursor, we prepared substrate 3a in order to verify the applicability of this dearomatizative [4 + 2] hetero-Diels–Alder cycloaddition to the optimal conditions (Table 2). To our delight, this reaction proceeded smoothly to give the desired thiophene-dearomatized adduct 4a with an excellent yield and enantioselectivity (89% yield, 90% ee). Thus, with the optimal conditions of the atroposelective 6π-electrocyclization as the standard reaction conditions, we examined the substrates with substituents on the 6- or 7-position of 2-naphthols. Substrates 3b–d with bromo and phenyl substituents on the 6- or 7-position of the 2-naphthol moiety were well tolerated and delivered the corresponding products 4b–d in excellent yields and good to excellent enantioselectivities (87–95% yields, 85–96% ee). In detail, the substrates with bromo substituents performed better than phenyl-substituted and non-substituted substrates in enantioselective control. Substrates 3e–g, with fluoro or methyl substituents on the phenyl moiety, were also tolerated and delivered products 4e–g in excellent yields and good to excellent enantioselectivities (85–90% yields, 85–90% ee). After that, we further explored the influences of the substituent groups on the thiophene moiety. After introducing methyl and bromo substituents on thiophene (ring B) or changing the connection position of thiophene from 2-position to 3-position (substrates 3h–l), we furnished the desired products 4h–l with 74–92% yields and 86–90% ee. Then, the stability of the chiral axes of the dearomatized products was tested. However, the product fully decomposed after 8 h at 100 °C. Due to the poor thermostability of these products, the rotational barrier of the axis was obtained by DFT calculation (see the ESI† for details). According to the calculation results, the rotational barrier of product 4l was determined to be 16.2 kcal mol^−1^. And the configuration of the axis is controlled by the stereogenic centers. We then tested the substrates with cyclohexene and cycloheptene as ring A (3m, 3n). Similar to the axially chiral products 2w and 2x, the corresponding products (4m and 4n) were obtained with good yields but with less satisfactory enantioselectivities. These results indicated that the aliphatic rings tethered between VQM and thiophene impaired the stereogenicity control. We then replaced the thiophene motif with furan and benzothiophene (3o and 3p) and found that the reactions showed good yields but unsatisfactory stereoselectivities under standard reaction conditions. Finally, we examined the products with a hydro or iodo substituent instead of the bromo substituent. At room temperature, with cat-E as the catalyst, under the conditions without NBS, the hydro-substituted products 4q, 4r and 4s were obtained in excellent stereoselectivities from substrates 3a, 3o and 3p. The conversion of thiophene dearomatization through hydro-substituted VQM was much lower than other cases due to the lower reactivity of the hydro-substituted VQM intermediate and high stability of thiophene. The iodo-substituted product was obtained through a highly active iodo-substituted VQM in an excellent yield but with lower stereoselectivity (94% yield, 27% ee). All the substrates tested gave excellent diastereoselectivity (>20 : 1 dr). These results indicated a broad substrate scope of the given methodology. Through this reaction, the catalytic asymmetric dearomatization of thiophene could be efficiently achieved. The absolute configurations of 4b and 4l were determined by X-ray crystallographic analysis.^13^

**Table 3:** Substrate scope^a^

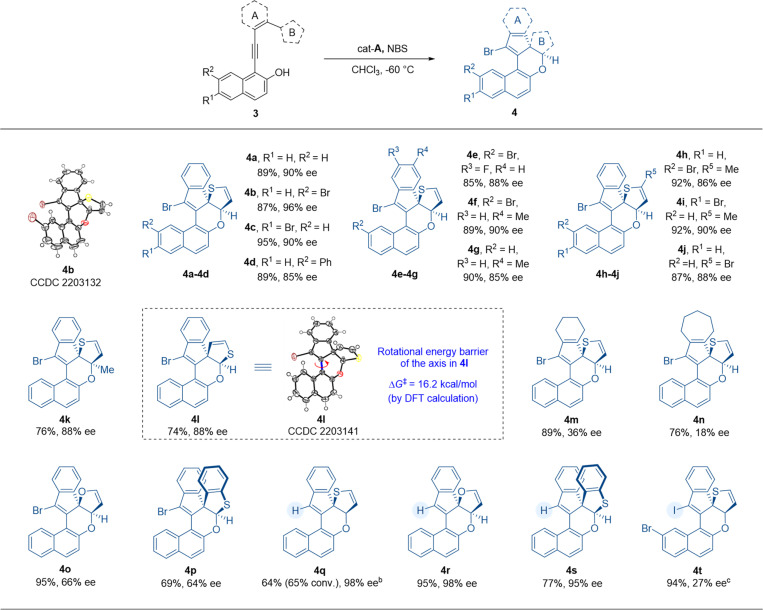

a Reaction conditions: 3 (0.05 mmol, 1.0 equiv.), cat-A (0.005 mmol, 10 mol%) in CHCl_3_ (1.0 mL) at −60 °C for 30 min, then NBS (0.05 mmol, 1.0 equiv.) was added at −60 °C, with stirring continued at −60 °C for 3 h.

b Reaction conditions for 4q–4s: 3 (0.05 mmol, 1.0 equiv.), cat-E (0.005 mmol, 10 mol%) in CHCl_3_ (1.0 mL) at rt for 24 h.

c Reaction conditions for 4t: 3t (0.05 mmol, 1.0 equiv.), cat-A (0.005 mmol, 10 mol%) in CHCl_3_ (1.0 mL) at −60 °C for 30 min, then NIS (0.05 mmol, 1.0 equiv.) was added at −60 °C, with stirring continued at −60 °C for 3 h.

**Scheme 2:**
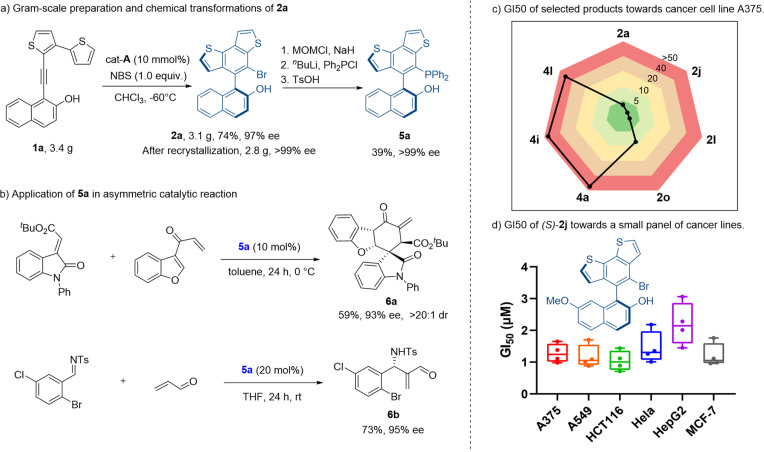
(a) Gram-scale preparation and chemical transformations of 2a. (b) Application of 5a in an asymmetric catalytic reaction. (c) GI_50_ values of selected products towards cancer cell line A375. (d) GI50 of (*S*)-2j towards a small panel of cancer cell lines.

We then explored the synthetic application of our method. This thermostable benzodithiophene-naphthol biaryl skeleton may be applied in the design of novel catalysts and ligands. We started from the gram-scale synthesis of axially chiral adduct 2a, under the amplified standard reaction conditions. Product 2a was obtained in 74% yield with 97% ee. Despite a slight decrease of yield, the gram-scale reaction gave a good result for further derivatization. After recrystallization, the enantiomeric excess of compound 2a was improved to >99%. The optically pure product was transformed into a novel chiral phosphine catalyst 5a (Scheme 2a) with excellent retention of optical purity (99% ee) and medium yield (39% over three steps). The application of this phosphine catalyst 5a was illustrated by the stereoselective formal [4 + 2] cycloaddition reaction and aza-Morita–Baylis–Hillman (MBH) reaction (Scheme 2b). With 5a as the reaction catalyst, corresponding chiral products 6a and 6b were obtained in moderate yields with excellent stereoselectivities (93% ee, >20 : 1 dr, 95% ee respectively).

As indicated in extensive studies,^1^ thiophenes are significant substructure moieties in numerous bioactive molecules. Thus, we evaluated whether these products possessed interesting pharmacological profiles. The *in vitro* antiproliferation assays were performed with a small panel of cancer cell lines. Both kinds of products were tested. Interestingly, product 2 generally possessed considerable antiproliferation potency toward all cancer cell lines of A375, A549, HCT116, HeLa, HepG2, and MCF-7 other than the non-cancer cells L02 (Scheme 2c). The most potent compound 2j with a methoxyl group exhibited single-digital micromolar GI_50_ values ranging from 1.04 to 2.2 μM (Scheme 2d). In contrast, product 4 lost the impressive anticancer activity, as indicated by the poor or zero inhibition effect (GI_50_ > 50 μM) observed in the screened cell lines (Table S3†). This finding suggested that both the modifications in certain positions of the scaffold and the presence of the sp^2^ carbon (unique three-dimensionality, physicochemical properties, *etc.*) were the determinant factors of bioactivity in this case.

**Scheme 3:**
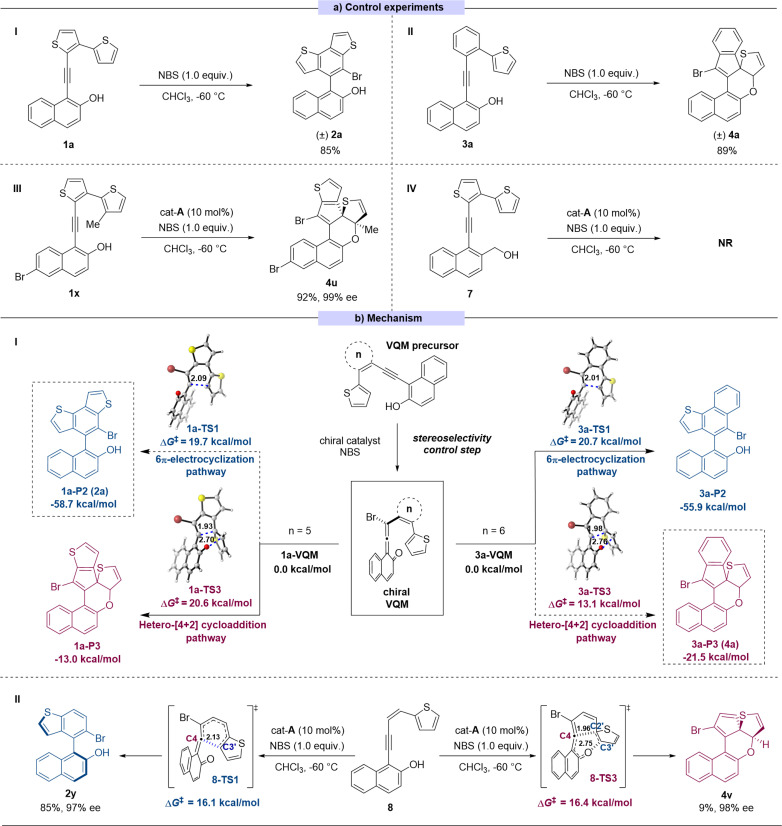
(a): Control experiments. (b(I)): DFT calculations for the regioselectivity of substrates 1a and 3a. (b(II)): DFT calculations for specialized substrate 8 (the values of bond lengths are given in angstrom).

To better understand the selectivity control process of this reaction, a series of control experiments were performed (Scheme 3a). In the absence of catalyst, the 6π-electrocyclization exclusively occurred to 1a to give the sole product (±)-2a and 3a gave the dearomatized product (±)-4a (Scheme 3a(I) and (II)). These results suggested that the pathway selectivity of the reactions only depended on the substrate structures, whereas the catalysts only controlled the stereoselectivity of the transformation. In order to explore the switch between the two pathways, we made some modifications on substrate 1. By introducing a methyl group onto the C3′ position of thiophene, the reaction site of 6π-electrocyclization was locked theoretically (substrate 1x). As expected, in this case, the reaction pathway switched to the intramolecular [4 + 2] cycloaddition process to give the corresponding product 4u with excellent yield and stereoselectivity (Scheme 3a(III)). The absolute configuration of 4u was determined by X-ray crystallographic analysis of the corresponding transformed product 4u′ (for synthesis of 4u′ see ESI Section XII†). To verify the presumed VQM-mediated mechanism, we designed substrate 7 in which the hydroxyl on the naphthyl moiety was replaced with the hydroxymethyl group to prohibit the formation of the VQM intermediate. The reaction did not take place or give any product, demonstrating the key role of the VQM intermediate in this transformation (Scheme 3a(IV)).

Based on our previous reports^14^ and the conducted control experiments, we have elucidated the mechanism underlying the developed reaction (Scheme 3b(I)). In the presence of a chiral catalyst, the VQM precursor substrate can undergo transformation into a chiral VQM intermediate through asymmetric bromination, utilizing NBS as the bromine source. Once the chiral VQM moiety is formed, the central carbon (C4) of the allene demonstrates unique reactivity, capable of reacting with the intramolecular thiophenyl group at either the C3′ position through pericyclic 6π-electrocyclization or the C2′ position through hetero-[4 + 2] cycloaddition, resulting in the formation of axially chiral naphthyl-benzodithiophene architectures 2 and the thiophene-dearomatized product 4. We hypothesize that ring strain restricts the formation of new C–C bonds, thereby influencing the corresponding regioselectivity.

To further confirm this hypothesis, DFT calculations were performed at the M06-2X/6-311+G(d,p)/SMD(chloroform) level of theory^15^ to investigate the regioselectivity and diastereoselectivity (see the ESI†). All relative free energies are reported with respect to the corresponding VQMs, and values are given in kcal mol^−1^. Our calculations indicate that the 6π-electrocyclization proceeds *via* a stepwise reaction, whereas the [4 + 2] cycloaddition pathway occurs through a concerted pericyclic process. Two typical VQM intermediates, 1a-VQM, and 3a-VQM with 5- or 6-membered rings as the linkage, were chosen as model reactants. As shown in Scheme 3b(I), when the conjugating group is set to a thiophene (1a-VQM), the calculated activation free energy of 6π-electrocyclization is 19.7 kcal mol^−1^, which is 0.9 kcal mol^−1^ lower than that of [4 + 2] hetero-Diels–Alder cycloaddition. In another case, when the conjugating group is mutated to a phenyl ring (3a-VQM), the free energy barrier for [4 + 2] hetero-Diels–Alder cycloaddition (Δ*G*^‡^ = 13.1 kcal mol^−1^) is lower than that for 6π-electrocyclization (Δ*G*^‡^ = 20.7 kcal mol^−1^), indicating that the latter becomes the favorable pathway. Additionally, we considered specialized substrates 8, where the conjunction fragment is an open-chain alkene without restriction. The competition between pericyclic 6π-electrocyclization and hetero-[4 + 2] cycloaddition has been assessed through DFT calculations. As illustrated in Scheme 3b(II), 6π-electrocyclization can take place *via* a concerted six-membered ring-type transition state 8-TS1, leading to the formation of a C4–C3′ bond with a calculated activation free energy of 16.1 kcal mol^−1^. The resulting cyclic intermediate III-P1 could undergo a pericyclic [1,9]-hydrogen shift *via* transition state 8-TS2, leading to aromatization and the production of product 2y (see the ESI† for details). In contrast, in the other pathway, an intramolecular [4 + 2] hetero-Diels–Alder cycloaddition could occur *via* a concerted transition state 8-TS3, resulting in the formation of 4v with a C4–C2′ single bond. The calculated activation free energy for this process is 16.4 kcal mol^−1^, which is close to that of the 6π-electrocyclization pathway *via*8-TS1 (8-ΔΔ*G*_(__TS1–TS3_^‡^_)_) = −0.3 kcal mol^−1^). This suggests that both reactions may occur, leading to the formation of different products, which is consistent with the experimental result (2y : 4v = 85 : 9).

## Conclusions

In conclusion, we have developed an effective strategy for the asymmetric transformation of thiophene derivatives. By carefully designing the substrates, we enabled a stereoselective intramolecular reaction between vinylquinone imines (VQMs) and thiophenes to proceed through two distinct pathways: one involving an atroposelective 6π-electrocyclization, resulting in a new type of axially chiral naphthyl-benzothiophene derivatives, and the other corresponding to an efficient catalytic asymmetric dearomatization (CADA) of inert thiophenes *via* a dearomatizative intramolecular [4 + 2] hetero-Diels–Alder cycloaddition. Preliminary studies indicate that some of these compounds have anti-cancer properties and can catalyze asymmetric transformations. Furthermore, density functional theory (DFT) calculations clarified the selectivity between these two pathways.

## Data availability

Data for this article, including experimental procedures, DFT calculations, and characterization data of all the substrates and products, are available in the ESI† of the manuscript. Deposition numbers 2203136 (for 2a), 2203144 (for 2l), 2203132 (for 4b), 2203141 (for 4l) and 2203146 (for 4u′) contain the supplementary crystallographic data for this paper.†

## Author contributions

Hailong Yan and Wenling Qin conceived and directed the project. Zhengxing Zhao designed and performed the experiments. Yingxin Li, Lei Peng, and Zian Zhang undertook part of the work in the substrate scope. Fengdi Wu and Pengfei Wang finished the pharmacological research. Zhengxing Zhao and Shiqi Jia prepared the ESI.† Zhengxing and Shiqi Jia analysed and interpreted the experimental data. Zhengxing, Shiqi Jia, Wenling Qin, and Hailong Yan wrote the paper. Shiqi Jia and Yu Lan performed the DFT calculations. All authors discussed the results and commented on the manuscript.

## Conflicts of interest

There are no conflicts to declare.

## Supplementary Material

SC-OLF-D4SC03530E-s001

SC-OLF-D4SC03530E-s002
