# Supplementary material for: Catalytic asymmetric functionalization and dearomatization of thiophenes
Source: Chem Sci. 2024 Aug 2;15(35):14295–303. doi: 10.1039/d4sc03530e (PMC11325187; doi:10.1039/d4sc03530e)
Supplement: SC-015-D4SC03530E-s001 [file SC-015-D4SC03530E-s001.pdf]

Supplementary Information (SI) for Chemical Science.
This journal is © The Royal Society of Chemistry 2024

Supplementary Information

Catalytic Asymmetric Functionalization and Dearomatization of Thiophenes

Zhengxing Zhao[1], Yingxin Li[1], Shiqi Jia[2]*, Lei Peng[1, 3], Zian Zhang[1], Fengdi Wu[1], Pengfei Wang[1], Wenling Qin[1]*, Yu Lan[2,4], Hailong Yan[1]*

[1]Chongqing Key Laboratory of Natural Product Synthesis and Drug Research, School of Pharmaceutical Sciences, Chongqing University, Chongqing 401331, P. R. China

[2]Green Catalysis Center, College of Chemistry, Zhengzhou University, Zhengzhou, 450001, P. R. China.

[3]Chongqing University FuLing Hospital, Chongqing University, Chongqing 408000, P. R. China.

[4]School of Chemistry and Chemical Engineering, Chongqing Key Laboratory of Theoretical and Computational Chemistry, Chongqing University, Chongqing, 400030, P. R. China.

*Corresponding authors. E-mails: jiashiqi17@zzu.edu.cn; wenling.qin@cqu.edu.cn; yhl198151@cqu.edu.cn

Table of Contents

I. General information S2
II. General procedure for the synthesis of the substrates S2
III. DFT Calculation S6
IV. Optimization of the reaction condition. S18
V. General procedure for asymmetric reaction S19
VI. Experiments about other substituted VQMs....................S19
VII. ^{1}H, ^{13}C NMR and HRMS data of compounds (1a-1am)....................S20
VIII. ^{1}H, ^{13}C NMR and HRMS data of compounds (2a-2x, 3a-3t) S36
IX. Rotational barriers of 2l, 3l....................S65
X. Pharmacological research S68
XI. Gram-scale preparation and transformations....................S69
XII. Mechanistic studies....................S75
XIII. ^{1}H and ^{13}C NMR spectra....................S81
XIV. X-ray crystallographic information S181
References....................S189

I. General information

^{1}H and ^{13}C NMR spectra were recorded on Agilent 400MR DD2 (400 MHz) spectrometer and Agilent 600MR DD2 (600 MHz) spectrometer. Chemical shifts were reported in parts per million (ppm), and tetramethylsilane or the residual solvent peak was used as an internal reference: $CDCl_3$ (^{1}H NMR tetramethylsilane δ 0.00, ^{13}C NMR δ 77.00), data are reported as follows: chemical shift, multiplicity (s = singlet, d = doublet, t = triplet, q = quartet, m = multiplet, br = broad), coupling constants (Hz) and integration. Enantiomeric excesses (ee) were determined by HPLC analysis on Hitachi Chromaster using DAICEL CHIRALCEL AD-H, 4.6mm*Φ*×250mm, DAICEL CHIRALCEL AS-H, 4.6mm*Φ*×250mm, DAICEL CHIRALCEL OD-H, 4.6mm*Φ*×250mm, DAICEL CHIRALCEL IA-H, 4.6mm*Φ*×250mm, DAICEL CHIRALCEL IB-H, 4.6mm*Φ*×250mm. High resolution mass spectra (HRMS) were performed on Bruker Solarix 7.0 T. X-ray crystallography analysis of single crystal was performed on an Agilent SuperNova-CCD X-Ray diffractometer. Optical rotations were measured on a Rudolph Autopol I polarimeter and are reported as follows: $[\alpha]_D^{25}$ (*c* in g per 10 mL solvent). Unless otherwise stated, all reagents were purchased from commercial suppliers (MERYER, Adamas, J&K, Sigma-Aldrich, TCI, etc.) and used without further purification.

II. General procedure for the synthesis of the substrates

Method A: (1a-1u, 1x)

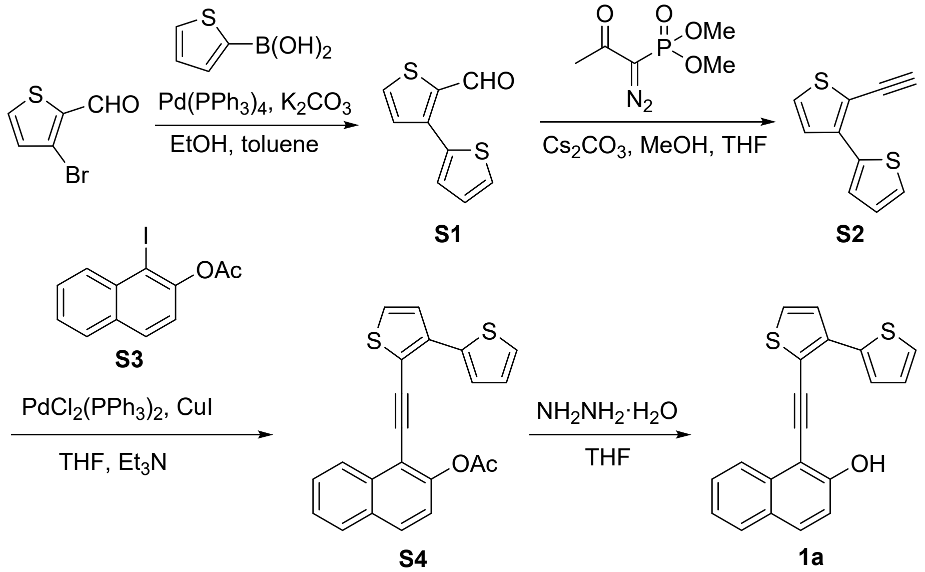

[2,3'-bithiophene]-2'-carbaldehyde (S1)

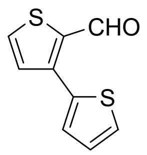

To a stirring solution of 3-bromo-thiophene-2-carbaldehyde (3.82 g, 20 mmol, 1.0 equiv.), $Pd(PPh)_4$ (462 mg, 0.4 mmol, 0.02 equiv.), and 2-thiopheneboronic acid (2.82 g, 22 mmol, 1.1 equiv.) in toluene(40 ml) under nitrogen was added sodium carbonate solution (1.0 M, 20 ml). The solution was heated under reflux for 8 h, then was extracted with EA three times. The combined organic layers were dried over $MgSO_4$. The solvent was evaporated to yield [2,3']bithiophenyl-2'-carbaldehyde as a colorless oil which was used directly in next step, without further purification.

2'-ethynyl-2,3'-bithiophene (S2)

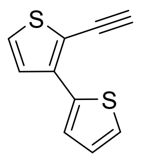

S1 (1.94 g, 10 mmol, 1.0 equiv.) was dissolved in THF (20 ml) and MeOH (10 ml). Cs_2CO_3 (276.4 mg, 20 mmol, 1.0 equiv.) was added. Bestmann-Ohira Reagent (288.2 mg, 1.1 mmol) was added dropwise. The resulting mixture was stirred at rt overnight and then concentrated. The residue was purified on silica gel with PE to give the corresponding substituted acetylene **S2**.

1-([2,3'-bithiophen]-2'-ylethynyl)naphthalen-2-yl acetate (S4)

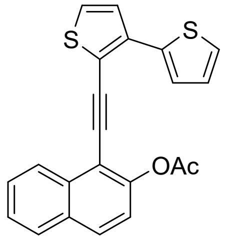

S3 (1.25 g, 4 mmol, 1.0 equiv.), $PdCl_2(PPh_3)_2$ (56 mg, 0.08 mmol, 0.02 equiv.) and CuI (38 mg, 0.2 mmol, 0.05 equiv.) were weighed and added into an oven dried flask, evacuated and backfilled with nitrogen (3 times). Et_3N (8 mL) and THF (8 mL) was injected into the flask. Then **S2** (836 mg, 4.4 mmol, 1.1 equiv.) was added. The resulting mixture kept stirring at room temperature for 24 h. Then the mixture was filtered through a pad of celite and washed with EA. Removal of solvent under reduced pressure, purified by column chromatography on silica gel (PE/EA = 20:1) to afford **S4** (1.12 g, 3.0 mmol, 75 % yield) as a yellow solid.

General procedure for the synthesis of **S3**: see reference **1**.

1-([2,3'-bithiophen]-2'-ylethynyl)naphthalen-2-ol (1a)

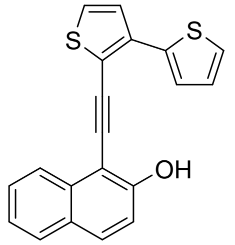

To a stirred solution of **S4** (1.12 g, 3.0 mmol, 1.0 equiv.) in THF (10 mL) was added hydrazine monohydrate (0.52 mL, 9 mmol, 3.0 equiv., 80%) slowly at rt. Then, the resulting solution was kept stirring until **S4** was consumed. Quenched with saturated aqueous NH_4Cl, extracted with EA, washed with brine, dried over Na_2SO_4 and filtered, concentrated under reduced pressure and purified by column chromatography on silica gel (PE/EA = 20:1) to afford the desired product **1a** (945 mg, 2.85 mmol, 95% yield) as a yellow solid.

Method B: (3a-3g, 3k, 3l, 3o, 3p)

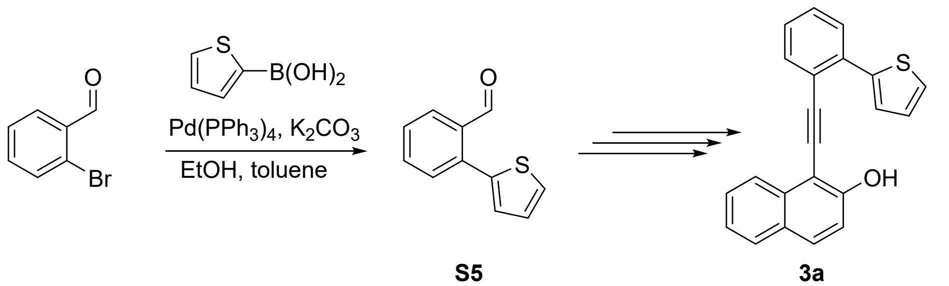

2-(thiophen-2-yl)benzaldehyde (S5)

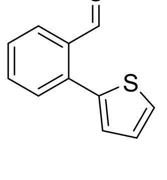

To a stirring solution of 2-thiopheneboronic acid (1.54 g, 12 mmol, 1.2 equiv.), Pd(PPh)$_4$ (231 mg, 0.2 mmol, 0.02 equiv.), and 2-bromobenzaldehyde (1.85 g, 10 mmol, 1.0 equiv.) in toluene(20 ml) under nitrogen was added sodium carbonate solution (1.0 M, 10 ml). The solution was heated under reflux for 4 h, then was extracted with EA three times. The combined organic layers were dried over $MgSO_4$. The solvent was evaporated, the crude product was purified by silica gel.

Compound **3a** was prepared according to the general procedure as described for **1a**

Method C: (3h-3j)

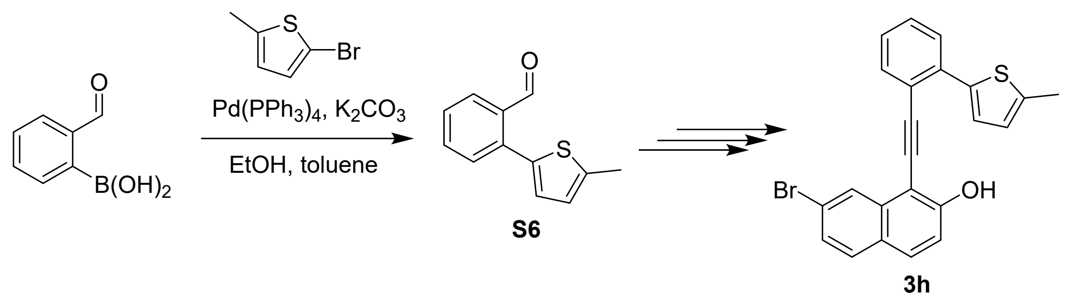

2-(5-methylthiophen-2-yl)benzaldehyde (S6)

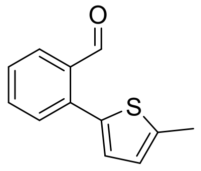

To a stirring solution of (2-formylphenyl)boronic acid (1.8 g, 12 mmol, 1.2 equiv.), $Pd(PPh)_4$ (231 mg, 0.2 mmol, 0.02 equiv.), and 2-bromo-5-methylthiophene (1.7 g, 10 mmol, 1.0 equiv.) in toluene(20 ml) under nitrogen was added sodium carbonate solution (1.0 M, 10 ml). The solution was heated under reflux for 4 h, then was extracted with EA three times. The combined organic layers were dried over $MgSO_4$. The solvent was evaporated, the crude product was purified by silica gel.

Compound **3h** was prepared according to the general procedure as described for **1a**

Method D: (8)

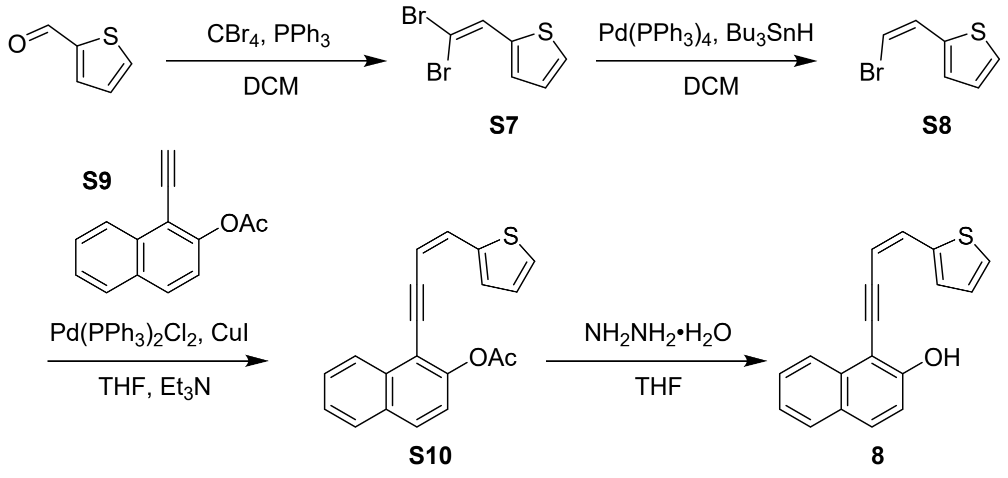

2-(2,2-dibromovinyl)thiophene (S7)

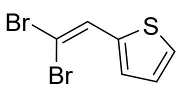

To an ice-cold stirred solution of PPh_3 (15.8 g, 60 mmol, 3 equiv.) in DCM (60 mL), CBr_4 (10 g, 30 mmol, 1.1 equiv.) in DCM (10 mL) was added. After 1 hour, 2-thenaldehyde (2.25 g, 20 mmol, 1.0 equiv.) and in DCM (20 mL) was added dropwise. After the reaction was complete, PE was added to the mixture. The suspended mixture was filtered to remove triphenylphosphine oxide. After evaporation of solvents, the crude product **S7** was used directly in next step, without further purification.

(*Z*)-2-(2-bromovinyl)thiophene (S8)

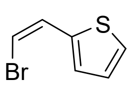

To a stirred solution of **S7** (900 mg, 3.37 mmol, 1.0 equiv.) and $Pd(PPh_3)_4$ (128 mg, 0.111 mmol, 0.03 equiv.) in dried DCM (10 mL) was added *n*-Bu_3SnH (1 mL, 3.7 mmol, 1.1 equiv.) dropwise, and the mixture

was stirred overnight. The mixture was hydrolyzed by the addition of aqueous potassium fluoride. After 90 min, the precipitated tin salts were filtered under vacuum and the residual solution was extracted with DCM. The organic layer was washed with brine and water, dried over sodium sulfate and concentrated. Purification of the residue by flash column chromatography on basic alumina and finely ground KF (90:10%, w/w) using PE as eluent furnished the product as a yellow oil. Yield; (445 mg, 70%).

(*Z*)-1-(4-(thiophen-2-yl)but-3-en-1-yn-1-yl)naphthalen-2-yl acetate (S10)

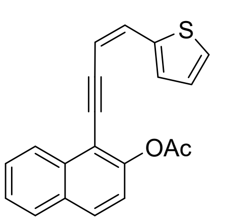

$PdCl_2(PPh_3)_2$ (70 mg, 0.1 mmol, 0.03 equiv.) and CuI (30 mg, 0.16 mmol, 0.05 equiv.) were weighed and added into an oven dried flask, evacuated and backfilled with nitrogen (3 times). **S8** (0.6 g, 3.2 mmol, 1.0 equiv.) in Et_3N (8 mL) was injected into the flask. The mixture was heated to 70 °C, then alkyne **S9** (882 mg, 4.2 mmol, 1.3 equiv.) in THF (8 mL) was added slowly. The resulting mixture kept stirring at 70 °C for 3 h. Then the mixture was filtered through a pad of celite and washed with EA. Removal of solvent under reduced pressure, purified by column chromatography on silica gel (PE/EA = 10:1) to afford **S10** (882 mg, 2.5 mmol, 78 % yield) as a yellow oil.

General procedure for the synthesis of **S9**: see reference **1**.

Compound **8** was prepared according to the general procedure as described for **1a**

Method E: (1v, 1w, 3m, 3n)

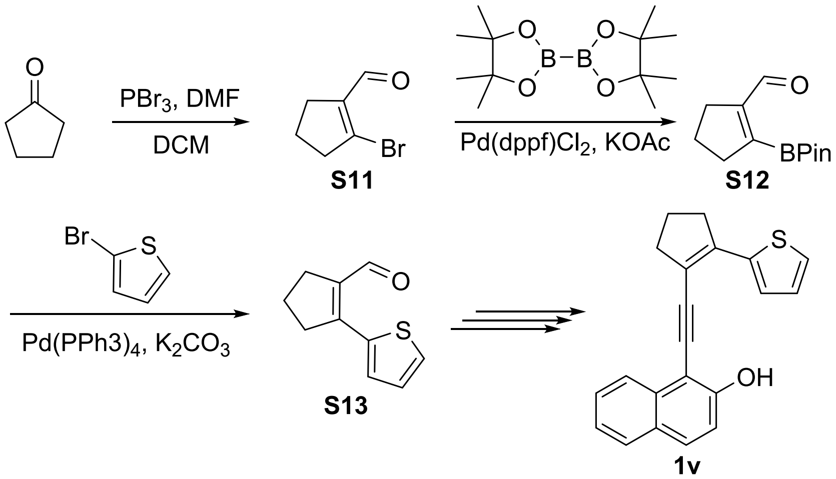

2-bromocyclopent-1-ene-1-carbaldehyde(S11)

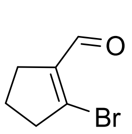

In a 500ml dry three-port bottle, DMF (615 mmol, 47.5 ml) and 300 ml DCM were added under a nitrogen atmosphere. After stirring at 0 °C for 10 minutes, phosphorus tribromide (532 mmol, 50.0 ml) was added. After stirring at 0 °C for 1 h, (205 mmol, 18.2 ml) was added dropwise. After 0.5 hours, turn off the cold trap. Stirring was continued for 8 hours after slowly rising to room temperature, then the reaction mixture was poured into a bath of ice, and completely quenched with $NaHCO_3$. Extract three times with DCM and combine the organic phases. Wash with water, saturated sodium chloride, dry over anhydrous sodium sulfate, and filter. After evaporation of solvents, the crude product **S11** was used directly in next step, without further purification.

2-(4,4,5,5-tetramethyl-1,3,2-dioxaborolan-2-yl)cyclopent-1-ene-1-carbaldehyde(S12)

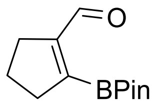

S11 (1.86 g, 10 mmol), bis(pinacolato)diboron (3.0 g, 12 mmol), $Pd(dppf)_2Cl_2$ (0.4 g, 0.5 mmol), and potassium acetate (2.9 g, 30 mmol) were dissolved in 30 mL of 1,4-dioxane. The reaction mixture was degassed with nitrogen and then heated to 85 °C. under nitrogen for 18 hours. The reaction mixture was filtered and an extraction was performed in ethyl acetate. The organic phase was washed with water and brine. The extract was dried over sodium sulfate, filtered, and concentrated. The resulting residue was purified by a silica gel column.

2-(thiophen-2-yl)cyclopent-1-ene-1-carbaldehyde(S13)

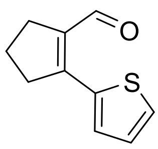

To a stirring solution of **S12** (1.33 g, 6 mmol, 1.2 equiv.), $Pd(PPh_3)_4$ (115 mg, 0.1 mmol, 0.02 equiv.), and 2-bromo-5-methylthiophene (1.7 g, 10 mmol, 1.0 equiv.) in toluene(20 ml) under nitrogen was added sodium bicarbonate solution (1.0 M, 10 ml). The solution was heated under reflux for 4 h, then was extracted with EA three times. The combined organic layers were dried over $MgSO_4$. The solvent was evaporated, the crude product was purified by silica gel.

Compound **1v** was prepared according to the general procedure as described for **1a**

III. DFT Calculation

1. Computational Methods.

All density functional theory (DFT) calculations were performed with the Gaussian 09[2] software package. Geometries were optimized in toluene with the SMD solvation model[3] using the B3-LYP-D3[4]functional and a basis set of 6-31G(d)[5]. Vibrational frequencies were computed at the same level to evaluate its zero-point vibrational energy and thermal corrections at 298 K, and to check whether each optimized structure is a transition state or not. The single-point energies and solvent effects in toluene were computed at the M06-2X level of theory with the 6-311+G(d,p)[6]basis set, using the solvent-phase optimized structures. Solvation energies were evaluated by a self-consistent reaction field (SCRF) using the SMD model.

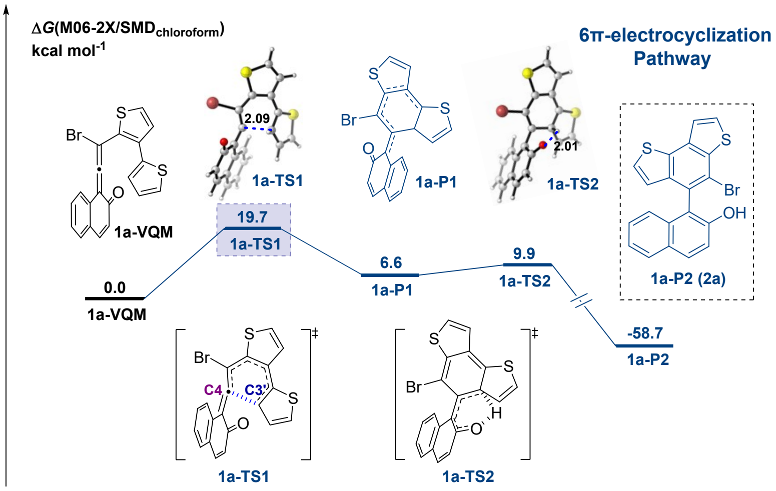
ΔG(M06-2X/SMDchloroform)
kcal mol-1
6π-electrocyclization Pathway
1a-VQM
1a-TS1
1a-P1
1a-TS2
1a-P2 (2a)
2.09
2.01
19.7
6.6
9.9
0.0
-58.7
1a-P2
C4
C3'
Br
OH

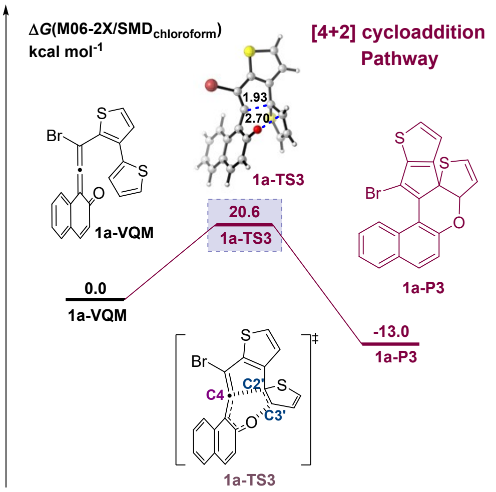
ΔG(M06-2X/SMDchloroform)
kcal mol-1
[4+2] cycloaddition Pathway
1a-VQM
1a-TS3
1a-P3
1.93
2.70
20.6
0.0
-13.0
C4
C2'
C3'
Br

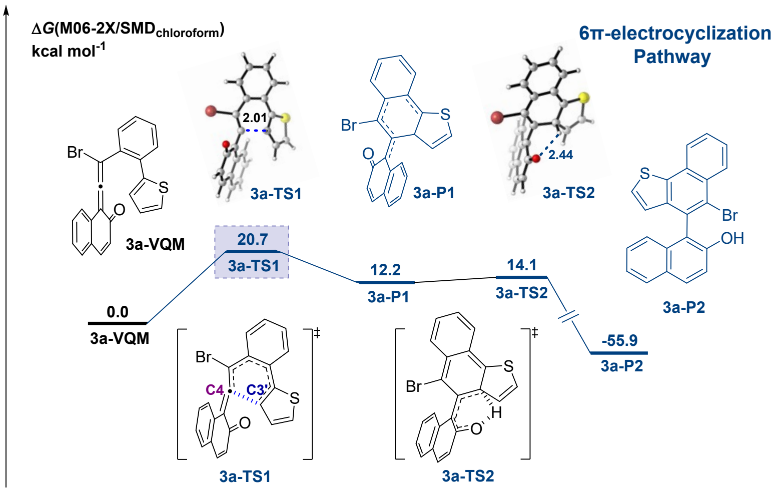
ΔG(M06-2X/SMDchloroform)
kcal mol-1
6π-electrocyclization Pathway
3a-VQM
3a-TS1
3a-P1
3a-TS2
3a-P2
2.01
2.44
20.7
12.2
14.1
0.0
-55.9
C4
C3'
Br
OH

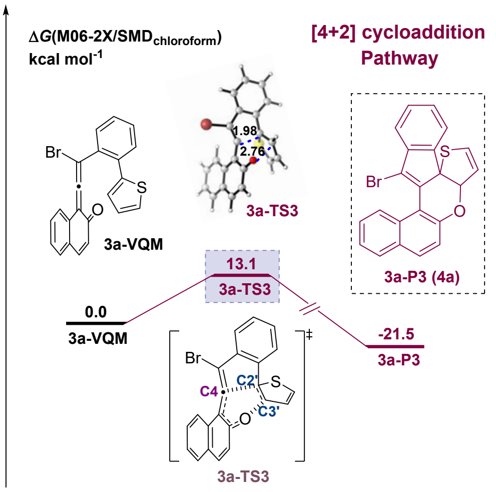
ΔG(M06-2X/SMDchloroform)
kcal mol-1
[4+2] cycloaddition Pathway
3a-VQM
3a-TS3
3a-P3 (4a)
3a-P3
1.98
2.76
13.1
0.0
-21.5
C4
C2'
C3'
Br

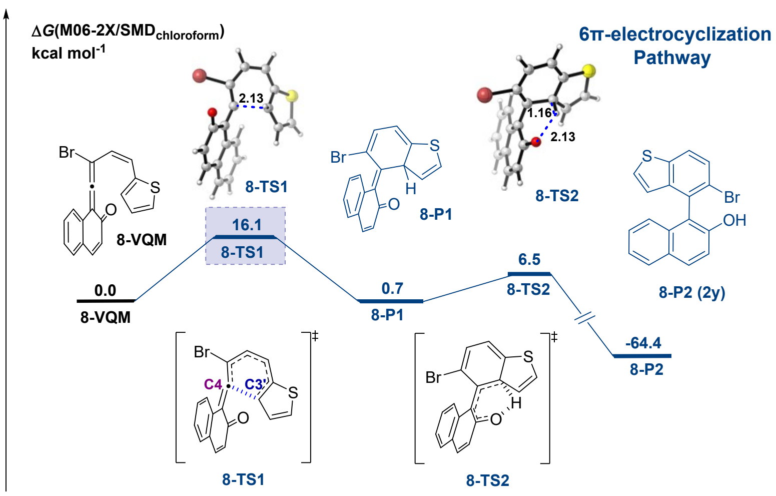
ΔG(M06-2X/SMDchloroform)
kcal mol-1
6π-electrocyclization Pathway
8-VQM
8-TS1
8-P1
8-TS2
8-P2 (2y)
8-P2
2.13
1.16
2.13
16.1
0.7
6.5
0.0
-64.4
C4
C3'
Br
OH

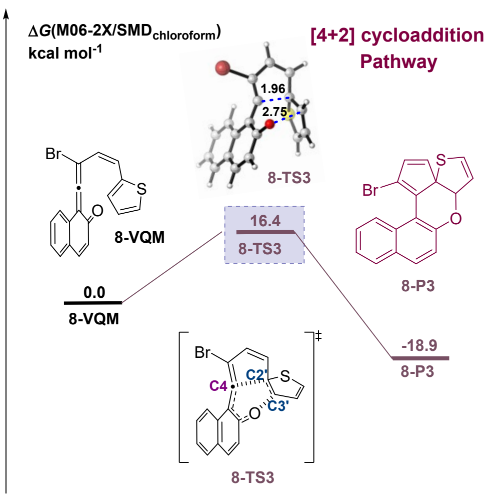
ΔG(M06-2X/SMDchloroform)
kcal mol-1
[4+2] cycloaddition Pathway
8-VQM
8-TS3
8-P3
1.96
2.75
16.4
0.0
-18.9
C4
C2'
C3'
Br

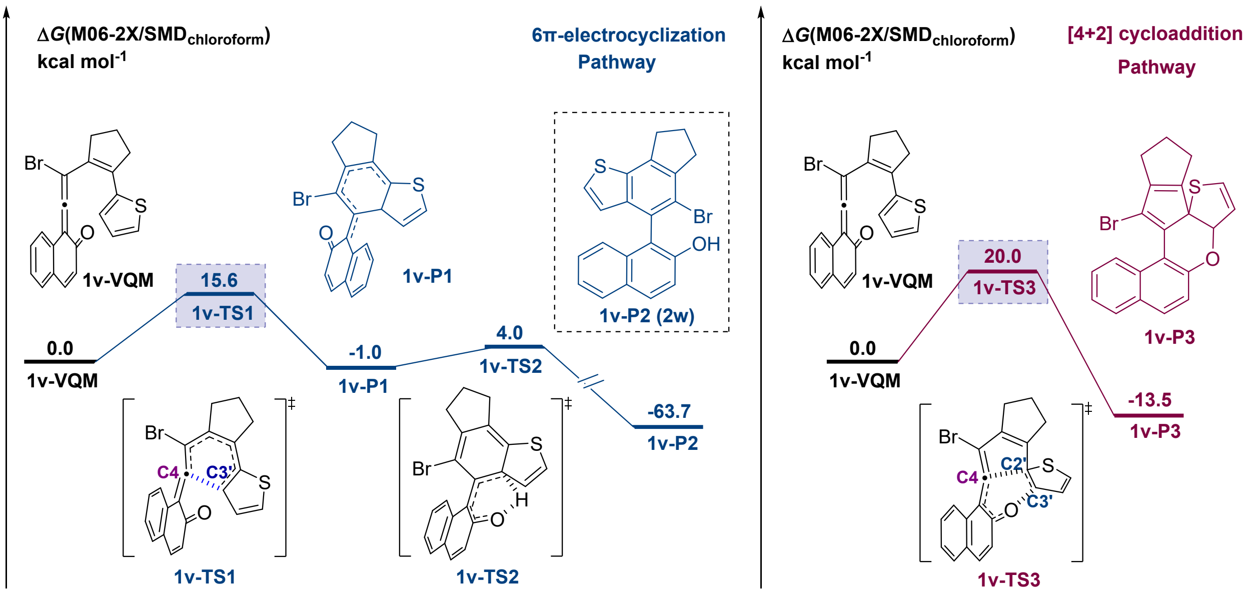

Supplementary Figure 1. DFT-computed Gibbs free energy profiles.

3a-VQM as the template substrate for DFT theoretical calculations to study the chirality transfer process

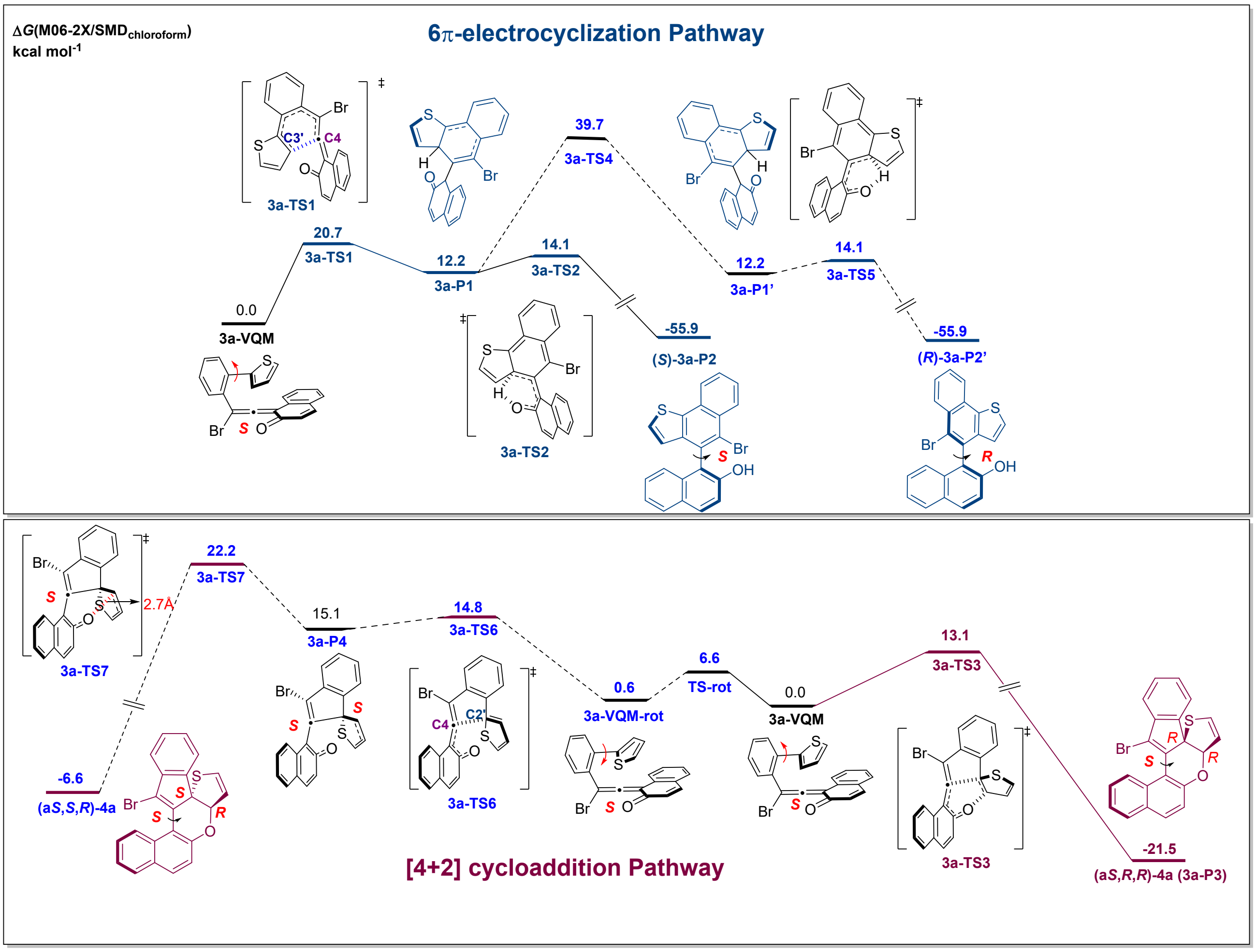

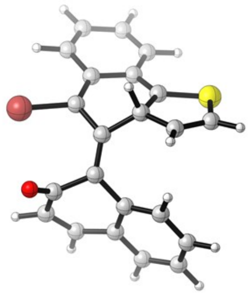

3a-TS4

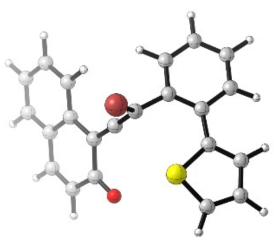

TS-rot

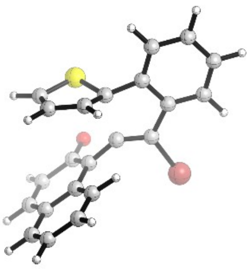

3a-TS6

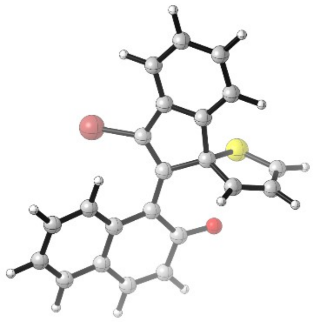

3a-TS7

2. B3-LYP-D3 and M06-2X calculated absolute energies, and free energies of all structures

Supplementary Table 1. Energy datas

Geometry	$E_{(elec\text{-}B3\text{-}LYP\text{-}D3)}$[1]	$G_{(corr\text{-}B3\text{-}LYP\text{-}D3)}$[2]	$H_{(corr\text{-}B3\text{-}LYP\text{-}D3)}$[3]	$E_{(solv\text{-}M06\text{-}2X)}$[4]	IF[5]
**1a-VQM**	-4211.73	0.1941	0.2674	-4214.35	-
**1a-TS1**	-4211.71	0.1970	0.2659	-4214.32	377.99*i*
**1a-TS2**	-4211.72	0.1990	0.2653	-4214.33	50.94*i*
**1a-TS3**	-4211.7	0.1968	0.2657	-4214.32	345.76*i*
**1a-P1**	-4211.73	0.1993	0.2674	-4214.34	-
**1a-P2**	-4211.82	0.2010	0.2698	-4214.45	-
**1a-P3**	-4211.75	0.2024	0.2695	-4214.37	-
**3a-VQM**	-3890.98	0.2285	0.3014	-3893.58	-
**3a-TS1**	-3890.95	0.2302	0.2998	-3893.55	376.83*i*
**3a-TS2**	-3890.96	0.2326	0.2996	-3893.56	46.44*i*
**3a-TS3**	-3890.96	0.2303	0.2997	-3893.56	353.32*i*
**3a-P1**	-3890.97	0.2319	0.3009	-3893.56	-
**3a-P2**	-3891.06	0.2346	0.3037	-3893.67	-
**3a-P3**	-3891.02	0.2364	0.3035	-3893.62	-
**8-VQM**	-3737.31	0.1836	0.2514	-3739.94	-
**8-TS1**	-3737.29	0.1864	0.2501	-3739.92	331.54*i*
**8-TS2**	-3737.31	0.1881	0.2490	-3739.93	83.13*i*
**8-TS3**	-3737.29	0.1865	0.2496	-3739.92	369.77*i*
**8-P1**	-3737.32	0.1891	0.2516	-3739.94	-
**8-P2**	-3737.41	0.1908	0.2539	-3740.05	-
**8-P3**	-3737.35	0.1924	0.2533	-3739.98	-
**1v-VQM**	-3854.06	0.2429	0.3176	-3856.66	-
**1v-TS1**	-3854.05	0.2471	0.3171	-3856.64	316.61*i*
**1v-TS2**	-3854.07	0.2497	0.3162	-3856.66	65.11*i*
**1v-TS3**	-3854.04	0.2472	0.3168	-3856.63	341.19*i*
**1v-P1**	-3854.07	0.2500	0.3185	-3856.67	-
**1v-P2**	-3854.16	0.2520	0.3209	-3856.77	-
**1v-P3**	-3854.09	0.2528	0.3206	-3856.69	-
**3a-TS4**	-3890.92	0.2334	0.3001	-3893.52	28.40*i*
**3-VQM-rot**	-3890.98	0.2289	0.3014	-3893.58	-
**TS-rot**	-3890.97	0.2269	0.3002	-3893.56	47.37*i*
**3a-TS6**	-3890.96	0.2306	0.2997	-3893.56	333.20*i*
**3a-P4**	-3890.96	0.2313	0.3012	-3893.56	-
**3a-TS7**	-3890.95	0.2328	0.2997	-3893.55	143.95*i*
**(a*S*,*S*,*R*,)-4a**	-3890.99	0.2371	0.3031	-3893.60	-

[1]The electronic energy calculated by B3-LYP-D3 in chloroform solvent. [2]The thermal correction to Gibbs free energy calculated by B3-LYP-D3 in chloroform solvent. [3]The thermal correction to enthalpy calculated by B3-LYP-D3 in chloroform solvent. [4]The electronic energy calculated by M06-2X in chloroform solvent. [5]The B3-LYP-D3 calculated imaginary frequencies for the transition states.

Cartesian coordinates of the structures

1a-P1

Br	-0.90934400	-2.66746400	-0.59784400
O	0.85791600	-2.27833700	1.99717100
C	2.44048900	0.13210700	-0.29020200
C	1.39241300	-0.58136900	0.39862200
C	2.16899400	1.15894300	-1.23083800
H	1.13807900	1.39438900	-1.47830000
C	-1.08397700	-0.87685800	0.05618900
C	0.04635500	-0.11738900	0.35483500
C	1.71122300	-1.70861400	1.28645000
C	3.19448000	1.84116400	-1.87172100
H	2.95241000	2.63178900	-2.57745600
C	3.80651100	-0.24170200	-0.09280300
C	4.53564700	1.49632600	-1.63705500
H	5.33532500	2.02285900	-2.15049600
C	4.10512200	-1.36228000	0.76491300
H	5.15013800	-1.64072200	0.89090100
C	3.12871600	-2.06731600	1.38766300
H	3.35916000	-2.91418400	2.02865100
C	4.82794100	0.45132100	-0.76891200
H	5.86009700	0.14858200	-0.60573800
C	-2.36992200	-0.30482200	-0.01188200
C	-2.62550200	1.10245200	0.12373400
S	-3.83188800	-1.14190700	-0.48315200
C	-3.99414000	1.44517600	-0.18060100
C	-4.74154400	0.36313100	-0.49761600
H	-4.38290800	2.45635100	-0.13116600
H	-5.79649200	0.33698600	-0.74249100
C	-1.55726800	1.91168600	0.45368000
C	-0.28362300	1.28857900	0.91705700
S	-1.46412800	3.64105400	0.40380500
C	0.73598900	2.37486700	1.06347400
C	0.24250000	3.60790400	0.88347900
H	1.74993000	2.17485500	1.38117200
H	0.76295400	4.55197500	0.99324600
H	-0.51116400	0.95888400	1.95760400

1a-P2

Br	-0.16137500	-2.65834400	-0.11233700
O	0.98684100	-0.74487700	2.85560500
C	2.40576800	0.30084400	-0.40606300
C	1.43146600	0.00562100	0.59963200
C	2.04609300	0.74897200	-1.70722000
H	0.99610800	0.87079800	-1.95346700
C	-0.86495500	-0.88325300	0.01768300
C	-0.02565000	0.18222600	0.33484100
C	1.84878700	-0.43890600	1.84689600
C	3.01086600	1.02154900	-2.65423400
H	2.71295900	1.36076400	-3.64300200
C	3.79850000	0.13503100	-0.10485600
C	4.38500200	0.86048900	-2.35409500
H	5.13365200	1.07789100	-3.11098200
C	4.17230000	-0.32092900	1.18965600
H	5.22814600	-0.44513900	1.41734400
C	3.22640100	-0.60145000	2.14288600
H	3.50079600	-0.94999500	3.13378800
C	4.76579300	0.42391200	-1.10349100
H	5.81789200	0.29244900	-0.86057200
C	-2.23760800	-0.68343100	-0.22925000
C	-2.82173700	0.60383000	-0.17138900
S	-3.43363000	-1.89735200	-0.64586500
C	-4.22893500	0.57735800	-0.46404500
C	-4.68179600	-0.67603500	-0.73144800
H	-4.86085000	1.45879800	-0.47096600
H	-5.69356300	-0.97158500	-0.98180500
C	-1.97596600	1.68363800	0.14953500
C	-0.60217500	1.48600800	0.40045600
S	-2.41451500	3.37409800	0.29219700
C	0.07241900	2.72282200	0.70551300
H	0.07202400	-0.60595200	2.55071700
C	-0.75884000	3.79636700	0.68298900
H	1.13110000	2.78432200	0.92626700
H	-0.50893300	4.83277700	0.87435400

1a-P3

Br	0.01305100	-2.81807800	-0.61359700
O	-0.18288000	2.34717500	-1.55603100
C	-2.39561400	-0.05340000	0.21748700
C	-1.18894100	0.53182800	-0.29311400
C	-2.39323400	-1.12005800	1.15502000
H	-1.44671400	-1.49723200	1.52387100
C	0.78077900	-1.15308500	-0.15944800
C	0.15904400	0.05444500	-0.04667600
C	-1.27164300	1.69733800	-1.05924600
C	-3.57413600	-1.65791700	1.62316700
H	-3.54541200	-2.46952500	2.34553800
C	-3.66009000	0.48531800	-0.19179100
C	-4.82108200	-1.15666100	1.18095100
H	-5.74367700	-1.59339300	1.55336000
C	-3.68496500	1.62071200	-1.04914100
H	-4.64612600	2.02208500	-1.36027200
C	-2.52317600	2.22890900	-1.45473300
H	-2.52990000	3.11240600	-2.08519100
C	-4.85794900	-0.09870800	0.29837800
H	-5.80891300	0.31521100	-0.02883000
C	2.21576200	-0.98018600	-0.01675900
C	2.51803200	0.35096700	0.15658900
S	3.63120700	-1.97390500	-0.02153900
C	3.90735800	0.60551600	0.29754000
C	4.63496800	-0.55588100	0.21803700
H	4.35018700	1.58334900	0.44978000
H	5.70919400	-0.67601300	0.28251100
C	1.22790400	1.12570200	0.15848000
C	1.13770600	2.19869600	-0.96246400
S	1.02072500	2.04426000	1.78312300
C	1.52185200	3.51885900	-0.35158100
H	1.78380500	1.89984700	-1.79222000
C	1.44930300	3.56080700	0.98306300
H	1.74551600	4.37726500	-0.97567900
H	1.61614000	4.43643700	1.60336800

1a-TS1

Br	-1.07838600	-2.58774000	-0.90883200
O	0.76230700	-2.39635100	1.93056800
C	2.51230300	0.03246400	-0.23978700
C	1.40746100	-0.67913700	0.38731600
C	2.30383500	1.09485900	-1.14304700
H	1.28824700	1.38658800	-1.39096700
C	-1.10175900	-0.85384300	-0.02527300
C	0.10440600	-0.30336500	0.20948000

C	1.66864200	-1.81110900	1.32236500
C	3.37737800	1.75985300	-1.72605400
H	3.19022700	2.57973900	-2.41443700
C	3.84922700	-0.36768300	0.03853700
C	4.69391800	1.37269200	-1.43713800
H	5.53072500	1.89072800	-1.89718800
C	4.08327500	-1.48947100	0.92462600
H	5.11714700	-1.77604800	1.10883600
C	3.07529200	-2.17711600	1.51094200
H	3.26630800	-3.01637600	2.17364100
C	4.92115300	0.31137100	-0.56740500
H	5.93716000	-0.00826700	-0.34748500
C	-2.38777900	-0.23658600	0.12020900
C	-2.60429500	1.14490700	0.31112100
S	-3.90501400	-1.05235400	-0.19015600
C	-3.98198100	1.51468100	0.16114500
C	-4.78787400	0.44525800	-0.08157300
H	-4.34130900	2.53176900	0.27352300
H	-5.86535900	0.43745000	-0.19174100
C	-1.48779800	1.98781000	0.54342000
C	-0.29778200	1.51585400	1.14797100
S	-1.29868300	3.61376700	-0.05732700
C	0.73463600	2.53461600	1.15142000
H	-0.38941600	0.82991400	1.98686900
C	0.34220800	3.68922200	0.55767000
H	1.70955300	2.39213500	1.60085700
H	0.91412600	4.59980800	0.42669700

1a-TS2

Br	-0.48534400	-2.73197200	-0.30544700
O	0.66152800	-0.55643900	2.71301700
C	2.49603500	0.07393500	-0.37801500
C	1.42067300	-0.24834900	0.49298700
C	2.29905300	0.52400700	-1.71381100
H	1.28404200	0.61492700	-2.09352900
C	-0.95418900	-0.90029700	-0.03760200
C	0.02996100	0.03066600	0.17704500
C	1.62445000	-0.55757600	1.88646300
C	3.37415300	0.82778000	-2.52850000
H	3.19881900	1.17782700	-3.54300100
C	3.84283300	-0.08194300	0.09352200
C	4.69757300	0.66887300	-2.06224000
H	5.53593700	0.89590100	-2.71511500
C	4.04921400	-0.52602000	1.44176500
H	5.07398900	-0.66206800	1.78391900
C	3.00832200	-0.75071100	2.29498600
H	3.17665600	-1.05276600	3.32567900
C	4.91841300	0.21269000	-0.77288100
H	5.93426900	0.08169700	-0.40506700
C	-2.32541100	-0.53506600	-0.13041400
C	-2.78502300	0.80910800	-0.02388700
S	-3.64270300	-1.60664500	-0.49576800
C	-4.19978900	0.92688000	-0.26663500
C	-4.78123900	-0.27195500	-0.51696000
H	-4.74051000	1.86603600	-0.23319700
H	-5.82740400	-0.47517700	-0.71074600
C	-1.83093700	1.79430500	0.22362200
C	-0.45001600	1.41408500	0.57746300
S	-2.01344900	3.50520100	0.08053600
C	0.41512200	2.62505100	0.51018900
H	-0.47456100	1.11820700	1.68155600
C	-0.27493500	3.76026800	0.32602900
H	1.48328900	2.57923200	0.67902600
H	0.10312000	4.77542800	0.30195300

1a-TS3

Br	-1.09113600	-2.66920000	0.73076100
O	0.74445700	1.15817600	2.67339400
C	2.17787400	-0.46991100	-0.29465900
C	1.19713700	0.03448600	0.63911400
C	1.82222200	-1.09601600	-1.51209200
H	0.77221000	-1.20500700	-1.76346600
C	-1.21328800	-0.81821000	0.28877900
C	-0.17308100	0.01453400	0.36557900
C	1.59656300	0.69166400	1.87901200
C	2.79452600	-1.57284700	-2.37886200
H	2.49725000	-2.04870700	-3.30972600
C	3.56385600	-0.34321500	0.02644000
C	4.15857200	-1.44901400	-2.05943900
H	4.91524700	-1.82843600	-2.74056100
C	3.94533900	0.30589000	1.25645500
H	5.00853500	0.39265000	1.47383100
C	3.02972800	0.80849000	2.12500200
H	3.32725200	1.30182900	3.04623200
C	4.53168100	-0.84217400	-0.86833700
H	5.58374800	-0.74215800	-0.61042300
C	-2.47506300	-0.23109400	-0.11727200
C	-2.40102400	1.12337100	-0.34148000
S	-4.07732000	-0.84000900	-0.33324600
C	-3.65042400	1.70326100	-0.70044900
C	-4.64797300	0.76325800	-0.73744900
H	-3.80180000	2.75381200	-0.92136300
H	-5.69379600	0.90836300	-0.97763600
C	-1.03019200	1.65970800	-0.15826600
C	-0.65524900	2.59200800	0.86572000
S	-0.06883100	2.03949900	-1.64937400
C	0.50392300	3.30415500	0.55782500
H	-1.19473600	2.65331000	1.80017700
C	0.96957900	3.04988300	-0.72280900
H	1.02169200	3.95202000	1.25513800
H	1.86866700	3.45772100	-1.17093300

1a-VQM

Br	-2.07639100	-2.63315100	0.31407700
O	0.78102100	-0.19647600	2.95937400
C	2.22566600	-0.76685400	-0.41725400
C	1.20043800	-0.61958200	0.63348800
C	1.88982100	-1.03515900	-1.75208400
H	0.84483800	-1.14434900	-2.02677900
C	-1.37960800	-0.81117200	0.16405400
C	-0.09811000	-0.68406600	0.37761100
C	1.61174900	-0.32783500	2.06094500
C	2.88045100	-1.15668700	-2.72358800
H	2.60054000	-1.36220100	-3.75298500
C	3.59046100	-0.62298200	-0.06456200
C	4.22997900	-1.01313500	-2.37762500
H	5.00196600	-1.10684200	-3.13609300
C	3.95728000	-0.34395500	1.31321100
H	5.01849900	-0.24531800	1.53352600
C	3.04892800	-0.20298600	2.30477800
H	3.34432000	0.00770700	3.32821900

C	4.57828900	-0.75019900	-1.05628800
H	5.62302800	-0.63867800	-0.77677000
C	-2.33632300	0.23994500	-0.16839600
C	-2.07061200	1.60172200	-0.21716600
S	-3.99995800	-0.08895400	-0.59178700
C	-3.21283600	2.36643200	-0.60600900
C	-4.31863400	1.59614000	-0.83796600
H	-3.19505000	3.44687000	-0.69609300
H	-5.30980600	1.91855900	-1.13193500
C	-0.74620600	2.16055200	0.09893700
C	-0.21465300	2.44608000	1.33195900
S	0.44399500	2.42327100	-1.16426400
C	1.15003900	2.85867700	1.27440700
H	-0.77104100	2.31316900	2.25243000
C	1.64437300	2.88550900	-0.00256400
H	1.74101100	3.10972100	2.14840900
H	2.63964700	3.16171700	-0.32816900

3a-P1

Br	-0.64778500	-2.73145000	-0.69092000
O	0.98665600	-2.22254600	2.05822000
C	-2.41298700	-0.54300500	-0.03618700
C	2.39349800	0.23147400	-0.28709900
C	1.40841100	-0.53822300	0.41906900
C	-2.72578200	0.86483200	0.11299200
C	2.04394200	1.23336400	-1.23228400
H	0.99694800	1.39512900	-1.47170900
C	-1.03956100	-0.98485500	0.01586300
C	0.02072700	-0.17955100	0.35639400
C	-4.05883600	1.32121300	-0.10923300
H	-4.27884300	2.37959900	0.00198100
C	1.80318600	-1.60461200	1.34064400
C	3.01427900	1.97923700	-1.88572900
H	2.71310300	2.74555300	-2.59563400
C	3.78647200	-0.03693900	-0.08868800
C	-3.49045300	-1.41247600	-0.34270800
H	-3.29738700	-2.47307100	-0.44341100
C	4.37886000	1.73153700	-1.65766700
H	5.13564600	2.30832500	-2.18213900
C	-4.77631900	-0.94191300	-0.51367600
H	-5.57540400	-1.64377200	-0.73388300
C	4.16636700	-1.10893200	0.79603900
H	5.22861600	-1.31121500	0.92403300
C	3.24212300	-1.85921600	1.44786100
H	3.53404400	-2.66753300	2.11329600
C	4.75063600	0.72087500	-0.77928600
H	5.80324800	0.49804800	-0.61710400
C	-5.06704900	0.43929200	-0.40933800
H	-6.08312300	0.79446400	-0.55120400
C	-1.68000700	1.73230500	0.45199500
C	-0.39626500	1.19213400	0.95521400
S	-1.65826600	3.45586700	0.34165000
C	0.56561400	2.32478900	1.11621000
H	-0.62325100	0.83708400	1.98649400
C	0.02159700	3.52958900	0.89005300
H	1.57742600	2.17733100	1.46794400
H	0.48971500	4.50177800	0.98873500

3a-P2

Br	-0.04359500	-2.70162600	-0.16575600
O	1.01966900	-0.84869500	2.82252700
C	-2.26628500	-0.83196400	-0.24665700
C	2.38570400	0.32915600	-0.41697500
C	1.42688300	-0.03358200	0.58084800
C	-2.85775600	0.47441100	-0.15283400
C	2.00447000	0.78422500	-1.70968900
H	0.94986500	0.85266100	-1.95711400
C	-0.85371300	-0.96336900	-0.00433500
C	-0.03670600	0.10017500	0.32337100
C	-4.24675100	0.62528500	-0.39311000
H	-4.69265500	1.61381100	-0.32117400
C	1.86458600	-0.48239500	1.81940000
C	2.95459700	1.13030800	-2.64730600
H	2.64075000	1.47455900	-3.62937800
C	3.78485300	0.22721000	-0.11703700
C	-3.10938100	-1.92316900	-0.58073900
H	-2.68168900	-2.91556300	-0.65654400
C	4.33490200	1.03699600	-2.34666500
H	5.07184400	1.31308900	-3.09588000
C	-4.45809000	-1.74432900	-0.80924500
H	-5.07837100	-2.59911700	-1.06293500
C	4.18011000	-0.23692100	1.16807300
H	5.24059900	-0.31268400	1.39552000
C	3.24834300	-0.58512300	2.11285700
H	3.53876800	-0.94106100	3.09655400
C	4.73664500	0.59161200	-1.10578300
H	5.79396800	0.51003700	-0.86364000
C	-5.03393800	-0.45936800	-0.71582400
H	-6.09638900	-0.32488700	-0.89756400
C	-2.00594000	1.56524200	0.18271500
C	-0.63681500	1.39958300	0.41978000
S	-2.47279400	3.24732800	0.35698900
C	0.02279300	2.63714700	0.74180300
H	0.09855400	-0.75723700	2.51903000
C	-0.82808700	3.69715000	0.74335400
H	1.08181800	2.71117100	0.95622800
H	-0.59642800	4.73477800	0.95030200

3a-P3

Br	-0.13288600	-2.85006600	-0.60517100
O	-0.03762500	2.23720900	-1.61901700
C	2.20213900	-1.17587500	-0.02155000
C	-2.37427000	0.02043100	0.22614100
C	-1.14257400	0.53398100	-0.29635200
C	2.55680500	0.17482300	0.15618400
C	-2.42460500	-1.02769600	1.18337700
H	-1.49742700	-1.44541300	1.55801700
C	0.74516000	-1.23547600	-0.15811200
C	0.18393100	-0.00548900	-0.05000200
C	3.88486200	0.55325600	0.28721800
H	4.15140900	1.59648500	0.43247300
C	-1.16482500	1.67725600	-1.09679300
C	-3.63103800	-1.49801000	1.65827000
H	-3.64395500	-2.29735100	2.39465200
C	-3.60984700	0.61448300	-0.19537500
C	3.17885200	-2.16831400	-0.07962400
H	2.90436400	-3.20881100	-0.22529500
C	-4.85141100	-0.94376100	1.20413900
H	-5.79480600	-1.32848200	1.58177800
C	4.51874400	-1.78594300	0.04929800

H	5.29669000	-2.54347900	0.00852300
C	-3.57740500	1.72923300	-1.07886300
H	-4.51734800	2.17233600	-1.39819700
C	-2.38659300	2.26469800	-1.50429700
H	-2.34928400	3.12953300	-2.15902300
C	-4.83570300	0.09866200	0.30291600
H	-5.76501200	0.55225800	-0.03392000
C	4.87031300	-0.44252400	0.22963800
H	5.91704100	-0.16824900	0.32672100
C	1.29553200	1.00986000	0.15886800
C	1.25399700	2.09596600	-0.95920600
S	1.11362200	1.92806800	1.79102300
C	1.61132500	3.41568200	-0.33467600
H	1.93449600	1.80274300	-1.76244500
C	1.52751100	3.45113800	0.99926000
H	1.83264500	4.27905600	-0.95289100
H	1.67986600	4.32663200	1.62362300

3a-TS1

Br	-0.86371300	-2.63387000	-0.99725000
O	0.85042100	-2.28268200	2.07951500
C	-2.46284400	-0.43809000	0.06326000
C	2.44238900	0.11069300	-0.23778300
C	1.39420800	-0.63165400	0.43438400
C	-2.69604600	0.96115200	0.25713900
C	2.16103100	1.11777100	-1.18584400
H	1.12625000	1.34119300	-1.42749700
C	-1.09251600	-0.94184800	-0.05836000
C	0.05975300	-0.34143000	0.25356900
C	-4.00791300	1.47162600	0.17887700
H	-4.16487600	2.53746500	0.32064300
C	1.72047800	-1.67973400	1.43074400
C	3.18563200	1.81390300	-1.81578900
H	2.94290200	2.58805500	-2.53900500
C	3.80517800	-0.19291100	0.04441800
C	-3.58572400	-1.26997300	-0.11759700
H	-3.44109400	-2.33558900	-0.24843800
C	4.52725100	1.51795800	-1.52836400
H	5.32615700	2.06088600	-2.02561600
C	-4.87387300	-0.75270800	-0.14310100
H	-5.71614400	-1.42556600	-0.27491400
C	4.11150600	-1.24260000	0.99215400
H	5.16162400	-1.45654200	1.18326900
C	3.14749200	-1.94900200	1.63019600
H	3.39145200	-2.73224500	2.34251900
C	4.82742000	0.51822000	-0.60987700
H	5.86309600	0.27167500	-0.38712500
C	-5.08947700	0.62715200	-0.01343000
H	-6.09684100	1.03134000	-0.04553100
C	-1.56637100	1.80742600	0.51727200
C	-0.42069400	1.36070700	1.21150800
S	-1.33745900	3.40531500	-0.12719200
C	0.60236400	2.39171500	1.26518500
H	-0.55789000	0.68762800	2.05678000
C	0.24747500	3.52445800	0.61172900
H	1.54729900	2.26346700	1.77873600
H	0.82367600	4.43311000	0.48714000

3a-TS2

Br	-0.33742300	-2.75868400	-0.47031100
O	0.74863400	-0.87640300	2.69565100
C	-2.36603700	-0.69324200	-0.14921500
C	2.44798000	0.15733900	-0.36308200
C	1.41606100	-0.29079500	0.50402300
C	-2.82874200	0.66340100	0.01451600
C	2.18635100	0.73167600	-1.63896600
H	1.15575200	0.81893800	-1.97436600
C	-0.94239300	-0.97937500	-0.06595300
C	0.00800200	-0.05671500	0.23247200
C	-4.20711900	0.96344300	-0.15193500
H	-4.54578600	1.98808300	-0.02549400
C	1.68132700	-0.72908500	1.85313400
C	3.21952300	1.15884000	-2.45343300
H	2.99501000	1.60122800	-3.42103700
C	3.81571400	0.01965900	0.05143500
C	-3.33139400	-1.67879900	-0.44041200
H	-3.01661100	-2.70754300	-0.56382200
C	4.56278500	1.01044600	-2.04657700
H	5.36862200	1.33466900	-2.69941500
C	-4.67397300	-1.36061500	-0.56975000
H	-5.38908500	-2.14926000	-0.78425900
C	4.08758900	-0.53741000	1.34644100
H	5.12861000	-0.65402100	1.64367500
C	3.08722900	-0.89217200	2.20263000
H	3.30296000	-1.28130000	3.19459500
C	4.84683600	0.44237700	-0.81408300
H	5.87954700	0.32193400	-0.49254600
C	-5.11894600	-0.03104800	-0.43038600
H	-6.17292200	0.20716700	-0.53455600
C	-1.86883500	1.65823900	0.29998800
C	-0.50771400	1.30010100	0.72275900
S	-2.05220100	3.36153200	0.10577400
C	0.34550000	2.52252800	0.70493700
H	-0.57154300	0.97575800	1.80411400
C	-0.34328000	3.64854000	0.46794300
H	1.40147000	2.49218300	0.93976400
H	0.02115200	4.66882200	0.45772000

3a-TS3

Br	0.93973000	2.78848400	0.67185100
O	-0.70583900	-0.99902500	2.74767500
C	2.51732900	0.43385900	-0.09861600
C	-2.17320000	0.36828200	-0.33831700
C	-1.18022800	-0.02641700	0.63828100
C	2.49663400	-0.96006500	-0.28888400
C	-1.83230900	0.93735200	-1.58637800
H	-0.78577800	1.08243300	-1.83378400
C	1.19431700	0.92891800	0.28391400
C	0.18273700	0.07458600	0.38584500
C	3.66102400	-1.64496700	-0.62406000
H	3.63701900	-2.72128900	-0.76857200
C	-1.56448400	-0.63091900	1.91393800
C	-2.81554300	1.31517500	-2.48990500
H	-2.52900700	1.74950400	-3.44414200
C	-3.55424100	0.18998500	-0.02574800
C	3.70519000	1.14851100	-0.25074200
H	3.72128900	2.22338400	-0.10216200
C	-4.17536400	1.14373300	-2.17771700
H	-4.94101300	1.44631700	-2.88668100
C	4.87188800	0.45758200	-0.59141800
H	5.80171700	1.00636000	-0.71202900

C	-3.92055600	-0.40333600	1.23788700
H	-4.98129700	-0.53065500	1.44690800
C	-2.99534800	-0.80559100	2.14650300
H	-3.28252200	-1.25728700	3.09204200
C	-4.53367500	0.58906800	-0.95658100
H	-5.58280000	0.45216500	-0.70370700
C	4.85409000	-0.92823400	-0.77483500
H	5.76855500	-1.45379200	-1.03438000
C	1.15465500	-1.57715300	-0.09801200
C	0.81516700	-2.46536100	0.96449600
S	0.21419700	-2.04947200	-1.56273500
C	-0.31560000	-3.23940600	0.69061400
H	1.34274200	-2.44868800	1.90796100
C	-0.78648900	-3.06589100	-0.60009400
H	-0.80918200	-3.87262300	1.41830100
H	-1.66667100	-3.52727900	-1.03323500

3a-VQM

Br	-1.61937400	-2.85976300	0.32692000
O	0.82228700	-0.11586200	2.94303200
C	-2.47634300	-0.06667000	-0.17799000
C	2.23703200	-0.53727600	-0.46504300
C	1.22754900	-0.46042100	0.60581600
C	-2.32479700	1.34529600	-0.18743900
C	1.88974900	-0.78418700	-1.80192500
H	0.84435000	-0.91444400	-2.06491100
C	-1.31864100	-0.92457900	0.15806500
C	-0.07186100	-0.60261400	0.36798300
C	-3.41779600	2.15222400	-0.52256200
H	-3.28391500	3.23010200	-0.52413900
C	1.64498600	-0.18804400	2.02821200
C	2.86930900	-0.85894100	-2.78862400
H	2.58036800	-1.04988500	-3.81841800
C	3.60287000	-0.36166800	-0.12889600
C	-3.72986500	-0.60896400	-0.49945400
H	-3.86679100	-1.68341100	-0.49666400
C	4.21998900	-0.68755900	-2.45822800
H	4.98334700	-0.74590600	-3.22893300
C	-4.80796100	0.21099000	-0.82917300
H	-5.76463500	-0.24082600	-1.07542100
C	3.97956100	-0.10179800	1.24919500
H	5.03986100	0.02786200	1.45790200
C	3.08007900	-0.01412300	2.25567300
H	3.38269000	0.18344100	3.27970500
C	4.57957300	-0.44196400	-1.13702500
H	5.62484800	-0.30798700	-0.86926200
C	-4.65711800	1.59677400	-0.84248000
H	-5.49335100	2.24141300	-1.09742800
C	-1.03428800	1.98296700	0.15200100
C	-0.54520700	2.31038800	1.39328200
S	0.11257300	2.42347800	-1.10211000
C	0.76409500	2.87195900	1.35020800
H	-1.08814300	2.10275300	2.30775100
C	1.25554200	2.97998500	0.07582400
H	1.32420100	3.16311200	2.23195100
H	2.21705300	3.36610100	-0.23934800

8-P1

Br	-1.40923700	-2.79076600	-0.78594000
O	0.24558500	-2.40136400	1.81407100
C	1.80872000	0.24423800	-0.21084400
C	0.75817400	-0.57609300	0.35514200
C	1.53865900	1.29214500	-1.12539400
H	0.51778800	1.46217800	-1.45344000
C	-1.68720300	-1.01020900	-0.11909100
C	-0.59974000	-0.19421400	0.24904000
C	1.09971800	-1.74567800	1.18917300
C	2.55922500	2.07967100	-1.64422700
H	2.31666800	2.88618700	-2.33124300
C	3.17695100	-0.05118400	0.07391100
C	3.89680000	1.81937000	-1.31046200
H	4.69459700	2.42824900	-1.72645500
C	3.49203100	-1.21071200	0.87577900
H	4.54216100	-1.42703700	1.06442900
C	2.52758500	-2.02651500	1.36404000
H	2.76859600	-2.90283800	1.95949600
C	4.19523600	0.74790800	-0.47598200
H	5.23042900	0.50653300	-0.24522000
C	-2.97661800	-0.50942400	-0.31798500
C	-3.30057600	0.86185200	-0.21544200
C	-2.32984100	1.72402000	0.21959500
C	-1.05838900	1.18155900	0.79065700
S	-2.35068900	3.46517600	0.20759200
C	-0.13257100	2.33195400	1.03955200
H	-1.35376100	0.82252800	1.80412000
C	-0.69279900	3.53180700	0.82903300
H	0.85865000	2.19899400	1.45050500
H	-0.24507500	4.50455500	0.99710000
H	-3.73994200	-1.18368900	-0.69092600
H	-4.27265200	1.20627700	-0.55347400

8-P2

Br	-0.13882600	2.88250800	-0.65721900
O	-0.38334100	1.22755000	2.61592000
C	-1.69121200	-0.76197200	-0.22661700
C	-0.77455600	-0.00592700	0.57123800
C	-1.33292800	-1.30325000	-1.49247600
H	-0.33064300	-1.13619500	-1.87412600
C	1.08445800	1.42381600	-0.41409700
C	0.63213700	0.21777000	0.12885600
C	-1.19194700	0.50315600	1.79386900
C	-2.24042500	-2.02889500	-2.23532500
H	-1.94505100	-2.43173600	-3.20066100
C	-3.02465400	-0.98283800	0.25415000
C	-3.55375700	-2.25158900	-1.75596700
H	-4.25797300	-2.82511100	-2.35246300
C	-3.40106400	-0.43805500	1.51294400
H	-4.41213700	-0.60517700	1.87597900
C	-2.51211900	0.28743400	2.26495800
H	-2.78797500	0.70666300	3.22760300
C	-3.93366900	-1.73534800	-0.53588800
H	-4.94099300	-1.89485400	-0.15771500
C	2.41809000	1.62768800	-0.81222500
C	3.35025700	0.60775700	-0.67880800
C	2.92883400	-0.61289000	-0.14316700
C	1.58820500	-0.82255000	0.26408200
S	3.90475200	-2.04826600	0.12604300
C	1.38377800	-2.15092100	0.78483000
H	0.49982300	1.30623400	2.21263200
C	2.51569800	-2.90130100	0.76898200

H	0.42767800	-2.51013500	1.14515100
H	2.63144900	-3.92586000	1.10134700
H	2.71543100	2.58401900	-1.22722300
H	4.37926700	0.76373200	-0.98795300

8-P3

Br	1.12324600	2.83405400	-0.57095100
O	-1.62697200	-1.57306800	-1.48482400
C	1.60250200	-0.82741300	0.19640200
C	0.25919100	-0.63439400	-0.26990000
C	2.22849000	0.05765400	1.11382500
H	1.66710900	0.89902400	1.50231200
C	-0.43463500	1.86698800	-0.09620500
C	-0.57704100	0.52014900	0.00135200
C	-0.34876100	-1.64282900	-1.02182700
C	3.52435300	-0.15561100	1.53571400
H	3.97969500	0.53455200	2.24110300
C	2.33220000	-1.98506300	-0.23210100
C	4.26207500	-1.26864300	1.06836600
H	5.28494800	-1.42016200	1.40221400
C	1.68646000	-2.94251500	-1.06346600
H	2.24386000	-3.81828300	-1.38594400
C	0.37360700	-2.79017500	-1.43268200
H	-0.13737000	-3.52581900	-2.04571800
C	3.66929300	-2.16886600	0.20913800
H	4.21396300	-3.04508800	-0.13521400
C	-1.72237300	2.55155000	0.06975300
C	-2.68727200	1.62731400	0.23100000
C	-2.05558800	0.25827600	0.20874900
C	-2.60490600	-0.65579600	-0.91781500
S	-2.43554300	-0.61992000	1.82445300
C	-3.71029000	-1.48562400	-0.32190500
H	-2.92954000	-0.03234200	-1.75629400
C	-3.68473900	-1.57184000	1.01239700
H	-4.39779700	-2.03673300	-0.95439900
H	-4.34545700	-2.17697700	1.62601700
H	-1.83667900	3.62869200	0.04652100
H	-3.74727200	1.78554700	0.38526500

8-TS1

Br	-1.76701800	-2.61590800	-0.95613000
O	0.15650600	-2.51323500	1.80875000
C	1.87937100	0.05200700	-0.22709100
C	0.77911100	-0.70763800	0.35773300
C	1.66042800	1.13202300	-1.10503600
H	0.64289700	1.40185200	-1.36856800
C	-1.74340400	-0.90064500	-0.02387200
C	-0.52420900	-0.35884300	0.17469500
C	1.05672300	-1.87707300	1.24784100
C	2.72746000	1.84264600	-1.64592300
H	2.53186800	2.67578100	-2.31569900
C	3.21974500	-0.32240600	0.06612000
C	4.04708300	1.48369900	-1.33846900
H	4.87898800	2.03708400	-1.76503900
C	3.46767000	-1.47026000	0.91594600
H	4.50532200	-1.73434400	1.11172900
C	2.46937700	-2.21066000	1.45118800
H	2.67127800	-3.07073400	2.08316100
C	4.28507700	0.40290600	-0.49560400
H	5.30473000	0.10346500	-0.26472200
C	-3.03722700	-0.30635400	0.11579700
C	-3.25502200	1.04437800	0.27819300
C	-2.17548400	1.91784100	0.51863600
C	-0.96324400	1.49830600	1.12188900
S	-2.05706900	3.56642700	-0.05860400
C	0.02519800	2.55432500	1.12783600
H	-1.01840400	0.79304800	1.94626500
C	-0.41685400	3.69635400	0.54032500
H	1.00602700	2.45163800	1.57518500
H	0.12185400	4.62746100	0.41129000
H	-3.89309100	-0.94421400	-0.07632100
H	-4.25558000	1.43513400	0.11373600

8-TS2

Br	0.35634200	3.02070300	-0.59300100
O	0.09070200	0.74458300	2.55327800
C	-1.85073900	-0.46527500	-0.28025400
C	-0.80996500	0.17721200	0.44348200
C	-1.68586600	-0.93699900	-1.61236600
H	-0.72678000	-0.79522200	-2.10527500
C	1.28405700	1.37084300	-0.32303200
C	0.56884100	0.23322300	-0.00251200
C	-0.93486600	0.49272400	1.84480400
C	-2.72715200	-1.55183200	-2.28247900
H	-2.57636600	-1.91265800	-3.29706100
C	-3.13365000	-0.62158500	0.34324000
C	-3.99069400	-1.69752700	-1.66855500
H	-4.80740400	-2.16742500	-2.20961500
C	-3.30282500	-0.16067600	1.69065500
H	-4.28627200	-0.26296700	2.14644200
C	-2.26807200	0.36413100	2.40943600
H	-2.39549800	0.67247200	3.44379800
C	-4.18422500	-1.22925200	-0.37963800
H	-5.15510900	-1.33247300	0.10072900
C	2.68142800	1.36691200	-0.53826200
C	3.45115700	0.21065000	-0.41681300
C	2.81980200	-0.96306100	-0.03242700
C	1.41303200	-0.94721600	0.40240600
S	3.45718500	-2.57299500	-0.07970300
C	0.90986300	-2.34295100	0.49035400
H	1.40972700	-0.54895700	1.49089100
C	1.87500200	-3.26104300	0.31901100
H	-0.11752100	-2.57280000	0.74172700
H	1.78434200	-4.33882200	0.38709600
H	3.16426100	2.28491600	-0.85569400
H	4.50330300	0.23089800	-0.68127900

8-TS3

Br	-2.19049700	-2.36497800	0.44052800
O	-0.16348100	1.23843900	2.56735400
C	1.50889900	-0.63352500	-0.12339600
C	0.45958800	0.00927500	0.63834000
C	1.27527500	-1.25180700	-1.37308700
H	0.27195700	-1.24532100	-1.78660700
C	-1.96890300	-0.53441200	-0.07610500
C	-0.83937700	0.12714200	0.15367400
C	0.73670200	0.65461800	1.92087800
C	2.30646600	-1.86814800	-2.06748900
H	2.10277800	-2.33677700	-3.02683900
C	2.83168600	-0.65972700	0.41215200
C	3.60803600	-1.89535300	-1.53668600
H	4.41058900	-2.38345300	-2.08263700

C	3.09135100	-0.02228000	1.68044900
H	4.11060900	-0.05298300	2.06138700
C	2.11913600	0.60861000	2.38796400
H	2.32705600	1.09155400	3.33886400
C	3.86021500	-1.29779300	-0.30935000
H	4.86305600	-1.31282500	0.11165600
C	-3.08004400	0.17076300	-0.71439300
C	-2.79134200	1.45456000	-0.97321700
C	-1.41407000	1.85224500	-0.58050200
C	-1.09835700	2.76332400	0.47342700
S	-0.18072300	2.04045000	-1.88695600
C	0.17906400	3.31620800	0.35485100
H	-1.76499600	2.91341200	1.31116200
C	0.81191000	2.96159700	-0.82487300
H	0.65287000	3.91962200	1.11996700
H	1.81526400	3.23949500	-1.12707400
H	-4.02753000	-0.31372400	-0.91850400
H	-3.47224500	2.16872900	-1.42320300

8-VQM

Br	-3.29865000	-1.61254700	0.11407700
O	-0.20248000	0.46620000	2.84428300
C	1.42392100	-1.09312700	-0.11113200
C	0.36245900	-0.49032200	0.71715000
C	1.18161100	-1.54975900	-1.41472100
H	0.18181800	-1.46712600	-1.83048200
C	-2.08365900	-0.10330700	-0.16639800
C	-0.86098700	-0.27026600	0.25659600
C	0.66449800	-0.03131900	2.12573000
C	2.20855200	-2.10099400	-2.17695200
H	2.00169300	-2.44724900	-3.18566900
C	2.72948100	-1.20541500	0.42820000
C	3.50142300	-2.20721400	-1.64873500
H	4.30210000	-2.63605800	-2.24446900
C	2.99546500	-0.74557000	1.78027400
H	4.01287100	-0.85506300	2.15055700
C	2.04673900	-0.20081600	2.57512400
H	2.26705700	0.13434600	3.58428400
C	3.75527200	-1.76308000	-0.35479500
H	4.75445000	-1.84389200	0.06600300
C	-2.69279400	1.05651200	-0.83058200
C	-2.06823300	2.22791600	-1.04669700
C	-0.68320500	2.49240500	-0.62182400
C	-0.23144700	2.90702000	0.60671000
S	0.66638600	2.16232900	-1.69655900
C	1.19212000	2.93617600	0.70346200
H	-0.90040900	3.13086100	1.42960400
C	1.81231600	2.54998400	-0.45445500
H	1.72961500	3.21651600	1.60274400
H	2.87441100	2.48231700	-0.65529200
H	-3.72054400	0.92900700	-1.15692700
H	-2.61383800	3.01570100	-1.56409400

1v-P1

Br	-0.86304700	-2.75957600	-0.66103600
O	0.85629200	-2.33205700	1.92194200
C	2.33267700	0.24320100	-0.25591000
C	1.31019100	-0.55018600	0.39010700
C	2.02158500	1.26946900	-1.18337000
H	0.98475600	1.44668200	-1.45250000
C	-1.14937800	-0.98717000	0.02163600
C	-0.05367100	-0.16233800	0.33720300
C	1.68264000	-1.69408900	1.24038300
C	3.01788900	2.02629300	-1.78613100
H	2.74368800	2.81615000	-2.48078600
C	3.71302500	-0.05837500	-0.04182300
C	4.37126200	1.75705800	-1.52746900
H	5.14926100	2.34164000	-2.01050800
C	4.06096700	-1.19100100	0.78212500
H	5.11792900	-1.41313200	0.91981000
C	3.11650700	-1.97647000	1.35415300
H	3.38299800	-2.83356600	1.96675000
C	4.70608400	0.70904800	-0.67847400
H	5.75044200	0.46088900	-0.50163000
C	-2.45486100	-0.49822100	-0.08578200
C	-2.77398300	0.88250600	0.04788200
C	-4.20211300	1.14224800	-0.37151800
C	-4.84382100	-0.26239800	-0.28281500
H	-4.70193100	1.88462100	0.26030600
H	-5.23105700	-0.42147400	0.73003600
C	-1.78724400	1.75212900	0.40912800
C	-0.47575700	1.21824700	0.89254100
S	-1.82395300	3.49409700	0.38478800
C	0.45996400	2.37761600	1.05538000
H	-0.68630900	0.87054900	1.93051700
C	-0.12144200	3.57235600	0.87796900
H	1.48124700	2.25423800	1.38832000
H	0.33352500	4.54807800	1.00337200
C	-3.68185200	-1.24752600	-0.54698300
H	-3.79821100	-2.20839200	-0.03579000
H	-3.58106700	-1.47568400	-1.61820700
H	-5.67332700	-0.39376100	-0.98283700
H	-4.22437300	1.52483100	-1.40213000

1v-P2

Br	-0.14508200	-2.73417500	-0.30378600
O	0.86460200	-0.97973900	2.76726200
C	2.31266200	0.38451500	-0.35973300
C	1.32734500	-0.03990900	0.58742400
C	1.96595400	0.90769100	-1.63656200
H	0.91820600	0.98432500	-1.90899600
C	-0.93225700	-0.99532800	-0.09019200
C	-0.13040700	0.08724900	0.29638900
C	1.73568100	-0.55460400	1.81064600
C	2.93966900	1.31185400	-2.52549500
H	2.65109200	1.70755700	-3.49590500
C	3.70372700	0.27665400	-0.02532900
C	4.31155900	1.21331200	-2.18956000
H	5.06763100	1.53719900	-2.89962400
C	4.06658300	-0.25594400	1.24256200
H	5.12071000	-0.33695300	1.49615100
C	3.11105500	-0.66423500	2.13843400
H	3.37624400	-1.07267700	3.10876600
C	4.68071100	0.70364300	-0.96346200
H	5.73122900	0.61823900	-0.69438900
C	-2.30449700	-0.85636600	-0.34470000
C	-2.91504200	0.39445100	-0.23652700
C	-4.37669300	0.31866500	-0.60510600
C	-4.67110700	-1.20137900	-0.54526000
H	-5.01619900	0.90558900	0.06446800
H	-5.03060700	-1.45861500	0.45761700

C	-2.14414900	1.49323100	0.13683100
C	-0.76060900	1.35339400	0.41365400
S	-2.67234200	3.15631100	0.34103000
C	-0.16048000	2.60996300	0.78954000
H	-0.04708600	-0.86070400	2.44515100
C	-1.04653700	3.63880600	0.79189900
H	0.88813400	2.71815800	1.03888800
H	-0.85088600	4.67659100	1.03349600
C	-3.30746800	-1.89628100	-0.79005600
H	-3.20679700	-2.84069600	-0.24570000
H	-3.15666300	-2.12845400	-1.85388800
H	-5.43681700	-1.51162000	-1.26189500
H	-4.52389500	0.71881000	-1.61858800

1v-P3

Br	0.17824800	2.88251600	-0.61534500
O	-0.15132400	-2.31064500	-1.51614400
C	2.30499800	-0.11747500	0.20701000
C	1.04038200	-0.58123200	-0.28897800
C	2.41797000	0.95874700	1.12706700
H	1.51721400	1.43839200	1.49080300
C	-0.75289900	1.30014200	-0.14957200
C	-0.24777600	0.03974900	-0.04011300
C	0.99861000	-1.76333400	-1.03278100
C	3.64998400	1.37861000	1.58373700
H	3.70865300	2.20123700	2.29172200
C	3.50423200	-0.79972200	-0.18503700
C	4.83631100	0.74118000	1.14984900
H	5.80111300	1.08712000	1.51070900
C	3.40697200	-1.95070500	-1.01566300
H	4.31894000	-2.46190700	-1.31362300
C	2.18582700	-2.43654500	-1.41157500
H	2.09631400	-3.32943900	-2.02220100
C	4.75865600	-0.33280200	0.28964200
H	5.65965500	-0.85233000	-0.02876800
C	-2.20686600	1.28339200	-0.01032900
C	-2.62205800	0.01330200	0.14791500
C	-4.10002700	-0.09711100	0.37339400
C	-4.57942500	1.34160900	-0.01256500
H	-4.58344000	-0.87408700	-0.23109100
H	-4.95004200	1.32739400	-1.04324700
C	-1.43225600	-0.90395600	0.15562800
C	-1.45648300	-1.97552600	-0.96466900
S	-1.35918100	-1.84559900	1.77691000
C	-2.03472400	-3.23086000	-0.36886900
H	-2.03052700	-1.59025600	-1.81253600
C	-1.98645500	-3.28627600	0.96650300
H	-2.36496200	-4.04864700	-1.00030500
H	-2.28067000	-4.13194500	1.58106300
C	-3.32805700	2.27714600	0.06233900
H	-3.31218600	3.02097800	-0.74250900
H	-3.28712600	2.83431800	1.00909000
H	-5.39669500	1.69167400	0.62466100
H	-4.31894100	-0.33581400	1.42432400

1v-ts1

Br	-1.08382500	-2.67069800	-0.99425200
O	0.76272000	-2.48983300	1.82904400
C	2.42459900	0.11067000	-0.21416100
C	1.34234500	-0.67979600	0.36429400
C	2.18142600	1.16302900	-1.11908900
H	1.15906600	1.38667900	-1.40621700
C	-1.16643600	-0.96205200	-0.04388600
C	0.03129700	-0.37286200	0.15964300
C	1.64693900	-1.82421500	1.27570000
C	3.22986700	1.90546700	-1.65305000
H	3.01500800	2.71623900	-2.34413700
C	3.77274700	-0.20357300	0.11248100
C	4.55653500	1.60745500	-1.31108000
H	5.37417800	2.18602700	-1.73185900
C	4.04753800	-1.32415400	0.98936800
H	5.09032600	-1.54249600	1.21282100
C	3.06763100	-2.09495100	1.51614200
H	3.29019400	-2.93489800	2.16809100
C	4.81974900	0.55456100	-0.44123800
H	5.84536000	0.30130200	-0.18289700
C	-2.47921800	-0.42231900	0.12106700
C	-2.74193600	0.92972500	0.29336900
C	-4.20728800	1.22662500	0.06072000
C	-4.86364800	-0.15658100	0.26499300
H	-4.59567400	1.99697000	0.73567500
H	-5.10416800	-0.28877100	1.32614900
C	-1.70564800	1.85440300	0.50303600
C	-0.46657400	1.49868700	1.09011800
S	-1.67780000	3.50373900	-0.08946900
C	0.47264200	2.59857400	1.06443900
H	-0.47132300	0.79973600	1.92083300
C	-0.03108800	3.71288400	0.47386200
H	1.46474600	2.54622900	1.49536300
H	0.46229900	4.66612100	0.32624900
C	-3.77185200	-1.16810700	-0.14550000
H	-3.83139800	-2.11632200	0.39882400
H	-3.83606000	-1.41652600	-1.21426500
H	-5.78812700	-0.27805000	-0.30647400
H	-4.35528100	1.59023300	-0.96755100

1v-TS2

Br	-0.39146500	-2.80967000	-0.36240700
O	0.51565100	-0.49924100	2.69979600
C	2.40145200	0.16551400	-0.35179900
C	1.32293300	-0.20517800	0.49443900
C	2.21766300	0.57923600	-1.70165500
H	1.21045200	0.60335700	-2.11133700
C	-1.00085800	-1.01589800	-0.09185100
C	-0.07452200	-0.01801400	0.14040700
C	1.50253300	-0.47253500	1.89851200
C	3.29610800	0.92960500	-2.49199000
H	3.13009400	1.25000400	-3.51784000
C	3.74116200	0.09645000	0.15952800
C	4.61322800	0.85734600	-1.98610600
H	5.45523400	1.12054800	-2.62045600
C	3.93308600	-0.31307300	1.51963000
H	4.95324500	-0.38556100	1.89333000
C	2.88135600	-0.58175900	2.34787000
H	3.03696900	-0.85593400	3.38831500
C	4.82274000	0.43812700	-0.68306600
H	5.83336700	0.37294400	-0.28459100
C	-2.38447700	-0.75301400	-0.21651700
C	-2.91215400	0.54567900	-0.11307100
C	-4.38486200	0.55867300	-0.44698300
C	-4.78024900	-0.93217900	-0.30975500

H	-4.96253600	1.21803100	0.20964700
H	-5.11398100	-1.12583900	0.71542700
C	-2.05494300	1.59213000	0.16003300
C	-0.64933600	1.32184500	0.51837100
S	-2.37714200	3.29185300	0.04360000
C	0.11606400	2.59787600	0.47541600
H	-0.64641500	1.00195100	1.62533200
C	-0.66392400	3.67570500	0.29927700
H	1.18328400	2.63632400	0.65210200
H	-0.36342300	4.71678700	0.29013900
C	-3.47759400	-1.72326600	-0.57758000
H	-3.40933400	-2.65675300	-0.01048500
H	-3.37240300	-1.99497000	-1.63884600
H	-5.59052100	-1.21732600	-0.98546000
H	-4.52610500	0.91955700	-1.47565700

1v-TS3

Br	-1.07772800	-2.75904800	0.69572500
O	0.54040100	1.18816000	2.66421700
C	2.08722300	-0.39901100	-0.26874600
C	1.07141600	0.06426900	0.64838600
C	1.77808300	-1.06274600	-1.47936700
H	0.73775300	-1.23184600	-1.73722300
C	-1.29352200	-0.91062500	0.26061500
C	-0.29302700	-0.03335300	0.35874700
C	1.42288800	0.75884100	1.88092200
C	2.78257100	-1.50200800	-2.32883700
H	2.52039400	-2.00891200	-3.25397300
C	3.46178100	-0.19421800	0.06214000
C	4.13528400	-1.30164700	-1.99911700
H	4.91769500	-1.65208700	-2.66662000
C	3.79591600	0.49470800	1.28375700
H	4.85061600	0.64367100	1.50879200
C	2.84532100	0.95949300	2.13594300
H	3.10636700	1.48358300	3.05132000
C	4.46358100	-0.65611200	-0.81546700
H	5.50626100	-0.49597200	-0.54972100
C	-2.59133500	-0.39504400	-0.15402900
C	-2.56833800	0.93044700	-0.36638300
C	-3.89626700	1.46633900	-0.82786100
C	-4.84313600	0.26037000	-0.53459900
H	-4.20025500	2.37857500	-0.30044300
H	-5.34484100	0.42738000	0.42452200
C	-1.23831600	1.55348800	-0.17860500
C	-0.91859000	2.52191100	0.83025700
S	-0.29010300	1.96474200	-1.67278100
C	0.19726900	3.29758900	0.51379200
H	-1.46153500	2.56626200	1.76371700
C	0.67863000	3.05648500	-0.76309200
H	0.67349700	3.98565800	1.20222600
H	1.54931300	3.51545200	-1.21789500
C	-3.93606000	-1.00485300	-0.41740100
H	-4.26402000	-1.68810300	0.37371000
H	-3.91505400	-1.58702100	-1.34949100
H	-5.61944100	0.14553100	-1.29631000
H	-3.86606200	1.71744100	-1.89853600

1v-VQM

Br	-2.12281600	-2.69114800	0.30948700
O	0.65140400	-0.13965900	2.94771000
C	2.12750400	-0.74599500	-0.40921700
C	1.09387600	-0.59897900	0.63344800
C	1.80409100	-1.03731500	-1.74254700
H	0.76200800	-1.16151300	-2.02211500
C	-1.47625900	-0.84946700	0.13352700
C	-0.20164300	-0.69638700	0.36907400
C	1.48951200	-0.27534500	2.05576900
C	2.80251500	-1.16310300	-2.70524600
H	2.53179500	-1.38692500	-3.73336100
C	3.48852800	-0.58141400	-0.05030800
C	4.14843300	-1.00090700	-2.35258900
H	4.92637900	-1.09851000	-3.10447800
C	3.84119000	-0.27273400	1.32468700
H	4.89931200	-0.15553400	1.55102800
C	2.92338300	-0.12527800	2.30686500
H	3.20885600	0.10921000	3.32800800
C	4.48462500	-0.71352700	-1.03347900
H	5.52614700	-0.58599600	-0.74868600
C	-2.46877600	0.16363700	-0.24398200
C	-2.21647400	1.49014100	-0.28174300
C	-3.41622300	2.28628600	-0.73973900
C	-4.52152100	1.22139900	-0.98145100
H	-3.69712100	3.02674200	0.02144000
H	-5.39184800	1.41194200	-0.34703500
C	-0.93236100	2.11003000	0.07134800
C	-0.44613400	2.41793200	1.31795800
S	0.26649100	2.46918600	-1.16267700
C	0.89036400	2.91830000	1.29597200
H	-1.01193200	2.23857400	2.22473500
C	1.40993900	2.99444600	0.03192800
H	1.44521600	3.19281600	2.18658800
H	2.39275700	3.33555200	-0.26913000
C	-3.89029000	-0.16063300	-0.64871800
H	-4.42003300	-0.67565300	0.16171900
H	-3.90754300	-0.84559600	-1.50561200
H	-4.87015000	1.24850600	-2.01781400
H	-3.17605400	2.86031000	-.64551000

3a-TS4

Br	1.93405200	-2.48533800	-0.57033700
O	-0.97314000	-3.19892000	-1.30247500
C	2.26826600	0.25183900	0.13053400
C	-2.42270500	-0.35085000	-0.04421200
C	-1.13770800	-0.93055300	-0.54097900
C	1.85930100	1.64496600	0.40315500
C	-3.21755400	0.62330500	-0.65578800
H	-2.98815200	0.95220700	-1.65563800
C	1.39036200	-0.63996100	-0.49384300
C	0.01189800	-0.22487400	-0.81119800
C	2.64829400	2.45884900	1.29312400
H	2.29589900	3.46058000	1.52620000
C	-1.14035200	-2.43117900	-0.36258200
C	-4.34082400	1.15561000	-0.01796700
H	-4.93326000	1.90921300	-0.52981400
C	-2.87132700	-0.88228100	1.20622400
C	3.56873900	-0.12561400	0.63795800
H	3.95473900	-1.10805300	0.39895700
C	-4.70663800	0.71664900	1.25485100
H	-5.56815400	1.14319800	1.76066600
C	4.30380100	0.70572900	1.43042500
H	5.27031600	0.37194600	1.79859000

C	-2.26300000	-2.09079700	1.75606500
H	-2.58123100	-2.40801400	2.74687000
C	-1.45719100	-2.87280800	1.00621300
H	-1.12504300	-3.85696200	1.32231400
C	-3.98017500	-0.31296200	1.84934600
H	-4.28808200	-0.71299500	2.81261400
C	3.82999000	2.01082400	1.79582200
H	4.42954300	2.63905100	2.44780000
C	0.76682600	2.11427100	-0.27794800
C	0.12789000	1.22719300	-1.30923200
S	0.03476500	3.70841100	-0.22476300
C	-0.95212300	2.01803300	-1.98183800
C	-1.05843200	3.28175300	-1.56302500
H	-1.49237700	1.62309300	-2.83379200
H	-1.71024500	4.05345700	-1.95671200
H	0.90908100	1.09405200	-2.09089400

TS-rot

Br	0.75396300	0.69829400	2.49069100
O	-0.67860000	-2.87628700	-0.33811400
C	1.24661500	1.34039300	-0.33953200
C	-3.07867000	-0.03654400	-0.20980200
C	-1.79006800	-0.75887800	-0.12316400
C	2.49909500	0.92063600	-0.86388600
C	-3.19337800	1.32800900	0.09245700
H	-2.31352000	1.88216200	0.40589100
C	0.38849500	0.53005600	0.55798400
C	-0.67161200	-0.13663400	0.22401100
C	3.19172200	1.86079900	-1.66198600
H	4.16098300	1.60061700	-2.06915900
C	-1.72644400	-2.23869900	-0.43000500
C	-4.42264900	1.97700500	0.00170400
H	-4.48992100	3.03462600	0.24083100
C	-4.23520200	-0.75280700	-0.60620200
C	0.73953600	2.61752700	-0.64060000
H	-0.22227200	2.90049300	-0.22380400
C	-5.56560300	1.27141500	-0.39267000
H	-6.52394100	1.77770000	-0.46367000
C	1.43909300	3.50950500	-1.44269400
H	1.02720200	4.48966800	-1.66370100
C	-4.13914900	-2.16965300	-0.91782100
H	-5.05430900	-2.67364100	-1.22261100
C	-2.98320200	-2.86636900	-0.84041600
H	-2.93531900	-3.92574700	-1.07350600
C	-5.46817200	-0.08395800	-0.69268900
H	-6.34975200	-0.64218500	-0.99750400
C	2.68186000	3.12054100	-1.94849600
H	3.25997300	3.80009500	-2.56873200
C	3.12065900	-0.40168000	-0.66147200
C	4.28541500	-0.85723400	-1.26014000
S	2.48102100	-1.66343700	0.38284700
C	4.66079100	-2.17199800	-0.88231700
H	4.86360500	-0.27501600	-1.96695200
C	3.78145700	-2.73263300	0.00663600
H	5.54264300	-2.67830500	-1.26002000
H	3.81487800	-3.71824900	0.45471600

3a-VQM-rot

Br	1.71077500	-2.82267800	0.40308400
O	-0.85519000	-0.15611200	2.97156900
C	2.45622100	-0.02584100	-0.23905700
C	-2.21425200	-0.60627200	-0.45461000
C	-1.21761300	-0.50014100	0.62566600
C	2.21740900	1.35503600	-0.47153100
C	-1.85221500	-0.92009000	-1.77353000
H	-0.80800800	-1.10193000	-2.00930700
C	1.33720000	-0.90718500	0.16396900
C	0.08385800	-0.62123700	0.38846200
C	3.27833600	2.18241100	-0.85572900
H	3.07692400	3.23593500	-1.02638700
C	-1.65466900	-0.19348100	2.03586900
C	-2.81546600	-0.99920400	-2.77613300
H	-2.51536400	-1.24416200	-3.79121000
C	-3.57909000	-0.37186000	-0.15214500
C	3.75969100	-0.51730100	-0.40845700
H	3.96407600	-1.56690700	-0.23607900
C	-4.16426600	-0.76459900	-2.47973800
H	-4.91515200	-0.82506800	-3.26242700
C	4.80387700	0.32182100	-0.79427300
H	5.80145900	-0.09077400	-0.91473800
C	-3.96977300	-0.03849100	1.20601100
H	-5.02755300	0.14088700	1.38888200
C	-3.08546600	0.05464200	2.22512600
H	-3.39857200	0.30017400	3.23553600
C	-4.53889100	-0.45563000	-1.17573400
H	-5.58325700	-0.27345500	-0.93435300
C	4.56821500	1.67689600	-1.02079600
H	5.37740300	2.33609300	-1.32164000
C	0.86882000	1.94153300	-0.31622700
C	-0.12743700	2.05314800	-1.25764800
S	0.34945200	2.61129900	1.21778800
C	-1.32595700	2.62372400	-0.74053200
H	-0.01387500	1.69203400	-2.27369400
C	-1.22449200	2.96016900	0.58510000
H	-2.23052200	2.76053900	-1.32281500
H	-1.97863200	3.41021000	1.21875000

3a-TS6

Br	1.14513500	2.80401800	-0.48364000
O	-0.71382200	-0.18472700	-2.87985800
C	2.53366300	0.32013500	0.23940000
C	-2.16868700	0.34050900	0.46104500
C	-1.17250100	0.16749600	-0.56769500
C	2.37858300	-1.06225600	0.44775900
C	-1.82519100	0.59320600	1.81026600
H	-0.77915700	0.70056400	2.07764500
C	1.25672500	0.92629700	-0.14202900
C	0.18880200	0.14013600	-0.24594600
C	3.46207200	-1.84789000	0.82776300
H	3.33455400	-2.91479600	0.98727100
C	-1.55422400	-0.06156500	-1.96684200
C	-2.80597900	0.73435800	2.78385900
H	-2.51714700	0.93050600	3.81324800
C	-3.55282800	0.25213800	0.11859800
C	3.77770100	0.92464700	0.41278900
H	3.89628600	1.99131000	0.25263400
C	-4.16485300	0.63716300	2.44376100
H	-4.92920300	0.75229900	3.20726300

C	4.86607300	0.13261000	0.79120100
H	5.84070000	0.59339000	0.92516600
C	-3.92003100	-0.00092000	-1.25379900
H	-4.98080300	-0.06959700	-1.48908400
C	-2.99251600	-0.15558600	-2.23172900
H	-3.27820600	-0.34469100	-3.26296500
C	-4.52709600	0.40461000	1.12167200
H	-5.57738000	0.33700400	0.84667100
C	4.71299200	-1.24189900	0.99737500
H	5.56751600	-1.84498200	1.29035400
C	0.97786900	-1.52983500	0.24270000
C	0.12662300	-1.96282600	1.31517300
S	0.59473200	-2.52388600	-1.21355500
C	-0.95140300	-2.71653200	0.87455100
H	0.26367400	-1.59879800	2.32579600
C	-0.86860000	-3.02939200	-0.48433900
H	-1.79137200	-3.00974200	1.49357000
H	-1.59746900	-3.59073300	-1.05763600

3a-P4

Br	-0.36060800	-2.76824300	-0.81918000
O	0.47877000	1.52154000	-2.37807900
C	-2.39012900	-0.84075800	0.07354900
C	2.22320400	-0.21015400	0.33367300
C	1.17478900	0.25412200	-0.51090100
C	-2.52070700	0.51820800	0.40493600
C	1.98697300	-0.95846900	1.52166700
H	0.96554200	-1.20556700	1.79360500
C	-0.98839900	-1.07259600	-0.27131100
C	-0.22887700	0.04269100	-0.19447500
C	-3.74431300	1.05428700	0.77677000
H	-3.83812000	2.10443100	1.03898400
C	1.43536600	1.06039500	-1.65910600
C	3.03378000	-1.38437500	2.31680400
H	2.82711500	-1.95677600	3.21786800
C	3.58378300	0.09807000	-0.01102000
C	-3.49412800	-1.68860000	0.10226100
H	-3.39101900	-2.73930700	-0.15088100
C	4.36968600	-1.08809200	1.96732800
H	5.18577000	-1.43205500	2.59703000
C	-4.73387100	-1.14823900	0.46297000
H	-5.61002500	-1.79013000	0.48511800
C	3.83834800	0.88133800	-1.18235600
H	4.87289700	1.10756900	-1.43454300
C	2.82156700	1.35729600	-1.95964400
H	3.01573400	1.96557200	-2.83919700
C	4.63144000	-0.35895100	0.82038100
H	5.65668200	-0.12148600	0.54385700
C	-4.86058600	0.20557900	0.79505600
H	-5.83285500	0.60488800	1.06874700
C	-1.14985700	1.17331500	0.34145800
C	-0.66084900	1.62973100	1.67898700
S	-1.08042400	2.69795000	-0.74663900
C	-0.18281300	2.90063300	1.70965300
H	-0.65357100	0.92761800	2.50421100
C	-0.36427000	3.58983800	0.47542900
H	0.26481700	3.37592400	2.57513200
H	-0.06589500	4.61671400	0.28955700

3a-TS7

Br	0.53879200	-2.43551700	-1.18018000
O	0.20479900	2.93639200	-0.65234200
C	-1.86781700	-1.40617600	0.09074600
C	2.35346400	0.15338900	0.38478200
C	1.15446100	0.83020600	-0.06681800
C	-2.44883600	-0.21738400	0.56476700
C	2.34473800	-1.06549000	1.11615700
H	1.39645700	-1.51737500	1.38067700
C	-0.50828800	-1.10529900	-0.33358200
C	-0.14199100	0.19522600	-0.07230400
C	-3.73682500	-0.18458000	1.06961400
H	-4.17724600	0.74626900	1.41668100
C	1.23441500	2.21519600	-0.45198400
C	3.51315300	-1.67244300	1.54114100
H	3.45553300	-2.60150000	2.10352800
C	3.63308000	0.77382500	0.18938700
C	-2.59456600	-2.59635200	0.12342200
H	-2.15461000	-3.51893000	-0.24251200
C	4.76928200	-1.09623400	1.26503400
H	5.68367300	-1.58622300	1.58846800
C	-3.89210900	-2.57431700	0.65075800
H	-4.46394500	-3.49730800	0.69435500
C	3.68868500	2.08953000	-0.37228200
H	4.66577700	2.54065600	-0.53777800
C	2.55296300	2.78930900	-0.64348600
H	2.58590100	3.80876300	-1.01859300
C	4.81445500	0.12054000	0.60961700
H	5.76800100	0.61249600	0.42726500
C	-4.45872700	-1.38818000	1.12709700
H	-5.46190800	-1.39326700	1.54308400
C	-1.45798800	0.93290400	0.28362300
C	-1.47115000	1.99716000	1.31733300
S	-2.22365800	1.81101400	-1.20584400
C	-2.27509100	3.07431400	1.06086100
H	-0.95638700	1.82458200	2.25704600
C	-2.76934500	3.07374900	-0.26368100
H	-2.47674000	3.87678400	1.76007700
H	-3.39785500	3.84741000	-0.69496100

(a*S*,*S*,*R*)-4a

Br	-0.30002300	-2.76682000	0.72946700
O	-0.03762200	2.90555800	-0.45735700
C	1.99454200	-1.30510700	-0.24854000
C	-2.48610800	0.13283800	-0.28784000
C	-1.17375900	0.75019000	-0.18996800
C	2.46952100	-0.00317100	-0.50088800
C	-2.69079900	-1.19078700	-0.74800800
H	-1.84865000	-1.76036800	-1.11089000
C	0.56684600	-1.21181100	0.06884200
C	0.10352600	0.07273600	-0.09114800
C	3.78144800	0.21597500	-0.89273200
H	4.14987800	1.22066300	-1.07161000
C	-1.14590800	2.15687400	-0.23815500
C	-3.95379500	-1.75013300	-0.81158800
H	-4.06914000	-2.76588500	-1.18063000
C	-3.65746400	0.92051100	-0.03177700
C	2.84220100	-2.40478600	-0.36302600
H	2.48260800	-3.40745100	-0.15407900
C	-5.09006800	-1.00830900	-0.42784900

H	-6.07695400	-1.46131100	-0.46282800
C	4.16371500	-2.18570300	-0.77272700
H	4.83487900	-3.03289100	-0.88467000
C	-3.52537400	2.32668900	0.15225400
H	-4.41314500	2.91929300	0.35767400
C	-2.31210800	2.93486600	-0.02200300
H	-2.19794000	4.01350800	0.01579000
C	-4.93884900	0.31428500	-0.06550400
H	-5.80763600	0.92728600	0.16280800
C	4.62715600	-0.89473800	-1.04327400
H	5.65260700	-0.74653700	-1.37009300
C	1.35749300	0.94767400	-0.13067800
C	1.21105100	2.27284200	-0.85508700
S	1.80079400	1.53773300	1.65520500
C	2.28612400	3.19413600	-0.33729600
H	1.19817700	2.12993300	-1.94144600
C	2.59450700	2.94801200	0.94351300
H	2.67110400	4.02487200	-0.91755100
H	3.26989500	3.53089000	1.56262300

IV. Optimization of the reaction condition.

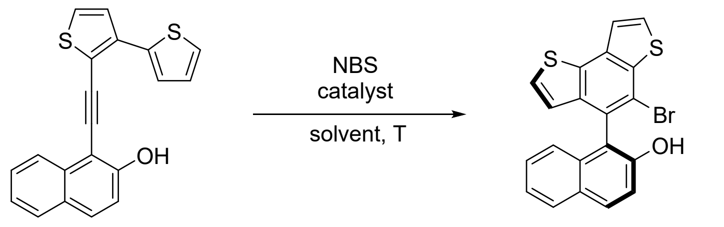

1a **2a**

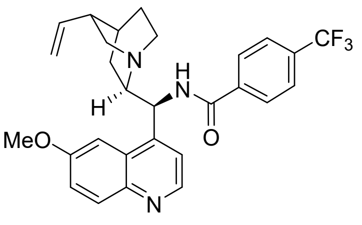

A

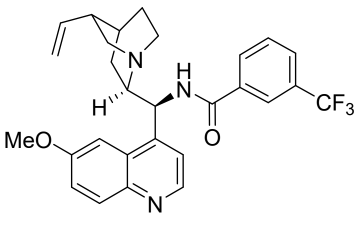

B

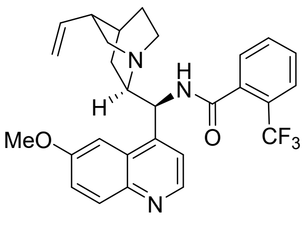

C

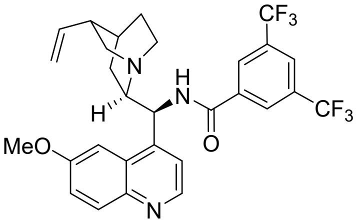

D

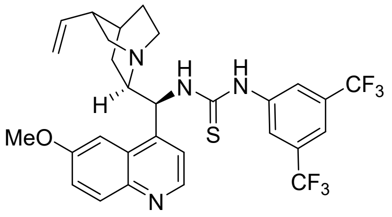

E

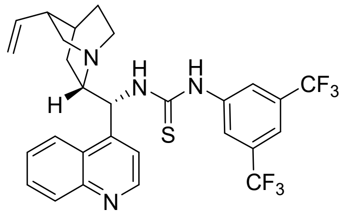

F

Supplementary Table 2. reaction screening

entry	catalyst (mol%)	solvent	T (°C)	yield[a] (%)	ee[b] (%)
1	**A** (20)	DCM	-40	93	72
2	**B** (20)	DCM	-40	91	48
3	**C** (20)	DCM	-40	87	21
4	**D** (20)	DCM	-40	82	63
5	**E** (20)	DCM	-40	98	9
6	**F** (20)	DCM	-40	97	-4
7	**A** (20)	toluene	-40	97	93
8	**A** (20)	acetone	-40	95	28
9	**A** (20)	EA	-40	96	78
10	**A** (20)	THF	-40	95	26
11	**A** (20)	$CHCl_3$	-40	94	95
12	**A** (20)	$CHCl_3$	0	85	65
13	**A** (20)	$CHCl_3$	-20	93	84
14	**A** (20)	$CHCl_3$	-60	99	97
15[c]	**A** (20)	$CHCl_3$	-60	97	97
16[d]	**A** (20)	$CHCl_3$	-60	99	97
17	**A** (10)	$CHCl_3$	-60	94	97

Reaction conditions: **1a** (0.05 mmol, 1.0 equiv.), catalyst (0.01 mmol, 20 mol%) in solvent (1 mL) at corresponding temperature for 30 min. then NBS (0.05 mmol, 1.0 equiv.) at corresponding temperature for 3 h. [a]Isolated yield. [b]Enantiomeric excess (ee) determined by HPLC. [c]Reaction in $CHCl_3$ (0.5 mL). [d]Reaction in $CHCl_3$ (1.5 mL).

V. General procedure for asymmetric reaction

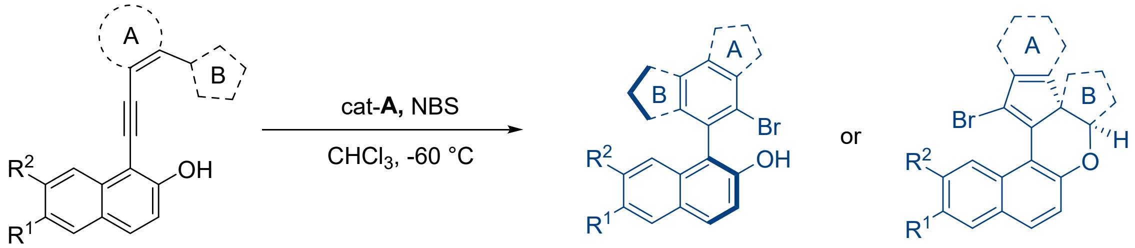

1 or 3 **2** **4**

Reaction condition [A]: A solution of **1** or **3** (0.05 mmol, 1.0 equiv.) and catalyst-**A** (0.005 mmol, 10 mol%) in $CHCl_3$ (1 mL) was stirred at -60 °C for 30 min, then NBS (0.05 mmol, 1.0 equiv.) was added. After stirring at -60 °C for 3 h, the reaction mixture was concentrated under reduced pressure. The resulting residue was purified by silica gel flash column chromatography using PE/EA eluent to afford the product **2** or **4**.

VI. Experiments about other substituted VQMs

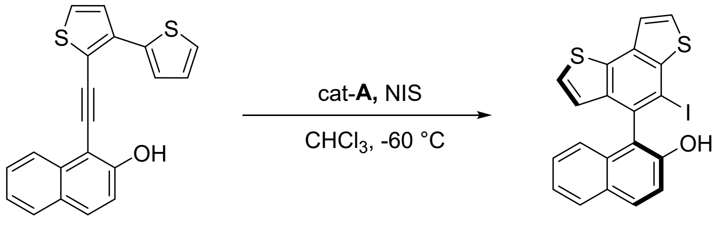

1a **2v**

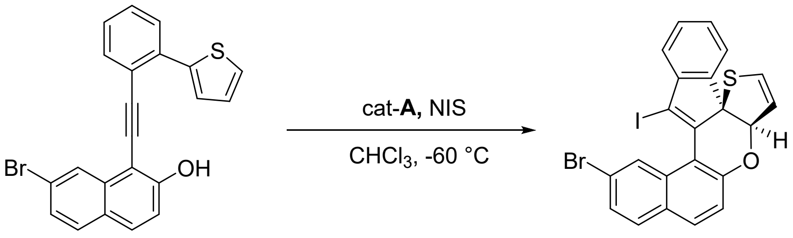

3a **4t**

Reaction condition: A solution of **1** (0.05 mmol, 1.0 equiv.) and catalyst-**A** (0.005 mmol, 10 mol%) in $CHCl_3$ (1 mL) was stirred at -60 °C for 30 min, then NIS (0.05 mmol, 1.0 equiv.) was added. After stirring at -60 °C for 3 h, the reaction mixture was concentrated under reduced pressure. The resulting residue was purified by silica gel flash column chromatography using PE/EA eluent to afford the product **2x** or **3s**.

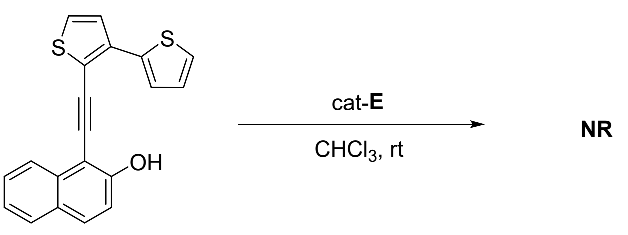

1a

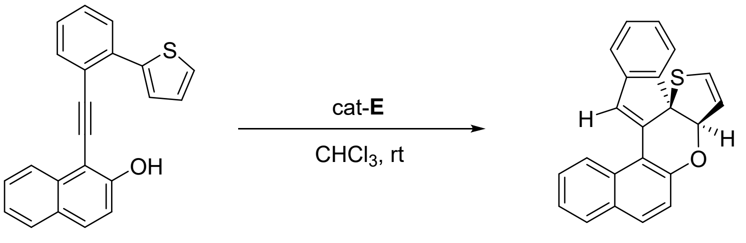

3a **4q**

Reaction condition: A solution of **3** (0.05 mmol, 1.0 equiv.) and catalyst-**E** (0.005 mmol, 10 mol%) in $CHCl_3$ (1 mL) was stirred at rt for 24h. Then the reaction mixture was concentrated under reduced pressure. The resulting residue was purified by silica gel flash column chromatography using PE/EA eluent to afford the product **4q, 4r, 4s**.

VII. ^{1}H, ^{13}C NMR and HRMS data of compounds (1a-1w)

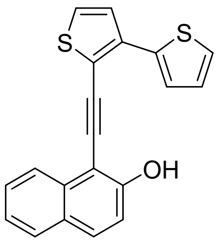

1-([2,3'-bithiophen]-2'-ylethynyl)naphthalen-2-ol (1a)

Compound **1a** was synthesized following the general procedure (**Method A**).

Yellow solid. (R_f = 0.4, PE/EA = 6:1)

^{1}H NMR (400 MHz, $CDCl_3$) δ 8.18 (d, J = 8.3 Hz, 1H), 7.75 (d, J = 8.9 Hz, 2H), 7.53 (dd, J = 9.4, 5.7 Hz, 2H), 7.36 (t, J = 7.5 Hz, 1H), 7.31 – 7.23 (m, 3H), 7.20 (d, J = 8.8 Hz, 1H), 7.06 (t, J = 4.4 Hz, 1H), 6.39 (s, 1H).
^{13}C NMR (100 MHz, $CDCl_3$) δ 156.26, 138.03, 137.52, 133.42, 131.21, 128.39, 128.28, 127.52, 127.41, 127.39, 127.05, 125.54, 125.21, 124.85, 124.16, 116.51, 116.08, 102.62, 94.22, 90.24.
HRMS (ESI) *m/z* Calcd for $C_{20}H_{12}NaOS_2^+$ [M + Na]$^+$: 355.0222, Found: 355.0221.

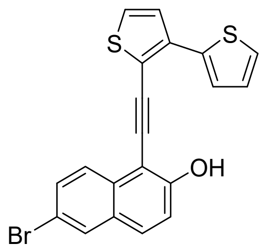

1-([2,3'-bithiophen]-2'-ylethynyl)-6-bromonaphthalen-2-ol (1b)

Compound **1b** was synthesized following the general procedure (**Method A**).

White solid. (R_f = 0.5, PE/EA= 5:1)

^{1}H NMR (400 MHz, $CDCl_3$) δ 8.01 (d, J = 8.9 Hz, 1H), 7.89 (s, 1H), 7.63 (d, J = 8.9 Hz, 1H), 7.59 – 7.46 (m, 2H), 7.36 – 7.22 (m, 3H), 7.19 (d, J = 9.0 Hz, 1H), 7.07 (s, 1H), 6.38 (s, 1H).
^{13}C NMR (100 MHz, $CDCl_3$) δ 156.36, 138.31, 137.40, 131.92, 130.63, 130.16, 130.05, 129.49, 127.62, 127.44, 127.11, 126.66, 125.63, 125.32, 117.87, 117.64, 115.77, 102.93, 94.60, 89.53.
HRMS (ESI) *m/z* Calcd for $C_{20}H_{11}BrNaOS_2^+$ [M + Na]$^+$: 432.9327, Found: 432.9327.

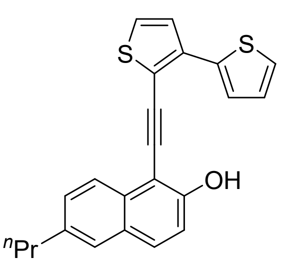

1-([2,3'-bithiophen]-2'-ylethynyl)-6-propylnaphthalen-2-ol (1c)

Compound **1c** was synthesized following the general procedure (**Method A**).

Black solid. (R_f = 0.5, PE/EA= 5:1)

^{1}H NMR (400 MHz, $CDCl_3$) δ 8.10 (d, J = 8.5 Hz, 1H), 7.71 (d, J = 8.9 Hz, 1H), 7.56 (d, J = 2.8 Hz, 1H), 7.54 (s, 1H), 7.38 (dd, J = 8.5, 1.8 Hz, 1H), 7.34 – 7.24 (m, 3H), 7.17 (d, J = 8.9 Hz, 1H), 7.07 (dd, J = 5.1, 3.6 Hz, 1H), 6.30 (s, 1H), 2.72 (t, J = 7.6 Hz, 2H), 1.71 (h, J = 7.4 Hz, 2H), 0.97 (t, J = 7.3 Hz, 3H).
^{13}C NMR (100 MHz, $CDCl_3$) δ 155.78, 138.52, 138.00, 137.61, 131.84, 130.78, 129.11, 128.60, 127.43, 127.40, 127.31, 127.09, 126.93, 125.54, 125.21, 124.77, 116.42, 116.29, 102.48, 94.01, 90.51, 37.83, 24.46, 13.82.
HRMS (ESI) *m/z* Calcd for $C_{23}H_{18}NaOS_2^+$ [M + Na]$^+$: 397.0691, Found: 397.0693.

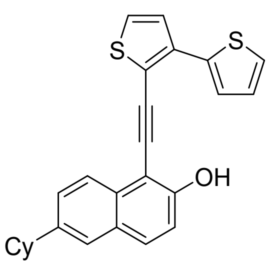

1-([2,3'-bithiophen]-2'-ylethynyl)-6-cyclohexylnaphthalen-2-ol (1d)

Compound **1d** was synthesized following the general procedure (**Method A**).

Black solid. (R_f = 0.4, PE/EA= 5:1)

^{1}H NMR (400 MHz, $CDCl_3$) δ 8.11 (d, J = 8.6 Hz, 1H), 7.71 (d, J = 8.9 Hz, 1H), 7.56 (s, 2H), 7.42 (d, J = 8.6 Hz, 1H), 7.28 (q, J = 5.4 Hz, 3H), 7.17 (d, J = 8.9 Hz, 1H), 7.07 (t, J = 4.4 Hz, 1H), 6.29 (s, 1H), 2.63 (t, J = 11.2 Hz, 1H), 1.94 (d, J = 12.1 Hz, 2H), 1.87 (d, J = 11.5 Hz, 2H), 1.77 (d, J = 12.6 Hz, 1H), 1.55 – 1.38 (m, 4H), 1.33 – 1.24 (m, 1H).
^{13}C NMR (100 MHz, $CDCl_3$) δ 155.80, 143.93, 137.97, 137.61, 131.97, 130.95, 128.64, 127.80, 127.41, 127.28, 127.07, 125.52, 125.19, 125.07, 124.78, 116.38, 116.29, 102.43, 93.95, 90.55, 44.29, 34.41, 26.90, 26.17.
HRMS (ESI) *m/z* Calcd for $C_{26}H_{22}NaOS_2^+$ $[M + Na]^+$: 437.1004, Found: 437.1008.

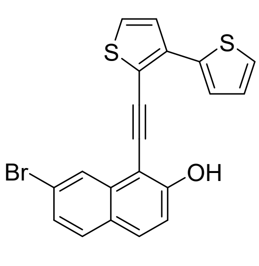

1-([2,3'-bithiophen]-2'-ylethynyl)-7-bromonaphthalen-2-ol (1e)

Compound **1e** was synthesized following the general procedure (**Method A**).

Yellow solid. (R_f = 0.5, PE/EA= 5:1)

^{1}H NMR (400 MHz, $CDCl_3$) δ 8.34 (s, 1H), 7.70 (d, J = 8.9 Hz, 1H), 7.60 (d, J = 8.6 Hz, 1H), 7.54 (d, J = 3.7 Hz, 1H), 7.44 (d, J = 8.5 Hz, 1H), 7.33 (t, J = 4.5 Hz, 2H), 7.28 (d, J = 5.3 Hz, 1H), 7.19 (d, J = 8.9 Hz, 1H), 7.11 (t, J = 4.4 Hz, 1H), 6.39 (s, 1H).
^{13}C NMR (100 MHz, $CDCl_3$) δ 156.80, 138.36, 137.38, 134.58, 130.96, 129.81, 127.64, 127.59, 127.53, 127.18, 127.16, 126.84, 125.65, 125.37, 122.19, 116.95, 115.74, 102.09, 94.71, 89.32.
HRMS (ESI) *m/z* Calcd for $C_{20}H_{11}BrNaOS_2^+$ $[M + Na]^+$: 432.9327, Found: 432.9328.

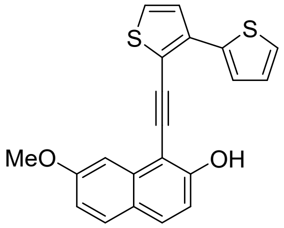

1-([2,3'-bithiophen]-2'-ylethynyl)-7-methoxynaphthalen-2-ol (1f)

Compound **1f** was synthesized following the general procedure (**Method A**).

Yellow solid. (R_f = 0.5, PE/EA= 5:1)

^{1}H NMR (400 MHz, $CDCl_3$) δ 7.67 (t, J = 10.4 Hz, 2H), 7.53 (d, J = 22.3 Hz, 2H), 7.38 – 7.21 (m, 3H), 7.15 – 6.94 (m, 3H), 6.31 (s, 1H), 3.88 (s, 3H).
^{13}C NMR (100 MHz, $CDCl_3$) δ 159.28, 156.78, 137.89, 137.60, 135.12, 131.00, 129.86, 127.42, 127.30, 127.08, 125.46, 125.16, 123.66, 116.56, 116.14, 113.84, 103.64, 101.88, 94.30, 90.52, 55.27.
HRMS (ESI) *m/z* Calcd for $C_{21}H_{14}NaO_2S_2^+$ $[M + Na]^+$: 385.0327, Found: 385.0325.

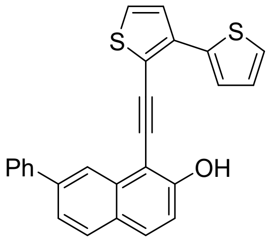

1-([2,3'-bithiophen]-2'-ylethynyl)-7-phenylnaphthalen-2-ol (1g)

Compound **1g** was synthesized following the general procedure (**Method A**).

Yellow solid. (R_f = 0.5, PE/EA= 5:1)

^{1}H NMR (400 MHz, $CDCl_3$) δ 8.41 (s, 1H), 7.80 (dd, *J* = 20.5, 8.7 Hz, 2H), 7.70 (d, *J* = 7.6 Hz, 2H), 7.63 (d, *J* = 8.4 Hz, 1H), 7.56 (s, 1H), 7.45 (t, *J* = 7.6 Hz, 2H), 7.36 (t, *J* = 7.4 Hz, 1H), 7.30 – 7.19 (m, 4H), 7.02 (s, 1H), 6.36 (s, 1H).
^{13}C NMR (100 MHz, $CDCl_3$) δ 156.60, 140.87, 140.19, 138.06, 137.52, 133.72, 130.89, 128.82, 127.60, 127.48, 127.44, 127.40, 127.13, 125.55, 125.24, 123.85, 122.92, 116.50, 116.06, 102.96, 94.64, 90.22.
HRMS (ESI) *m/z* Calcd for $C_{26}H_{16}NaOS_2^+$ [M + Na]$^+$: 431.0535, Found: 431.0535.

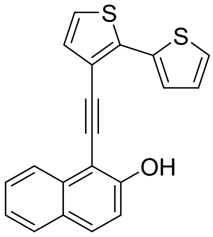

1-([2,2'-bithiophen]-3-ylethynyl)naphthalen-2-ol (1h)

Compound **1h** was synthesized following the general procedure (**Method A**).

Yellow solid. (R_f = 0.4, PE/EA= 5:1)

^{1}H NMR (400 MHz, $CDCl_3$) δ 8.20 (d, *J* = 8.4 Hz, 1H), 7.78 – 7.70 (m, 2H), 7.51 (t, *J* = 7.7 Hz, 1H), 7.45 (s, 1H), 7.35 (t, *J* = 7.6 Hz, 1H), 7.28 (d, *J* = 5.2 Hz, 1H), 7.22 – 7.16 (m, 2H), 7.14 (d, *J* = 4.9 Hz, 1H), 7.03 (s, 1H), 6.44 (s, 1H).
^{13}C NMR (100 MHz, $CDCl_3$) δ 156.20, 139.54, 135.40, 133.49, 131.22, 130.89, 128.39, 128.25, 127.44, 127.39, 125.97, 125.79, 124.87, 124.07, 123.36, 117.12, 116.46, 102.78, 96.36, 86.42.
HRMS (ESI) *m/z* Calcd for $C_{20}H_{12}NaOS_2^+$ [M + Na]$^+$: 355.0222, Found: 355.0222.

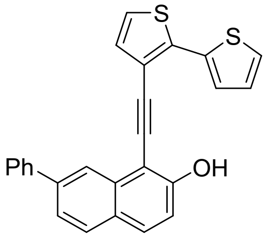

1-([2,2'-bithiophen]-3-ylethynyl)-7-phenylnaphthalen-2-ol (1i)

Compound **1i** was synthesized following the general procedure (**Method A**).

Yellow solid. (R_f = 0.5, PE/EA= 5:1)

^{1}H NMR (400 MHz, $CDCl_3$) δ 8.43 (s, 1H), 7.83 (d, *J* = 8.4 Hz, 1H), 7.77 (d, *J* = 8.9 Hz, 1H), 7.70 (d, *J* = 7.7 Hz, 2H), 7.63 (d, *J* = 8.4 Hz, 1H), 7.55 – 7.40 (m, 3H), 7.37 (t, *J* = 7.4 Hz, 1H), 7.25 (d, *J* = 5.1 Hz, 1H), 7.24 – 7.18 (m, 3H), 7.01 (t, *J* = 4.4 Hz, 1H), 6.42 (s, 1H).
^{13}C NMR (100 MHz, $CDCl_3$) δ 156.57, 141.00, 140.14, 139.64, 135.40, 133.80, 131.29, 130.60, 128.82, 128.79, 127.61, 127.50, 127.47, 127.45, 126.01, 125.86, 123.80, 123.42, 123.01, 117.08, 116.45, 103.12, 96.77, 86.36.

HRMS (ESI) *m/z* Calcd for $C_{26}H_{16}NaOS_2^+$ [M + Na]$^+$: 431.0535, Found: 431.0533.

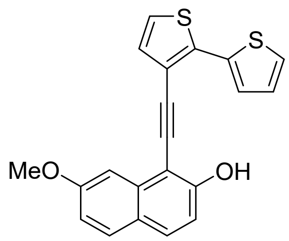

1-([2,2'-bithiophen]-3-ylethynyl)-7-methoxynaphthalen-2-ol (1j)

Compound **1j** was synthesized following the general procedure (**Method A**).

Yellow solid. (R_f = 0.5, PE/EA= 5:1)

^{1}H NMR (400 MHz, $CDCl_3$) δ 7.67 (t, *J* = 8.5 Hz, 2H), 7.53 (s, 1H), 7.49 (d, *J* = 3.4 Hz, 1H), 7.30 (d, *J* = 5.1 Hz, 1H), 7.19 (s, 2H), 7.07 – 7.00 (m, 3H), 6.35 (s, 1H), 3.87 (s, 3H).
^{13}C NMR (100 MHz, $CDCl_3$) δ 159.26, 156.80, 139.55, 135.55, 135.20, 131.27, 130.71, 129.85, 127.47, 125.93, 125.77, 123.73, 123.39, 117.19, 116.45, 113.83, 103.81, 102.07, 96.43, 86.65, 55.29.

HRMS (ESI) *m/z* Calcd for $C_{21}H_{14}NaO_2S_2^+$ [M + Na]$^+$: 385.0327, Found: 385.0328.

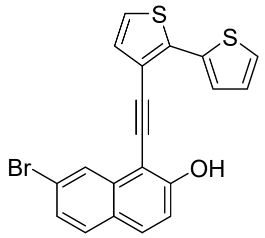

1-([2,2'-bithiophen]-3-ylethynyl)-7-bromonaphthalen-2-ol (1k)

Compound **1k** was synthesized following the general procedure (**Method A**).

Yellow solid. (R_f = 0.5, PE/EA= 5:1)

^{1}H NMR (400 MHz, $CDCl_3$) δ 8.37 (s, 1H), 7.70 (d, *J* = 8.9 Hz, 1H), 7.61 (d, *J* = 8.6 Hz, 1H), 7.47 (d, *J* = 3.6 Hz, 1H), 7.44 (d, *J* = 8.6 Hz, 1H), 7.34 (d, *J* = 5.1 Hz, 1H), 7.25 – 7.18 (m, 3H), 7.09 (t, *J* = 4.4 Hz, 1H), 6.43 (s, 1H).
^{13}C NMR (100 MHz, $CDCl_3$) δ 156.82, 139.92, 135.27, 134.73, 131.25, 130.68, 129.82, 127.59, 127.53, 127.25, 126.90, 126.17, 126.00, 123.54, 122.11, 116.94, 102.31, 96.88, 93.48, 85.55.

HRMS (ESI) *m/z* Calcd for $C_{20}H_{11}BrNaOS_2^+$ [M + Na]$^+$: 432.9327, Found: 432.9329.

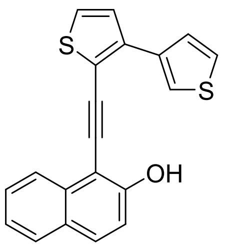

1-([3,3'-bithiophen]-2-ylethynyl)naphthalen-2-ol (1l)

Compound **1l** was synthesized following the general procedure (**Method A**).

Yellow solid. (R_f = 0.5, PE/EA= 5:1)

^{1}H NMR (400 MHz, $CDCl_3$) δ 8.15 (d, *J* = 8.3 Hz, 1H), 7.83 (s, 1H), 7.78 (dd, *J* = 8.4, 4.5 Hz, 2H), 7.65 (d, *J* = 4.5 Hz, 1H), 7.54 (t, *J* = 7.5 Hz, 1H), 7.40 (dt, *J* = 10.2, 5.2 Hz, 2H), 7.35 (d, *J* = 5.2 Hz, 1H), 7.26 (d, *J* = 5.3 Hz, 1H), 7.21 (d, *J* = 8.9 Hz, 1H), 6.15 (s, 1H).

^{13}C NMR (100 MHz, $CDCl_3$) δ 156.04, 139.98, 136.16, 133.39, 131.03, 128.42, 128.30, 127.74, 127.53, 127.23, 126.99, 126.11, 124.82, 124.18, 122.67, 116.76, 116.50, 102.72, 94.71.

HRMS (ESI) *m/z* Calcd for $C_{20}H_{12}NaOS_2^+$ $[M + Na]^+$: 355.0222, Found: 355.0222.

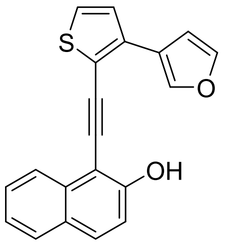

1-((3-(furan-3-yl)thiophen-2-yl)ethynyl)naphthalen-2-ol (1m)

Compound **1m** was synthesized following the general procedure (**Method A**).

Yellow solid. (R_f = 0.4, PE/EA= 5:1)

^{1}H NMR (400 MHz, $CDCl_3$) δ 8.17 (d, *J* = 8.4 Hz, 1H), 8.06 (s, 1H), 7.79 (d, *J* = 8.7 Hz, 2H), 7.54 (t, *J* = 7.6 Hz, 1H), 7.48 (s, 1H), 7.39 (t, *J* = 7.5 Hz, 1H), 7.35 (d, *J* = 5.3 Hz, 1H), 7.22 (d, *J* = 9.0 Hz, 1H), 7.17 (d, *J* = 5.3 Hz, 1H), 6.96 (s, 1H), 6.18 (s, 1H).

^{13}C NMR (100 MHz, $CDCl_3$) δ 156.11, 143.30, 140.18, 136.70, 133.46, 131.15, 128.42, 128.32, 127.60, 127.57, 126.98, 124.74, 124.19, 120.95, 116.49, 116.23, 109.33, 102.62, 94.23, 88.62.

HRMS (ESI) *m/z* Calcd for $C_{20}H_{12}NaO_2S^+$ $[M + Na]^+$: 339.0450, Found: 339.0455.

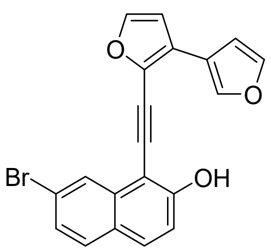

1-([3,3'-bifuran]-2-ylethynyl)-7-bromonaphthalen-2-ol (1n)

Compound **1n** was synthesized following the general procedure (**Method A**).

Yellow solid. (R_f = 0.5, PE/EA= 5:1)

^{1}H NMR (400 MHz, $CDCl_3$) δ 8.32 (s, 1H), 7.89 (s, 1H), 7.76 (d, *J* = 9.0 Hz, 1H), 7.65 (d, *J* = 8.6 Hz, 1H), 7.58 – 7.39 (m, 3H), 7.22 (d, *J* = 9.0 Hz, 1H), 6.90 (s, 1H), 6.63 (d, *J* = 1.6 Hz, 1H), 6.27 (s, 1H).
^{13}C NMR (100 MHz, $CDCl_3$) δ 157.05, 144.49, 143.77, 139.79, 134.67, 131.33, 129.91, 127.75, 127.01, 126.88, 123.10, 122.44, 117.28, 117.04, 110.51, 108.76, 101.46, 91.37, 88.73.
HRMS (ESI) *m/z* Calcd for $C_{20}H_{11}BrNaO^+$ $[M + Na]^+$: 400.9784, Found: 400.9785.

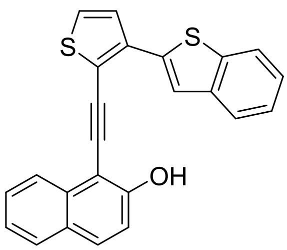

1-((3-(benzo[b]thiophen-2-yl)thiophen-2-yl)ethynyl)naphthalen-2-ol (1o)

Compound **1o** was synthesized following the general procedure (**Method A**).

Yellow solid. (R_f = 0.5, PE/EA= 5:1)

^{1}H NMR (400 MHz, $CDCl_3$) δ 8.21 (d, *J* = 8.3 Hz, 1H), 7.86 – 7.71 (m, 5H), 7.52 (t, *J* = 7.6 Hz, 1H), 7.40 – 7.30 (m, 5H), 7.22 (d, *J* = 9.2 Hz, 1H), 6.44 (s, 1H).
^{13}C NMR (100 MHz, $CDCl_3$) δ 156.49, 139.69, 139.25, 137.82, 137.35, 133.46, 131.34, 128.45, 128.33, 127.63, 127.48, 127.35, 124.87, 124.80, 124.71, 124.24, 123.70, 122.22, 122.05, 116.63, 102.60, 94.07, 90.95.
HRMS (ESI) *m/z* Calcd for $C_{24}H_{14}NaOS_2^+$ $[M + Na]^+$: 405.0378, Found: 405.0380.

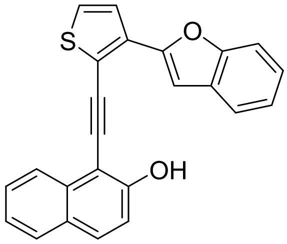

1-((3-(benzofuran-2-yl)thiophen-2-yl)ethynyl)naphthalen-2-ol (1p)

Compound **1p** was synthesized following the general procedure (**Method A**).

Yellow solid. (R_f = 0.5, PE/EA= 5:1)

^{1}H NMR (400 MHz, $CDCl_3$) δ 8.21 (d, *J* = 8.2 Hz, 1H), 7.78 (d, *J* = 8.4 Hz, 2H), 7.66 (d, *J* = 8.1 Hz, 1H), 7.55 (d, *J* = 6.8 Hz, 2H), 7.43 – 7.35 (m, 2H), 7.35 – 7.29 (m, 2H), 7.28 – 7.18 (m, 3H), 6.89 (s, 1H).
^{13}C NMR (100 MHz, $CDCl_3$) δ 156.87, 154.49, 151.96, 133.79, 133.19, 131.15, 128.47, 128.40, 128.33, 127.55, 127.32, 126.15, 124.98, 124.86, 124.18, 123.28, 121.08, 117.71, 116.55, 111.46, 103.94, 102.76, 94.44, 90.17.
HRMS (ESI) *m/z* Calcd for $C_{24}H_{14}NaO_2S^+$ $[M + Na]^+$: 389.0607, Found: 389.0607.

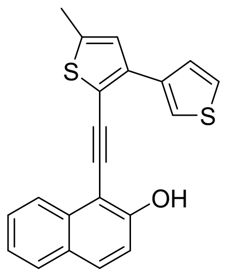

1-((5-methyl-[3,3'-bithiophen]-2-yl)ethynyl)naphthalen-2-ol (1q)

Compound **1q** was synthesized following the general procedure (**Method A**).

Yellow solid. (R_f = 0.5, PE/EA= 5:1)

^{1}H NMR (400 MHz, $CDCl_3$) δ 8.11 (d, *J* = 8.1 Hz, 1H), 7.82 – 7.62 (m, 3H), 7.58 (d, *J* = 4.1 Hz, 1H), 7.48 (t, *J* = 7.3 Hz, 1H), 7.38 – 7.25 (m, 2H), 7.16 (d, *J* = 8.6 Hz, 1H), 6.84 (s, 1H), 6.06 (s, 1H), 2.43 (s, 3H).
^{13}C NMR (100 MHz, $CDCl_3$) δ 155.85, 141.78, 139.94, 136.37, 133.36, 130.70, 128.38, 128.22, 127.37, 126.90, 126.00, 125.84, 124.80, 124.05, 122.40, 116.43, 114.17, 102.95, 95.07, 87.48, 15.43.
HRMS (ESI) *m/z* Calcd for $C_{21}H_{14}NaOS_2^+$ $[M + Na]^+$: 369.0378, Found: 369.0380.

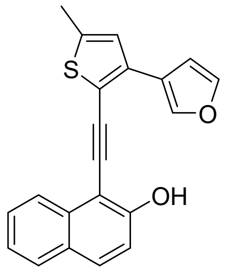

1-((3-(furan-3-yl)-5-methylthiophen-2-yl)ethynyl)naphthalen-2-ol (1r)

Compound **1r** was synthesized following the general procedure (**Method A**).

Yellow solid. (R_f = 0.5, PE/EA= 5:1)

^{1}H NMR (400 MHz, $CDCl_3$) δ 8.15 (d, J = 8.4 Hz, 1H), 8.00 (s, 1H), 7.76 (dd, J = 8.4, 5.3 Hz, 2H), 7.52 (t, J = 7.6 Hz, 1H), 7.44 (s, 1H), 7.37 (t, J = 7.5 Hz, 1H), 7.20 (d, J = 8.9 Hz, 1H), 6.92 (s, 1H), 6.82 (s, 1H), 6.17 (s, 1H), 2.49 (s, 3H).
^{13}C NMR (100 MHz, $CDCl_3$) δ 156.02, 143.19, 142.25, 140.14, 136.79, 133.52, 130.92, 128.48, 128.30, 127.51, 125.33, 124.81, 124.14, 121.20, 116.46, 113.78, 109.34, 102.90, 94.71, 87.84, 15.50.
HRMS (ESI) *m/z* Calcd for $C_{21}H_{14}NaO_2S^+$ $[M + Na]^+$: 353.0607, Found: 353.0603.

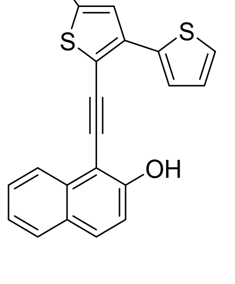

1-((5'-methyl-[2,3'-bithiophen]-2'-yl)ethynyl)naphthalen-2-ol (1s)

Compound **1s** was synthesized following the general procedure (**Method A**).

Orange solid. (R_f = 0.5, PE/EA= 5:1)

^{1}H NMR (400 MHz, $CDCl_3$) δ 8.18 (d, J = 8.3 Hz, 1H), 7.76 (dd, J = 8.9, 4.1 Hz, 2H), 7.59 – 7.45 (m, 2H), 7.37 (t, J = 7.4 Hz, 1H), 7.27 (d, J = 4.7 Hz, 1H), 7.20 (d, J = 8.9 Hz, 1H), 7.06 (d, J = 4.4 Hz, 1H), 6.96 (s, 1H), 6.35 (s, 1H), 2.49 (s, 3H).
^{13}C NMR (100 MHz, $CDCl_3$) δ 156.17, 142.04, 138.13, 137.88, 133.50, 130.99, 128.47, 128.27, 127.46, 127.36, 125.38, 125.36, 124.99, 124.94, 124.13, 116.51, 113.72, 102.92, 94.68, 89.49, 15.52.
HRMS (ESI) *m/z* Calcd for $C_{21}H_{14}NaOS_2^+$ [M + Na]$^+$: 369.0378, Found: 369.0380.

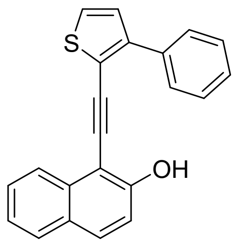

1-((3-phenylthiophen-2-yl)ethynyl)naphthalen-2-ol (1t)

Compound **1t** was synthesized following the general procedure (**Method A**).

Yellow solid. (R_f = 0.5, PE/EA= 5:1)

^{1}H NMR (400 MHz, $CDCl_3$) δ 8.07 (d, *J* = 8.4 Hz, 1H), 7.81 (d, *J* = 7.2 Hz, 2H), 7.73 (t, *J* = 8.9 Hz, 2H), 7.48 (dt, *J* = 11.0, 7.8 Hz, 3H), 7.41 – 7.33 (m, 3H), 7.22 (d, *J* = 5.2 Hz, 1H), 7.15 (d, *J* = 8.9 Hz, 1H), 6.00 (s, 1H).
^{13}C NMR (100 MHz, $CDCl_3$) δ 155.94, 145.37, 135.50, 133.28, 130.82, 128.78, 128.41, 128.25, 128.23, 128.03, 127.92, 127.40, 127.02, 124.88, 124.12, 117.74, 116.44, 102.79, 94.77, 87.39.
HRMS (ESI) *m/z* Calcd for $C_{22}H_{14}NaOS^+$ [M + Na]$^+$: 349.0658, Found: 349.0662 .

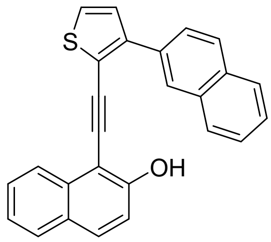

1-((3-(naphthalen-2-yl)thiophen-2-yl)ethynyl)naphthalen-2-ol (1u)

Compound **1u** was synthesized following the general procedure (**Method A**).

Yellow solid. (R_f = 0.5, PE/EA= 5:1)

^{1}H NMR (400 MHz, $CDCl_3$) δ 8.28 (s, 1H), 8.04 (d, *J* = 8.2 Hz, 1H), 7.91 (s, 2H), 7.86 – 7.78 (m, 2H), 7.70 (t, *J* = 8.7 Hz, 2H), 7.52 – 7.44 (m, 2H), 7.43 – 7.25 (m, 4H), 7.13 (d, *J* = 8.9 Hz, 1H), 6.03 (s, 1H).
^{13}C NMR (100 MHz, $CDCl_3$) δ 155.97, 145.07, 133.41, 133.29, 132.82, 130.81, 128.43, 128.39, 128.22, 127.72, 127.47, 127.09, 126.83, 126.47, 126.30, 125.86, 124.85, 124.10, 117.91, 116.44, 102.77, 94.88, 87.67.
HRMS (ESI) *m/z* Calcd for $C_{26}H_{16}NaOS^+$ [M + Na]$^+$: 399.0814 , Found: 399.0814 .

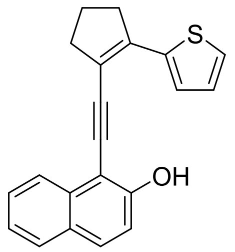

1-((2-(thiophen-2-yl)cyclopent-1-en-1-yl)ethynyl)naphthalen-2-ol (1v)

Compound **1v** was synthesized following the general procedure (**Method E**).

Black solid. (R_f = 0.5, PE/EA= 5:1)

^{1}H NMR (400 MHz, $CDCl_3$) δ 8.20 (d, J = 8.3 Hz, 1H), 7.74 (t, J = 8.0 Hz, 2H), 7.52 (t, J = 7.5 Hz, 1H), 7.35 (t, J = 7.5 Hz, 1H), 7.28 (d, J = 5.0 Hz, 1H), 7.23 – 7.17 (m, 2H), 7.02 (t, J = 4.2 Hz, 1H), 6.60 (s, 1H), 2.97 – 2.87 (m, 4H), 2.07 (p, J = 7.6 Hz, 2H).

^{13}C NMR (100 MHz, $CDCl_3$) δ 155.99, 140.63, 139.16, 133.46, 130.74, 128.42, 128.23, 127.32, 126.77, 126.35, 125.57, 124.91, 124.01, 116.52, 116.44, 103.28, 99.23, 91.06, 38.74, 36.14, 22.71.

HRMS (ESI) *m/z* Calcd for $C_{21}H_{16}NaOS^+$ $[M + Na]^+$: 339.0814, Found: 339.0816.

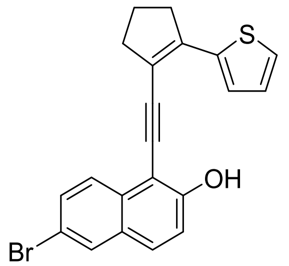

6-bromo-1-((2-(thiophen-2-yl)cyclopent-1-en-1-yl)ethynyl)naphthalen-2-ol (1w)

Compound **1w** was synthesized following the general procedure (**Method E**).

Black solid. (R_f = 0.5, PE/EA= 5:1)

^{1}H NMR (400 MHz, $CDCl_3$) δ 8.05 (d, J = 8.8 Hz, 1H), 7.91 (s, 1H), 7.64 (d, J = 8.9 Hz, 1H), 7.57 (d, J = 8.8 Hz, 1H), 7.31 (d, J = 5.0 Hz, 1H), 7.24 – 7.14 (m, 2H), 7.07 – 7.02 (m, 1H), 6.58 (s, 1H), 2.93 (dt, J = 26.6, 7.3 Hz, 4H), 2.14 – 2.06 (m, 2H).

^{13}C NMR (100 MHz, $CDCl_3$) δ 156.13, 141.20, 139.05, 132.00, 130.48, 130.14, 129.62, 129.55, 126.84, 126.79, 126.54, 125.73, 117.73, 117.59, 116.24, 103.62, 99.59, 90.33, 38.70, 36.20, 22.74.

HRMS (ESI) *m/z* Calcd for $C_{21}H_{15}BrNaOS^+$ $[M + Na]^+$: 416.9919, Found: 416.9925.

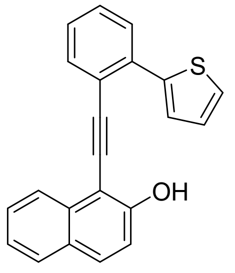

1-((2-(thiophen-2-yl)phenyl)ethynyl)naphthalen-2-ol (3a)

Compound **3a** was synthesized following the general procedure (**Method B**).

Yellow solid. (R_f = 0.5, PE/EA= 5:1)

^{1}H NMR (400 MHz, $CDCl_3$) δ 8.10 (d, *J* = 8.1 Hz, 1H), 7.80 – 7.70 (m, 3H), 7.59 – 7.55 (m, 1H), 7.54 – 7.48 (m, 2H), 7.43 (dd, *J* = 5.1, 1.3 Hz, 1H), 7.42 – 7.32 (m, 3H), 7.21 – 7.12 (m, 2H), 6.14 (s, 1H).
^{13}C NMR (100 MHz, $CDCl_3$) δ 156.28, 142.13, 135.74, 133.49, 133.15, 130.83, 129.74, 128.78, 128.37, 128.24, 127.64, 127.51, 127.35, 126.82, 126.17, 124.85, 124.03, 121.32, 116.46, 102.82, 100.74, 86.14.
HRMS (ESI) *m/z* Calcd for $C_{22}H_{14}NaOS^+$ $[M + Na]^+$: 349.0658, Found: 349.0657.

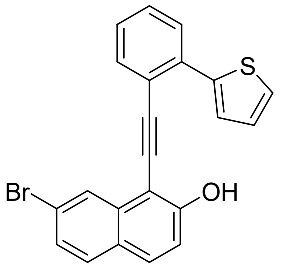

7-bromo-1-((2-(thiophen-2-yl)phenyl)ethynyl)naphthalen-2-ol (3b)

Compound **3b** was synthesized following the general procedure (**Method B**).

Yellow solid. (R_f = 0.5, PE/EA= 5:1)

^{1}H NMR (400 MHz, $CDCl_3$) δ 8.24 (s, 1H), 7.75 (d, *J* = 7.1 Hz, 1H), 7.67 (d, *J* = 8.9 Hz, 1H), 7.62 – 7.53 (m, 2H), 7.51 (d, *J* = 3.0 Hz, 1H), 7.48 – 7.29 (m, 4H), 7.19 (t, *J* = 4.4 Hz, 1H), 7.14 (d, *J* = 8.9 Hz, 1H), 6.17 (s, 1H).
^{13}C NMR (100 MHz, $CDCl_3$) δ 156.89, 141.97, 135.86, 134.66, 133.24, 130.64, 129.81, 129.79, 129.01, 127.71, 127.65, 127.48, 127.13, 126.84, 126.81, 126.33, 122.04, 120.99, 116.89, 102.26, 101.18, 85.27.
HRMS (ESI) *m/z* Calcd for $C_{22}H_{13}BrNaOS^+$ $[M + Na]^+$: 426.9763, Found: 426.9763.

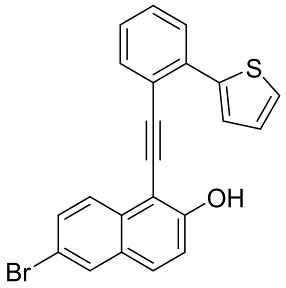

6-bromo-1-((2-(thiophen-2-yl)phenyl)ethynyl)naphthalen-2-ol (3c)

Compound **3c** was synthesized following the general procedure (**Method B**).

Yellow solid. (R_f = 0.6, PE/EA= 5:1)

^{1}H NMR (400 MHz, $CDCl_3$) δ 7.94 – 7.85 (m, 2H), 7.73 (d, *J* = 6.4 Hz, 1H), 7.61 (d, *J* = 9.0 Hz, 1H), 7.58 – 7.51 (m, 2H), 7.49 (d, *J* = 2.8 Hz, 1H), 7.44 – 7.34 (m, 3H), 7.20 – 7.11 (m, 2H), 6.13 (s, 1H).
^{13}C NMR (100 MHz, $CDCl_3$) ^{13}C NMR (101 MHz, cdcl$_3$) δ 156.39, 142.03, 135.87, 133.10, 132.00, 130.48, 130.13, 129.84, 129.71, 129.47, 128.98, 127.71, 127.52, 126.86, 126.68, 126.25, 121.08, 117.73, 117.59, 103.12, 101.09, 85.42.
HRMS (ESI) *m/z* Calcd for $C_{22}H_{18}NaO^+$ $[M + Na]^+$: 426.9763, Found: 426.9763.

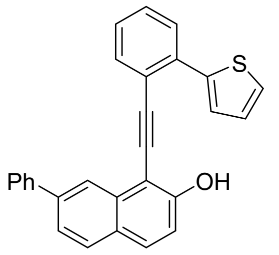

7-phenyl-1-((2-(thiophen-2-yl)phenyl)ethynyl)naphthalen-2-ol (3d)

Compound **3d** was synthesized following the general procedure (**Method B**).

Yellow solid. (R_f = 0.6, PE/EA= 5:1)

^{1}H NMR (400 MHz, $CDCl_3$) δ 8.32 (s, 1H), 7.81 (d, *J* = 8.4 Hz, 1H), 7.74 (t, *J* = 7.9 Hz, 2H), 7.69 (d, *J* = 7.7 Hz, 2H), 7.61 (d, *J* = 8.4 Hz, 1H), 7.55 (d, *J* = 7.3 Hz, 1H), 7.53 (d, *J* = 3.5 Hz, 1H), 7.48 (t, *J* = 7.5 Hz, 2H), 7.43 – 7.34 (m, 3H), 7.32 (d, *J* = 5.2 Hz, 1H), 7.16 (d, *J* = 8.9 Hz, 1H), 7.06 (t, *J* = 4.3 Hz, 1H), 6.17 (s, 1H).
^{13}C NMR (100 MHz, $CDCl_3$) δ 156.66, 142.05, 141.11, 140.13, 135.78, 133.76, 133.26, 130.55, 129.74, 128.88, 128.83, 128.75, 127.62, 127.53, 127.45, 126.81, 126.19, 123.79, 122.93, 121.14, 116.44, 103.11, 101.09, 86.09.
HRMS (ESI) *m/z* Calcd for $C_{28}H_{18}NaOS^+$ $[M + Na]^+$: 425.0971, Found: 425.0971.

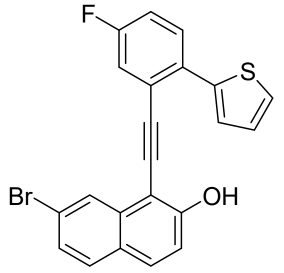

7-bromo-1-((5-fluoro-2-(thiophen-2-yl)phenyl)ethynyl)naphthalen-2-ol (3e)

Compound **3e** was synthesized following the general procedure (**Method B**).

Yellow solid. (R_f = 0.6, PE/EA= 5:1)

^{1}H NMR (400 MHz, $CDCl_3$) δ 8.17 (s, 1H), 7.66 (d, *J* = 9.0 Hz, 1H), 7.57 (d, *J* = 8.7 Hz, 1H), 7.49 (t, *J* = 7.1 Hz, 1H), 7.42 (d, *J* = 5.4 Hz, 4H), 7.19 – 7.07 (m, 3H), 6.06 (s, 1H).
^{13}C NMR (100 MHz, $CDCl_3$) δ 161.73 (d, *J* = 249.1 Hz), 157.20, 140.95, 134.62, 132.27, 131.67 (d, *J* = 8.6 Hz), 131.06, 129.82, 127.69, 127.61, 127.01, 126.97, 126.80, 126.39, 122.90 (d, *J* = 9.8 Hz), 122.22, 119.36 (d, *J* = 23.3 Hz), 116.94, 116.40 (d, *J* = 21.7 Hz), 101.74, 99.89, 86.19.
HRMS (ESI) *m/z* Calcd for $C_{22}H_{12}BrFNaOS^+$ $[M + Na]^+$: 444.9668, Found: 444.9665.

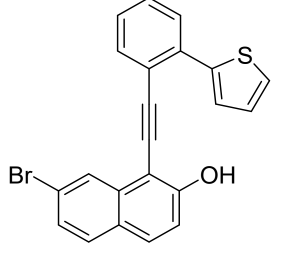

7-bromo-1-((4-methyl-2-(thiophen-2-yl)phenyl)ethynyl)naphthalen-2-ol (3f)

Compound **3f** was synthesized following the general procedure (**Method B**).

Yellow solid. (R_f = 0.4, PE/EA= 5:1)

^{1}H NMR (400 MHz, $CDCl_3$) δ 8.22 (s, 1H), 7.61 (dd, *J* = 8.4, 3.9 Hz, 2H), 7.55 (d, *J* = 8.6 Hz, 1H), 7.48 (s, 1H), 7.40 (t, *J* = 6.5 Hz, 2H), 7.34 (s, 1H), 7.21 – 7.07 (m, 3H), 6.16 (s, 1H), 2.39 (s, 3H).
^{13}C NMR (100 MHz, $CDCl_3$) δ 156.68, 142.10, 139.30, 135.65, 134.60, 133.13, 130.44, 130.34, 129.72, 128.55, 127.59, 127.38, 127.13, 126.77, 126.69, 126.13, 121.92, 117.99, 116.82, 102.46, 101.38, 84.53, 21.46.
HRMS (ESI) *m/z* Calcd for $C_{23}H_{15}BrNaOS^+$ [M + Na]$^+$: 440.9919, Found: 440.9919.

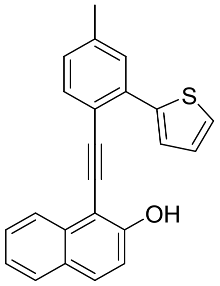

1-((4-methyl-2-(thiophen-2-yl)phenyl)ethynyl)naphthalen-2-ol (3g)

Compound **3g** was synthesized following the general procedure (**Method B**).

Yellow solid. (R_f = 0.5, PE/EA= 5:1)

^{1}H NMR (400 MHz, $CDCl_3$) δ 8.09 (d, *J* = 8.3 Hz, 1H), 7.70 (dd, *J* = 13.1, 8.5 Hz, 2H), 7.62 (dd, *J* = 7.9, 1.6 Hz, 1H), 7.53 – 7.44 (m, 2H), 7.42 – 7.36 (m, 1H), 7.36 – 7.26 (m, 2H), 7.13 (dd, *J* = 8.6, 2.4 Hz, 3H), 6.14 (s, 1H), 2.38 (s, 3H).
^{13}C NMR (100 MHz, $CDCl_3$) δ 156.09, 142.26, 139.06, 135.55, 133.45, 133.05, 130.57, 130.39, 128.52, 128.36, 128.19, 127.45, 127.25, 126.69, 125.98, 124.88, 123.96, 118.34, 116.41, 103.03, 100.92, 85.37, 21.43.
HRMS (ESI) *m/z* Calcd for $C_{23}H_{16}NaOS^+$ [M + Na]$^+$: 363.0814, Found: 363.0812.

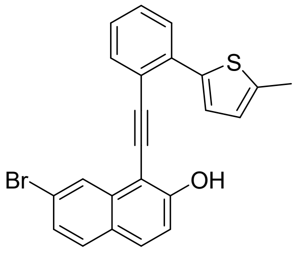

7-bromo-1-((2-(5-methylthiophen-2-yl)phenyl)ethynyl)naphthalen-2-ol (3h)

Compound **3h** was synthesized following the general procedure (**Method B**).

Yellow solid. (R_f = 0.5, PE/EA= 5:1)

^{1}H NMR (400 MHz, $CDCl_3$) δ 8.26 (s, 1H), 7.72 (d, *J* = 7.5 Hz, 1H), 7.66 (d, *J* = 8.9 Hz, 1H), 7.58 (d, *J* = 8.7 Hz, 1H), 7.51 (d, *J* = 7.4 Hz, 1H), 7.44 – 7.30 (m, 4H), 7.15 (d, *J* = 8.9 Hz, 1H), 6.82 (d, *J* = 3.6 Hz, 1H), 6.30 (s, 1H), 2.55 (s, 3H).
^{13}C NMR (100 MHz, $CDCl_3$) δ 156.96, 141.03, 139.54, 136.12, 134.70, 133.29, 130.55, 129.77, 129.43, 128.97, 127.44, 127.27, 127.18, 126.80, 126.76, 125.88, 121.98, 120.56, 116.92, 102.37, 101.40, 15.41.
HRMS (ESI) *m/z* Calcd for $C_{23}H_{15}BrNaOS^+$ [M + Na]$^+$: 440.9919, Found: 440.9919.

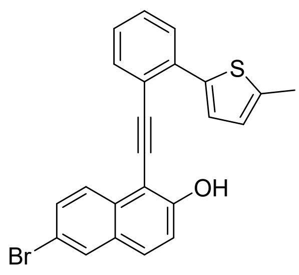

6-bromo-1-((2-(5-methylthiophen-2-yl)phenyl)ethynyl)naphthalen-2-ol (3i)

Compound **3i** was synthesized following the general procedure (**Method C**).

Yellow solid. (R_f = 0.5, PE/EA= 5:1)

^{1}H NMR (400 MHz, $CDCl_3$) δ 7.95 (d, *J* = 8.8 Hz, 1H), 7.88 (s, 1H), 7.70 (d, *J* = 7.4 Hz, 1H), 7.60 (d, *J* = 9.0 Hz, 1H), 7.52 (dd, *J* = 14.9, 8.3 Hz, 2H), 7.37 (t, *J* = 7.4 Hz, 1H), 7.32 (d, *J* = 7.5 Hz, 1H), 7.29 (d, *J* = 3.7 Hz, 1H), 7.16 (d, *J* = 8.9 Hz, 1H), 6.80 (d, *J* = 2.4 Hz, 1H), 6.27 (s, 1H), 2.54 (s, 3H).
^{13}C NMR (100 MHz, $CDCl_3$) δ 156.48, 141.01, 139.62, 136.11, 133.18, 132.05, 130.41, 130.12, 129.63, 129.46, 128.94, 127.29, 126.73, 125.71, 120.68, 117.69, 117.63, 103.23, 101.30, 85.41, 15.35.
HRMS (ESI) *m/z* Calcd for $C_{23}H_{15}BrNaOS^+$ $[M + Na]^+$: 440.9919, Found: 440.9919.

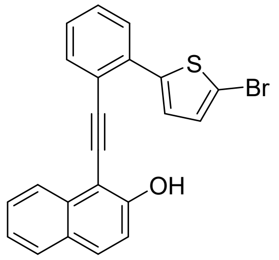

1-((2-(5-bromothiophen-2-yl)phenyl)ethynyl)naphthalen-2-ol (3j)

Compound **3j** was synthesized following the general procedure (**Method C**).

Yellow solid. (R_f = 0.5, PE/EA= 5:1)

^{1}H NMR (400 MHz, $CDCl_3$) δ 8.06 (d, *J* = 8.3 Hz, 1H), 7.76 (dt, *J* = 9.2, 4.6 Hz, 3H), 7.56 – 7.48 (m, 2H), 7.39 (q, *J* = 9.0, 7.6 Hz, 3H), 7.30 (d, *J* = 3.8 Hz, 1H), 7.19 (d, *J* = 8.9 Hz, 1H), 7.10 (d, *J* = 3.8 Hz, 1H), 6.15 (s, 1H).
^{13}C NMR (100 MHz, $CDCl_3$) δ 156.34, 143.60, 134.84, 133.53, 133.41, 131.04, 130.29, 129.39, 128.95, 128.42, 128.28, 128.02, 127.46, 127.11, 124.82, 124.13, 121.11, 116.51, 112.89, 102.67, 100.31, 86.64.
HRMS (ESI) *m/z* Calcd for $C_{22}H_{13}BrNaOS^+$ $[M + Na]^+$: 426.9763, Found: 426.9765.

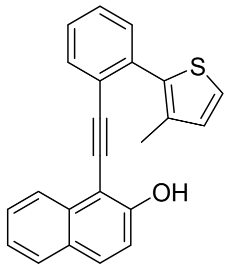

1-((2-(3-methylthiophen-2-yl)phenyl)ethynyl)naphthalen-2-ol (3k)

Compound **3k** was synthesized following the general procedure (**Method C**).

Yellow solid. (R_f = 0.5, PE/EA= 5:1)

^{1}H NMR (400 MHz, $CDCl_3$) δ 7.95 (d, *J* = 8.4 Hz, 1H), 7.78 – 7.67 (m, 3H), 7.49 (t, *J* = 7.7 Hz, 1H), 7.46 – 7.28 (m, 5H), 7.11 (d, *J* = 8.8 Hz, 1H), 7.03 (d, *J* = 4.7 Hz, 1H), 5.76 (s, 1H), 2.19 (s, 3H).
^{13}C NMR (100 MHz, $CDCl_3$) δ 156.14, 136.08, 135.87, 135.46, 133.31, 131.81, 131.29, 130.68, 130.20, 128.28, 128.16, 128.11, 127.30, 124.80, 124.40, 123.97, 116.41, 102.73, 100.42, 85.26, 14.51.
HRMS (ESI) *m/z* Calcd for $C_{23}H_{16}NaOS^+$ [M + Na]$^+$: 363.0814, Found: 363.0815.

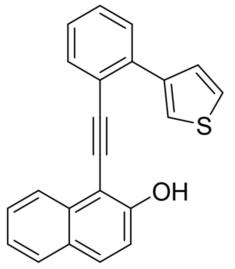

1-((2-(thiophen-3-yl)phenyl)ethynyl)naphthalen-2-ol (3l)

Compound **3l** was synthesized following the general procedure (**Method B**).

Yellow solid. (R_f = 0.5, PE/EA= 5:1)

^{1}H NMR (400 MHz, $CDCl_3$) δ 8.03 (d, *J* = 8.3 Hz, 1H), 7.71 (dd, *J* = 13.3, 8.3 Hz, 3H), 7.57 (s, 1H), 7.52 – 7.42 (m, 4H), 7.39 – 7.32 (m, 3H), 7.13 (d, *J* = 9.0 Hz, 1H), 5.91 (s, 1H).
^{13}C NMR (100 MHz, $CDCl_3$) δ 156.12, 141.36, 138.13, 133.38, 132.61, 130.67, 129.32, 128.72, 128.34, 128.30, 128.20, 127.30, 126.06, 124.83, 124.01, 123.50, 121.34, 116.40, 102.83, 101.06, 85.23.
HRMS (ESI) *m/z* Calcd for $C_{22}H_{14}NaOS^+$ [M + Na]$^+$: 349.0658, Found: 349.0658.

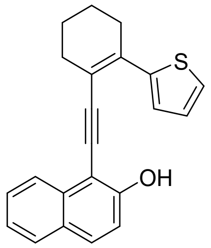

1-((2-(thiophen-2-yl)cyclohex-1-en-1-yl)ethynyl)naphthalen-2-ol (3m)

Compound **3m** was synthesized following the general procedure (**Method E**).

Yellow solid. (R_f = 0.5, PE/EA= 5:1)

^{1}H NMR (400 MHz, $CDCl_3$) δ 8.11 (d, *J* = 8.3 Hz, 1H), 7.73 (dd, *J* = 11.3, 8.8 Hz, 2H), 7.49 (t, *J* = 7.6 Hz, 1H), 7.38 (d, *J* = 3.4 Hz, 1H), 7.35 (t, *J* = 7.5 Hz, 1H), 7.27 (d, *J* = 5.0 Hz, 1H), 7.17 (d, *J* = 8.9 Hz, 1H), 7.04 (t, 1H), 6.43 (s, 1H), 2.67 – 2.57 (m, 4H), 1.86 – 1.75 (m, 4H).
^{13}C NMR (100 MHz, $CDCl_3$) δ 155.88, 144.11, 135.87, 133.56, 130.53, 128.41, 128.20, 127.24, 126.62, 125.43, 124.90, 124.87, 123.95, 116.43, 115.67, 103.38, 103.19, 88.20, 32.18, 30.53, 22.53, 22.22.
HRMS (ESI) *m/z* Calcd for $C_{22}H_{18}NaOS^+$ [M + Na]$^+$: 353.0971, Found: 353.0972.

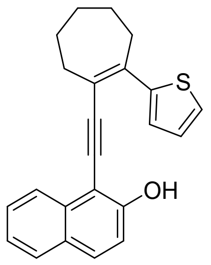

1-((2-(thiophen-2-yl)cyclohept-1-en-1-yl)ethynyl)naphthalen-2-ol (3n)

Compound **3n** was synthesized following the general procedure (**Method E**).

Yellow solid. (R_f = 0.5, PE/EA= 5:1)

^{1}H NMR (400 MHz, $CDCl_3$) δ 8.06 (d, *J* = 8.3 Hz, 1H), 7.73 (dd, *J* = 15.1, 8.5 Hz, 2H), 7.49 (t, *J* = 7.4 Hz, 1H), 7.40 (d, *J* = 3.4 Hz, 1H), 7.35 (t, *J* = 7.4 Hz, 1H), 7.30 (d, *J* = 5.0 Hz, 1H), 7.15 (d, *J* = 8.9 Hz, 1H), 6.36 (s, 1H), 2.83 – 2.75 (m, 4H), 1.91 – 1.84 (m, 2H), 1.79 – 1.68 (m, 4H).
^{13}C NMR (100 MHz, $CDCl_3$) δ 155.71, 145.19, 142.64, 133.43, 130.34, 128.31, 128.11, 127.10, 126.73, 125.92, 124.99, 124.78, 123.82, 120.98, 116.34, 104.56, 103.48, 88.04, 36.73, 35.49, 31.86, 26.13, 25.98.
HRMS (ESI) *m/z* Calcd for $C_{23}H_{20}NaOS^+$ $[M + Na]^+$: 367.1127, Found: 367.1125.

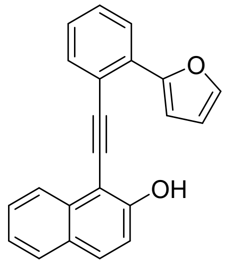

1-((2-(furan-2-yl)phenyl)ethynyl)naphthalen-2-ol (3o)

Compound **3o** was synthesized following the general procedure (**Method B**).

White solid. (R_f = 0.4, PE/EA= 8:1)

^{1}H NMR (400 MHz, $CDCl_3$) δ 8.21 (dd, *J* = 8.4, 3.8 Hz, 1H), 7.82 – 7.64 (m, 4H), 7.59 – 7.48 (m, 2H), 7.37 (q, *J* = 7.0 Hz, 2H), 7.28 (t, *J* = 7.6 Hz, 1H), 7.21 (dd, *J* = 8.8, 3.0 Hz, 1H), 7.12 (t, *J* = 2.9 Hz, 1H), 6.74 – 6.64 (m, 1H), 6.50 (s, 1H).
^{13}C NMR (100 MHz, $CDCl_3$) δ 156.57, 152.76, 142.49, 133.70, 133.49, 131.57, 130.83, 128.84, 128.42, 128.28, 127.39, 127.25, 126.55, 124.84, 124.04, 118.36, 116.43, 111.76, 109.05, 102.99, 100.99, 85.75.
HRMS (ESI) *m/z* Calcd for $C_{22}H_{14}NaO_2^+$ $[M + Na]^+$: 333.0886, Found: 333.0883.

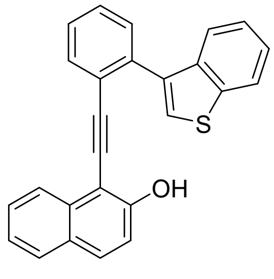

1-((2-(benzo[b]thiophen-3-yl)phenyl)ethynyl)naphthalen-2-ol (3p)

Compound **3p** was synthesized following the general procedure (**Method B**).

White solid. (R_f = 0.5, PE/EA= 6:1)

^{1}H NMR (400 MHz, $CDCl_3$) δ 7.99 (d, *J* = 7.8 Hz, 1H), 7.82 (dd, *J* = 5.6, 3.3 Hz, 1H), 7.71 (d, *J* = 7.5 Hz, 1H), 7.68 – 7.59 (m, 3H), 7.58 (s, 1H), 7.52 – 7.44 (m, 3H), 7.39 (dt, *J* = 15.7, 7.2 Hz, 3H), 7.28 (t, *J* = 7.5 Hz, 1H), 7.01 (d, *J* = 8.9 Hz, 1H), 5.29 (s, 1H).
^{13}C NMR (100 MHz, $CDCl_3$) δ 155.89, 140.17, 138.18, 137.46, 136.65, 133.34, 132.43, 130.58, 130.46, 128.64, 128.24, 128.08, 127.93, 127.22, 124.98, 124.77, 124.62, 124.50, 123.89, 123.27, 123.01, 116.33, 102.58, 100.64, 85.42.
HRMS (ESI) *m/z* Calcd for $C_{26}H_{16}NaOS^+$ [M + Na]$^+$: 399.0814, Found: 399.0814.

VIII. ^{1}H, ^{13}C NMR and HRMS data of compounds (2a-2x, 4a-4t)

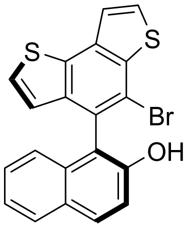

(*aS*)-1-(5-bromobenzo[1,2-b:3,4-b']dithiophen-4-yl)naphthalen-2-ol (2a)

Compound **2a** was synthesized in 94% yield (116.0 mg, 0.3 mmol scale) under condition [A].

White solid. (R_f = 0.3, PE/EA= 5:1)

^{1}H NMR (400 MHz, $CDCl_3$) δ 7.96 (d, *J* = 8.8 Hz, 1H), 7.90 (d, *J* = 8.0 Hz, 1H), 7.85 – 7.65 (m, 2H), 7.44 – 7.23 (m, 4H), 7.12 (d, *J* = 8.4 Hz, 1H), 6.78 (d, *J* = 5.2 Hz, 1H), 4.97 (s, 1H).
^{13}C NMR (100 MHz, $CDCl_3$) δ 150.72, 139.14, 138.76, 134.14, 134.07, 132.70, 130.56, 129.14, 128.89, 128.14, 126.90, 125.99, 125.72, 124.59, 124.35, 123.59, 122.73, 118.24, 117.53, 116.22.
HRMS (ESI) *m/z* Calcd for $C_{20}H_{11}BrNaOS_2^+$ [M + Na]$^+$: 432.9327, Found: 432.9327.

Optical Rotation: $[\alpha]_D^{25}$ = - 95.0° (*c* = 0.2, acetone).

HPLC analysis: Chiralcel IA-H (Hexane/*i*-PrOH = 90:10, flow rate = 1.0 mL/min, wave length = 254 nm), t_R = 12.202 min (minor), t_R = 19.388 min (major), 97% **ee**

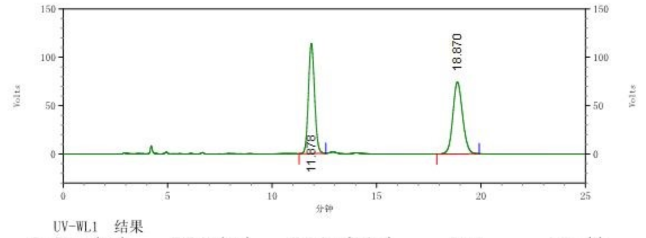

UV-WL1 结果

RetTime (min)	Width (min)	Height (Volts)	Area	Area (%)
11.878	1.28	454988	9301919	49.232
18.870	2.03	297521	9592103	50.768

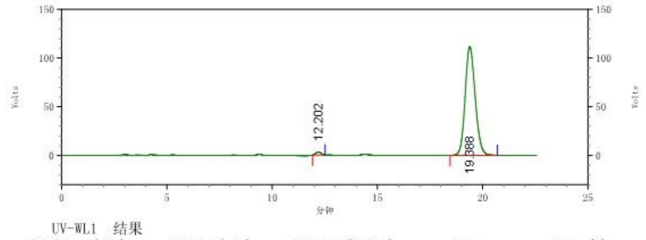

UV-WL1 结果

RetTime (min)	Width (min)	Height (Volts)	Area	Area (%)
12.202	0.59	12251	223119	1.483
19.388	2.25	446702	14824249	98.517

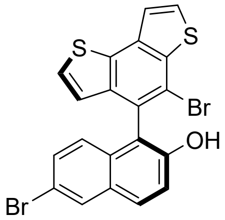

(*aS*)-6-bromo-1-(5-bromobenzo[1,2-b:3,4-b']dithiophen-4-yl)naphthalen-2-ol (2b)

Compound **2b** was synthesized in 93% yield (136.7 mg, 0.3 mmol scale) under condition [A].

White solid. (R_f = 0.3, PE/EA= 5:1)

^{1}H NMR (400 MHz, $CDCl_3$) δ 8.04 (d, *J* = 2.1 Hz, 1H), 7.85 (d, *J* = 8.9 Hz, 1H), 7.77 (q, *J* = 5.4 Hz, 2H), 7.41 – 7.29 (m, 3H), 6.96 (d, *J* = 9.0 Hz, 1H), 6.73 (d, *J* = 5.4 Hz, 1H), 4.94 (s, 1H).
^{13}C NMR (100 MHz, $CDCl_3$) δ 151.09, 139.15, 138.58, 134.27, 131.29, 130.15, 130.12, 130.05, 129.64, 129.42, 126.22, 126.03, 125.21, 124.31, 122.77, 118.74, 118.48, 117.43, 116.20.
HRMS (ESI) *m/z* Calcd for $C_{20}H_{10}Br_2NaOS_2^+$ [M + Na]$^+$: 510.8432, Found: 510.8434.

Optical Rotation: $[\alpha]_D^{25}$ = - 89.0° (*c* = 0.2, acetone).

HPLC analysis: Chiralcel IA-H (Hexane/*i*-PrOH = 90:10, flow rate = 1.0 mL/min, wave length = 254 nm), t_R = 12.632 min (minor), t_R = 18.188 min (major), 96% **ee**

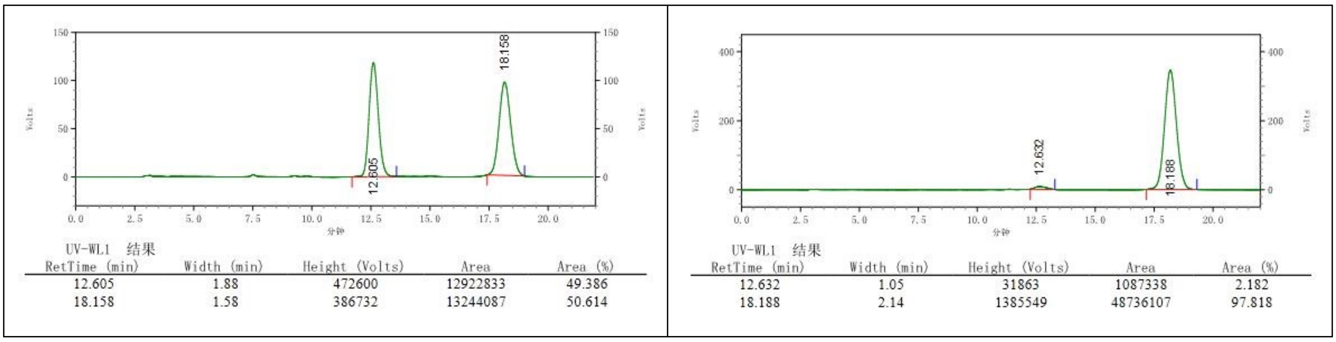

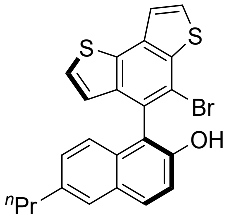

(*aS*)-1-(5-bromobenzo[1,2-b:3,4-b']dithiophen-4-yl)-6-propylnaphthalen-2-ol (2c)

Compound **2c** was synthesized in 90% yield (122.4 mg, 0.3 mmol scale) under condition [A].

White solid. (R_f = 0.3, PE/EA= 5:1)

^{1}H NMR (400 MHz, $CDCl_3$) δ 7.86 (d, *J* = 8.9 Hz, 1H), 7.82 – 7.66 (m, 2H), 7.64 (s, 1H), 7.29 (d, *J* = 8.7 Hz, 2H), 7.11 (d, *J* = 8.5 Hz, 1H), 6.98 (d, *J* = 8.6 Hz, 1H), 6.75 (d, *J* = 5.2 Hz, 1H), 4.84 (s, 1H), 2.69 (t, *J* = 7.6 Hz, 2H), 1.69 (h, *J* = 7.1 Hz, 2H), 0.96 (t, *J* = 7.3 Hz, 3H).
^{13}C NMR (100 MHz, $CDCl_3$) δ 150.12, 139.17, 138.81, 137.85, 134.11, 134.03, 131.10, 130.04, 129.14, 129.06, 128.47, 126.71, 126.27, 125.63, 124.70, 124.23, 122.74, 118.16, 117.42, 116.18, 37.85, 24.38, 13.96.
HRMS (ESI) *m/z* Calcd for $C_{23}H_{17}BrNaOS_2^+$ [M + Na]$^+$: 474.9796, Found: 474.9794.

Optical Rotation: $[\alpha]_D^{25}$ = - 26.0° (c = 0.2, acetone).

HPLC analysis: Chiralcel IA-H (Hexane/i-PrOH = 90:10, flow rate = 1.0 mL/min, wave length = 254 nm), t_R = 15.003 min (minor), t_R = 16.785 min (major), 96% **ee**

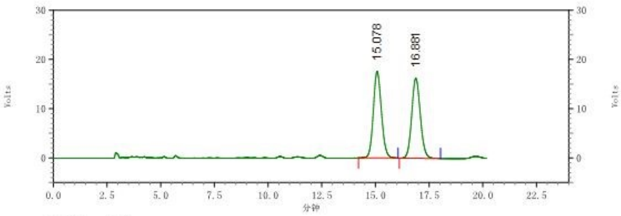

UV-WL1 结果

RetTime (min)	Width (min)	Height (Volts)	Area	Area (%)
15.078	1.84	70400	1852123	50.551
16.881	1.92	64911	1811726	49.449

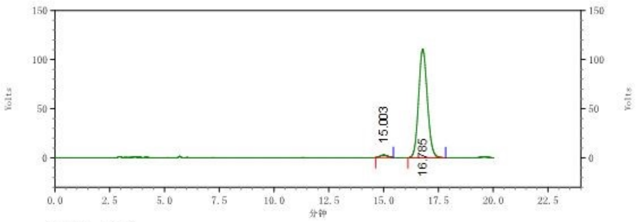

UV-WL1 结果

RetTime (min)	Width (min)	Height (Volts)	Area	Area (%)
15.003	0.81	10420	248346	1.974
16.785	1.72	441818	12333379	98.026

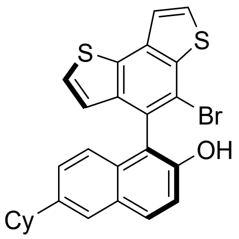

(*aS*)-1-(5-bromobenzo[1,2-b:3,4-b']dithiophen-4-yl)-6-cyclohexylnaphthalen-2-ol (2d)

Compound **2d** was synthesized in 89% yield (131.8 mg, 0.3 mmol scale) under condition [A].

White solid. (R_f = 0.3, PE/EA= 5:1)

^{1}H NMR (400 MHz, $CDCl_3$) δ 7.88 (d, J = 8.9 Hz, 1H), 7.84 – 7.68 (m, 2H), 7.66 (s, 1H), 7.39 – 7.26 (m, 2H), 7.16 (d, J = 8.6 Hz, 1H), 6.99 (d, J = 8.7 Hz, 1H), 6.77 (d, J = 5.4 Hz, 1H), 4.82 (s, 1H), 2.60 (t, J = 11.1 Hz, 1H), 1.94 (d, J = 11.9 Hz, 2H), 1.85 (d, J = 11.9 Hz, 2H), 1.76 (d, J = 12.9 Hz, 1H), 1.53 – 1.35 (m, 4H), 1.30 – 1.25 (m, 1H).

^{13}C NMR (100 MHz, $CDCl_3$) δ 150.14, 143.18, 139.17, 138.83, 134.10, 134.02, 131.25, 130.22, 129.10, 129.05, 127.23, 126.33, 125.59, 124.82, 124.76, 124.24, 122.75, 118.12, 117.35, 116.17, 44.20, 34.38, 34.31, 26.91, 26.17.

HRMS (ESI) *m/z* Calcd for $C_{26}H_{21}BrNaOS_2^+$ [M + Na]$^+$: 515.0109, Found: 515.0110.

Optical Rotation: $[\alpha]_D^{25}$ = - 65.5° (c = 0.2, acetone).

HPLC analysis: Chiralcel IA-H (Hexane/i-PrOH = 90:10, flow rate = 1.0 mL/min, wave length = 254 nm), t_R = 15.497 min (major), t_R = 17.197 min (minor), 96% **ee**

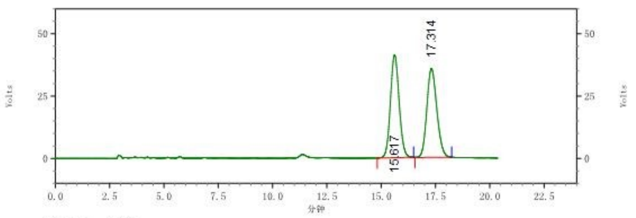

UV-WL1 结果

RetTime (min)	Width (min)	Height (Volts)	Area	Area (%)
15.617	1.67	164846	4594498	50.170
17.314	1.69	142533	4563419	49.830

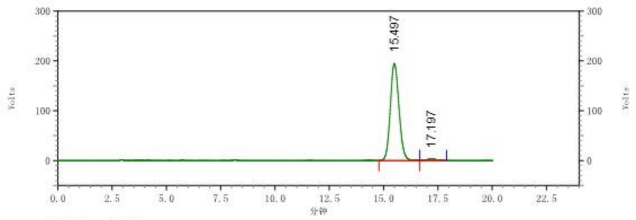

UV-WL1 结果

RetTime (min)	Width (min)	Height (Volts)	Area	Area (%)
15.497	1.88	778784	21726363	98.128
17.197	1.24	12577	414370	1.872

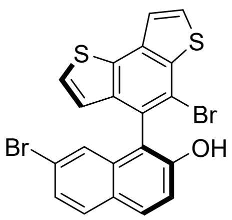

(*aS*)-7-bromo-1-(5-bromobenzo[1,2-b:3,4-b']dithiophen-4-yl)naphthalen-2-ol (2e)

Compound **2e** was synthesized in 79% yield (116.3 mg, 0.3 mmol scale) under condition [A].

White solid. (R_f = 0.3, PE/EA= 5:1)

^{1}H NMR (400 MHz, $CDCl_3$) δ 7.89 (d, *J* = 8.9 Hz, 1H), 7.76 (dt, *J* = 15.9, 7.2 Hz, 3H), 7.42 (d, *J* = 8.6 Hz, 1H), 7.38 – 7.28 (m, 2H), 7.22 (s, 1H), 6.73 (d, *J* = 5.4 Hz, 1H), 4.94 (s, 1H).
^{13}C NMR (100 MHz, $CDCl_3$) δ 151.64, 139.19, 138.53, 134.38, 134.37, 134.03, 130.53, 129.84, 129.45, 127.37, 127.10, 126.33, 126.10, 124.97, 124.26, 122.79, 121.60, 118.02, 117.64, 116.27.
HRMS (ESI) *m/z* Calcd for $C_{20}H_{10}Br_2NaOS_2^+$ [M + Na]$^+$: 510.8432, Found: 510.8434.

Optical Rotation: $[\alpha]_D^{25}$ = - 101.0° (*c* = 0.2, acetone).

HPLC analysis: Chiralcel IA-H (Hexane/*i*-PrOH = 90:10, flow rate = 1.0 mL/min, wave length = 254 nm), t_R = 11.180 min (minor), t_R = 16.563 min (major), 99% **ee**

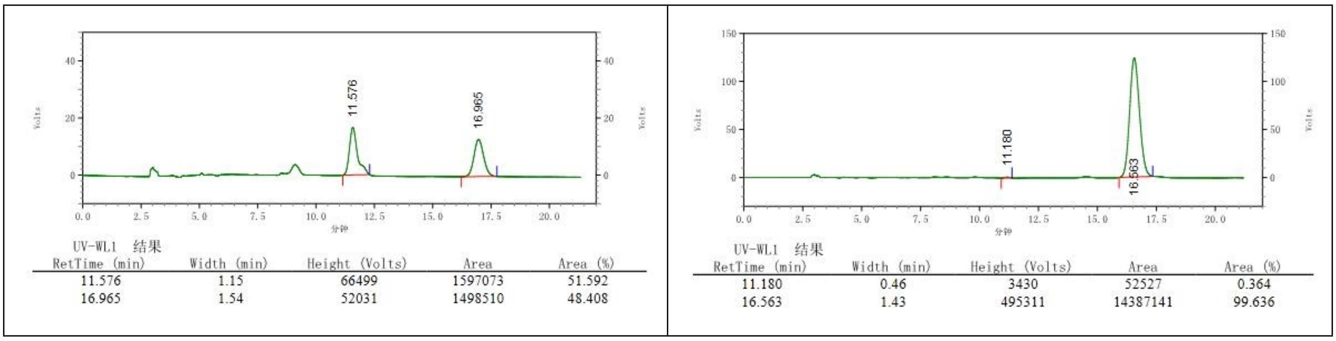

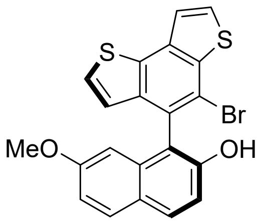

(*aS*)-1-(5-bromobenzo[1,2-b:3,4-b']dithiophen-4-yl)-7-methoxynaphthalen-2-ol (2f)

Compound **2f** was synthesized in 89% yield (117.5 mg, 0.3 mmol scale) under condition [A].

White solid. (R_f = 0.3, PE/EA= 5:1)

^{1}H NMR (400 MHz, $CDCl_3$) δ 7.83 (d, *J* = 8.8 Hz, 1H), 7.80 – 7.63 (m, 3H), 7.30 (d, *J* = 5.4 Hz, 1H), 7.16 (d, *J* = 8.8 Hz, 1H), 6.99 (dd, *J* = 8.9, 2.2 Hz, 1H), 6.78 (d, *J* = 5.4 Hz, 1H), 6.36 (d, *J* = 2.0 Hz, 1H), 4.91 (s, 1H), 3.51 (s, 3H).
^{13}C NMR (100 MHz, $CDCl_3$) δ 158.54, 151.39, 139.22, 138.66, 134.20, 134.10, 134.05, 130.29, 129.73, 129.09, 126.20, 125.66, 124.69, 124.31, 122.76, 117.49, 116.15, 115.37, 114.98, 103.75, 55.16.

HRMS (ESI) *m/z* Calcd for $C_{21}H_{13}BrNaO_2S_2^+$ $[M + Na]^+$: 462.9433, Found: 462.9433.

Optical Rotation: $[\alpha]_D^{25}$ = - 71.0° (*c* = 0.2, acetone).

HPLC analysis: Chiralcel IA-H (Hexane/*i*-PrOH = 90:10, flow rate = 1.0 mL/min, wave length = 254 nm), t_R = 14.652 min (minor), t_R = 23.736 min (major), 98% **ee**

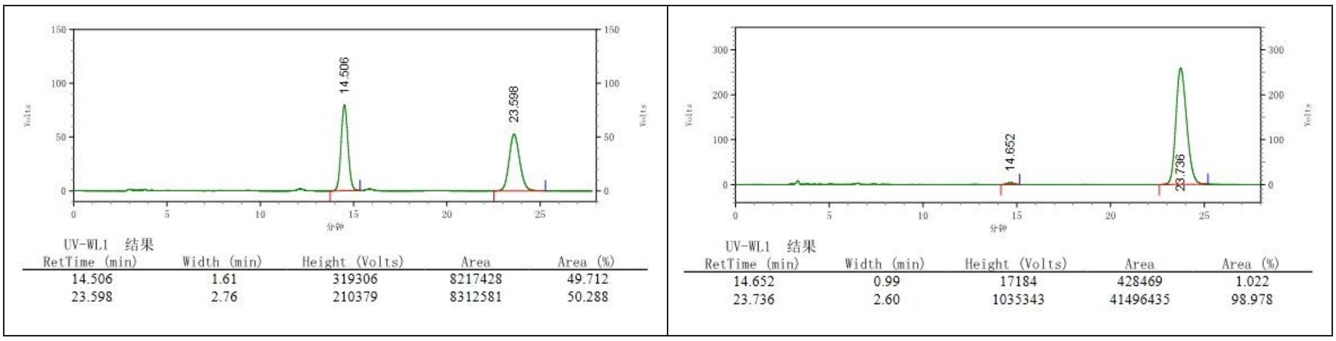

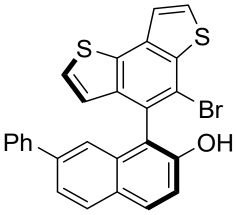

(*aS*)-1-(5-bromobenzo[1,2-b:3,4-b']dithiophen-4-yl)-7-phenylnaphthalen-2-ol (2g)

Compound **2d** was synthesized in 88% yield (128.7 mg, 0.3 mmol scale) under condition [A].

White solid. (R_f = 0.3, PE/EA= 5:1)

^{1}H NMR (400 MHz, CDCl) δ 7.96 (dd, *J* = 8.6, 3.7 Hz, 2H), 7.82 – 7.72 (m, 2H), 7.60 (d, *J* = 7.9 Hz, 1H), 7.40 (d, *J* = 7.4 Hz, 2H), 7.36 – 7.28 (m, 4H), 7.25 (d, *J* = 3.1 Hz, 2H), 6.81 (d, *J* = 5.4 Hz, 1H), 4.90 (s, 1H).

^{13}C NMR (100 MHz, CDCl) δ 151.21, 141.22, 139.80, 139.26, 138.75, 134.31, 134.22, 132.98, 130.35, 129.20, 128.72, 128.59, 128.15, 127.51, 127.22, 125.83, 124.61, 123.56, 122.81, 122.43, 118.56, 117.61, 116.25.

HRMS (ESI) *m/z* Calcd for $C_{26}H_{15}BrNaOS_2^+$ $[M + Na]^+$: 508.9640, Found: 508.9643.

Optical Rotation: $[\alpha]_D^{25}$ = - 54.5° (*c* = 0.2, acetone).

HPLC analysis: Chiralcel IA-H (Hexane/*i*-PrOH = 90:10, flow rate = 1.0 mL/min, wave length = 254 nm), t_R = 17.013 min (minor), t_R = 18.675 min (major), 99% **ee**

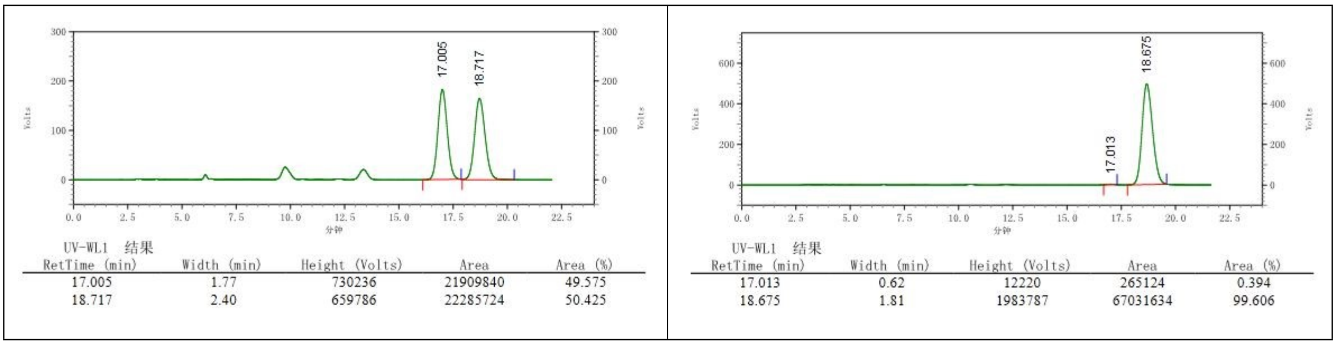

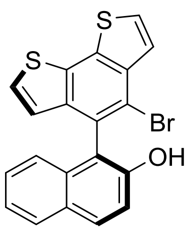

(*aS*)-1-(5-bromobenzo[2,1-b:3,4-b']dithiophen-4-yl)naphthalen-2-ol (2h)

Compound **2h** was synthesized in 89% yield (109.9 mg, 0.3 mmol scale) under condition [A].

White solid. (R_f = 0.3, PE/EA= 5:1)

^{1}H NMR (400 MHz, $CDCl_3$) δ 7.88 (dd, *J* = 22.4, 8.5 Hz, 2H), 7.68 (d, *J* = 5.4 Hz, 1H), 7.50 (d, *J* = 5.3 Hz, 1H), 7.31 (d, *J* = 8.8 Hz, 2H), 7.27 – 7.21 (m, 2H), 7.06 (d, *J* = 8.3 Hz, 1H), 6.69 (d, *J* = 5.3 Hz, 1H), 4.92 (s, 1H).
^{13}C NMR (100 MHz, $CDCl_3$) δ 150.62, 138.67, 137.49, 134.26, 133.29, 132.67, 130.45, 128.92, 128.15, 126.87, 126.54, 126.40, 125.73, 125.11, 124.83, 124.32, 123.58, 118.61, 118.26, 117.55.
HRMS (ESI) *m/z* Calcd for $C_{20}H_{11}BrNaOS_2^+$ [M + Na]$^+$: 432.9327, Found: 432.9327.

Optical Rotation: $[\alpha]_D^{25}$ = 4.8° (*c* = 0.5, acetone).

HPLC analysis: Chiralcel IA-H (Hexane/*i*-PrOH = 90:10, flow rate = 1.0 mL/min, wave length = 254 nm), t_R = 10.201 min (minor), t_R = 17.425 min (major), 93% **ee**

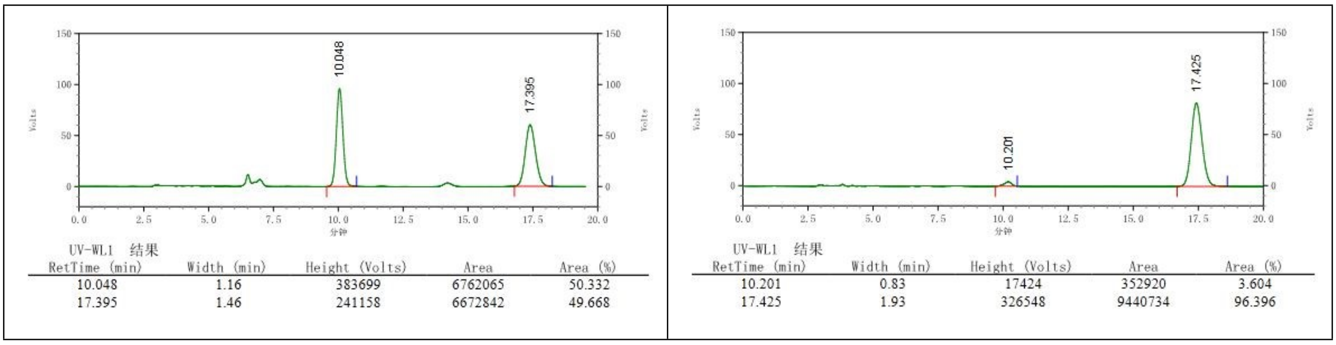

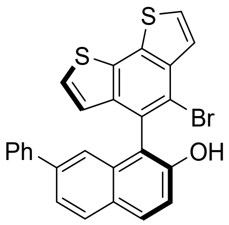

(*aS*)-1-(5-bromobenzo[2,1-b:3,4-b']dithiophen-4-yl)-7-phenylnaphthalen-2-ol (2i)

Compound **2i** was synthesized in 93% yield (136.0 mg, 0.3 mmol scale) under condition [A].

White solid. (R_f = 0.3, PE/EA= 5:1)

^{1}H NMR (400 MHz, $CDCl_3$) δ 7.93 (d, *J* = 8.4 Hz, 2H), 7.68 (d, *J* = 4.8 Hz, 1H), 7.58 (d, *J* = 8.1 Hz, 1H), 7.49 (d, *J* = 4.9 Hz, 1H), 7.39 (d, *J* = 7.1 Hz, 2H), 7.34 – 7.18 (m, 6H), 6.75 (d, *J* = 4.8 Hz, 1H), 4.93 (s, 1H).
^{13}C NMR (100 MHz, $CDCl_3$) δ 151.07, 141.16, 139.73, 138.61, 137.53, 134.35, 133.38, 132.92, 130.20, 128.71, 128.56, 128.13, 127.46, 127.17, 126.45, 126.38, 125.81, 125.09, 124.80, 123.50, 122.36, 118.92, 118.27, 117.62.

HRMS (ESI) *m/z* Calcd for $C_{26}H_{15}BrNaOS_2^+$ $[M + Na]^+$: 508.9640, Found: 508.9640.

Optical Rotation: $[\alpha]_D^{25}$ = 11.8° (c = 0.5, acetone).

HPLC analysis: Chiralcel IA-H (Hexane/i-PrOH = 90:10, flow rate = 1.0 mL/min, wave length = 254 nm), t_R = 12.938 min (minor), t_R = 19.270 min (major), 98% **ee**

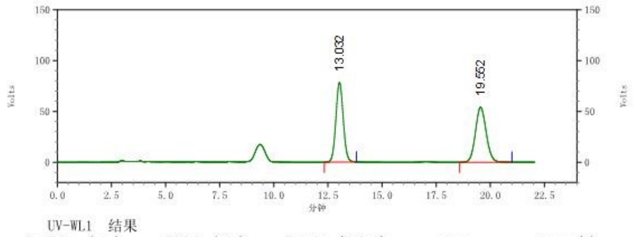

UV-WL1 结果

RetTime (min)	Width (min)	Height (Volts)	Area	Area (%)
13.032	1.49	313058	7402392	49.757
19.552	2.42	216310	7474587	50.243

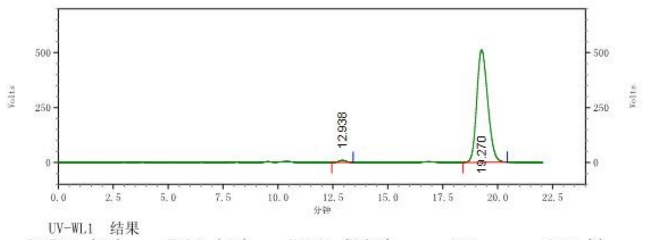

UV-WL1 结果

RetTime (min)	Width (min)	Height (Volts)	Area	Area (%)
12.938	0.97	38164	873422	1.215
19.270	2.03	2046037	71004027	98.785

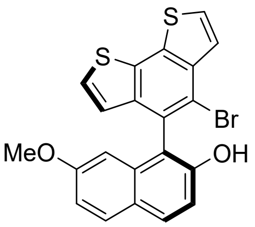

(*aS*)-1-(5-bromobenzo[2,1-b:3,4-b']dithiophen-4-yl)-7-methoxynaphthalen-2-ol (2j)

Compound **2j** was synthesized in 94% yield (124.5 mg, 0.3 mmol scale) under condition [A].

White solid. (R_f = 0.3, PE/EA= 5:1)

^{1}H NMR (400 MHz, $CDCl_3$) δ 7.82 (d, J = 8.8 Hz, 1H), 7.74 (d, J = 8.9 Hz, 1H), 7.68 (d, J = 5.4 Hz, 1H), 7.49 (d, J = 5.4 Hz, 1H), 7.25 (d, J = 5.4 Hz, 1H), 7.15 (d, J = 8.8 Hz, 1H), 6.98 (dd, J = 8.9, 2.1 Hz, 1H), 6.73 (d, J = 5.4 Hz, 1H), 6.36 (d, J = 1.6 Hz, 1H), 4.92 (s, 1H), 3.51 (s, 3H).

^{13}C NMR (100 MHz, $CDCl_3$) δ 158.52, 151.27, 138.56, 137.54, 134.22, 134.05, 133.31, 130.17, 129.72, 126.73, 126.47, 125.68, 125.03, 124.92, 124.32, 118.20, 117.85, 115.36, 114.98, 103.68, 55.17.

HRMS (ESI) *m/z* Calcd for $C_{28}H_{21}BrNaO^+$ $[M + Na]^+$: 462.9433, Found: 462.9435.

Optical Rotation: $[\alpha]_D^{25}$ = 48.0° (c = 0.5, acetone).

HPLC analysis: Chiralcel IA-H (Hexane/i-PrOH = 90:10, flow rate = 1.0 mL/min, wave length = 254 nm), t_R = 11.747 min (minor), t_R = 20.553 min (major), 91% **ee**

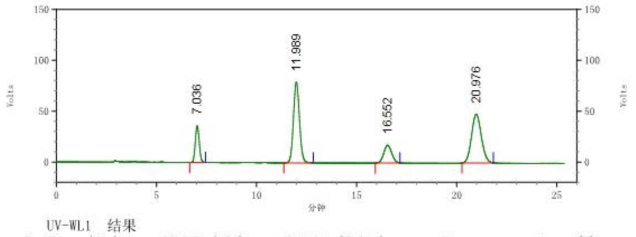

UV-WL1 结果

RetTime (min)	Width (min)	Height (Volts)	Area	Area (%)
7.036	0.80	143455	1982807	11.592
11.989	1.48	317681	6648697	38.871
16.552	1.24	69662	1943528	11.363
20.976	1.57	192042	6529343	38.174

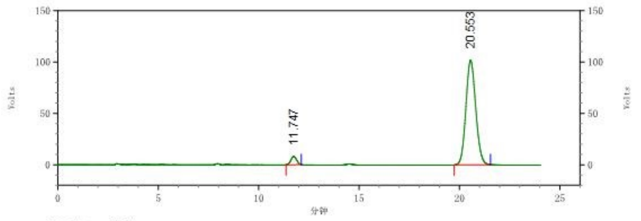

UV-WL1 结果

RetTime (min)	Width (min)	Height (Volts)	Area	Area (%)
11.747	0.76	31980	635449	4.383
20.553	1.81	407146	13861422	95.617

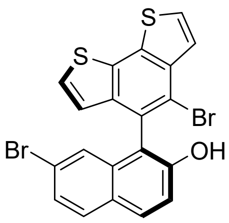

(*aS*)-7-bromo-1-(5-bromobenzo[2,1-b:3,4-b']dithiophen-4-yl)naphthalen-2-ol (2k)

Compound **2k** was synthesized in 92% yield (134.5 mg, 0.3 mmol scale) under condition [A].

White solid. (R_f = 0.3, PE/EA= 5:1)

^{1}H NMR (400 MHz, $CDCl_3$) δ 7.87 (d, *J* = 8.9 Hz, 1H), 7.71 (d, *J* = 8.7 Hz, 1H), 7.68 (d, *J* = 5.5 Hz, 1H), 7.53 (d, *J* = 5.4 Hz, 1H), 7.41 (d, *J* = 8.6 Hz, 1H), 7.32 (d, *J* = 8.9 Hz, 1H), 7.28 (d, *J* = 5.4 Hz, 1H), 7.22 (d, *J* = 5.5 Hz, 1H), 6.68 (d, *J* = 5.4 Hz, 1H), 4.99 (s, 1H).

^{13}C NMR (100 MHz, $CDCl_3$) δ 151.52, 138.37, 137.48, 134.56, 133.98, 133.48, 130.38, 129.82, 127.35, 127.04, 126.38, 126.28, 126.06, 125.57, 125.28, 124.47, 121.56, 118.27, 118.03.

HRMS (ESI) *m/z* Calcd for $C_{20}H_{10}Br_2NaOS_2^+$ $[M + Na]^+$: 510.8432, Found: 510.8432.

Optical Rotation: $[\alpha]_D^{25}$ = 5.0° (*c* = 0.5, acetone).

HPLC analysis: Chiralcel IA-H (Hexane/*i*-PrOH = 90:10, flow rate = 1.0 mL/min, wave length = 254 nm), t_R = 10.483 min (minor), t_R = 17.864 min (major), 98% **ee**

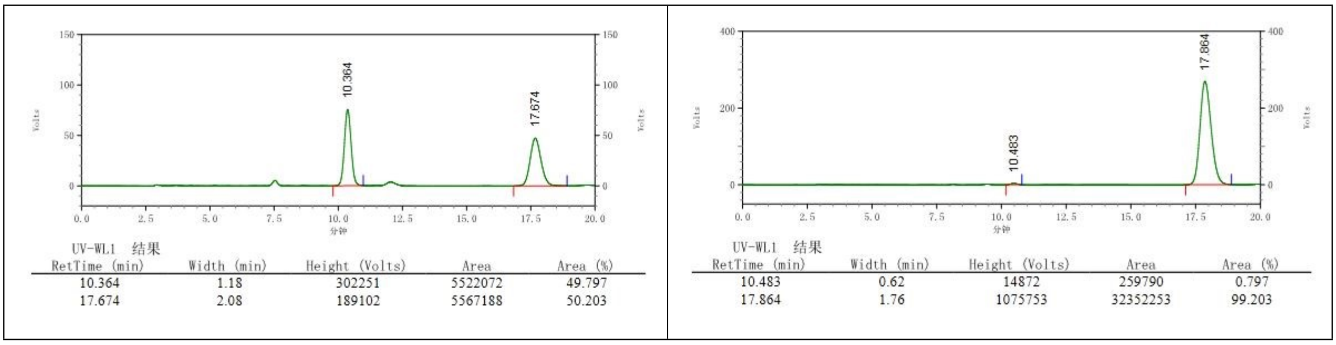

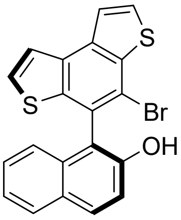

(*aS*)-1-(5-bromobenzo[1,2-b:4,3-b']dithiophen-4-yl)naphthalen-2-ol (2l)

Compound **2l** was synthesized in 76% yield (93.3 mg, 0.3 mmol scale) under condition [A].

White solid. (R_f = 0.3, PE/EA= 4:1)

^{1}H NMR (400 MHz, $CDCl_3$) δ 7.94 (d, *J* = 8.7 Hz, 1H), 7.91 – 7.80 (m, 2H), 7.73 (d, *J* = 4.6 Hz, 1H), 7.70 (d, *J* = 4.6 Hz, 1H), 7.48 (d, *J* = 4.5 Hz, 1H), 7.41 – 7.23 (m, 3H), 7.10 (d, *J* = 7.9 Hz, 1H), 4.98 (s, 1H).

^{13}C NMR (100 MHz, $CDCl_3$) δ 150.71, 140.18, 139.81, 134.93, 134.07, 132.09, 131.08, 129.05, 128.51, 128.40, 128.27, 127.03, 125.02, 123.98, 123.74, 123.00, 122.10, 118.10, 117.68, 115.58.

HRMS (ESI) *m/z* Calcd for $C_{20}H_{11}BrNaOS_2^+$ $[M + Na]^+$: 432.9327, Found: 432.9328.

Optical Rotation: $[\alpha]_D^{25}$ = 4.8° (*c* = 0.5, acetone).

HPLC analysis: Chiralcel AS-H (Hexane/*i*-PrOH = 80:20, flow rate = 1.0 mL/min, wave length = 254 nm), t_R = 11.019 min (major), t_R = 14.383 min (minor), 97% **ee**

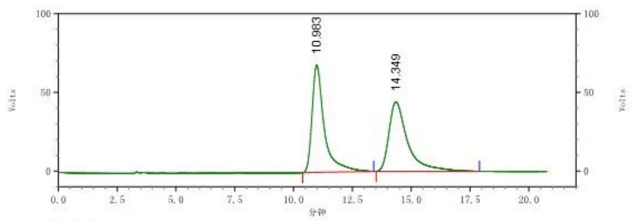

UV-WL1 结果

RetTime (min)	Width (min)	Height (Volts)	Area	Area (%)
10.983	3.01	272501	9910353	50.371
14.349	4.39	176638	9764249	49.629

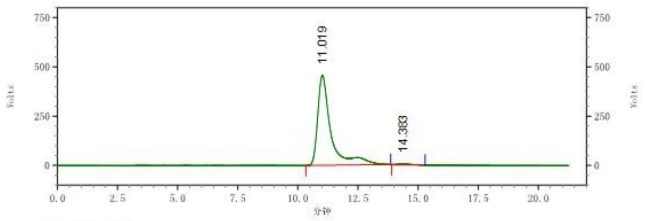

UV-WL1 结果

RetTime (min)	Width (min)	Height (Volts)	Area	Area (%)
11.019	3.53	1823617	70097790	98.476
14.383	1.40	22439	1084813	1.524

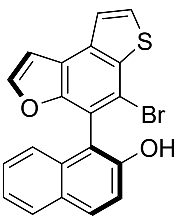

(*aS*)-1-(5-bromothieno[3,2-e]benzofuran-4-yl)naphthalen-2-ol (2m)

Compound **2m** was synthesized in 97% yield (114.7 mg, 0.3 mmol scale) under condition [A].

White solid. (R_f = 0.3, PE/EA= 5:1)

^{1}H NMR (400 MHz, $CDCl_3$) δ 7.96 (d, *J* = 8.9 Hz, 1H), 7.88 (d, *J* = 8.0 Hz, 1H), 7.80 (d, *J* = 5.5 Hz, 1H), 7.75 (d, *J* = 5.5 Hz, 1H), 7.62 (d, *J* = 2.0 Hz, 1H), 7.35 (q, *J* = 8.7 Hz, 2H), 7.30 (t, *J* = 7.1 Hz, 1H), 7.14 (d, *J* = 2.0 Hz, 1H), 7.10 (d, *J* = 8.3 Hz, 1H), 4.95 (s, 1H).

^{13}C NMR (100 MHz, $CDCl_3$) δ 151.97, 151.20, 145.79, 138.22, 132.85, 132.77, 130.98, 129.12, 128.95, 128.28, 126.94, 124.16, 123.64, 122.76, 121.92, 117.59, 116.69, 114.81, 114.53, 105.71.

HRMS (ESI) *m/z* Calcd for $C_{26}H_{15}BrNaO_2S^+$ $[M + Na]^+$: 416.9555, Found: 416.9557.

Optical Rotation: $[\alpha]_D^{25}$ = - 59.0° (*c* = 0.3, acetone).

HPLC analysis: Chiralcel IA-H (Hexane/*i*-PrOH = 85:15, flow rate = 1.0 mL/min, wave length = 254 nm), t_R = 12.337 min (minor), t_R = 14.200 min (major), 96% **ee**

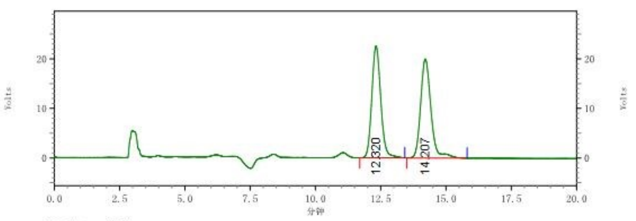

UV-WL1 结果

RetTime (min)	Width (min)	Height (Volts)	Area	Area (%)
12.320	1.72	90318	2153299	49.638
14.207	2.31	79916	2184686	50.362

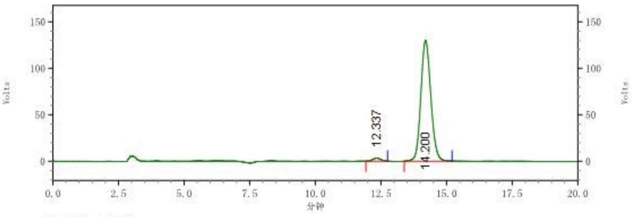

UV-WL1 结果

RetTime (min)	Width (min)	Height (Volts)	Area	Area (%)
12.337	0.83	12706	280263	1.998
14.200	1.82	519614	13745393	98.002

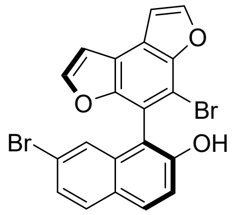

(*aS*)-7-bromo-1-(5-bromobenzo[1,2-b:4,3-b']difuran-4-yl)naphthalen-2-ol (2n)

Compound **2n** was synthesized in 90% yield (123.0 mg, 0.3 mmol scale) under condition [A].

White solid. (R_f = 0.4, PE/EA= 5:1)

^{1}H NMR (400 MHz, $CDCl_3$) δ 7.88 (s, 2H), 7.71 (d, *J* = 8.4 Hz, 1H), 7.61 (s, 1H), 7.41 (d, *J* = 8.2 Hz, 1H), 7.33 (d, *J* = 8.7 Hz, 1H), 7.23 (s, 1H), 7.14 (s, 1H), 7.02 (s, 1H), 5.10 (s, 1H).

^{13}C NMR (100 MHz, $CDCl_3$) δ 152.09, 150.51, 149.47, 146.57, 146.39, 134.25, 130.84, 129.90, 127.40, 127.03, 126.17, 121.57, 121.05, 119.84, 118.03, 114.48, 113.50, 106.66, 105.84, 104.26.

HRMS (ESI) *m/z* Calcd for $C_{20}H_{10}Br_2NaO_3^+$ [M + Na]$^+$: 478.8889, Found: 478.8886.

Optical Rotation: $[\alpha]_D^{25}$ = - 23.8° (*c* = 0.5, acetone).

HPLC analysis: Chiralcel IA-H (Hexane/*i*-PrOH = 85:15, flow rate = 1.0 mL/min, wave length = 254 nm), t_R = 12.167 min (major), t_R = 13.200 min (minor), 93% **ee**

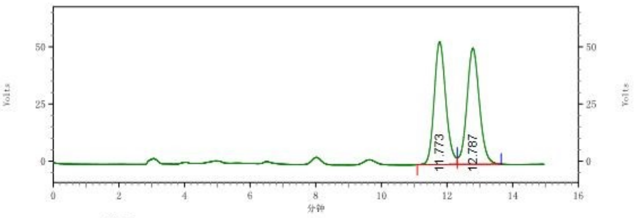

UV-WL1 结果

RetTime (min)	Width (min)	Height (Volts)	Area	Area (%)
11.773	1.22	214323	5260621	49.509
12.787	1.34	203106	5364882	50.491

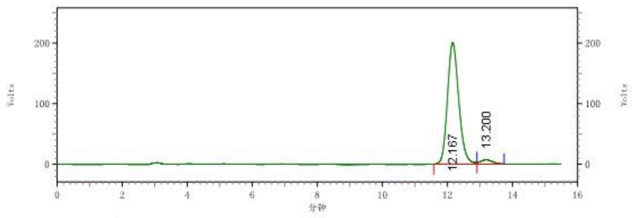

UV-WL1 结果

RetTime (min)	Width (min)	Height (Volts)	Area	Area (%)
12.167	1.33	801926	19382731	96.427
13.200	0.84	28106	718126	3.573

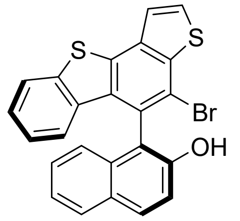

(*aS*)-1-(4-bromobenzo[b]thieno[2,3-g]benzothiophene-5-yl)naphthalen-2-ol (2o)

Compound **2o** was synthesized in 88% yield (121.4 mg, 0.3 mmol scale) under condition [A].

Yellow solid. (R_f = 0.4, PE/EA= 5:1)

^{1}H NMR (400 MHz, $CDCl_3$) δ 8.04 (d, *J* = 8.9 Hz, 1H), 7.92 (d, *J* = 8.2 Hz, 1H), 7.83 (d, *J* = 8.0 Hz, 1H), 7.80 – 7.74 (m, 2H), 7.39 (d, *J* = 8.9 Hz, 1H), 7.34 (t, *J* = 7.5 Hz, 1H), 7.29 (d, *J* = 7.5 Hz, 1H), 7.25 (d, *J* = 6.9 Hz, 1H), 7.21 (d, *J* = 7.2 Hz, 1H), 7.10 (d, *J* = 8.4 Hz, 1H), 6.92 (t, *J* = 7.8 Hz, 1H), 6.53 (d, *J* = 8.3 Hz, 1H), 4.91 (s, 1H).
^{13}C NMR (100 MHz, $CDCl_3$) δ 150.67, 140.81, 138.91, 135.18, 134.80, 134.29, 132.63, 132.54, 130.95, 129.57, 129.10, 128.25, 127.26, 126.42, 126.31, 124.93, 123.96, 123.89, 123.79, 123.10, 122.61, 118.41, 117.72, 117.22.
HRMS (ESI) *m/z* Calcd for $C_{24}H_{13}BrNaOS_2^+$ [M + Na]$^+$: 482.9483, Found: 482.9485.

Optical Rotation: $[\alpha]_D^{25}$ = - 44.5° (*c* = 0.6, acetone).

HPLC analysis: Chiralcel IA-H (Hexane/*i*-PrOH = 85:15, flow rate = 1.0 mL/min, wave length = 254 nm), t_R = 9.560 min (minor), t_R = 11.943 min (major), 98% **ee**

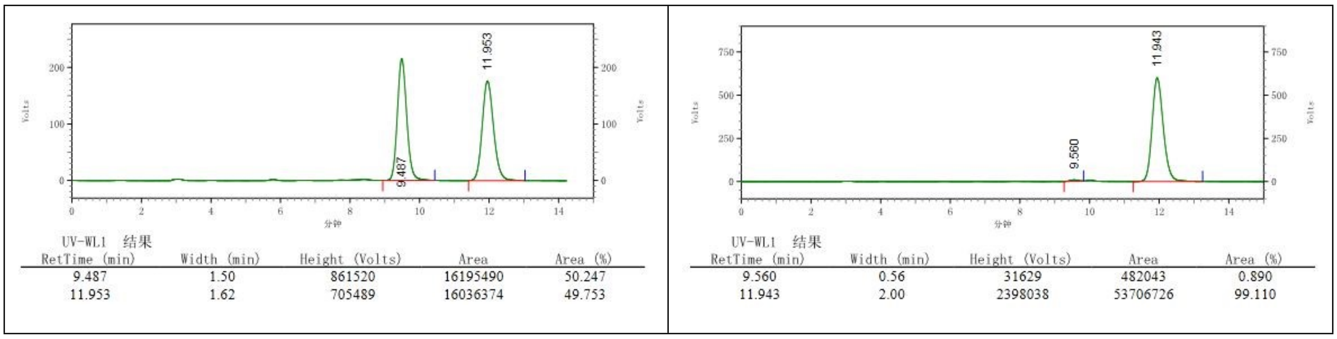

UV-WL1 结果

RetTime (min)	Width (min)	Height (Volts)	Area	Area (%)
9.487	1.50	861520	16195490	50.247
11.953	1.62	705489	16036374	49.753

UV-WL1 结果

RetTime (min)	Width (min)	Height (Volts)	Area	Area (%)
9.560	0.56	31629	482043	0.890
11.943	2.00	2398038	53706726	99.110

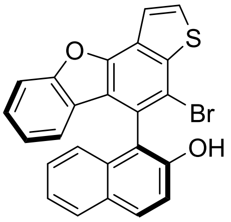

(*aS*)-1-(4-bromobenzo[b]thieno[2,3-g]benzofuran-5-yl)naphthalen-2-ol (2p)

Compound **2p** was synthesized in 90% yield (120.0 mg, 0.3 mmol scale) under condition [A].

Brown solid. (R_f = 0.5, PE/EA = 4:1)

^{1}H NMR (400 MHz, $CDCl_3$) δ 8.02 (d, *J* = 8.9 Hz, 1H), 7.93 (dd, *J* = 14.8, 6.8 Hz, 2H), 7.70 (d, *J* = 5.5 Hz, 1H), 7.58 (d, *J* = 8.3 Hz, 1H), 7.39 (d, *J* = 8.9 Hz, 1H), 7.36 – 7.28 (m, 2H), 7.23 (t, *J* = 3.3 Hz, 1H), 7.14 (d, *J* = 8.5 Hz, 1H), 6.91 (t, *J* = 7.6 Hz, 1H), 6.38 (d, *J* = 7.8 Hz, 1H), 5.01 (s, 1H).
^{13}C NMR (100 MHz, $CDCl_3$) δ 155.83, 150.68, 150.07, 142.42, 132.44, 130.91, 129.03, 128.97, 128.25, 127.18, 126.87, 125.93, 124.88, 124.00, 123.84, 123.56, 123.33, 121.32, 121.18, 120.53, 117.65, 117.09, 113.15, 111.50.
HRMS (ESI) *m/z* Calcd for $C_{24}H_{13}BrNaO_2S^+$ [M + Na]$^+$: 466.9712, Found: 466.9716.

Optical Rotation: $[\alpha]_D^{25}$ = - 7.0° (*c* = 0.2, acetone).

HPLC analysis: Chiralcel IA-H (Hexane/*i*-PrOH = 85:15, flow rate = 1.0 mL/min, wave length = 254 nm), t_R = 7.603 min (minor), t_R = 10.400 min (major), 90% **ee**

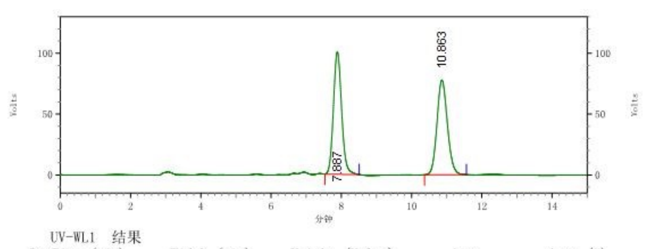

UV-WL1 结果

RetTime (min)	Width (min)	Height (Volts)	Area	Area (%)
7.887	0.98	401996	6542422	50.465
10.863	1.19	311336	6421847	49.535

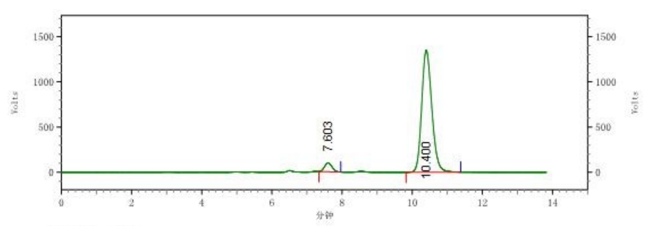

UV-WL1 结果

RetTime (min)	Width (min)	Height (Volts)	Area	Area (%)
7.603	0.63	389768	5805044	5.080
10.400	1.56	5392435	108456242	94.920

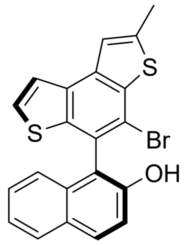

(*aS*)-1-(5-bromo-7-methylbenzo[1,2-b:4,3-b']dithiophen-4-yl)naphthalen-2-ol (2q)

Compound **2q** was synthesized in 92% yield (98.2 mg, 0.25 mmol scale) under condition [A].

Yellow solid. (R_f = 0.4, PE/EA= 5:1)

^{1}H NMR (400 MHz, $CDCl_3$) δ 7.93 (d, *J* = 8.9 Hz, 1H), 7.86 (d, *J* = 8.1 Hz, 1H), 7.65 (d, 3.6Hz, 1H), 7.53 (s, 1H), 7.43 (d, *J* = 5.5 Hz, 1H), 7.37 – 7.22 (m, 3H), 7.10 (d, *J* = 8.5 Hz, 1H), 4.92 (s, 1H), 2.71 (s, 3H).

^{13}C NMR (100 MHz, $CDCl_3$) δ 150.78, 143.62, 140.13, 139.00, 135.59, 133.41, 132.19, 130.99, 129.09, 128.25, 128.00, 126.97, 124.05, 123.69, 122.03, 120.95, 118.24, 117.64, 115.17, 16.31.

HRMS (ESI) *m/z* Calcd for $C_{21}H_{13}BrNaOS_2^+$ [M + Na]$^+$: 446.9483, Found: 446.9484.

Optical Rotation: $[\alpha]_D^{25}$ = - 49.8° (*c* = 0.5, acetone).

HPLC analysis: Chiralcel OD-H (Hexane/*i*-PrOH = 80:20, flow rate = 1.0 mL/min, wave length = 254 nm), t_R = 9.643 min (minor), t_R = 18.527 min (major), 89% **ee**

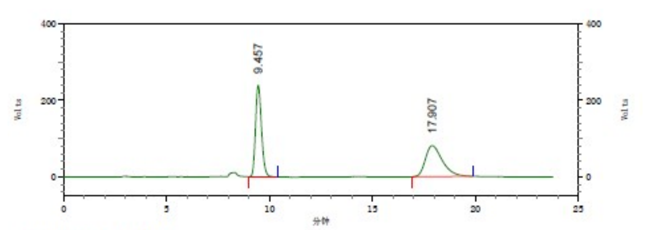

UV-WL1 254 nm 结果

RetTime (min)	Width (min)	Height (Volts)	Area	Area (%)
9.457	1.39	957247	19295174	50.853
17.907	2.95	324526	18647816	49.147

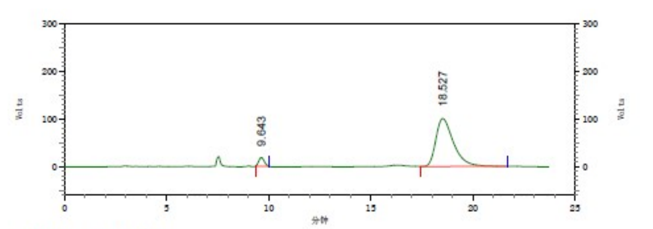

UV-WL1 254 nm 结果

RetTime (min)	Width (min)	Height (Volts)	Area	Area (%)
9.643	0.66	73724	1401755	5.491
18.527	4.25	402274	24128351	94.509

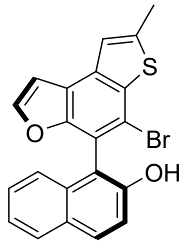

(*aS*)-1-(5-bromo-7-methylthieno[3,2-e]benzofuran-4-yl)naphthalen-2-ol (2r)

Compound **2r** was synthesized in 94% yield (77.2 mg, 0.2 mmol scale) under condition [A].

Pale yellow solid. (R_f = 0.4, PE/EA= 5:1)

^{1}H NMR (400 MHz, $CDCl_3$) δ 7.93 (d, *J* = 8.9 Hz, 1H), 7.86 (d, *J* = 8.0 Hz, 1H), 7.56 (s, 1H), 7.43 (s, 1H), 7.33 (d, *J* = 9.0 Hz, 2H), 7.29 (t, *J* = 7.5 Hz, 1H), 7.10 (d, *J* = 8.3 Hz, 1H), 7.03 (s, 1H), 4.92 (s, 1H), 2.71 (s, 3H).
^{13}C NMR (100 MHz, $CDCl_3$) δ 152.05, 151.22, 145.59, 144.25, 137.40, 133.42, 132.94, 130.89, 128.98, 128.26, 126.88, 124.22, 123.59, 121.22, 120.70, 117.53, 115.29, 114.63, 114.33, 105.59, 16.36.
HRMS (ESI) *m/z* Calcd for $C_{21}H_{13}BrNaO_2S^+$ $[M + Na]^+$: 430.9712, Found: 430.9714.

Optical Rotation: $[\alpha]_D^{25}$= - 29.7° (*c* = 0.3, acetone).

HPLC analysis: Chiralcel AD-H (Hexane/*i*-PrOH = 80:20, flow rate = 1.0 mL/min, wave length = 254 nm), t_R = 11.817 min (minor), t_R = 14.967 min (major), 95% **ee**

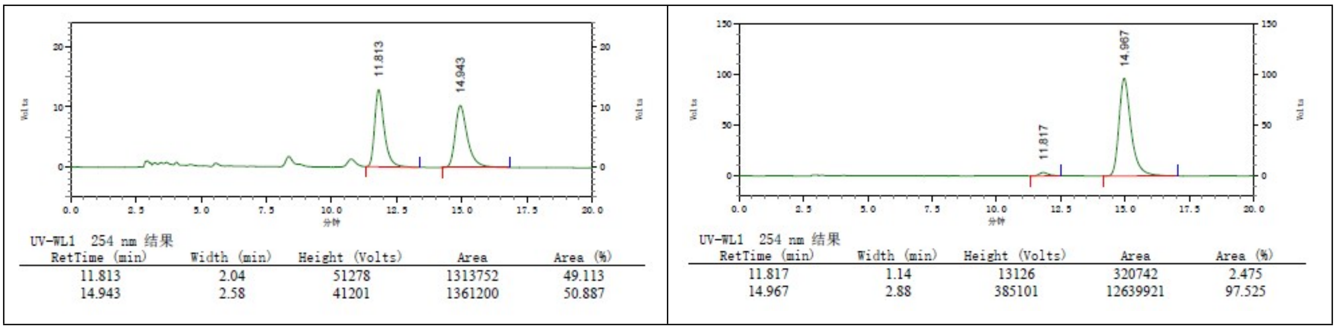

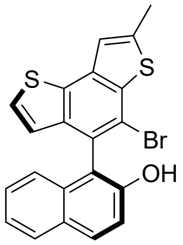

(*aS*)-1-(5-bromo-7-methylbenzo[1,2-b:3,4-b']dithiophen-4-yl)naphthalen-2-ol (2s)

Compound **2s** was synthesized in 95% yield (81.2 mg, 0.2 mmol scale) under condition [A].

White solid. (R_f = 0.4, PE/EA= 5:1)

^{1}H NMR (400 MHz, $CDCl_3$) δ 7.91 (d, *J* = 8.8 Hz, 1H), 7.85 (d, *J* = 8.0 Hz, 1H), 7.42 (s, 1H), 7.36 – 7.29 (m, 2H), 7.28 – 7.21 (m, 2H), 7.07 (d, *J* = 8.3 Hz, 1H), 6.71 (d, *J* = 5.2 Hz, 1H), 4.88 (s, 1H), 2.70 (s, 3H).
^{13}C NMR (100 MHz, $CDCl_3$) δ 150.81, 144.32, 138.74, 138.35, 134.68, 133.22, 132.84, 130.49, 128.94, 128.13, 126.85, 125.57, 124.78, 124.55, 124.43, 123.55, 120.65, 118.38, 117.50, 115.78, 16.29.
HRMS (ESI) *m/z* Calcd for $C_{21}H_{13}BrNaOS_2^+$ $[M + Na]^+$: 446.9483, Found: 446.9483 .

Optical Rotation: $[\alpha]_D^{25}$= - 43.3° (*c* = 1.0, acetone).

HPLC analysis: Chiralcel AD-H (Hexane/*i*-PrOH = 80:20, flow rate = 1.0 mL/min, wave length = 254 nm), t_R = 8.287 min (minor), t_R = 15.163 min (major), 97% **ee**

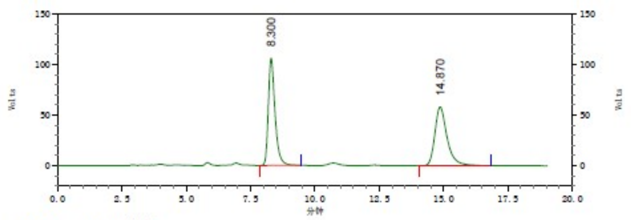

UV-WL1 254 nm 结果

RetTime (min)	Width (min)	Height (Volts)	Area	Area (%)
8.300	1.62	422678	7695841	50.038
14.870	2.78	232322	7684124	49.962

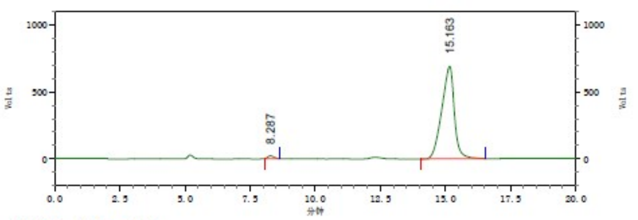

UV-WL1 254 nm 结果

RetTime (min)	Width (min)	Height (Volts)	Area	Area (%)
8.287	0.58	84877	1352126	1.442
15.163	2.49	2761383	92444305	98.558

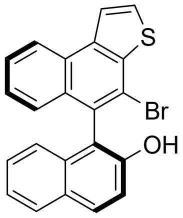

(*aS*)-1-(4-bromonaphtho[2,1-b]thiophen-5-yl)naphthalen-2-ol (2t)

Compound **2t** was synthesized in 90% yield (109.1 mg, 0.3 mmol scale) under condition [A].

White solid. (R_f = 0.4, PE/EA= 5:1)

^{1}H NMR (400 MHz, $CDCl_3$) δ 8.39 (d, *J* = 8.2 Hz, 1H), 8.16 (d, *J* = 4.9 Hz, 1H), 7.95 (d, *J* = 8.9 Hz, 1H), 7.88 (d, *J* = 8.1 Hz, 1H), 7.72 (d, *J* = 5.4 Hz, 1H), 7.61 (dt, *J* = 5.7, 2.8 Hz, 1H), 7.38 – 7.29 (m, 4H), 7.22 (t, *J* = 7.6 Hz, 2H), 7.00 (d, *J* = 8.4 Hz, 1H), 4.86 (s, 1H).

^{13}C NMR (100 MHz, $CDCl_3$) δ 151.02, 139.96, 137.17, 133.15, 131.63, 130.56, 129.00, 128.85, 128.53, 128.17, 127.91, 127.21, 126.99, 126.93, 126.74, 124.39, 124.02, 123.62, 123.07, 118.99, 118.07, 117.60.

HRMS (ESI) *m/z* Calcd for $C_{22}H_{13}BrNaOS^+$ $[M + Na]^+$: 426.9763, Found: 426.9765.

Optical Rotation: $[\alpha]_D^{25}$ = - 43.6° (*c* = 1.0, acetone).

HPLC analysis: Chiralcel IA-H (Hexane/*i*-PrOH = 80:20, flow rate = 1.0 mL/min, wave length = 254 nm), t_R = 7.827 min (minor), t_R = 11.787 min (major), 95% **ee**

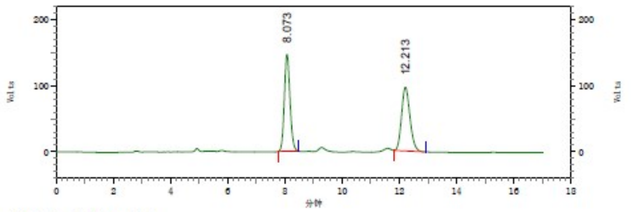

UV-WL1 254 nm 结果

RetTime (min)	Width (min)	Height (Volts)	Area	Area (%)
8.073	0.72	586695	8077143	50.630
12.213	1.13	385515	7876066	49.370

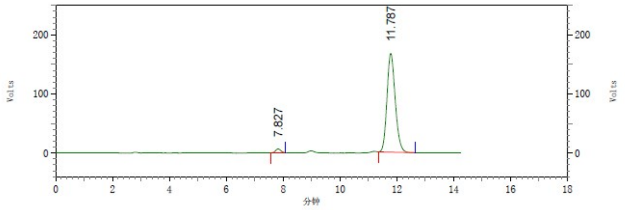

UV-WL1 254 nm 结果

RetTime (min)	Width (min)	Height (Volts)	Area	Area (%)
7.827	0.52	26767	342628	2.481
11.787	1.29	668656	13468333	97.519

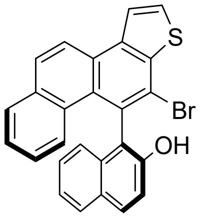

(*aR*)-1-(11-bromophenanthro[2,1-b]thiophen-10-yl)naphthalen-2-ol (2u)

Compound **2u** was synthesized in 67% yield (92.0 mg, 0.3 mmol scale) under condition [A].

Pale yellow solid. (R_f = 0.4, PE/EA= 6:1)

^{1}H NMR (400 MHz, $CDCl_3$) δ 8.40 (d, *J* = 8.9 Hz, 1H), 8.24 (d, *J* = 5.5 Hz, 1H), 8.02 (d, *J* = 8.9 Hz, 1H), 7.97 (d, *J* = 8.9 Hz, 1H), 7.91 (d, *J* = 8.2 Hz, 1H), 7.84 (d, *J* = 7.6 Hz, 1H), 7.76 (d, *J* = 5.5 Hz, 1H), 7.68 (d, *J* = 8.9 Hz, 1H), 7.40 – 7.28 (m, 3H), 7.19 (t, *J* = 7.4 Hz, 1H), 7.12 (d, *J* = 8.4 Hz, 1H), 6.96 (q, *J* = 8.1 Hz, 1H), 4.84 (s, 1H).
^{13}C NMR (100 MHz, $CDCl_3$) δ 150.23, 141.26, 137.91, 132.85, 132.82, 130.63, 130.46, 129.42, 129.40, 128.79, 128.50, 128.37, 128.24, 127.64, 127.21, 126.74, 126.31, 125.75, 124.17, 123.94, 123.58, 123.12, 122.75, 121.61, 117.91, 109.98.
HRMS (ESI) *m/z* Calcd for $C_{26}H_{15}BrNaOS^+$ $[M + Na]^+$: 476.9919, Found: 476.9916.

Optical Rotation: $[\alpha]_D^{25}$ = 10.4° (*c* = 1.0, acetone).

HPLC analysis: Chiralcel IA-H (Hexane/*i*-PrOH = 80:20, flow rate = 1.0 mL/min, wave length = 254 nm), t_R = 9.213 min (minor), t_R = 10.737 min (major), 90% **ee**

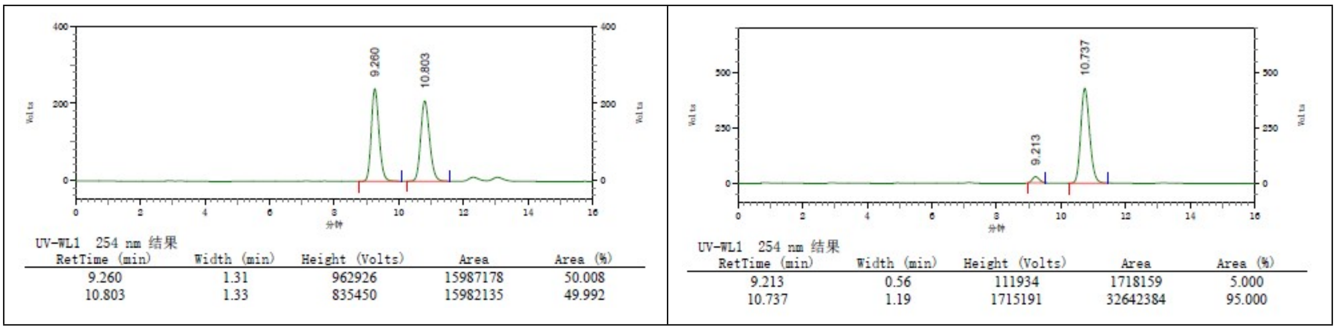

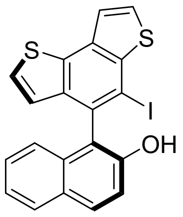

(*aS*)-1-(5-iodobenzo[1,2-b:3,4-b']dithiophen-4-yl)naphthalen-2-ol (2v)

Compound **2v** was synthesized in 92% yield (84.2 mg, 0.2 mmol scale).

White solid. (R_f = 0.5, PE/EA = 6:1)

^{1}H NMR (400 MHz, $CDCl_3$) δ 7.99 – 7.87 (m, 2H), 7.85 (d, *J* = 8.1 Hz, 1H), 7.69 (d, *J* = 5.6 Hz, 1H), 7.37 – 7.28 (m, 2H), 7.26 – 7.15 (m, 2H), 7.02 (d, *J* = 8.4 Hz, 1H), 6.71 (d, *J* = 4.8 Hz, 1H), 4.88 (s, 1H).
^{13}C NMR (100 MHz, $CDCl_3$) δ 150.47, 143.84, 138.19, 135.28, 132.85, 132.61, 131.06, 130.56, 128.91, 128.54, 128.12, 126.90, 125.50, 124.94, 124.36, 123.62, 123.19, 121.83, 117.59, 91.99.
HRMS (ESI) *m/z* Calcd for $C_{20}H_{11}INaOS_2^+$ $[M + Na]^+$: 480.9188, Found: 480.9185.

HPLC analysis: Chiralcel IA-H (Hexane/*i*-PrOH = 80:20, flow rate = 1.0 mL/min, wave length = 254 nm), t_R = 9.507 min (minor), t_R = 12.223 min (major), 16% **ee**

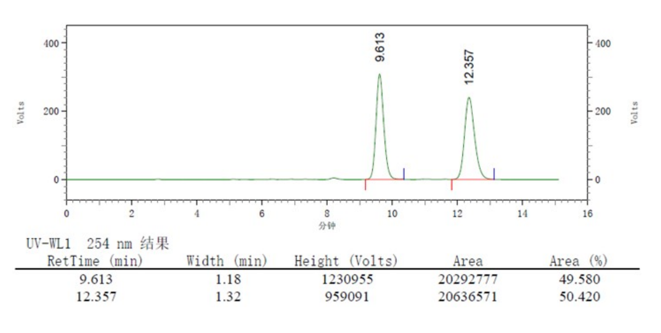

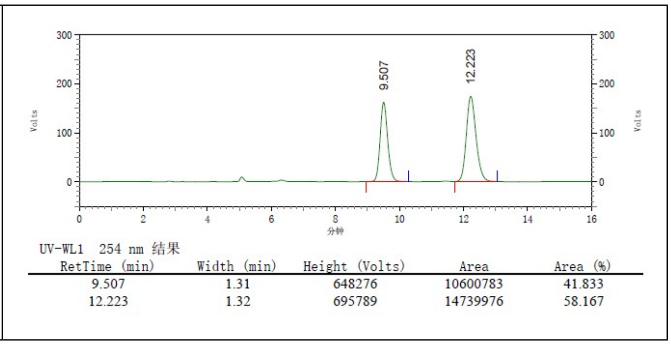

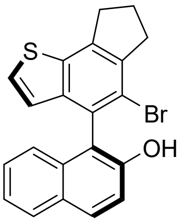

(*aS*)-1-(5-bromo-7,8-dihydro-6H-indeno[4,5-b]thiophen-4-yl)naphthalen-2-ol (2w)

Compound **2w** was synthesized in 90% yield (106.6 mg, 0.3 mmol scale) under condition [A].

White solid. (R_f = 0.5, PE/EA = 4:1)

^{1}H NMR (400 MHz, $CDCl_3$) δ 7.89 (d, *J* = 8.9 Hz, 1H), 7.84 (d, *J* = 7.9 Hz, 1H), 7.38 – 7.20 (m, 4H), 7.08 (d, *J* = 8.2 Hz, 1H), 6.65 (d, *J* = 5.6 Hz, 1H), 4.89 (s, 1H), 3.31 (td, *J* = 7.6, 3.5 Hz, 2H), 3.21 (q, *J* = 10.0, 8.7 Hz, 2H), 2.34 (p, *J* = 7.6 Hz, 2H).

^{13}C NMR (100 MHz, $CDCl_3$) δ 150.52, 141.48, 140.74, 138.98, 135.48, 132.74, 130.19, 128.90, 128.10, 127.20, 127.08, 126.70, 124.40, 124.18, 123.45, 120.61, 118.86, 117.45, 35.83, 33.84, 24.00.

HRMS (ESI) *m/z* Calcd for $C_{21}H_{15}BrNaOS^+$ $[M + Na]^+$: 416.9919, Found: 416.9919.

HPLC analysis: Chiralcel IA-H (Hexane/*i*-PrOH = 90:10, flow rate = 1.0 mL/min, wave length = 254 nm), t_R = 8.089 min (minor), t_R = 16.144 min (major), 71% **ee**. Racemic reference sample for chiral HPLC analysis were prepared following the general method using 5 mol% of (R)- and (S)-catalyst A.

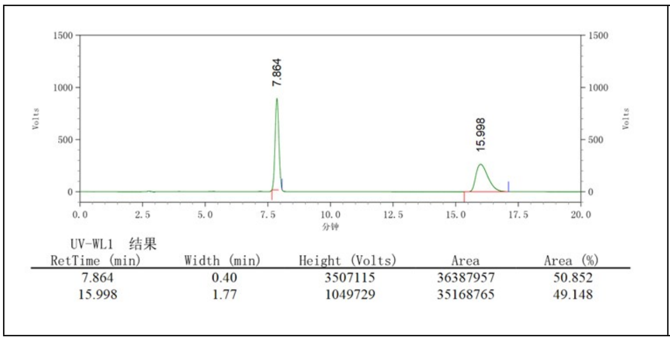

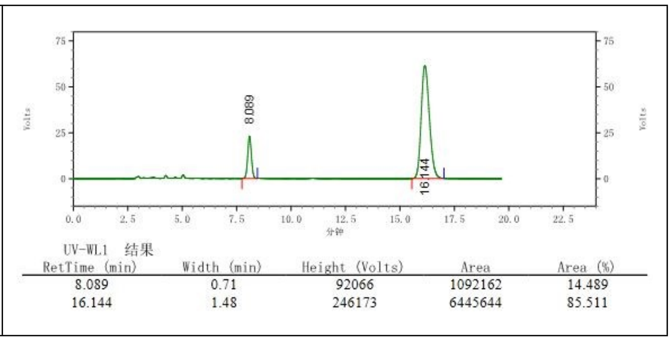

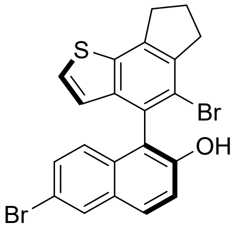

(*aS*)-6-bromo-1-(5-bromo-7,8-dihydro-6H-indeno[4,5-b]thiophen-4-yl)naphthalen-2-ol (2x)

Compound **2x** was synthesized in 89% yield (126.6 mg, 0.3 mmol scale) under condition [A].

White solid. (R_f = 0.5, PE/EA = 4:1)

^{1}H NMR (400 MHz, $CDCl_3$) δ 7.99 (d, *J* = 1.6 Hz, 1H), 7.79 (d, *J* = 8.9 Hz, 1H), 7.32 (d, *J* = 8.9 Hz, 2H), 7.27 (d, *J* = 5.5 Hz, 1H), 6.95 (d, *J* = 9.0 Hz, 1H), 6.62 (d, *J* = 5.5 Hz, 1H), 4.92 (s, 1H), 3.36 – 3.26 (m, 2H), 3.25 – 3.17 (m, 2H), 2.34 (p, *J* = 7.4 Hz, 2H).
^{13}C NMR (100 MHz, $CDCl_3$) δ 150.87, 141.54, 140.60, 139.28, 135.59, 131.30, 130.04, 129.92, 129.25, 127.40, 126.46, 126.28, 123.87, 120.52, 119.09, 118.65, 117.25, 35.81, 33.85, 23.98.
HRMS (ESI) *m/z* Calcd for $C_{21}H_{14}Br_2NaOS^+$ $[M + Na]^+$: 494.9024, Found: 494.9024.
HPLC analysis: Chiralcel IA-H (Hexane/*i*-PrOH = 90:10, flow rate = 1.0 mL/min, wave length = 254 nm), t_R = 8.263 min (minor), t_R = 17.052 min (major), 70% **ee**

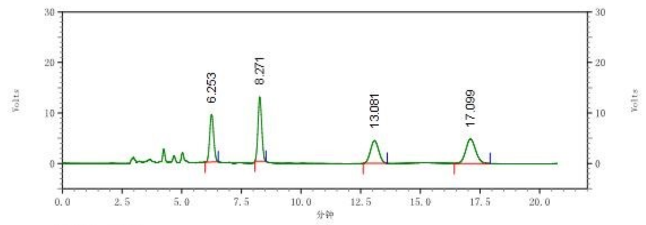

UV-WL1 结果

RetTime (min)	Width (min)	Height (Volts)	Area	Area (%)
6.253	0.55	37533	436307	21.983
8.271	0.46	50928	557455	28.087
13.081	1.01	18066	436424	21.989
17.099	1.51	19316	554542	27.940

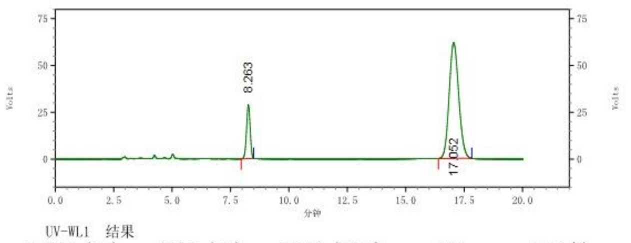

UV-WL1 结果

RetTime (min)	Width (min)	Height (Volts)	Area	Area (%)
8.263	0.54	115000	1231576	14.864
17.052	1.43	247222	7053965	85.136

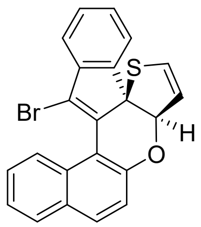

(4bR,7aR)-15-bromo-7aH-benzo[f]indeno[1,2-c]thieno[3,2-b]chromene (4a)

Compound **4a** was synthesized in 89% yield (107.6 mg, 0.3 mmol scale) under condition [A].

White solid. (R_f = 0.5, PE/EA = 20:1)

^{1}H NMR (400 MHz, $CDCl_3$) δ 7.97 (d, *J* = 8.4 Hz, 1H), 7.79 (t, *J* = 9.5 Hz, 2H), 7.53 (t, *J* = 8.6 Hz, 3H), 7.47 (t, *J* = 7.5 Hz, 1H), 7.40 (t, *J* = 7.2 Hz, 1H), 7.33 (t, *J* = 7.4 Hz, 1H), 7.20 (d, *J* = 8.8 Hz, 1H), 6.71 (d, *J* = 5.9 Hz, 1H), 5.96 (dd, *J* = 5.9, 3.0 Hz, 1H), 5.44 (d, *J* = 3.0 Hz, 1H).
^{13}C NMR (100 MHz, $CDCl_3$) δ 154.17, 146.61, 141.74, 134.95, 134.81, 130.52, 129.78, 129.55, 129.01, 128.31, 127.65, 126.62, 126.29, 124.09, 122.18, 121.76, 121.05, 118.75, 118.10, 113.93, 88.20, 69.53.
HRMS (ESI) *m/z* Calcd for $C_{22}H_{13}BrNaOS^+$ $[M + Na]^+$: 426.9763, Found: 426.9763.

Optical Rotation: $[\alpha]_D^{25}$ = - 95.0° (*c* = 0.5, acetone).

HPLC analysis: Chiralcel OD-H (Hexane/*i*-PrOH = 95:5, flow rate = 1.0 mL/min, wave length = 254 nm), t_R = 6.292 min (minor), t_R = 8.225 min (major), 90% **ee**

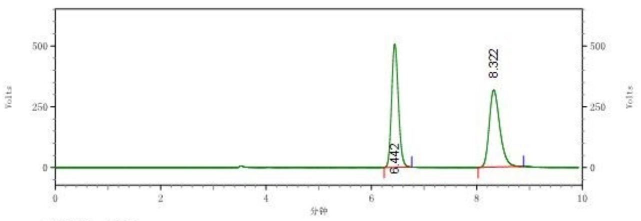

UV-WL1 结果

RetTime (min)	Width (min)	Height (Volts)	Area	Area (%)
6.442	0.52	2026317	18073556	50.549
8.322	0.86	1266836	17680957	49.451

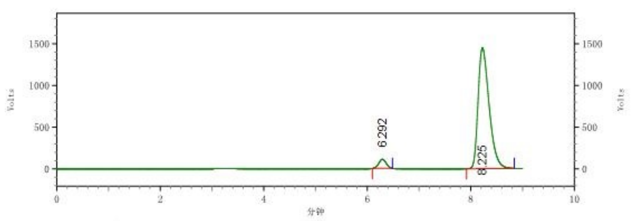

UV-WL1 结果

RetTime (min)	Width (min)	Height (Volts)	Area	Area (%)
6.292	0.39	439722	4427806	4.901
8.225	0.92	5788718	85908719	95.099

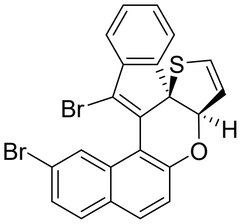

(4bR,7aR)-13,15-dibromo-7aH-benzo[f]indeno[1,2-c]thieno[3,2-b]chromene (4b)

Compound **4b** was synthesized in 87% yield (107.6 mg, 0.3 mmol scale) under condition [A].

White solid. (R_f = 0.6, PE/EA = 20:1)

^{1}H NMR (400 MHz, $CDCl_3$) δ 8.17 (s, 1H), 7.74 (d, *J* = 8.9 Hz, 1H), 7.67 (d, *J* = 8.7 Hz, 1H), 7.60 – 7.44 (m, 4H), 7.36 (t, *J* = 7.4 Hz, 1H), 7.21 (d, *J* = 8.9 Hz, 1H), 6.77 (d, *J* = 6.0 Hz, 1H), 6.00 (dd, *J* = 6.0, 3.0 Hz, 1H), 5.42 (d, *J* = 3.0 Hz, 1H).

^{13}C NMR (100 MHz, $CDCl_3$) δ 155.11, 146.35, 141.54, 135.19, 134.06, 130.88, 130.38, 129.85, 129.16, 128.92, 127.88, 127.40, 122.07, 121.74, 121.21, 120.90, 119.33, 118.51, 112.89, 88.30, 69.17.

HRMS (ESI) *m/z* Calcd for $C_{22}H_{12}Br_2NaOS^+$ $[M + Na]^+$: 504.8868, Found: 504.8866.

Optical Rotation: $[\alpha]^{25}_{D}$ = - 185.0° (*c* = 0.5, acetone).

HPLC analysis: Chiralcel AD-H (Hexane/*i*-PrOH = 95:5, flow rate = 1.0 mL/min, wave length = 254 nm), t_R = 5.519min (minor), t_R = 7.183 min (major), 96% **ee**

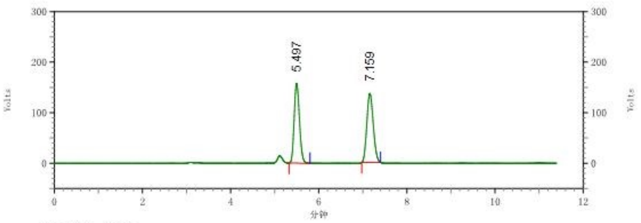

UV-WL1 结果

RetTime (min)	Width (min)	Height (Volts)	Area	Area (%)
5.497	0.48	631202	5244615	48.598
7.159	0.43	546154	5547263	51.402

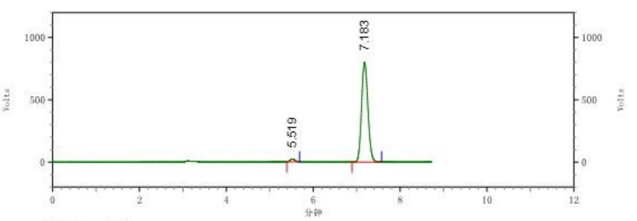

UV-WL1 结果

RetTime (min)	Width (min)	Height (Volts)	Area	Area (%)
5.519	0.29	92389	736172	2.212
7.183	0.68	3197857	32540710	97.788

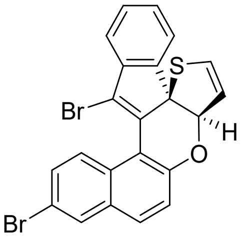

(4b*R*,7a*R*)-12,15-dibromo-7aH-benzo[f]indeno[1,2-c]thieno[3,2-b]chromene (4c)

Compound **4c** was synthesized in 95% yield (137.6 mg, 0.3 mmol scale) under condition [A].

White solid. (R_f = 0.5, PE/EA = 20:1)

^{1}H NMR (400 MHz, $CDCl_3$) δ 7.97 (s, 1H), 7.84 (d, *J* = 8.9 Hz, 1H), 7.70 (d, *J* = 8.9 Hz, 1H), 7.60 (d, *J* = 8.9 Hz, 1H), 7.57 – 7.44 (m, 3H), 7.36 (t, *J* = 7.5 Hz, 1H), 7.23 (d, *J* = 8.8 Hz, 1H), 6.76 (d, *J* = 5.9 Hz, 1H), 5.99 (dd, *J* = 6.0, 3.0 Hz, 1H), 5.44 (d, *J* = 3.0 Hz, 1H).
^{13}C NMR (100 MHz, $CDCl_3$) δ 154.46, 146.47, 141.53, 135.00, 134.32, 130.67, 130.21, 129.55, 129.46, 129.12, 128.34, 127.87, 122.13, 121.78, 121.18, 119.23, 117.87, 113.99, 88.33, 69.32.
HRMS (ESI) *m/z* Calcd for $C_{22}H_{12}Br_2NaOS^+$ $[M + Na]^+$: 504.8868, Found: 504.8868.

Optical Rotation: $[\alpha]_D^{25}$ = - 147.0° (*c* = 0.5, acetone).

HPLC analysis: Chiralcel AD-H (Hexane/*i*-PrOH = 95:5, flow rate = 1.0 mL/min, wave length = 254 nm), t_R = 5.551 min (minor), t_R = 6.929 min (major), 90% **ee**

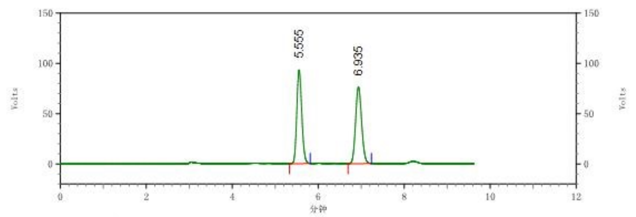

UV-WL1 结果

RetTime (min)	Width (min)	Height (Volts)	Area	Area (%)
5.555	0.49	372488	3012247	50.261
6.935	0.54	304852	2980986	49.739

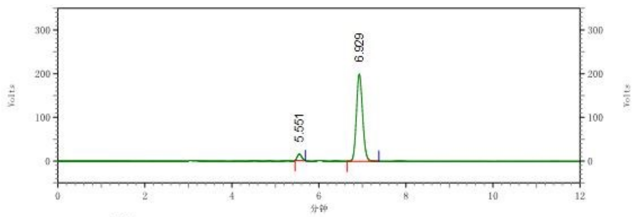

UV-WL1 结果

RetTime (min)	Width (min)	Height (Volts)	Area	Area (%)
5.551	0.24	58076	415397	5.028
6.929	0.73	798118	7846376	94.972

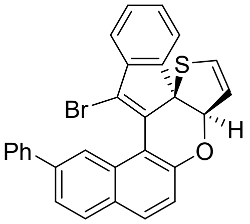

(4b*R*,7a*R*)-15-bromo-13-phenyl-7aH-benzo[f]indeno[1,2-c]thieno[3,2-b]chromene (4d)

Compound **4d** was synthesized in 89% yield (107.6 mg, 0.3 mmol scale) under condition [A].

White solid. (R_f = 0.5, PE/EA = 20:1)

^{1}H NMR (400 MHz, $CDCl_3$) δ 8.19 (s, 1H), 7.89 (d, *J* = 8.4 Hz, 1H), 7.82 (t, *J* = 7.6 Hz, 3H), 7.68 (dd, *J* = 4.2, 0.6 Hz 1H), 7.58 (d, *J* = 7.6 Hz, 1H), 7.53 (d, *J* = 7.5 Hz, 1H), 7.48 (q, *J* = 7.2 Hz, 3H), 7.36 (q, *J* = 6.6 Hz, 2H), 7.21 (d, *J* = 8.8 Hz, 1H), 6.74 (d, *J* = 5.9 Hz, 1H), 5.99 (dd, *J* = 5.9, 3.0 Hz, 1H), 5.46 (d, *J* = 2.9 Hz, 1H).
^{13}C NMR (100 MHz, $CDCl_3$) δ 154.58, 146.59, 141.72, 141.08, 139.08, 134.98, 134.92, 130.25, 130.05, 129.05, 128.88, 128.80, 128.74, 127.71, 127.51, 127.43, 124.69, 123.75, 122.17, 121.80, 121.08, 118.89, 118.09, 114.10, 88.23, 69.49.
HRMS (ESI) *m/z* Calcd for $C_{28}H_{17}BrNaOS^+$ $[M + Na]^+$: 503.0076, Found: 503.0077.

Optical Rotation: $[\alpha]_D^{25}$ = - 219.4.0° (*c* = 0.5, acetone).

HPLC analysis: Chiralcel OD-H (Hexane/*i*-PrOH = 95:5, flow rate = 1.0 mL/min, wave length = 254 nm), t_R = 6.331 min (minor), t_R = 7.183 min (major), 85% **ee**

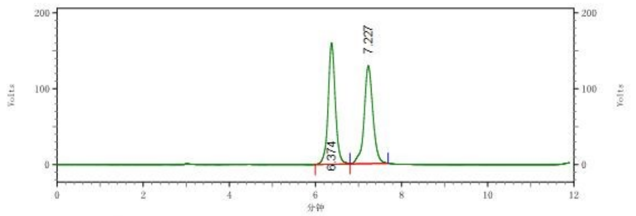

UV-WL1 结果

RetTime (min)	Width (min)	Height (Volts)	Area	Area (%)
6.374	0.80	639764	7414906	50.466
7.227	0.89	516082	7278047	49.534

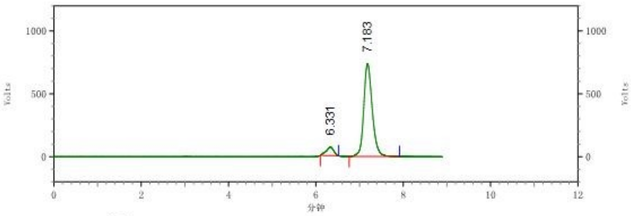

UV-WL1 结果

RetTime (min)	Width (min)	Height (Volts)	Area	Area (%)
6.331	0.42	272405	3241367	7.493
7.183	1.16	2954412	40014841	92.507

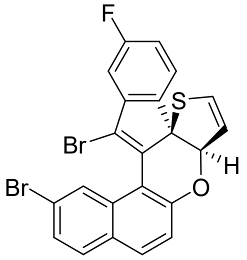

(4b*R*,7a*R*)-13,15-dibromo-2-fluoro-7aH-benzo[f]indeno[1,2-c]thieno[3,2-b]chromene (4e)

Compound **4e** was synthesized in 85% yield (127.5 mg, 0.3 mmol scale) under condition [A].

White solid. (R_f = 0.5, PE/EA = 20:1)

^{1}H NMR (400 MHz, $CDCl_3$) δ 8.12 (s, 1H), 7.74 (d, *J* = 8.9 Hz, 1H), 7.65 (d, *J* = 8.6 Hz, 1H), 7.46 (dd, *J* = 8.3, 4.6 Hz, 2H), 7.26 – 7.22 (m, 1H), 7.19 (d, *J* = 8.8 Hz, 1H), 7.02 (t, *J* = 7.8 Hz, 1H), 6.75 (d, *J* = 5.9 Hz, 1H), 5.98 (dd, *J* = 6.0, 3.0 Hz, 1H), 5.37 (d, *J* = 3.0 Hz, 1H).

^{13}C NMR (100 MHz, $CDCl_3$) δ 163.67 (d, *J* = 246.9 Hz), 155.14, 143.86 (d, *J* = 9.7 Hz), 141.84, 136.22, 135.12, 130.70, 129.87, 128.81, 127.86, 127.47, 123.04 (d, *J* = 9.2 Hz), 121.90, 121.01, 118.43, 117.90 (d, *J* = 3.2 Hz), 114.48 (d, *J* = 23.5 Hz), 112.43, 108.65 (d, *J* = 25.1 Hz), 88.15, 68.54.

HRMS (ESI) *m/z* Calcd for $C_{22}H_{11}Br_2FNaOS^+$ $[M + Na]^+$: 522.8774, Found: 522.8775.

Optical Rotation: $[\alpha]_D^{25}$ = - 228.0° (*c* = 0.4, acetone).

HPLC analysis: Chiralcel AD-H (Hexane/*i*-PrOH = 95:5, flow rate = 1.0 mL/min, wave length = 254 nm), t_R = 6.010 min (minor), t_R = 6.968 min (major), 88% **ee**

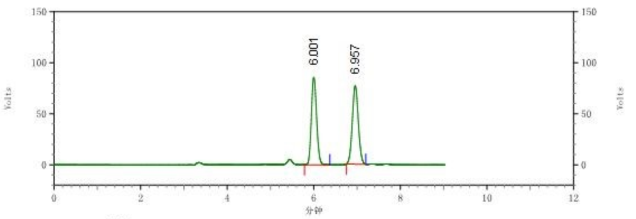

UV-WL1 结果

RetTime (min)	Width (min)	Height (Volts)	Area	Area (%)
6.001	0.58	342597	2861926	49.176
6.957	0.45	306809	2957802	50.824

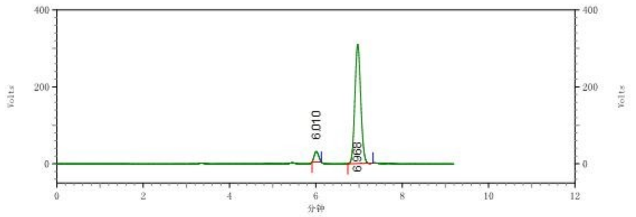

UV-WL1 结果

RetTime (min)	Width (min)	Height (Volts)	Area	Area (%)
6.010	0.22	109781	767773	6.164
6.968	0.58	1235730	11687494	93.836

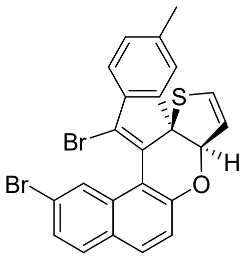

(4b*R*,7a*R*)-13,15-dibromo-3-methyl-7aH-benzo[f]indeno[1,2-c]thieno[3,2-b]chromene (4f)

Compound **4f** was synthesized in 89% yield (132.5 mg, 0.3 mmol scale) under condition [A].

White solid. (R_f = 0.5, PE/EA = 20:1)

^{1}H NMR (400 MHz, $CDCl_3$) δ 8.16 (s, 1H), 7.73 (d, *J* = 8.8 Hz, 1H), 7.66 (d, *J* = 8.6 Hz, 1H), 7.45 (dd, *J* = 13.8, 8.3 Hz, 2H), 7.34 (s, 1H), 7.29 (d, *J* = 7.8 Hz, 1H), 7.20 (d, *J* = 8.9 Hz, 1H), 6.77 (d, *J* = 5.9 Hz, 1H), 5.99 (dd, *J* = 6.0, 3.0 Hz, 1H), 5.40 (d, *J* = 3.0 Hz, 1H), 2.45 (s, 3H).
^{13}C NMR (100 MHz, $CDCl_3$) δ 155.01, 146.54, 139.03, 138.17, 135.18, 132.96, 130.91, 130.16, 129.84, 129.81, 128.94, 127.90, 127.35, 122.45, 122.03, 120.92, 120.80, 119.40, 118.50, 113.06, 88.39, 69.02, 21.62.
HRMS (ESI) *m/z* Calcd for $C_{23}H_{14}Br_2NaOS^+$ $[M + Na]^+$: 518.9024, Found: 518.9028.

Optical Rotation: $[\alpha]_D^{25}$ = - 185.0° (*c* = 0.5, acetone).

HPLC analysis: Chiralcel AD-H (Hexane/*i*-PrOH = 95:5, flow rate = 1.0 mL/min, wave length = 254 nm), t_R = 4.872 min (minor), t_R = 6.448 min (major), 90% **ee**

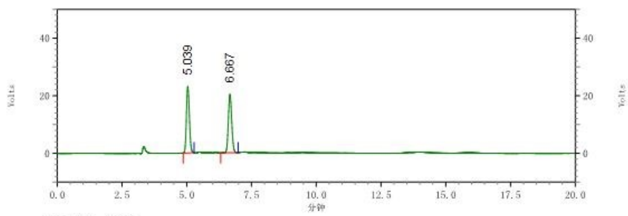

UV-WL1 结果

RetTime (min)	Width (min)	Height (Volts)	Area	Area (%)
5.039	0.42	92155	733244	49.787
6.667	0.67	81133	739520	50.213

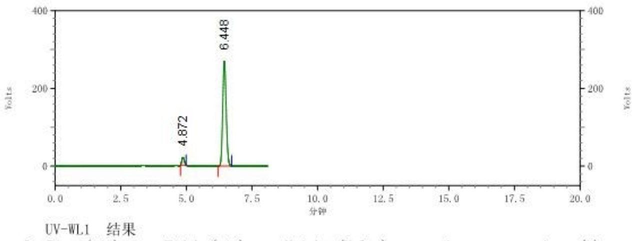

UV-WL1 结果

RetTime (min)	Width (min)	Height (Volts)	Area	Area (%)
4.872	0.21	79960	527805	5.037
6.448	0.52	1077479	9951592	94.963

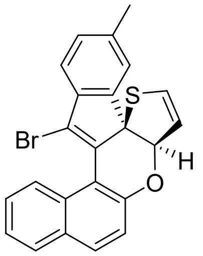

(4b*R*,7a*R*)-15-bromo-3-methyl-7aH-benzo[f]indeno[1,2-c]thieno[3,2-b]chromene (4g)

Compound **4g** was synthesized in 90% yield (107.6 mg, 0.3 mmol scale) under condition [B].

White solid. (R_f = 0.5, PE/EA = 20:1)

^{1}H NMR (400 MHz, $CDCl_3$) δ 8.04 (d, *J* = 8.4 Hz, 1H), 7.84 (dd, *J* = 13.8, 8.5 Hz, 2H), 7.59 (t, *J* = 7.6 Hz, 1H), 7.55 – 7.42 (m, 2H), 7.40 (s, 1H), 7.32 (d, *J* = 7.8 Hz, 1H), 7.26 (d, *J* = 8.8 Hz, 1H), 6.75 (d, *J* = 5.9 Hz, 1H), 6.01 (dd, *J* = 6.0, 3.0 Hz, 1H), 5.49 (d, *J* = 3.0 Hz, 1H), 2.49 (s, 3H).

^{13}C NMR (100 MHz, $CDCl_3$) δ 154.07, 146.76, 139.20, 137.88, 134.78, 133.86, 130.29, 129.80, 129.67, 129.52, 128.26, 126.63, 126.19, 124.02, 122.47, 122.13, 120.74, 118.79, 118.08, 114.06, 88.27, 69.37, 21.57.

HRMS (ESI) *m/z* Calcd for $C_{23}H_{15}BrNaOS^+$ $[M + Na]^+$: 440.9919, Found: 440.9920.

Optical Rotation: $[\alpha]_D^{25}$ = - 185.0° (*c* = 0.6, acetone).

HPLC analysis: Chiralcel AD-H (Hexane/*i*-PrOH = 95:5, flow rate = 1.0 mL/min, wave length = 254 nm), t_R = 4.448 min (minor), t_R = 6.694 min (major), 88% **ee**

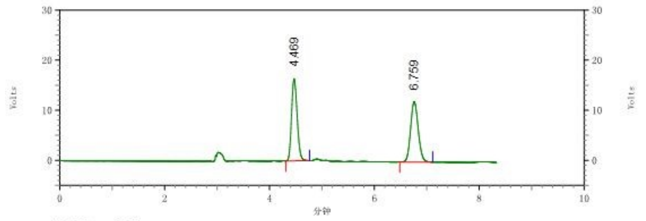

UV-WL1 结果

RetTime (min)	Width (min)	Height (Volts)	Area	Area (%)
4.469	0.45	65493	487115	50.026
6.759	0.63	48250	486600	49.974

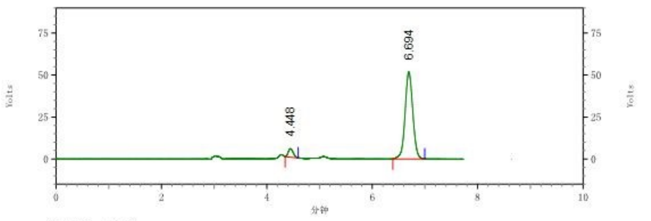

UV-WL1 结果

RetTime (min)	Width (min)	Height (Volts)	Area	Area (%)
4.448	0.25	20212	130222	5.815
6.694	0.61	207046	2109117	94.185

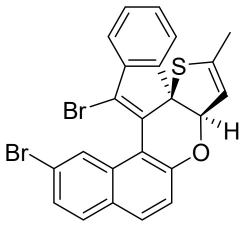

(4b*R*,7a*R*)-13,15-dibromo-6-methyl-7aH-benzo[f]indeno[1,2-c]thieno[3,2-b]chromene (4h)

Compound **4h** was synthesized in 92% yield (136.6 mg, 0.3 mmol scale) under condition [A].

White solid. (R_f = 0.5, PE/EA = 20:1)

^{1}H NMR (400 MHz, $CDCl_3$) δ 8.15 (s, 1H), 7.74 (d, *J* = 8.8 Hz, 1H), 7.66 (d, *J* = 8.6 Hz, 1H), 7.55 (dd, *J* = 7.6, 3.9 Hz, 2H), 7.48 (t, *J* = 8.2 Hz, 2H), 7.35 (t, *J* = 7.5 Hz, 1H), 7.20 (d, *J* = 8.9 Hz, 1H), 5.71 (s, 1H), 5.31 (d, *J* = 2.9 Hz, 1H), 2.07 (s, 3H).

^{13}C NMR (100 MHz, $CDCl_3$) δ 155.27, 148.30, 146.72, 141.40, 134.36, 130.89, 130.32, 129.81, 129.04, 128.94, 127.78, 127.25, 121.74, 121.09, 120.79, 119.03, 118.53, 118.23, 112.51, 89.30, 70.01, 16.90.

HRMS (ESI) *m/z* Calcd for $C_{23}H_{14}Br_2NaOS^+$ $[M + Na]^+$: 518.9024, Found: 518.9025.

Optical Rotation: $[\alpha]_D^{25}$ = - 18.5° (*c* = 0.2, acetone).

HPLC analysis: Chiralcel AD-H (Hexane/*i*-PrOH = 95:5, flow rate = 1.0 mL/min, wave length = 254 nm), t_R = 4.162 min (minor), t_R = 4.878 min (major), 86% **ee**

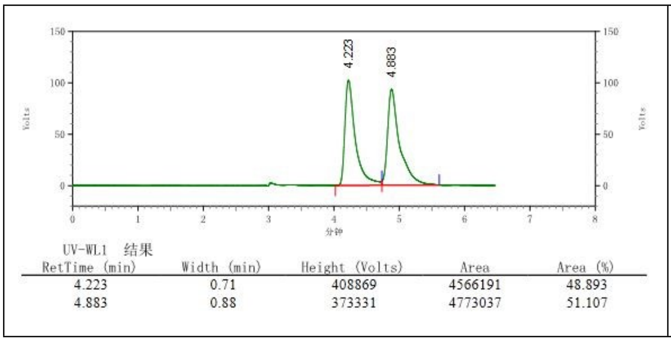

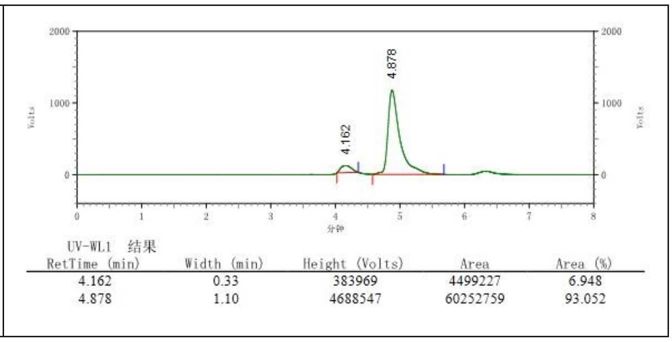

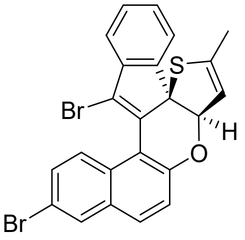

(4b*R*,7a*R*)-12,15-dibromo-6-methyl-7aH-benzo[f]indeno[1,2-c]thieno[3,2-b]chromene (4i)

Compound **4i** was synthesized in 92% yield (136.4 mg, 0.3 mmol scale) under condition [A].

White solid. (R_f = 0.5, PE/EA = 20:1)

^{1}H NMR (400 MHz, $CDCl_3$) δ 7.96 (s, 1H), 7.84 (d, *J* = 8.9 Hz, 1H), 7.69 (d, *J* = 8.9 Hz, 1H), 7.59 (dd, *J* = 8.9, 2.1 Hz, 1H), 7.54 (t, *J* = 7.9 Hz, 2H), 7.47 (t, *J* = 7.5 Hz, 1H), 7.35 (t, *J* = 7.4 Hz, 1H), 7.22 (d, *J* = 8.9 Hz, 1H), 5.69 (s, 1H), 5.33 (d, *J* = 2.0 Hz, 1H), 2.05 (s, 3H).

^{13}C NMR (100 MHz, $CDCl_3$) δ 154.65, 148.08, 146.86, 141.40, 134.66, 130.58, 130.19, 129.46, 129.42, 129.02, 128.38, 127.79, 121.80, 121.06, 119.28, 118.94, 118.31, 117.71, 113.65, 89.34, 70.22, 16.87.

HRMS (ESI) *m/z* Calcd for $C_{23}H_{14}Br_2NaOS^+$ [M + Na]$^+$: 518.9024, Found: 518.9024.

Optical Rotation: $[\alpha]_D^{25}$ = - 12.5° (*c* = 0.2, acetone).

HPLC analysis: Chiralcel IA-H*2 (Hexane/*i*-PrOH = 95:5, flow rate = 1.0 mL/min, wave length = 254 nm), t_R = 9.543 min (minor), t_R = 10.911 min (major), 90% **ee**

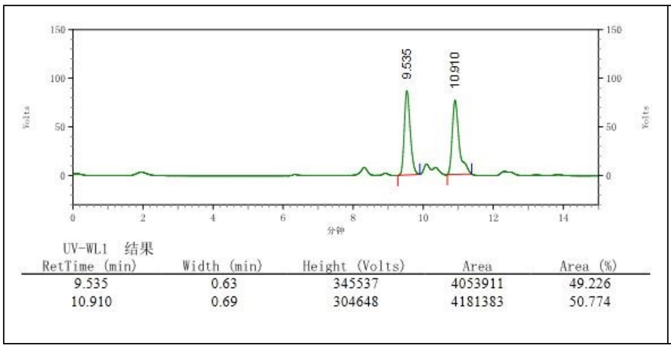

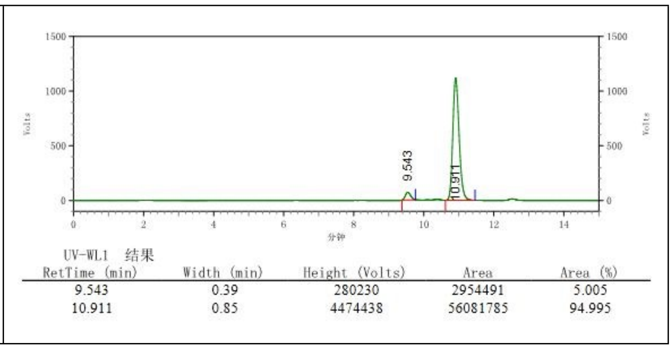

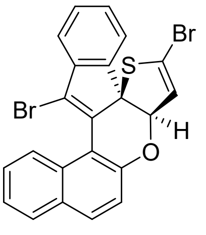

(4b*R*,7a*R*)-6,15-dibromo-7aH-benzo[f]indeno[1,2-c]thieno[3,2-b]chromene (4j)

Compound **4j** was synthesized in 87% yield (125.8 mg, 0.3 mmol scale) under condition [A].

White solid. (R_f = 0.5, PE/EA = 20:1)

^{1}H NMR (400 MHz, $CDCl_3$) δ 7.94 (d, *J* = 8.4 Hz, 1H), 7.82 (t, *J* = 7.7 Hz, 2H), 7.60 (d, *J* = 7.5 Hz, 1H), 7.57 – 7.51 (m, 2H), 7.48 (t, *J* = 7.5 Hz, 1H), 7.42 (t, *J* = 7.5 Hz, 1H), 7.36 (t, *J* = 7.4 Hz, 1H), 7.22 (d, *J* = 9.0 Hz, 1H), 6.12 (d, *J* = 3.2 Hz, 1H), 5.28 (d, *J* = 3.1 Hz, 1H).

^{13}C NMR (100 MHz, $CDCl_3$) δ 153.91, 145.76, 141.52, 133.83, 130.80, 129.69, 129.66, 129.41, 128.37, 127.97, 126.56, 126.38, 124.25, 123.95, 123.73, 121.98, 121.21, 119.75, 118.06, 113.51, 87.70, 72.47.

HRMS (ESI) *m/z* Calcd for $C_{22}H_{12}Br_2NaOS^+$ $[M + Na]^+$: 504.8868, Found: 504.8868.

Optical Rotation: $[\alpha]_D^{25}$ = - 16.0° (*c* = 0.3, acetone).

HPLC analysis: Chiralcel AD-H (Hexane/*i*-PrOH = 95:5, flow rate = 1.0 mL/min, wave length = 254 nm), t_R = 4.649 min (minor), t_R = 5.335 min (major), 88% **ee**

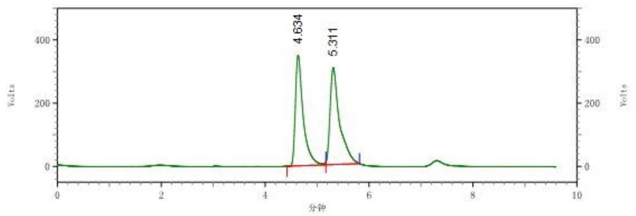

UV-WL1 结果

RetTime (min)	Width (min)	Height (Volts)	Area	Area (%)
4.634	0.75	1398334	14643907	48.367
5.311	0.65	1224490	15632827	51.633

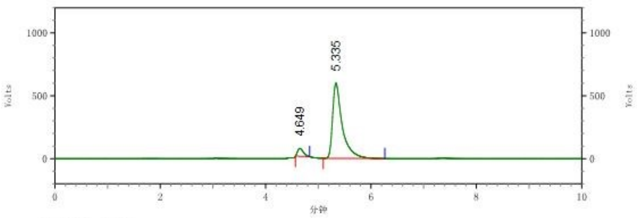

UV-WL1 结果

RetTime (min)	Width (min)	Height (Volts)	Area	Area (%)
4.649	0.27	248697	1933561	5.954
5.335	1.17	2391632	30542912	94.046

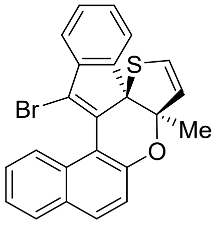

(4b*R*,7a*R*)-15-bromo-7a-methyl-7aH-benzo[f]indeno[1,2-c]thieno[3,2-b]chromene (4k)

Compound **4k** was synthesized in 76% yield (95.6 mg, 0.3 mmol scale) under condition [A].

Yellow oil. (R_f = 0.5, PE/EA = 20:1)

^{1}H NMR (400 MHz, $CDCl_3$) δ 7.94 (d, *J* = 8.4 Hz, 1H), 7.78 (t, *J* = 10.1 Hz, 2H), 7.61 – 7.47 (m, 3H), 7.44 (t, *J* = 7.6 Hz, 1H), 7.38 (t, *J* = 7.6 Hz, 1H), 7.31 (t, *J* = 7.6 Hz, 1H), 7.16 (d, *J* = 8.9 Hz, 1H), 6.65 (dd, *J* = 5.9, 2.6 Hz, 1H), 5.86 (d, *J* = 5.4 Hz, 1H), 1.29 (s, 3H).

^{13}C NMR (100 MHz, $CDCl_3$) δ 156.01, 145.05, 142.19, 135.94, 131.72, 130.58, 129.71, 129.33, 128.83, 128.32, 128.02, 127.30, 126.66, 126.12, 123.80, 123.32, 120.81, 118.41, 117.70, 112.28, 94.38, 72.10, 22.98.

HRMS (ESI) *m/z* Calcd for $C_{23}H_{15}BrNaOS^+$ $[M + Na]^+$: 440.9919, Found: 440.9919.

Optical Rotation: $[\alpha]_D^{25}$ = - 127.0° (*c* = 0.2, acetone).

HPLC analysis: Chiralcel AD-H (Hexane/*i*-PrOH = 95: 5, flow rate = 1.0 mL/min, wave length = 254 nm), t_R = 4.314 min (minor), t_R = 4.843 min (major), 88% **ee**

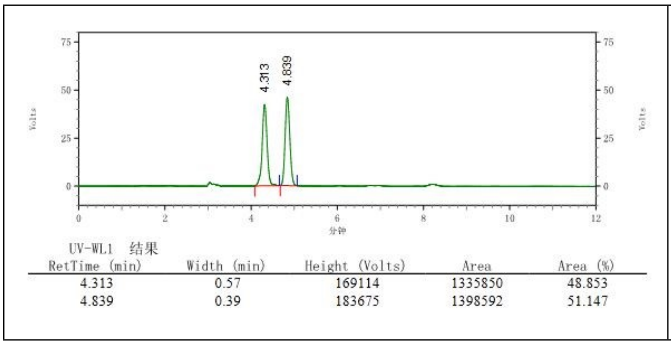

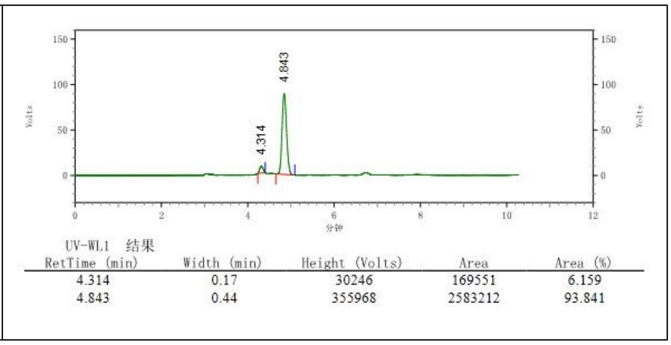

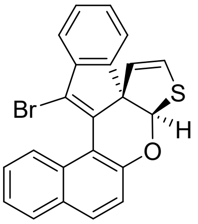

(7a*R*,10a*S*)-15-bromo-7aH-benzo[f]indeno[1,2-c]thieno[2,3-b]chromene (4l)

Compound **4l** was synthesized in 74% yield (89.7 mg, 0.3 mmol scale) under condition [A].

White solid. (R_f = 0.5, PE/EA = 20:1)

^{1}H NMR (400 MHz, $CDCl_3$) δ 8.04 (d, J = 8.4 Hz, 1H), 7.80 (t, J = 9.1 Hz, 2H), 7.59 – 7.51 (m, 3H), 7.48 (t, J = 7.5 Hz, 1H), 7.41 (t, J = 7.5 Hz, 1H), 7.31 (t, J = 7.4 Hz, 1H), 7.27 – 7.22 (m, 1H), 6.40 (d, J = 5.9 Hz, 1H), 6.11 (s, 1H), 5.07 (d, J = 5.9 Hz, 1H).

^{13}C NMR (100 MHz, $CDCl_3$) δ 153.21, 143.17, 142.89, 135.34, 130.54, 129.81, 129.42, 129.00, 128.51, 128.41, 127.38, 126.45, 126.30, 124.27, 123.51, 122.45, 121.01, 119.03, 118.12, 114.17, 91.11, 71.27.

HRMS (ESI) *m/z* Calcd for $C_{22}H_{13}BrNaOS^+$ $[M + Na]^+$: 426.9763, Found: 426.9765.

Optical Rotation: $[\alpha]_D^{25}$ = - 189.0° (c = 0.2, acetone).

HPLC analysis: Chiralcel OD-H (Hexane/i-PrOH = 95:5, flow rate = 1.0 mL/min, wave length = 254 nm), t_R = 5.503 min (minor), t_R = 5.860 min (major), 88% **ee**

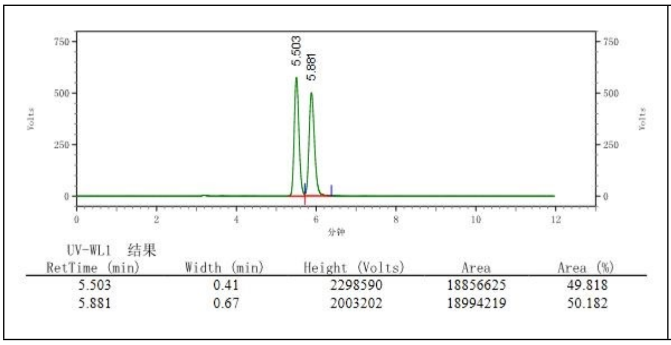

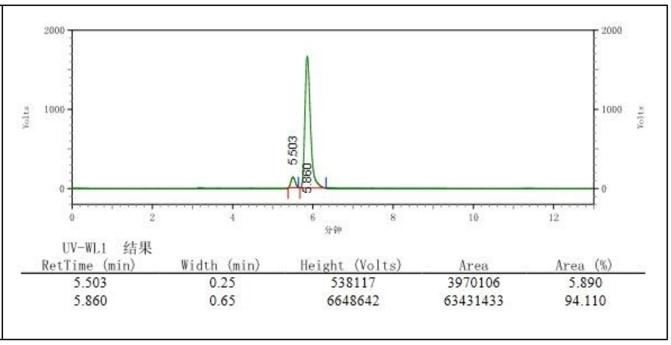

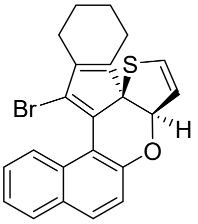

(4b*R*,7a*R*)-15-bromo-1,2,3,4-tetrahydro-7aH-benzo[f]indeno[1,2-c]thieno[3,2-b]chromene (4m)

Compound **4m** was synthesized in 89% yield (108.9 mg, 0.3 mmol scale) under condition [A].

White solid. (R_f = 0.5, PE/EA = 20:1)

^{1}H NMR (400 MHz, $CDCl_3$) δ 7.93 (d, *J* = 8.4 Hz, 1H), 7.77 (d, *J* = 8.1 Hz, 1H), 7.71 (d, *J* = 8.8 Hz, 1H), 7.50 (t, *J* = 7.7 Hz, 1H), 7.36 (t, *J* = 7.5 Hz, 1H), 7.16 (d, *J* = 8.9 Hz, 1H), 6.58 (d, *J* = 5.8 Hz, 1H), 5.90 (dd, *J* = 5.9, 3.0 Hz, 1H), 5.12 (d, *J* = 3.0 Hz, 1H), 2.48 – 2.31 (m, 3H), 2.23 – 2.14 (m, 1H), 1.88 – 1.68 (m, 4H).
^{13}C NMR (100 MHz, $CDCl_3$) δ 153.17, 143.94, 138.59, 134.06, 132.69, 129.50, 129.46, 128.15, 126.89, 125.89, 123.80, 122.66, 121.13, 117.89, 113.80, 85.29, 71.03, 23.57, 22.55, 22.53, 21.20.
HRMS (ESI) *m/z* Calcd for $C_{22}H_{17}BrNaOS^+$ $[M + Na]^+$: 431.0076, Found: 431.0078.

HPLC analysis: Chiralcel IA-H (Hexane/*i*-PrOH = 95:5, flow rate = 1.0 mL/min, wave length = 254 nm), t_R = 3.982 min (minor), t_R = 6.352 min (major), 36% **ee**

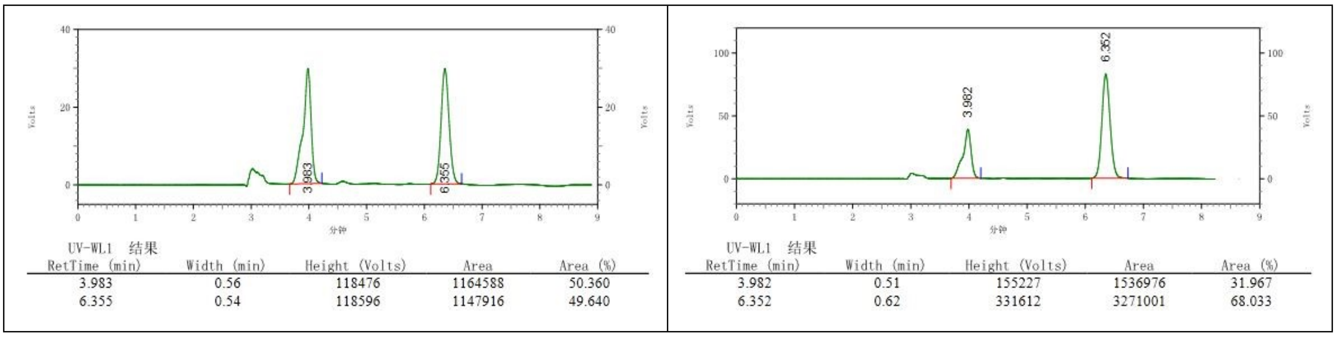

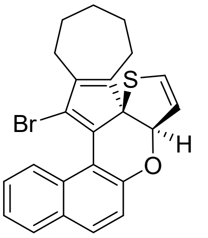

(5b*R*,8a*R*)-16-bromo-2,3,4,5-tetrahydro-1H,8aH-azuleno[1,2-c]benzo[f]thieno[3,2-b]chromene (4n)

Compound **4n** was synthesized in 76% yield (96.2 mg, 0.3 mmol scale) under condition [A].

White solid. (R_f = 0.5, PE/EA = 20:1)

^{1}H NMR (400 MHz, $CDCl_3$) δ 7.90 (d, *J* = 8.4 Hz, 1H), 7.78 (d, *J* = 8.1 Hz, 1H), 7.72 (d, *J* = 8.8 Hz, 1H), 7.50 (t, *J* = 7.6 Hz, 1H), 7.37 (t, *J* = 7.5 Hz, 1H), 7.16 (d, *J* = 8.8 Hz, 1H), 6.52 (d, *J* = 5.8 Hz, 1H), 5.87 (dd, *J* = 5.8, 2.9 Hz, 1H), 5.29 (d, *J* = 2.8 Hz, 1H), 2.70 – 2.56 (m, 2H), 2.53 – 2.45 (m, 1H), 2.41 – 2.32 (m, 1H), 1.91 – 1.65 (m, 6H).
^{13}C NMR (100 MHz, $CDCl_3$) δ 152.34, 147.25, 141.25, 133.81, 132.59, 129.65, 129.45, 129.41, 128.18, 126.80, 125.96, 123.92, 123.31, 123.15, 118.12, 115.46, 85.50, 73.11, 31.08, 27.94, 27.90, 26.77, 26.45.
HRMS (ESI) *m/z* Calcd for $C_{23}H_{19}BrNaOS^+$ $[M + Na]^+$: 445.0232, Found: 445.0233.

HPLC analysis: Chiralcel AD-H (Hexane/*i*-PrOH = 95:5, flow rate = 1.0 mL/min, wave length = 254 nm), t_R = 3.898 min (minor), t_R = 6.227 min (major), 18% **ee**

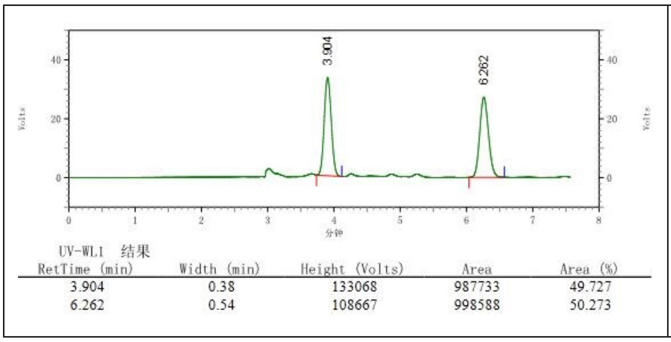

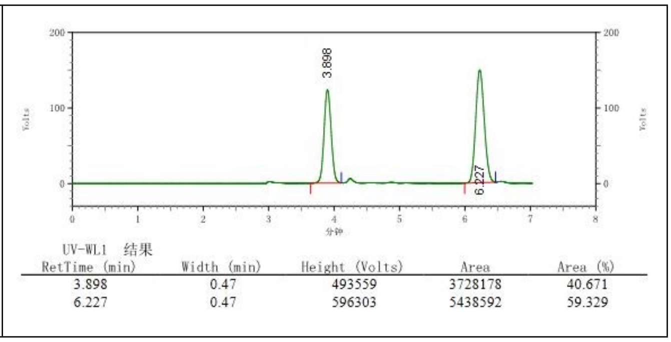

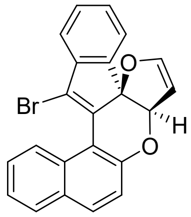

(4b*R*,7a*R*)-15-bromo-7aH-benzo[f]furo[3,2-b]indeno[1,2-c]chromene (4o)

Compound **4o** was synthesized in 90% yield (77.1 mg, 0.2 mmol scale) under condition [A].

White solid. (R_f = 0.4, PE/EA= 15:1)

^{1}H NMR (400 MHz, $CDCl_3$) δ 8.07 (d, *J* = 8.4 Hz, 1H), 7.83 (dd, *J* = 8.2, 5.9 Hz, 2H), 7.57 (t, *J* = 7.6 Hz, 1H), 7.53 – 7.48 (m, 2H), 7.46 – 7.40 (m, 2H), 7.32 (td, *J* = 7.3, 1.8 Hz, 1H), 7.22 (d, *J* = 8.8 Hz, 1H), 6.69 (d, *J* = 2.1 Hz, 1H), 5.63 (d, *J* = 2.1 Hz, 1H), 5.55 (t, *J* = 2.6 Hz, 1H).

^{13}C NMR (100 MHz, $CDCl_3$) δ 152.18, 151.74, 142.51, 142.17, 133.30, 130.66, 129.94, 129.81, 128.33, 127.75, 126.62, 126.41, 124.31, 121.61, 121.31, 121.14, 118.85, 115.09, 104.36, 95.83, 83.45.

HRMS (ESI) *m/z* Calcd for $C_{22}H_{13}BrNaO_2^+$ $[M + Na]^+$: 410.9991, Found: 410.9991.

HPLC analysis: Chiralcel OD-H (Hexane/*i*-PrOH = 95:5, flow rate = 1.0 mL/min, wave length = 254 nm), t_R = 5.499 min (minor), t_R = 6.068 min (major), 66% **ee**

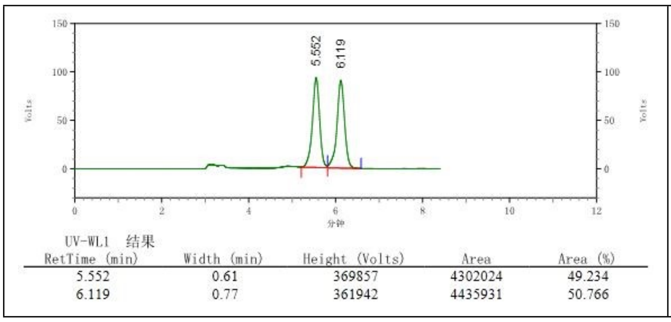

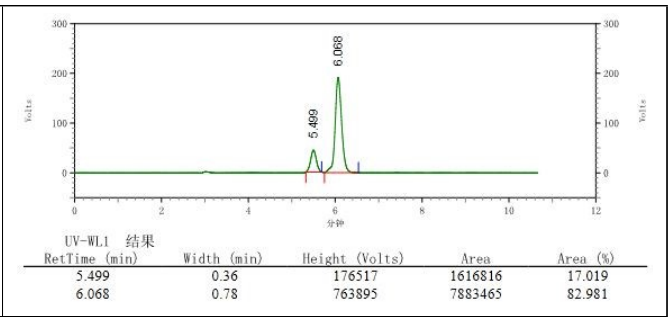

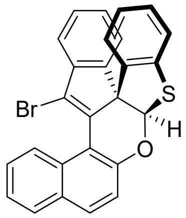

(4b*R*,9a*R*)-17-bromo-9aH-benzo[f]benzo[4,5]thieno[2,3-b]indeno[1,2-c]chromene (4p)

Compound **4p** was synthesized in 69% yield (62.5 mg, 0.2 mmol scale) under condition [A].

White solid. (R_f = 0.5, PE/EA= 15:1)

^{1}H NMR (400 MHz, CDCl$_3$) δ 8.14 (d, *J* = 8.4 Hz, 1H), 7.69 (d, *J* = 8.2 Hz, 1H), 7.63 (d, *J* = 8.9 Hz, 1H), 7.60 – 7.51 (m, 2H), 7.42 (q, *J* = 7.5 Hz, 2H), 7.35 (t, *J* = 7.7 Hz, 1H), 7.20 – 7.15 (m, 2H), 7.11 (d, *J* = 8.9 Hz, 1H), 6.99 (t, *J* = 7.5 Hz, 1H), 6.66 (td, *J* = 16.2, 7.5 Hz, 2H), 6.07 (s, 1H).

^{13}C NMR (100 MHz, CDCl$_3$) δ 153.26, 144.93, 142.08, 139.73, 138.21, 136.08, 130.71, 129.53, 129.29, 128.86, 128.30, 127.40, 126.49, 126.25, 125.60, 124.05, 122.99, 122.47, 121.15, 119.55, 117.62, 112.32, 92.58, 68.99.

HRMS (ESI) *m/z* Calcd for $C_{26}H_{15}BrNaOS^+$ [M + Na]$^+$: 476.9919, Found: 476.9921.

HPLC analysis: Chiralcel OD-H (Hexane/*i*-PrOH = 95:5, flow rate = 1.0 mL/min, wave length = 254 nm), t_R = 6.317 min (minor), t_R = 6.677 min (major), 64% **ee**

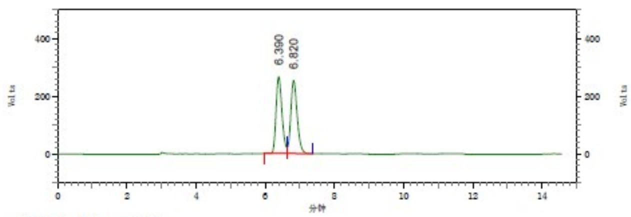

UV-WL1 254 nm 结果

RetTime (min)	Width (min)	Height (Volts)	Area	Area (%)
6.390	0.65	1060519	12594691	49.417
6.820	0.74	1009771	12891855	50.583

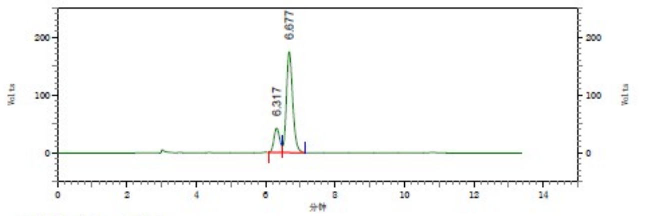

UV-WL1 254 nm 结果

RetTime (min)	Width (min)	Height (Volts)	Area	Area (%)
6.317	0.39	168280	1931814	18.024
6.677	0.67	696491	8786108	81.976

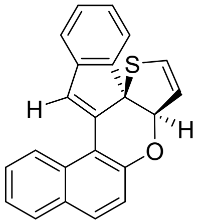

(4b*R*,7a*R*)-7aH-benzo[f]indeno[1,2-c]thieno[3,2-b]chromene (4q)

Compound **4q** was synthesized in 64% yield (42.1 mg, 0.2 mmol scale) under condition [B].

White solid. (R_f = 0.7, PE/EA = 15:1)

^{1}H NMR (400 MHz, CDCl$_3$) δ 8.31 (d, *J* = 8.5 Hz, 1H), 7.77 (d, *J* = 8.1 Hz, 1H), 7.66 (d, *J* = 8.9 Hz, 1H), 7.59 (d, *J* = 7.6 Hz, 1H), 7.52 (t, *J* = 7.6 Hz, 1H), 7.42 (d, *J* = 7.5 Hz, 1H), 7.35 (q, *J* = 7.3 Hz, 2H), 7.30 (s, 1H), 7.21 – 7.14 (m, 2H), 6.91 (d, *J* = 5.9 Hz, 1H), 6.10 (dd, *J* = 5.9, 3.1 Hz, 1H), 5.19 (d, *J* = 3.1 Hz, 1H).

^{13}C NMR (100 MHz, CDCl$_3$) δ 154.54, 146.89, 142.34, 136.50, 136.08, 130.92, 129.51, 129.26, 128.82, 128.60, 128.31, 127.03, 126.12, 123.75, 123.67, 122.55, 121.56, 121.27, 117.91, 111.47, 86.36, 67.11.

HRMS (ESI) *m/z* Calcd for $C_{22}H_{14}NaOS^+$ [M + Na]$^+$: 349.0658, Found: 349.0655.

Optical Rotation: $[\alpha]_D^{25}$ = - 145.0° (*c* = 0.2, acetone).

HPLC analysis: Chiralcel OD-H (Hexane/*i*-PrOH = 90:10, flow rate = 1.0 mL/min, wave length = 254 nm), t_R = 6.983 min (minor), t_R = 8.323 min (major), 98% **ee**

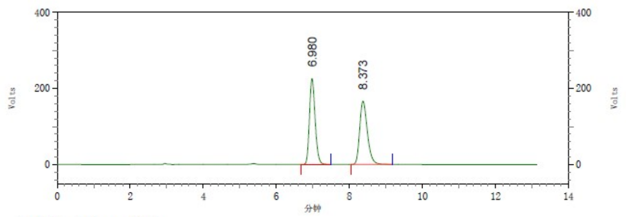

UV-WL1 254 nm 结果

RetTime (min)	Width (min)	Height (Volts)	Area	Area (%)
6.980	0.81	902250	9758146	50.030
8.373	1.13	664654	9746332	49.970

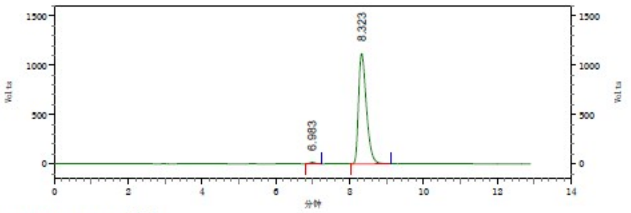

UV-WL1 254 nm 结果

RetTime (min)	Width (min)	Height (Volts)	Area	Area (%)
6.983	0.42	59802	625150	0.936
8.323	1.10	4469991	66162932	99.064

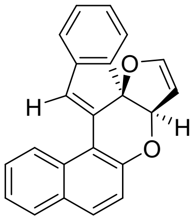

(4b*R*,7a*R*)-7aH-benzo[f]furo[3,2-b]indeno[1,2-c]chromene (4r)

Compound **4r** was synthesized in 95% yield (44.2 mg, 0.15 mmol scale) under condition [B].

White solid. (R_f = 0.5, PE/EA= 15:1)

^{1}H NMR (400 MHz, $CDCl_3$) δ 8.30 (dd, *J* = 8.5, 3.5 Hz, 1H), 7.78 (d, *J* = 8.0 Hz, 1H), 7.70 (dd, *J* = 8.9, 2.7 Hz, 1H), 7.54 – 7.48 (m, 1H), 7.45 – 7.31 (m, 4H), 7.21 – 7.11 (m, 3H), 6.72 (d, *J* = 2.6 Hz, 1H), 5.61 (t, *J* = 2.6 Hz, 1H), 5.55 (t, *J* = 2.9 Hz, 1H).
^{13}C NMR (100 MHz, $CDCl_3$) δ 151.74, 151.63, 143.44, 143.15, 134.91, 131.03, 129.76, 129.62, 129.15, 128.48, 127.02, 126.35, 124.05, 123.79, 121.77, 121.54, 118.88, 114.66, 104.82, 94.40, 82.27.
HRMS (ESI) *m/z* Calcd for $C_{22}H_{14}NaO_2^+$ [M + Na]$^+$: 333.0886, Found: 333.0885.

Optical Rotation: $[\alpha]_D^{25}$ = - 48.6° (*c* = 0.5, acetone).

HPLC analysis: Chiralcel AD-H (Hexane/*i*-PrOH = 95:5, flow rate = 1.0 mL/min, wave length = 254 nm), t_R = 10.623 min (minor), t_R = 18.113 min (major), 98% **ee**

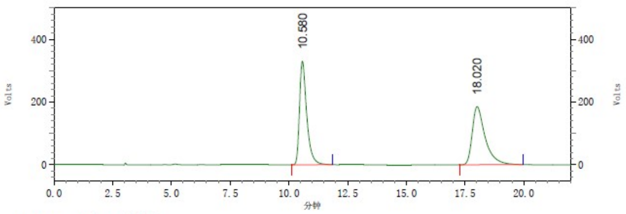

UV-WL1 254 nm 结果

RetTime (min)	Width (min)	Height (Volts)	Area	Area (%)
10.580	1.77	1319422	28255868	49.871
18.020	2.69	741332	28402422	50.129

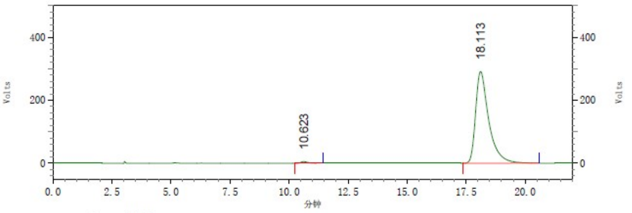

UV-WL1 254 nm 结果

RetTime (min)	Width (min)	Height (Volts)	Area	Area (%)
10.623	1.20	20770	433981	0.954
18.113	3.22	1163259	45037668	99.046

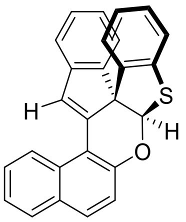

(4b*S*,9a*R*)-9aH-benzo[f]benzo[4,5]thieno[2,3-b]indeno[1,2-c]chromene (4s)

Compound **4s** was synthesized in 77% yield (87.0 mg, 0.3 mmol scale) under condition [B].

White solid. (R_f = 0.5, PE/EA= 15:1)

^{1}H NMR (400 MHz, $CDCl_3$) δ 8.48 (d, *J* = 8.5 Hz, 1H), 7.72 (d, *J* = 8.1 Hz, 1H), 7.64 – 7.52 (m, 3H), 7.47 (s, 1H), 7.45 (d, *J* = 7.5 Hz, 1H), 7.36 (t, *J* = 7.6 Hz, 1H), 7.30 (t, *J* = 7.1 Hz, 2H), 7.15 – 6.96 (m, 3H), 6.73 (t, *J* = 7.5 Hz, 1H), 6.66 (d, *J* = 7.6 Hz, 1H), 6.05 (s, 1H).

^{13}C NMR (100 MHz, $CDCl_3$) δ 152.98, 145.47, 142.74, 139.65, 139.56, 138.15, 130.68, 129.66, 129.53, 129.40, 128.63, 128.54, 127.16, 125.93, 125.64, 123.85, 123.51, 122.82, 122.77, 121.62, 117.81, 111.50, 93.12, 66.72.

HRMS (ESI) *m/z* Calcd for $C_{26}H_{16}NaOS^+$ $[M + Na]^+$: 399.0814, Found: 399.0816.

Optical Rotation: $[\alpha]_D^{25}$ = - 132.8° (*c* = 0.5, acetone).

HPLC analysis: Chiralcel IAIA-H (Hexane/*i*-PrOH = 95:5, flow rate = 1.0 mL/min, wave length = 254 nm), t_R = 13.490 min (minor), t_R = 13.947 min (major), 95% **ee**

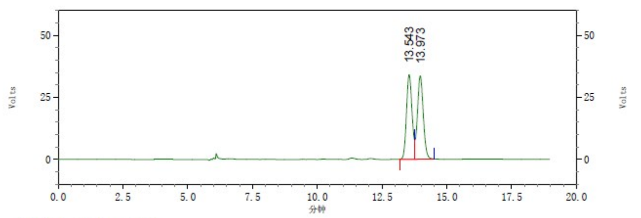

UV-WL1 254 nm 结果

RetTime (min)	Width (min)	Height (Volts)	Area	Area (%)
13.543	0.59	136144	2030338	49.027
13.973	0.76	134269	2110938	50.973

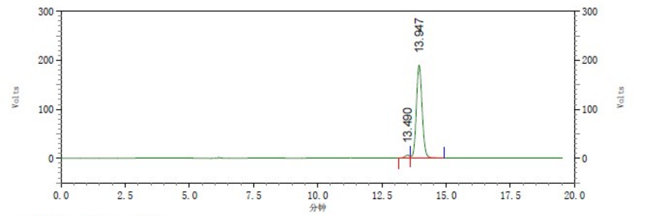

UV-WL1 254 nm 结果

RetTime (min)	Width (min)	Height (Volts)	Area	Area (%)
13.490	0.47	23170	300684	2.482
13.947	1.32	757878	11814841	97.518

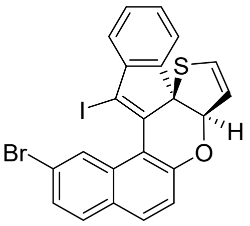

(4b*R*,7a*R*)-13-bromo-15-iodo-7aH-benzo[f]indeno[1,2-c]thieno[3,2-b]chromene (4t)

Compound **4t** was synthesized in 85% yield (127.5 mg, 0.3 mmol scale).

White solid. (R_f = 0.5, PE/EA = 20:1)

^{1}H NMR (400 MHz, $CDCl_3$) δ 8.16 (s, 1H), 7.73 (d, *J* = 8.9 Hz, 1H), 7.67 (d, *J* = 8.7 Hz, 1H), 7.57 – 7.40 (m, 4H), 7.34 (td, *J* = 7.9, 6.5, 3.6 Hz, 1H), 7.21 (d, *J* = 8.9 Hz, 1H), 6.74 (d, *J* = 5.9 Hz, 1H), 5.97 (dd, *J* = 5.9, 2.9 Hz, 1H), 5.43 (d, *J* = 2.9 Hz, 1H).

^{13}C NMR (100 MHz, $CDCl_3$) δ 155.08, 146.43, 144.15, 140.99, 134.95, 130.35, 130.10, 129.96, 129.48, 129.15, 128.01, 127.85, 127.43, 123.36, 121.93, 121.67, 120.88, 118.57, 114.51, 95.63, 88.65, 70.85.

HRMS (ESI) *m/z* Calcd for $C_{22}H_{12}BrINaOS^+$ $[M + Na]^+$: 552.8729, Found: 552.8729.

HPLC analysis: Chiralcel OD-H (Hexane/*i*-PrOH = 93:7, flow rate = 1.0 mL/min, wave length = 254 nm), t_R = 5.673 min (minor), t_R = 6.113 min (major), 27% **ee**

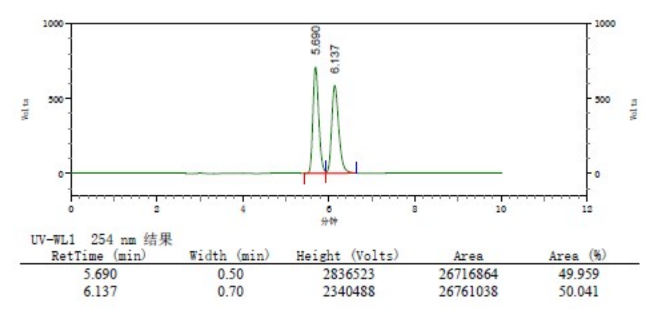

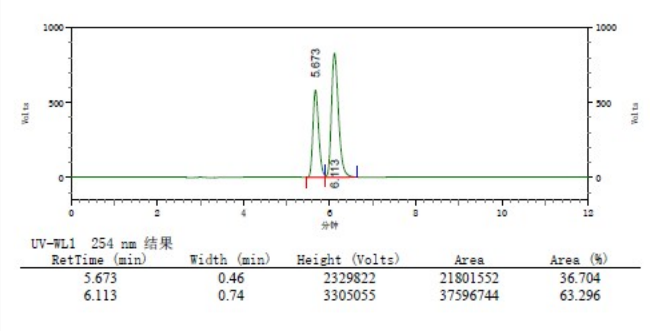

IX. Rotational barriers of 2l, 4l

2l used for racemization experiment was obtained by a 1-mmol-scale reaction in 92.16% ee, 82% yield (337.0 mg, 1.0 mmol scale) under condition [A].

The enantiomerisation barrier of **2l**, corresponding to the barrier to rotation for the following atropisomers, was obtained by kinetic of racemization of an enantiomer. The slope of the first order kinetic line gives the racemization constant ($K_{racemization}$ = 2 × $K_{enantiomerisation}$). Eyring equation gives the enantiomerization barrier ($\Delta G^{\neq}_{enaniomerization}$) from enantiomerisation constant ($K_{enantiomerization}$), R = 8.31451 J•K^{-1}mol^{-1}, h = 6.62608 × 10^{-34} Js and k_B = 1.38066 × 10^{-23} J•K^{-1}. Reactions were conducted at 20 mg/mL concentration. Enantiomeric excess values were determined by HPLC.

$$\Delta G^{\neq}_{enaniomerization} = \mathrm{RT} \times ln \frac{k_B \times \mathrm{T}}{hK_{enantiomerisation}}$$

$$t_{1/2} = ln2 / K_{racemization}$$

Racemization of 2l in xylene at 120 °C.

Times (s)	ee (%)	ln(ee$_0$/ee$_t$)
0	92.16	0
14400	92.15	0.000108513
43200	92.08	0.000868433
86400	92.04	0.001302932
172800	91.9	0.002825168
259200	91.7	0.005003818
432000	91.48	0.007405828

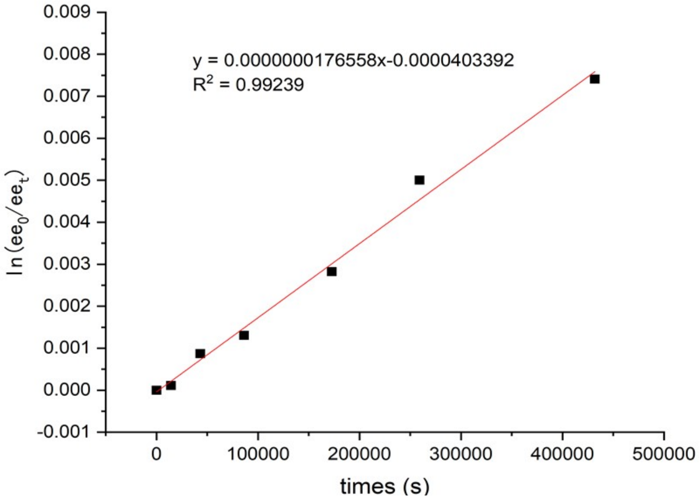

$K_{racemization}$ (120 °C) = 1.8×10^{-8} s^{-1}; $t_{1/2}$ = 10905 h

$K_{enantiomerization}$ (120 °C) = 8.8×10^{-9} s-1; $\Delta G^{\neq}_{enantiomerization}$ = 37.7 kcal/mol

Product **4l** decomposed after react at 100 °C for 24h, so the rotational barriers of **4l** was only carried out by DFT calculation.

The DFT calculation focuses on the axis of **4l**, the racemization of central chirality was not concerned.

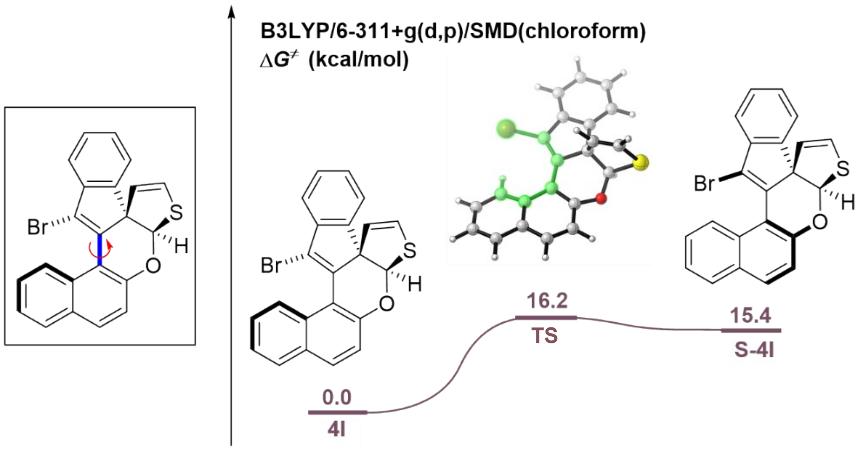

Geometry	$E_{(elec\text{-}B3\text{-}LYP\text{-}D3)}$[1]	$G_{(corr\text{-}B3\text{-}LYP\text{-}D3)}$[2]	$H_{(corr\text{-}B3\text{-}LYP\text{-}D3)}$[3]	$E_{(solv\text{-}M06\text{-}2X)}$[4]	IF[5]
**4l**	-3891.017	0.236304	0.303562	-3893.620944	
**TS**	-3890.991	0.238359	0.302253	-3893.593855	31.6*i*
**S-4l**	-3890.992	0.236181	0.302945	-3893.595772	

[1]The electronic energy calculated by B3-LYP-D3 in chloroform solvent. [2]The thermal correction to Gibbs free energy calculated by B3-LYP-D3 in chloroform solvent. [3]The thermal correction to enthalpy calculated by B3-LYP-D3 in chloroform solvent. [4]The electronic energy calculated by M06-2X in chloroform solvent. [5]The B3-LYP-D3 calculated imaginary frequencies for the transition states.

Cartesian coordinates of the structures

4l

Br	0.15045800	-2.91818300	-0.67725100
S	-1.60678000	3.64433900	0.03158700
O	-0.03792200	2.11881800	-1.55878800
C	-2.18120300	-1.27654900	0.02488500
C	-2.53520300	0.06324800	0.27595200
C	3.60127300	0.66503600	-0.12288700
C	1.14575800	0.46976900	-0.26026200
C	-3.85679400	0.42197900	0.49858200
H	-4.12334500	1.45427300	0.70877800
C	1.13007000	1.63186300	-1.02817200
C	2.39229000	-0.01078000	0.25366200
C	-0.72969800	-1.32137300	-0.16476300
C	-1.27847900	0.91625500	0.28464700
C	-1.25493800	2.02515200	-0.82322600
H	-2.02774300	1.86254500	-1.57340000
C	2.31811100	2.29853400	-1.39729000
H	2.24616300	3.18107400	-2.02448900
C	-4.48716000	-1.90959100	0.18563700
H	-5.26137500	-2.67128900	0.15083900
C	-4.83786500	-0.57885000	0.44449300
H	-5.88005500	-0.31866400	0.60751100
C	4.84465800	0.18821700	0.37327200
H	5.75497800	0.70157100	0.07242800
C	-1.08673700	1.60530600	1.62161900
H	-0.86775400	1.01980000	2.50807500
C	2.48067500	-1.09813400	1.16327700
H	1.57008000	-1.58078900	1.49861400
C	-3.15290700	-2.27469100	-0.02543700
H	-2.87884100	-3.30683300	-0.22277900

C	3.52821900	1.80891900	-0.96506300
H	4.45006800	2.31087000	-1.24742400
C	-0.17277100	-0.09569800	-0.02275600
C	4.89899900	-0.88962400	1.22948400
H	5.85509300	-1.24319500	1.60554800
C	-1.24476000	2.92948800	1.61446700
H	-1.18178300	3.58591800	2.47650300
C	3.70235000	-1.52823500	1.63572300
H	3.74700900	-2.36029500	2.33345400

4l-TS

Br	0.33697800	-3.00345800	0.30240000
S	-2.44979700	3.32228500	0.02526500
O	-0.05130500	2.71934800	-0.83061100
C	-1.99802000	-1.42315100	-0.13559400
C	-2.50504500	-0.11660000	-0.19505600
C	3.62845800	1.07324200	0.10266100
C	1.18246500	0.67434700	-0.24978300
C	-3.87055900	0.11476700	-0.31234800
H	-4.26572600	1.12429500	-0.33751100
C	1.06753500	2.06723300	-0.41252700
C	2.54733100	0.16741700	-0.17486900
C	-0.53830900	-1.34399000	-0.04760900
C	-1.34375300	0.83617600	0.02209000
C	-1.33405700	2.10517300	-0.82630100
H	-1.66486300	1.95249900	-1.85641900
C	2.14667800	2.95828100	-0.19053400
H	1.94364600	4.02141200	-0.26522100
C	-4.22763800	-2.29234600	-0.33131100
H	-4.90932500	-3.13649900	-0.38982600
C	-4.73107400	-0.98795500	-0.39671800
H	-5.79944000	-0.82577700	-0.50906200
C	4.95107000	0.58209600	0.24935500
H	5.73887600	1.29146300	0.49218500
C	-1.46634300	1.34681800	1.46717200
H	-1.17822900	0.71609300	2.30065000
C	2.91046600	-1.16716900	-0.47476200
H	2.16293000	-1.84250100	-0.85351100
C	-2.85686900	-2.52416700	-0.18999500
H	-2.46933700	-3.53600500	-0.13339200
C	3.38247200	2.47376200	0.13343200
H	4.20272500	3.15259500	0.35063900
C	-0.08071100	-0.05304500	-0.15289000
C	5.24516200	-0.74880600	0.04254300
H	6.26358800	-1.11313700	0.14440600
C	-2.04757000	2.54251300	1.57730000
H	-2.30121100	3.05309800	2.50023900
C	4.21307200	-1.61757200	-0.36566000
H	4.44075800	-2.64998200	-0.61808700

S-4l

Br	0.55513900	-2.81016300	0.71913900
S	-2.70951700	3.17356000	-0.05275900
O	-0.15189000	2.83726500	-0.30941800
C	-1.86387400	-1.51024700	-0.18254100
C	-2.44861700	-0.24651300	-0.39546200
C	3.61007500	1.11856900	0.00220200
C	1.14326800	0.76925600	-0.11215800
C	-3.78868800	-0.13430500	-0.73974800
H	-4.24974000	0.83713100	-0.88156900
C	1.01579600	2.16769600	-0.11565500
C	2.49482500	0.25350100	-0.25699100
C	-0.44383000	-1.31043800	0.10779700
C	-1.41350300	0.79546500	0.01019000
C	-1.33899100	2.13531100	-0.71288400
H	-1.38283300	2.06565700	-1.80354300
C	2.12060700	3.02608900	0.10602500
H	1.92762100	4.09156000	0.17783600
C	-3.96158100	-2.56321400	-0.68147700
H	-4.56064600	-3.46165000	-0.80276000
C	-4.53873100	-1.30882100	-0.90524600
H	-5.58078800	-1.23952000	-1.20488400
C	4.93225000	0.61139900	-0.08089000
H	5.75812500	1.27968100	0.15159000
C	-1.81960900	1.19610800	1.44443500
H	-1.62411600	0.53278600	2.27935100
C	2.78620800	-1.03362200	-0.77071800
H	1.97991800	-1.65159100	-1.13584800
C	-2.61898000	-2.67677400	-0.30532900
H	-2.16991200	-3.64875000	-0.12701600
C	3.37937100	2.50401000	0.23718700
H	4.22536200	3.15385400	0.44485100
C	-0.08524900	0.00732200	-0.02294000
C	5.17239400	-0.68167100	-0.49649900
H	6.18918600	-1.05711900	-0.57092300
C	-2.52700300	2.32510400	1.50950000
H	-2.99048100	2.74968300	2.39337200
C	4.08532000	-1.49243500	-0.88460300
H	4.26709200	-2.48184000	-1.29599500

X. Pharmacological research

Cell Culture

Six cancer cell lines A375, A549, HCT116, Hela, HepG2, MCF-7 and human hepatocyte cell line L02 were used in this work. All cell lines were grown in Dulbecco's modified Eagle's medium (DMEM, Hyclone), except for A375, which was maintained in DMEM 12430 (Invitrogen) with sodium pyruvate (Invitrogen) added. All media were supplemented with 10% foetal bovine serum (FBS, BI), 100 units/mL of penicillin–streptomycin (Sigma-Aldrich), incubated at 37 °C in a humidified atmosphere containing 5% CO_2.

Anti-proliferation assay

The anti-proliferative activities of the prepared compounds against the mentioned cell lines were evaluated using a standard GI_{50} assay. Cell lines were grown to log phase in DMEM supplemented with 10% foetal bovine serum. Cell suspensions were prepared and 100 µL/well dispensed into 96-well plates to give 2 x 10^3 cells/well. After incubation for 24 h, cells were treated with the target compounds at 0.39, 0.78, 1.56, 3.125, 6.25, 12.5, 25, and 50 µM and incubated for 48 h at 37 °C in a humidified atmosphere of 5% CO_2. Afterwards, cell viability was assessed by the conventional 3-(4, 5-dimethylthiazol-2-yl)-2, 5-diphenyltetrazolium bromide (MTT) reduction assay carried out strictly according to the manufacturer's instructions (Sigma). The absorbance (OD_{570}) was read on a Spectramax microplate reader (Molecular Devices, US). In all experiments, three replicate wells were used for each drug concentration. Each assay was performed at least three times.

Table 3. IC_{50} values of selected compounds toward cancer cells. Data are presented as the mean ± SD from at least three independent experiments.

	A375	A549	HCT116	Hela	HepG2	MCF-7	L02
**2a**	3.89±0.77	15.45±2.12	11.21±1.71	6.8±1.18	9.9±1.25	8.77±1.33	>50
**2j**	1.28±0.26	1.17±0.32	1.04±0.27	1.45±0.44	2.2±0.58	1.2±0.33	>50
**2l**	2.02±0.43	7.06±0.95	2.15±0.25	5.34±0.48	4.76±0.47	4.03±0.30	>50
**2o**	9.12±1.86	30.03±2.89	20.12±2.31	16.69±2.57	35.55±4.11	25.93±3.04	>50
**4a**	>50	>50	>50	>50	>50	>50	>50
**4i**	>50	>50	>50	>50	>50	>50	>50
**4l**	40.59±5.02	>50	>50	>50	38.27±3.93	>50	>50

XI. Gram-scale preparation and transformations

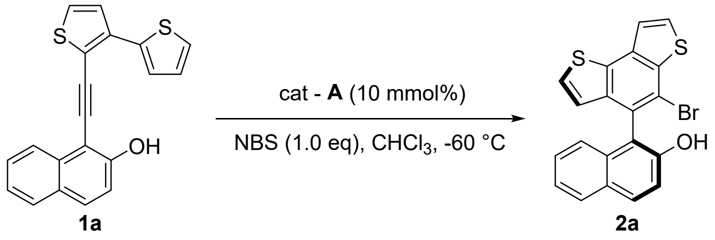

1a 3.4 g

2a 3.1 g 74%, 97% ee

A solution of **1a** (3.4 g, 10.2 mmol, 1.0 equiv.) and catalyst-**A** (505 mg, 1.02 mmol, 10 mol%) in $CHCl_3$ (200 mL) was stirred at -60 °C for 30 min, then NBS (1.82 g, 10.2 mmol, 1.0 equiv.) was added in 10 portions. After stirring at -60 °C for 12 h, the reaction mixture was concentrated under reduced pressure. The resulting residue was purified by silica gel flash column chromatography using PE/EA eluent (10:1 to 4:1) to afford the product **2a** (3.1 g, 7.54 mmol, 74% yield) as a yellow solid.

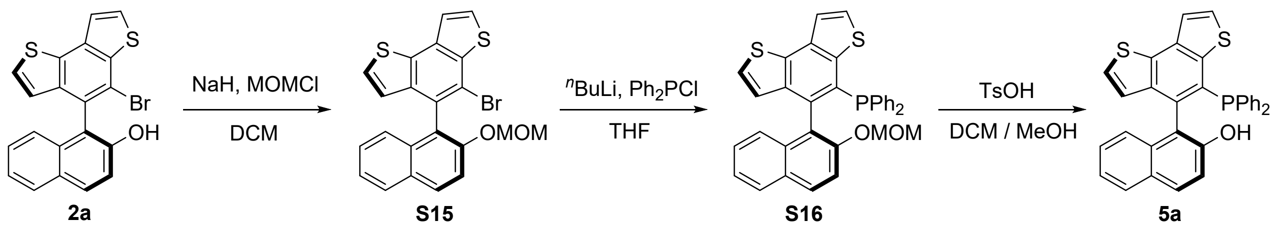

2a obtained in the Gram-scale reaction was further purified by recrystallization (DCM/hexane) to afford **2a** with higher enantiomeric excess as a white solid (2.8 g, >99% ee).

2a (2.06 g, 5 mmol, 1.0 equiv.) and NaH (240 mg, 10 mmol, 2.0 equiv.) was dissolved in DCM (20 mL) at room temperature under nitrogen atmosphere. MOMCl (0.45 ml, 6 mmol, 1.2 equiv.) was added dropwise. After stirred for 6 h at rt, the reaction was quenched with saturated aqueous NH_4Cl, extracted with DCM, washed with brine, dried over Na_2SO_4 and filtered, concentrated under reduced pressure and purified by column chromatography on silica gel (PE/EA = 10:1) to afford the desired product **S15** (1.82 g, 4.0 mmol, 80% yield) as a white solid. **^{1}H NMR** (400 MHz, $CDCl_3$) δ 7.99 (d, *J* = 9.0 Hz, 1H), 7.89 (d, *J* = 7.9 Hz, 1H), 7.76 (d, *J* = 4.9 Hz, 1H), 7.68 (d, *J* = 4.6 Hz, 1H), 7.60 (d, *J* = 9.0 Hz, 1H), 7.38 (t, *J* = 7.2 Hz, 1H), 7.30 – 7.23 (m, 2H), 7.13 (d, *J* = 8.2 Hz, 1H), 6.72 (d, *J* = 5.1 Hz, 1H), 5.17 – 5.03 (m, 2H), 3.24 (s, 3H). **^{13}C NMR** (100 MHz, $CDCl_3$) δ 152.20, 138.91, 138.82, 133.31, 133.21, 132.93, 130.17, 129.53, 129.27, 127.98, 126.77, 124.92, 124.70, 124.18, 123.41, 122.74, 116.39, 115.12, 94.79, 56.00.
HRMS (ESI) m/z Calcd for $C_{22}H_{15}BrNaO_2S_2^+$ $[M + Na]^+$: 476.9589, Found: 476.9589.

To an oven dried flask, **S15** (1.82 g, 4.0 mmol, 1.0 equiv.) was dissolved by dried THF under nitrogen atmosphere. The solvent was cooled to -78ºC and *n*-BuLi (1.6 M solution in hexanes, 3.0 ml, 4.8 mmol, 1.2 equiv) was added dropwise. The reaction mixture was held at -78 ºC for 2 hours before Ph_2PCl (1.1 ml, 6.0 mmol, 1.5 equiv.) in dried THF was added slowly. The resulting solution was stirred at -78 ºC for about 30 min, then allowed to warm to rt. After stirred overnight at rt, the mixture was quenched with water, extracted with EA, dried over anhydrous Na_2SO_4, and concentrated under reduced pressure. The crude product was purified by column chromatography (PE/EA = 8:1) to afford product **S16** (1.5 g, 2.68 mmol, 67% yield) as a white solid. **^{1}H NMR** (400 MHz, $CDCl_3$) δ 7.99 (d, *J* = 9.0 Hz, 1H), 7.89 (d, *J* = 8.0 Hz, 1H), 7.67 – 7.55 (m, 2H), 7.49 – 7.09 (m, 15H), 6.72 (d, *J* = 5.3 Hz, 1H), 4.91 (d, *J* = 7.0 Hz, 1H), 4.66 (d, *J* = 6.9 Hz, 1H), 3.08 (s, 3H). **^{13}C NMR** (100 MHz, $CDCl_3$) δ 152.82, 141.13, 141.10, 138.21, 138.18, 138.12, 137.77, 136.36, 135.62, 135.48, 134.84, 134.70, 133.90, 133.73, 133.13, 132.94, 132.89, 132.70, 129.73, 129.17, 128.67, 128.01, 127.95, 127.85, 126.31, 126.18, 125.38, 125.31, 124.30, 124.19, 123.80, 123.76, 120.42, 115.62, 94.40, 55.36.
HRMS (ESI) m/z Calcd for $C_{34}H_{25}BrNaO_2S_2^+$ $[M + Na]^+$: 583.0926, Found: 583.0928.

S16 (1.5 g, 2.68 mmol, 1.0 equiv.) and TsOH (3.69 g, 21.4 mmol, 8.0 equiv.) were weighed and added into an oven dried schlenk tube, evacuated and backfilled with nitrogen (3 times). DCM (10 mL) and MeOH (2 mL) was injected into the flask. Then, the resulting solution was kept stirring for 8 hours. Quenched with aq. Na_2CO_3, extracted with DCM, washed with brine, dried over Na_2SO_4 and filtered, concentrated under reduced pressure and purified by column chromatography on silica gel (PE/EA = 4:1) to afford the desired product **5a** (996 mg, 1.93 mmol, 72% yield) as a white solid. **^{1}H NMR** (400 MHz, $CDCl_3$) δ 7.87 (d, *J* = 8.9 Hz, 1H), 7.80 (d, *J* = 8.1 Hz, 1H), 7.60 (d, *J* = 5.5 Hz, 1H), 7.49 (d, *J* = 5.5 Hz, 1H), 7.36 (t, *J* = 6.9 Hz, 2H), 7.23 (ddq, *J* = 33.6, 20.1, 7.0 Hz, 11H), 7.07 (t, *J* = 7.6 Hz, 1H), 6.95 (d, *J* = 8.4 Hz, 1H), 6.70 (d, *J* = 5.4 Hz, 1H), 4.73 (s, 1H). **^{13}C NMR** (100 MHz, $CDCl_3$) δ 150.91, 141.18, 138.44, 138.36, 137.03, 134.98, 134.86,

134.73, 134.59, 134.28, 133.88, 133.67, 133.44, 133.25, 133.06, 130.15, 129.72, 128.96, 128.75, 128.63, 128.48, 128.41, 128.33, 128.17, 128.11, 127.96, 126.39, 124.96, 124.94, 124.82, 123.28, 120.47, 119.93, 119.82, 117.29.

HRMS (ESI) m/z Calcd for $C_{32}H_{21}NaOPS_2^+$ [M + Na]$^+$: 539.0664, Found: 539.0664.

HPLC analysis: Chiralcel IB-H (Hexane/*i*-PrOH = 90:10, flow rate = 1.0 mL/min, wave length = 254 nm), t_R = 8.173 min (major), t_R = 9.090 min (minor), >99% **ee**

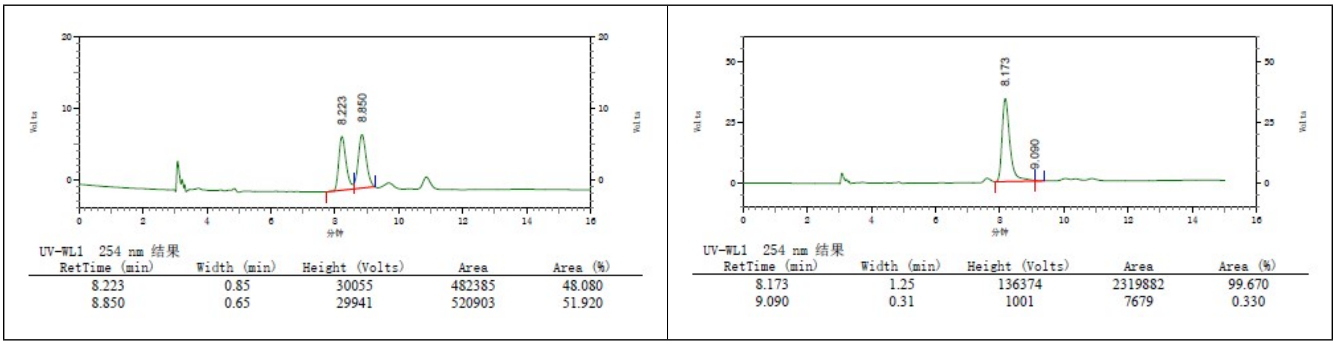

RetTime (min)	Width (min)	Height (Volts)	Area	Area (%)
8.223	0.85	30055	482385	48.080
8.850	0.65	29941	520903	51.920

RetTime (min)	Width (min)	Height (Volts)	Area	Area (%)
8.173	1.25	136374	2319882	99.670
9.090	0.31	1001	7679	0.330

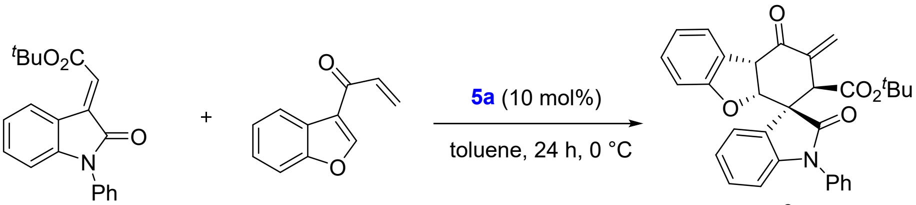

6a

59%, 93% ee, >20:1 dr

Reaction condition: A solution of **S17** (0.15 mmol, 1.5 equiv.), **S18** (0.1 mmol, 1.0 equiv.) and **5a** (0.01 mmol, 10 mol%) in toluene (1.0 mL) was stirred at 0 ºC for 24 h under nitrogen atmosphere. The reaction mixture was concentrated under reduced pressure. The resulting residue was purified by silica gel flash column chromatography using PE/EA eluent to afford the product **6a**.

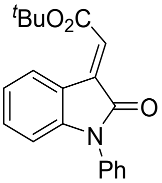

tert-butyl (*E*)-2-(2-oxo-1-phenylindolin-3-ylidene)acetate (S17)

Compound **S17** was synthesized following reference **7**

Orange solid. (R_f = 0.5, PE/EA = 8:1)

^{1}H NMR (400 MHz, $CDCl_3$) δ 8.62 (d, *J* = 7.7 Hz, 1H), 7.53 (t, *J* = 7.6 Hz, 2H), 7.40 (d, *J* = 8.1 Hz, 3H), 7.29 (d, *J* = 7.7 Hz, 1H), 7.10 (t, *J* = 7.7 Hz, 1H), 6.95 (s, 1H), 6.78 (d, *J* = 7.9 Hz, 1H), 1.59 (s, 9H).

^{13}C NMR (100 MHz, $CDCl_3$) δ 167.13, 164.90, 145.65, 136.47, 133.97, 131.95, 129.66, 128.72, 128.22, 126.57, 125.40, 123.17, 119.93, 109.36, 81.97, 28.09.

HRMS (ESI) *m/z* Calcd for $C_{20}H_{19}NNaO_3^+$ [M + Na]$^+$: 344.1257, Found: 344.1257.

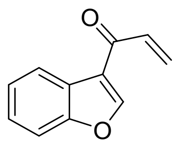

1-(benzofuran-3-yl)prop-2-en-1-one (S18)

Compound **S18** was synthesized following reference **7**

White solid. (R_f = 0.4, PE/EA = 8:1)

^{1}H NMR (400 MHz, $CDCl_3$) δ 8.42 – 8.12 (m, 2H), 7.60 – 7.46 (m, 1H), 7.44 – 7.30 (m, 2H), 6.93 (dd, *J* = 17.0, 10.5 Hz, 1H), 6.49 (dd, *J* = 17.1, 1.6 Hz, 1H), 5.86 (dd, *J* = 10.4, 1.6 Hz, 1H).
^{13}C NMR (100 MHz, $CDCl_3$) δ 185.16, 155.62, 151.03, 133.29, 128.58, 125.83, 124.55, 124.47, 123.02, 122.30, 111.42.
HRMS (ESI) *m/z* Calcd for $C_{11}H_8NaO_2^+$ $[M + Na]^+$: 195.0417, Found: 195.0416.

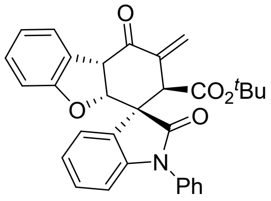

tert-butyl (3R,4S,4aR,9bS)-2-methylene-1,2'-dioxo-1'-phenyl-2,3,4a,9b-tetrahydro-1H-spiro[dibenzo[b,d]furan-4,3'-indoline]-3-carboxylate (6a)

White solid. (R_f = 0.5, PE/EA = 4:1)

^{1}H NMR (400 MHz, $CDCl_3$) δ 7.68 (d, *J* = 7.5 Hz, 1H), 7.54 – 7.48 (m, 4H), 7.38 – 7.30 (m, 3H), 7.24 – 7.17 (m, 2H), 6.99 – 6.91 (m, 3H), 6.46 (d, *J* = 3.1 Hz, 1H), 5.36 (d, *J* = 2.9 Hz, 1H), 5.09 (d, *J* = 9.4 Hz, 1H), 4.52 (d, *J* = 9.3 Hz, 1H), 4.46 (d, *J* = 3.3 Hz, 1H), 1.11 (s, 9H).
^{13}C NMR (100 MHz, $CDCl_3$) δ 190.47, 174.35, 168.09, 159.02, 143.83, 136.90, 133.78, 129.76, 129.44, 129.20, 128.03, 127.50, 126.50, 126.09, 125.79, 124.27, 124.19, 123.10, 121.76, 109.69, 83.33, 82.26, 51.72, 50.78, 50.08, 27.38.
HRMS (ESI) *m/z* Calcd for $C_{31}H_{27}NNaO_5^+$ $[M + Na]^+$: 516.1781, Found: 516.1783.

Optical Rotation: $[\alpha]_D^{25}$ = 21.5° (*c* = 0.2, $CHCl_3$).

HPLC analysis: Chiralcel AD-H (Hexane/*i*-PrOH = 80:20, flow rate = 1.0 mL/min, wave length = 254 nm), t_R = 6.173 min (major), t_R = 6.908 min (minor), 93% **ee**

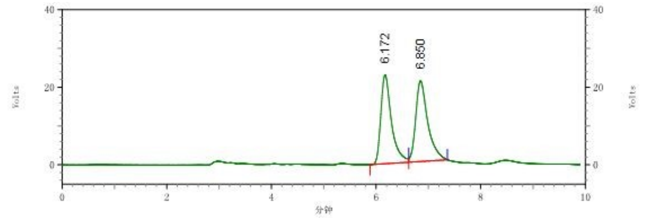

UV-WL1 结果

RetTime (min)	Width (min)	Height (Volts)	Area	Area (%)
6.172	0.74	91737	1298716	49.254
6.850	0.74	83036	1338078	50.746

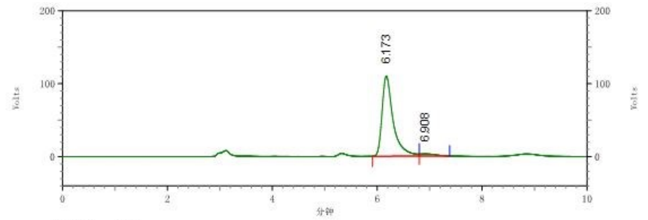

UV-WL1 结果

RetTime (min)	Width (min)	Height (Volts)	Area	Area (%)
6.173	0.89	440039	6486713	96.400
6.908	0.58	11924	242237	3.600

Product **5a** is a reported compound, the absolute configuration of chiral product **6a** was assigned according to reference **7**.

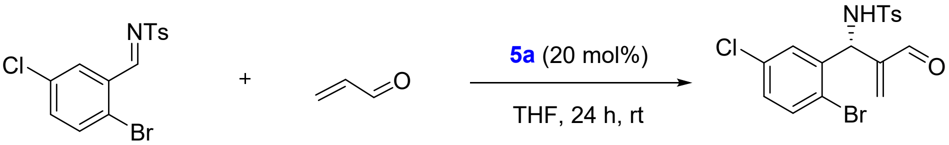

6b
73%, 95% ee

Reaction condition: To **S19** or **S20** (0.2 mmol, 1.0 equiv.) and **5a** (0.04 mmol, 20 mol%) in THF (2.0 ml) was added acrylaldehyde (27 μl, 0.8 mmol, 4.0 equiv.) stirred at rt for 24 h under nitrogen atmosphere. The reaction mixture was concentrated under reduced pressure. The resulting residue was purified by silica gel flash column chromatography using PE/EA eluent to afford the product **6b** (62.2 mg, 73% yield) or **6c** (59.2 mg, 72% yield).

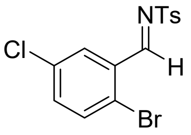

(*E*)-N-(2-bromo-5-chlorobenzylidene)-4-methylbenzenesulfonamide (S19)

Compound **S19** was synthesized following reference **8**

White solid. (R_f = 0.6, PE/EA = 5:1)

^{1}H NMR (400 MHz, $CDCl_3$) δ 9.37 (s, 1H), 8.19 (dd, *J* = 8.8, 6.1 Hz, 1H), 7.90 (d, *J* = 8.3 Hz, 2H), 7.41 (dd, *J* = 8.0, 2.5 Hz, 1H), 7.37 (d, *J* = 8.0 Hz, 2H), 7.14 – 7.08 (m, 1H), 2.45 (s, 3H).
^{13}C NMR (100 MHz, $CDCl_3$) δ 167.74, 145.10, 135.35, 134.80, 134.39, 134.16, 132.35, 130.03, 129.91, 128.31, 126.31, 77.00, 21.65.
HRMS (ESI) *m/z* Calcd for $C_{14}H_{11}BrClNNaO_2S^+$ $[M + Na]^+$: 393.9275, Found: 393.9272.

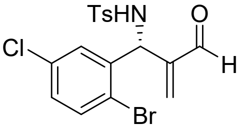

(*R*)-N-(1-(2-bromo-5-chlorophenyl)-2-formylallyl)-4-methylbenzenesulfonamide (5b)
White solid. (R_f = 0.6, PE/EA = 5:1)

^{1}H NMR (400 MHz, $CDCl_3$) δ 9.38 (s, 1H), 7.63 (d, *J* = 7.9 Hz, 2H), 7.35 – 7.30 (m, 1H), 7.24 – 7.12 (m, 3H), 6.85 (t, *J* = 7.1 Hz, 1H), 6.49 (s, 1H), 6.15 (s, 1H), 6.00 (d, *J* = 8.2 Hz, 1H), 5.69 (d, *J* = 8.2 Hz, 1H), 2.38 (s, 3H).
^{13}C NMR (100 MHz, $CDCl_3$) δ 192.74, 145.87, 143.71, 138.34, 137.77, 136.78, 134.09, 133.74, 129.50, 129.36, 127.12, 120.70, 77.00, 56.19, 21.48.
HRMS (ESI) *m/z* Calcd for $C_{17}H_{15}BrClNNaO_3S^+$ $[M + Na]^+$: 449.9537, Found: 449.9539.

Optical Rotation: $[\alpha]_D^{25}$ = - 62.7° (*c* = 0.3, $CHCl_3$).

HPLC analysis: Chiralcel AD-H (Hexane/*i*-PrOH = 80:20, flow rate = 1.0 mL/min, wave length = 254 nm), t_R = 10.440 min (minor), t_R = 12.420 min (major), 95% **ee**

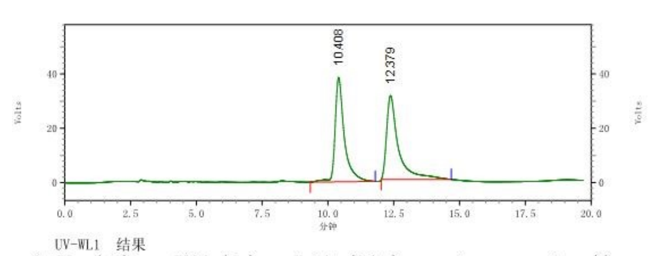

RetTime (min)	Width (min)	Height (Volts)	Area	Area (%)
10.408	2.47	153733	3768824	49.374
12.379	2.66	123408	3864324	50.626

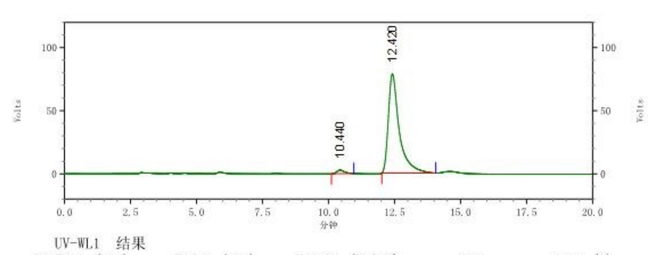

RetTime (min)	Width (min)	Height (Volts)	Area	Area (%)
10.440	0.84	11434	232132	2.512
12.420	2.05	313385	9008613	97.488

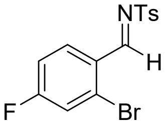

(*E*)-N-(2-bromo-4-fluorobenzylidene)-4-methylbenzenesulfonamide (S20)

Compound **S20** was synthesized following reference **8**

White solid. (R_f = 0.6, PE/EA = 5:1)

^{1}H NMR (400 MHz, $CDCl_3$) δ 9.37 (s, 1H), 8.19 (dd, *J* = 8.8, 6.1 Hz, 1H), 7.90 (d, *J* = 8.3 Hz, 2H), 7.41 (dd, *J* = 8.0, 2.5 Hz, 1H), 7.37 (d, *J* = 8.0 Hz, 2H), 7.14 – 7.08 (m, 1H), 2.45 (s, 3H).
^{13}C NMR (100 MHz, $CDCl_3$) δ 167.66, δ 165.96 (d, *J* = 262.4 Hz), 144.88, 134.51, 132.55 (d, *J* = 9.9 Hz), 129.85, 129.67, 128.23, 127.69 (d, *J* = 3.1 Hz), 121.13 (d, *J* = 24.8 Hz), 115.83 (d, *J* = 21.7 Hz), 21.64.
HRMS (ESI) *m/z* Calcd for $C_{14}H_{11}BrFNNaO_2S^+$ $[M + Na]^+$: 377.9570, Found: 377.9575.

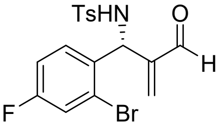

(R)-N-(1-(2-bromo-4-fluorophenyl)-2-formylallyl)-4-methylbenzenesulfonamide (6c)

White solid. (R_f = 0.6, PE/EA = 5:1)

^{1}H NMR (400 MHz, $CDCl_3$) δ 9.38 (s, 1H), 7.63 (d, *J* = 7.9 Hz, 2H), 7.35 – 7.30 (m, 1H), 7.24 – 7.12 (m, 3H), 6.85 (t, *J* = 7.1 Hz, 1H), 6.49 (s, 1H), 6.15 (s, 1H), 6.00 (d, *J* = 8.2 Hz, 1H), 5.69 (d, *J* = 8.2 Hz, 1H), 2.38 (s, 3H).
^{13}C NMR (100 MHz, $CDCl_3$) δ 192.62, 161.53 (d, *J* = 251.8 Hz), 146.58, 143.61, 137.24, 136.78, 133.04 (d, *J* = 3.3 Hz), 130.38 (d, *J* = 8.5 Hz), 129.42, 127.20, 123.03 (d, *J* = 9.3 Hz), 120.08 (d, *J* = 24.6 Hz), 114.67 (d, *J* = 21.0 Hz), 55.26, 21.42.
HRMS (ESI) *m/z* Calcd for $C_{17}H_{15}BrFNNaO_3S^+$ $[M + Na]^+$: 433.9832, Found: 433.9838.

Optical Rotation: $[\alpha]_D^{25}$ = - 2.5° (*c* = 0.6, $CHCl_3$).

HPLC analysis: Chiralcel IA-H (Hexane/*i*-PrOH = 80:20, flow rate = 1.0 mL/min, wave length = 254 nm), t_R = 11.363 min (minor), t_R = 11.983 min (major), 90% **ee**.

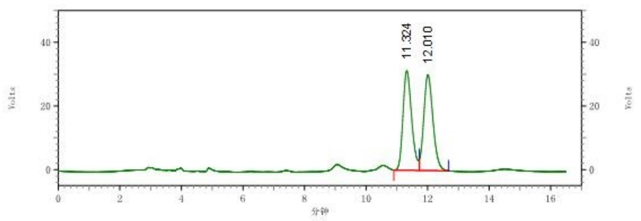

UV-WL1 结果

RetTime (min)	Width (min)	Height (Volts)	Area	Area (%)
11.324	0.83	124825	2443366	49.466
12.010	0.95	120306	2496113	50.534

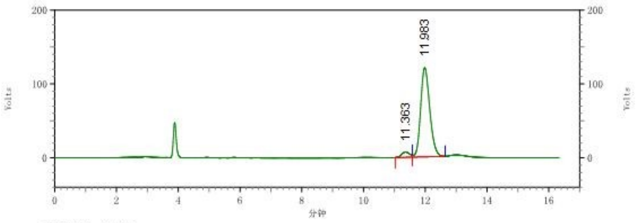

UV-WL1 结果

RetTime (min)	Width (min)	Height (Volts)	Area	Area (%)
11.363	0.56	28587	509765	4.890
11.983	1.06	482566	9914482	95.110

The absolute configuration of chiral product **6b, 6c** was assigned by analogy to those reported by similar catalysis, see reference **9**.

XII. Mechanistic studies

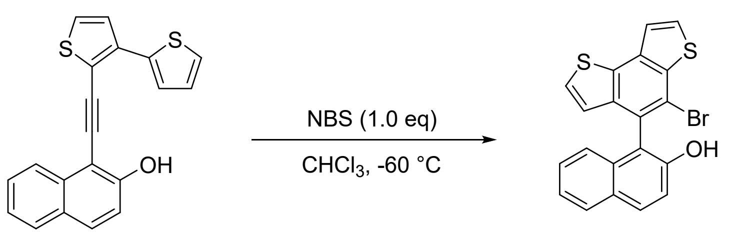

1a (±) **2a** 85%

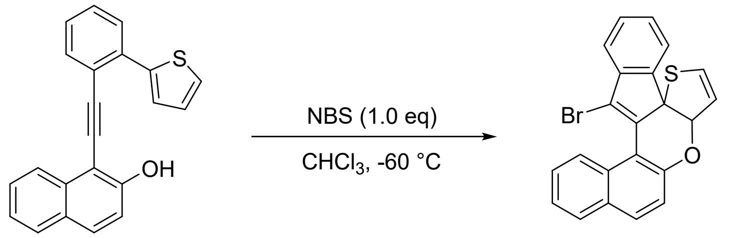

3a (±) **4a** 89%

Reaction condition: A solution of **1** or **3** (0.2 mmol, 1.0 equiv.) in $CHCl_3$ (2 mL) was stirred at -60 °C for 30 min, then NBS (0.2 mmol, 1.0 equiv.) was added. After stirring at -60 °C for 3 h, the reaction mixture was concentrated under reduced pressure. The resulting residue was purified by silica gel flash column chromatography using PE/EA eluent to afford the product **2** or **4**.

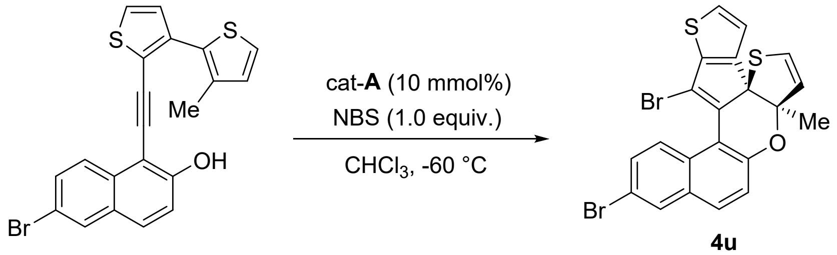

1x **4u** 92%, 99% ee

The control experiment was carried out following the general procedure (Condition [A]).

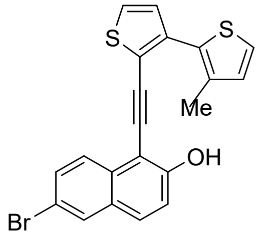

6-bromo-1-((3-methyl-[2,3'-bithiophen]-2'-yl)ethynyl)naphthalen-2-ol (1x)

Compound **1x** was synthesized following the general procedure (**Method B**).

Yellow solid. (R_f = 0.5, PE/EA = 6:1)

¹H NMR (400 MHz, CDCl$_3$) δ 7.85 (d, J = 2.0 Hz, 1H), 7.82 (d, J = 8.8 Hz, 1H), 7.58 (d, J = 9.0 Hz, 1H), 7.51 (dd, J = 8.8, 2.1 Hz, 1H), 7.35 (d, J = 5.2 Hz, 1H), 7.31 (d, J = 5.1 Hz, 1H), 7.14 (d, J = 4.9 Hz, 1H), 7.12 (s, 1H), 6.97 (d, J = 5.1 Hz, 1H), 6.14 (s, 1H), 2.29 (s, 3H).
¹³C NMR (100 MHz, CDCl$_3$) δ 156.20, 138.78, 135.74, 131.68, 130.72, 130.50, 130.38, 130.09, 129.70, 129.42, 129.28, 126.78, 126.63, 124.54, 119.56, 117.78, 117.57, 102.93, 94.69, 88.14, 15.14.
HRMS (ESI) *m/z* Calcd for $C_{21}H_{13}BrNaOS_2^+$ [M + Na]$^+$: 446.9483, Found: 446.9483.

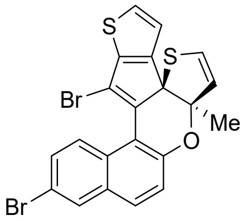

(3b*R*,6a*R*)-11,14-dibromo-6a-methyl-6aH-benzo[f]thieno[2',3':4,5]cyclopenta[1,2-c]thieno[3,2-b]chromene (4u)

Compound **4u** was synthesized in 92% yield (138.8 mg, 0.3 mmol scale) under condition [A].

Yellow oil. (R_f = 0.5, PE/EA = 20:1)

¹H NMR (400 MHz, CDCl$_3$) δ 7.94 (s, 1H), 7.84 (d, J = 9.0 Hz, 1H), 7.65 (d, J = 8.9 Hz, 1H), 7.57 (dd, J = 9.0, 2.1 Hz, 1H), 7.31 (d, J = 4.9 Hz, 1H), 7.16 (d, J = 8.9 Hz, 1H), 7.08 (d, J = 4.9 Hz, 1H), 6.62 (d, J = 5.9 Hz, 1H), 5.84 (d, J = 5.9 Hz, 1H), 1.35 (s, 3H).
¹³C NMR (100 MHz, CDCl$_3$) δ 155.67, 149.27, 146.44, 136.85, 131.21, 130.59, 130.19, 129.33, 129.24, 128.48, 128.18, 127.93, 127.86, 121.80, 118.88, 117.60, 112.59, 111.74, 93.47, 69.21, 22.80.
HRMS (ESI) *m/z* Calcd for $C_{21}H_{12}Br_2NaOS_2^+$ [M + Na]$^+$: 524.8589, Found: 524.8593.

HPLC analysis: Chiralcel IA-H (Hexane/*i*-PrOH = 95:5, flow rate = 1.0 mL/min, wave length = 254 nm), t_R = 5.228 min (minor), t_R = 5.918 min (major), 99% **ee**

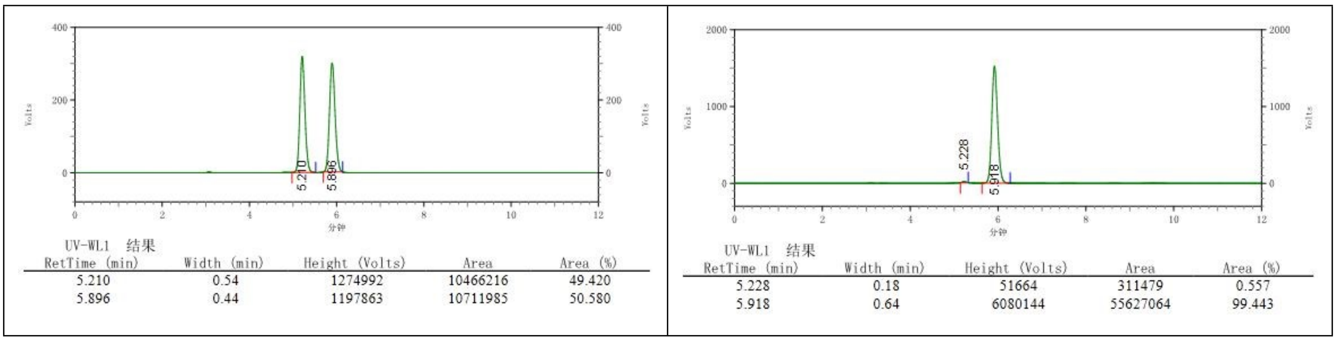

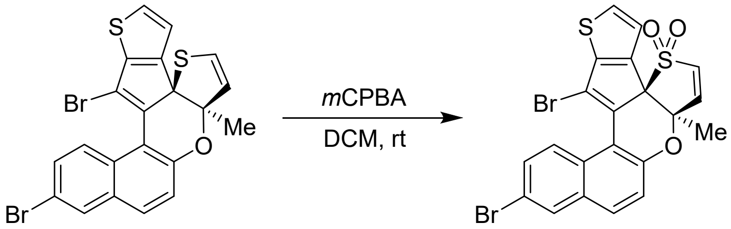

4u
99% ee

4u'
69%, 99% ee

4u (126 mg, 0.25 mmol, 1.0 equiv) and *m*CPBA (172 mg, 1.0 mmol, 4.0 equiv) were weighed and added into a flask, DCM (2 mL) added into the flask. Then, the resulting solution was kept stirring for 8 hours. Quenched with aq. Na_2CO_3, extracted with DCM, washed with brine, dried over Na_2SO_4 and filtered, concentrated under reduced pressure and purified by column chromatography on silica gel (PE/EA = 4:1) to afford the desired product **4u'** (92 mg, 0.17 mmol, 69% yield).

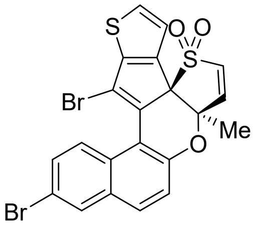

(3b*R*,6a*R*)-11,14-dibromo-6a-methyl-6aH-benzo[f]thieno[2',3':4,5]cyclopenta[1,2-c]thieno[3,2-b]chromene 4,4-dioxide (4u')

white solid. (R_f = 0.4, PE/EA = 4:1)

^{1}H NMR (400 MHz, $CDCl_3$) δ 7.98 (s, 1H), 7.93 (d, *J* = 8.9 Hz, 1H), 7.71 (d, *J* = 8.8 Hz, 1H), 7.64 (dd, *J* = 8.9, 2.0 Hz, 1H), 7.42 (d, *J* = 4.9 Hz, 1H), 7.25 – 7.16 (m, 2H), 6.64 (d, *J* = 6.8 Hz, 1H), 6.56 (d, *J* = 6.9 Hz, 1H), 1.82 (s, 3H).
^{13}C NMR (100 MHz, $CDCl_3$) δ 151.79, 150.60, 141.39, 139.30, 134.08, 132.61, 131.73, 130.27, 130.09, 129.82, 129.04, 127.84, 127.20, 125.68, 119.34, 118.65, 118.13, 116.15, 83.64, 82.60, 25.01.
HRMS (ESI) m/z Calcd for $C_{21}H_{12}Br_2NaO_3S_2^+$ [M + Na]$^+$: 556.8487, Found: 556.8487.

HPLC analysis: Chiralcel IA-H (Hexane/*i*-PrOH = 70:30, flow rate = 1.0 mL/min, wave length = 254 nm), t_R = 6.744 min (minor), t_R = 7.408 min (major), 99% **ee**

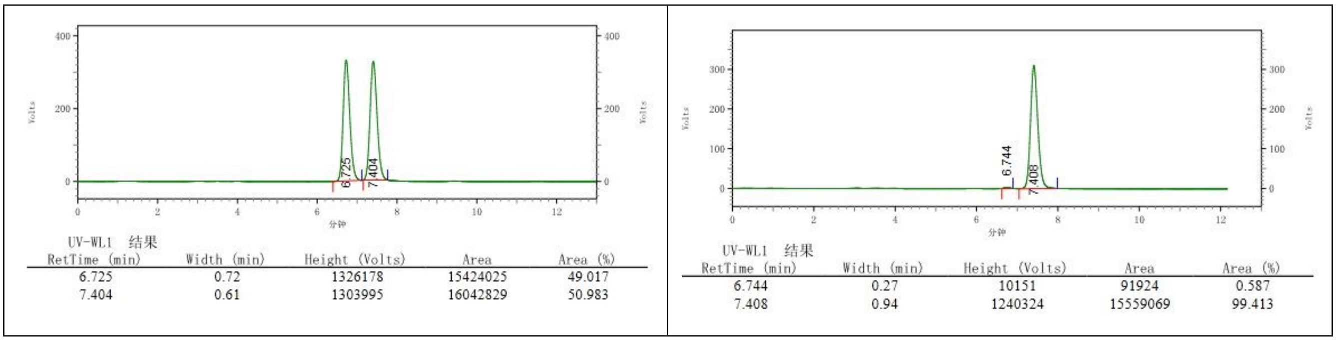

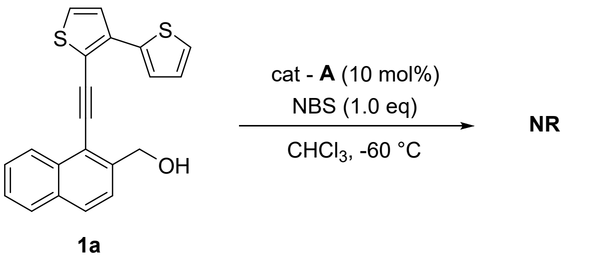

The control experiment was carried out following the general procedure (Condition [A]). After stirred at -60°C for 24h, monitored by TLC, no reaction was observed.

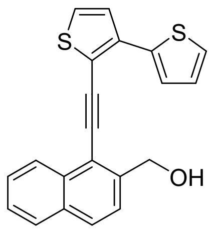

(1-([2,3'-bithiophen]-2'-ylethynyl)naphthalen-2-yl)methanol (7)

1-bromo-2-naphthaldehyde (705 mg, 3.0 mmol, 1.0 equiv), $PdCl_2(PPh_3)_2$ (70 mg, 0.1 mmol, 0.03 equiv) and CuI (30 mg, 0.16 mmol, 0.05 equiv) were weighed and added into an oven dried flask, evacuated and backfilled with nitrogen (3 times). **S2** (680 mg, 3.6 mmol, 1.2 equiv) in Et_3N (5 mL) and THF (5 mL) was injected into the flask. The mixture was heated to 70 °C for 3 h. Then the mixture was allowed to cool to rt, filtered through a pad of celite and washed with EA. Remove solvent under reduced pressure, purified by column chromatography on silica gel (PE/EA = 10:1) to afford the aldehyde. The aldehyde was dissolved in MeOH (5 mL) and DCM (5 mL), $NaBH_4$ (227mg, 6.0 mmol, 2.0 equiv) was added. After stirred at rt for 1h, the mixture was quenched by NH_4Cl. Extracted by DCM, remove solvent under reduced pressure. **7** was obtained without further purification. **^{1}H NMR** (400 MHz, $CDCl_3$) δ 8.44 (d, *J* = 8.3 Hz, 1H), 7.87 (t, *J* = 8.7 Hz, 2H), 7.73 – 7.62 (m, 2H), 7.55 (dt, *J* = 21.7, 7.2 Hz, 2H), 7.41 – 7.26 (m, 3H), 7.10 (t, *J* = 4.4 Hz, 1H), 5.11 (s, 2H), 2.06 (s, 1H). **^{13}C NMR** (100 MHz, $CDCl_3$) δ 141.55, 137.80, 137.68, 133.23, 132.55, 129.29, 128.20, 127.40, 127.28, 127.25, 127.14, 126.39, 126.27, 125.61, 125.26, 125.20, 117.98, 116.63, 93.31, 92.61, 64.23. **HRMS (ESI)** m/z Calcd for $C_{21}H_{14}NaOS_2^+$ $[M + Na]^+$: 369.0378, Found: 369.0378.

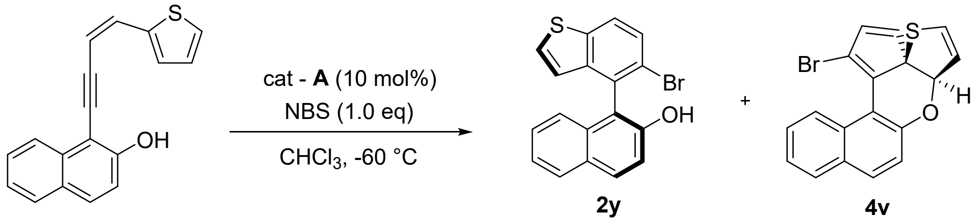

8

2y
85%, 97% ee

4v
9%, 98% ee

The control experiment was carried out following the general procedure (Condition [A]).

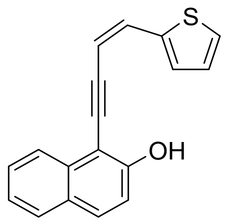

(*Z*)-1-(4-(thiophen-2-yl)but-3-en-1-yn-1-yl)naphthalen-2-ol (8)

Compound **8** was synthesized following the general procedure (**Method D**).

White solid. (R_f = 0.5, PE/EA = 6:1)

^{1}H NMR (400 MHz, $CDCl_3$) δ 8.23 (d, *J* = 8.4 Hz, 1H), 7.81 – 7.71 (m, 2H), 7.53 (dd, *J* = 8.4, 6.7 Hz, 1H), 7.37 (t, *J* = 7.5 Hz, 1H), 7.32 (d, *J* = 5.1 Hz, 1H), 7.26 (d, *J* = 3.6 Hz, 1H), 7.21 (d, *J* = 9.1 Hz, 1H), 7.08 – 7.01 (m, 1H), 6.99 (d, *J* = 11.4 Hz, 1H), 6.60 (s, 1H), 5.96 (d, *J* = 11.4 Hz, 1H).

^{13}C NMR (100 MHz, $CDCl_3$) δ 156.25, 140.25, 133.57, 131.87, 131.08, 129.97, 128.45, 128.26, 127.43, 126.82, 126.71, 124.97, 124.10, 116.53, 104.30, 103.05, 99.40, 91.48.

HRMS (ESI) *m/z* Calcd for $C_{18}H_{12}NaOS^+$ $[M + Na]^+$: 299.0501, Found: 299.0500.

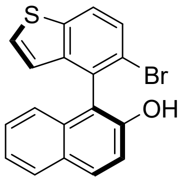

(*aS*)-1-(5-bromobenzo[b]thiophen-4-yl)naphthalen-2-ol (2y)

Compound **2y** was synthesized in 85% yield (90.2 mg, 0.3 mmol scale) under condition [A].

Purple oil. (R_f = 0.5, PE/EA = 6:1)

^{1}H NMR (400 MHz, $CDCl_3$) δ 7.87 (dd, *J* = 16.4, 8.8 Hz, 3H), 7.71 (d, *J* = 8.6 Hz, 1H), 7.37 – 7.23 (m, 4H), 7.04 (d, *J* = 8.2 Hz, 1H), 6.65 (d, *J* = 5.5 Hz, 1H), 4.89 (s, 1H).

^{13}C NMR (100 MHz, $CDCl_3$) δ 150.36, 141.91, 139.20, 132.42, 130.43, 129.78, 128.91, 128.75, 128.15, 126.86, 124.20, 123.70, 123.57, 122.22, 118.43, 117.57.

HRMS (ESI) *m/z* Calcd for $C_{18}H_{11}BrNaOS^+$ $[M + Na]^+$: 376.9606, Found: 376.9608.

HPLC analysis: Chiralcel IBIB-H (Hexane/*i*-PrOH = 90:10, flow rate = 1.0 mL/min, wave length = 254 nm), t_R = 19.375 min (minor), t_R = 20.872 min (major), 97% **ee**

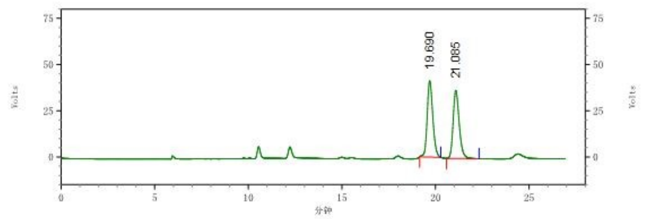

UV-WL1 结果

RetTime (min)	Width (min)	Height (Volts)	Area	Area (%)
19.690	1.13	165107	3448577	50.799
21.085	1.74	147500	3340057	49.201

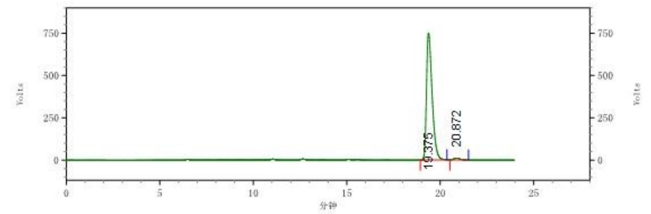

UV-WL1 结果

RetTime (min)	Width (min)	Height (Volts)	Area	Area (%)
19.375	1.42	3001842	62670641	98.512
20.872	0.99	42220	946925	1.488

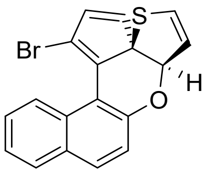

(3a*R*,6a*R*)-1-bromo-6aH-benzo[f]cyclopenta[c]thieno[3,2-b]chromene (4v)

Compound **4v** was synthesized in 9% yield (9.8 mg, 0.3 mmol scale) under condition [A].

Purple oil. (R_f = 0.5, PE/EA = 20:1)

^{1}H NMR (400 MHz, $CDCl_3$) δ 7.93 (d, *J* = 8.4 Hz, 1H), 7.76 (dd, *J* = 16.4, 8.5 Hz, 2H), 7.52 (t, *J* = 7.6 Hz, 1H), 7.38 (t, *J* = 7.5 Hz, 1H), 7.17 (d, *J* = 8.8 Hz, 1H), 6.70 – 6.51 (m, 3H), 6.04 (dd, *J* = 5.8, 3.0 Hz, 1H), 5.13 (d, *J* = 3.0 Hz, 1H).

^{13}C NMR (100 MHz, $CDCl_3$) δ 153.57, 138.32, 135.74, 133.79, 133.71, 130.18, 129.32, 129.17, 128.21, 126.99, 126.05, 123.86, 123.42, 117.53, 116.83, 111.79, 84.61, 70.69.

HRMS (ESI) *m/z* Calcd for $C_{18}H_{11}BrNaOS^+$ $[M + Na]^+$: 376.9606, Found: 376.9606.

HPLC analysis: Chiralcel IA-H (Hexane/*i*-PrOH = 95:5, flow rate = 1.0 mL/min, wave length = 254 nm), t_R = 4.760 min (minor), t_R = 5.699 min (major), 98% **ee**

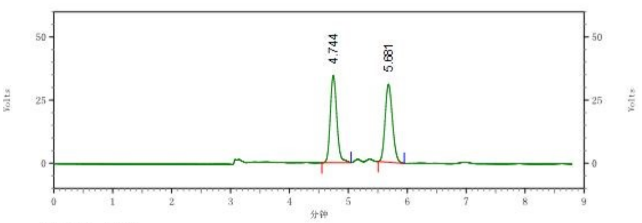

UV-WL1 结果

RetTime (min)	Width (min)	Height (Volts)	Area	Area (%)
4.744	0.49	138029	1062021	49.900
5.681	0.45	123506	1066272	50.100

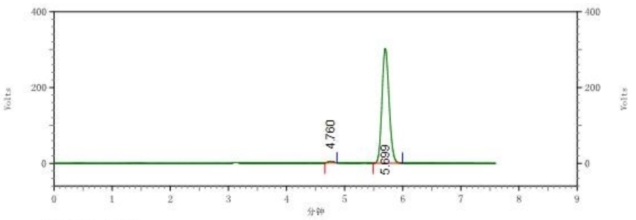

UV-WL1 结果

RetTime (min)	Width (min)	Height (Volts)	Area	Area (%)
4.760	0.21	17513	112891	1.089
5.699	0.51	1210187	10254155	98.911

XIII. ^{1}H and ^{13}C NMR spectra

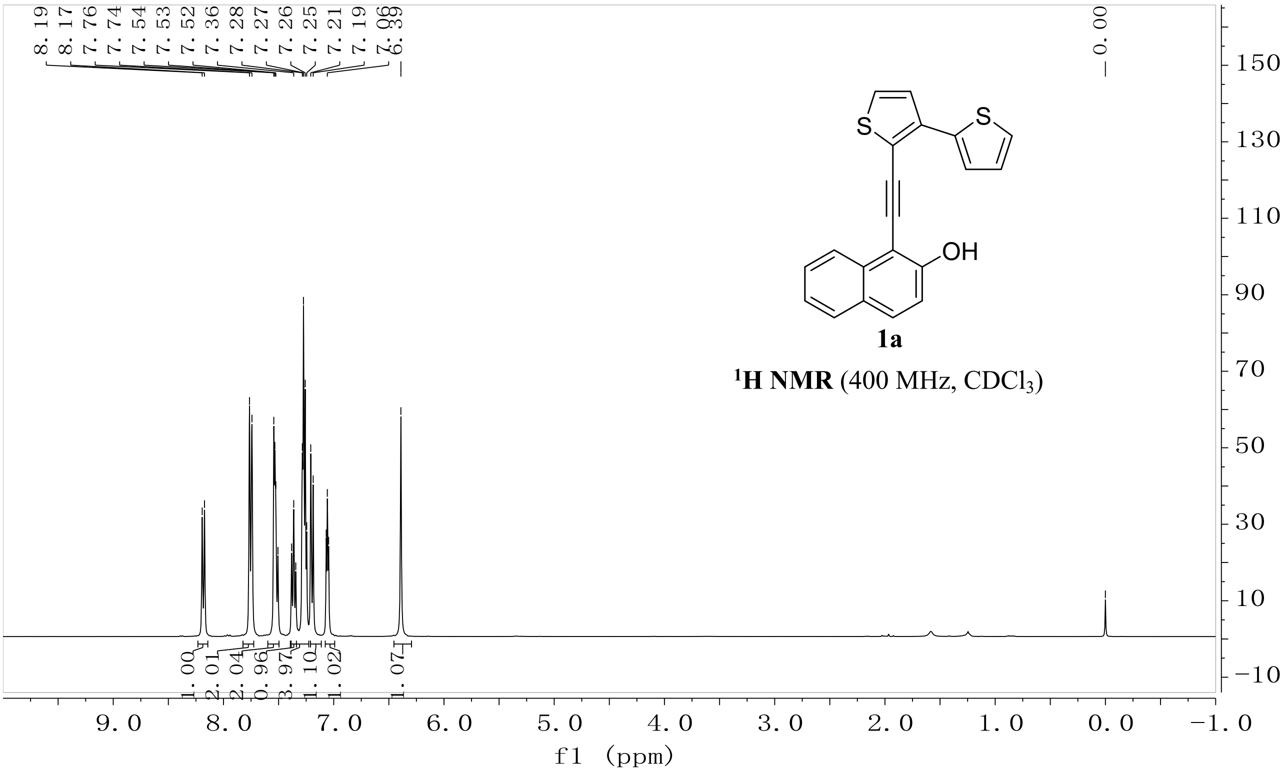

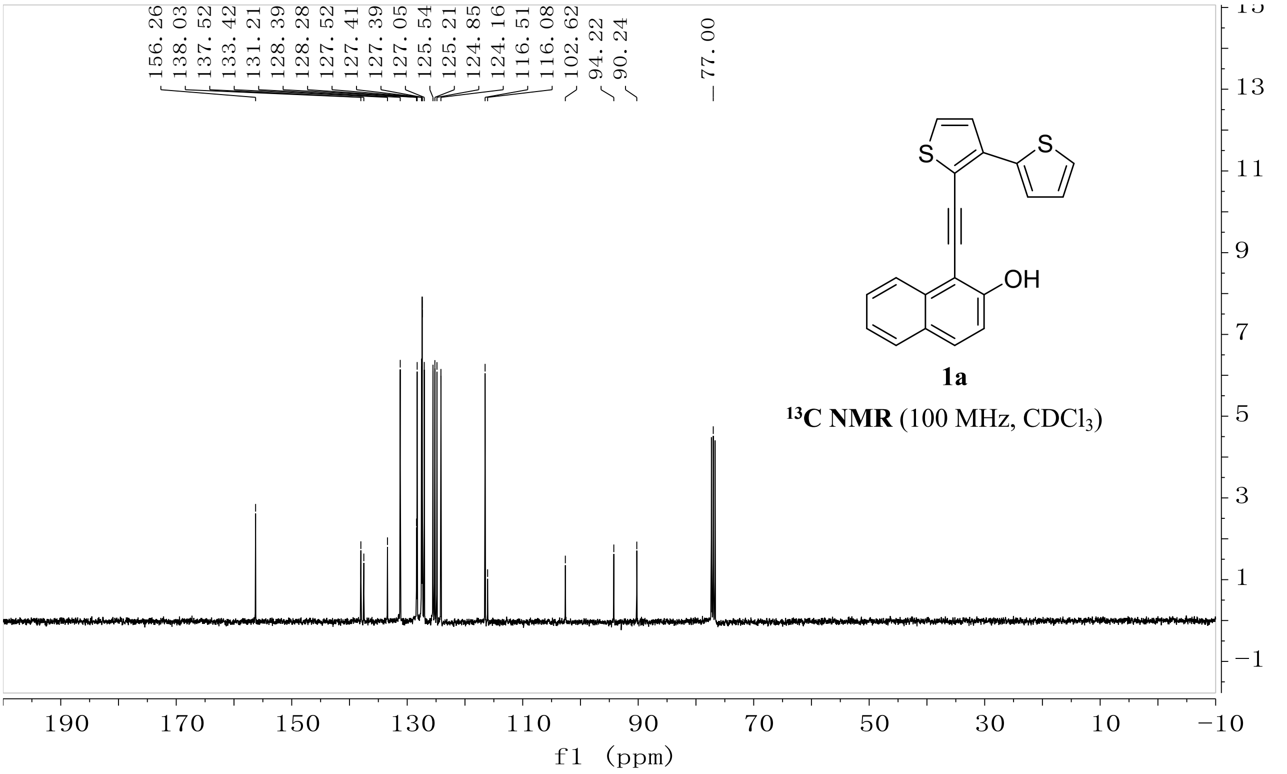

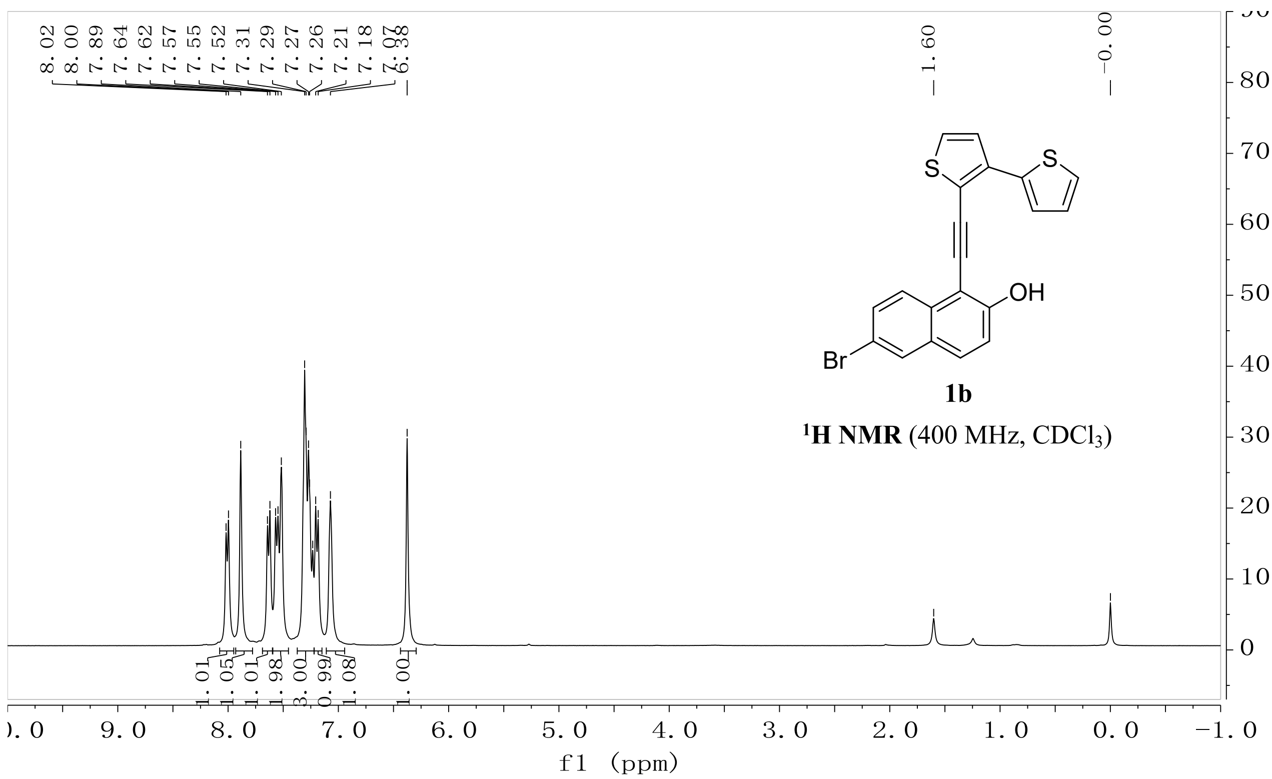

8.02
8.00
7.89
7.64
7.62
7.57
7.55
7.52
7.31
7.29
7.27
7.26
7.21
7.18
7.07
6.38
1.60
0.00
OH
S
S
Br
1b
1H NMR (400 MHz, CDCl3)
f1 (ppm)

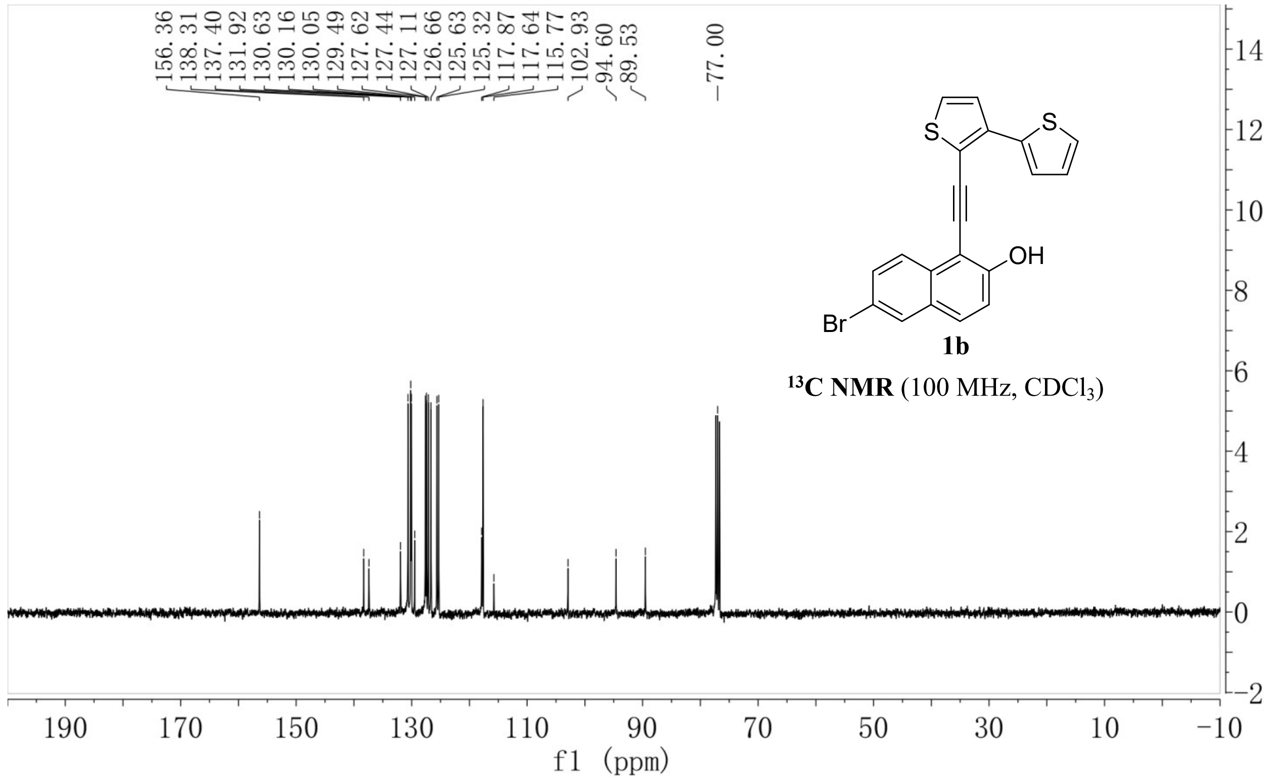

156.36
138.31
137.40
131.92
130.63
130.16
130.05
129.49
127.62
127.44
127.11
126.66
125.63
125.32
117.87
117.64
115.77
102.93
94.60
89.53
77.00
OH
S
S
Br
1b
13C NMR (100 MHz, CDCl3)
f1 (ppm)

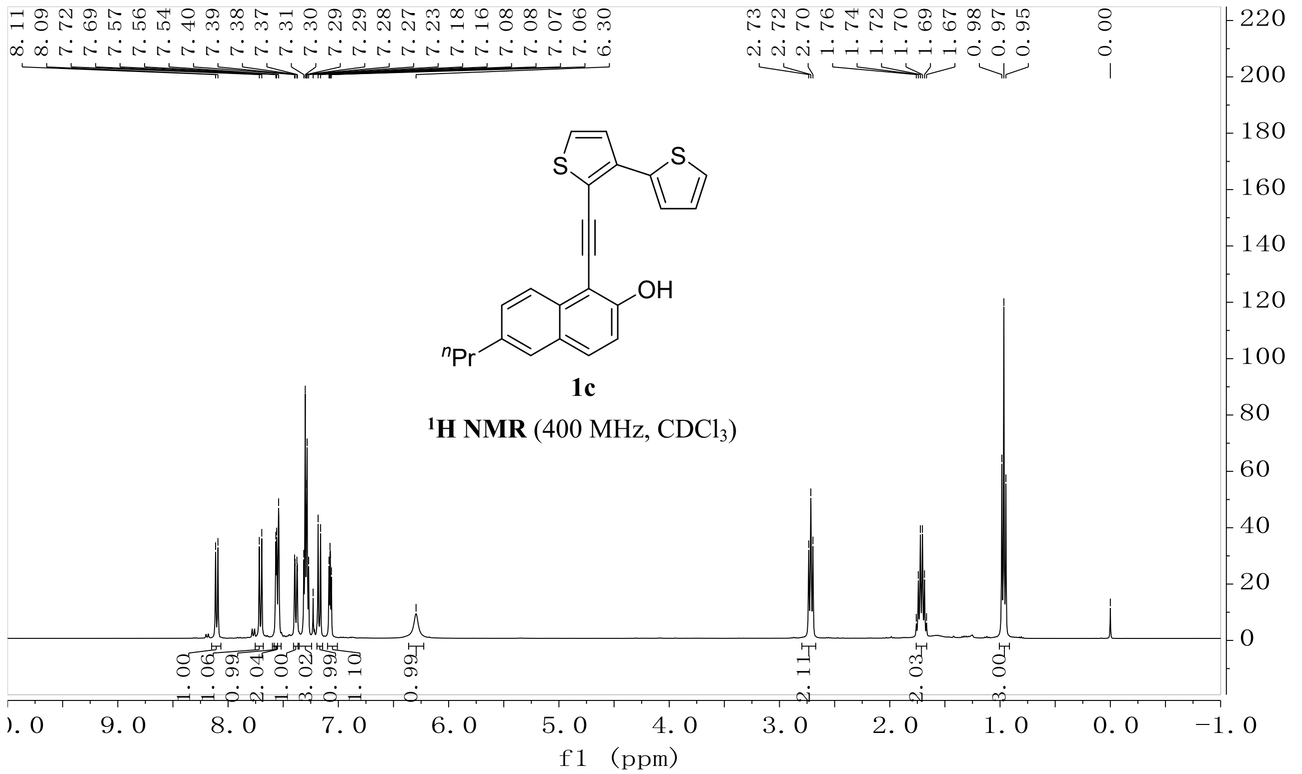

1c

^{1}H NMR (400 MHz, $CDCl_3$)

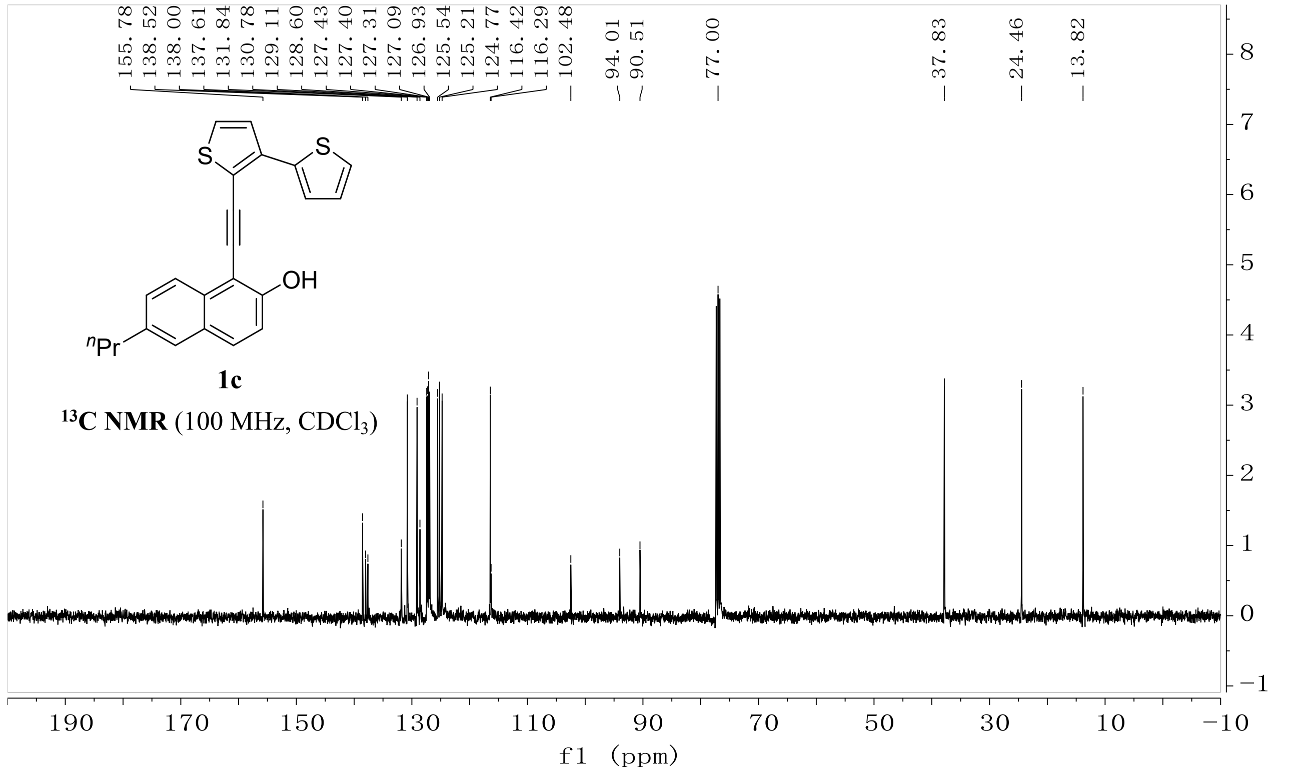

1c

^{13}C NMR (100 MHz, $CDCl_3$)

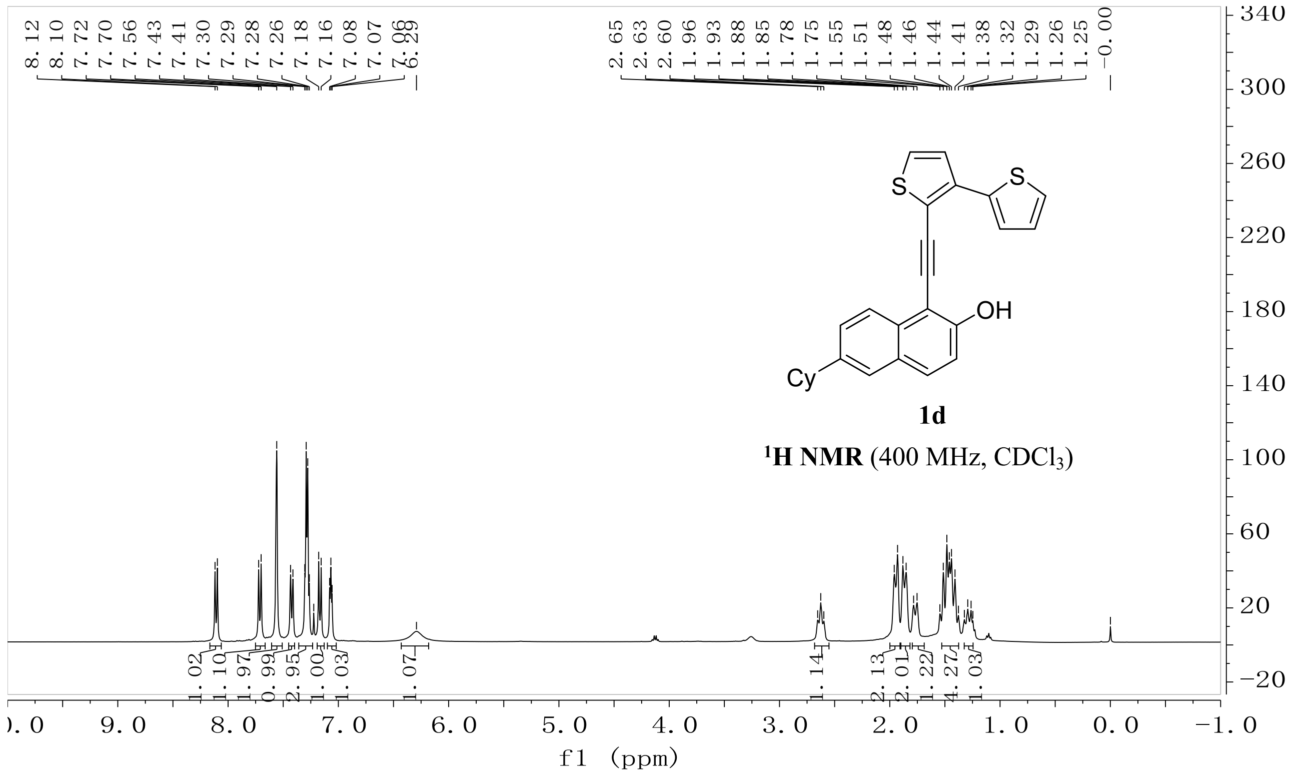
1d
¹H NMR (400 MHz, CDCl3)
f1 (ppm)

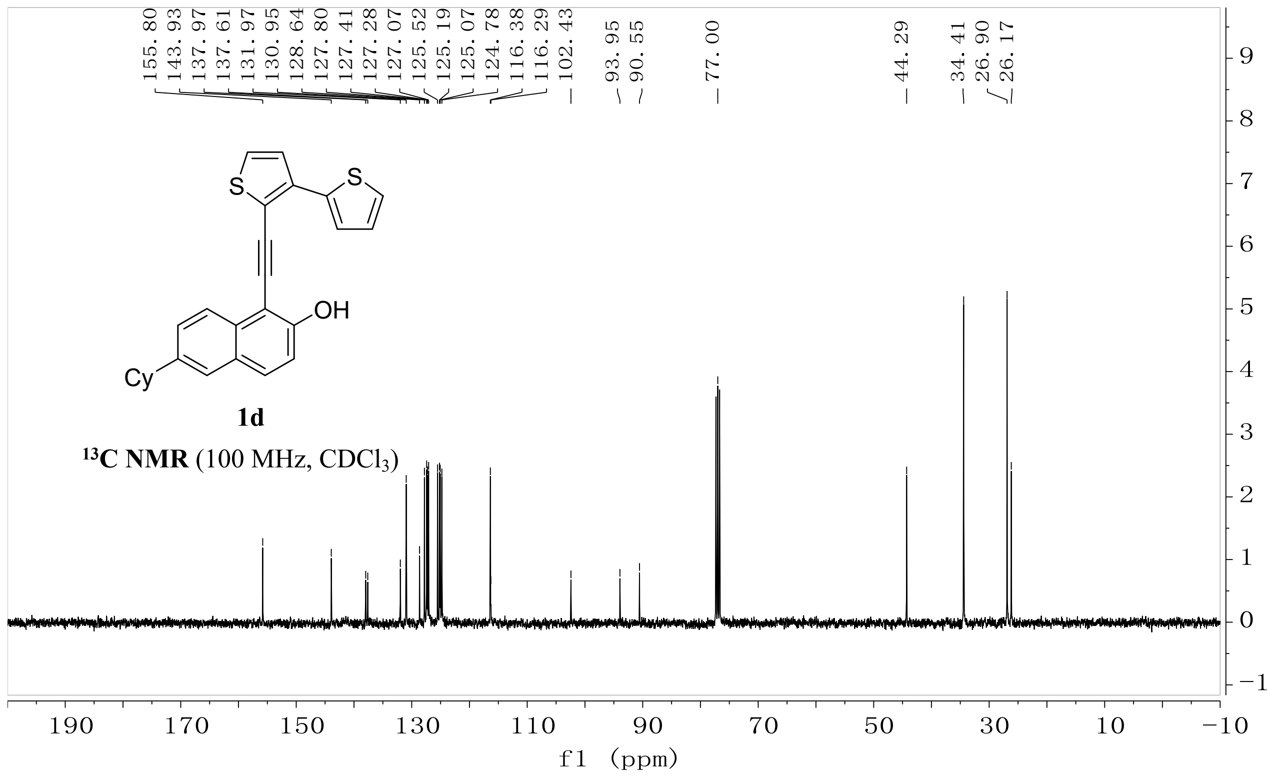
1d
¹³C NMR (100 MHz, CDCl3)
f1 (ppm)

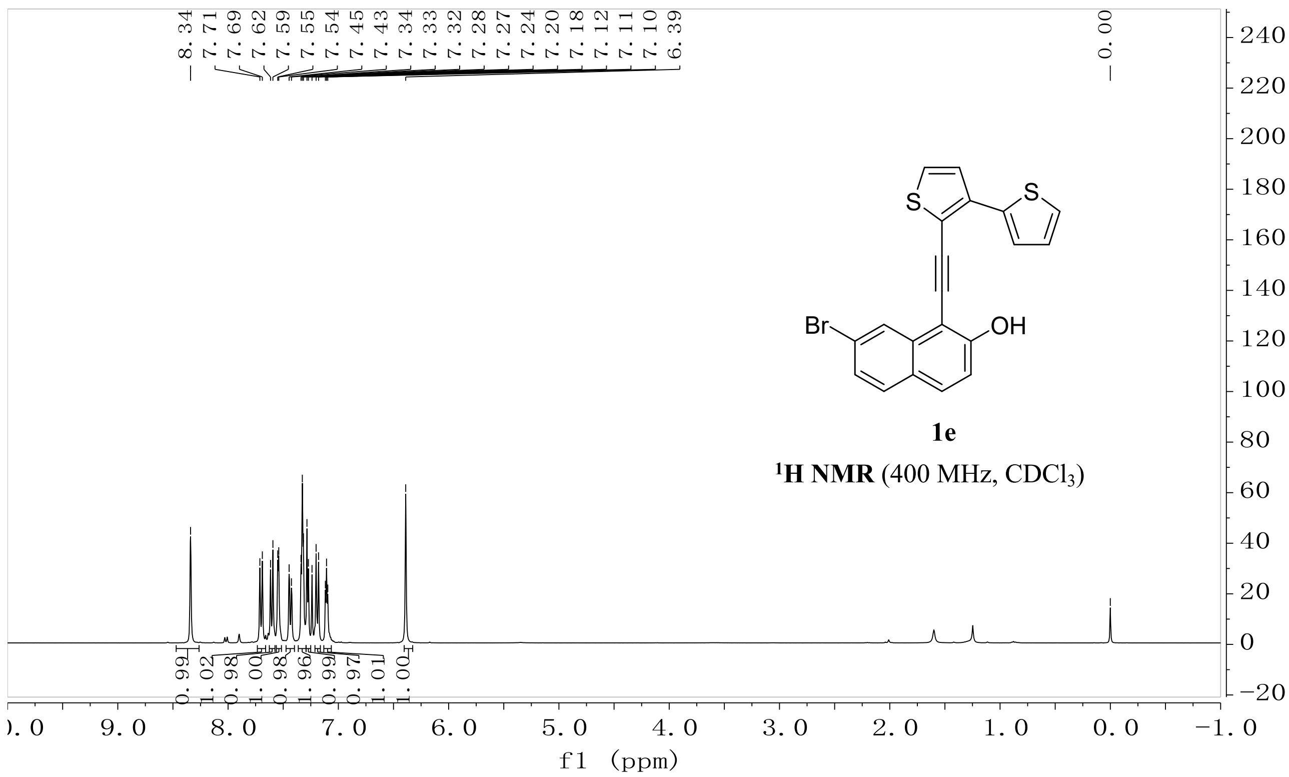
8.34
7.71
7.69
7.62
7.59
7.55
7.54
7.45
7.43
7.34
7.33
7.32
7.28
7.27
7.24
7.20
7.18
7.12
7.11
7.10
6.39
0.00
S
S
Br
OH
1e
¹H NMR (400 MHz, CDCl₃)
0.99
1.02
0.98
1.00
0.98
1.96
0.99
0.97
1.01
1.00
f1 (ppm)

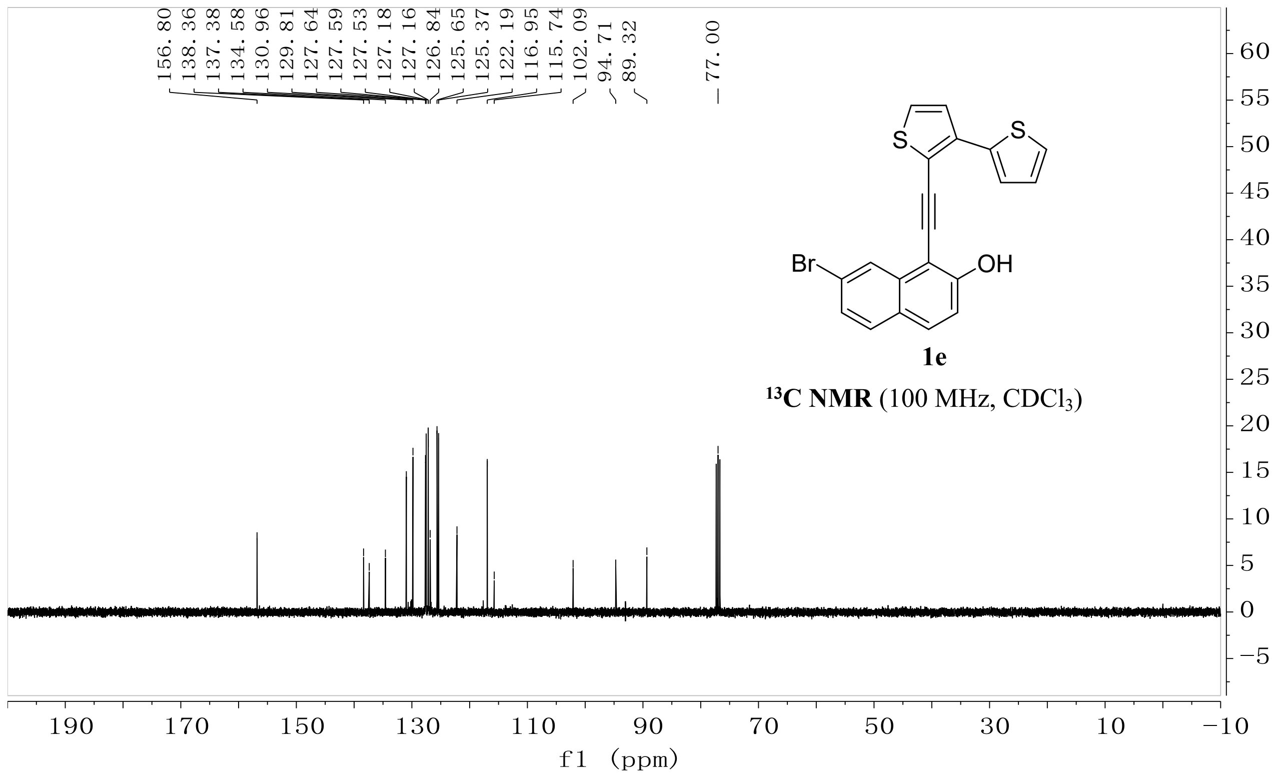
156.80
138.36
137.38
134.58
130.96
129.81
127.64
127.59
127.53
127.18
127.16
126.84
125.65
125.37
122.19
116.95
115.74
102.09
94.71
89.32
77.00
S
S
Br
OH
1e
¹³C NMR (100 MHz, CDCl₃)
f1 (ppm)

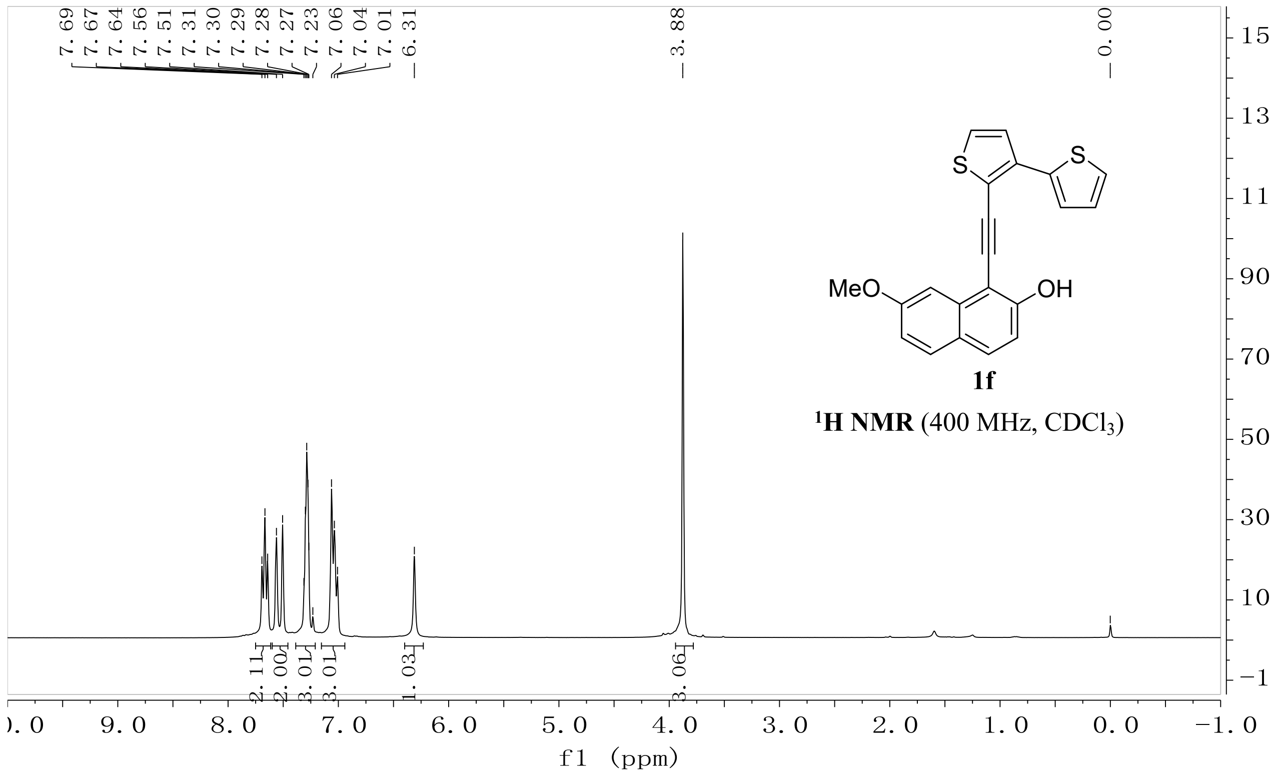
7.69
7.67
7.64
7.56
7.51
7.31
7.30
7.29
7.28
7.27
7.23
7.06
7.04
7.01
6.31
3.88
0.00
S
S
MeO
OH
1f
¹H NMR (400 MHz, CDCl₃)
2.11
2.00
3.01
3.01
1.03
3.06
f1 (ppm)

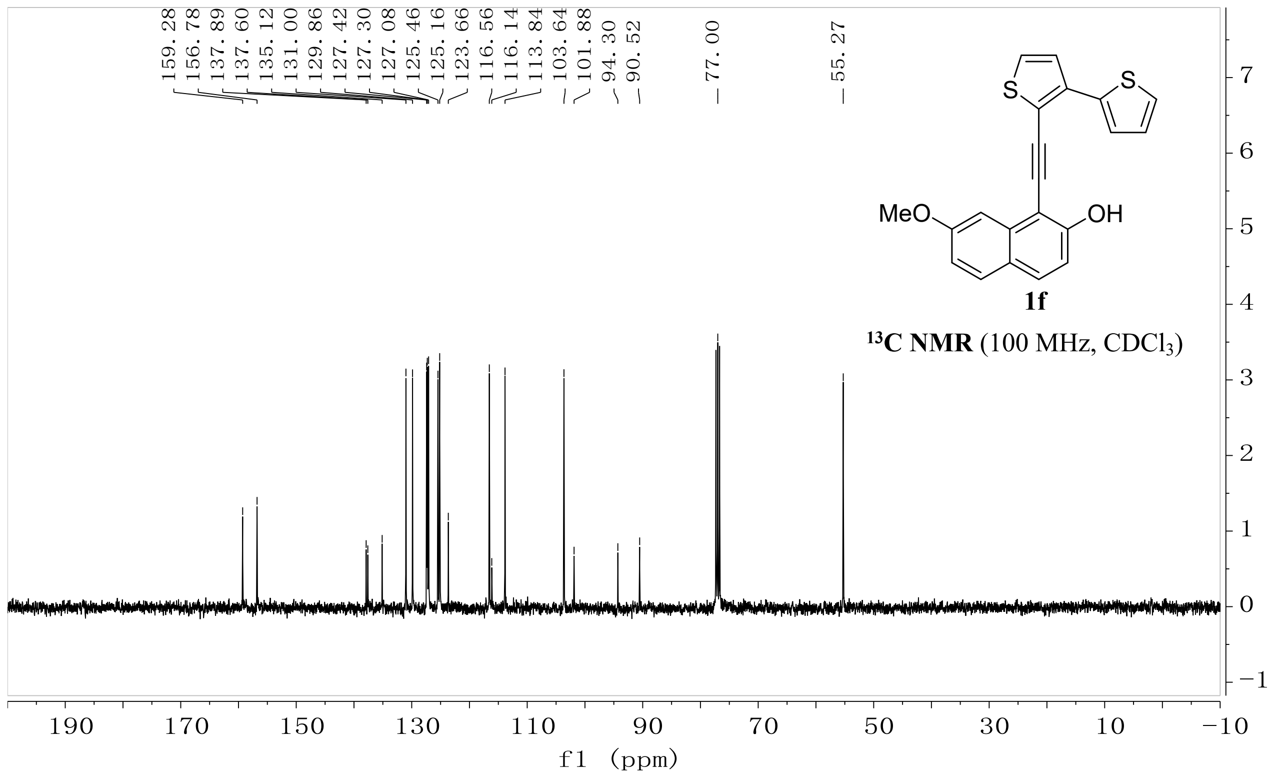
159.28
156.78
137.89
137.60
135.12
131.00
129.86
127.42
127.30
127.08
125.46
125.16
123.66
116.56
116.14
113.84
103.64
101.88
94.30
90.52
77.00
55.27
S
S
MeO
OH
1f
¹³C NMR (100 MHz, CDCl₃)
f1 (ppm)

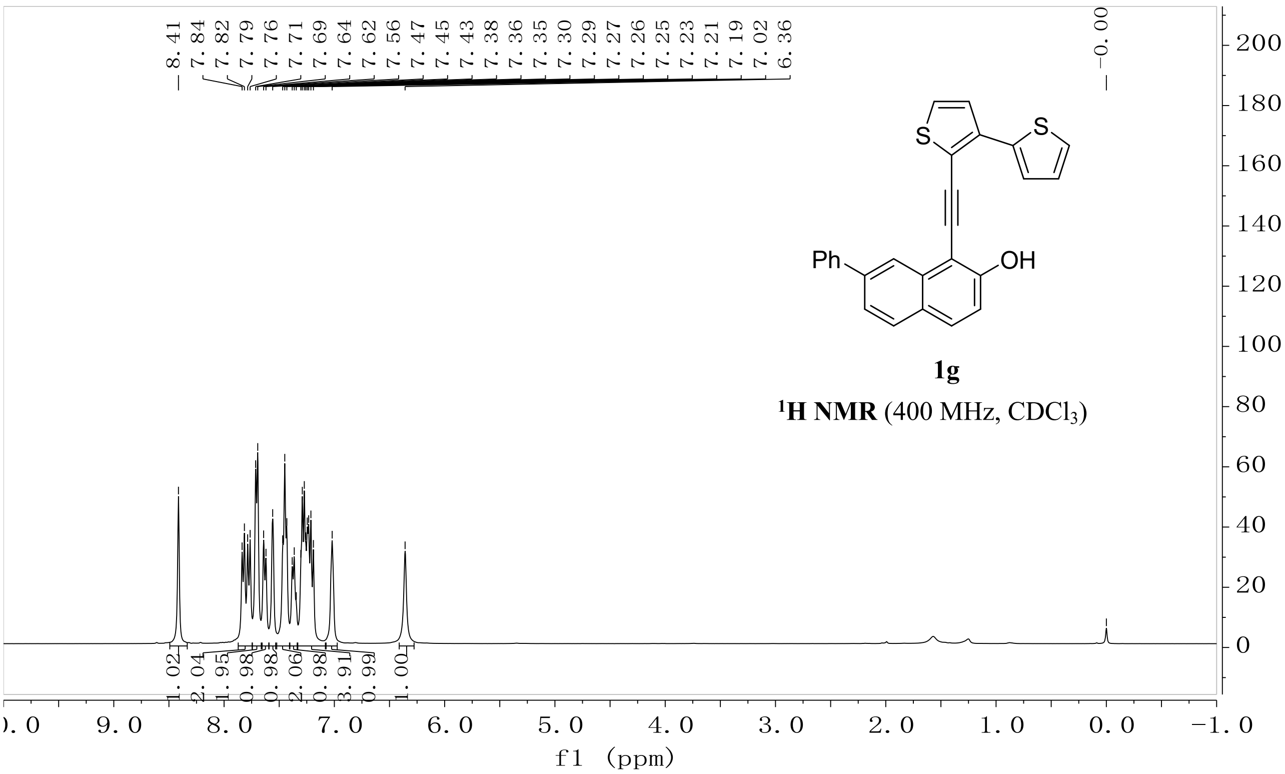

8.41
7.84
7.82
7.79
7.76
7.71
7.69
7.64
7.62
7.56
7.47
7.45
7.43
7.38
7.36
7.35
7.30
7.29
7.27
7.26
7.25
7.23
7.21
7.19
7.02
6.36
-0.00
1g
1H NMR (400 MHz, CDCl3)
f1 (ppm)

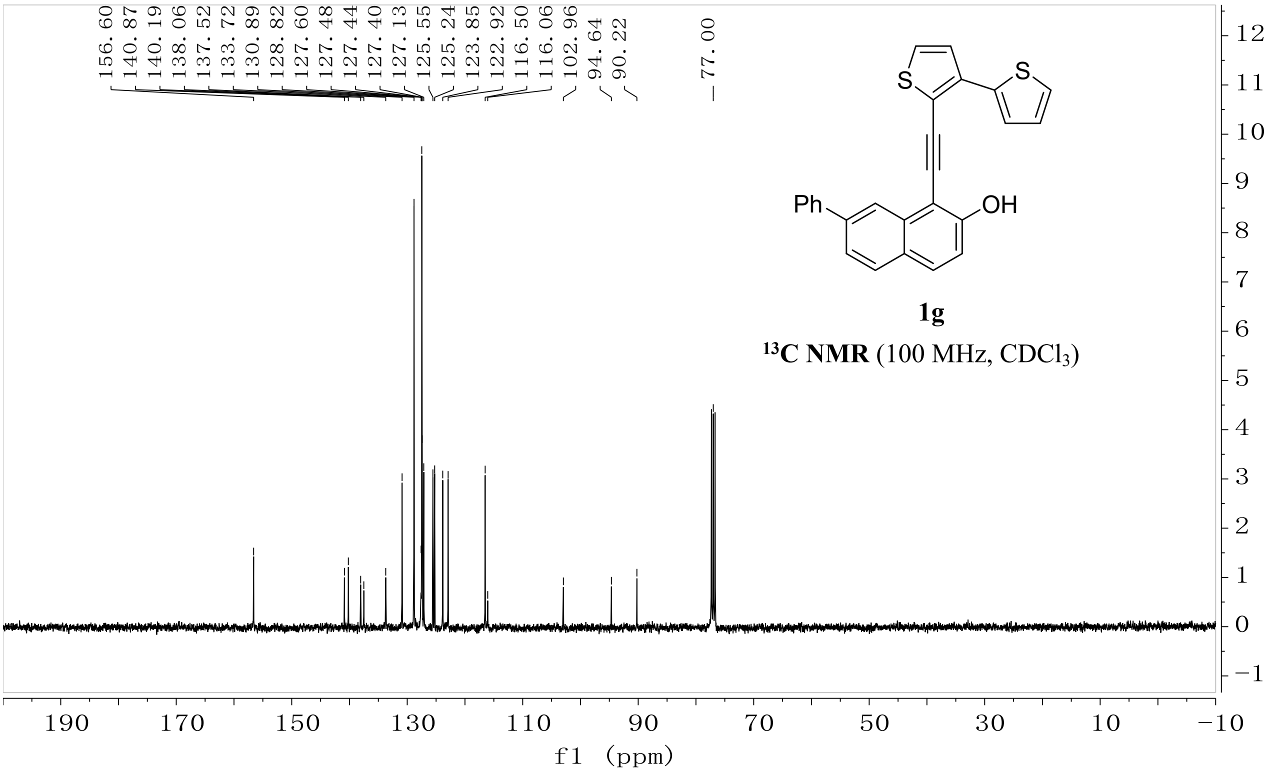

156.60
140.87
140.19
138.06
137.52
133.72
130.89
128.82
127.60
127.48
127.44
127.40
127.13
125.55
125.24
123.85
122.92
116.50
116.06
102.96
94.64
90.22
77.00
1g
13C NMR (100 MHz, CDCl3)
f1 (ppm)

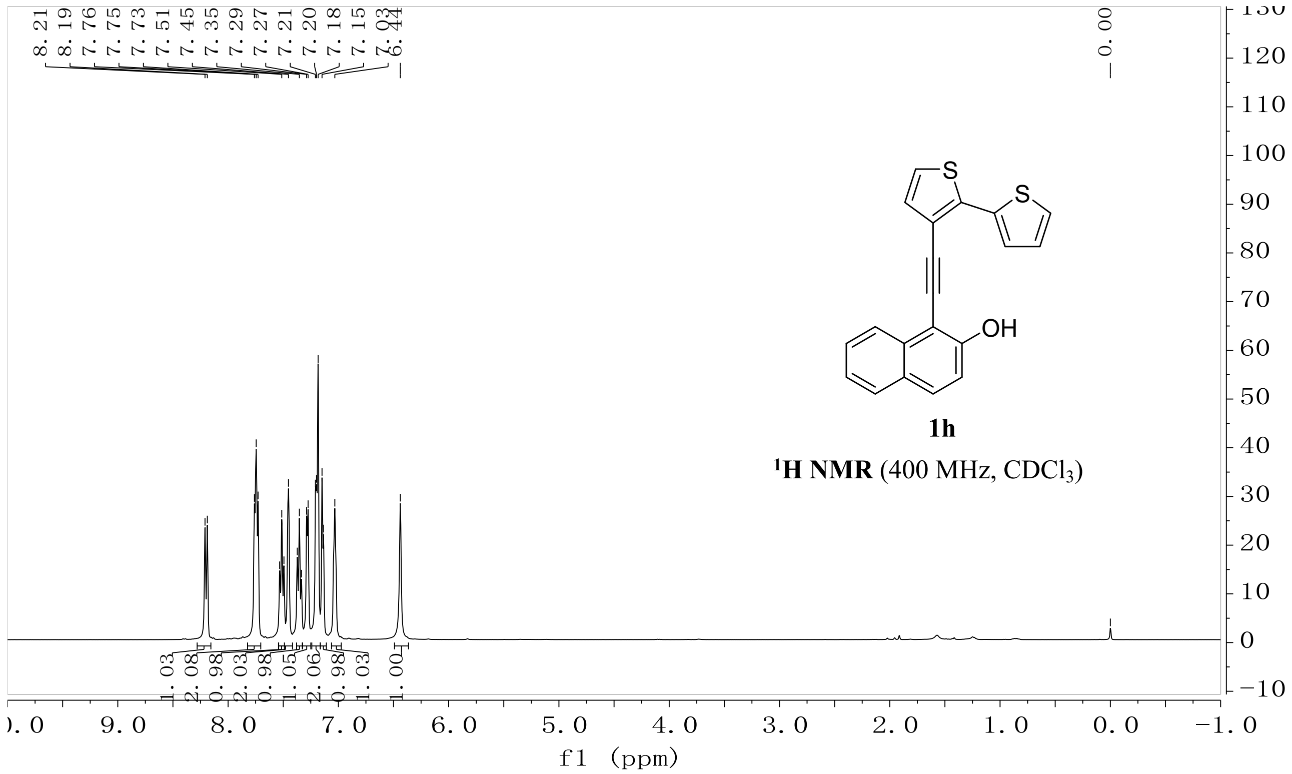
1h
^{1}H NMR (400 MHz, CDCl$_3$)
f1 (ppm)

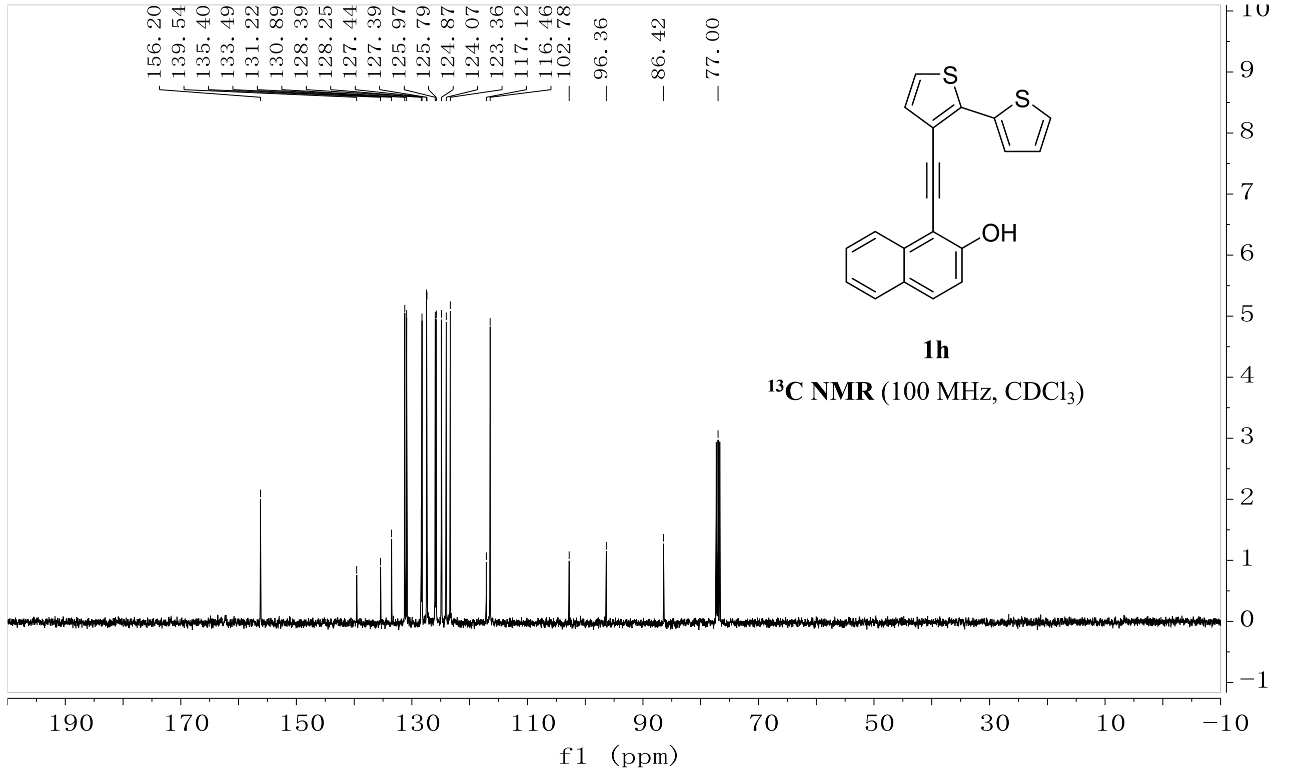
1h
^{13}C NMR (100 MHz, CDCl$_3$)
f1 (ppm)

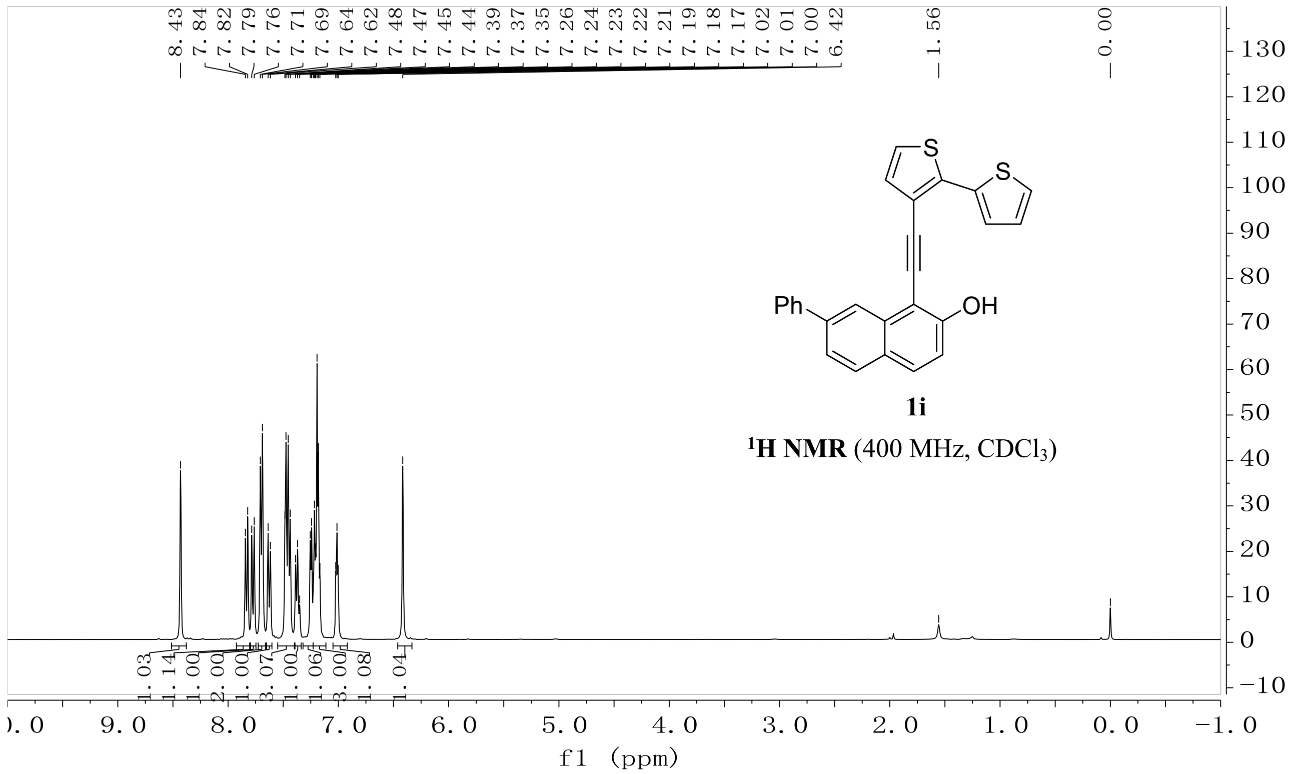
8.43
7.84
7.82
7.79
7.76
7.71
7.69
7.64
7.62
7.48
7.47
7.45
7.44
7.39
7.37
7.35
7.26
7.24
7.23
7.22
7.21
7.19
7.18
7.17
7.02
7.01
7.00
6.42
1.56
0.00
Ph
OH
S
S
1i
¹H NMR (400 MHz, CDCl₃)
f1 (ppm)

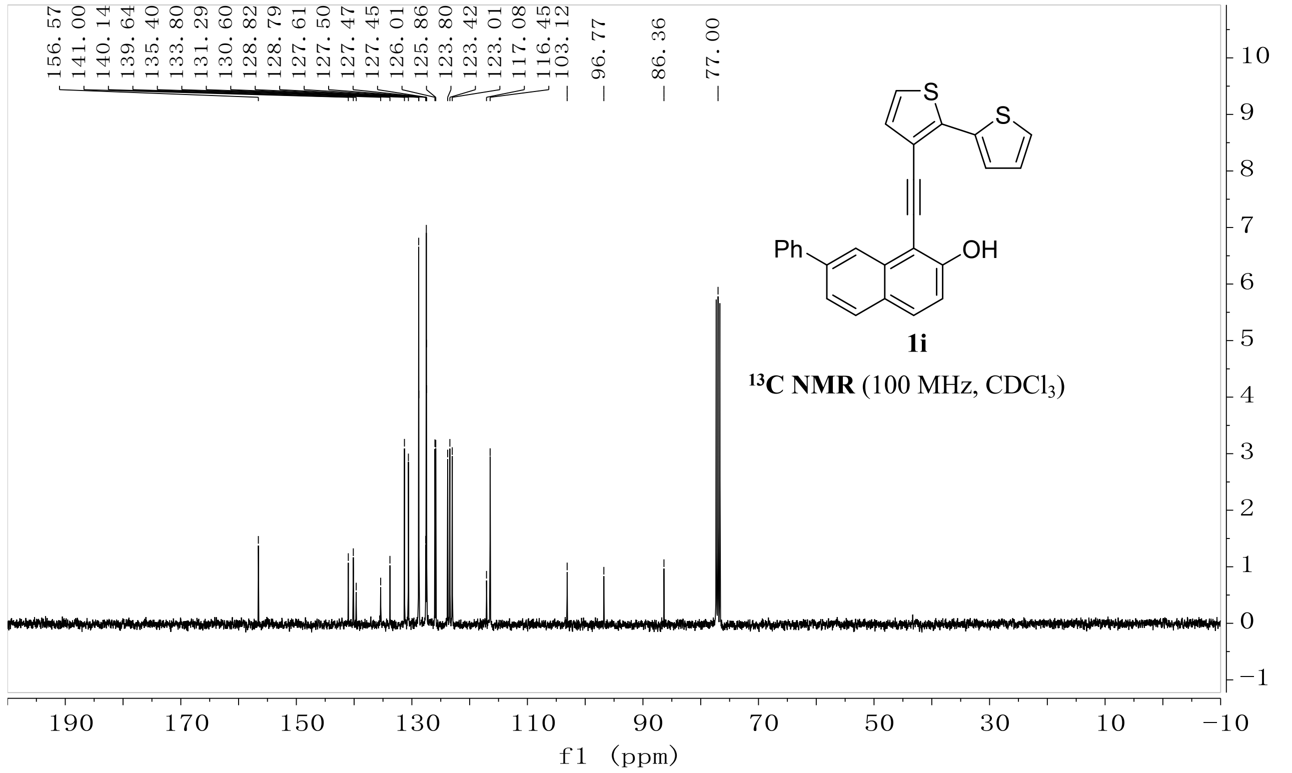
156.57
141.00
140.14
139.64
135.40
133.80
131.29
130.60
128.82
128.79
127.61
127.50
127.47
127.45
126.01
125.86
123.80
123.42
123.01
117.08
116.45
103.12
96.77
86.36
77.00
Ph
OH
S
S
1i
¹³C NMR (100 MHz, CDCl₃)
f1 (ppm)

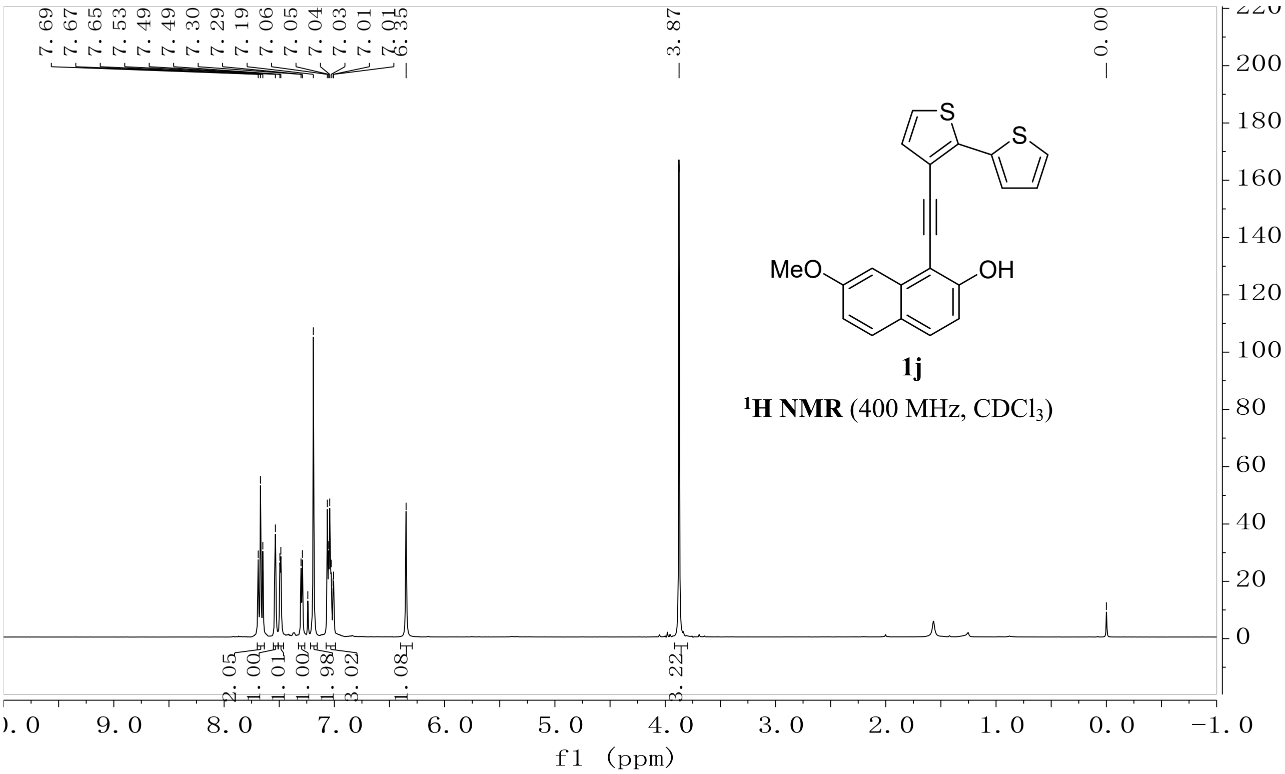

1j

^{1}H NMR (400 MHz, $CDCl_3$)

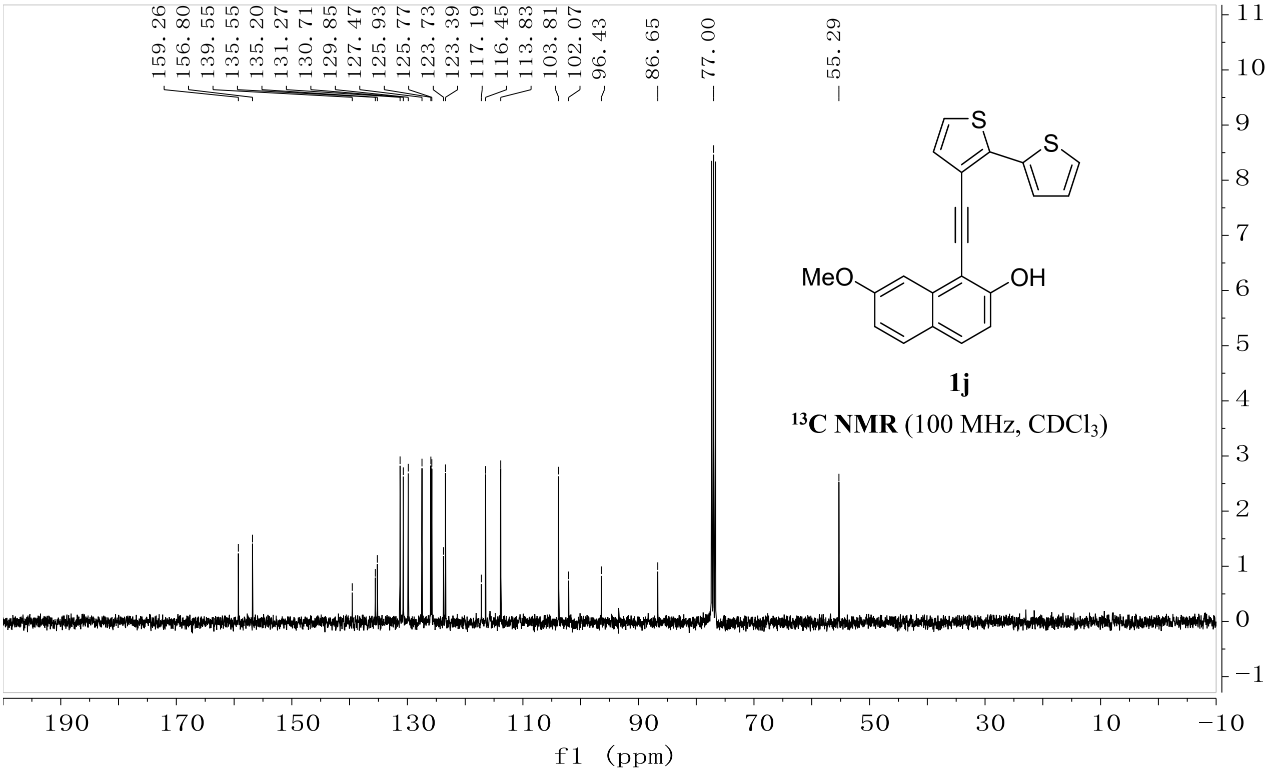

1j

^{13}C NMR (100 MHz, $CDCl_3$)

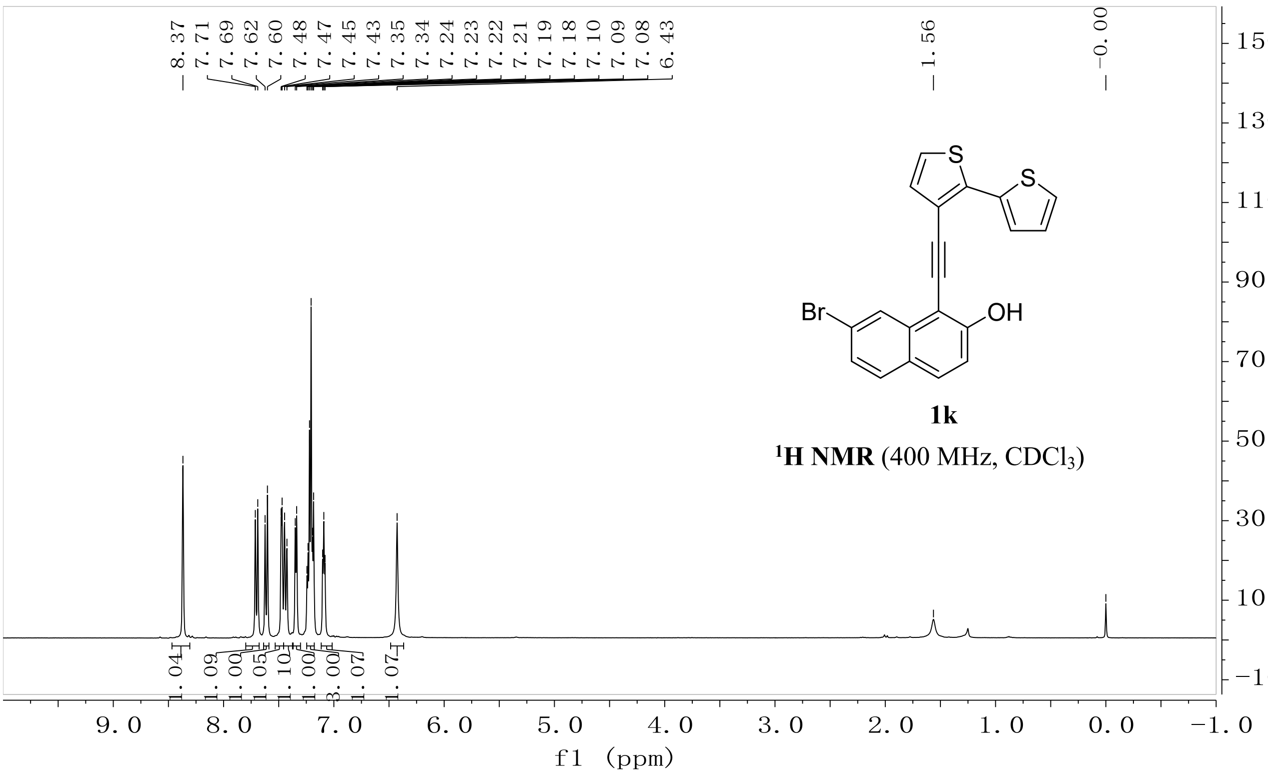

1k
^{1}H NMR (400 MHz, CDCl$_3$)

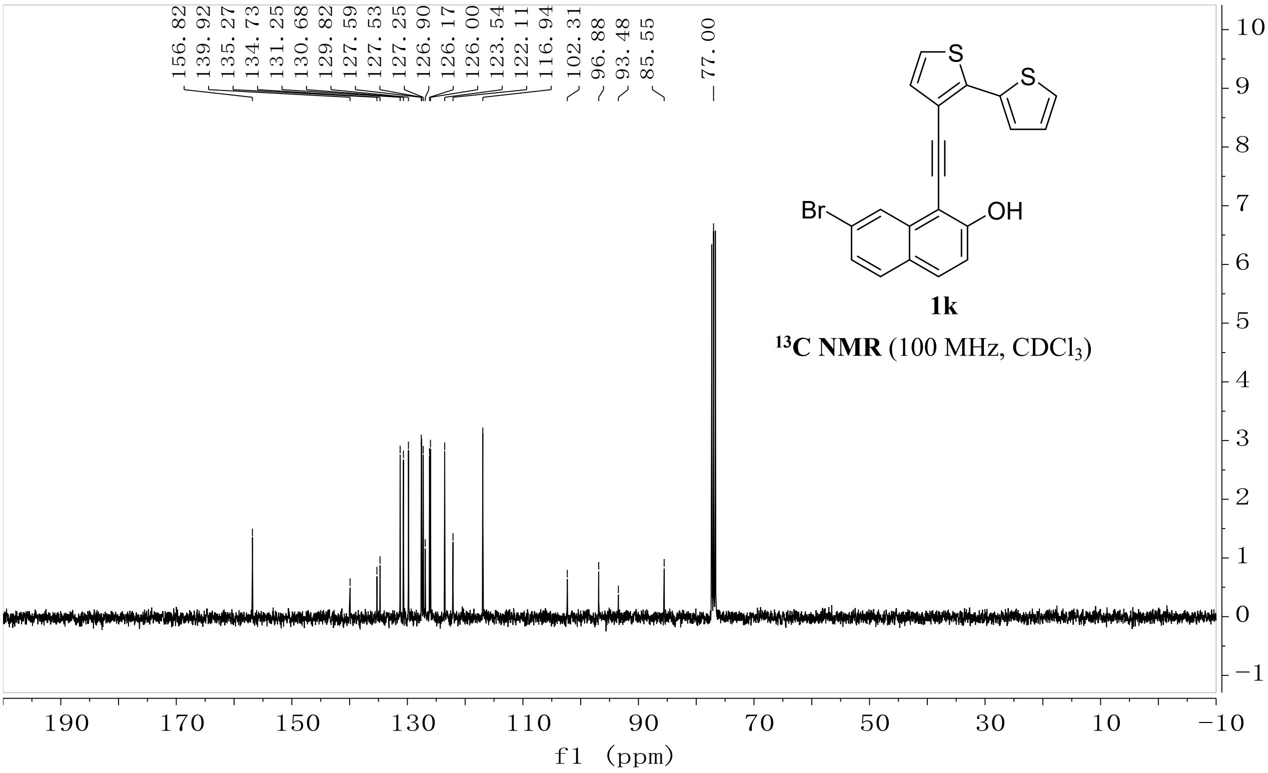

1k
^{13}C NMR (100 MHz, CDCl$_3$)

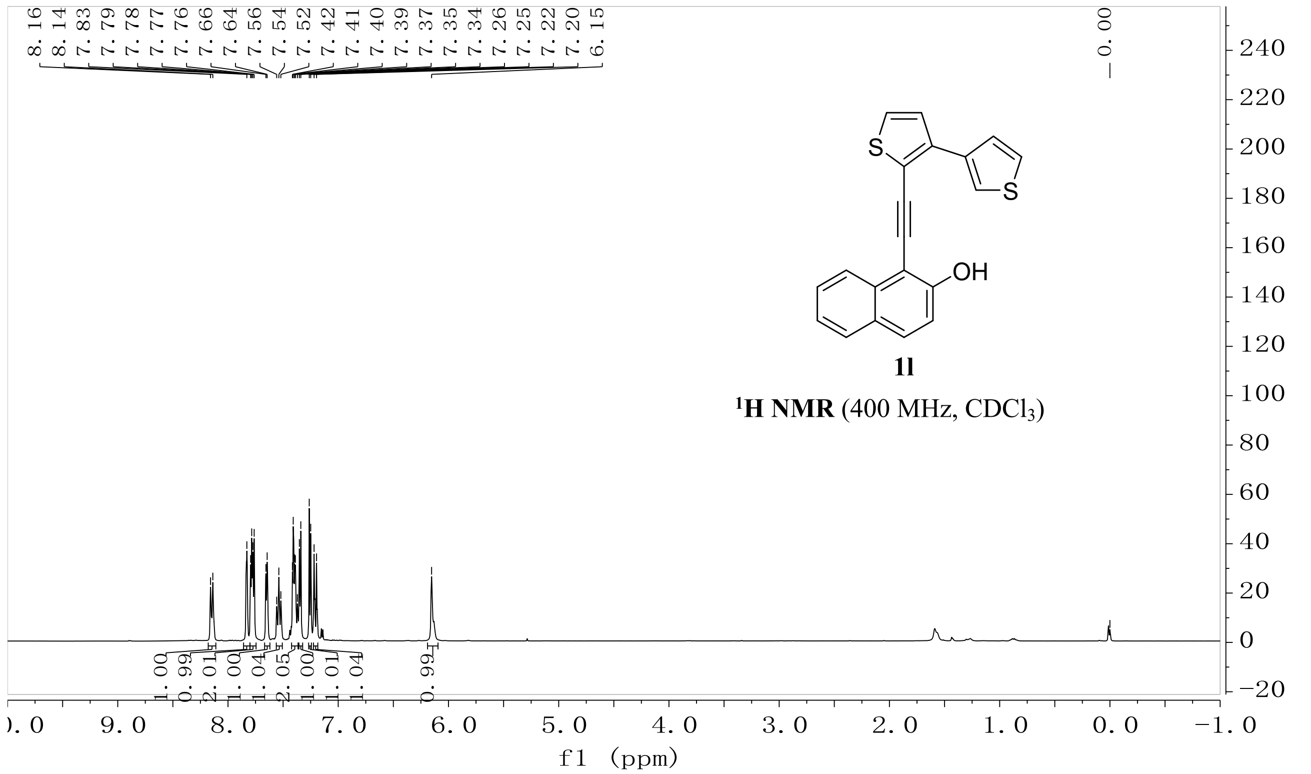
8.16
8.14
7.83
7.79
7.78
7.77
7.76
7.66
7.64
7.56
7.54
7.52
7.42
7.41
7.40
7.39
7.37
7.35
7.34
7.26
7.25
7.22
7.20
6.15
0.00
1l
^{1}H NMR (400 MHz, CDCl$_3$)
f1 (ppm)

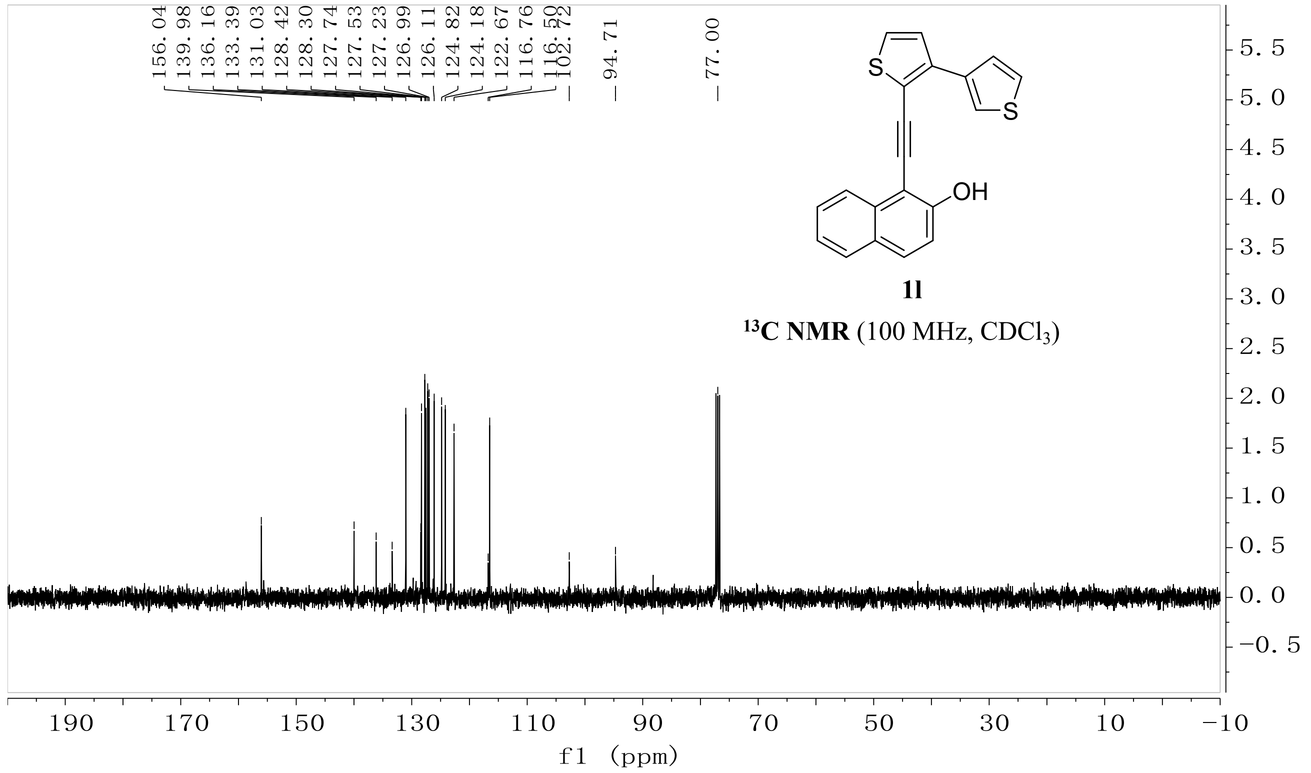
156.04
139.98
136.16
133.39
131.03
128.42
128.30
127.74
127.53
127.23
126.99
126.11
124.82
124.18
122.67
116.76
116.50
102.72
94.71
77.00
1l
^{13}C NMR (100 MHz, CDCl$_3$)
f1 (ppm)

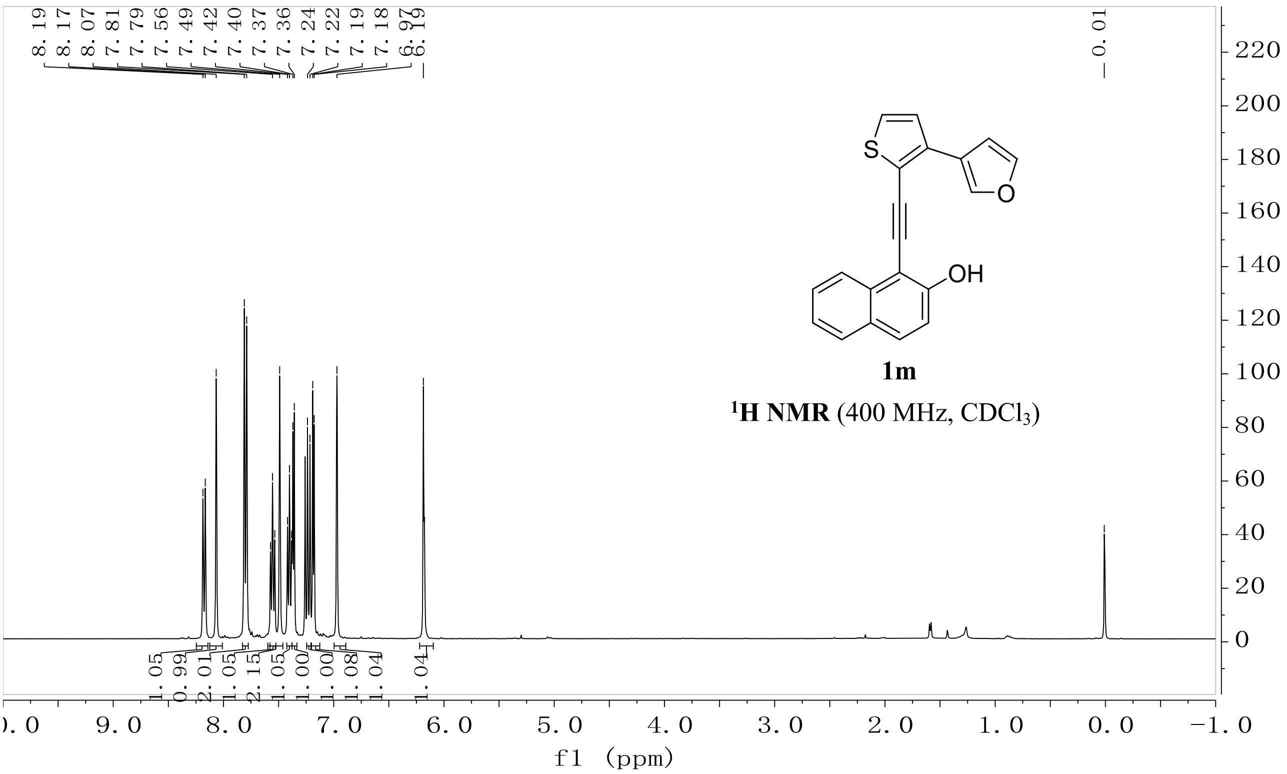

1m

^{1}H NMR (400 MHz, $CDCl_3$)

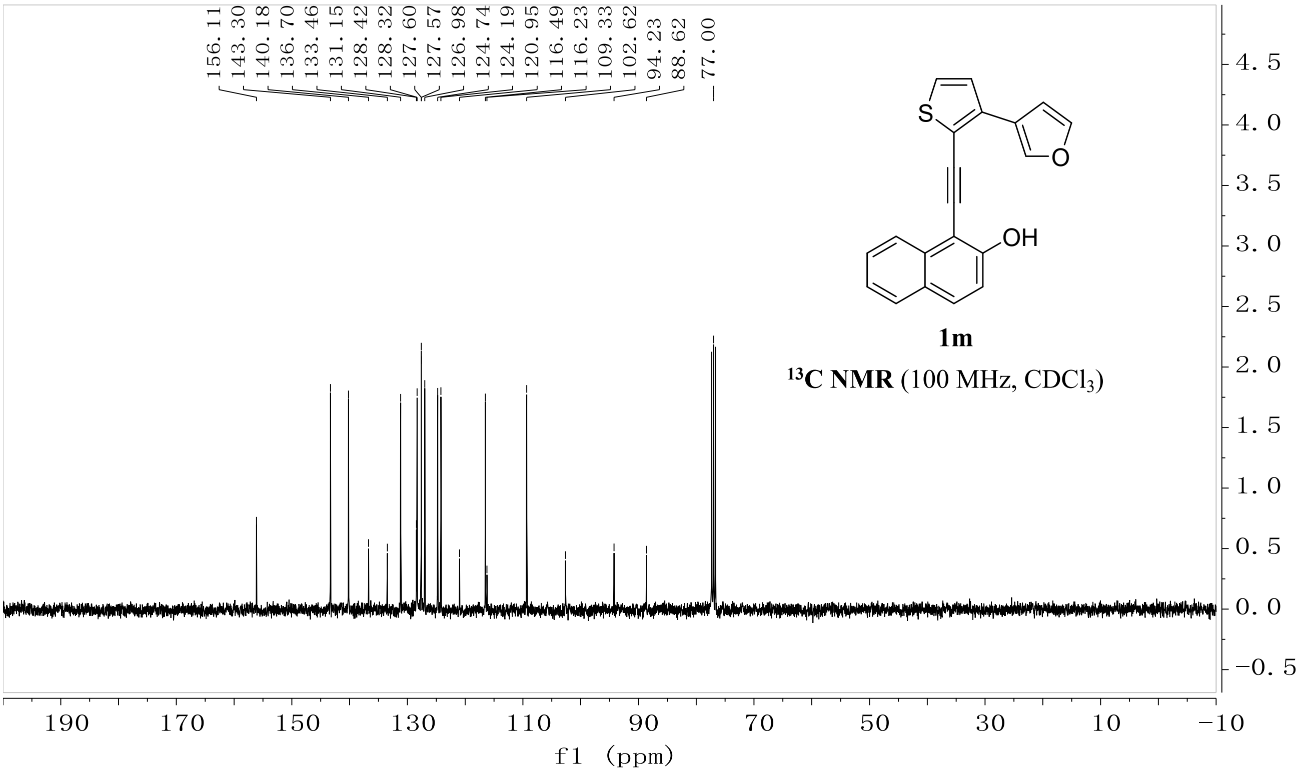

1m

^{13}C NMR (100 MHz, $CDCl_3$)

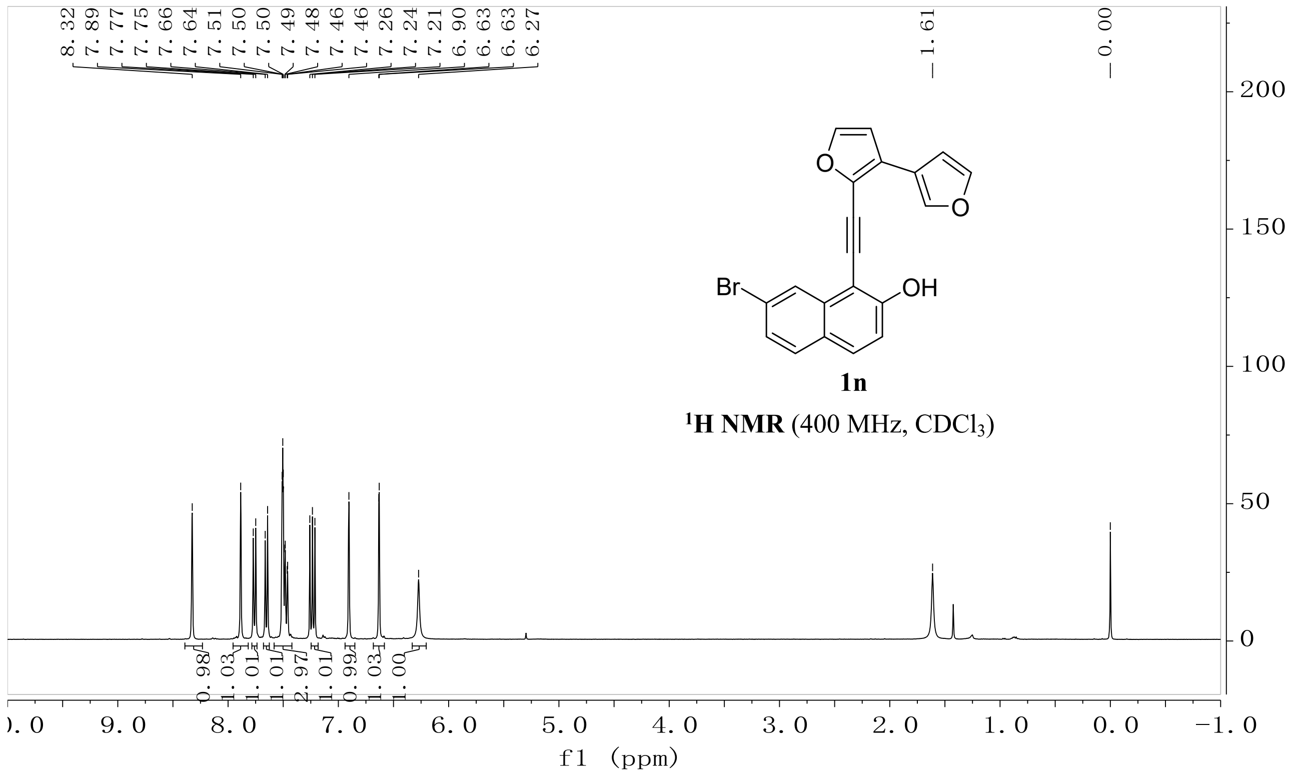

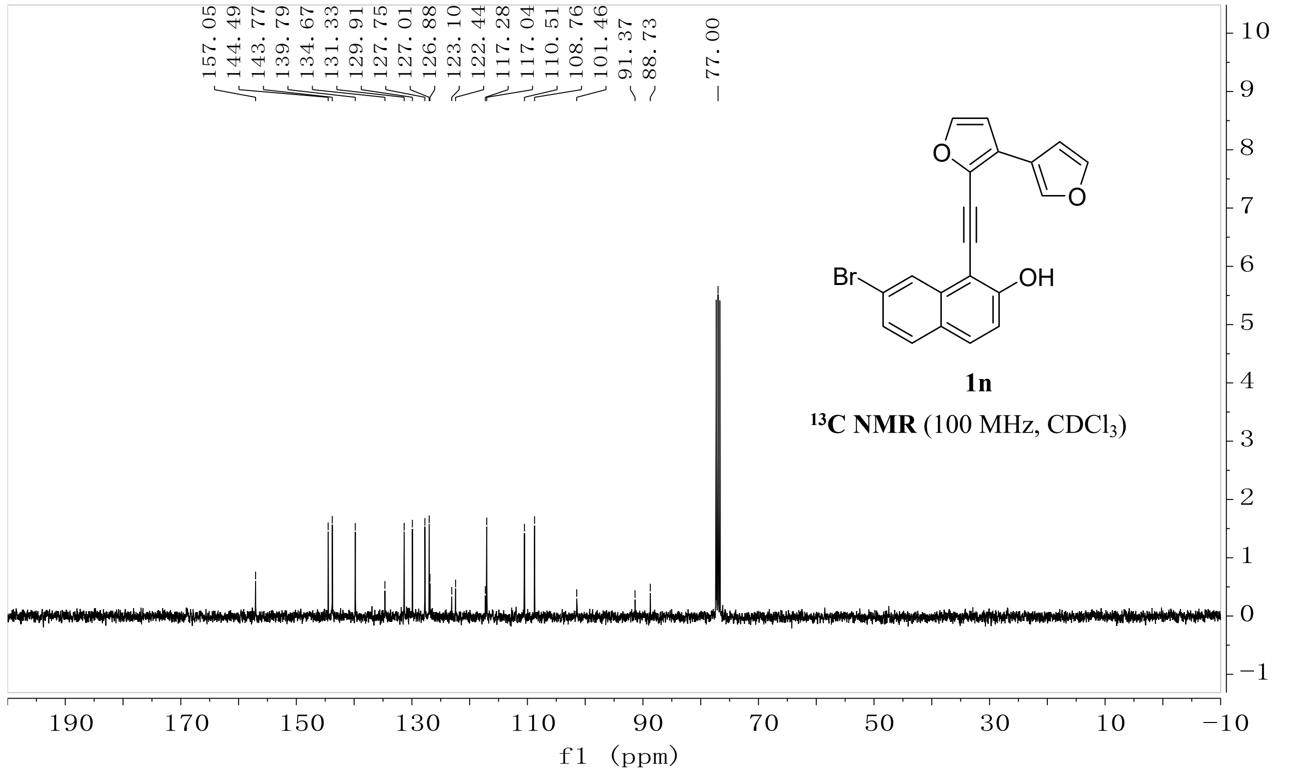

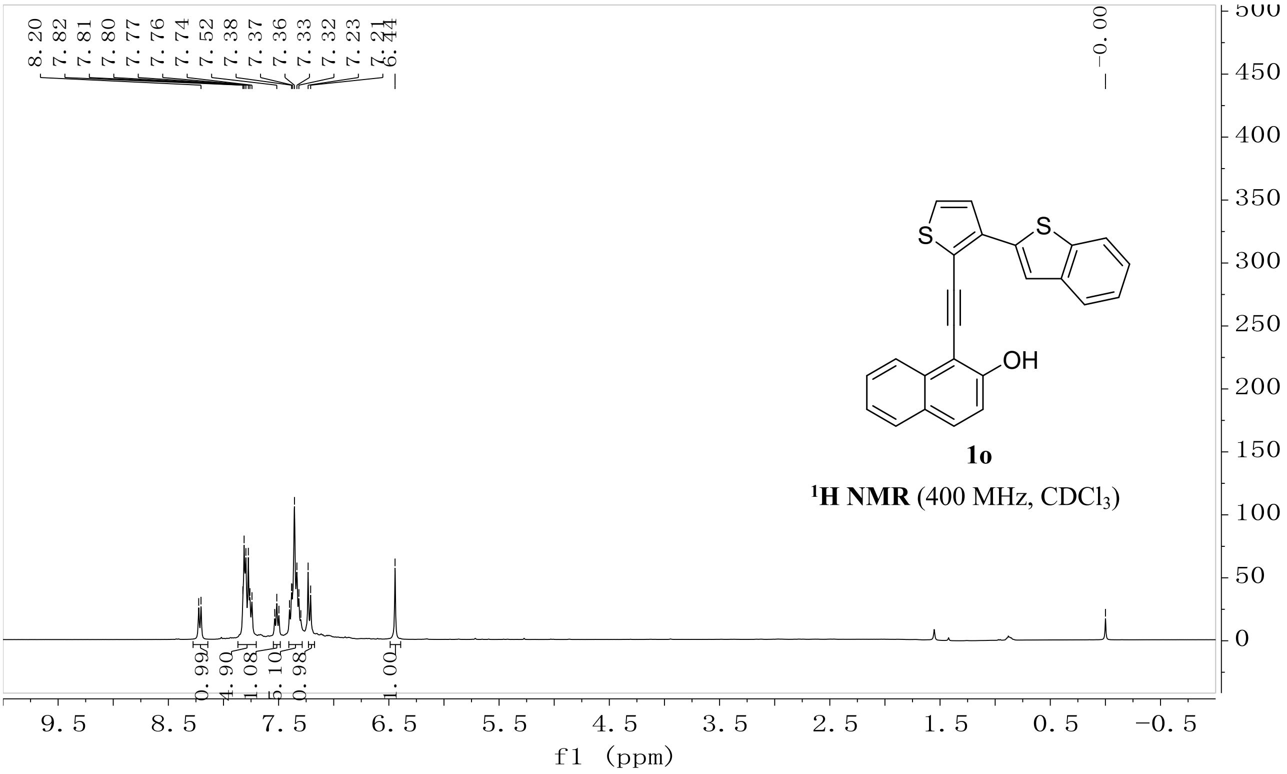

1o

^{1}H NMR (400 MHz, $CDCl_3$)

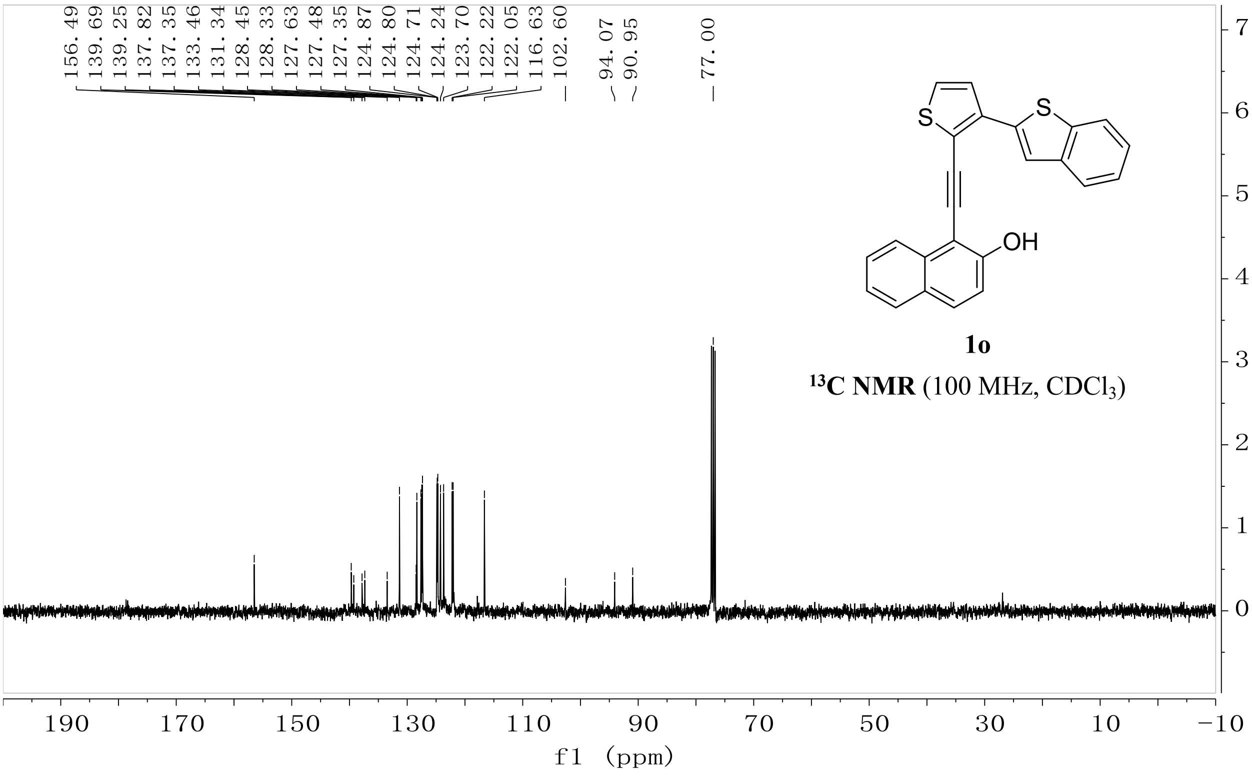

1o

^{13}C NMR (100 MHz, $CDCl_3$)

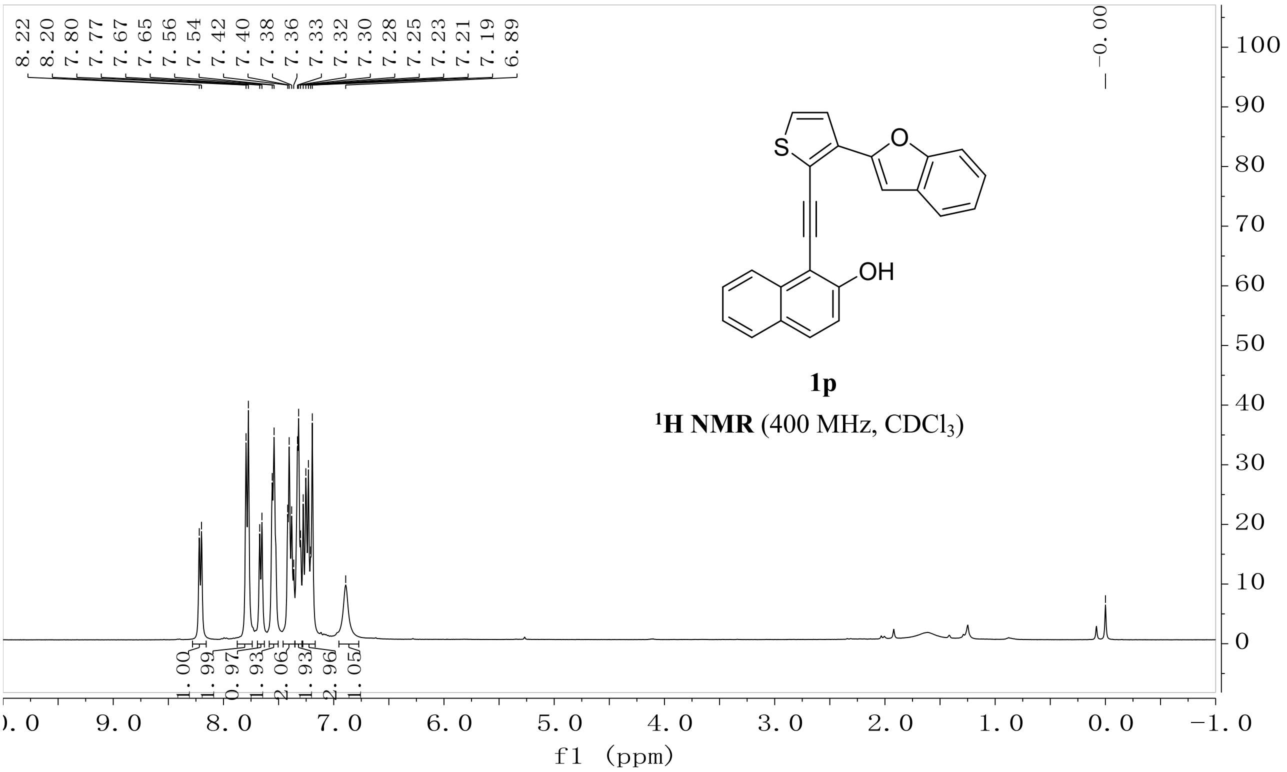
8.22
8.20
7.80
7.77
7.67
7.65
7.56
7.54
7.42
7.40
7.38
7.36
7.33
7.32
7.30
7.28
7.25
7.23
7.21
7.19
6.89
-0.00
S
O
OH
1p
1H NMR (400 MHz, CDCl3)
1.00
1.99
0.97
1.93
2.06
1.93
2.96
1.05
f1 (ppm)

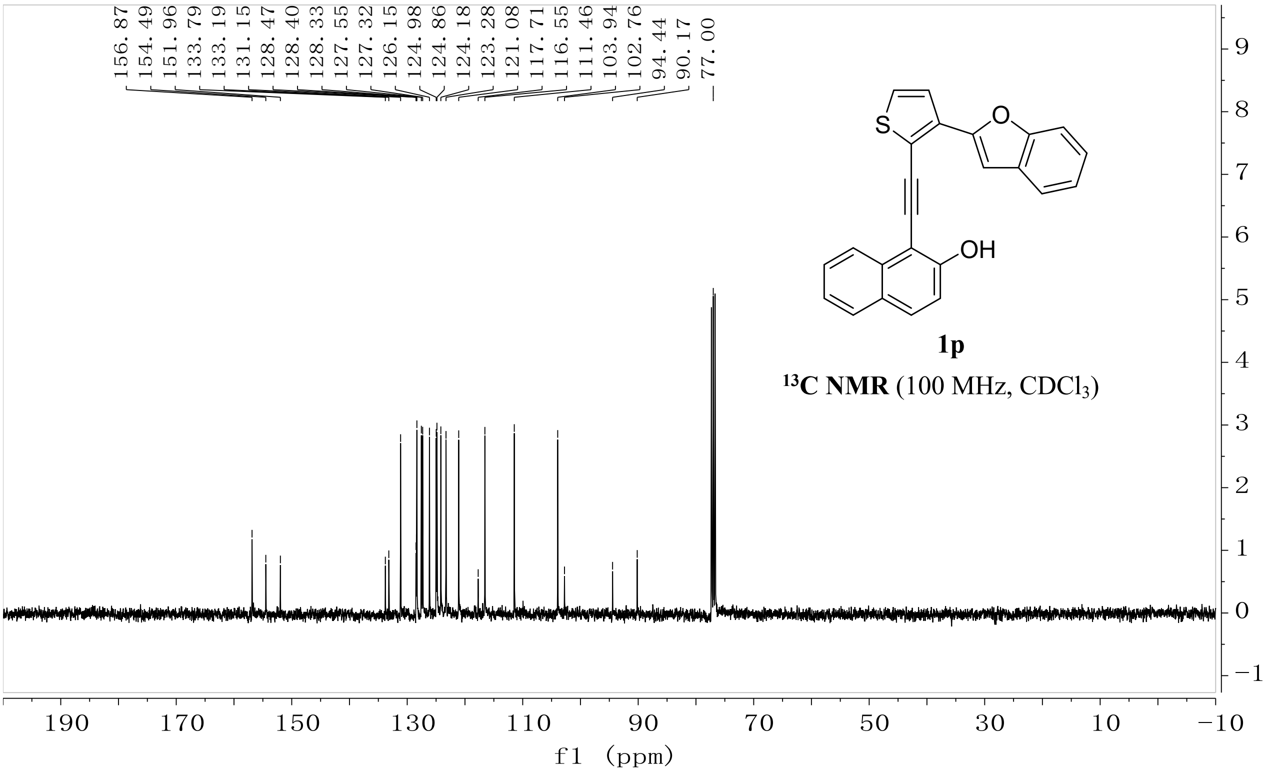
156.87
154.49
151.96
133.79
133.19
131.15
128.47
128.40
128.33
127.55
127.32
126.15
124.98
124.86
124.18
123.28
121.08
117.71
116.55
111.46
103.94
102.76
94.44
90.17
77.00
S
O
OH
1p
13C NMR (100 MHz, CDCl3)
f1 (ppm)

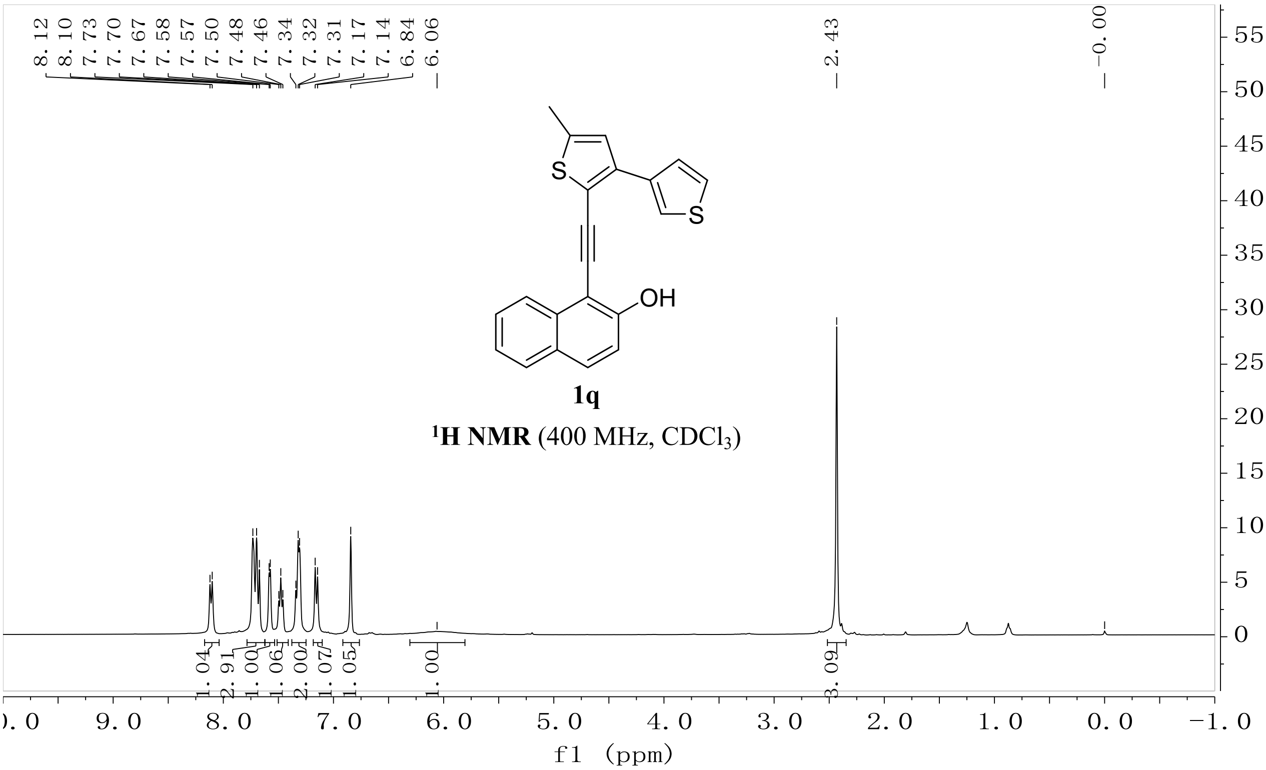

1q

^{1}H NMR (400 MHz, $CDCl_3$)

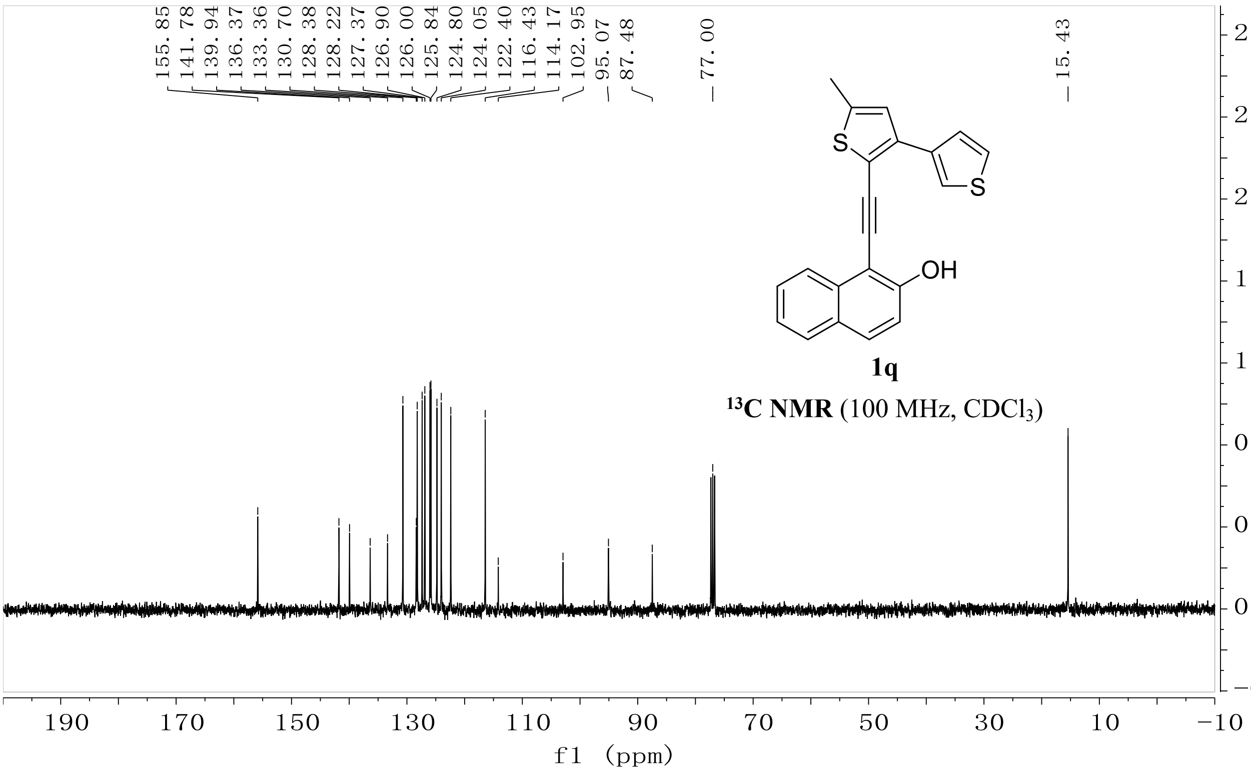

1q

^{13}C NMR (100 MHz, $CDCl_3$)

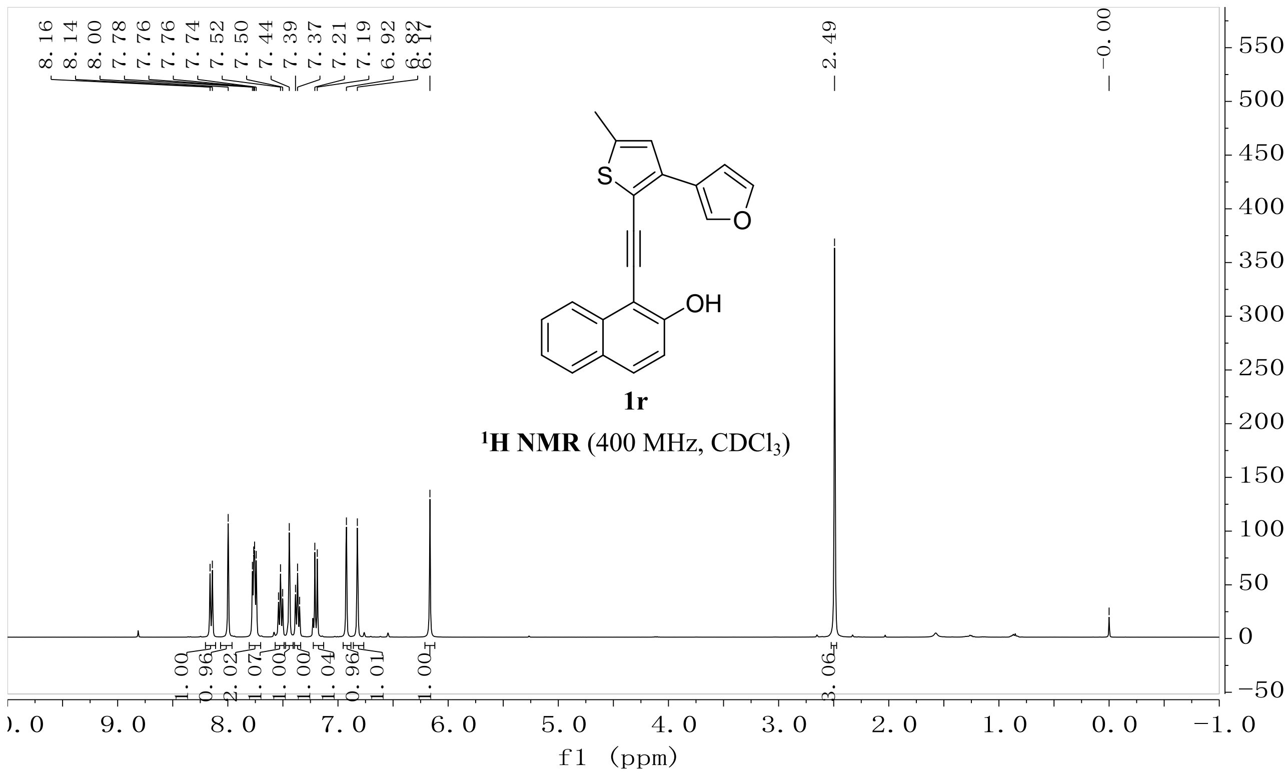
8.16 8.14 8.00 7.78 7.76 7.76 7.74 7.52 7.50 7.44 7.39 7.37 7.21 7.19 6.92 6.82 6.17
2.49
-0.00
S
O
OH
1r
^{1}H NMR (400 MHz, CDCl$_3$)
1.00 0.96 2.02 1.07 1.00 1.00 1.04 0.96 1.01 1.00
3.06
f1 (ppm)

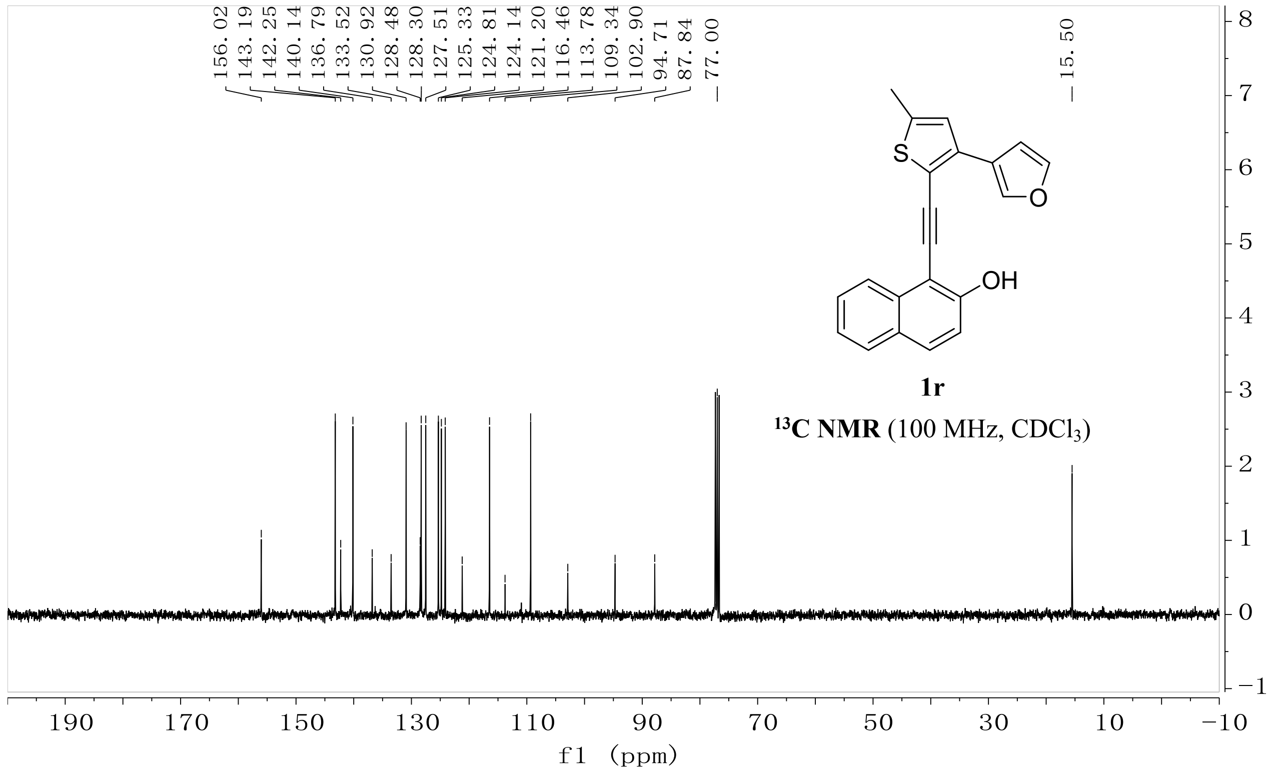
156.02 143.19 142.25 140.14 136.79 133.52 130.92 128.48 128.30 127.51 125.33 124.81 124.14 121.20 116.46 113.78 109.34 102.90 94.71 87.84 77.00
15.50
S
O
OH
1r
^{13}C NMR (100 MHz, CDCl$_3$)
f1 (ppm)

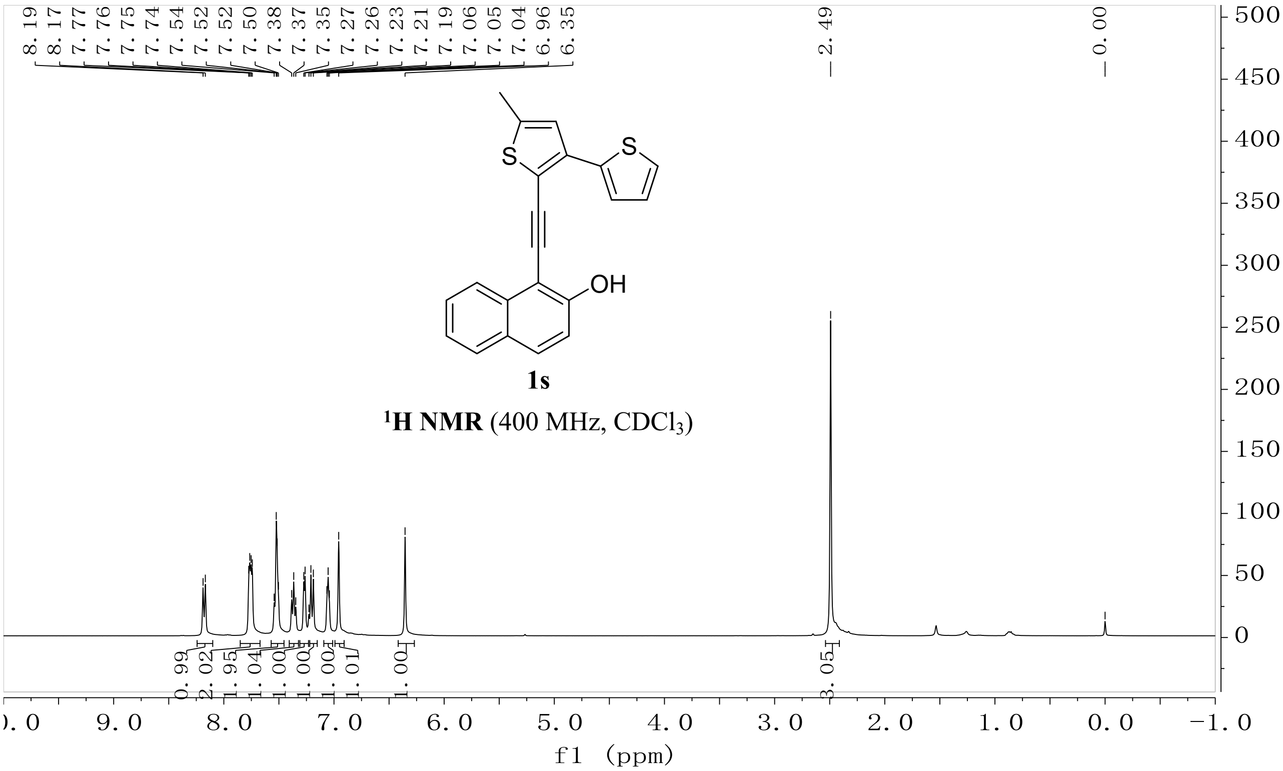
8.19 8.17 7.77 7.76 7.75 7.74 7.54 7.52 7.52 7.50 7.38 7.37 7.35 7.27 7.26 7.23 7.21 7.19 7.06 7.05 7.04 6.96 6.35
2.49
0.00
0.99 2.02 1.95 1.04 1.00 1.00 1.00 1.01 1.00 3.05
f1 (ppm)
OH
S
S
1s
^{1}H NMR (400 MHz, $CDCl_3$)

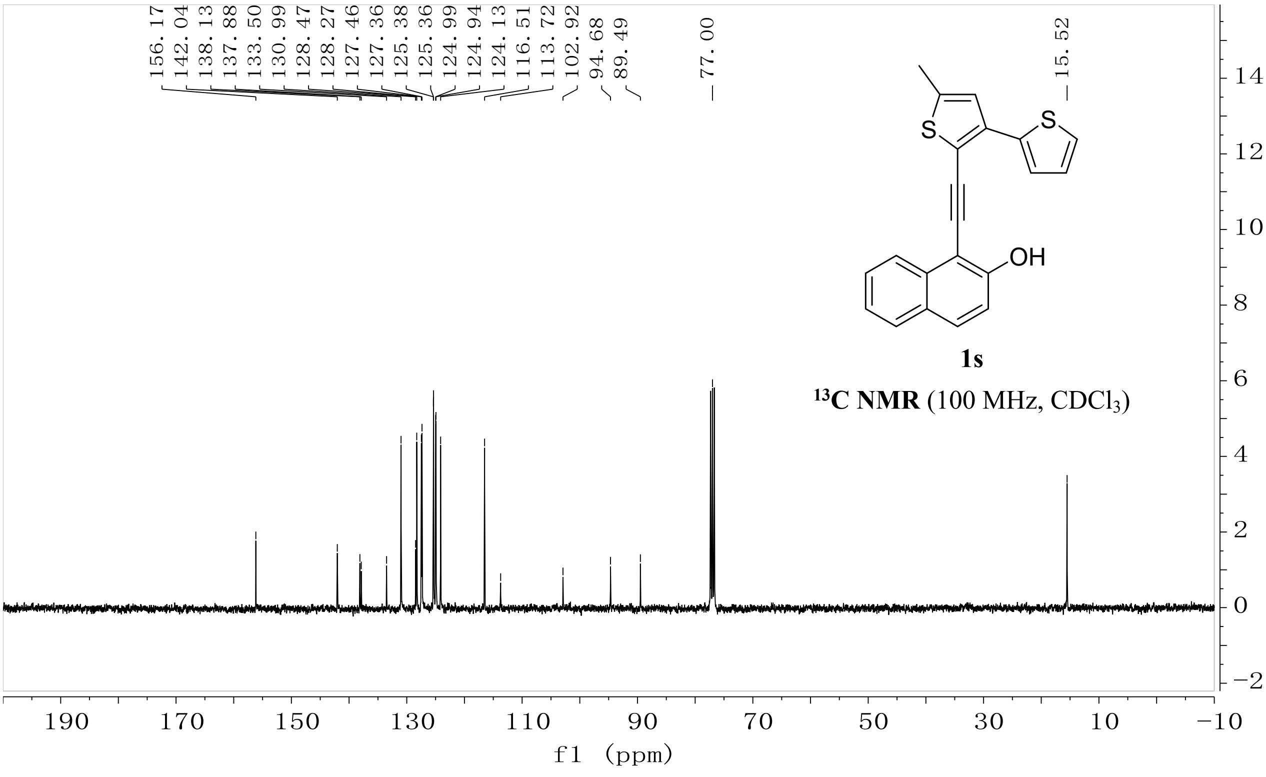
156.17 142.04 138.13 137.88 133.50 130.99 128.47 128.27 127.46 127.36 125.38 125.36 124.99 124.94 124.13 116.51 113.72 102.92 94.68 89.49
77.00
15.52
f1 (ppm)
OH
S
S
1s
^{13}C NMR (100 MHz, $CDCl_3$)

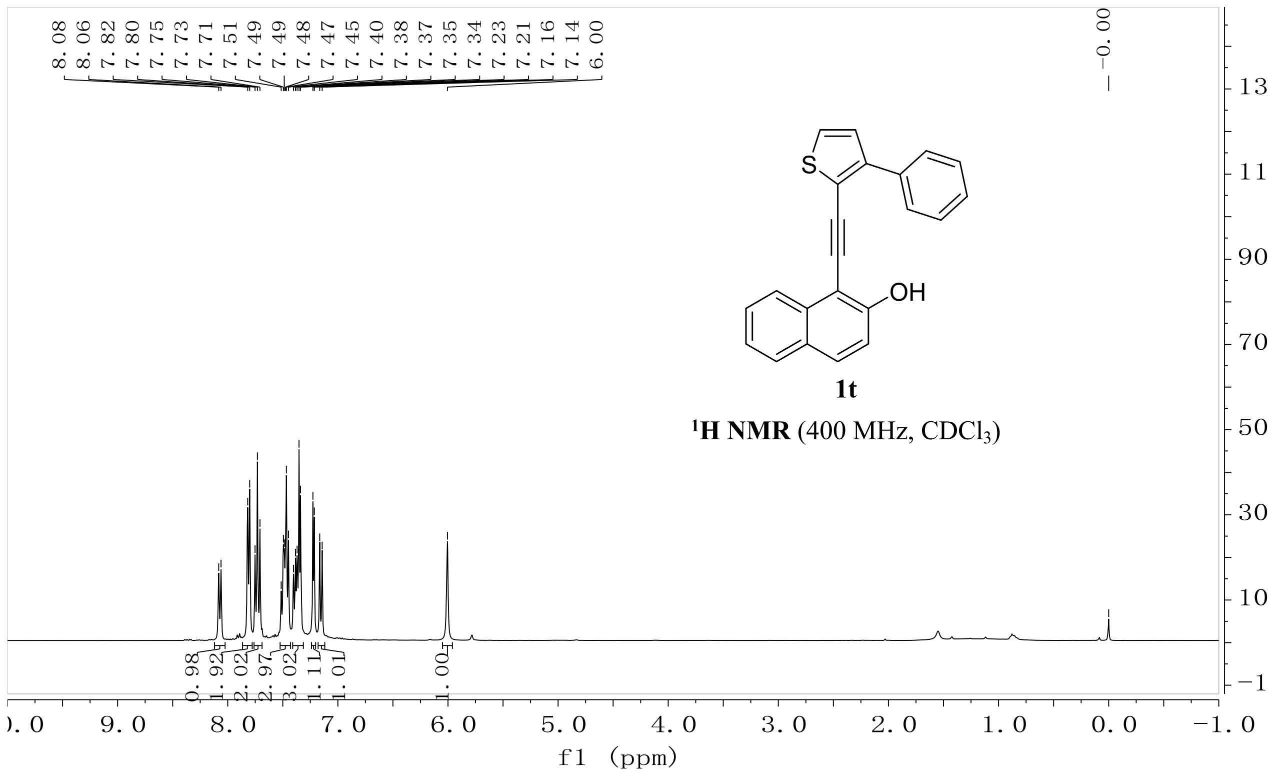
8.08 8.06 7.82 7.80 7.75 7.73 7.71 7.51 7.49 7.49 7.48 7.47 7.45 7.40 7.38 7.37 7.35 7.34 7.23 7.21 7.16 7.14 6.00
-0.00
OH
S
1t
1H NMR (400 MHz, CDCl3)
0.98 1.92 2.02 2.97 3.02 1.11 1.01 1.00
f1 (ppm)

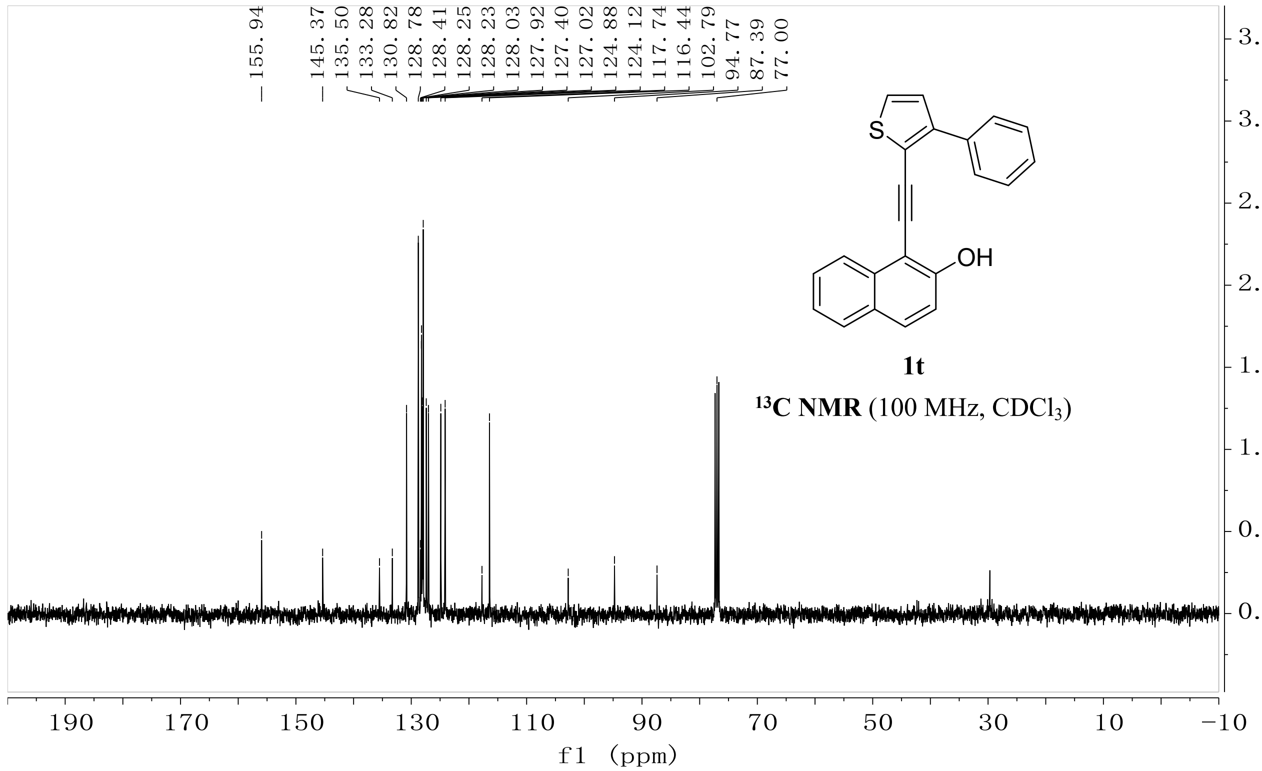
155.94
145.37 135.50 133.28 130.82 128.78 128.41 128.25 128.23 128.03 127.92 127.40 127.02 124.88 124.12 117.74 116.44 102.79 94.77 87.39 77.00
OH
S
1t
13C NMR (100 MHz, CDCl3)
f1 (ppm)

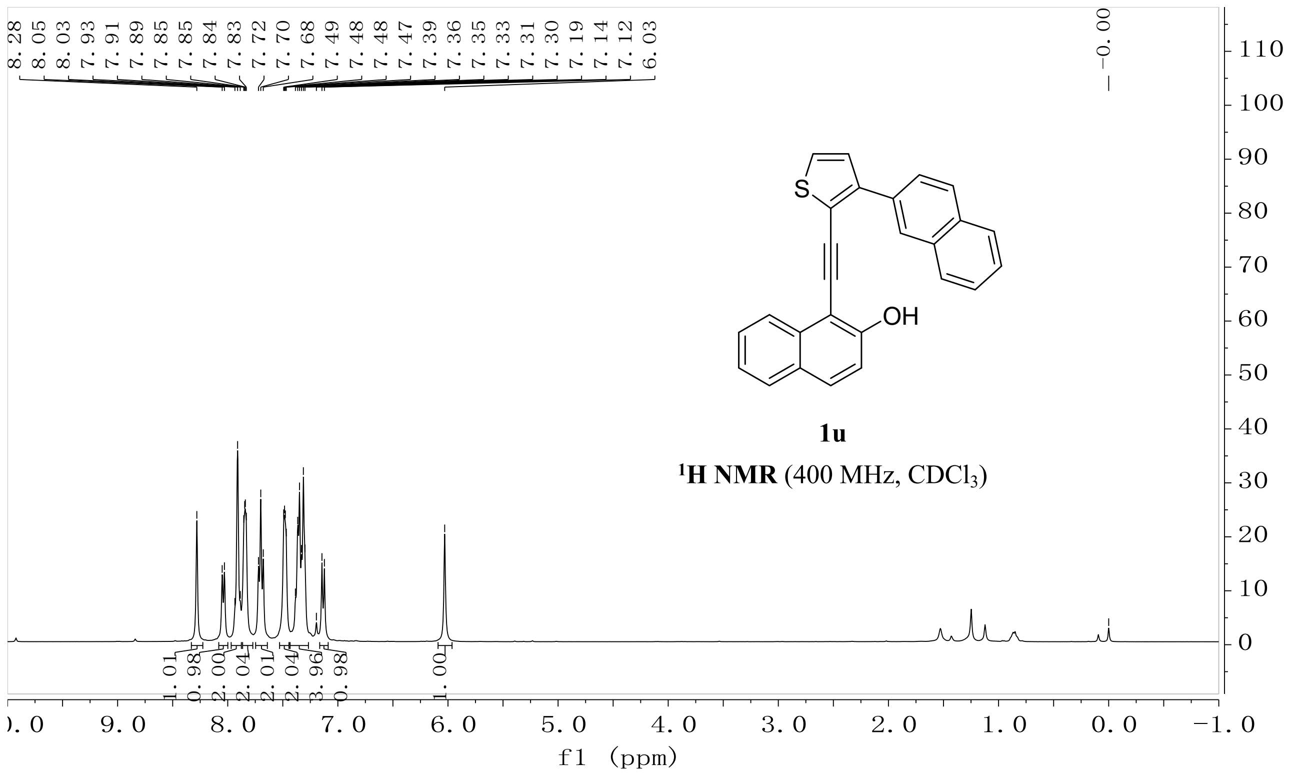
8.28
8.05
8.03
7.93
7.91
7.89
7.85
7.85
7.84
7.83
7.72
7.70
7.68
7.49
7.48
7.48
7.47
7.39
7.36
7.35
7.33
7.31
7.30
7.19
7.14
7.12
6.03
-0.00
S
OH
1u
¹H NMR (400 MHz, CDCl₃)
1.01
0.98
2.00
2.04
2.01
2.04
3.96
0.98
1.00
9.0
8.0
7.0
6.0
5.0
4.0
3.0
2.0
1.0
0.0
-1.0
f1 (ppm)

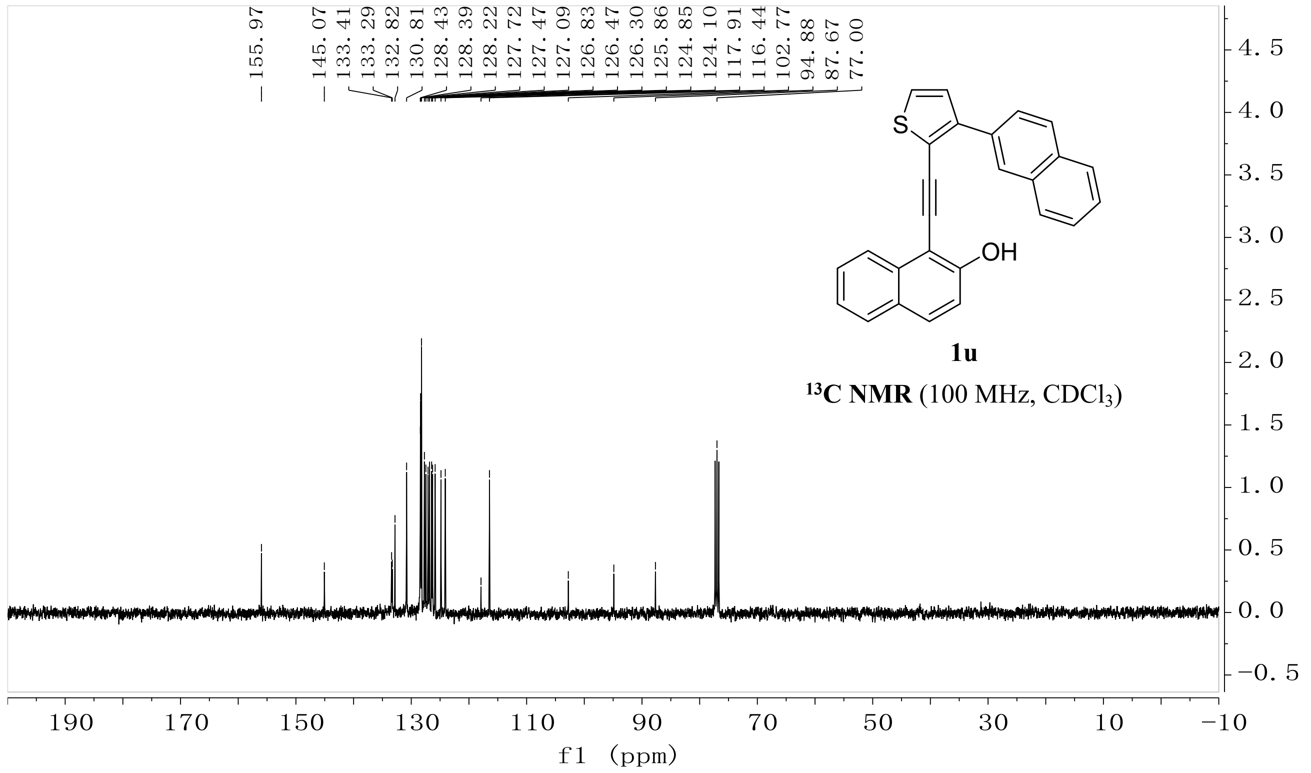
155.97
145.07
133.41
133.29
132.82
130.81
128.43
128.39
128.22
127.72
127.47
127.09
126.83
126.47
126.30
125.86
124.85
124.10
117.91
116.44
102.77
94.88
87.67
77.00
S
OH
1u
¹³C NMR (100 MHz, CDCl₃)
190
170
150
130
110
90
70
50
30
10
-10
f1 (ppm)

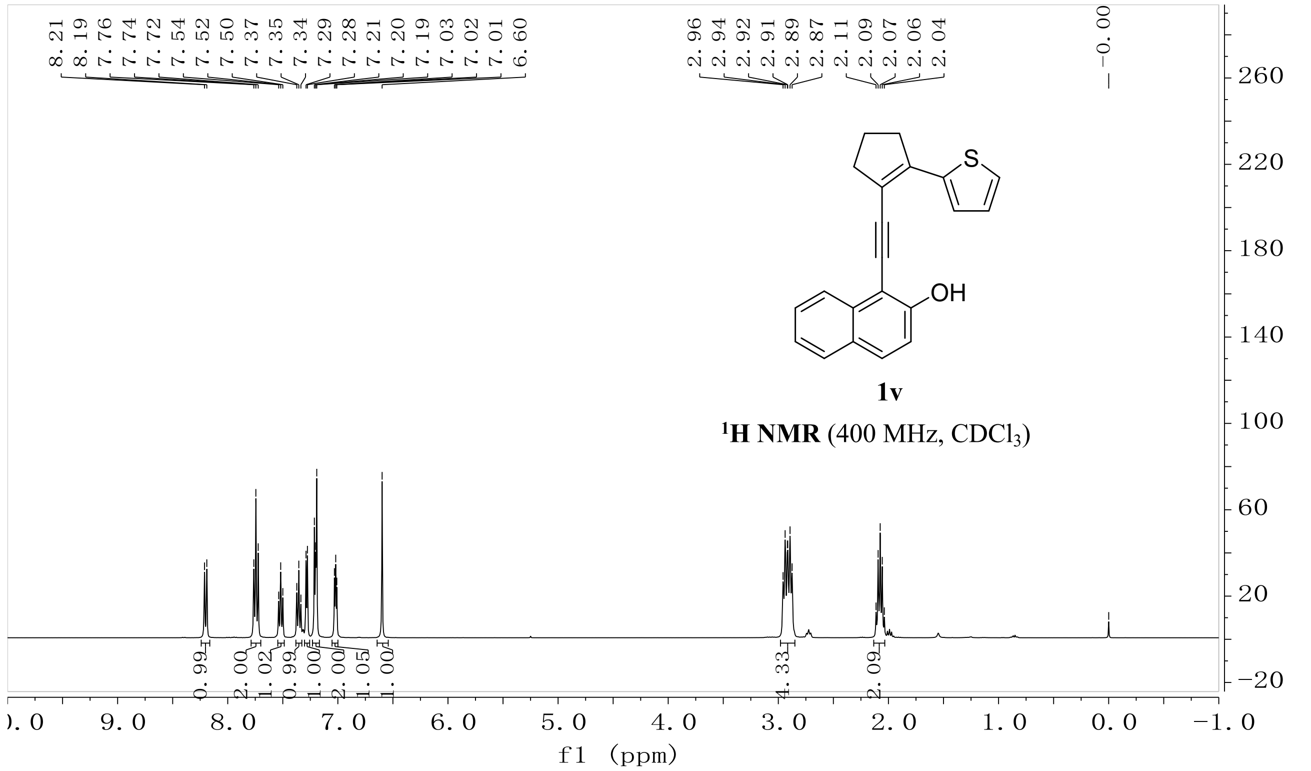

8.21 8.19 7.76 7.74 7.72 7.54 7.52 7.50 7.37 7.35 7.34 7.29 7.28 7.21 7.20 7.19 7.03 7.02 7.01 6.60
2.96 2.94 2.92 2.91 2.89 2.87 2.11 2.09 2.07 2.06 2.04
−0.00
S
OH
1v
1H NMR (400 MHz, CDCl3)
0.99 2.00 1.02 0.99 1.00 2.00 1.05 1.00
4.33 2.09
f1 (ppm)

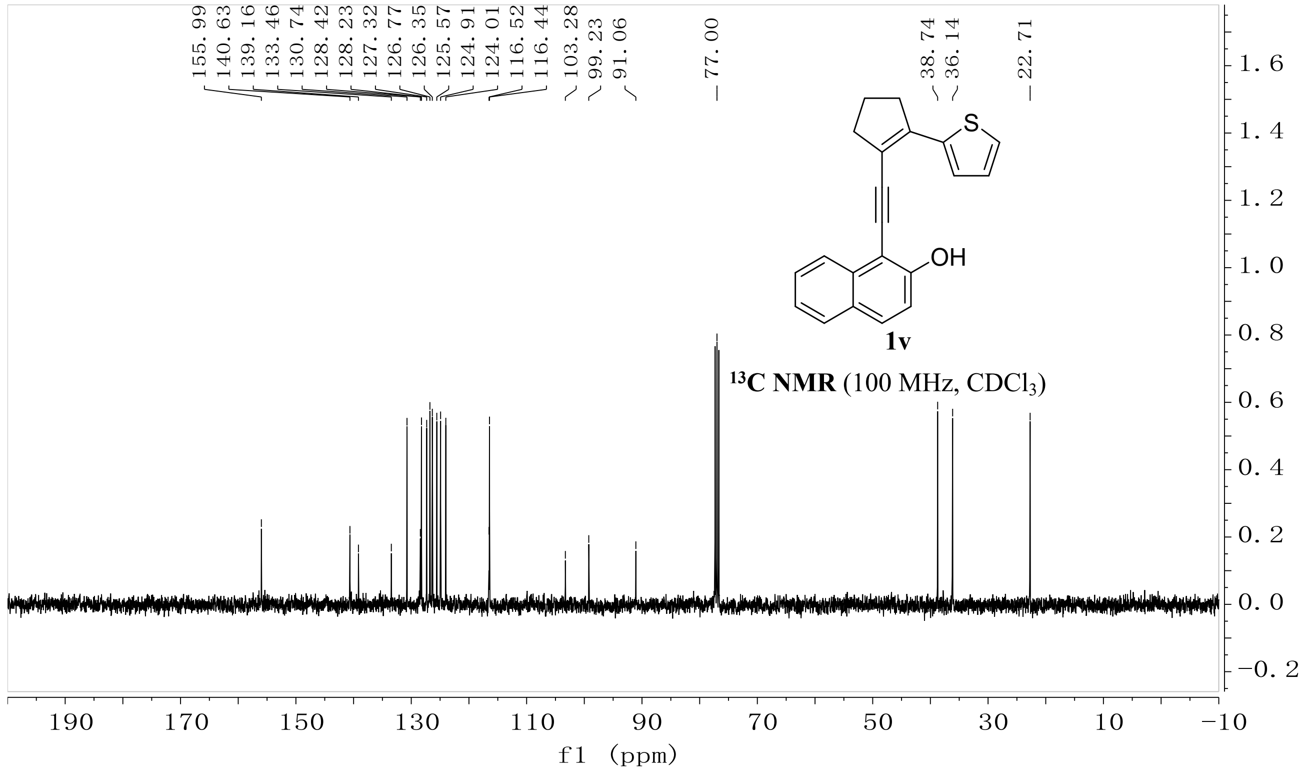

155.99 140.63 139.16 133.46 130.74 128.42 128.23 127.32 126.77 126.35 125.57 124.91 124.01 116.52 116.44 103.28 99.23 91.06
77.00
38.74 36.14
22.71
S
OH
1v
13C NMR (100 MHz, CDCl3)
f1 (ppm)

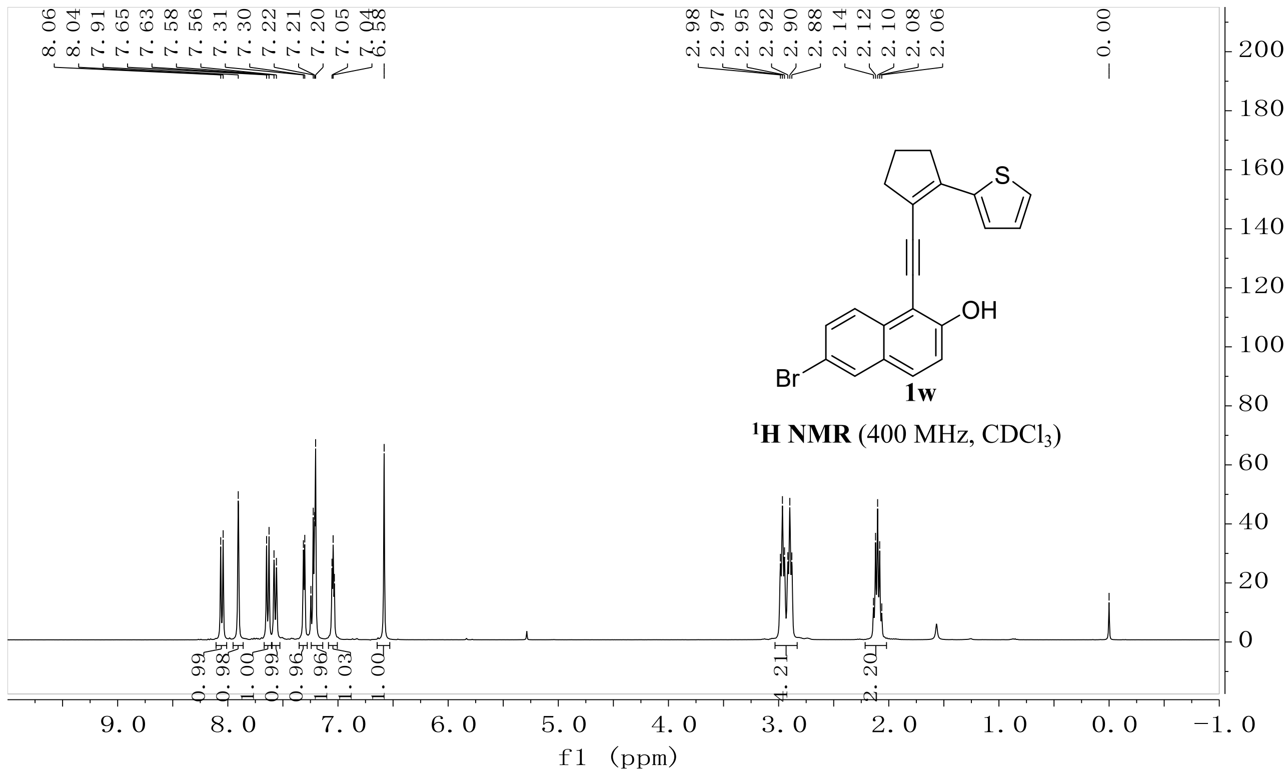
8.06 8.04 7.91 7.65 7.63 7.58 7.56 7.31 7.30 7.22 7.21 7.20 7.05 7.04 6.58
2.98 2.97 2.95 2.92 2.90 2.88 2.14 2.12 2.10 2.08 2.06
0.00
S
OH
Br
1w
1H NMR (400 MHz, CDCl3)
0.99 0.98 1.00 0.99 0.96 1.96 1.03 1.00 4.21 2.20
f1 (ppm)

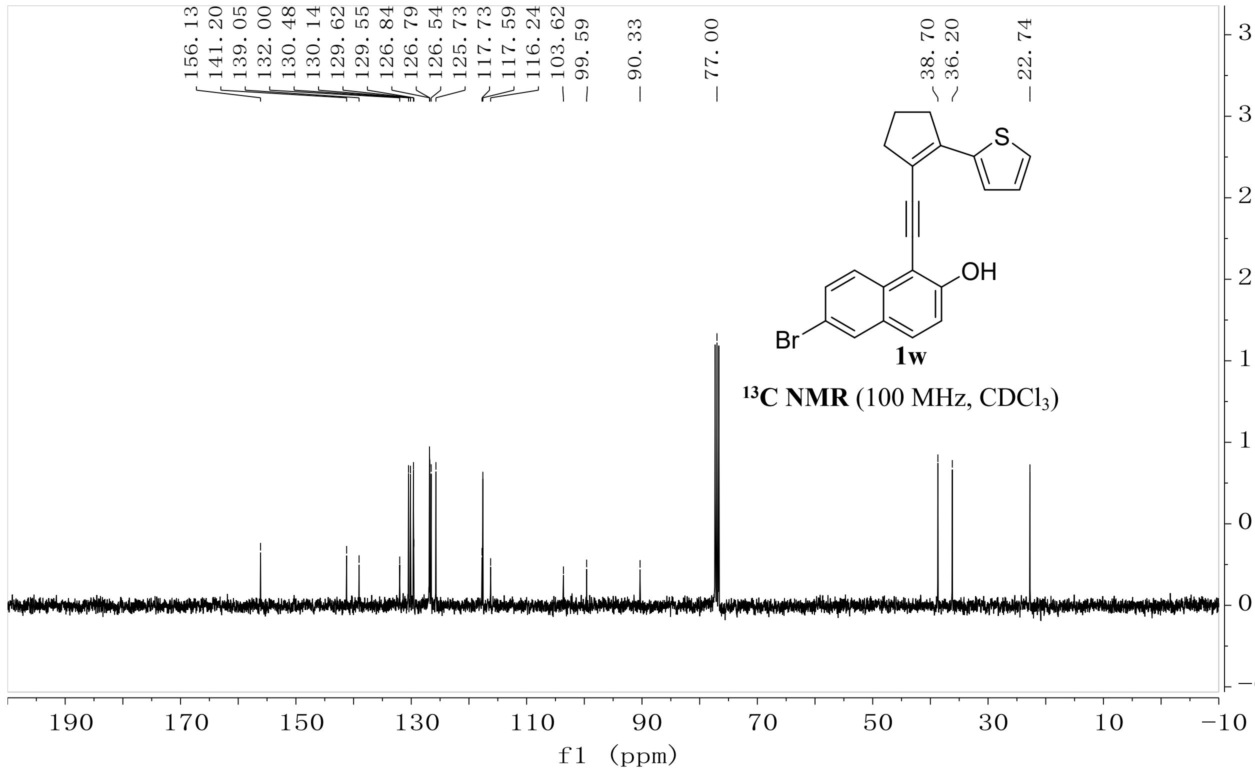
156.13 141.20 139.05 132.00 130.48 130.14 129.62 129.55 126.84 126.79 126.54 125.73 117.73 117.59 116.24 103.62 99.59 90.33 77.00 38.70 36.20 22.74
S
OH
Br
1w
13C NMR (100 MHz, CDCl3)
f1 (ppm)

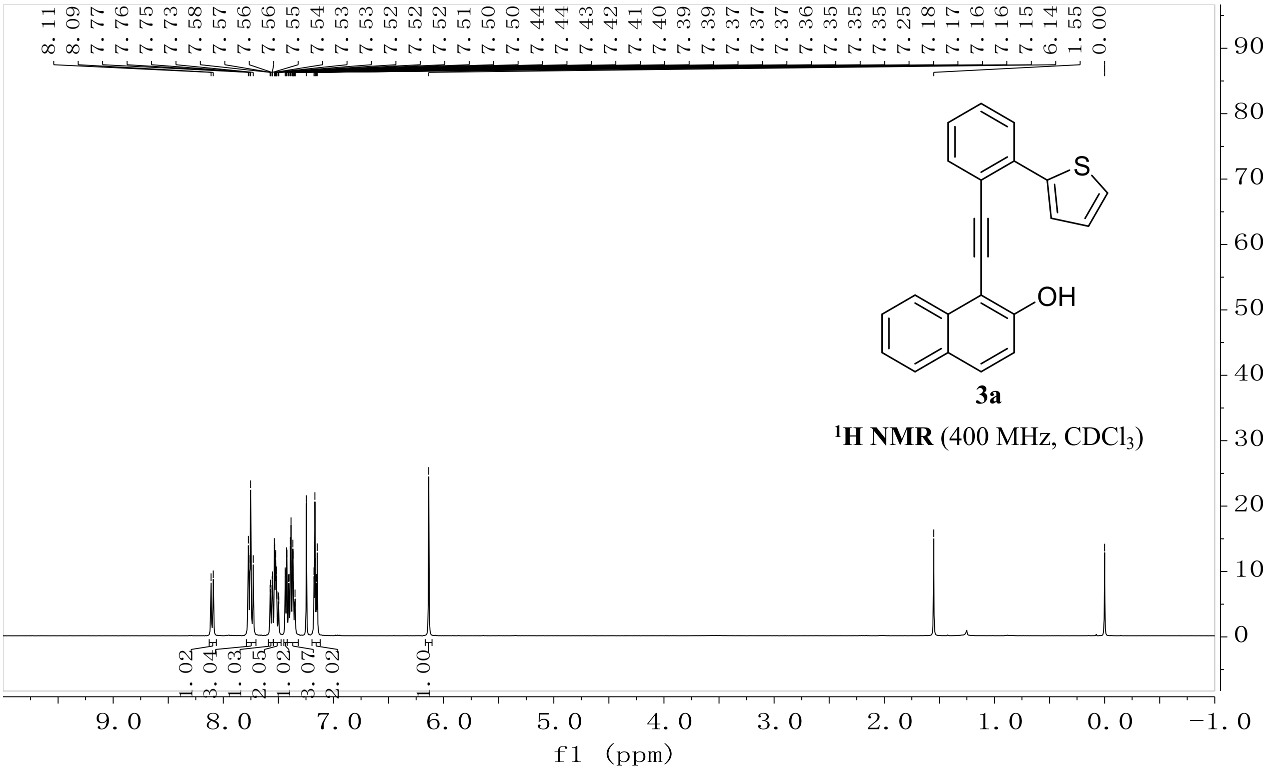
8.11 8.09 7.77 7.76 7.75 7.73 7.58 7.57 7.56 7.56 7.55 7.54 7.53 7.53 7.52 7.52 7.52 7.51 7.50 7.50 7.44 7.44 7.43 7.42 7.41 7.40 7.39 7.39 7.37 7.37 7.37 7.36 7.35 7.35 7.35 7.25 7.18 7.17 7.16 7.16 7.15 6.14 1.55 0.00
OH
S
3a
^{1}H NMR (400 MHz, CDCl$_3$)
1.02 3.04 1.03 2.05 1.02 3.07 2.02 1.00
f1 (ppm)

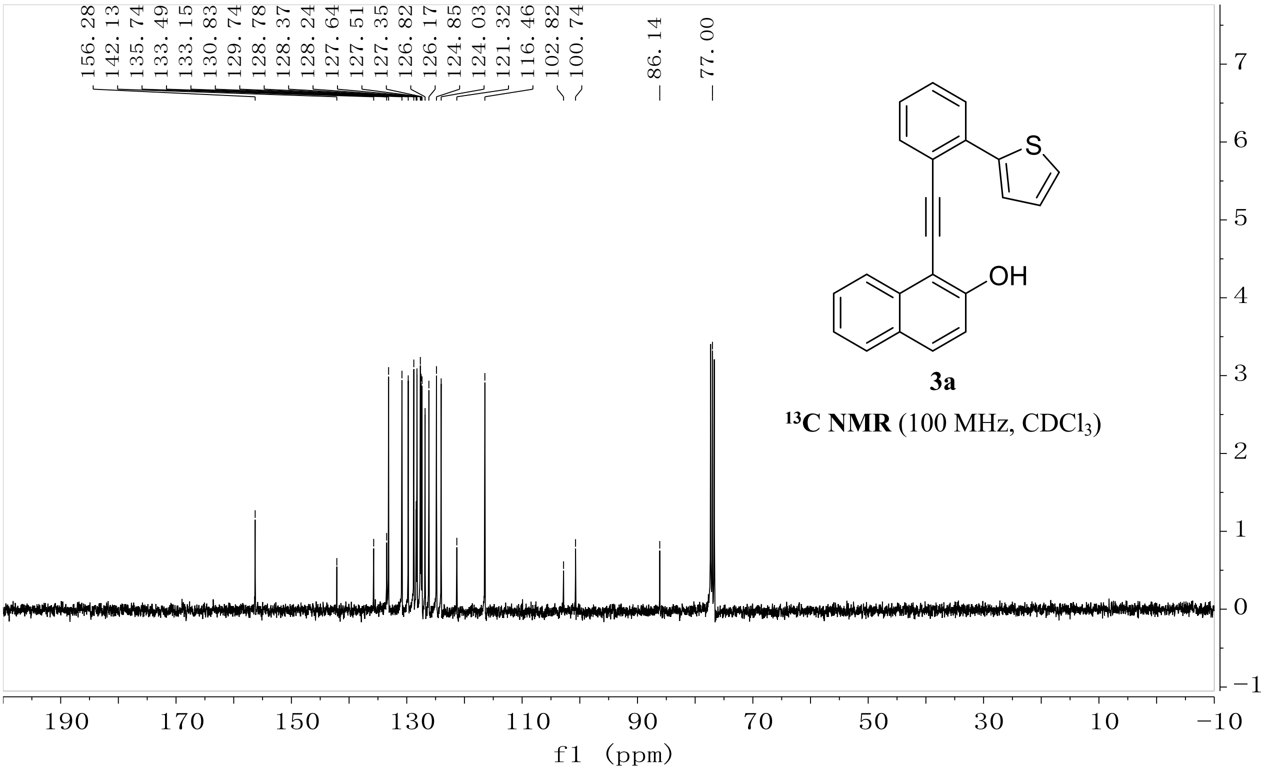
156.28 142.13 135.74 133.49 133.15 130.83 129.74 128.78 128.37 128.24 127.64 127.51 127.35 126.82 126.17 124.85 124.03 121.32 116.46 102.82 100.74 86.14 77.00
OH
S
3a
^{13}C NMR (100 MHz, CDCl$_3$)
f1 (ppm)

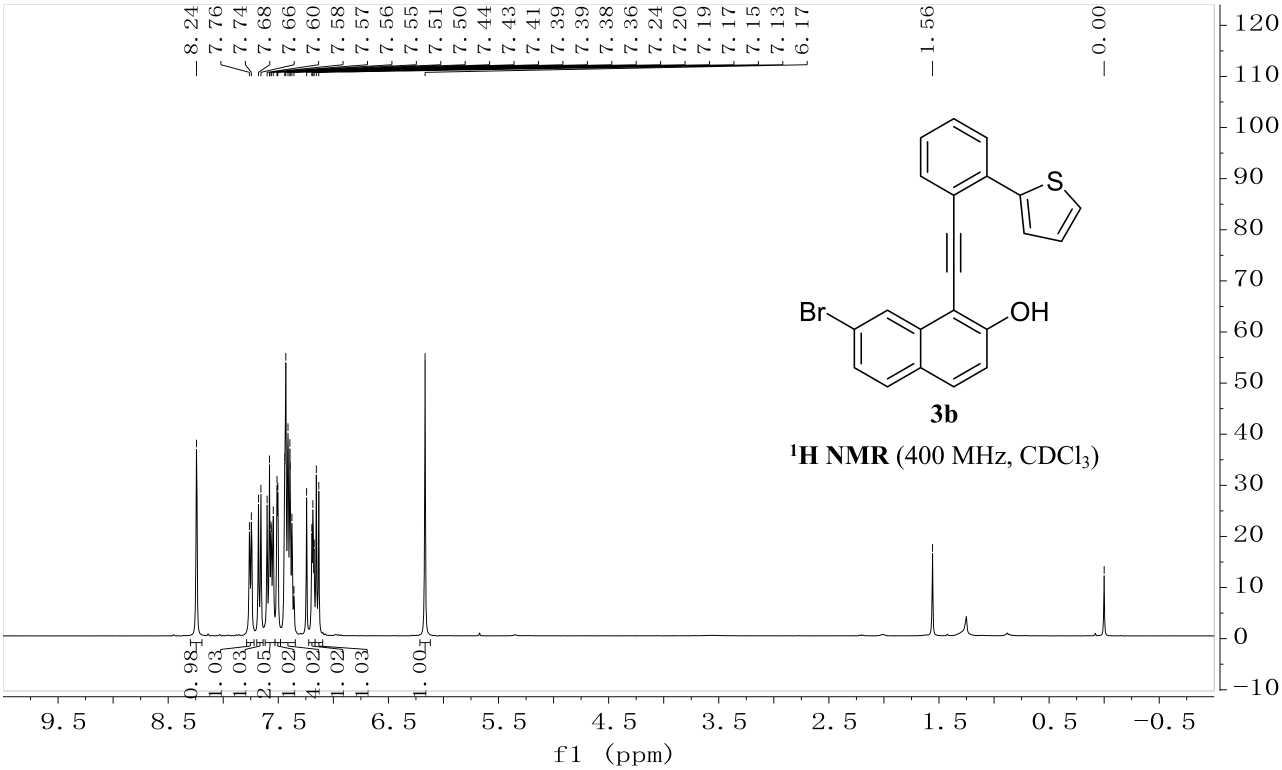
8.24
7.76
7.74
7.68
7.66
7.60
7.58
7.57
7.56
7.55
7.51
7.50
7.44
7.43
7.41
7.39
7.39
7.38
7.36
7.24
7.20
7.19
7.17
7.17
7.15
7.13
6.17
1.56
0.00
Br
OH
S
3b
^{1}H NMR (400 MHz, CDCl$_3$)
0.98
1.03
1.03
2.05
1.02
4.02
1.02
1.03
1.00
f1 (ppm)

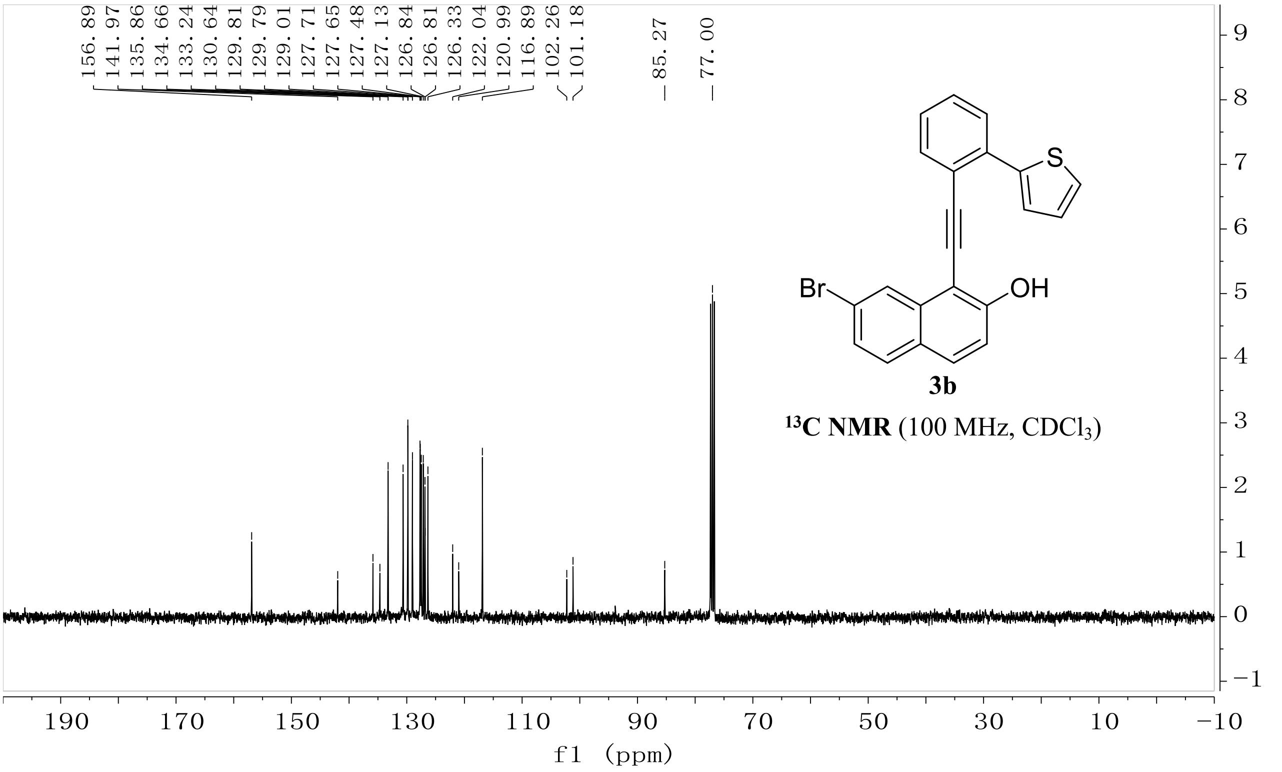
156.89
141.97
135.86
134.66
133.24
130.64
129.81
129.79
129.01
127.71
127.65
127.48
127.13
126.84
126.81
126.33
122.04
120.99
116.89
102.26
101.18
85.27
77.00
Br
OH
S
3b
^{13}C NMR (100 MHz, CDCl$_3$)
f1 (ppm)

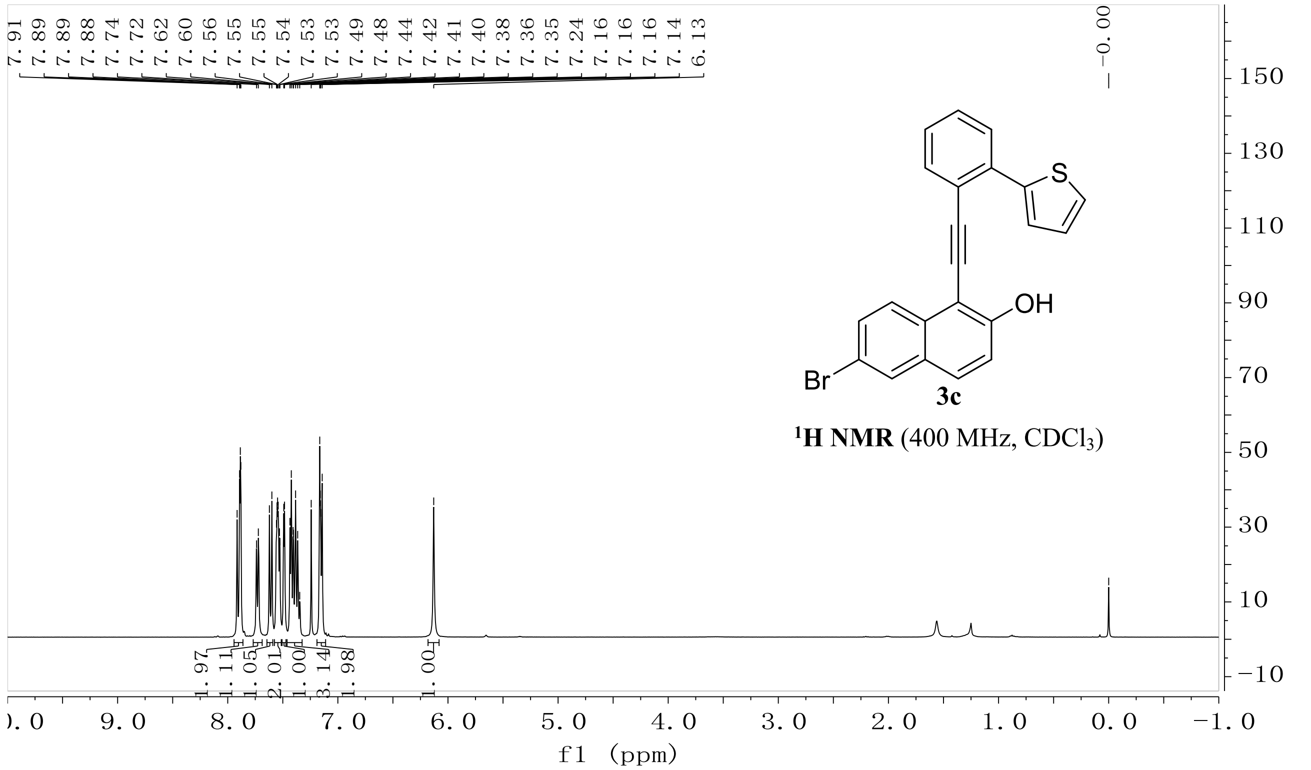
7.91
7.89
7.89
7.88
7.74
7.72
7.62
7.60
7.56
7.55
7.55
7.54
7.53
7.53
7.49
7.48
7.44
7.42
7.41
7.40
7.38
7.36
7.35
7.24
7.16
7.16
7.16
7.14
6.13
-0.00
OH
Br
S
3c
1H NMR (400 MHz, CDCl3)
1.97
1.11
1.05
2.01
1.00
3.14
1.98
1.00
f1 (ppm)

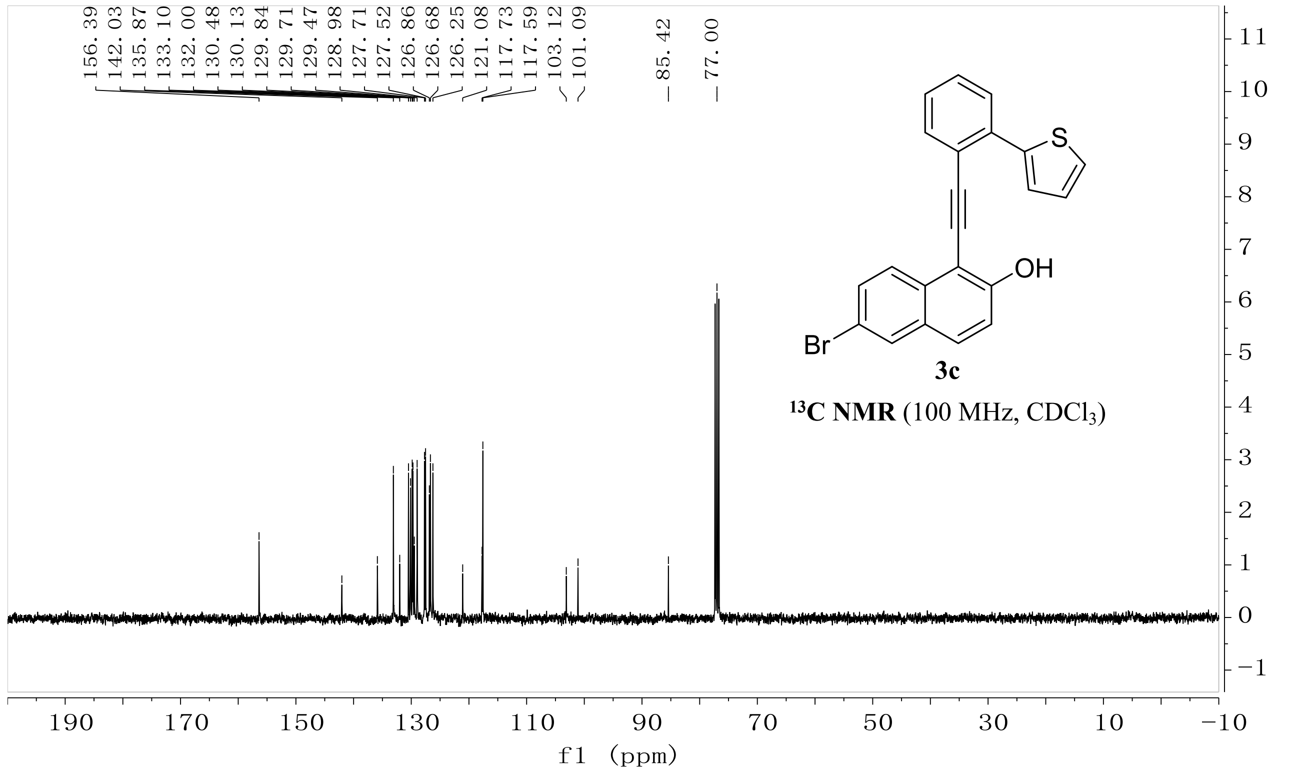
156.39
142.03
135.87
133.10
132.00
130.48
130.13
129.84
129.71
129.47
128.98
127.71
127.52
126.86
126.68
126.25
121.08
117.73
117.59
103.12
101.09
85.42
77.00
OH
Br
S
3c
13C NMR (100 MHz, CDCl3)
f1 (ppm)

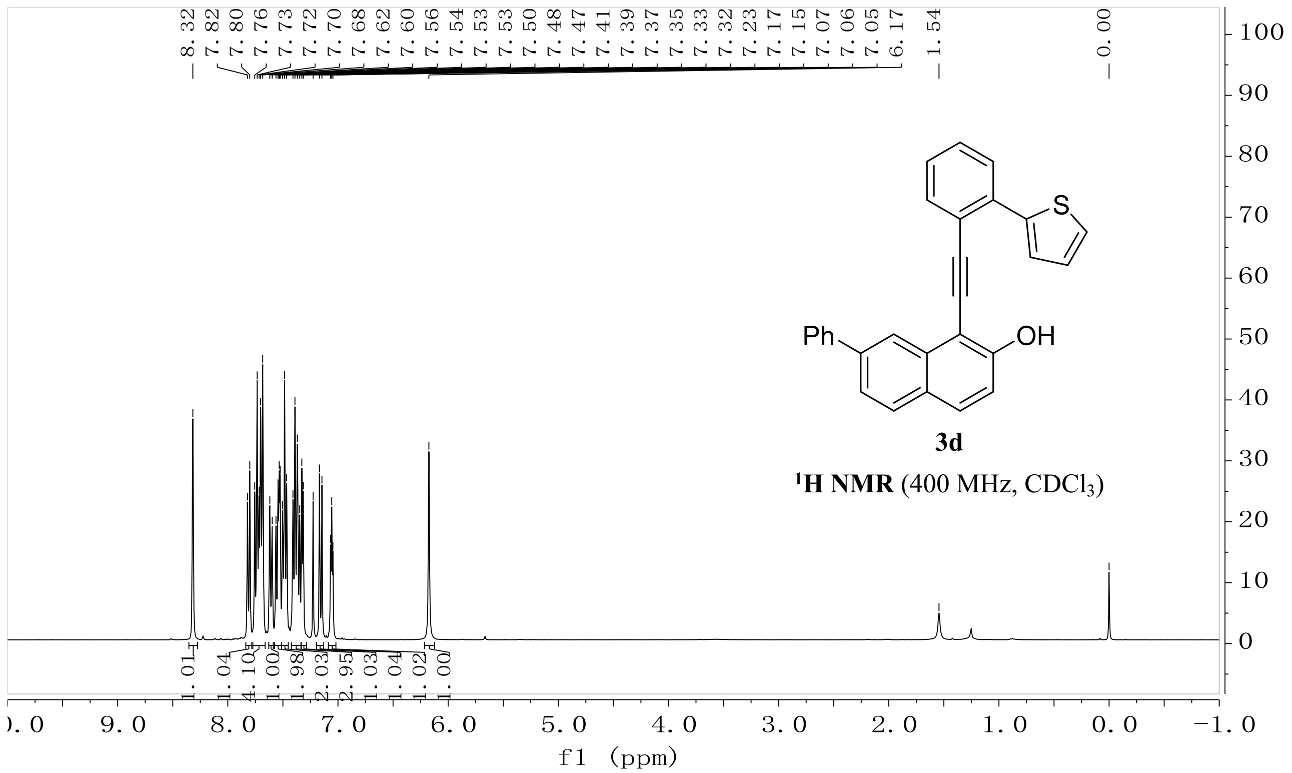

3d

^{1}H NMR (400 MHz, $CDCl_3$)

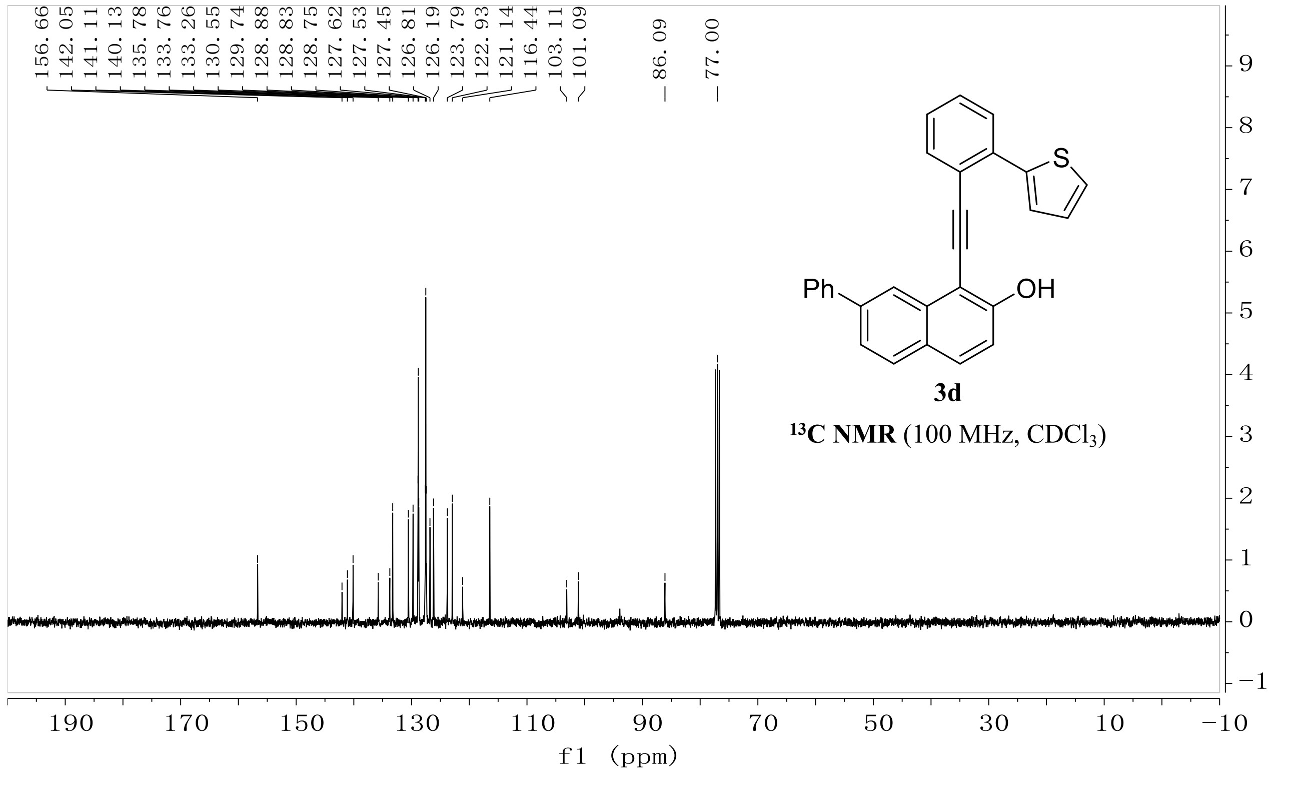

3d

^{13}C NMR (100 MHz, $CDCl_3$)

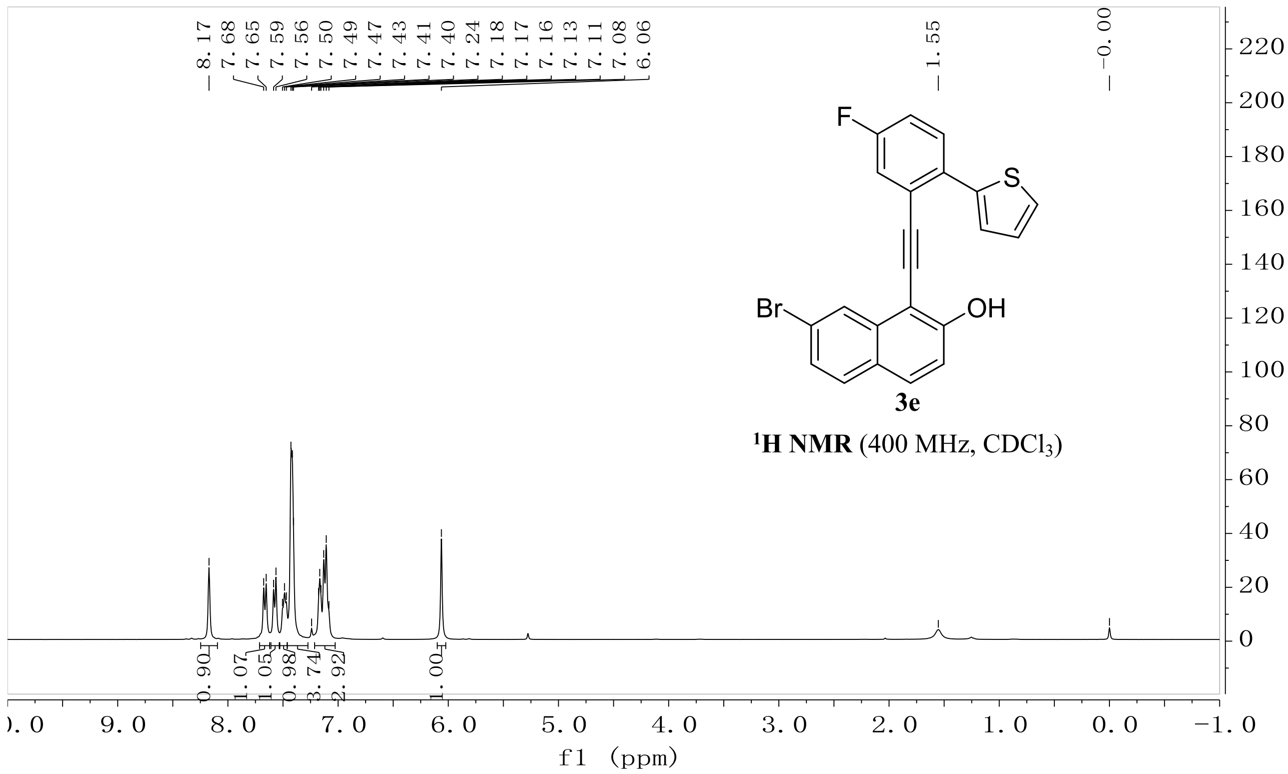
8.17
7.68
7.65
7.59
7.56
7.50
7.49
7.47
7.43
7.41
7.40
7.24
7.18
7.17
7.16
7.13
7.11
7.08
6.06
1.55
0.00
F
S
Br
OH
3e
¹H NMR (400 MHz, CDCl₃)
0.90
1.07
1.05
0.98
3.74
2.92
1.00
f1 (ppm)

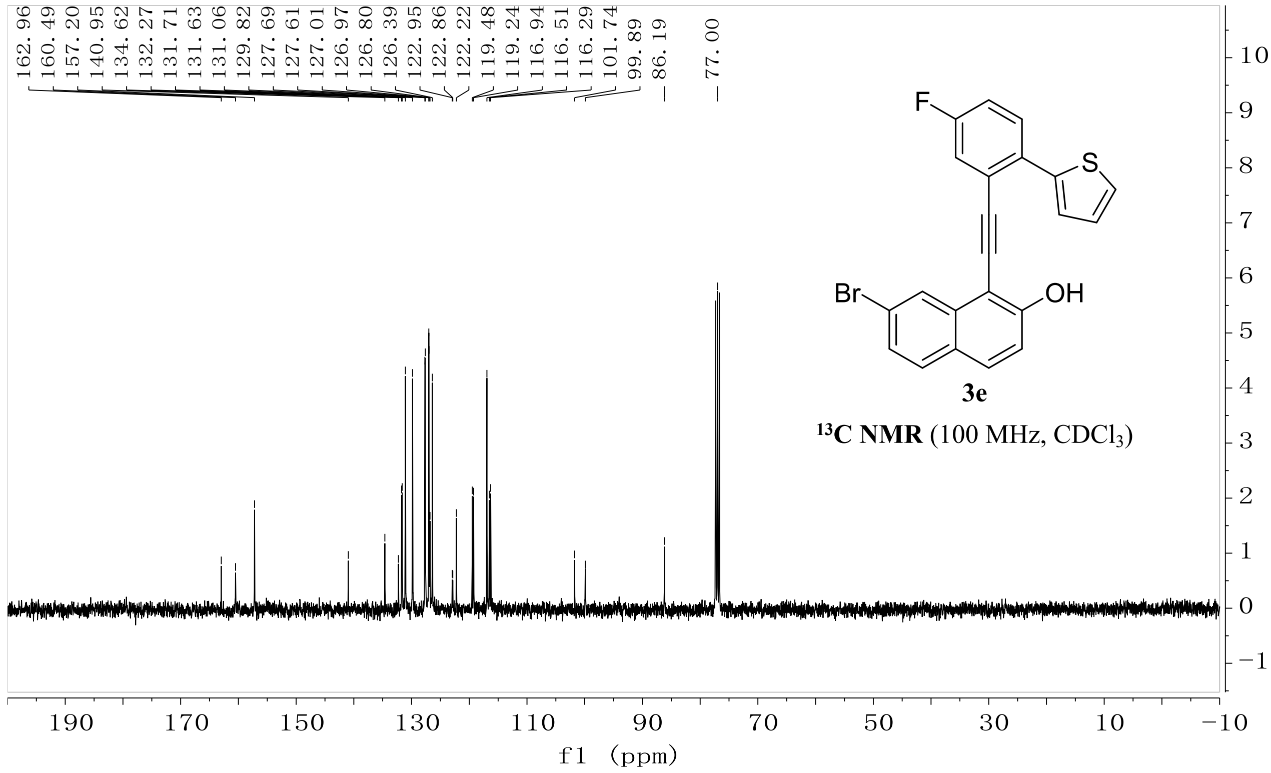
162.96
160.49
157.20
140.95
134.62
132.27
131.71
131.63
131.06
129.82
127.69
127.61
127.01
126.97
126.80
126.39
122.95
122.86
122.22
119.48
119.24
116.94
116.51
116.29
101.74
99.89
86.19
77.00
F
S
Br
OH
3e
¹³C NMR (100 MHz, CDCl₃)
f1 (ppm)

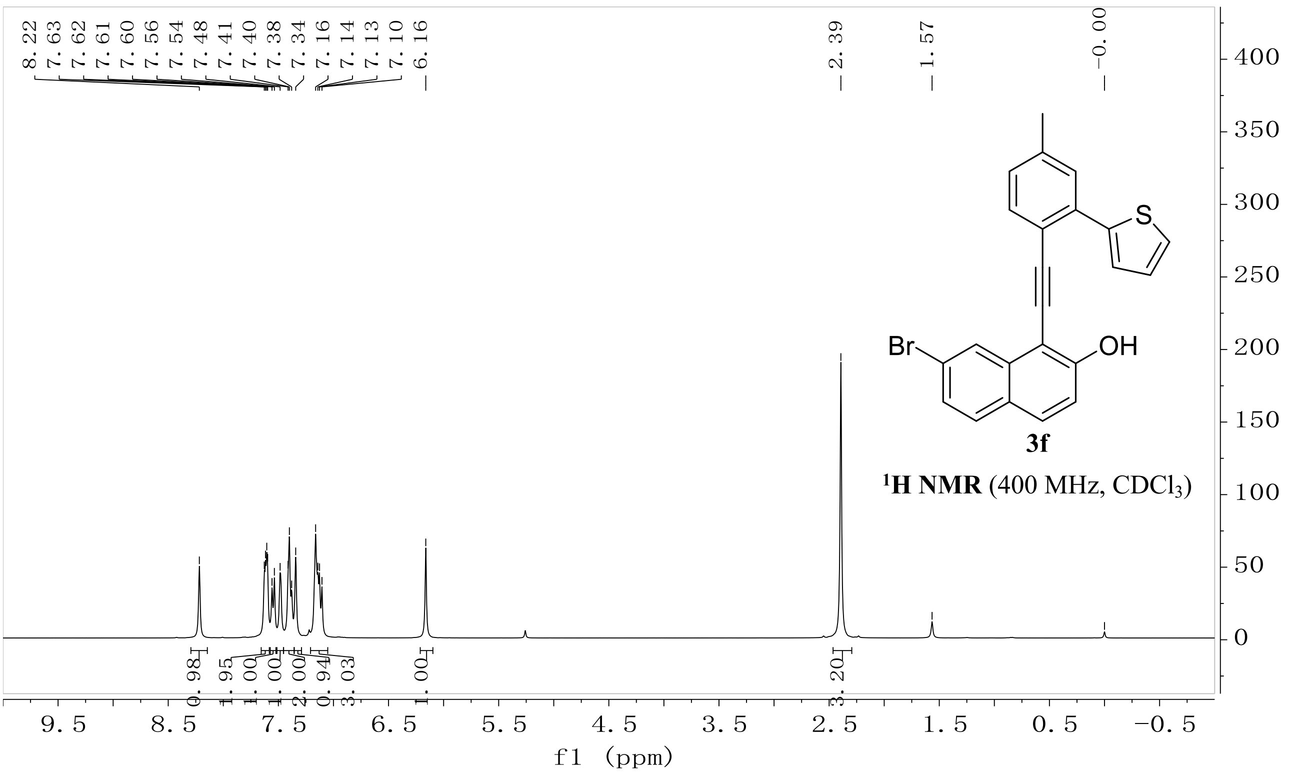
8.22
7.63
7.62
7.61
7.60
7.56
7.54
7.48
7.41
7.40
7.38
7.34
7.16
7.14
7.13
7.10
6.16
2.39
1.57
-0.00
Br
OH
S
3f
¹H NMR (400 MHz, CDCl3)
0.98
1.95
1.00
1.00
2.00
0.94
3.03
1.00
3.20
f1 (ppm)

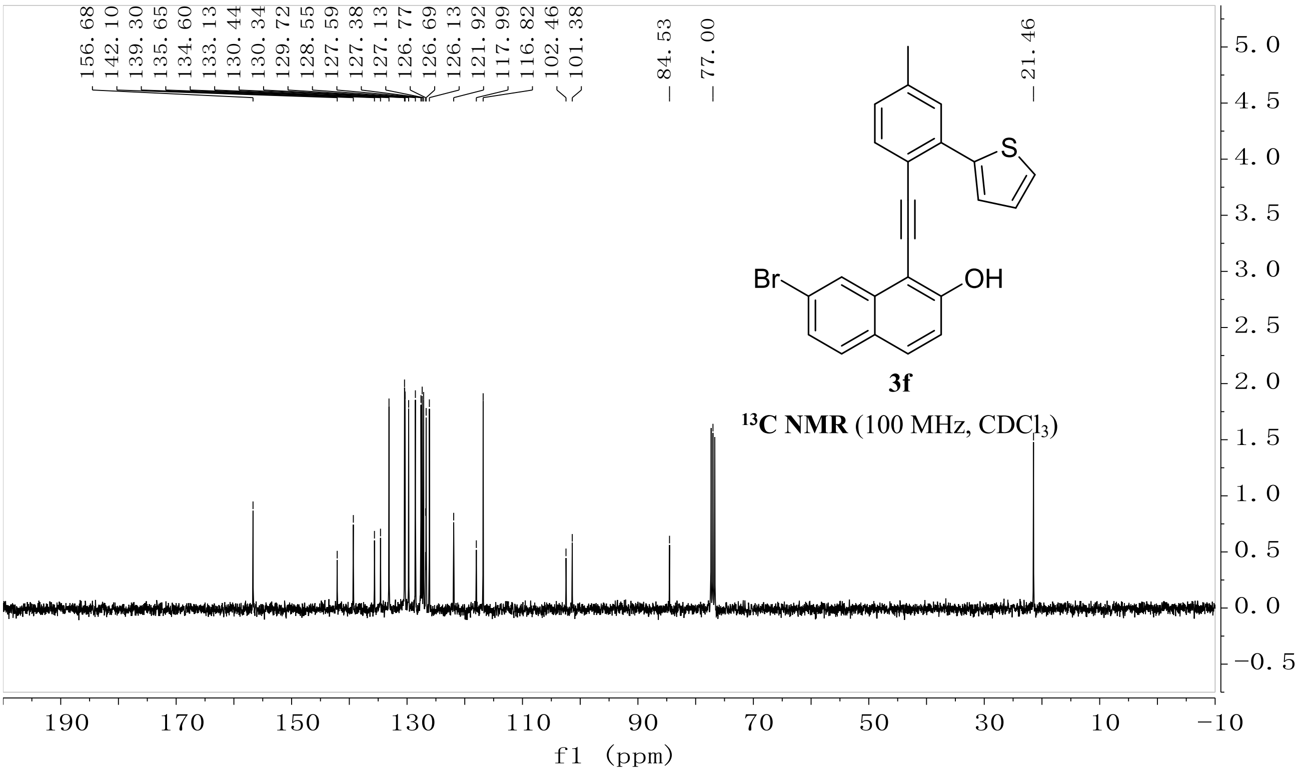
156.68
142.10
139.30
135.65
134.60
133.13
130.44
130.34
129.72
128.55
127.59
127.38
127.13
126.77
126.69
126.13
121.92
117.99
116.82
102.46
101.38
84.53
77.00
21.46
Br
OH
S
3f
¹³C NMR (100 MHz, CDCl3)
f1 (ppm)

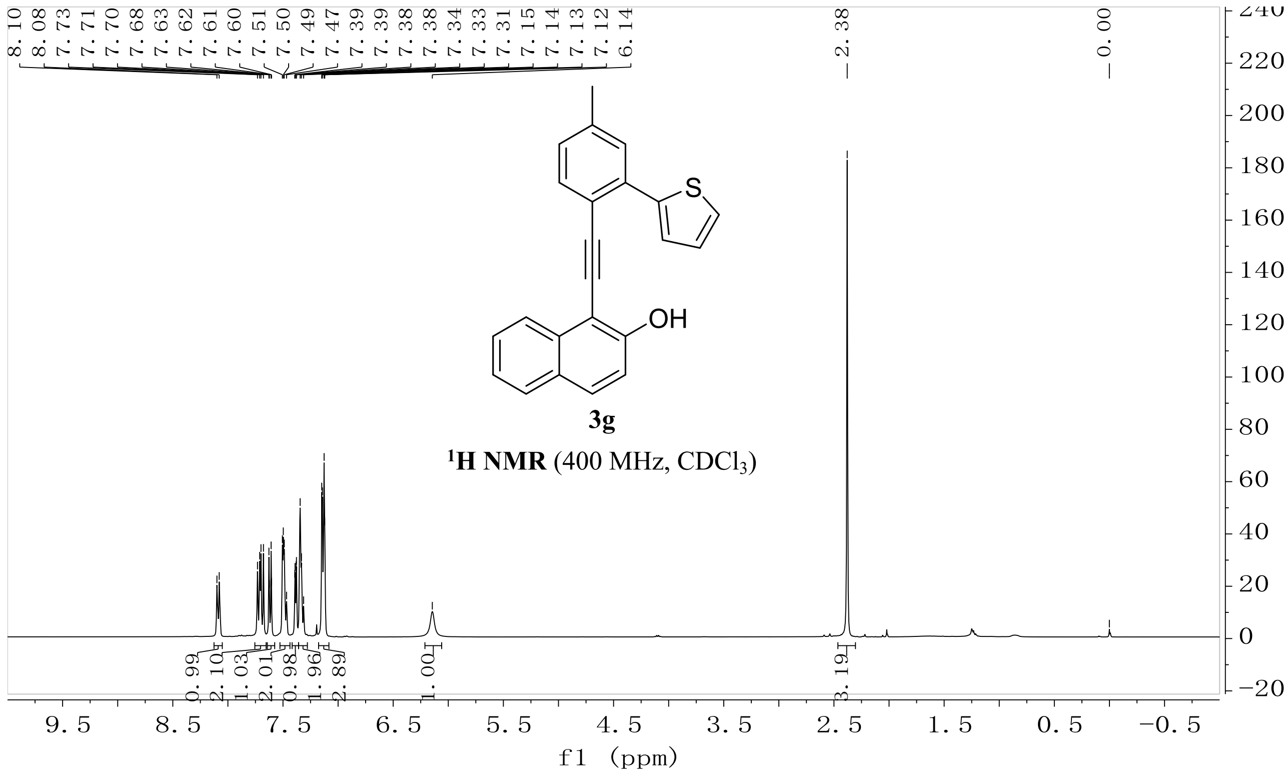
8.10 8.08 7.73 7.71 7.70 7.68 7.63 7.62 7.61 7.60 7.51 7.50 7.49 7.47 7.39 7.39 7.38 7.38 7.34 7.33 7.31 7.15 7.14 7.13 7.12 6.14
2.38
0.00
0.99 2.10 1.03 2.01 0.98 1.96 2.89
1.00
3.19
OH
S
3g
1H NMR (400 MHz, CDCl3)
f1 (ppm)

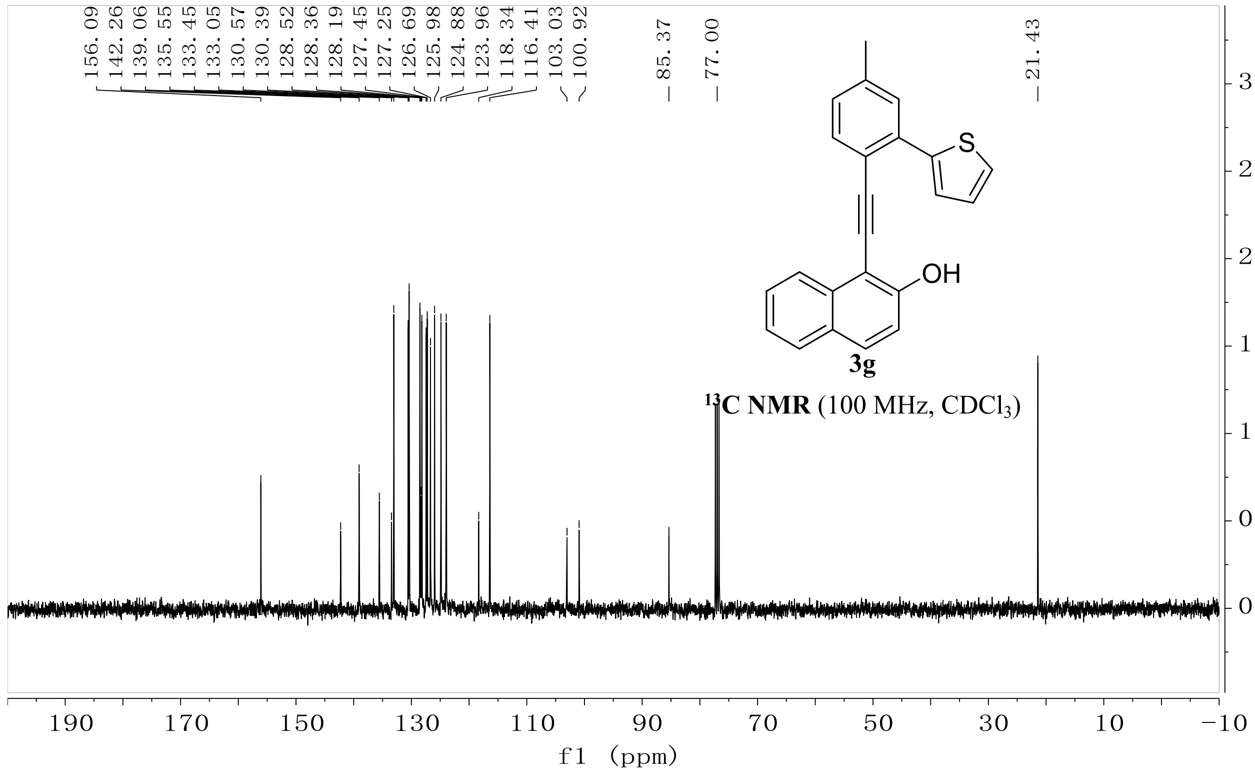
156.09 142.26 139.06 135.55 133.45 133.05 130.57 130.39 128.52 128.36 128.19 127.45 127.25 126.69 125.98 124.88 123.96 118.34 116.41 103.03 100.92
85.37
77.00
21.43
OH
S
3g
13C NMR (100 MHz, CDCl3)
f1 (ppm)

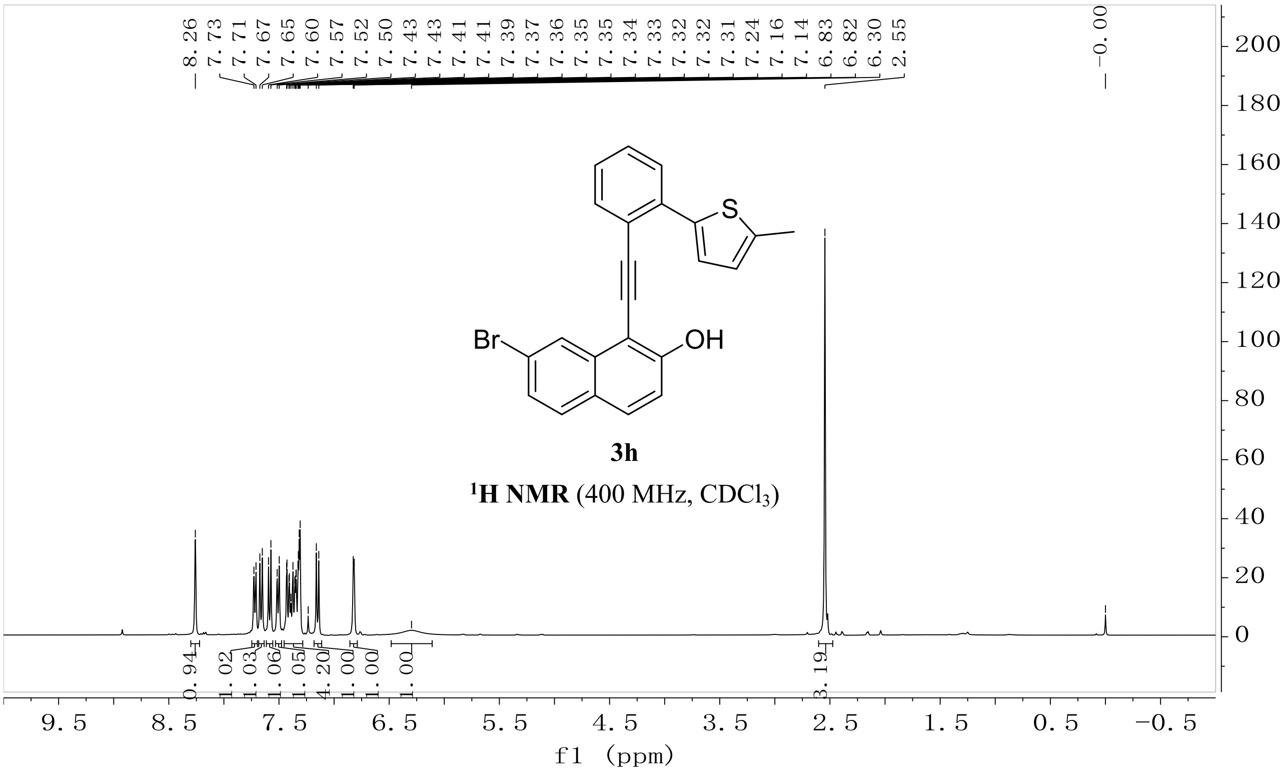
8.26
7.73
7.71
7.67
7.65
7.60
7.57
7.52
7.50
7.43
7.43
7.41
7.39
7.37
7.36
7.35
7.35
7.34
7.33
7.32
7.32
7.31
7.24
7.16
7.14
6.83
6.82
6.30
2.55
-0.00
S
Br
OH
3h
¹H NMR (400 MHz, CDCl₃)
0.94
1.02
1.03
1.06
1.05
4.20
1.00
1.00
1.00
3.19
f1 (ppm)

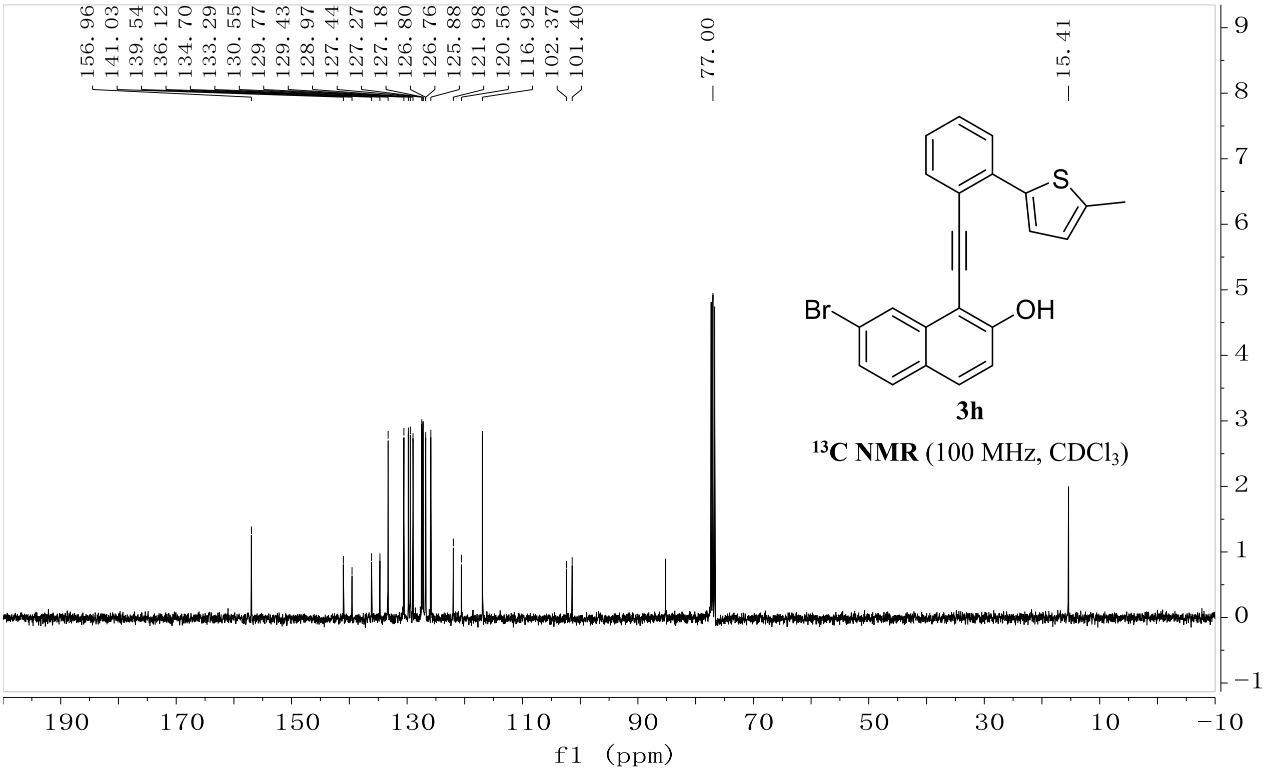
156.96
141.03
139.54
136.12
134.70
133.29
130.55
129.77
129.43
128.97
127.44
127.27
127.18
126.80
126.76
125.88
121.98
120.56
116.92
102.37
101.40
77.00
15.41
S
Br
OH
3h
¹³C NMR (100 MHz, CDCl₃)
f1 (ppm)

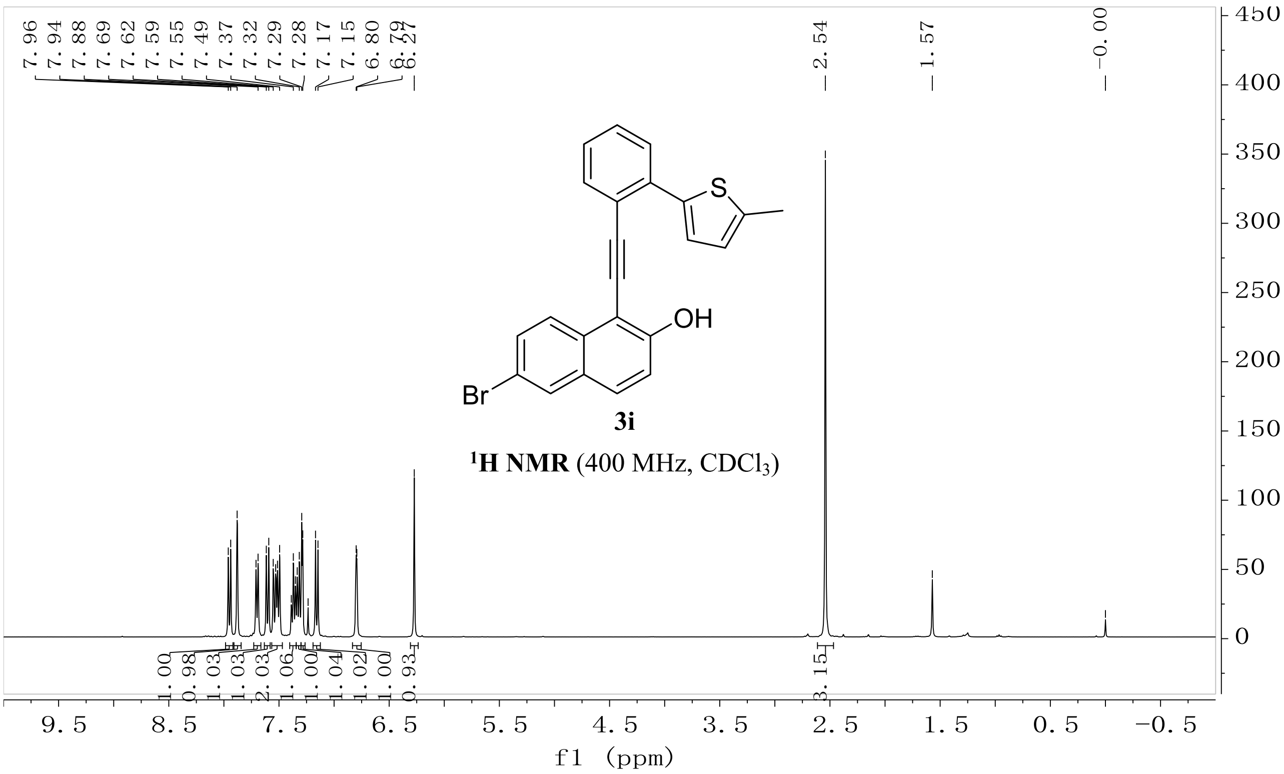
OH
Br
S
3i
1H NMR (400 MHz, CDCl3)
f1 (ppm)

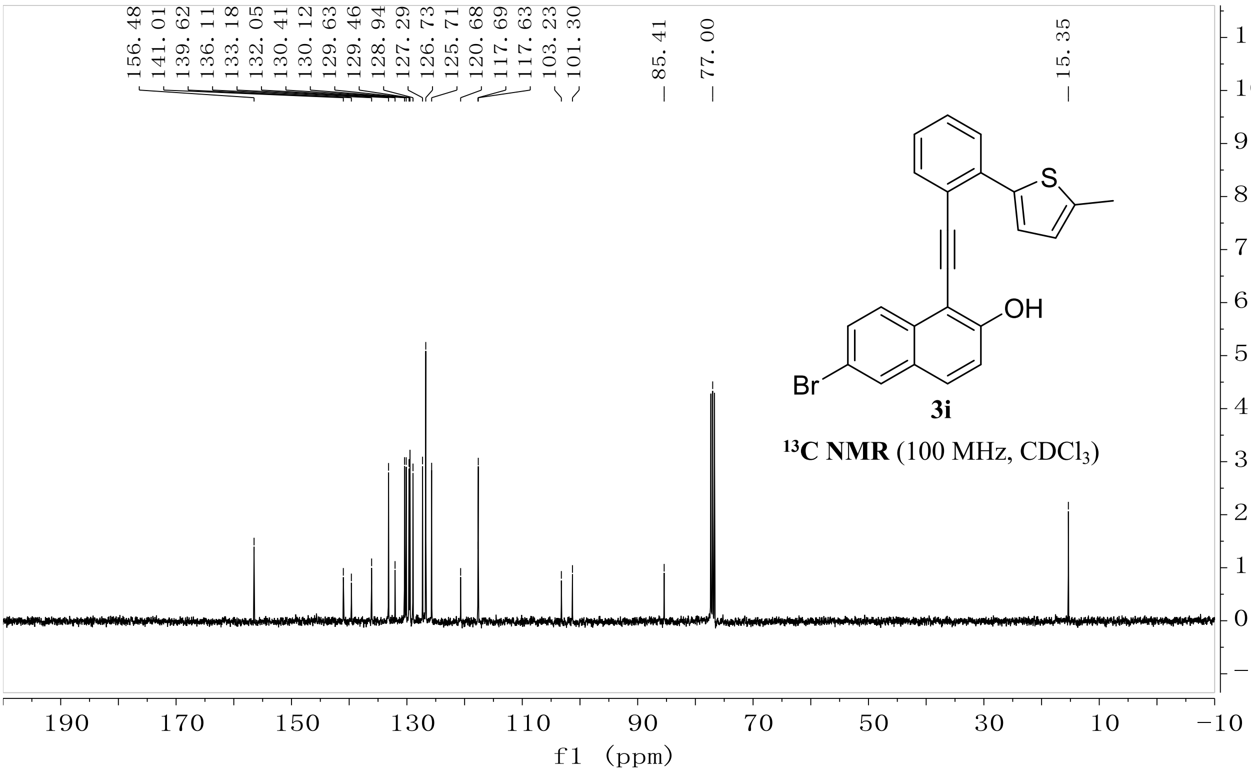
OH
Br
S
3i
13C NMR (100 MHz, CDCl3)
f1 (ppm)

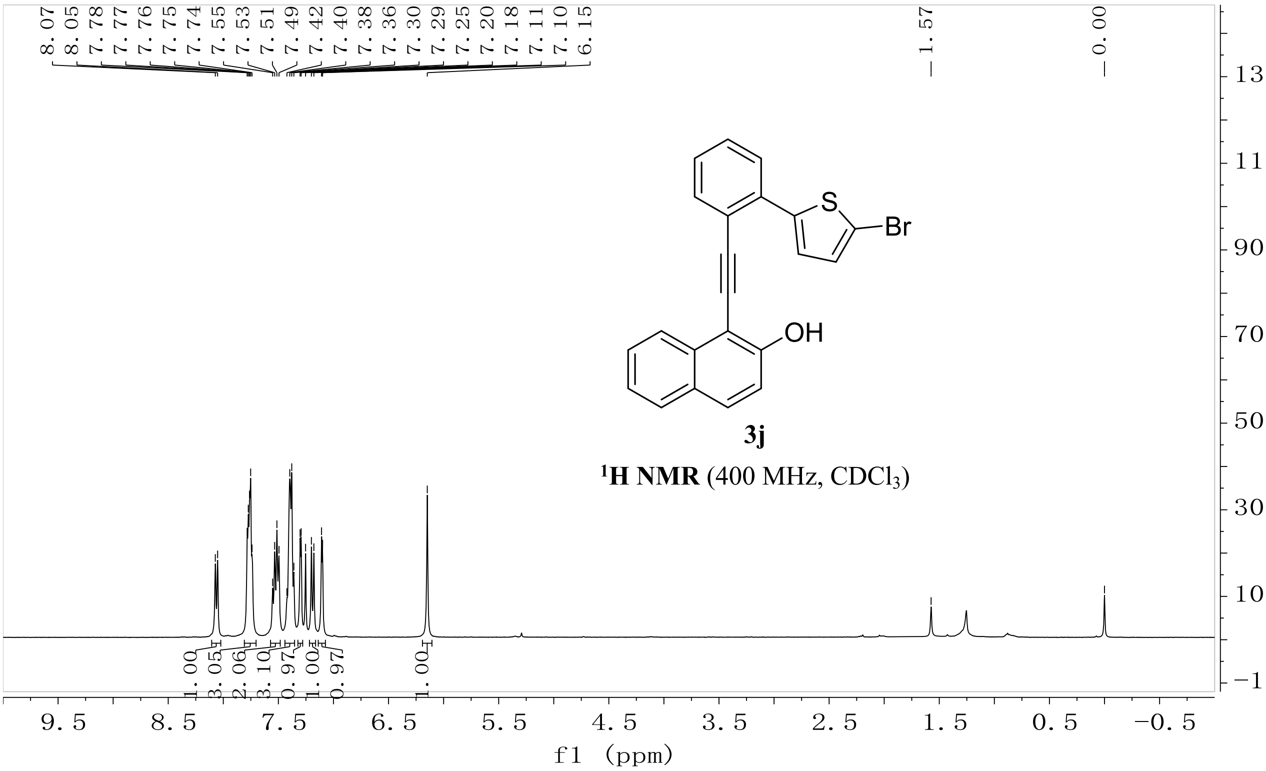
8.07 8.05 7.78 7.77 7.76 7.75 7.74 7.55 7.53 7.51 7.49 7.42 7.40 7.38 7.36 7.30 7.29 7.25 7.20 7.18 7.11 7.10 6.15
1.57
0.00
S
Br
OH
3j
1H NMR (400 MHz, CDCl3)
1.00 3.05 2.06 3.10 0.97 1.00 0.97
1.00
f1 (ppm)

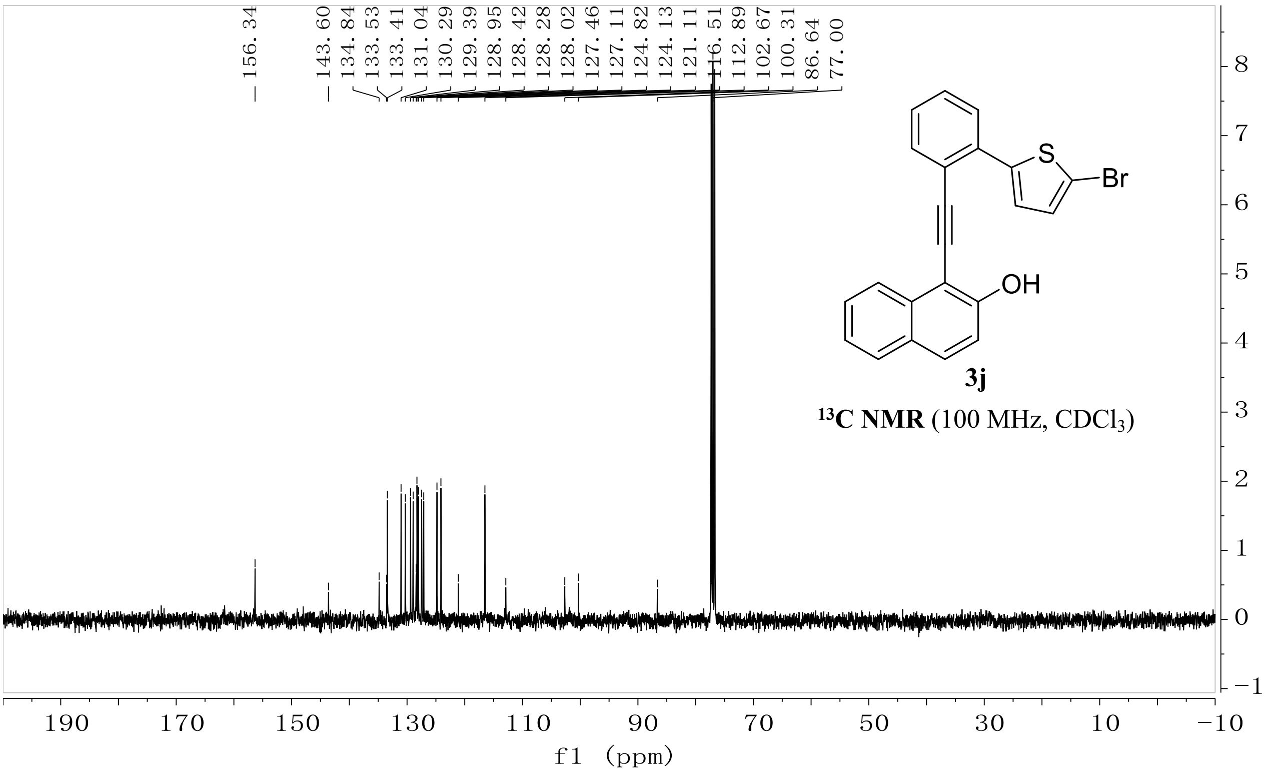
156.34
143.60 134.84 133.53 133.41 131.04 130.29 129.39 128.95 128.42 128.28 128.02 127.46 127.11 124.82 124.13 121.11 116.51 112.89 102.67 100.31 86.64 77.00
S
Br
OH
3j
13C NMR (100 MHz, CDCl3)
f1 (ppm)

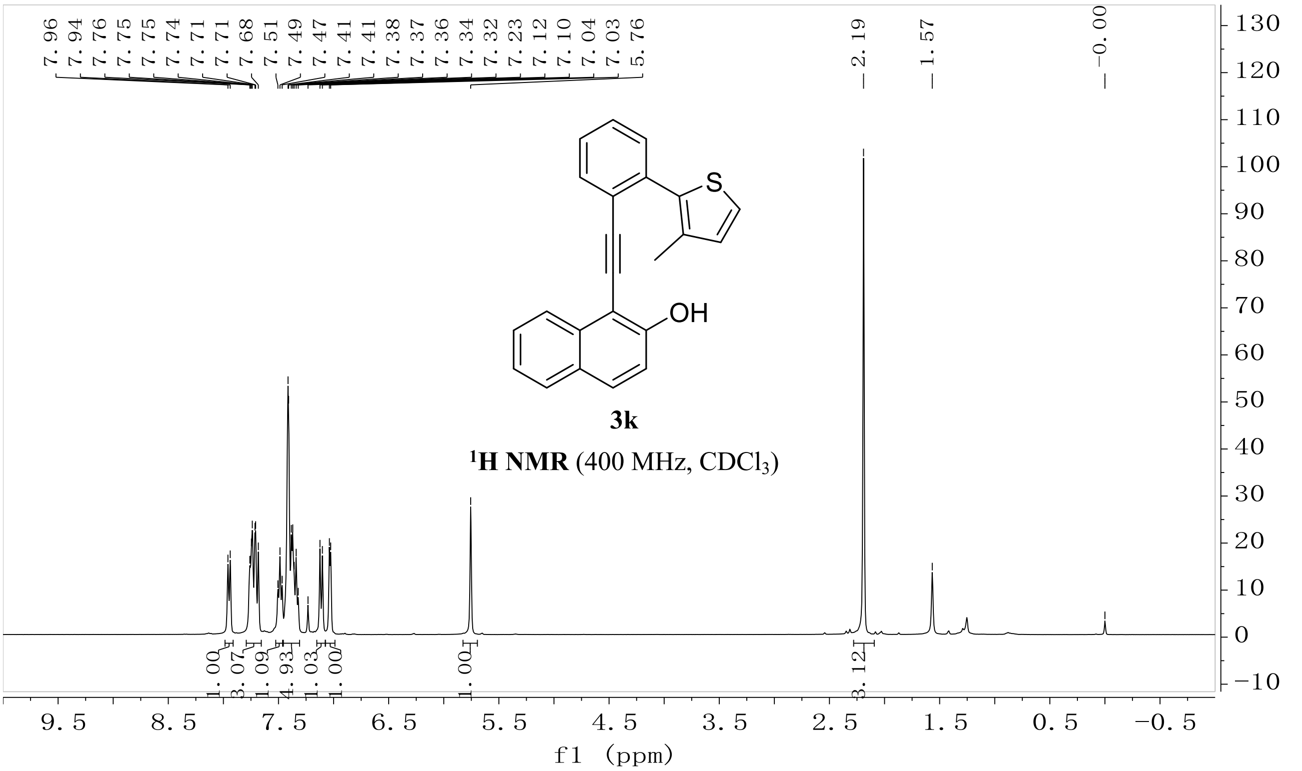

3k

^{1}H NMR (400 MHz, $CDCl_3$)

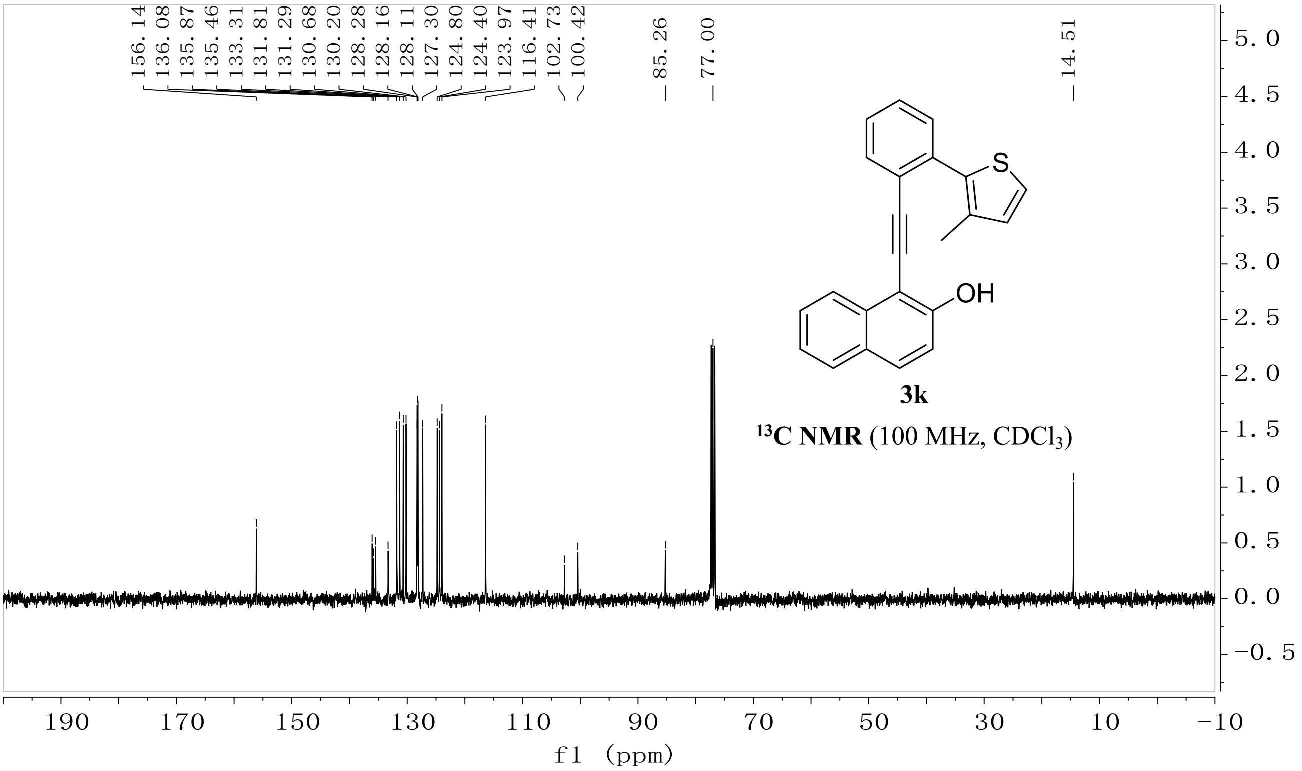

3k

^{13}C NMR (100 MHz, $CDCl_3$)

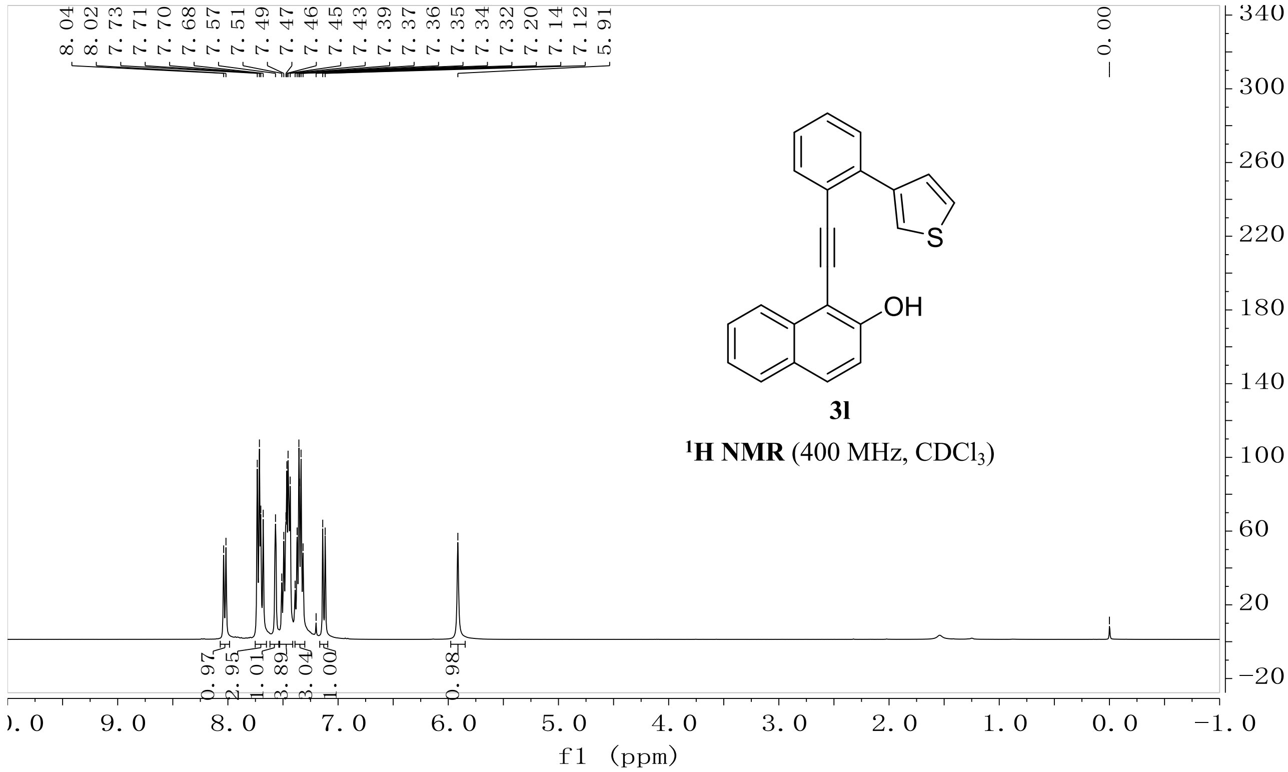
8.04 8.02 7.73 7.71 7.70 7.68 7.57 7.51 7.49 7.47 7.46 7.45 7.43 7.39 7.37 7.36 7.35 7.34 7.32 7.20 7.14 7.12 5.91
0.00
OH
S
3l
^{1}H NMR (400 MHz, CDCl$_3$)
0.97 2.95 1.01 3.89 3.04 1.00 0.98
f1 (ppm)

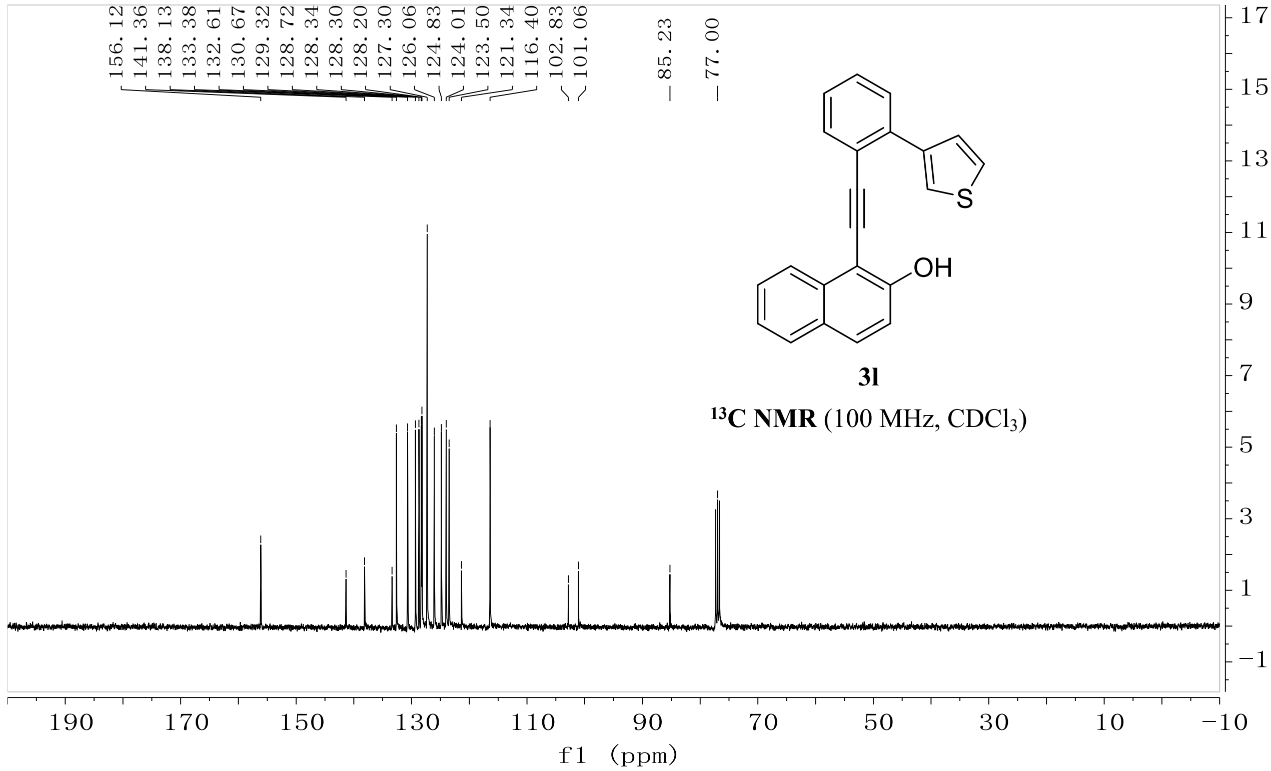
156.12 141.36 138.13 133.38 132.61 130.67 129.32 128.72 128.34 128.30 128.20 127.30 126.06 124.83 124.01 123.50 121.34 116.40 102.83 101.06
85.23
77.00
OH
S
3l
^{13}C NMR (100 MHz, CDCl$_3$)
f1 (ppm)

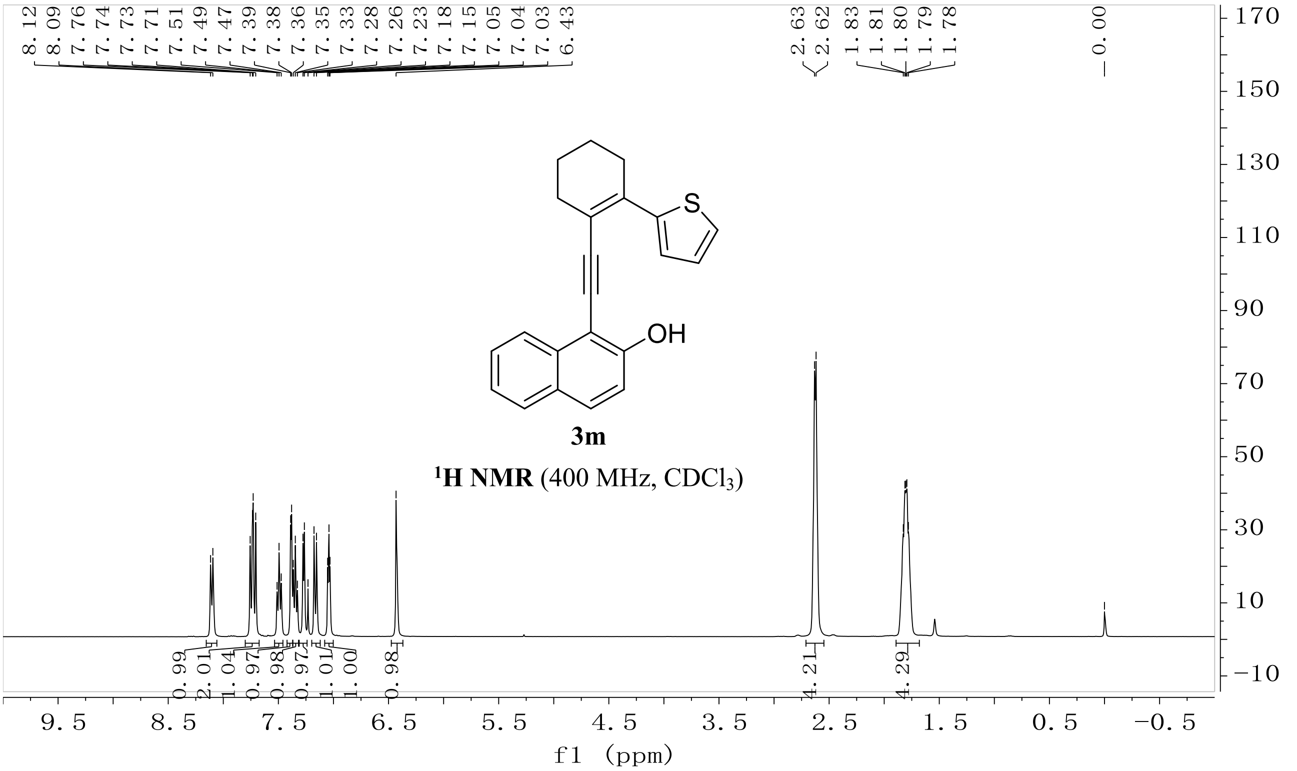
8.12 8.09 7.76 7.74 7.73 7.71 7.51 7.49 7.47 7.39 7.38 7.36 7.35 7.33 7.28 7.26 7.23 7.18 7.15 7.05 7.04 7.03 6.43
2.63 2.62 1.83 1.81 1.80 1.79 1.78
0.00
OH
S
3m
^{1}H NMR (400 MHz, CDCl$_3$)
0.99 2.01 1.04 0.97 0.98 0.97 1.01 1.00 0.98 4.21 4.29
f1 (ppm)

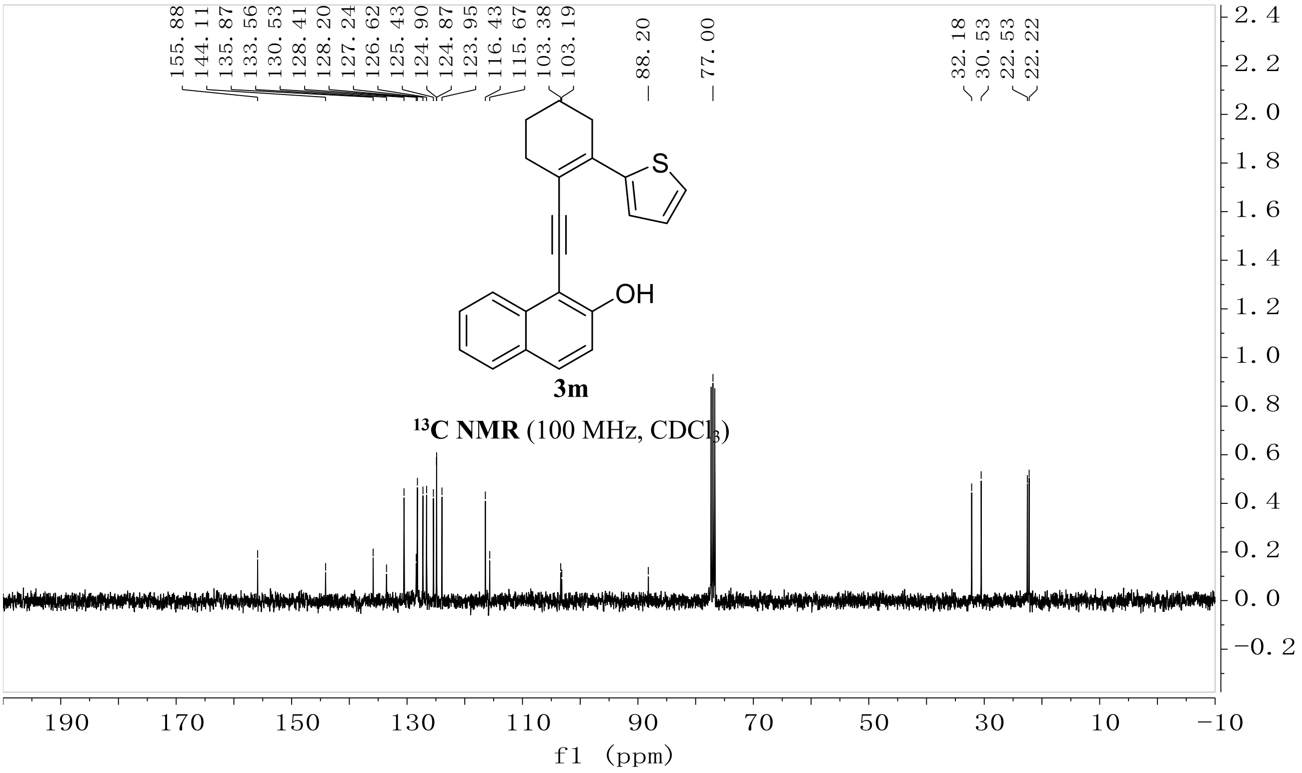
155.88 144.11 135.87 133.56 130.53 128.41 128.20 127.24 126.62 125.43 124.90 124.87 123.95 116.43 115.67 103.38 103.19
88.20
77.00
32.18 30.53 22.53 22.22
OH
S
3m
^{13}C NMR (100 MHz, CDCl$_3$)
f1 (ppm)

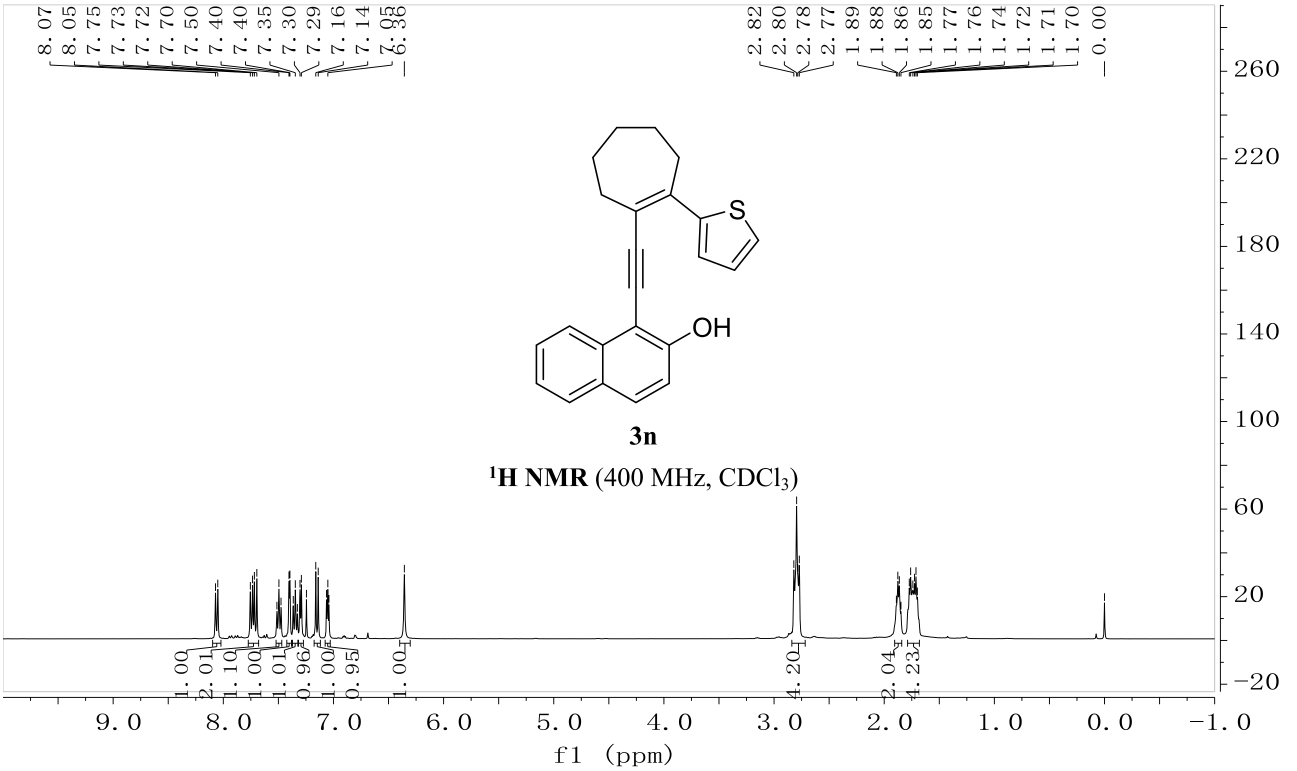

3n

¹H NMR (400 MHz, CDCl₃)

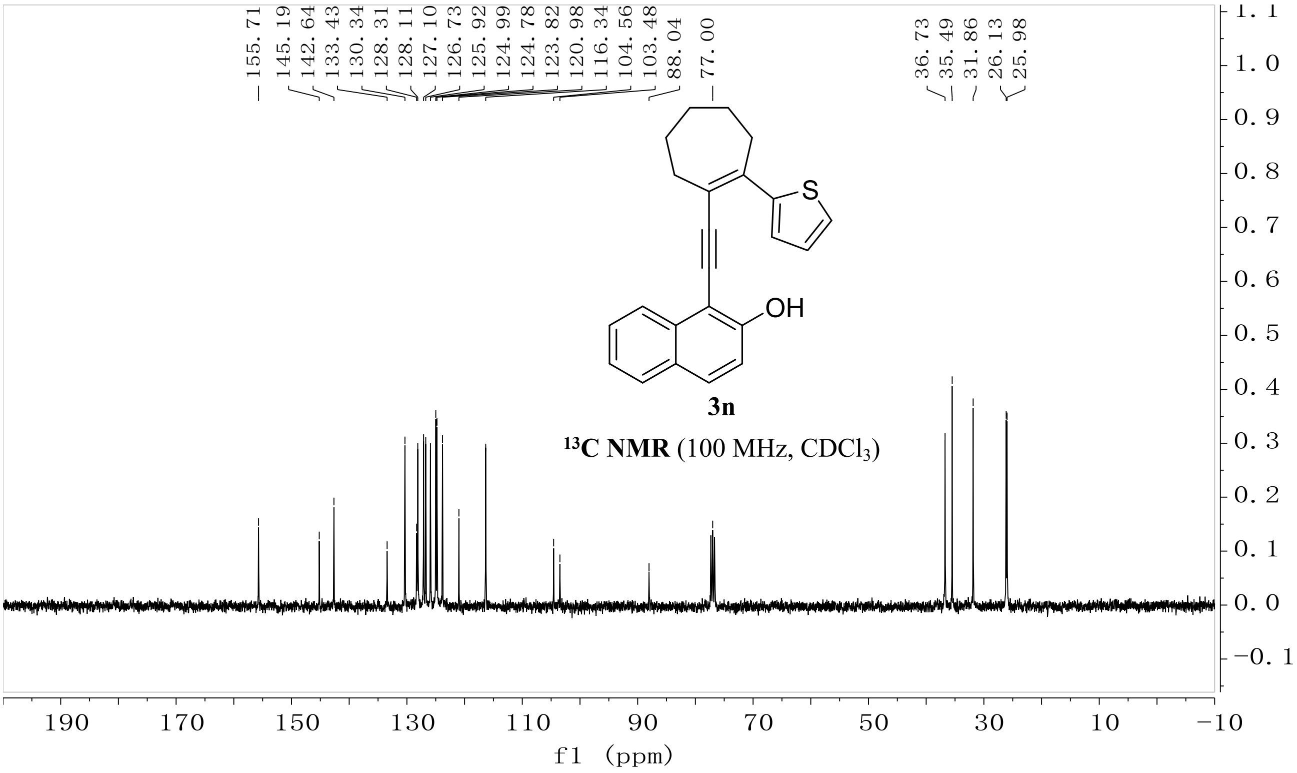

3n

¹³C NMR (100 MHz, CDCl₃)

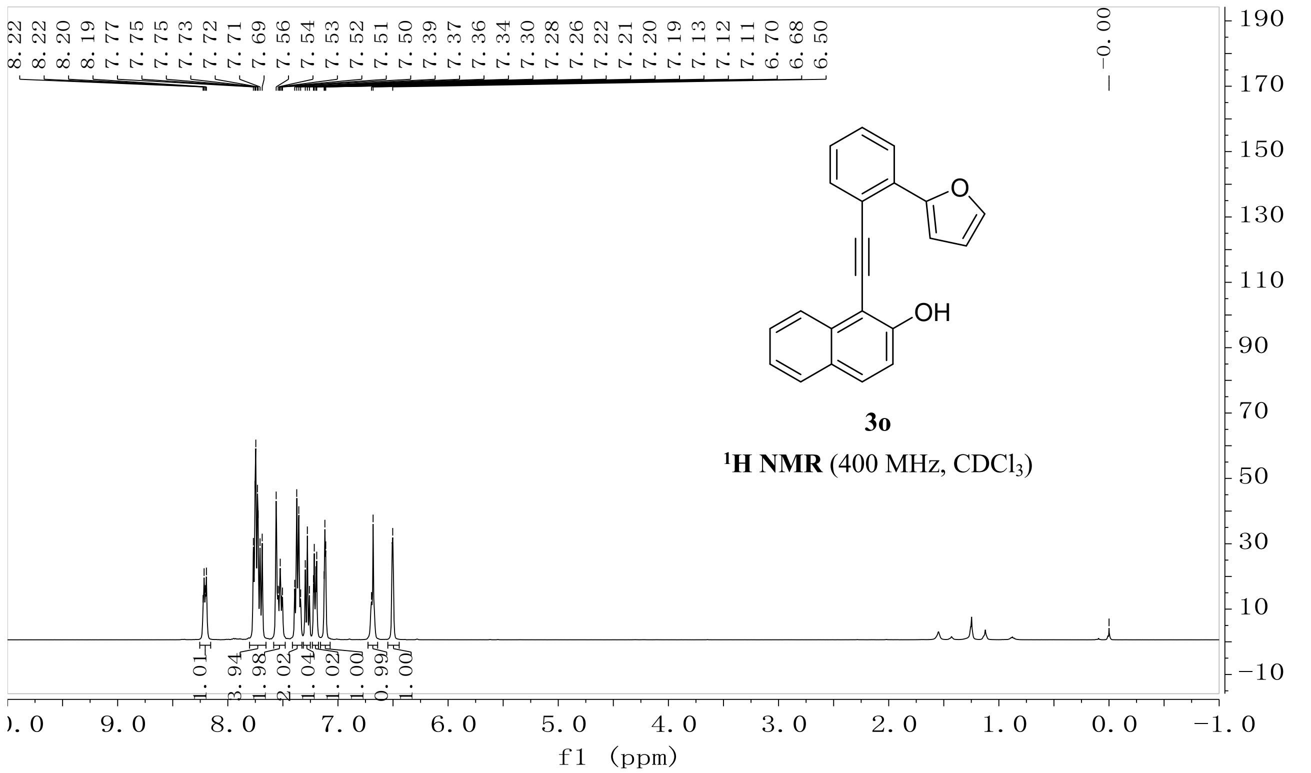
3o
1H NMR (400 MHz, CDCl3)
f1 (ppm)

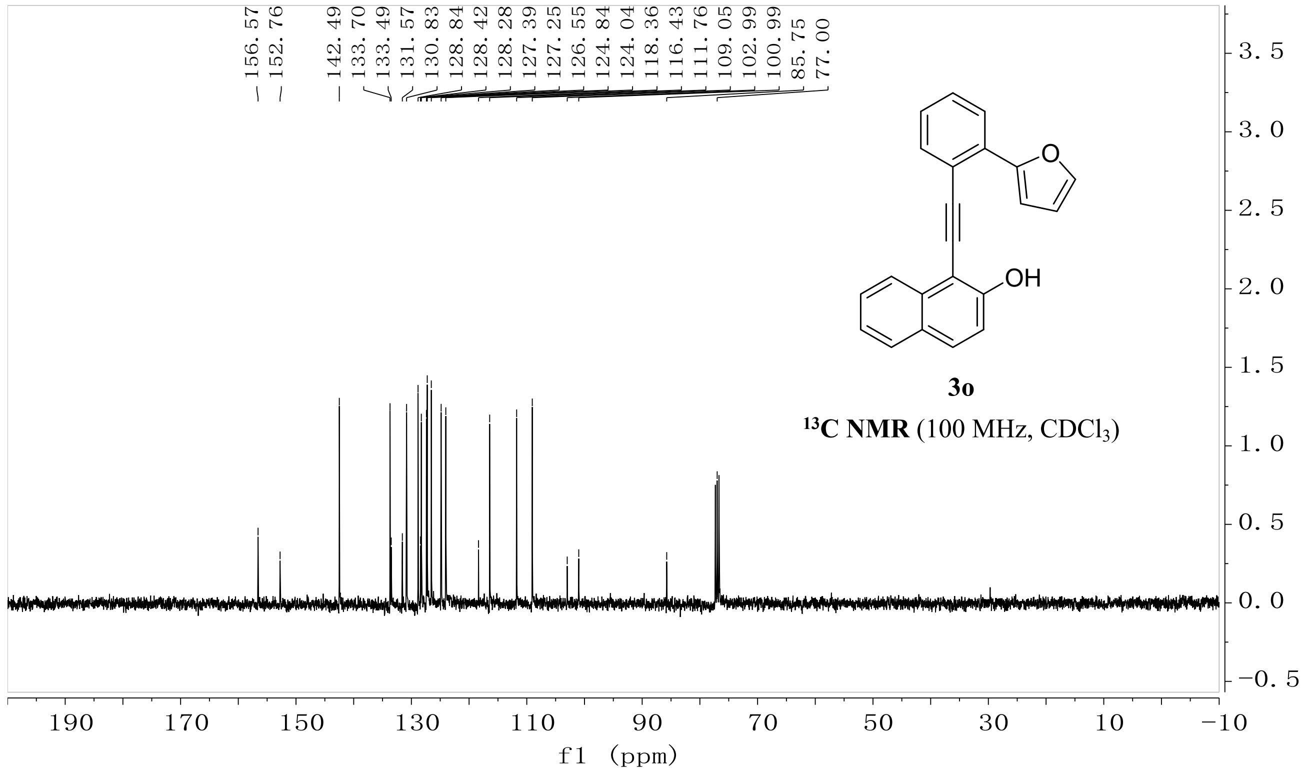
3o
13C NMR (100 MHz, CDCl3)
f1 (ppm)

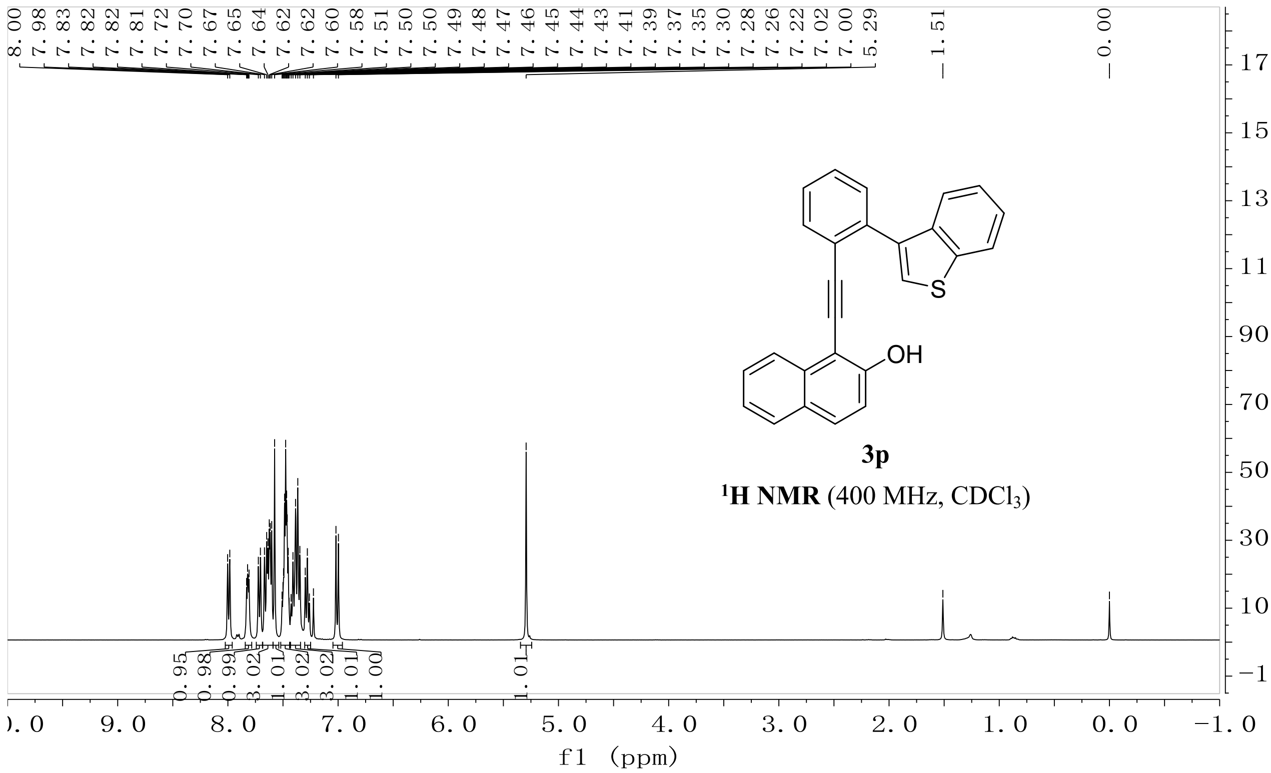

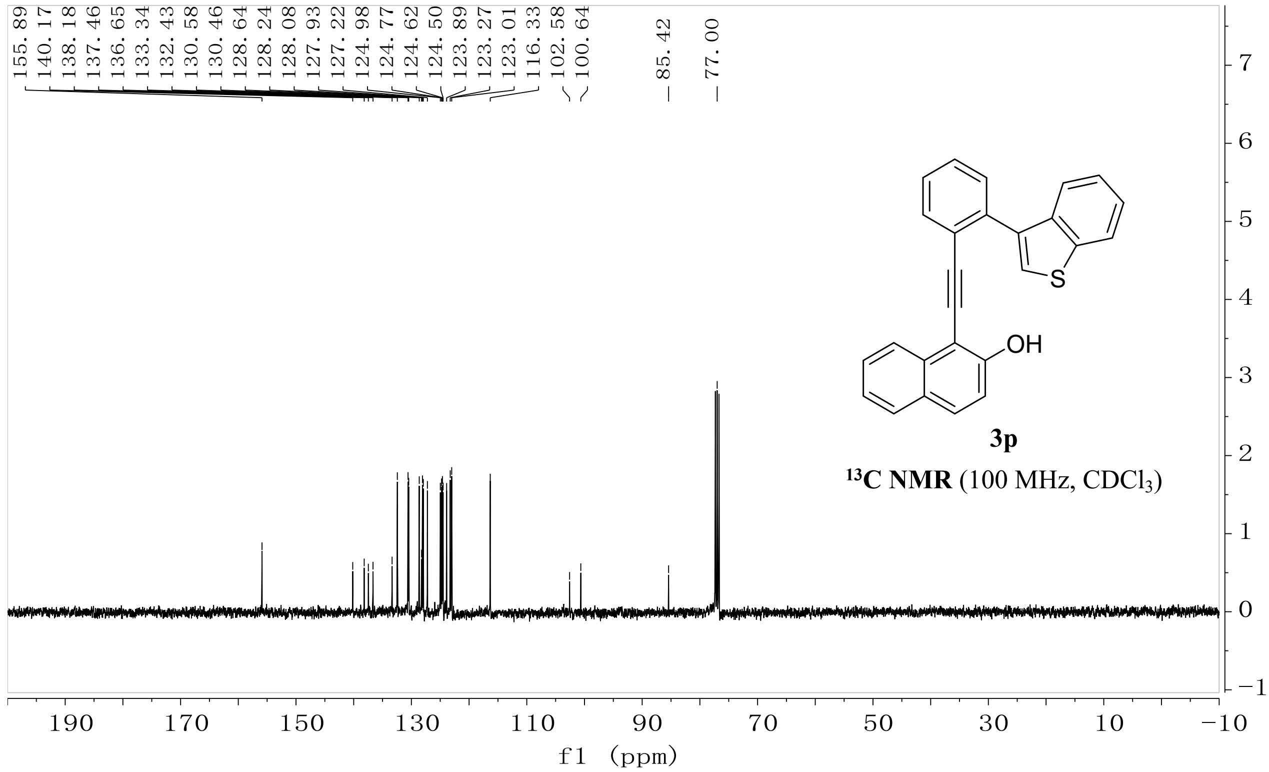

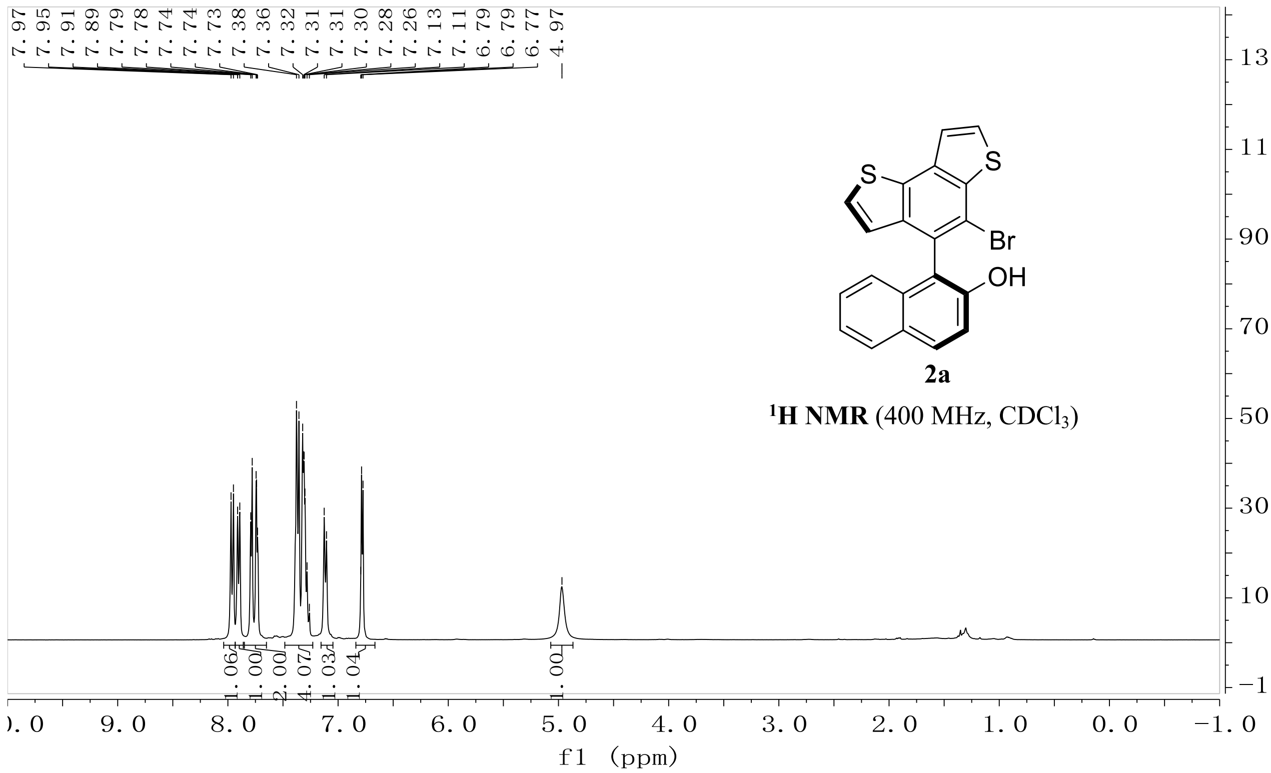
7.97 7.95 7.91 7.89 7.79 7.78 7.74 7.74 7.73 7.38 7.36 7.32 7.31 7.31 7.30 7.28 7.26 7.13 7.11 6.79 6.79 6.77 4.97
1.06 1.00 2.00 4.07 1.03 1.04 1.00
S
S
Br
OH
2a
¹H NMR (400 MHz, CDCl₃)
f1 (ppm)

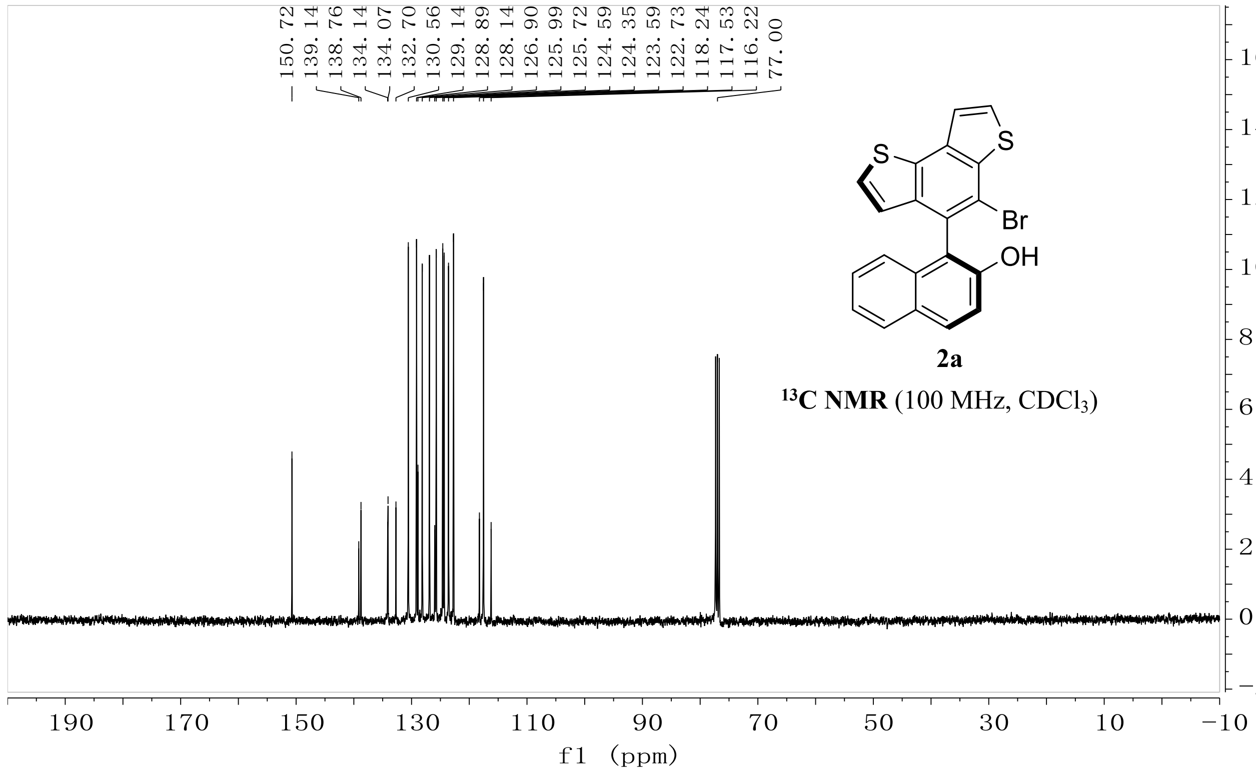
150.72 139.14 138.76 134.14 134.07 132.70 130.56 129.14 128.89 128.14 126.90 125.99 125.72 124.59 124.35 123.59 122.73 118.24 117.53 116.22 77.00
S
S
Br
OH
2a
¹³C NMR (100 MHz, CDCl₃)
f1 (ppm)

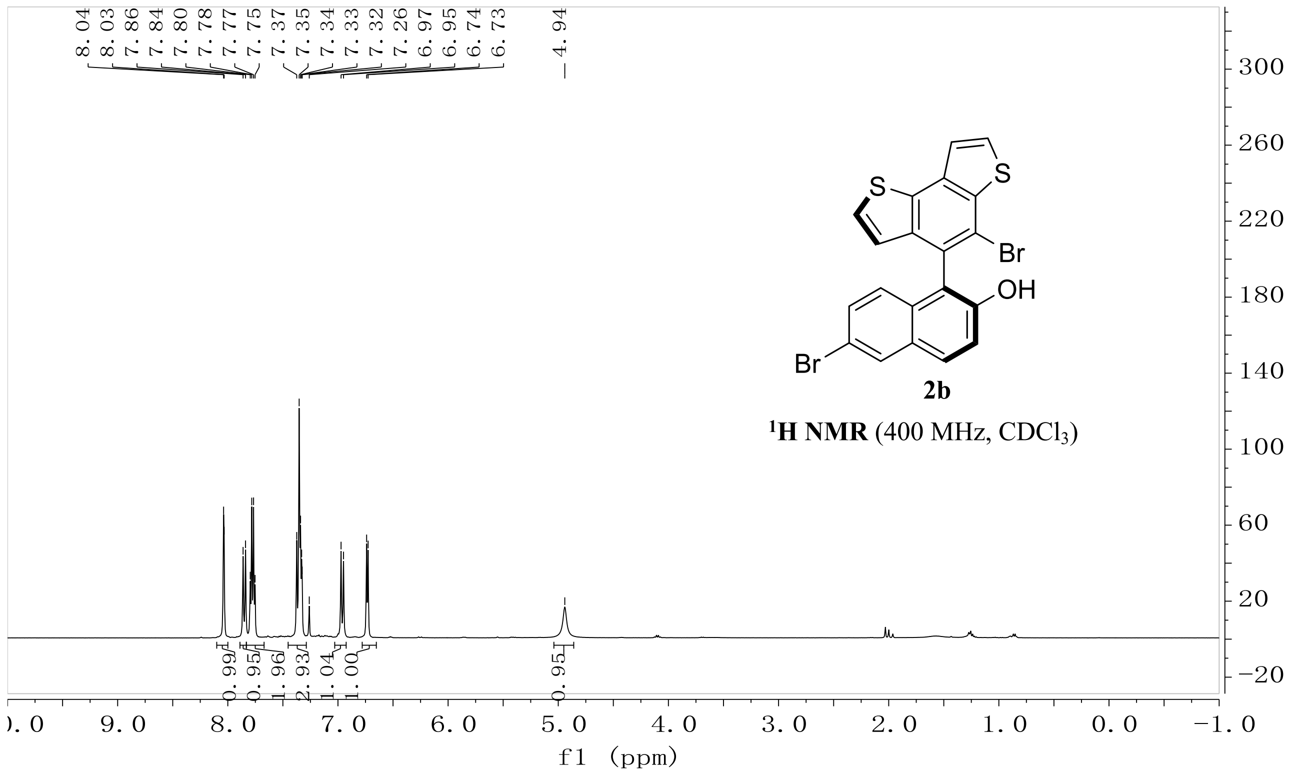

8.04 8.03 7.86 7.84 7.80 7.78 7.77 7.75 7.37 7.35 7.34 7.33 7.32 7.26 6.97 6.95 6.74 6.73 4.94
0.99 0.95 1.96 2.93 1.04 1.00 0.95
2b
^{1}H NMR (400 MHz, CDCl$_3$)
f1 (ppm)

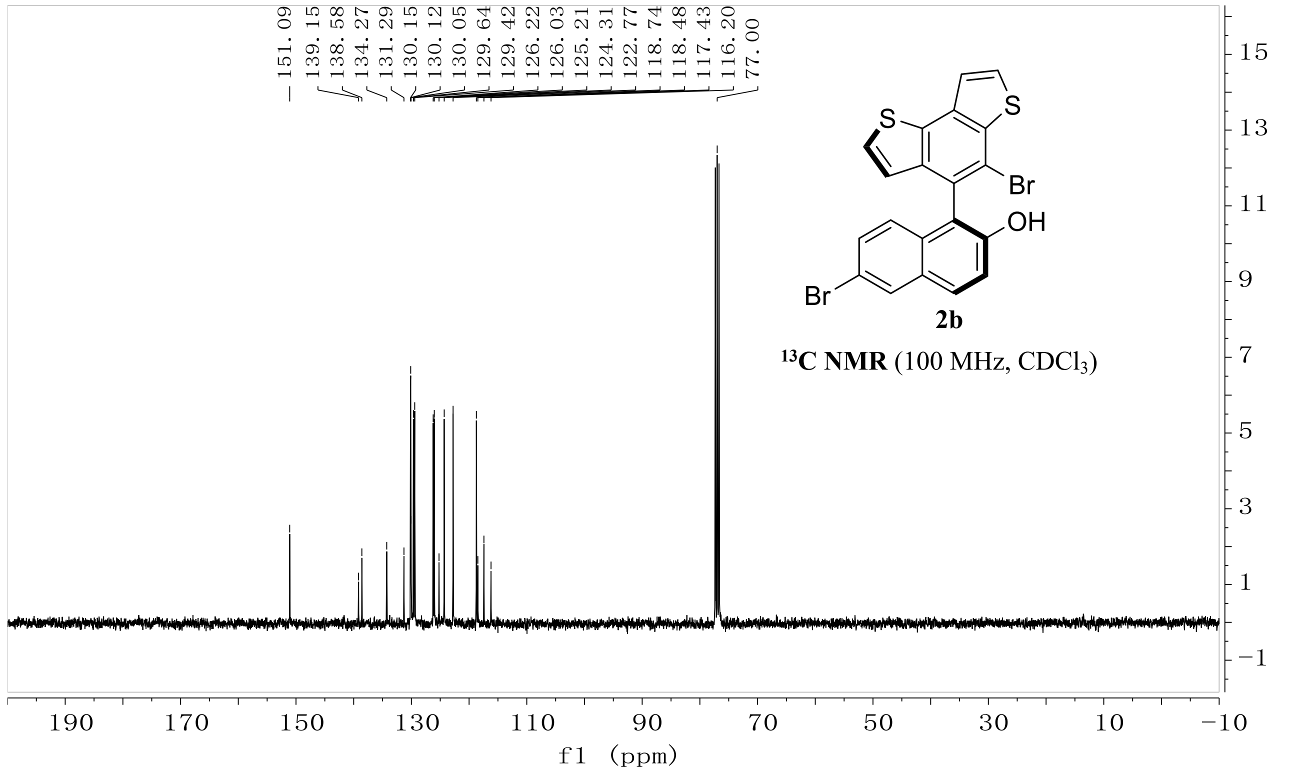

151.09 139.15 138.58 134.27 131.29 130.15 130.12 130.05 129.64 129.42 126.22 126.03 125.21 124.31 122.77 118.74 118.48 117.43 116.20 77.00
2b
^{13}C NMR (100 MHz, CDCl$_3$)
f1 (ppm)

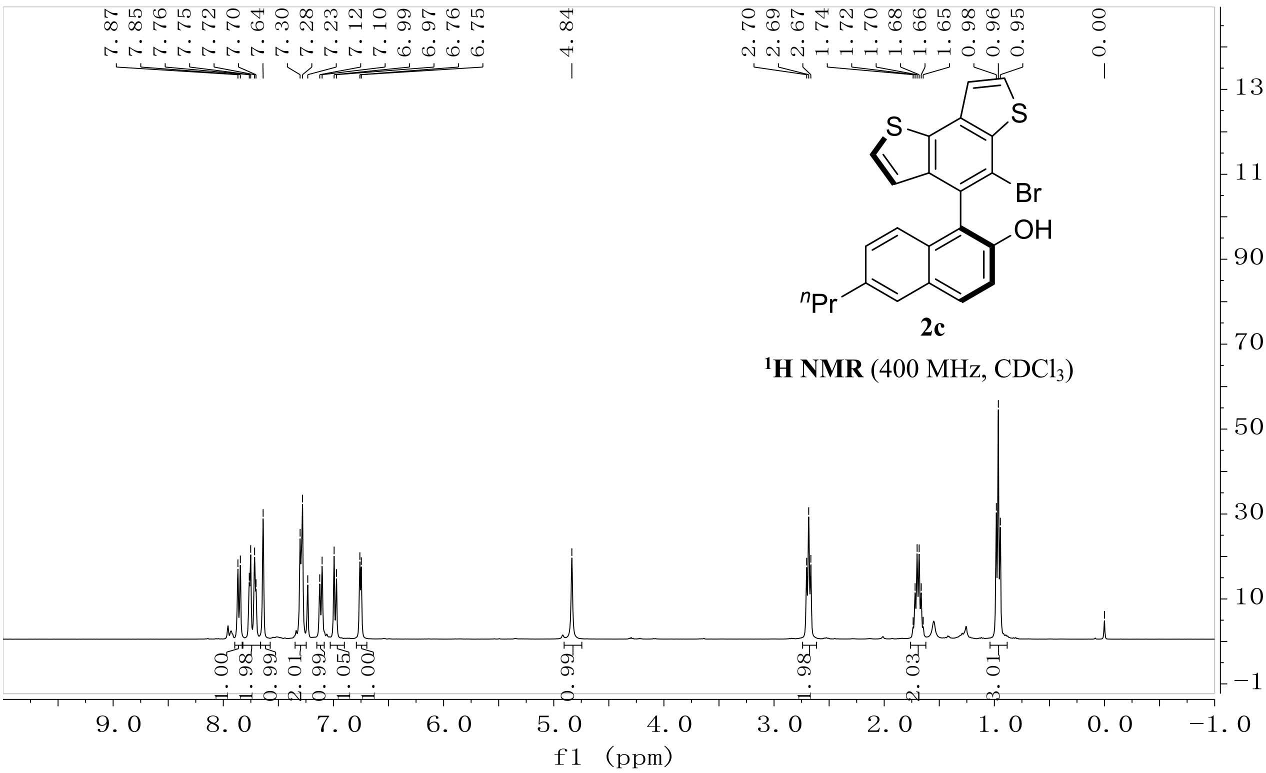
7.87
7.85
7.76
7.75
7.72
7.70
7.64
7.30
7.28
7.23
7.12
7.10
6.99
6.97
6.76
6.75
4.84
2.70
2.69
2.67
1.74
1.72
1.70
1.68
1.66
1.65
0.98
0.96
0.95
0.00
S
S
Br
OH
^{n}Pr
2c
^{1}H NMR (400 MHz, CDCl$_3$)
1.00
1.98
0.99
2.01
0.99
1.05
1.00
0.99
1.98
2.03
3.01
f1 (ppm)

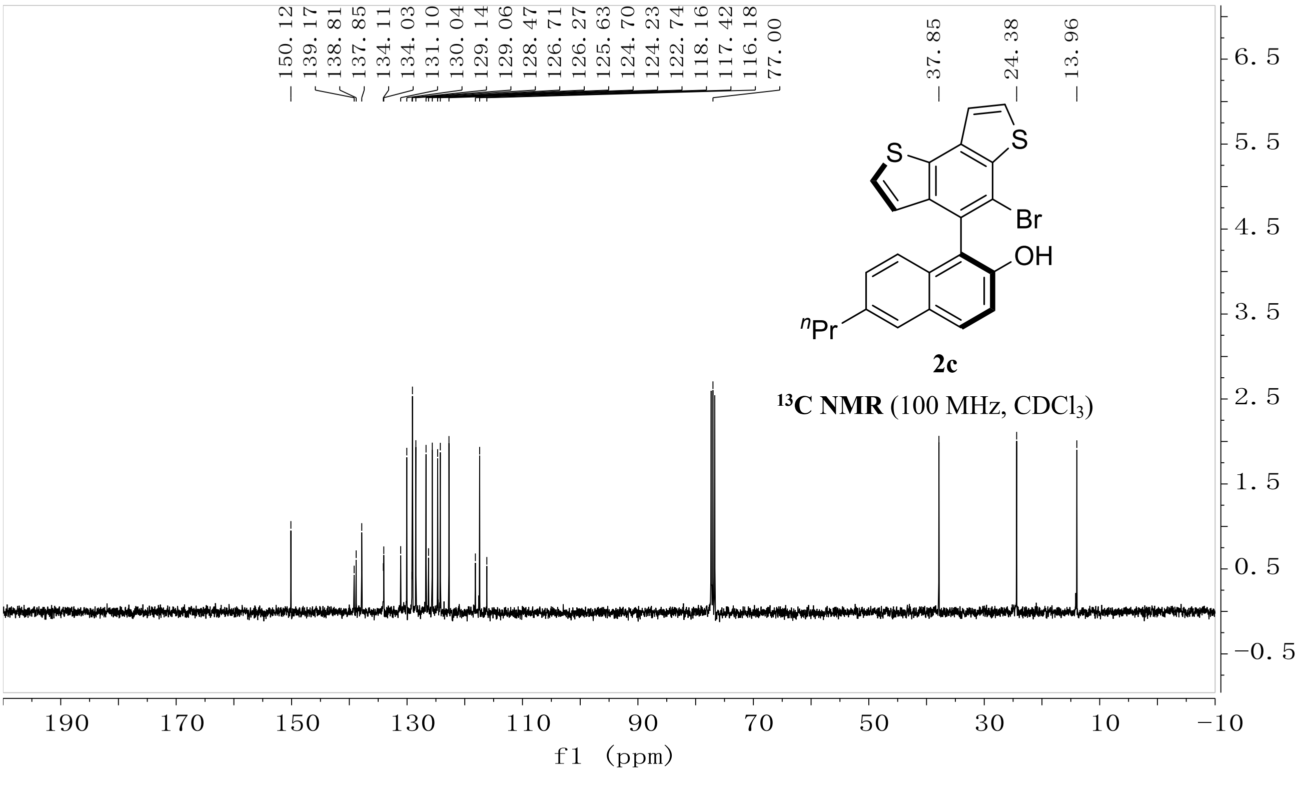
150.12
139.17
138.81
137.85
134.11
134.03
131.10
130.04
129.14
129.06
128.47
126.71
126.27
125.63
124.70
124.23
122.74
118.16
117.42
116.18
77.00
37.85
24.38
13.96
S
S
Br
OH
^{n}Pr
2c
^{13}C NMR (100 MHz, CDCl$_3$)
f1 (ppm)

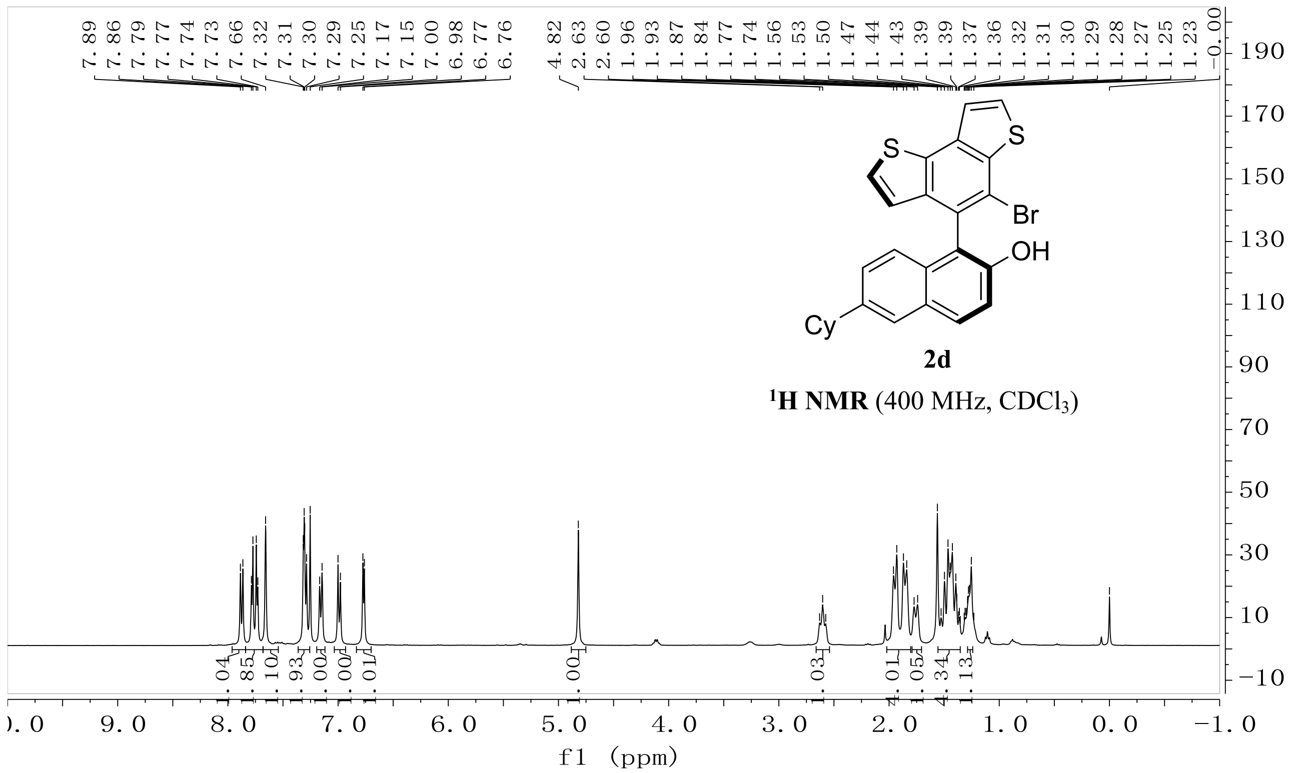

2d

^{1}H NMR (400 MHz, $CDCl_3$)

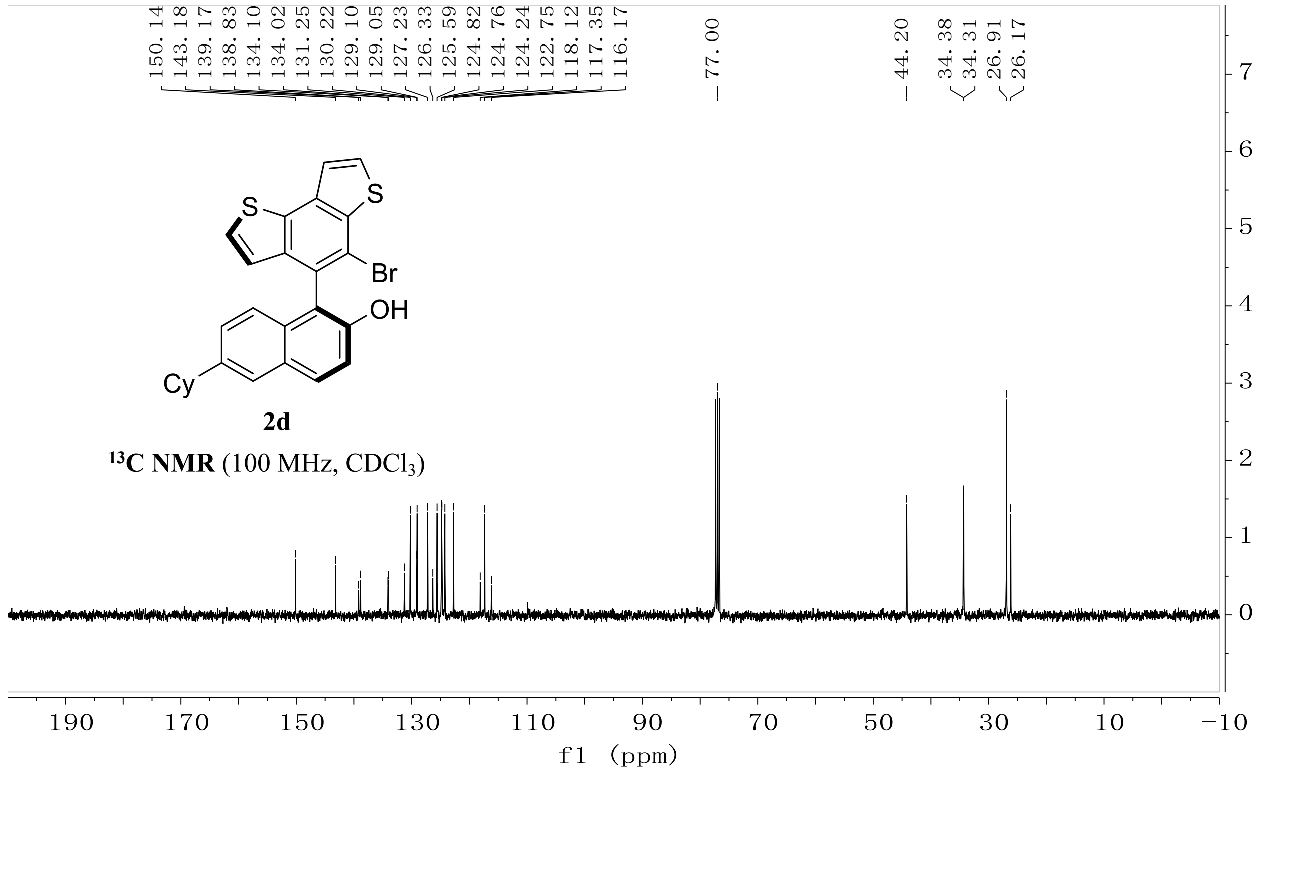

2d

^{13}C NMR (100 MHz, $CDCl_3$)

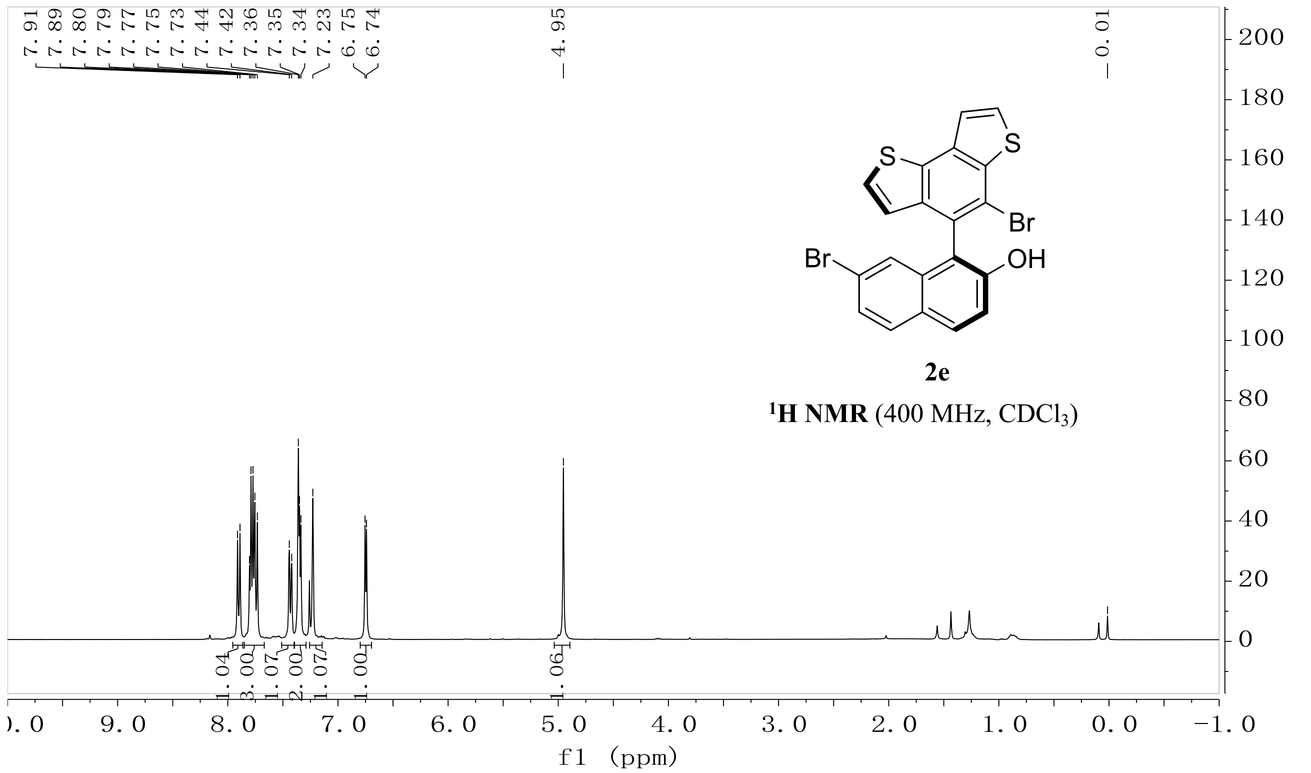

2e

^{1}H NMR (400 MHz, $CDCl_3$)

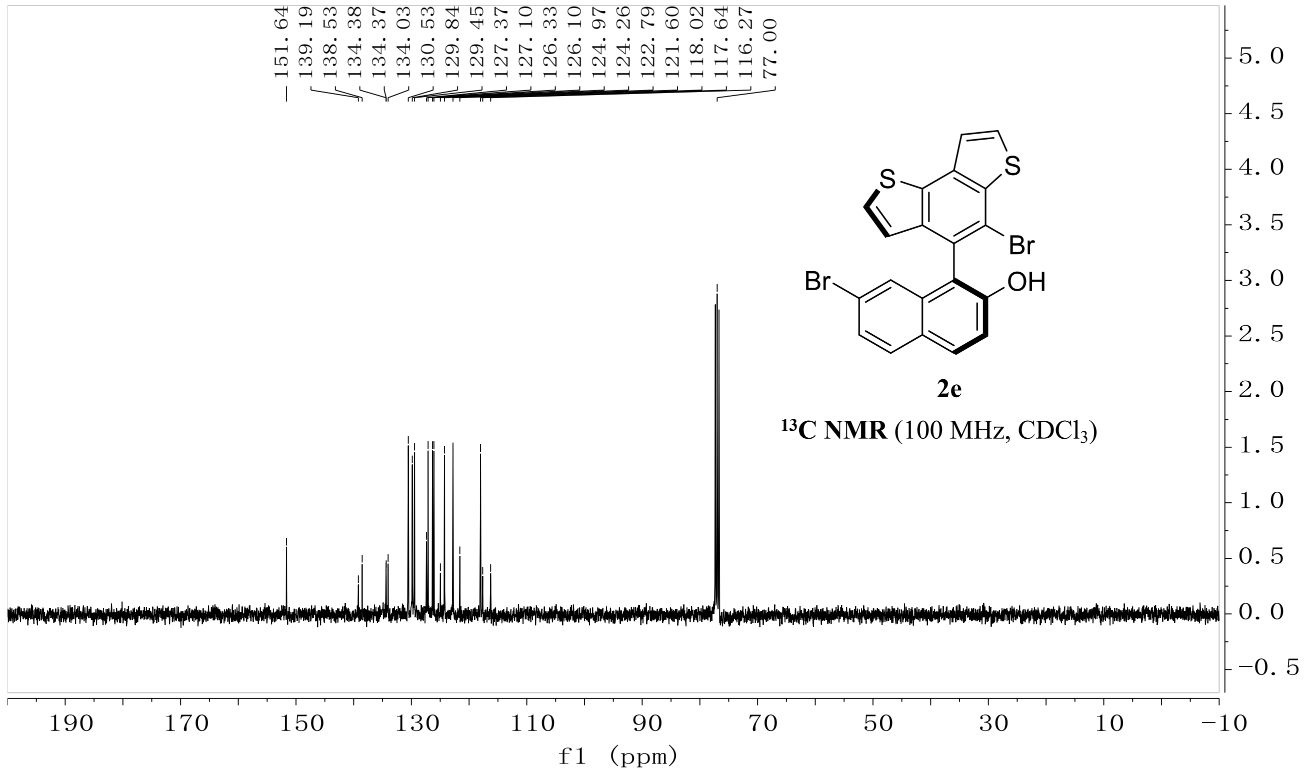

2e

^{13}C NMR (100 MHz, $CDCl_3$)

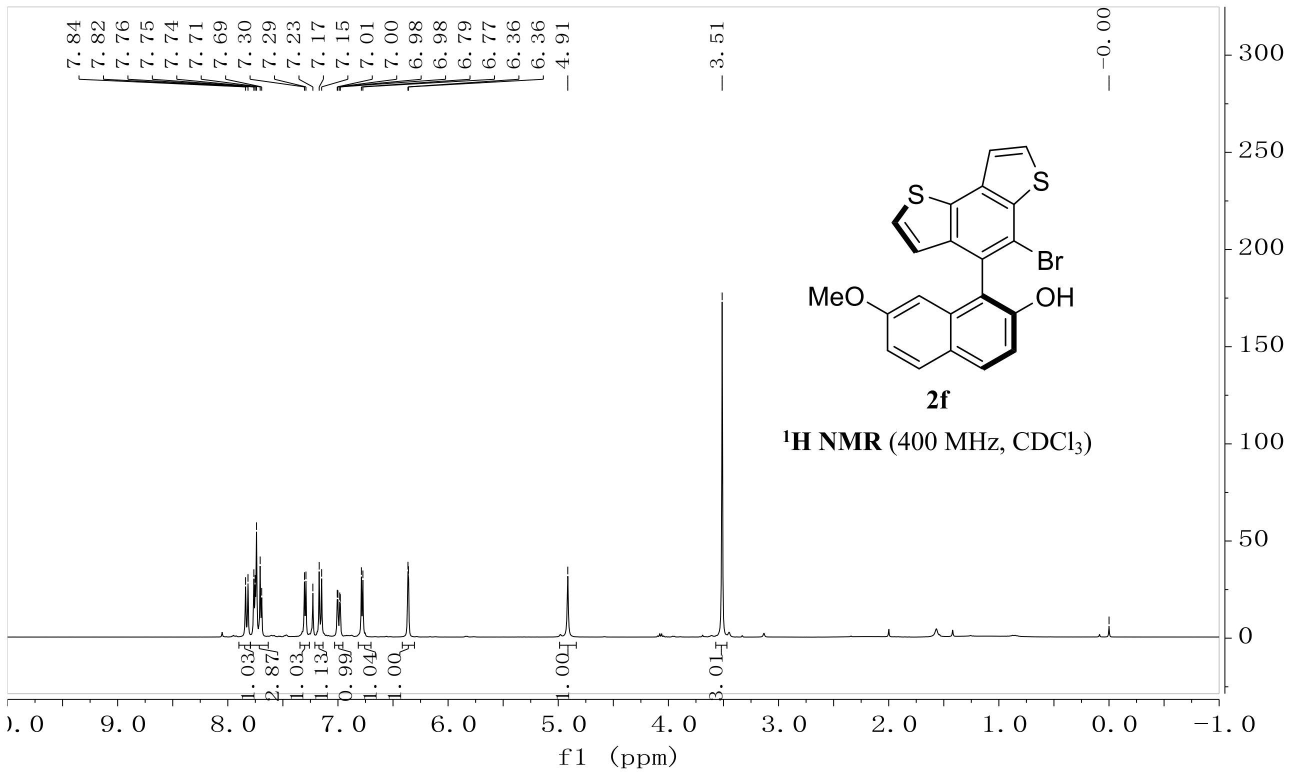
7.84 7.82 7.76 7.75 7.74 7.71 7.69 7.30 7.29 7.23 7.17 7.15 7.01 7.00 6.98 6.98 6.79 6.77 6.36 6.36 4.91
3.51
0.00
S
S
Br
MeO
OH
2f
1H NMR (400 MHz, CDCl3)
1.03 2.87 1.03 1.13 0.99 1.04 1.00
1.00
3.01
f1 (ppm)

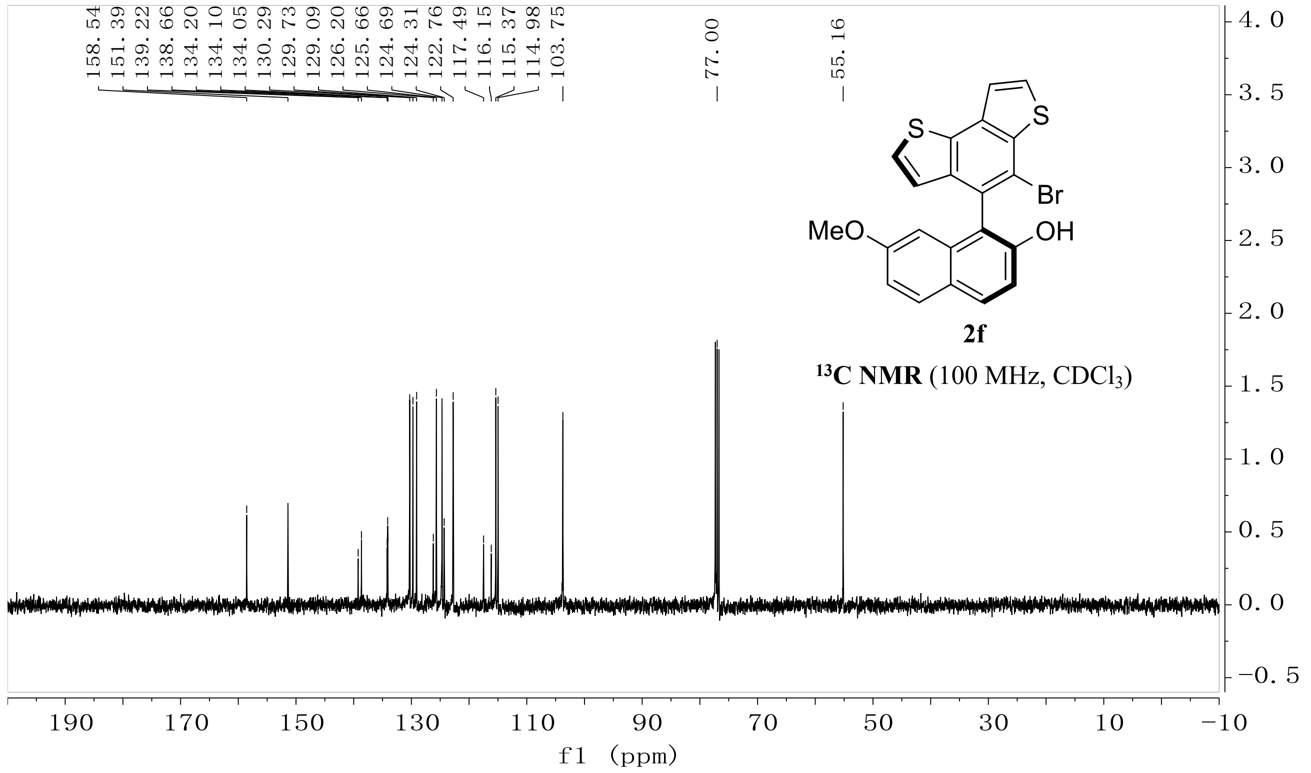
158.54 151.39 139.22 138.66 134.20 134.10 134.05 130.29 129.73 129.09 126.20 125.66 124.69 124.31 122.76 117.49 116.15 115.37 114.98 103.75
77.00
55.16
S
S
Br
MeO
OH
2f
13C NMR (100 MHz, CDCl3)
f1 (ppm)

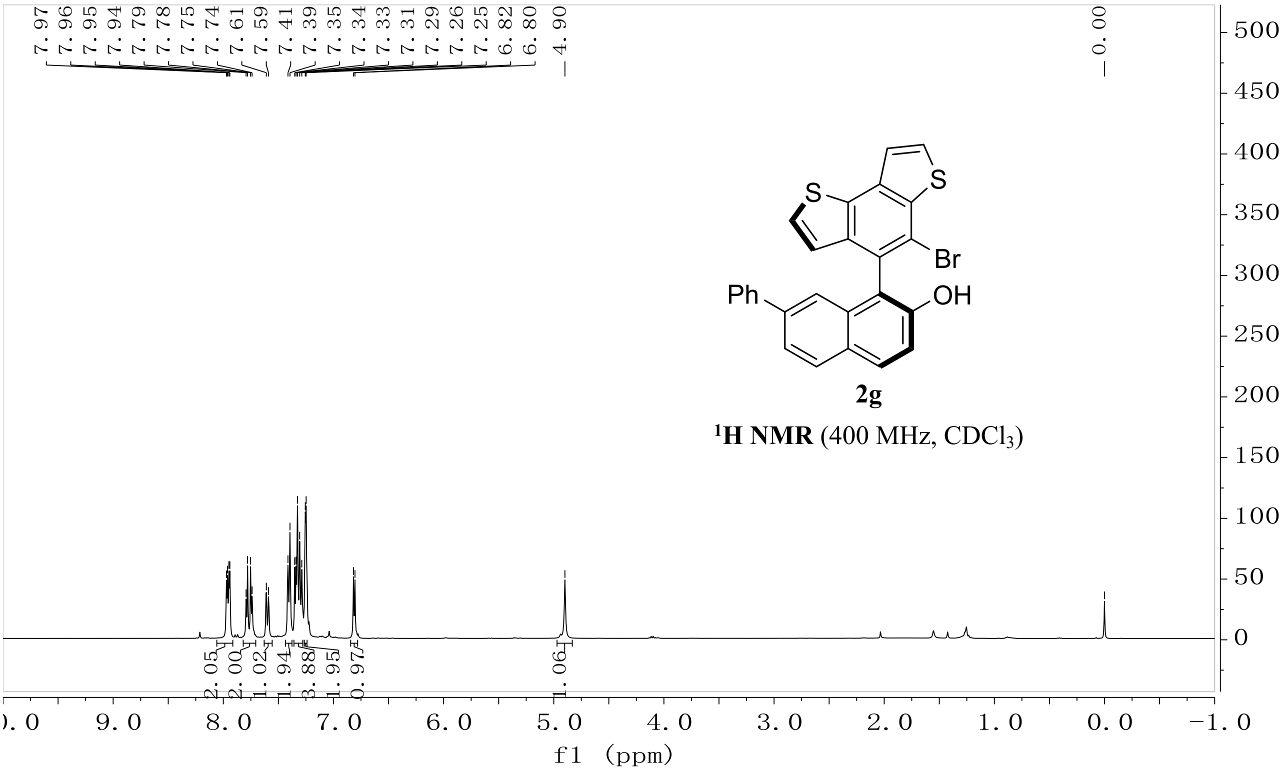

2g

^{1}H NMR (400 MHz, $CDCl_3$)

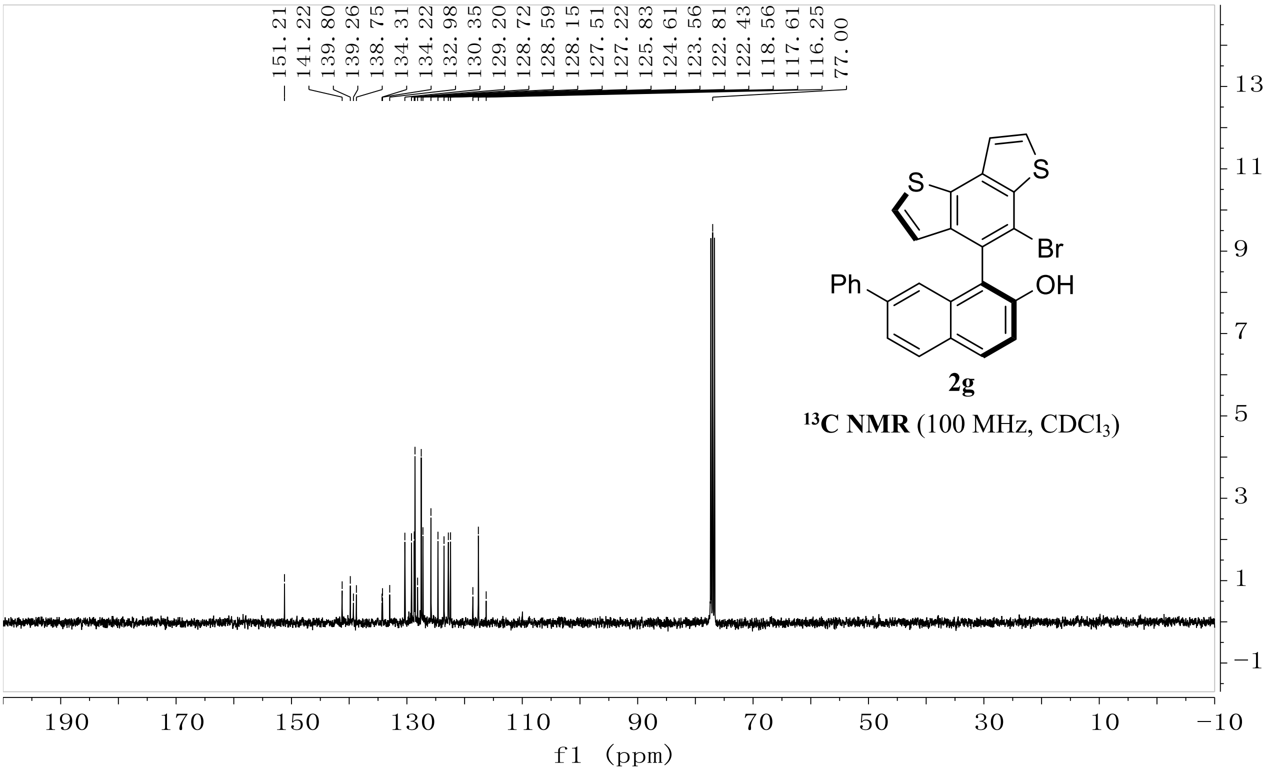

2g

^{13}C NMR (100 MHz, $CDCl_3$)

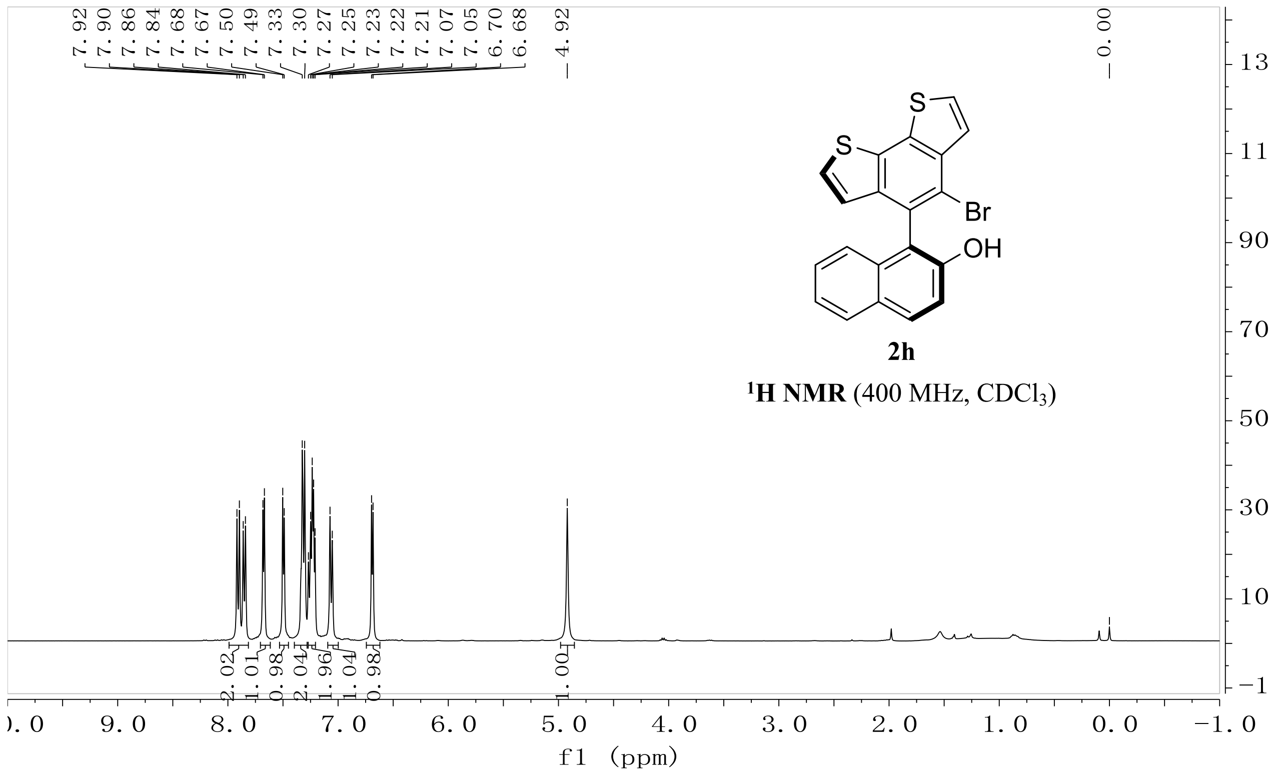

2h

^{1}H NMR (400 MHz, $CDCl_3$)

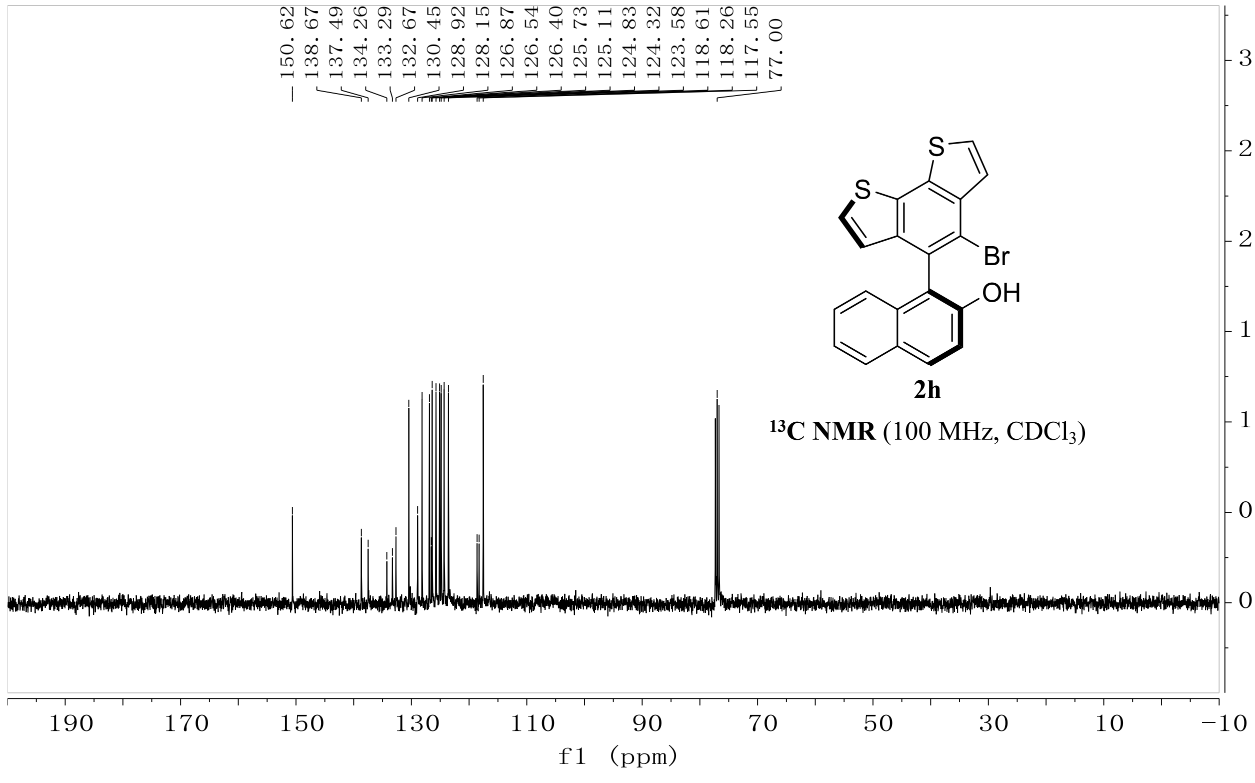

2h

^{13}C NMR (100 MHz, $CDCl_3$)

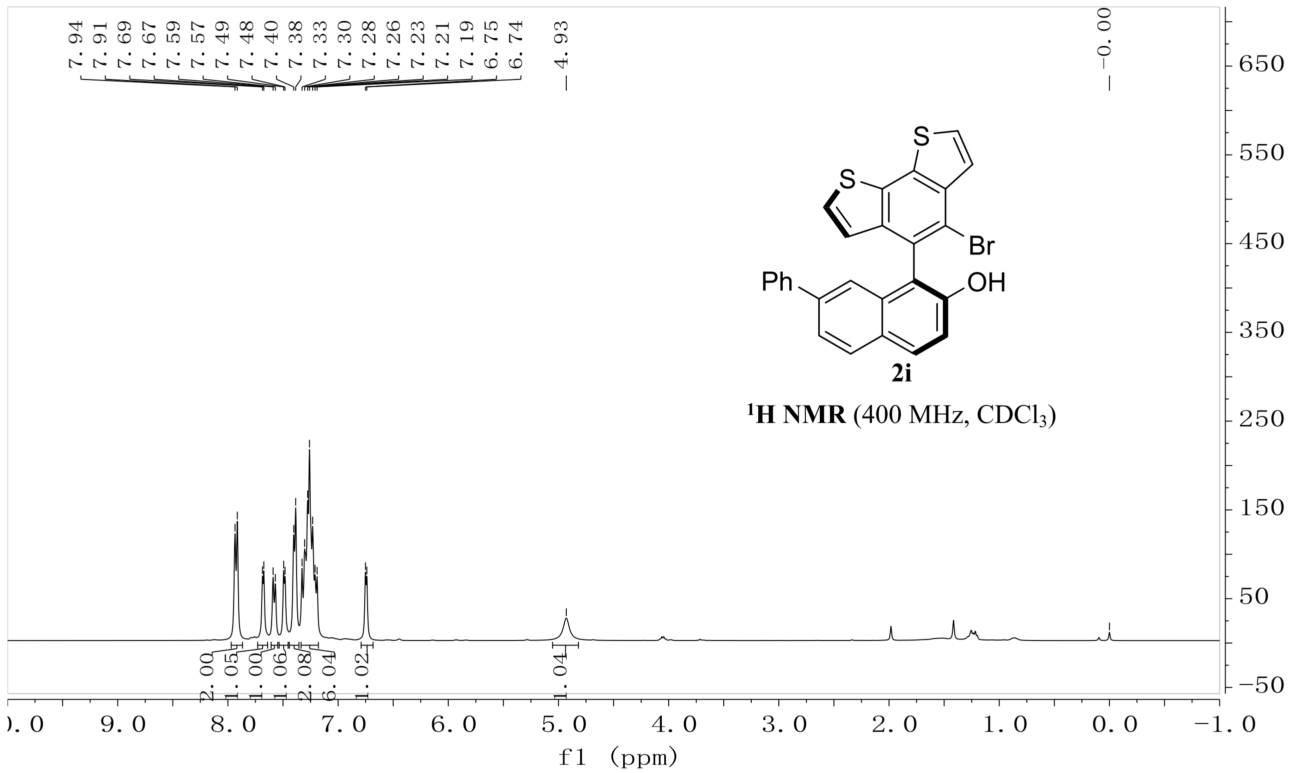
7.94
7.91
7.69
7.67
7.59
7.57
7.49
7.48
7.40
7.38
7.33
7.30
7.28
7.26
7.23
7.21
7.19
6.75
6.74
4.93
-0.00
S
S
Br
Ph
OH
2i
¹H NMR (400 MHz, CDCl₃)
2.00
1.05
1.00
1.06
2.08
6.04
1.02
1.04
f1 (ppm)

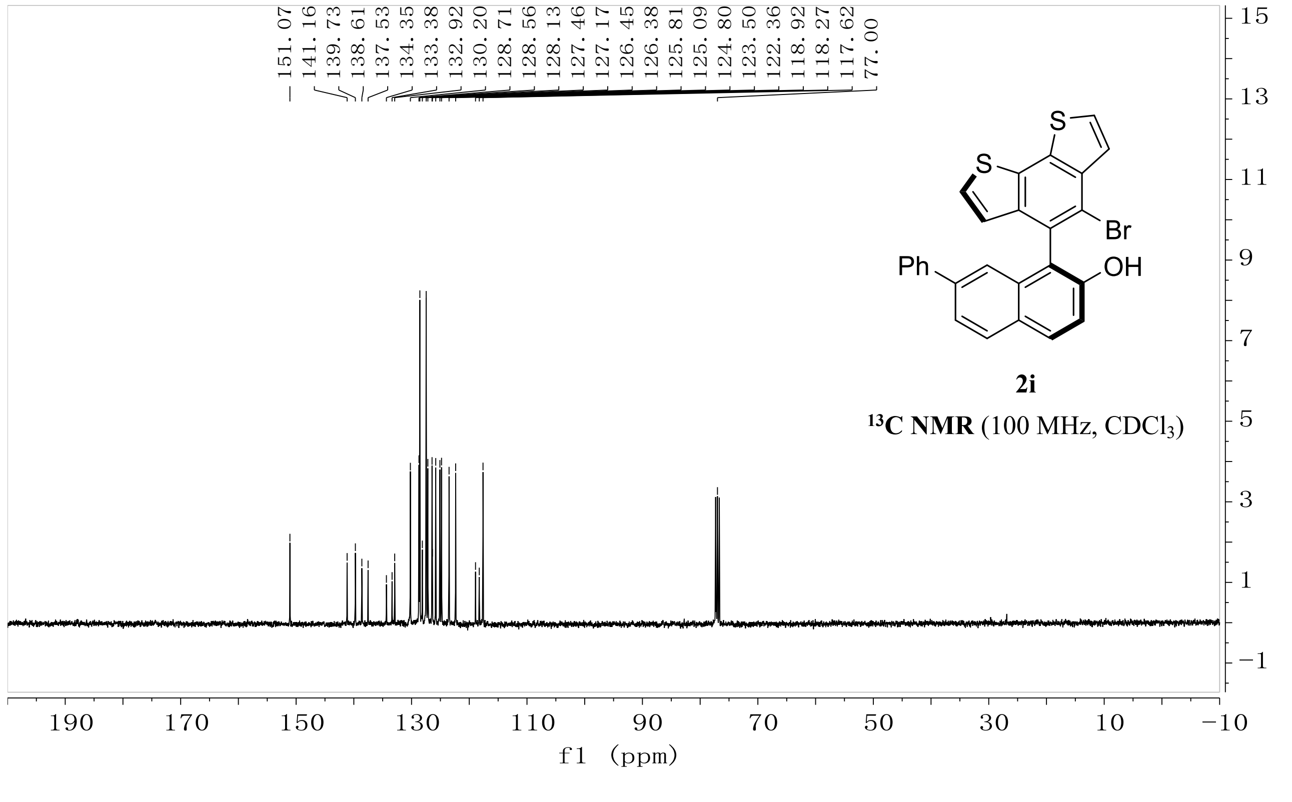
151.07
141.16
139.73
138.61
137.53
134.35
133.38
132.92
130.20
128.71
128.56
128.13
127.46
127.17
126.45
126.38
125.81
125.09
124.80
123.50
122.36
118.92
118.27
117.62
77.00
S
S
Br
Ph
OH
2i
¹³C NMR (100 MHz, CDCl₃)
f1 (ppm)

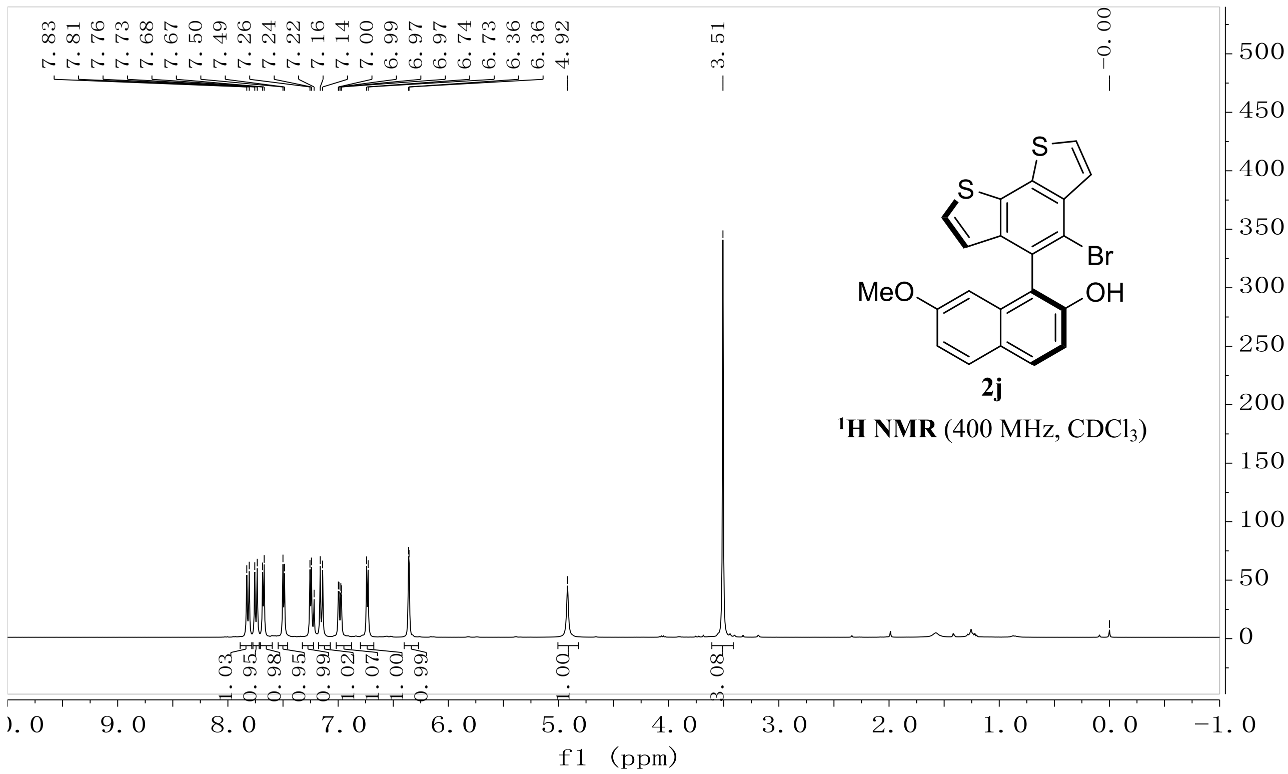

7.83 7.81 7.76 7.73 7.68 7.67 7.50 7.49 7.26 7.24 7.22 7.16 7.14 7.00 6.99 6.97 6.97 6.74 6.73 6.36 6.36 4.92
3.51
-0.00
MeO
OH
Br
S
S
2j
1H NMR (400 MHz, CDCl3)
1.03 0.95 0.98 0.95 0.99 1.02 1.07 1.00 0.99
1.00
3.08
f1 (ppm)

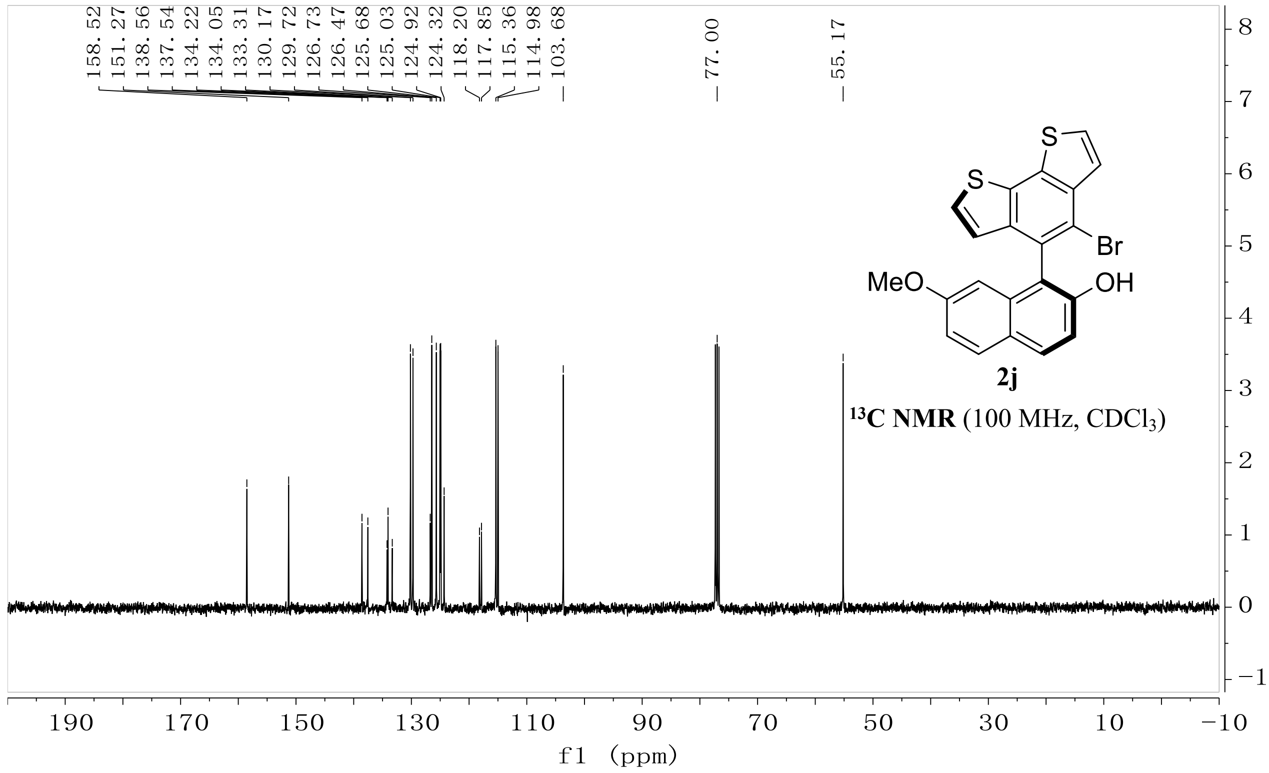

158.52 151.27 138.56 137.54 134.22 134.05 133.31 130.17 129.72 126.73 126.47 125.68 125.03 124.92 124.32 118.20 117.85 115.36 114.98 103.68
77.00
55.17
MeO
OH
Br
S
S
2j
13C NMR (100 MHz, CDCl3)
f1 (ppm)

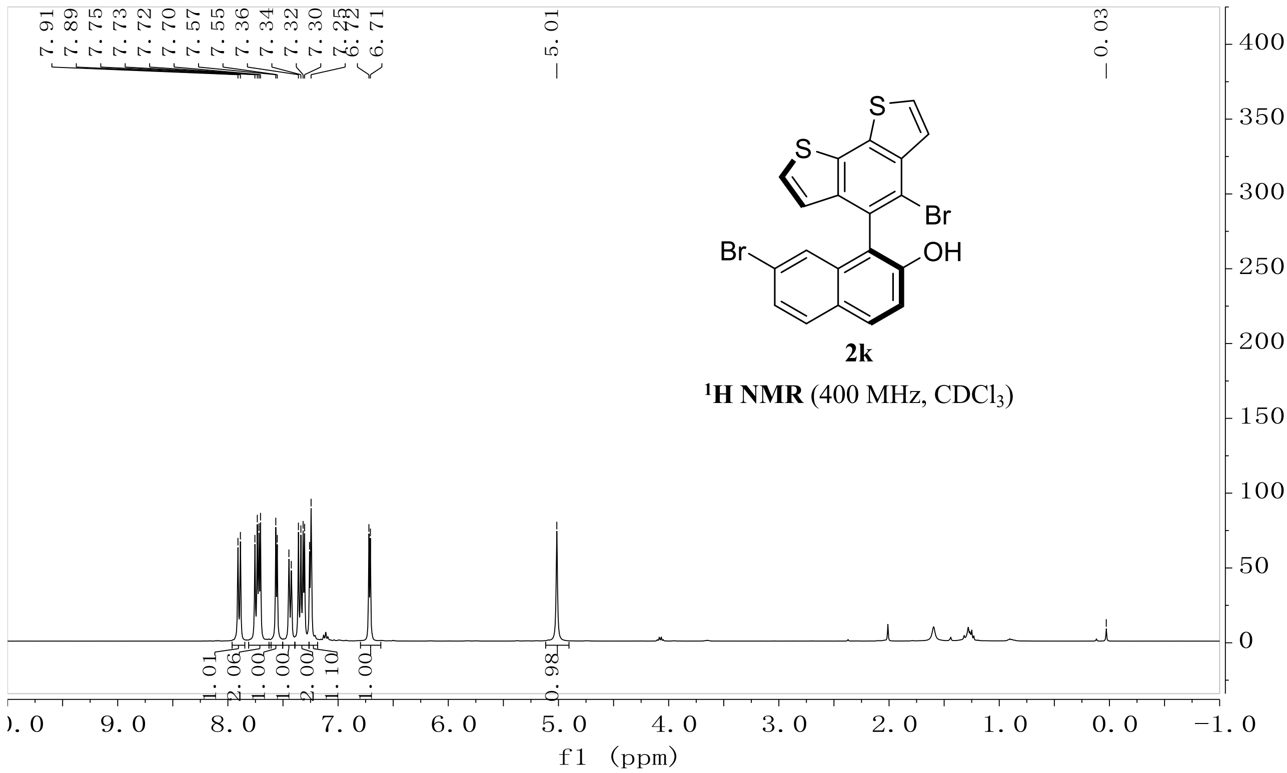
7.91
7.89
7.75
7.73
7.72
7.70
7.57
7.55
7.36
7.34
7.32
7.30
7.25
7.22
6.72
6.71
5.01
0.03
1.01
2.06
1.00
1.00
2.00
1.10
1.00
0.98
S
S
Br
Br
OH
2k
¹H NMR (400 MHz, CDCl3)
f1 (ppm)

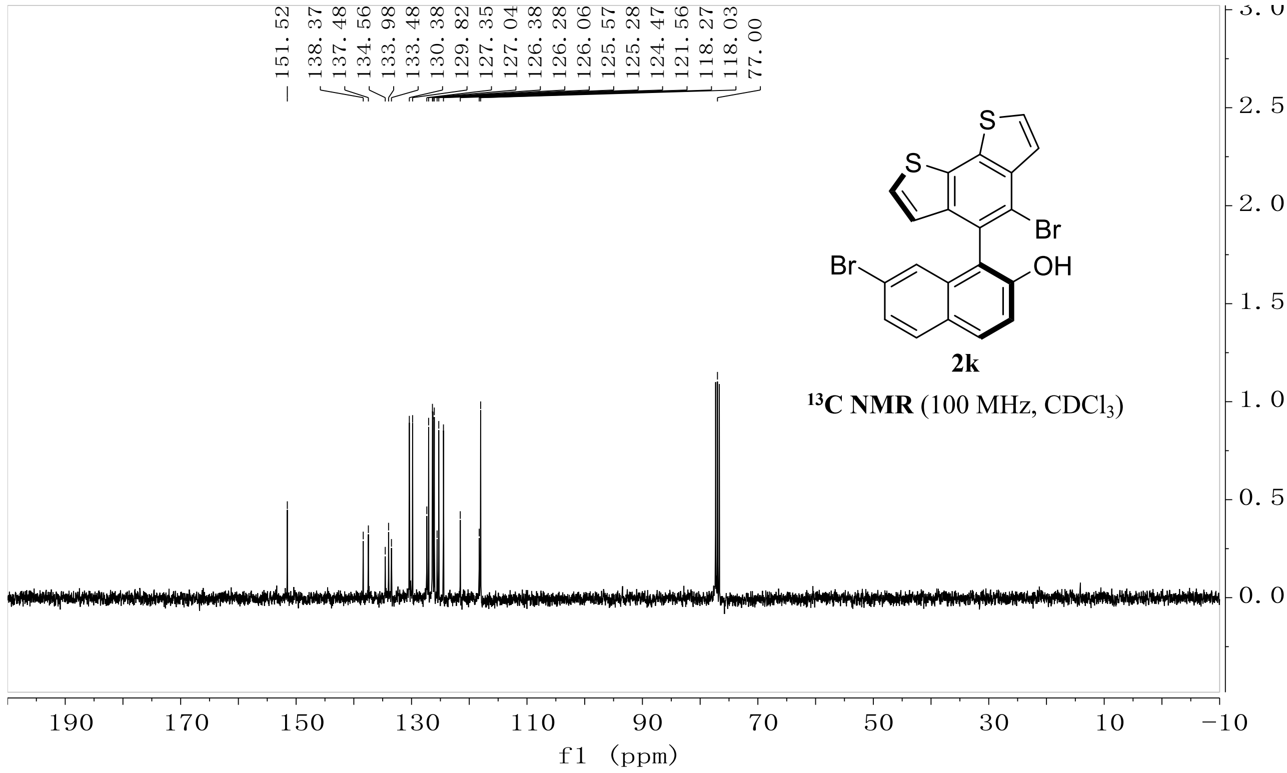
151.52
138.37
137.48
134.56
133.98
133.48
130.38
129.82
127.35
127.04
126.38
126.28
126.06
125.57
125.28
124.47
121.56
118.27
118.03
77.00
S
S
Br
Br
OH
2k
¹³C NMR (100 MHz, CDCl3)
f1 (ppm)

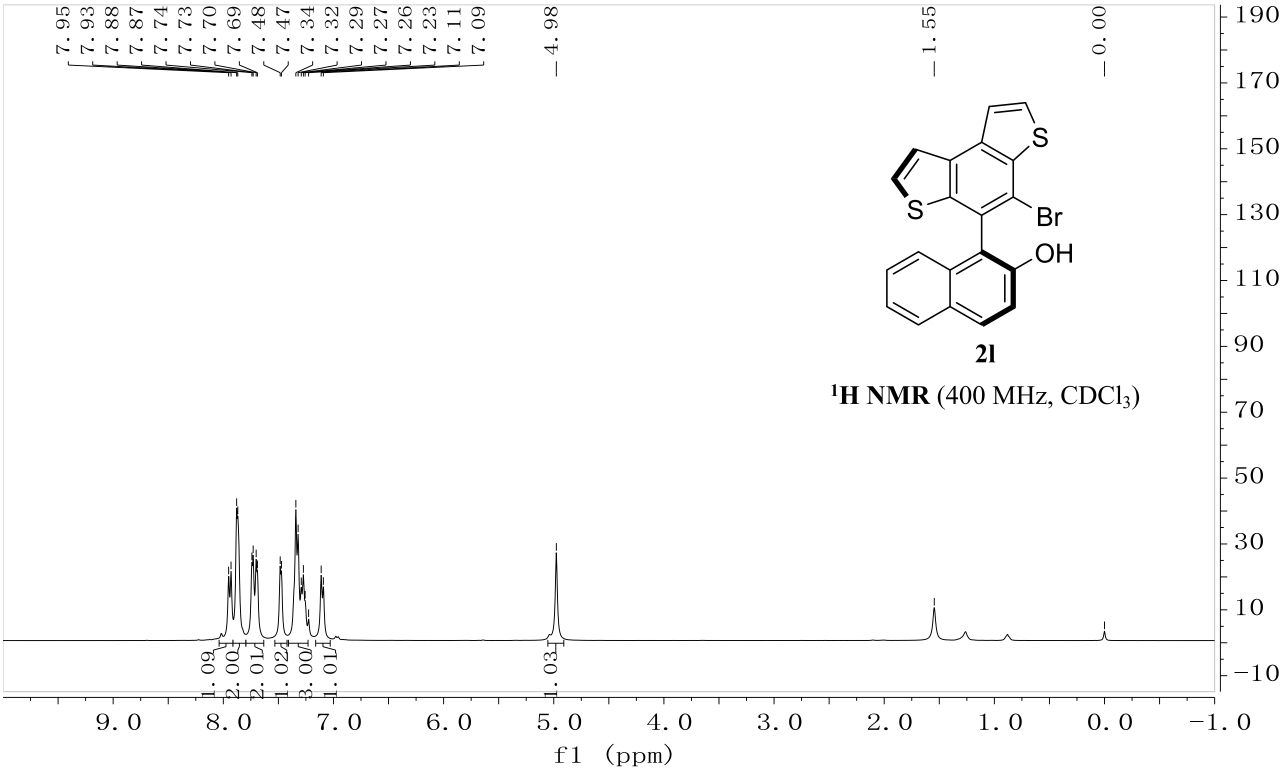

7.95 7.93 7.88 7.87 7.74 7.73 7.70 7.69 7.48 7.47 7.34 7.32 7.29 7.27 7.26 7.23 7.11 7.09
4.98
1.55
0.00
S
Br
OH
2l
^{1}H NMR (400 MHz, CDCl$_3$)
1.09 2.00 2.01 1.02 3.00 1.01
1.03
f1 (ppm)

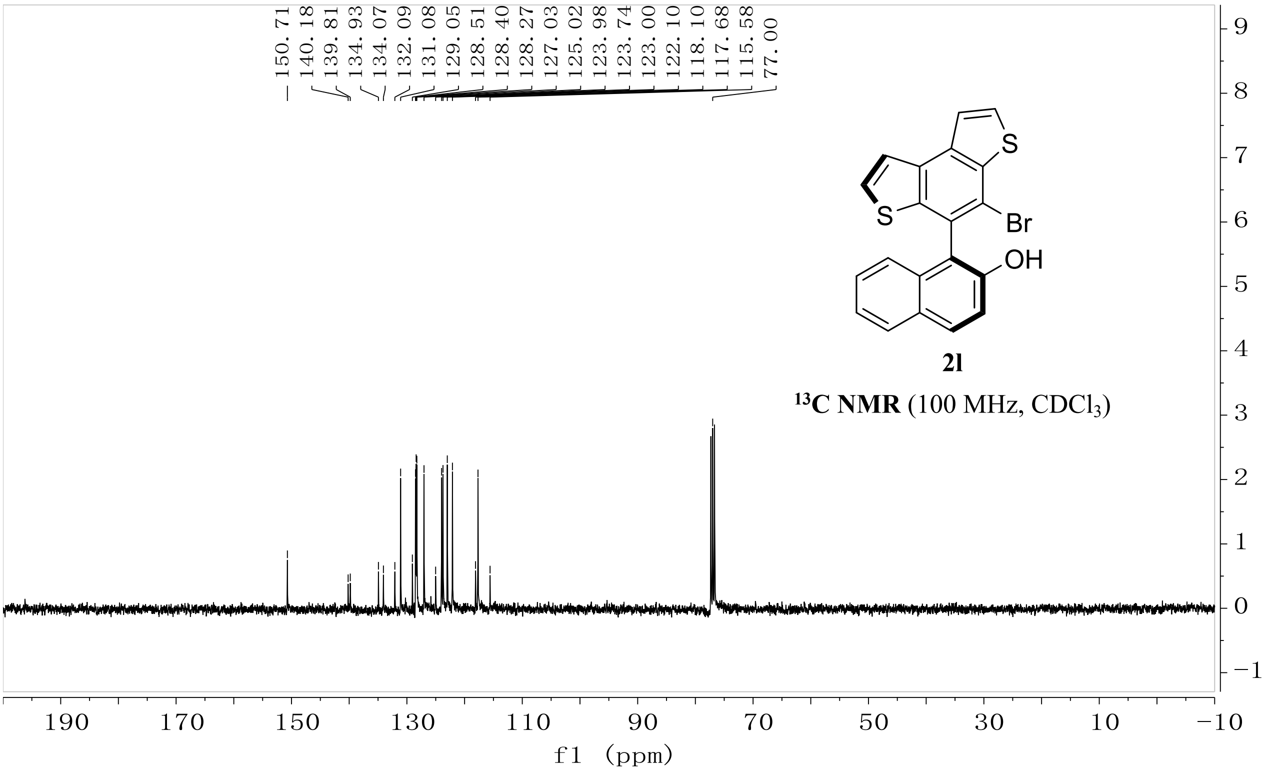

150.71 140.18 139.81 134.93 134.07 132.09 131.08 129.05 128.51 128.40 128.27 127.03 125.02 123.98 123.74 123.00 122.10 118.10 117.68 115.58 77.00
S
Br
OH
2l
^{13}C NMR (100 MHz, CDCl$_3$)
f1 (ppm)

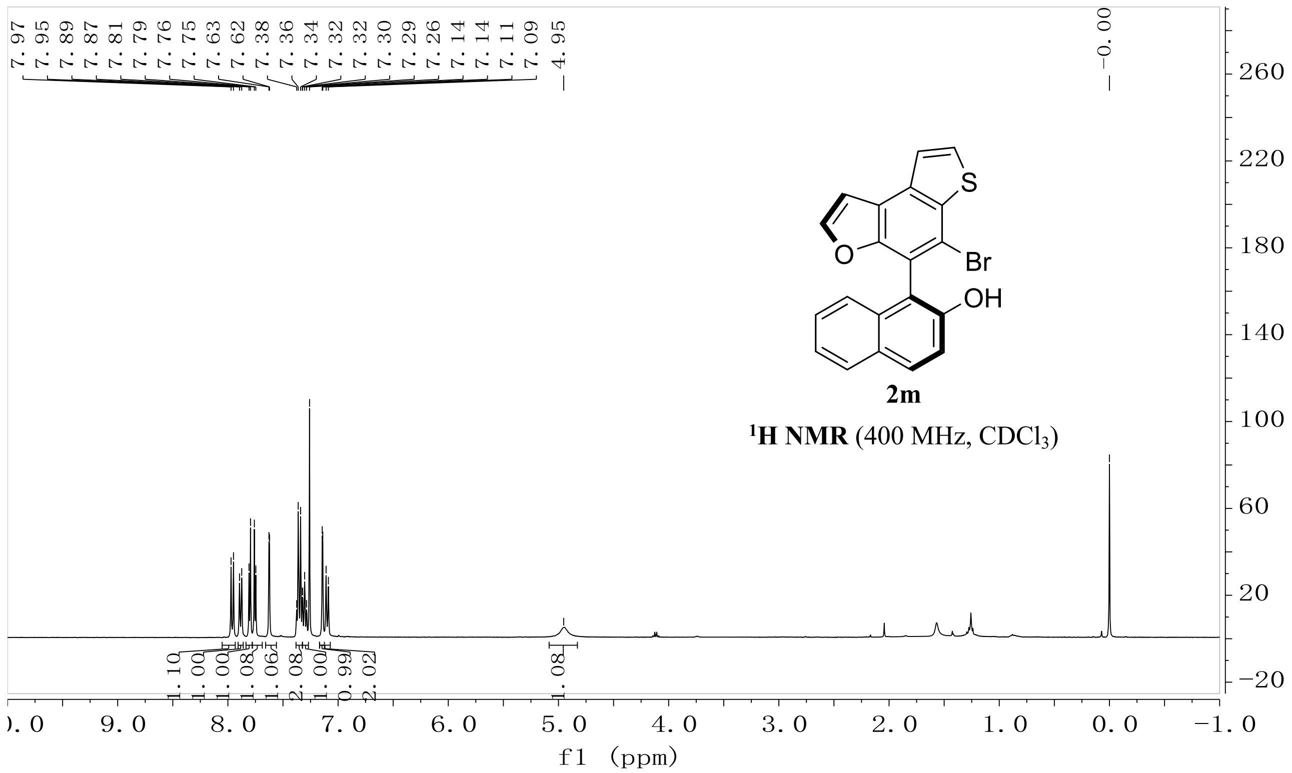

2m

^{1}H NMR (400 MHz, $CDCl_3$)

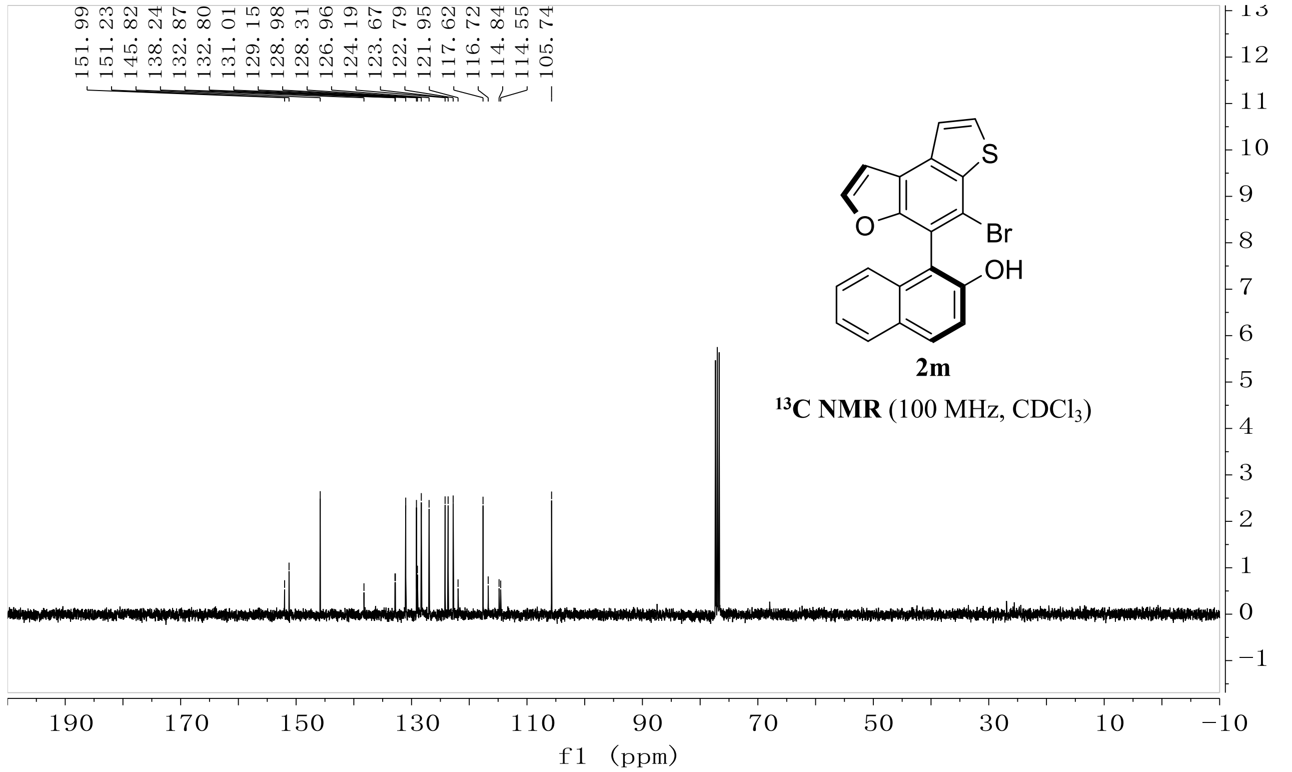

2m

^{13}C NMR (100 MHz, $CDCl_3$)

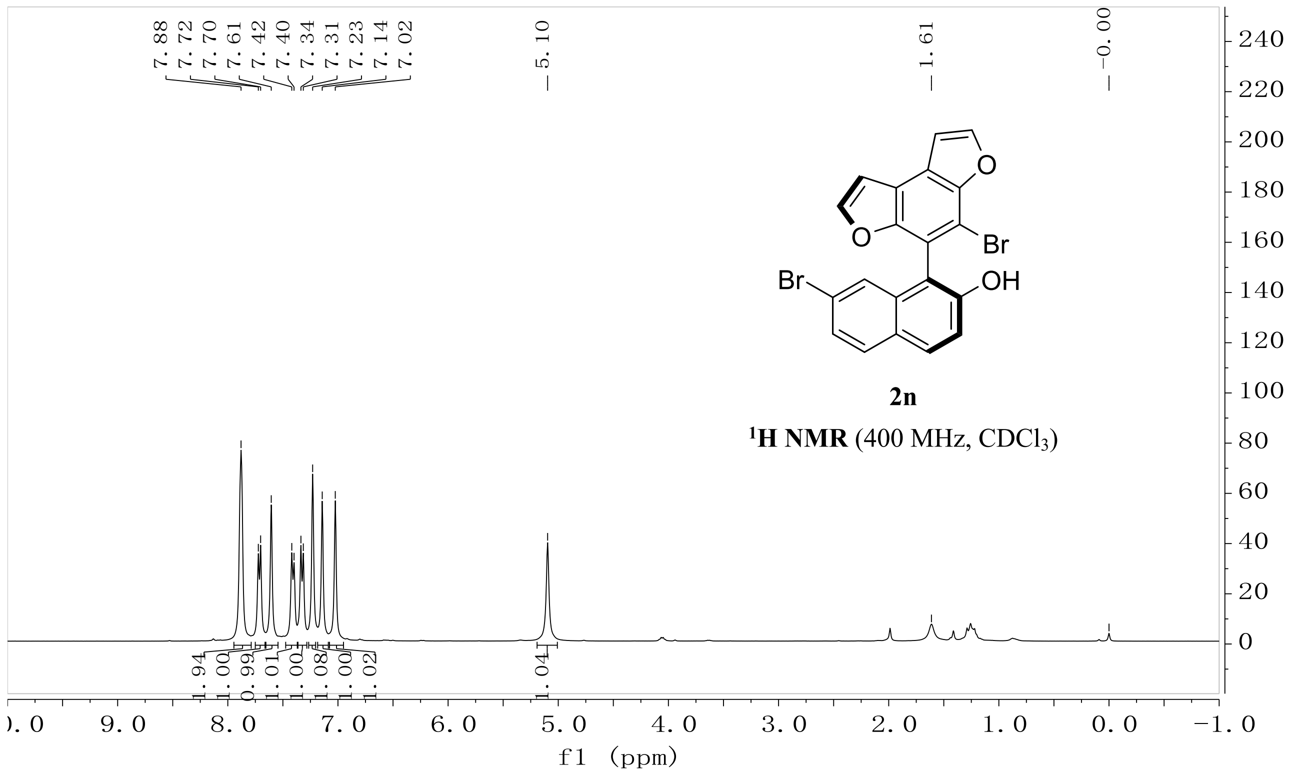
7.88
7.72
7.70
7.61
7.42
7.40
7.34
7.31
7.23
7.14
7.02
5.10
1.61
-0.00
Br
O
O
Br
OH
2n
1H NMR (400 MHz, CDCl3)
f1 (ppm)

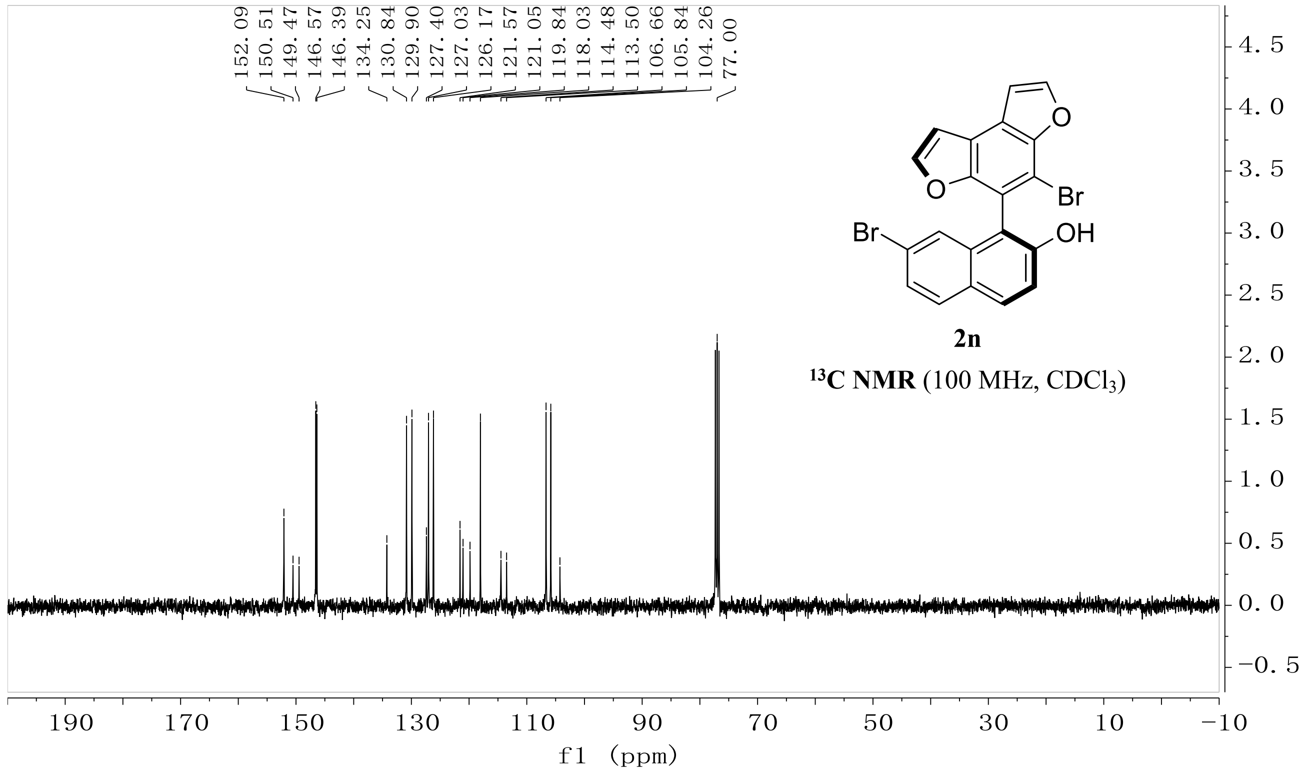
152.09
150.51
149.47
146.57
146.39
134.25
130.84
129.90
127.40
127.03
126.17
121.57
121.05
119.84
118.03
114.48
113.50
106.66
105.84
104.26
77.00
2n
13C NMR (100 MHz, CDCl3)
f1 (ppm)

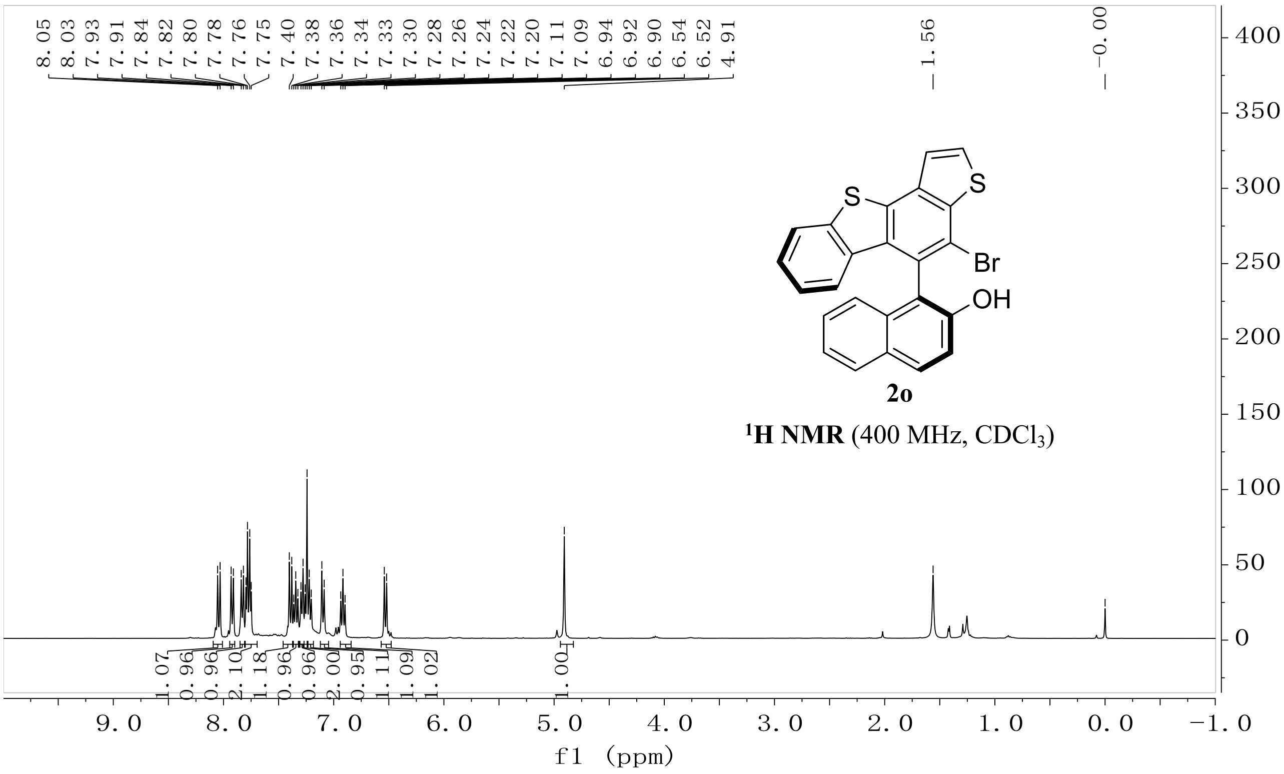

8.05 8.03 7.93 7.91 7.84 7.82 7.80 7.78 7.76 7.75 7.40 7.38 7.36 7.34 7.33 7.30 7.28 7.26 7.24 7.22 7.20 7.11 7.09 6.94 6.92 6.90 6.54 6.52 4.91
1.56
-0.00
S
S
Br
OH
2o
¹H NMR (400 MHz, CDCl₃)
1.07 0.96 0.96 2.10 1.18 0.96 0.96 2.00 0.95 1.11 1.09 1.02
1.00
f1 (ppm)

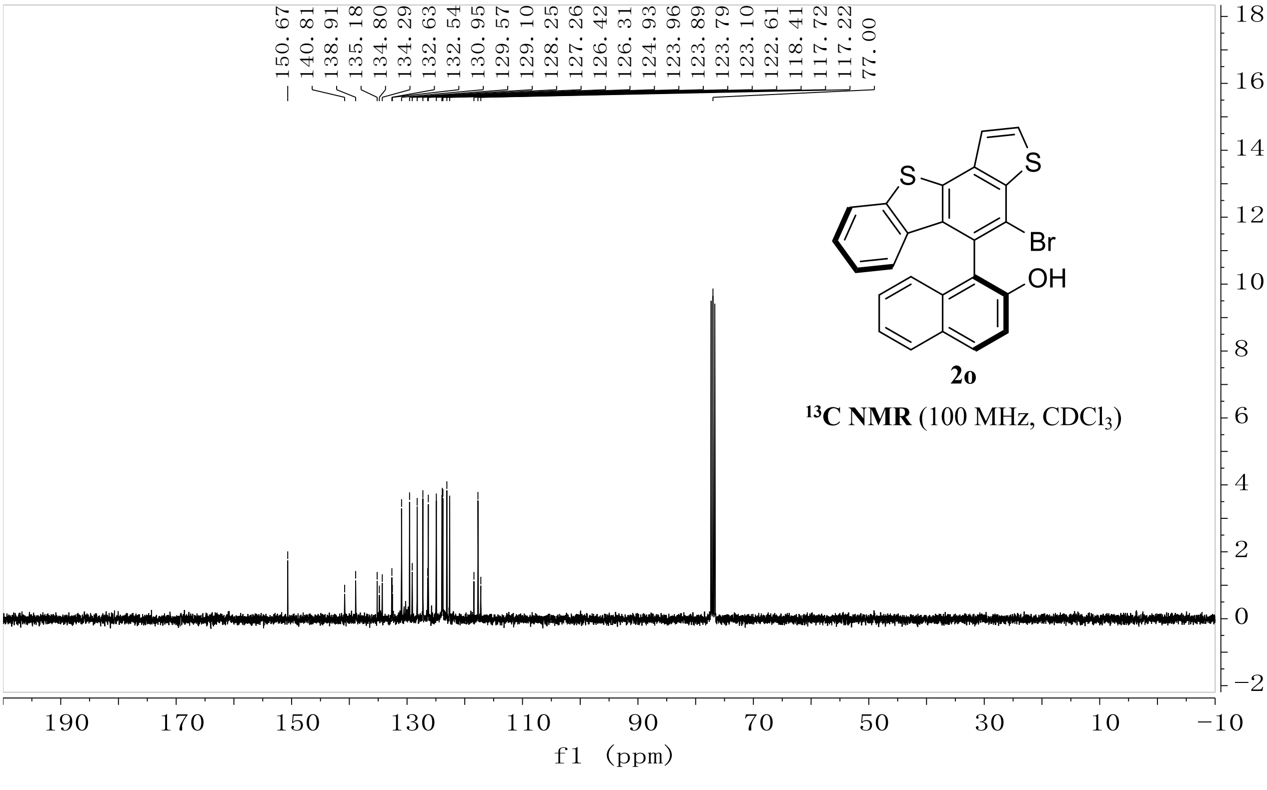

150.67 140.81 138.91 135.18 134.80 134.29 132.63 132.54 130.95 129.57 129.10 128.25 127.26 126.42 126.31 124.93 123.96 123.89 123.79 123.10 122.61 118.41 117.72 117.22 77.00
S
S
Br
OH
2o
¹³C NMR (100 MHz, CDCl₃)
f1 (ppm)

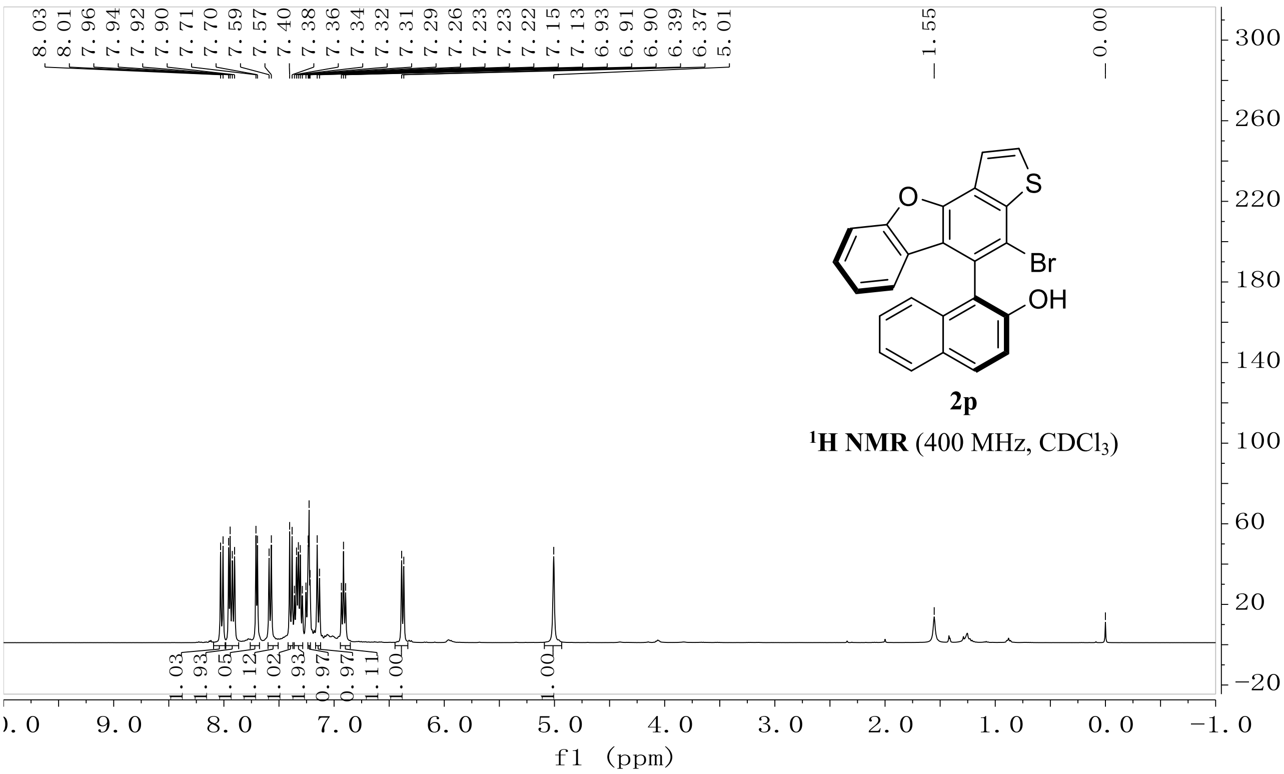

2p

^{1}H NMR (400 MHz, $CDCl_3$)

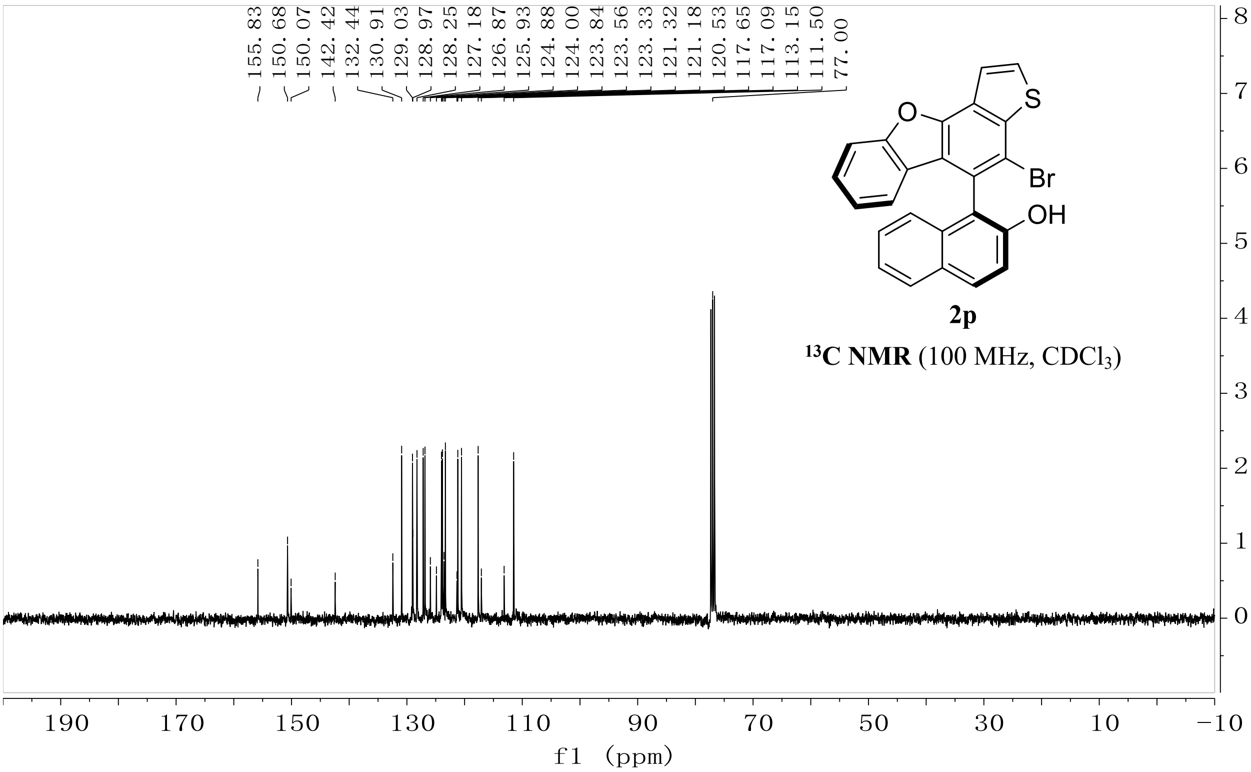

2p

^{13}C NMR (100 MHz, $CDCl_3$)

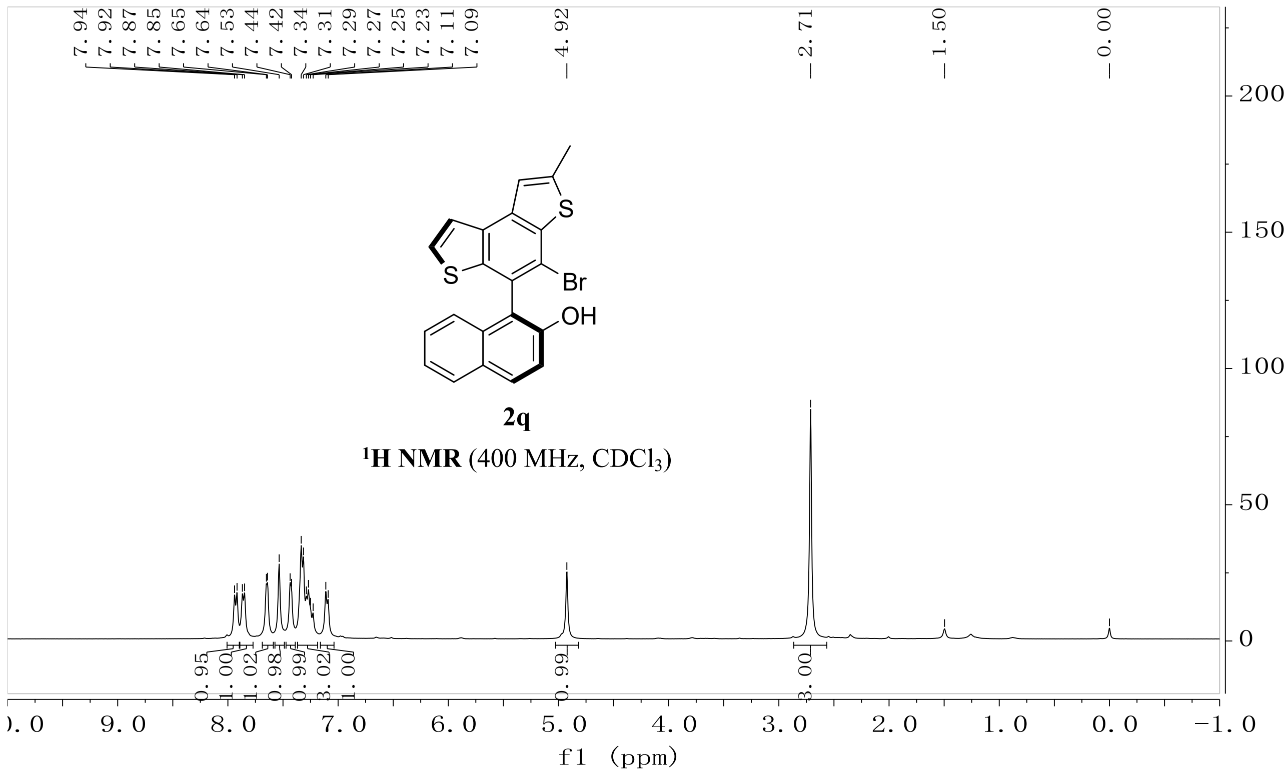
7.94
7.92
7.87
7.85
7.65
7.64
7.53
7.44
7.42
7.34
7.31
7.29
7.27
7.25
7.23
7.11
7.09
4.92
2.71
1.50
0.00
S
S
Br
OH
2q
1H NMR (400 MHz, CDCl3)
0.95
1.00
1.02
0.98
0.99
3.02
1.00
0.99
3.00
f1 (ppm)

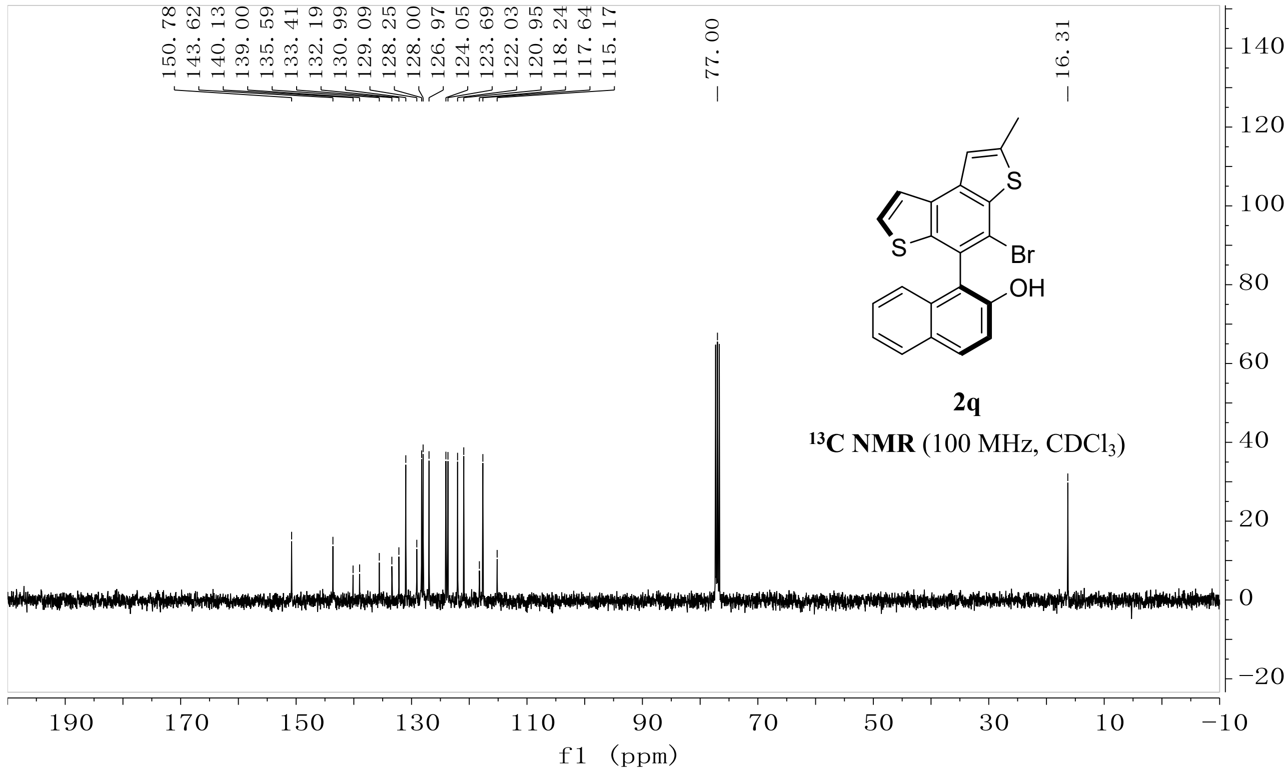
150.78
143.62
140.13
139.00
135.59
133.41
132.19
130.99
129.09
128.25
128.00
126.97
124.05
123.69
122.03
120.95
118.24
117.64
115.17
77.00
16.31
S
S
Br
OH
2q
13C NMR (100 MHz, CDCl3)
f1 (ppm)

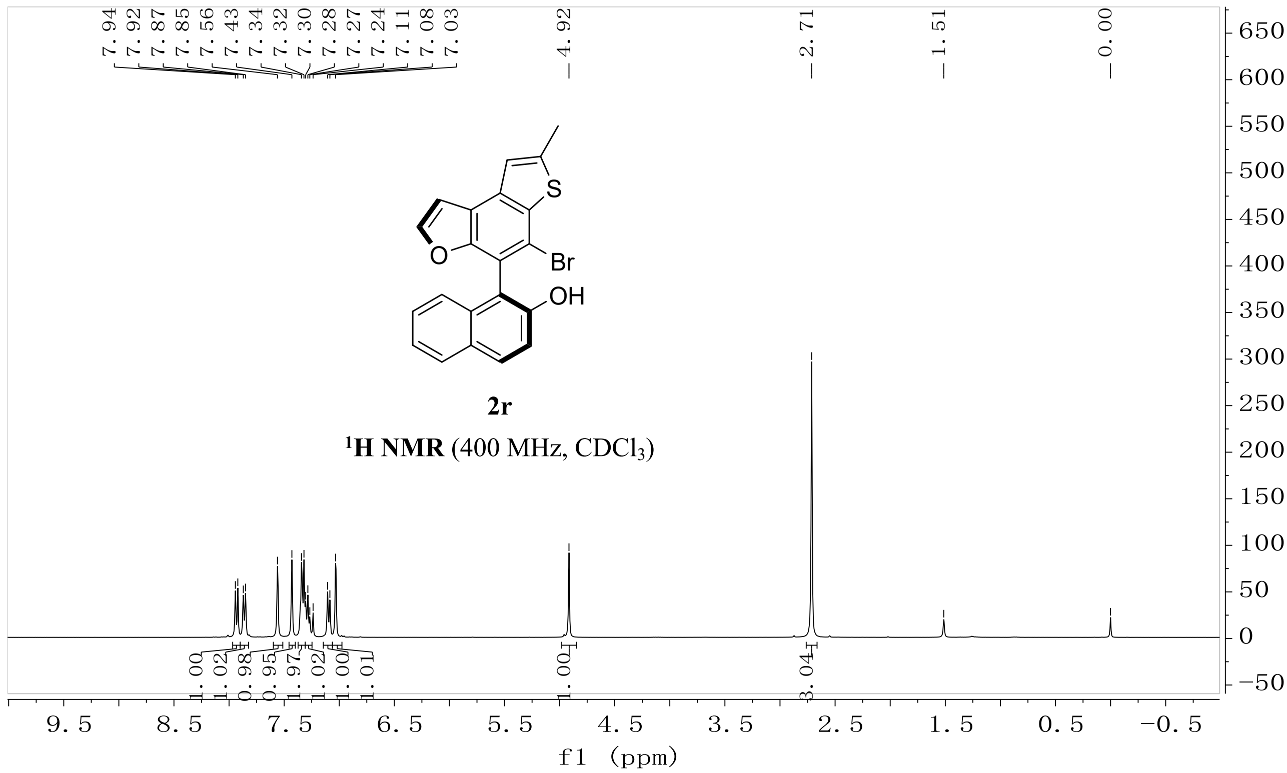
7.94 7.92 7.87 7.85 7.56 7.43 7.34 7.32 7.30 7.28 7.27 7.24 7.11 7.08 7.03
4.92
2.71
1.51
0.00
S
Br
O
OH
2r
1H NMR (400 MHz, CDCl3)
1.00 1.02 0.98 0.95 1.97 1.02 1.00 1.01
1.00
3.04
f1 (ppm)

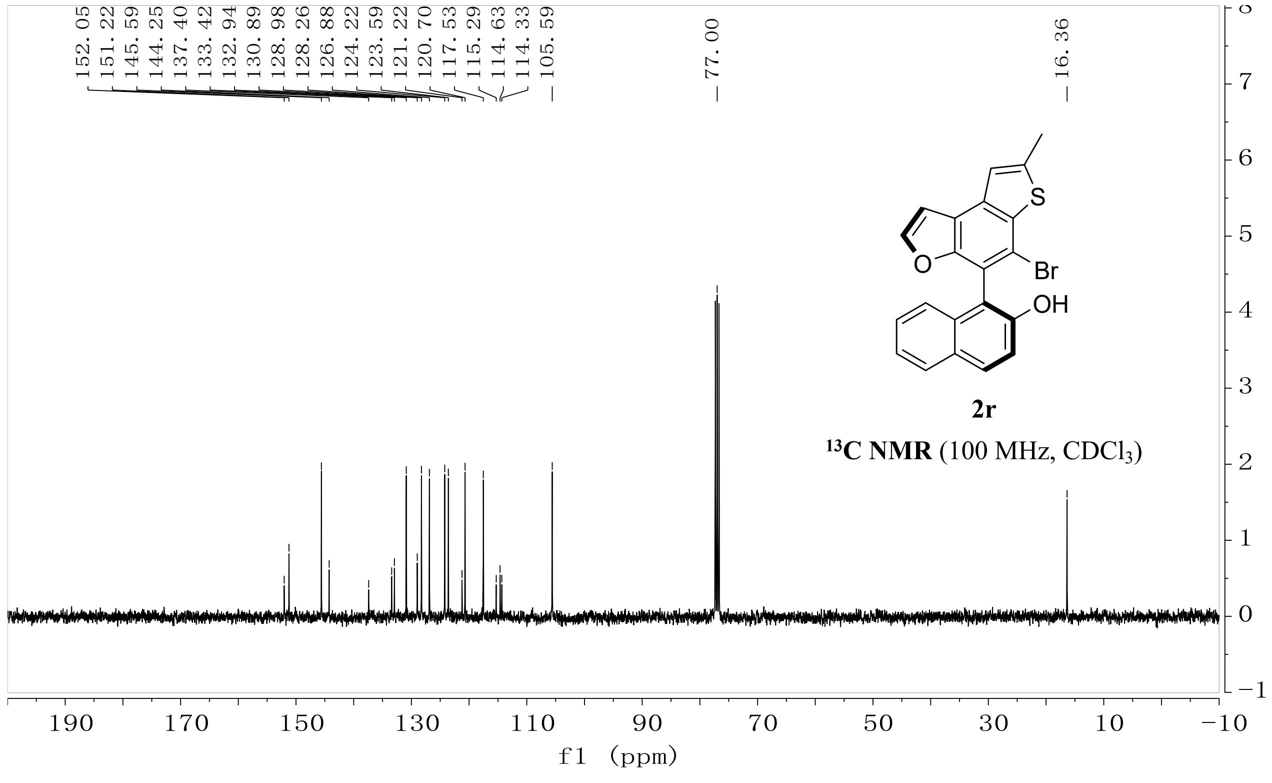
152.05 151.22 145.59 144.25 137.40 133.42 132.94 130.89 128.98 128.26 126.88 124.22 123.59 121.22 120.70 117.53 115.29 114.63 114.33 105.59
77.00
16.36
S
Br
O
OH
2r
13C NMR (100 MHz, CDCl3)
f1 (ppm)

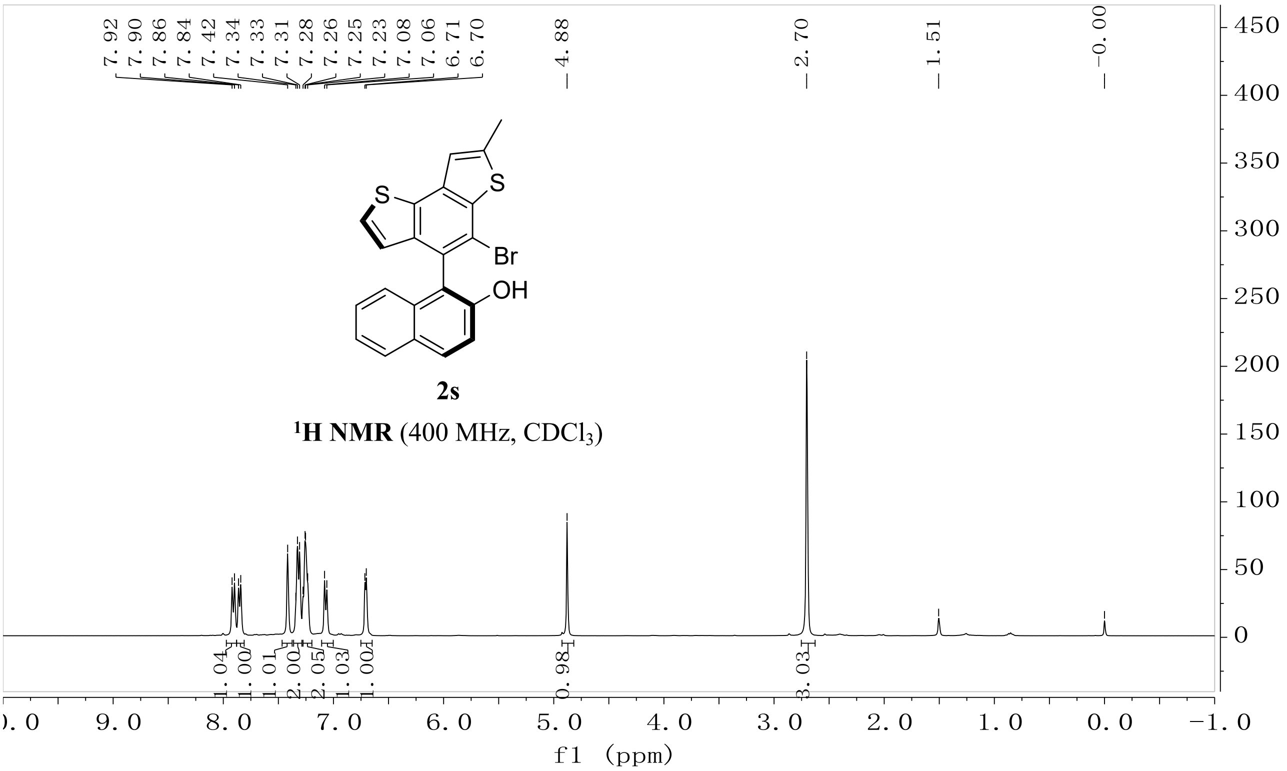

2s

¹H NMR (400 MHz, $CDCl_3$)

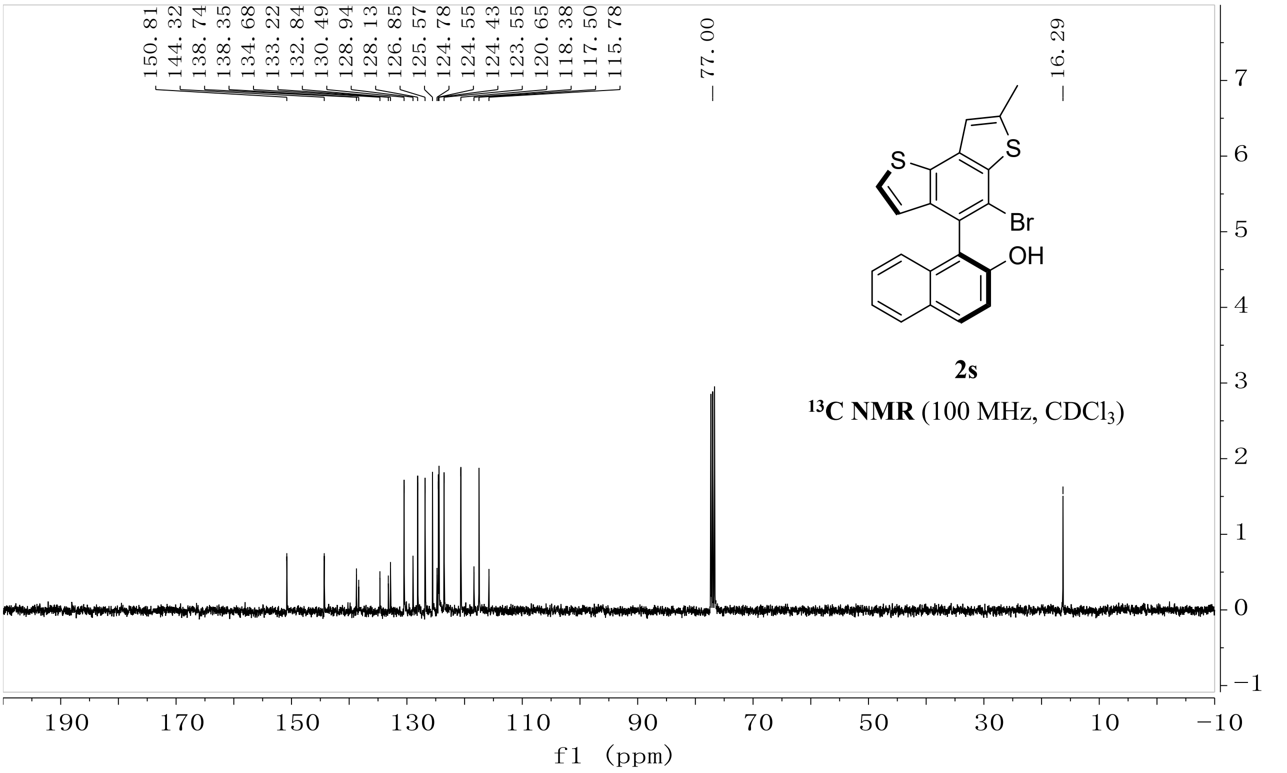

2s

¹³C NMR (100 MHz, $CDCl_3$)

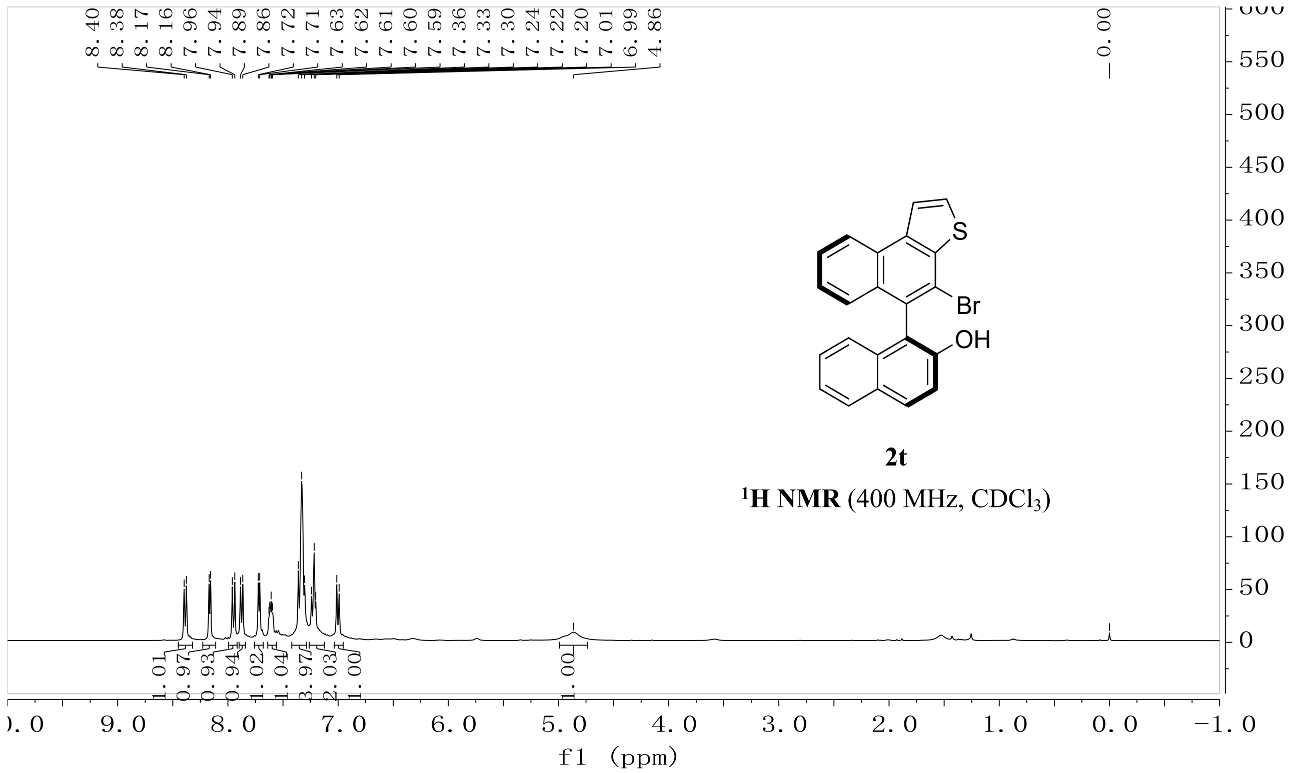

2t

^{1}H NMR (400 MHz, $CDCl_3$)

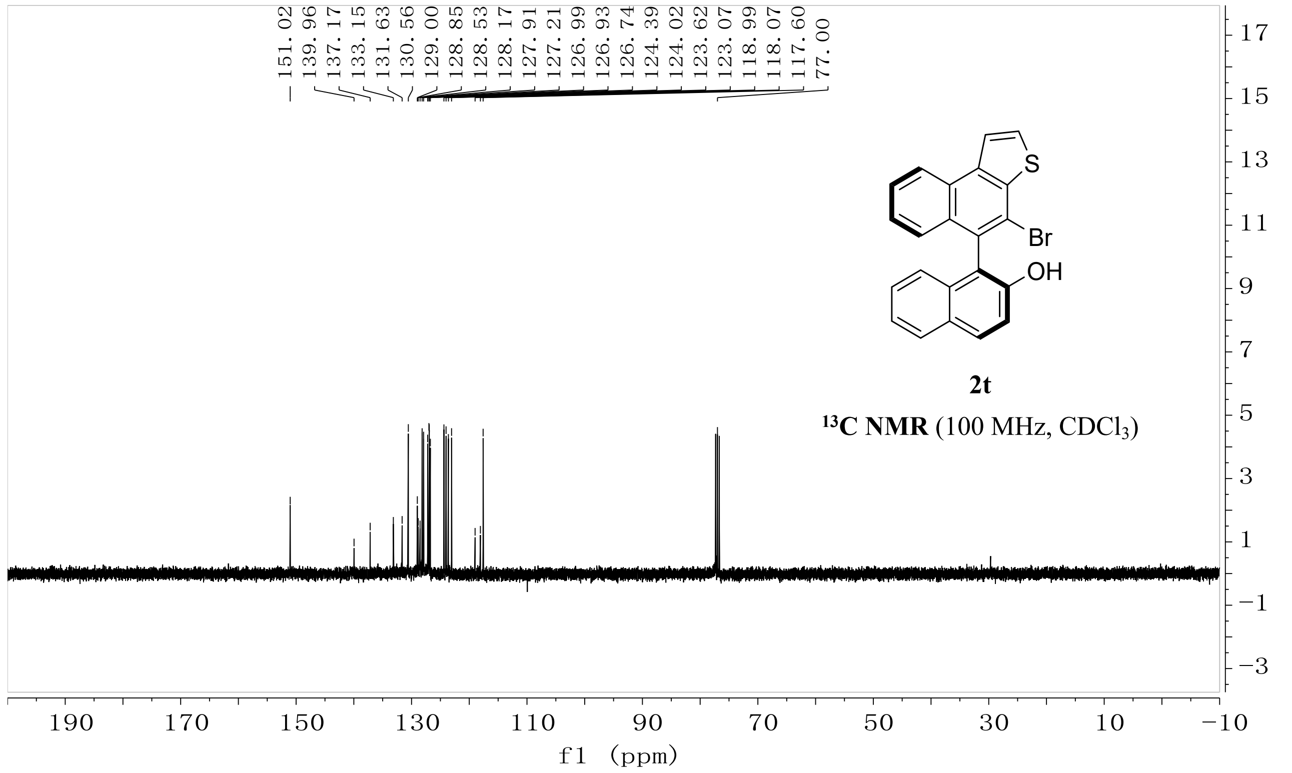

2t

^{13}C NMR (100 MHz, $CDCl_3$)

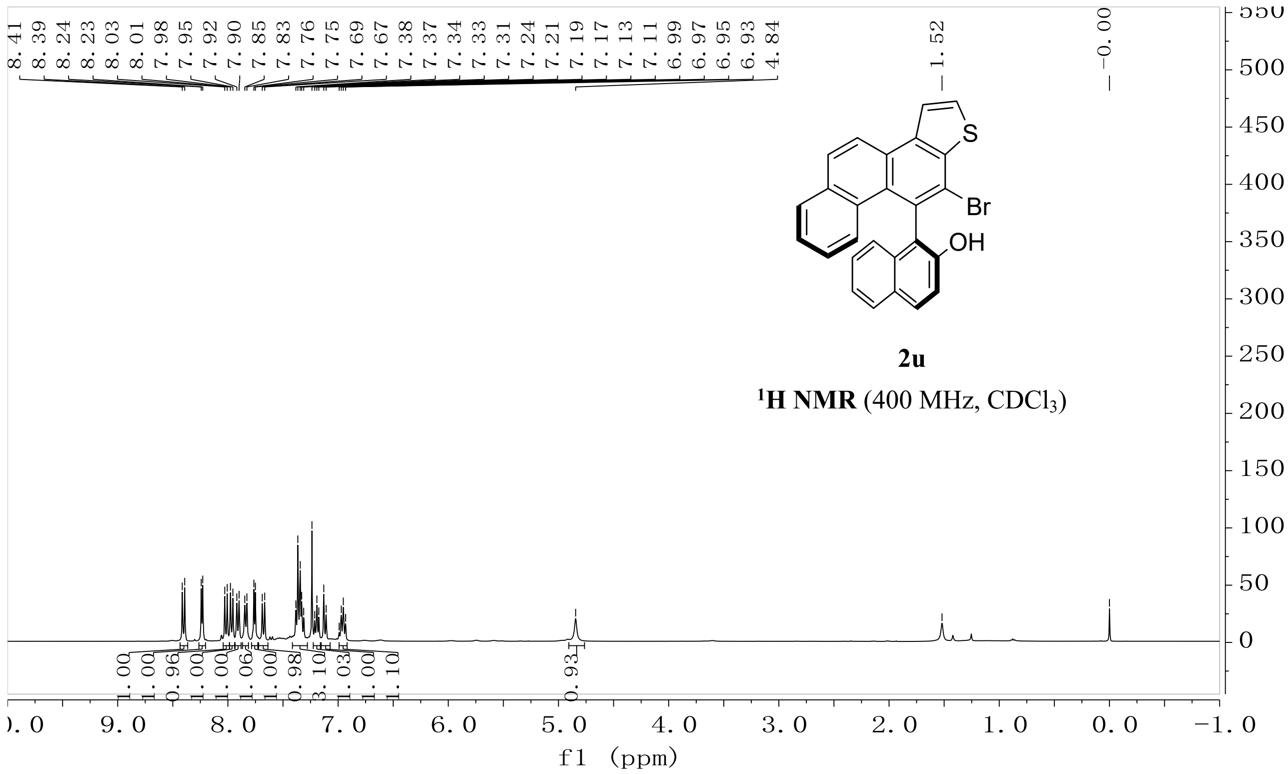
8.41
8.39
8.24
8.23
8.03
8.01
7.98
7.95
7.92
7.90
7.85
7.83
7.76
7.75
7.69
7.67
7.38
7.37
7.34
7.33
7.31
7.24
7.21
7.19
7.17
7.13
7.11
6.99
6.97
6.95
6.93
4.84
1.52
-0.00
S
Br
OH
2u
1H NMR (400 MHz, CDCl3)
1.00
1.00
0.96
1.00
1.00
1.06
1.00
0.98
3.10
1.03
1.00
1.10
0.93
f1 (ppm)

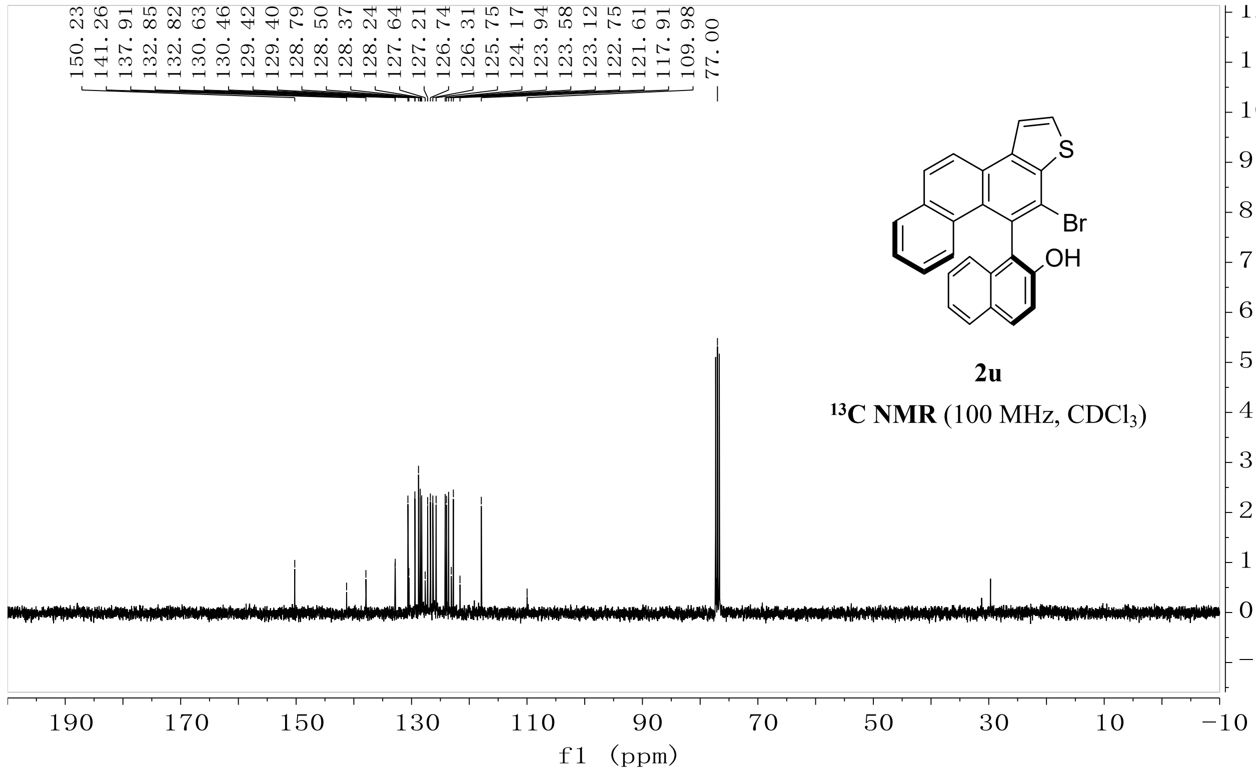
150.23
141.26
137.91
132.85
132.82
130.63
130.46
129.42
129.40
128.79
128.50
128.37
128.24
127.64
127.21
126.74
126.31
125.75
124.17
123.94
123.58
123.12
122.75
121.61
117.91
109.98
77.00
S
Br
OH
2u
13C NMR (100 MHz, CDCl3)
f1 (ppm)

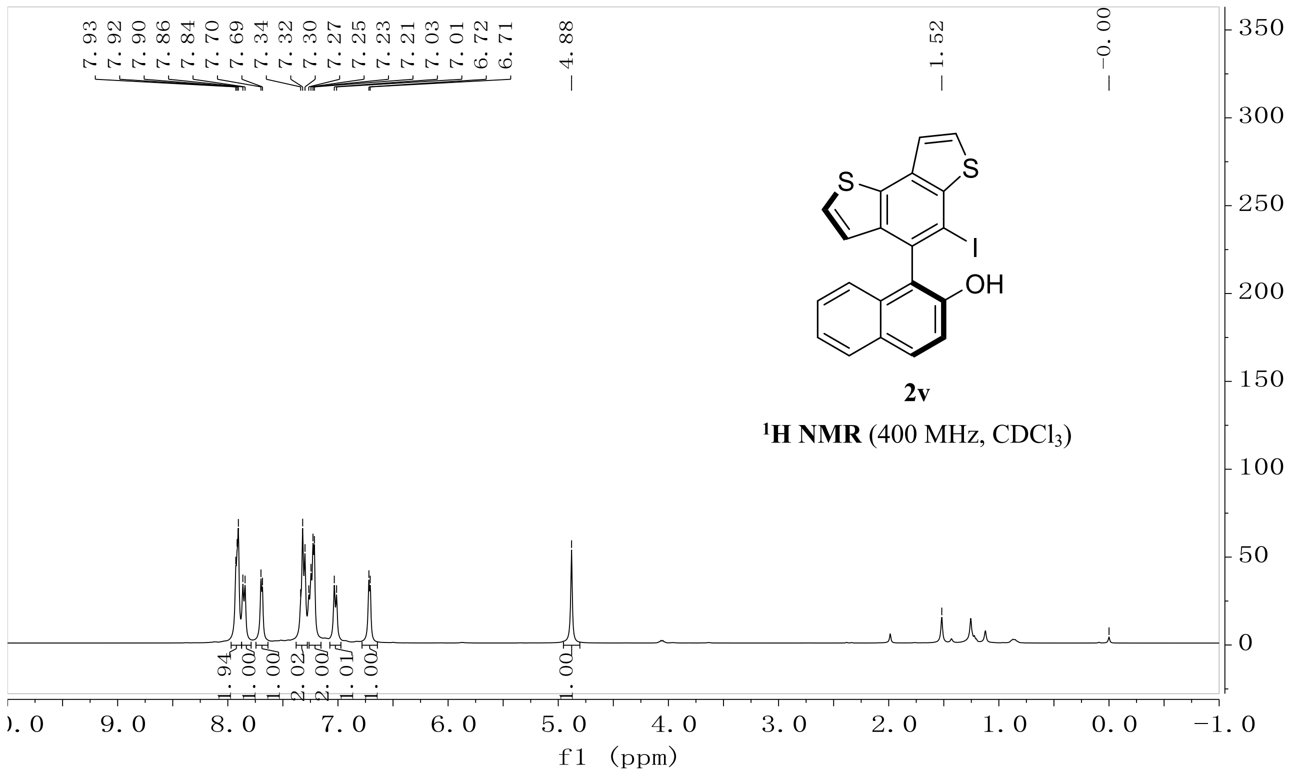
7.93
7.92
7.90
7.86
7.84
7.70
7.69
7.34
7.32
7.30
7.27
7.25
7.23
7.21
7.03
7.01
6.72
6.71
4.88
1.52
-0.00
2v
¹H NMR (400 MHz, CDCl₃)
f1 (ppm)

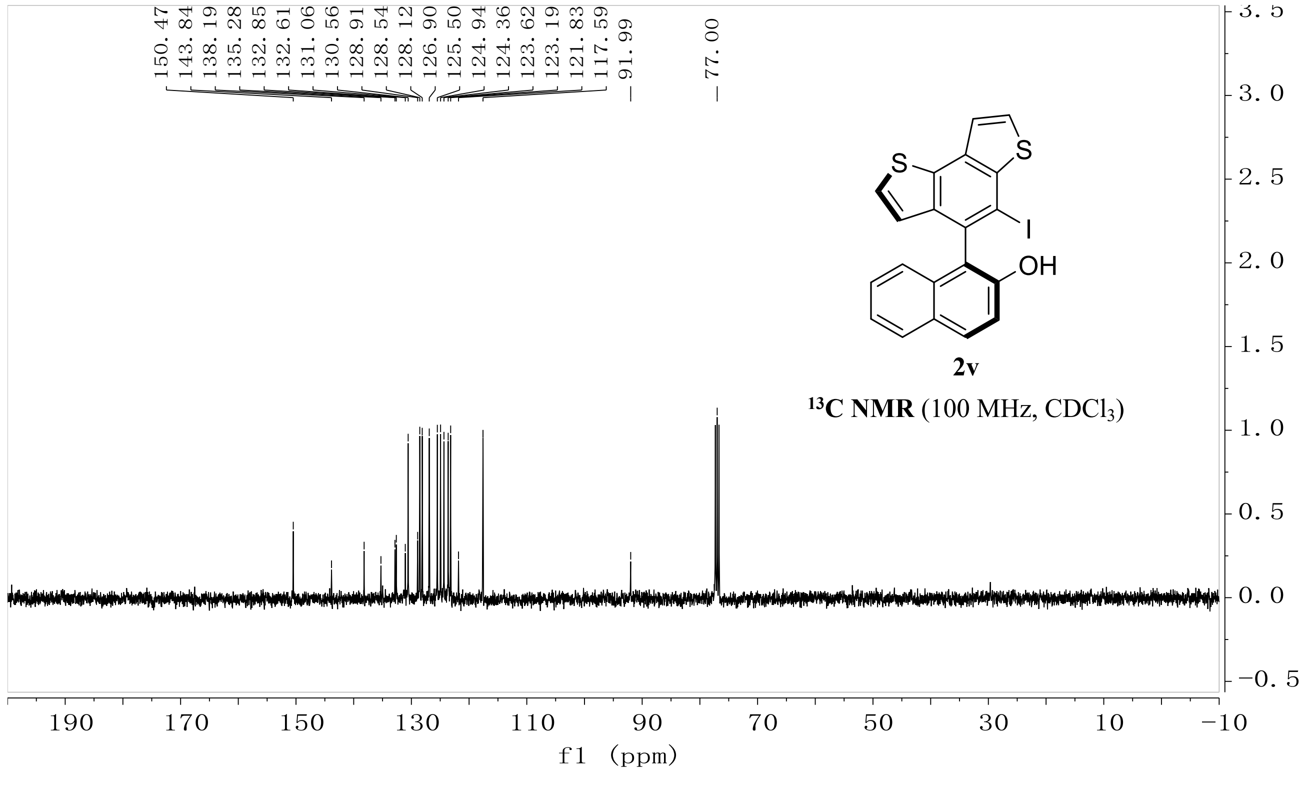
150.47
143.84
138.19
135.28
132.85
132.61
131.06
130.56
128.91
128.54
128.12
126.90
125.50
124.94
124.36
123.62
123.19
121.83
117.59
91.99
77.00
2v
¹³C NMR (100 MHz, CDCl₃)
f1 (ppm)

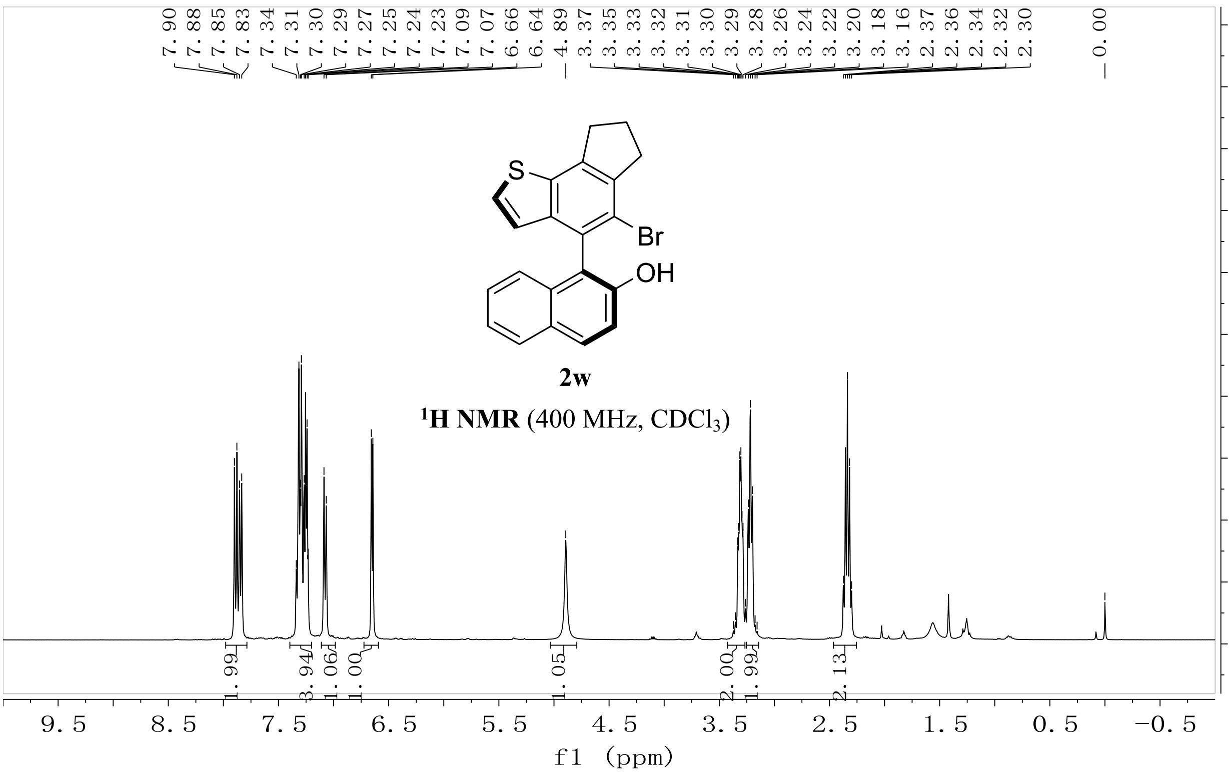
7.90 7.88 7.85 7.83 7.34 7.31 7.30 7.29 7.27 7.25 7.24 7.23 7.09 7.07 6.66 6.64 4.89 3.37 3.35 3.33 3.32 3.31 3.30 3.29 3.28 3.26 3.24 3.22 3.20 3.18 3.16 2.37 2.36 2.34 2.32 2.30 0.00
S
Br
OH
2w
1H NMR (400 MHz, CDCl3)
1.99 3.94 1.06 1.00 1.05 2.00 1.99 2.13
f1 (ppm)

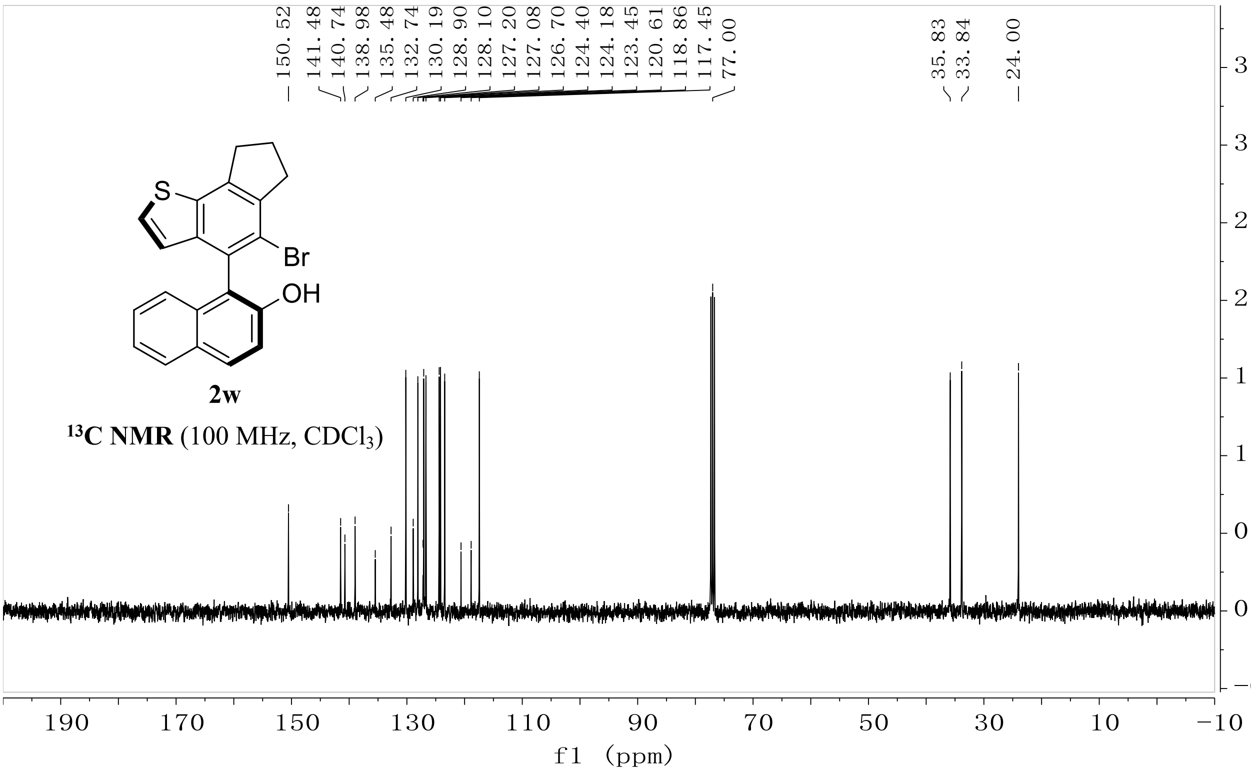
150.52 141.48 140.74 138.98 135.48 132.74 130.19 128.90 128.10 127.20 127.08 126.70 124.40 124.18 123.45 120.61 118.86 117.45 77.00 35.83 33.84 24.00
S
Br
OH
2w
13C NMR (100 MHz, CDCl3)
f1 (ppm)

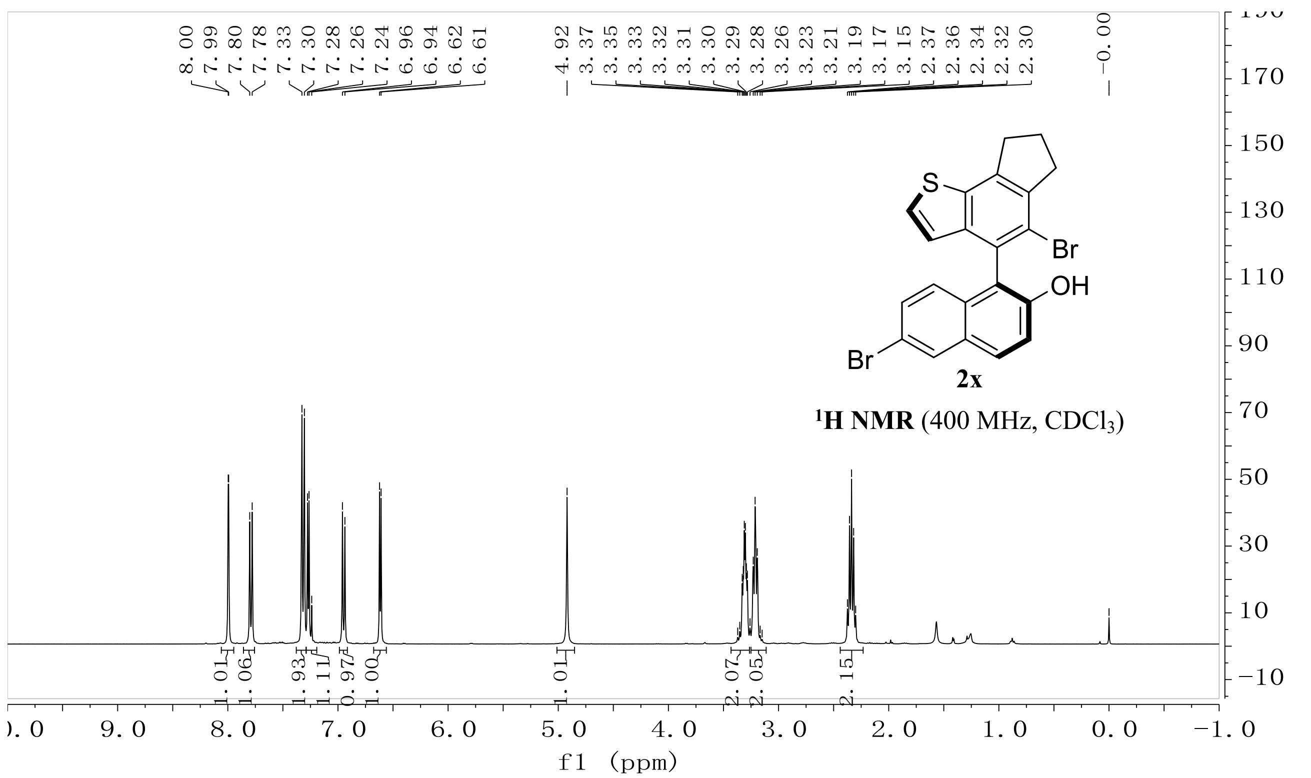

8.00 7.99 7.80 7.78 7.33 7.30 7.28 7.26 7.24 6.96 6.94 6.62 6.61
4.92 3.37 3.35 3.33 3.32 3.31 3.30 3.29 3.28 3.26 3.23 3.21 3.19 3.17 3.15 2.37 2.36 2.34 2.32 2.30
−0.00
S
Br
OH
Br
2x
1H NMR (400 MHz, CDCl3)
1.01 1.06 1.93 1.11 0.97 1.00 1.01 2.07 2.05 2.15
f1 (ppm)

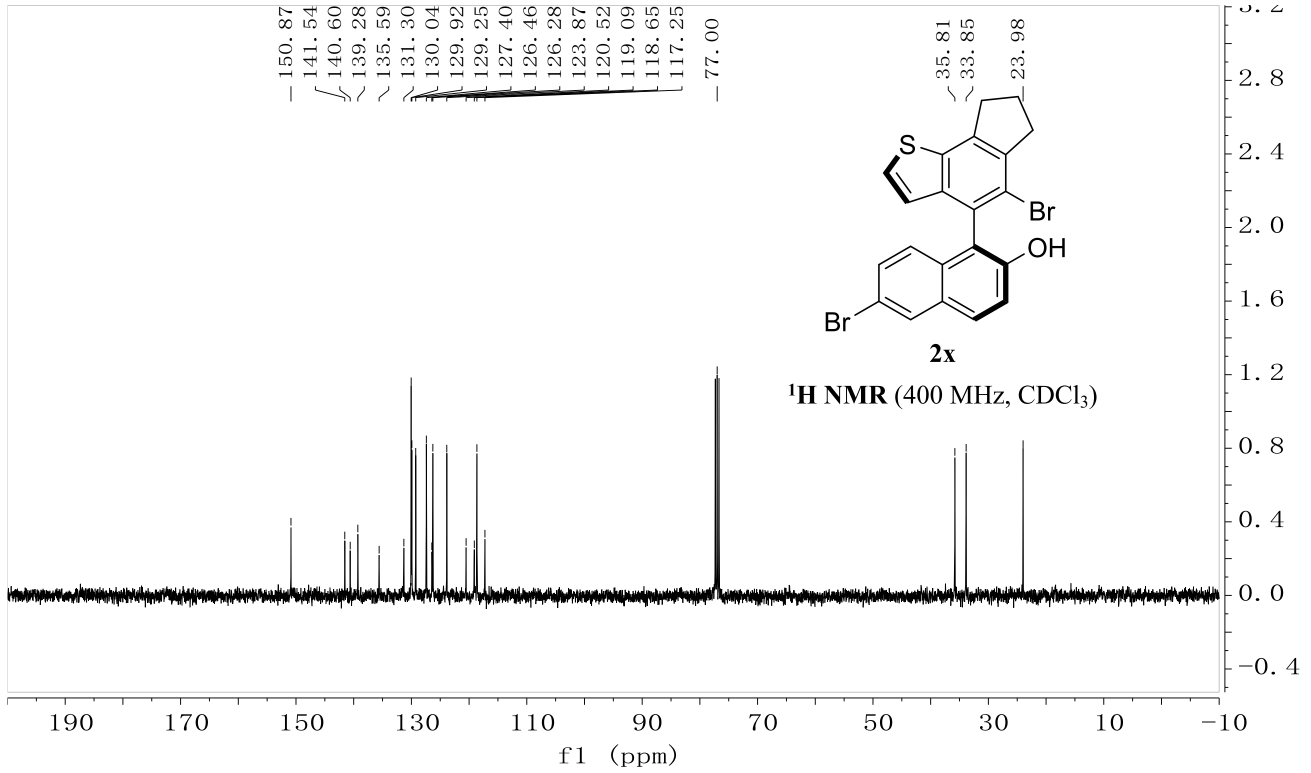

150.87 141.54 140.60 139.28 135.59 131.30 130.04 129.92 129.25 127.40 126.46 126.28 123.87 120.52 119.09 118.65 117.25
77.00
35.81 33.85 23.98
S
Br
OH
Br
2x
1H NMR (400 MHz, CDCl3)
f1 (ppm)

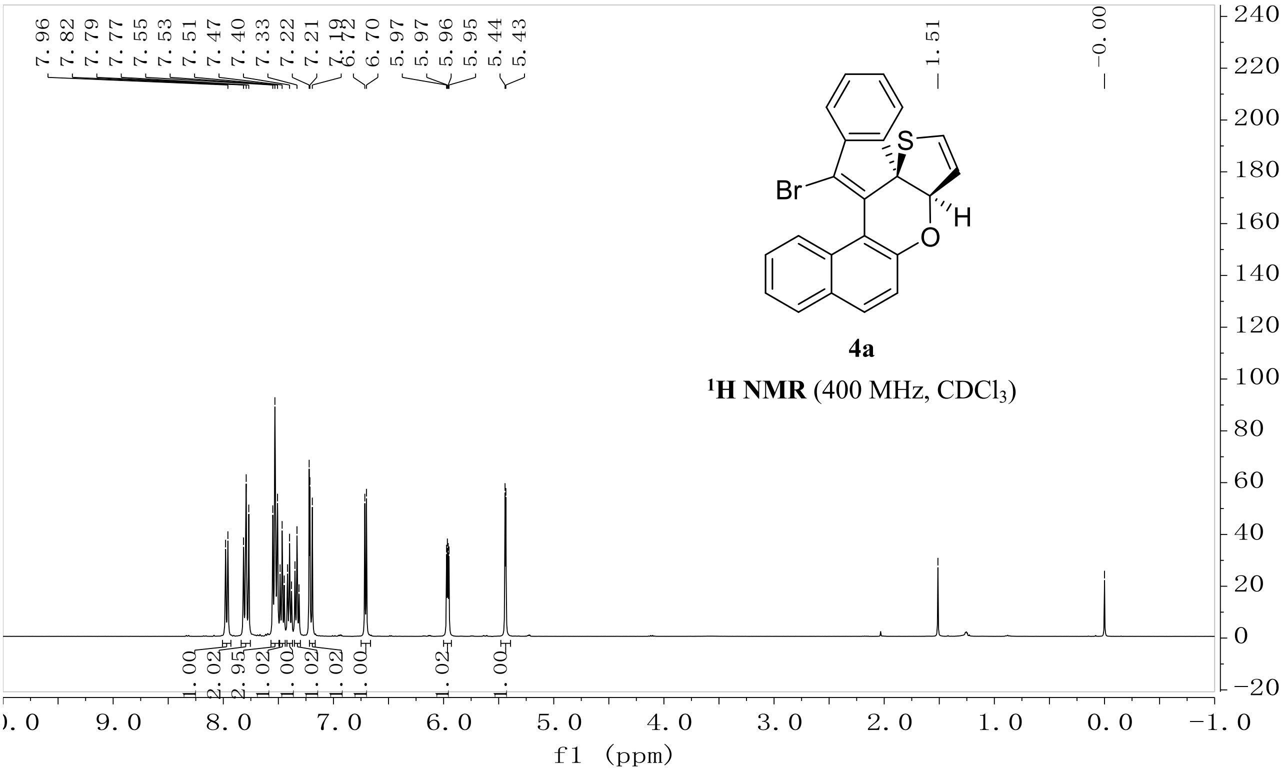

4a

^{1}H NMR (400 MHz, $CDCl_3$)

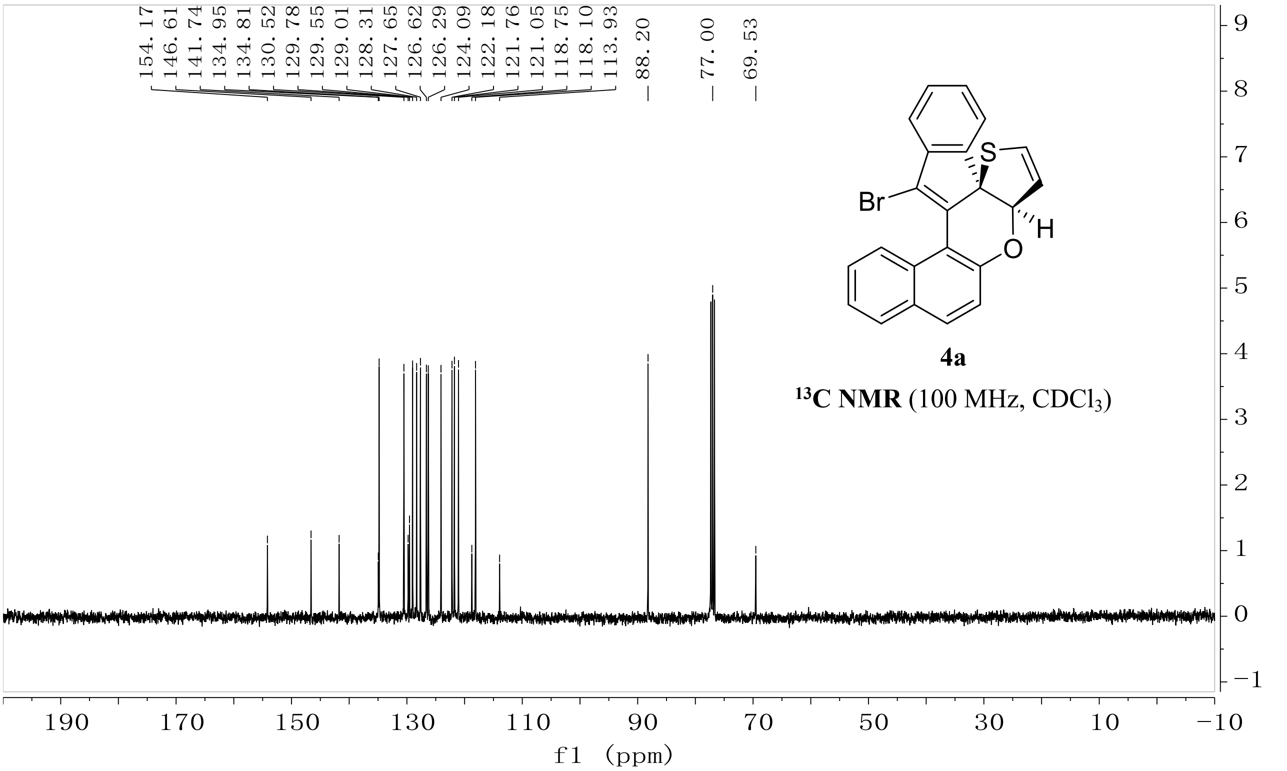

4a

^{13}C NMR (100 MHz, $CDCl_3$)

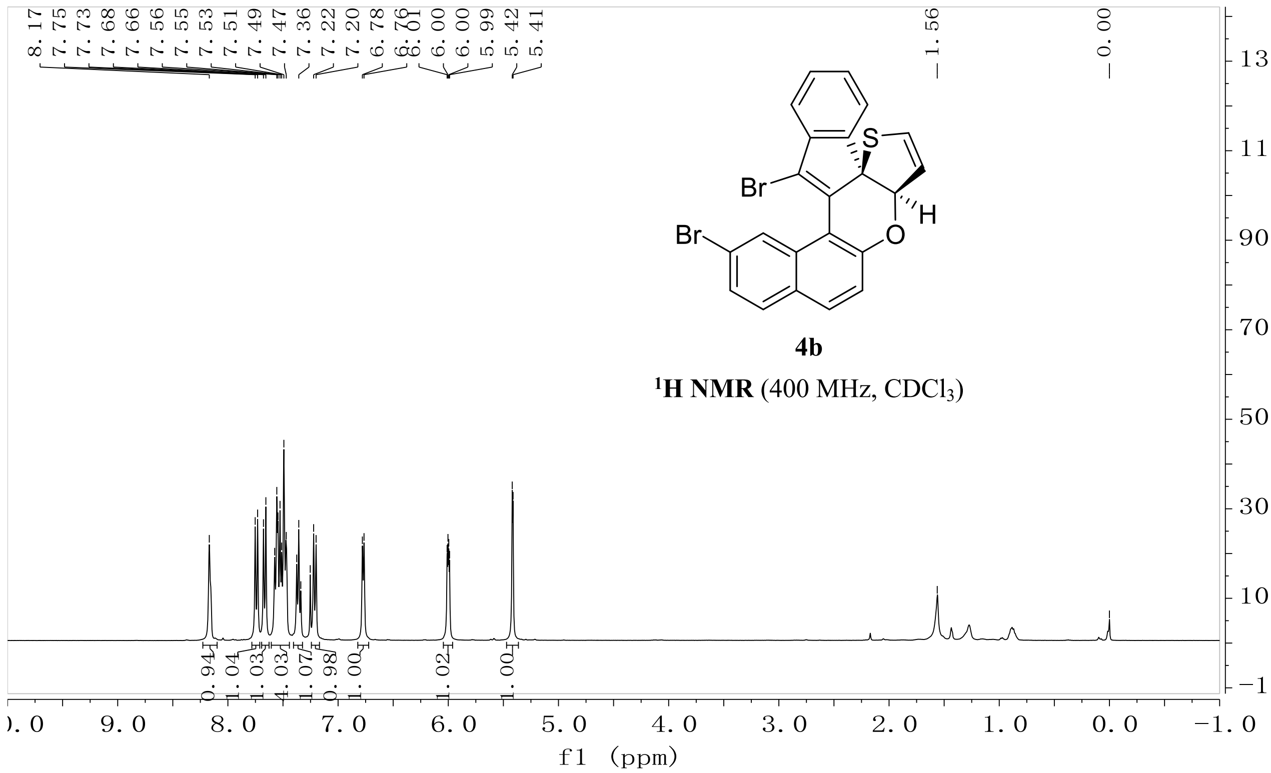

4b

^{1}H NMR (400 MHz, $CDCl_3$)

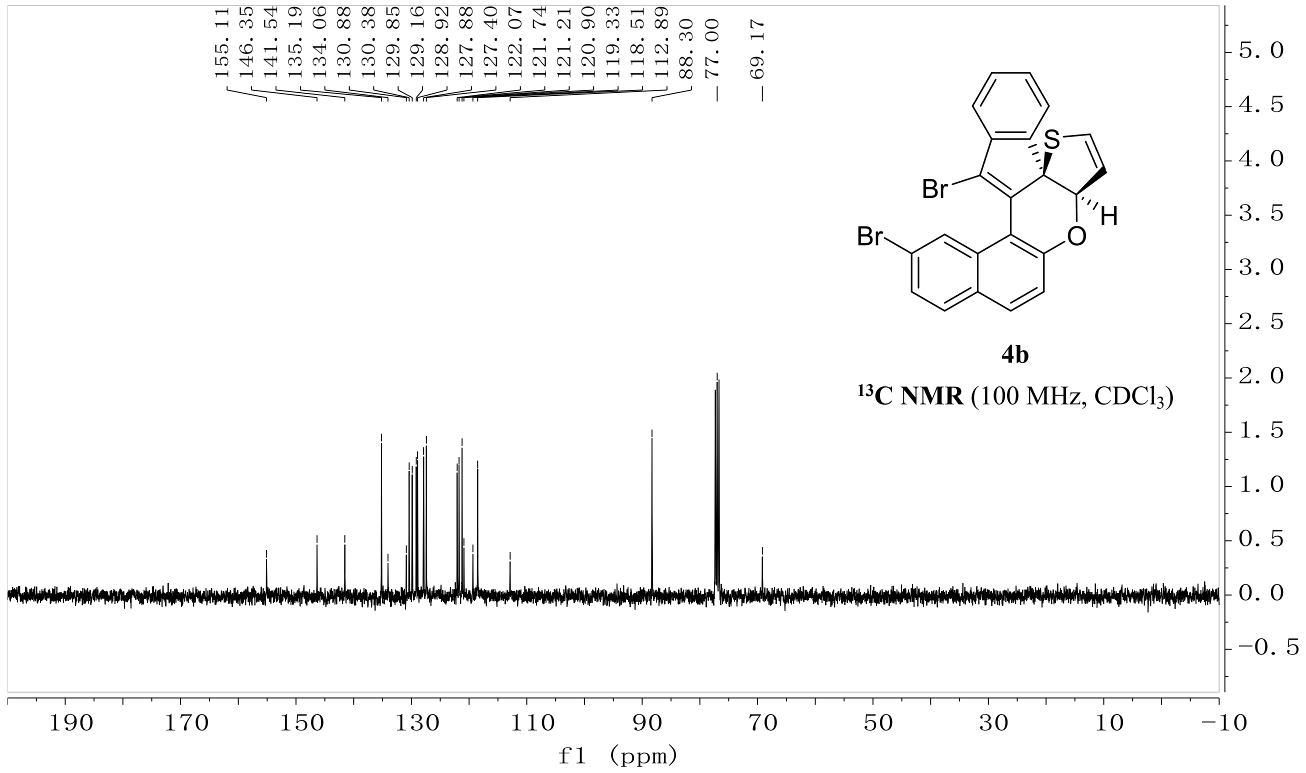

4b

^{13}C NMR (100 MHz, $CDCl_3$)

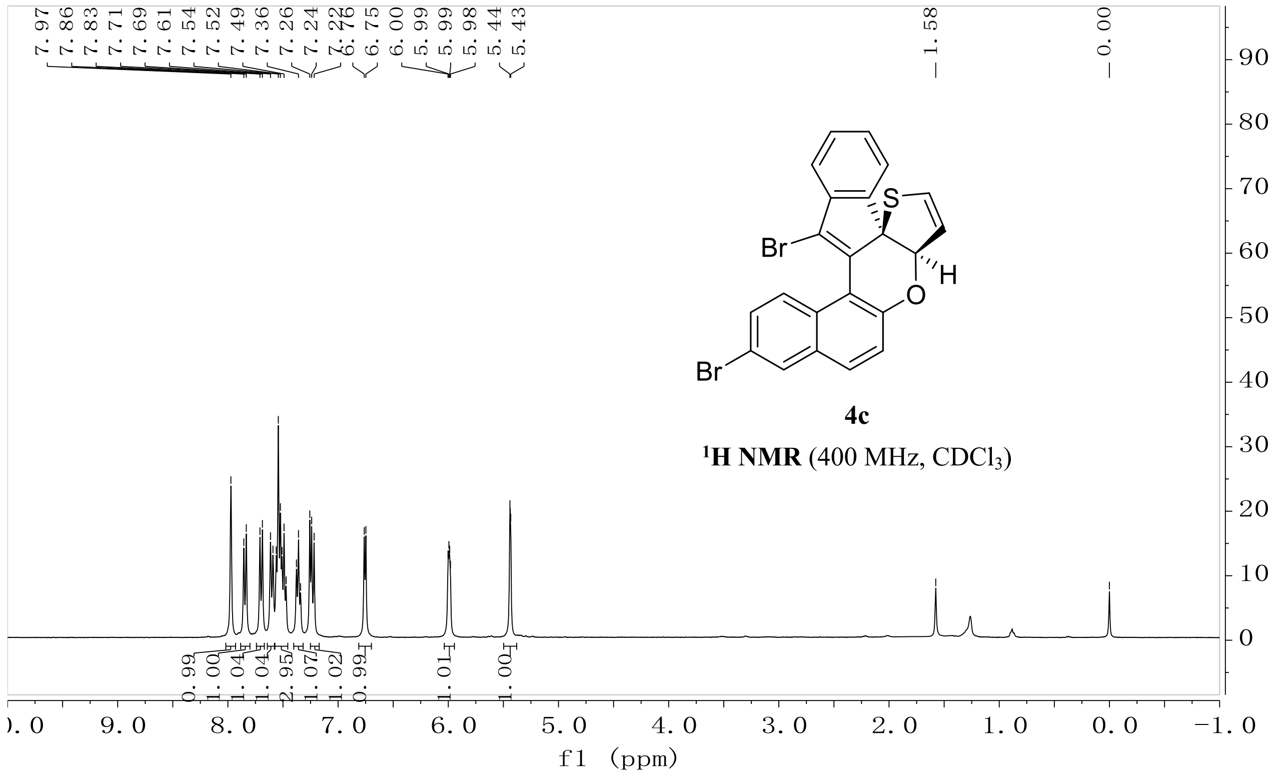

Br
Br
S
H
O
4c
¹H NMR (400 MHz, CDCl₃)
f1 (ppm)

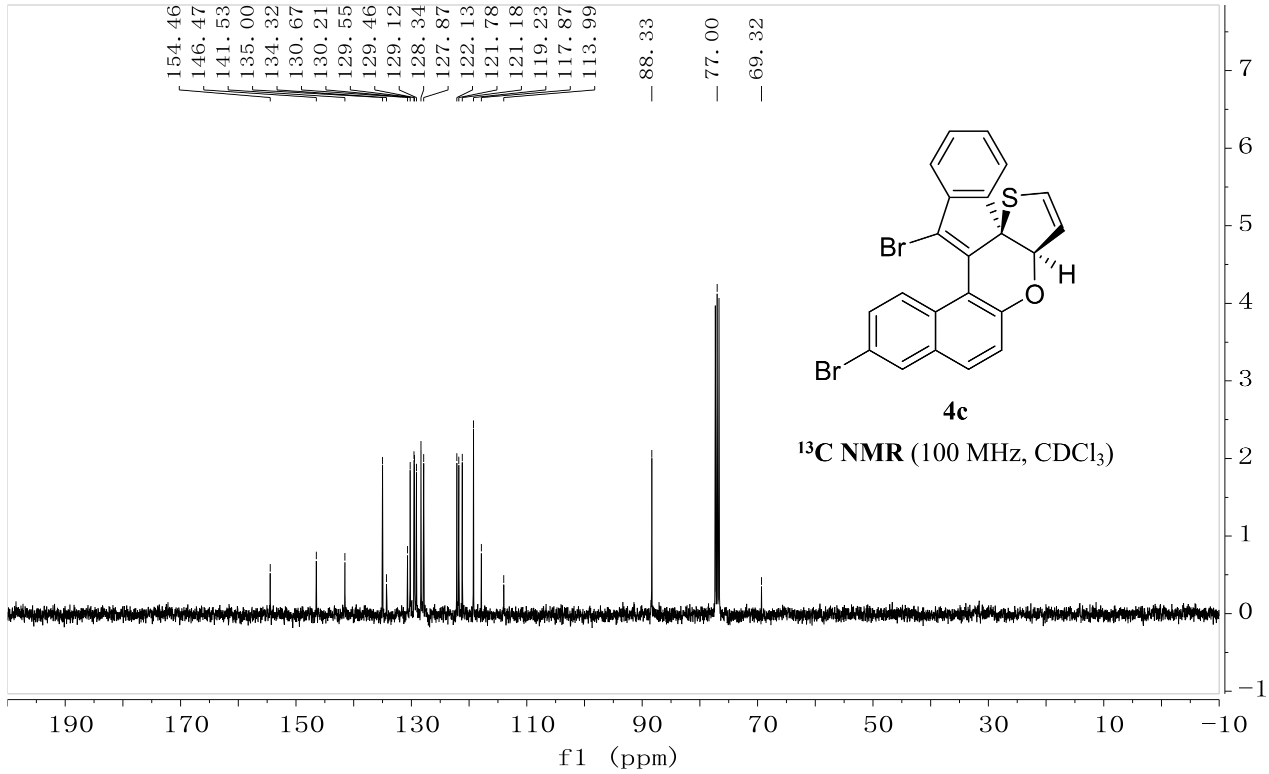

Br
Br
S
H
O
4c
¹³C NMR (100 MHz, CDCl₃)
f1 (ppm)

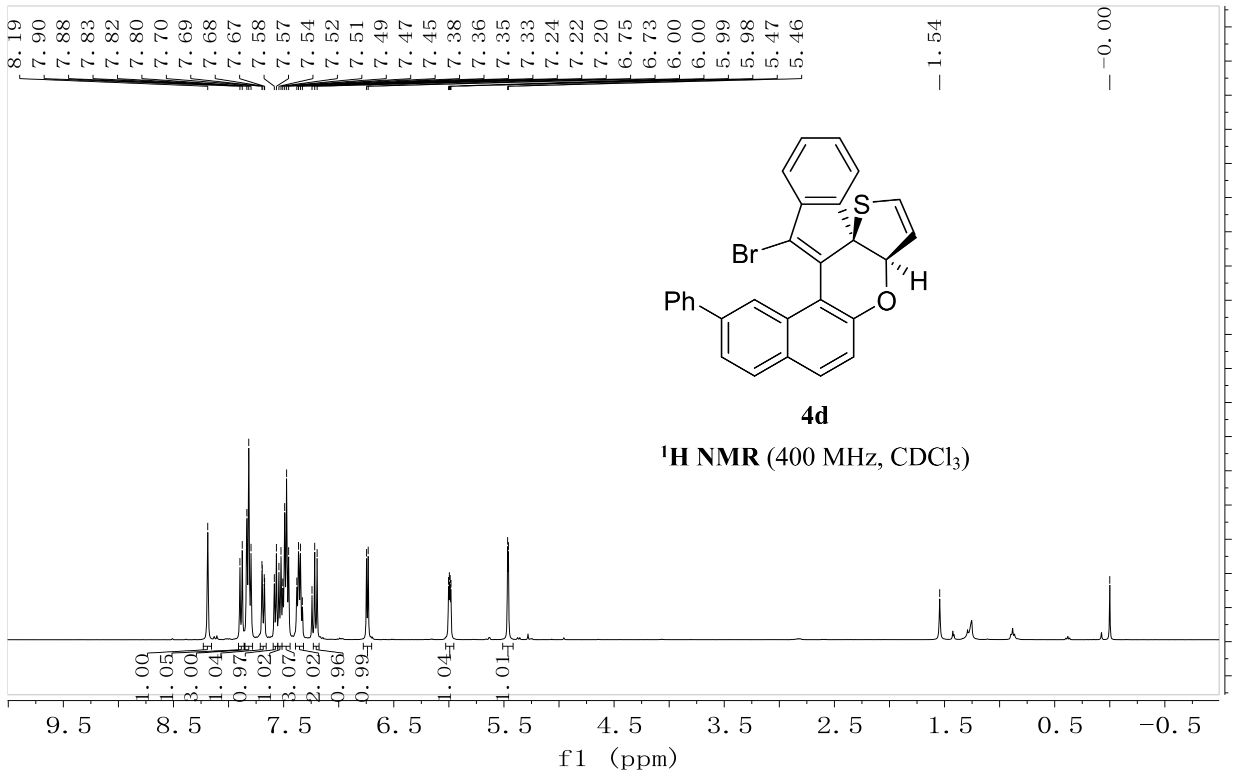
Br
Ph
S
O
H
4d
1H NMR (400 MHz, CDCl3)
f1 (ppm)

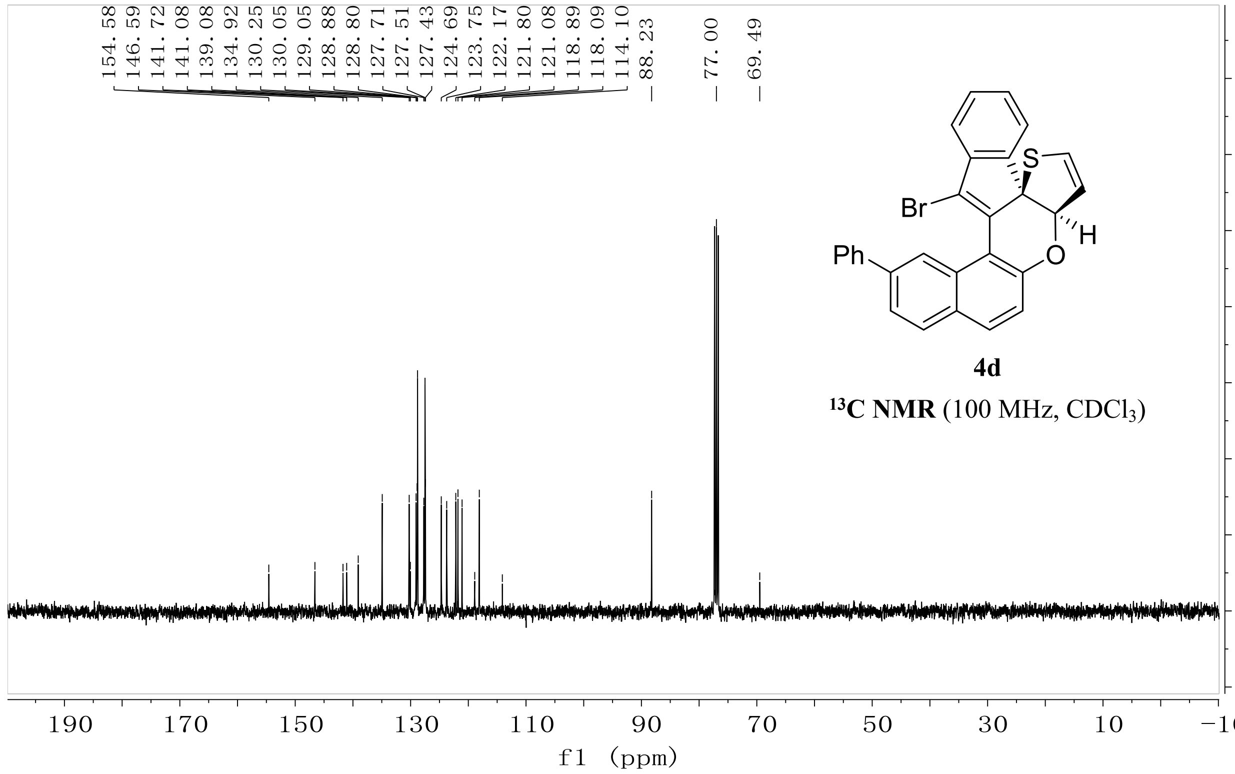
Br
Ph
S
O
H
4d
13C NMR (100 MHz, CDCl3)
f1 (ppm)

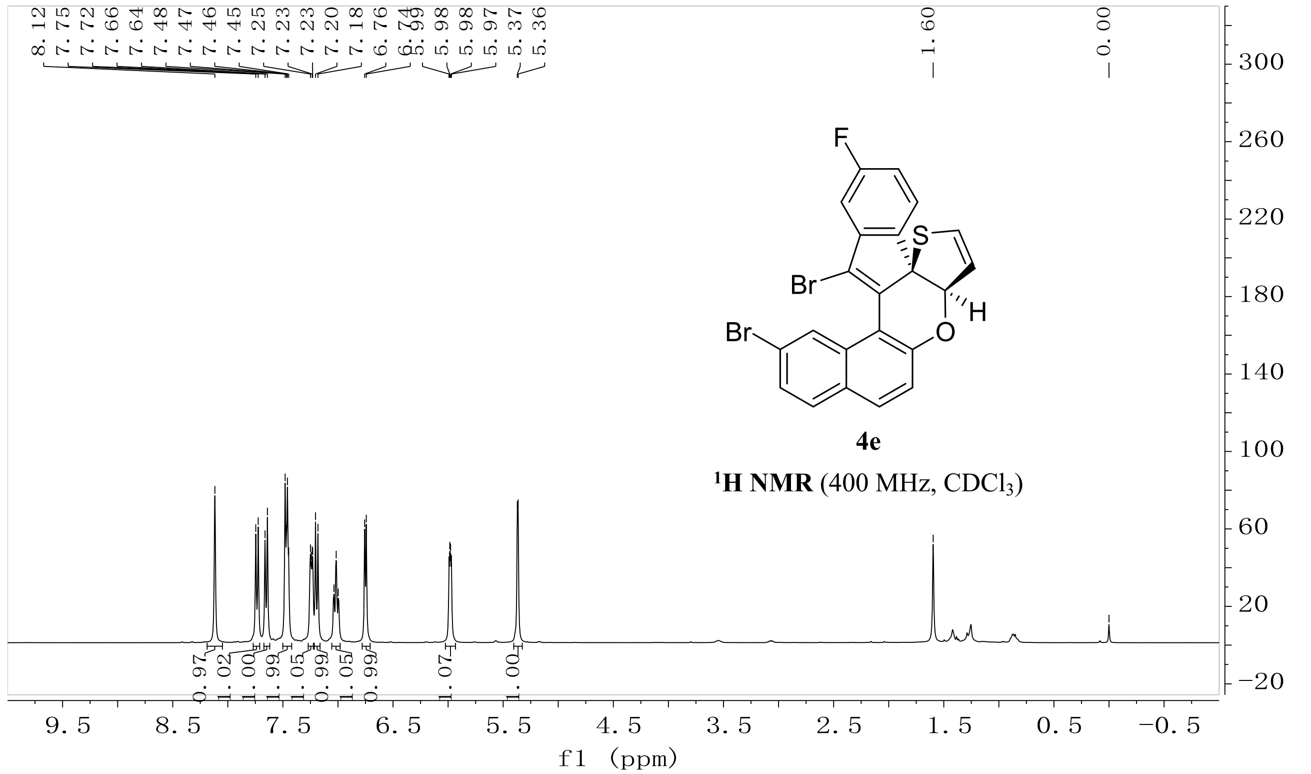
8.12 7.75 7.72 7.66 7.64 7.48 7.47 7.46 7.45 7.25 7.23 7.23 7.20 7.18 6.76 6.74 5.99 5.98 5.98 5.97 5.37 5.36
1.60
0.00
F
S
Br
H
Br
O
4e
1H NMR (400 MHz, CDCl3)
0.97 1.02 1.00 1.99 1.05 0.99 1.05 0.99 1.07 1.00
f1 (ppm)

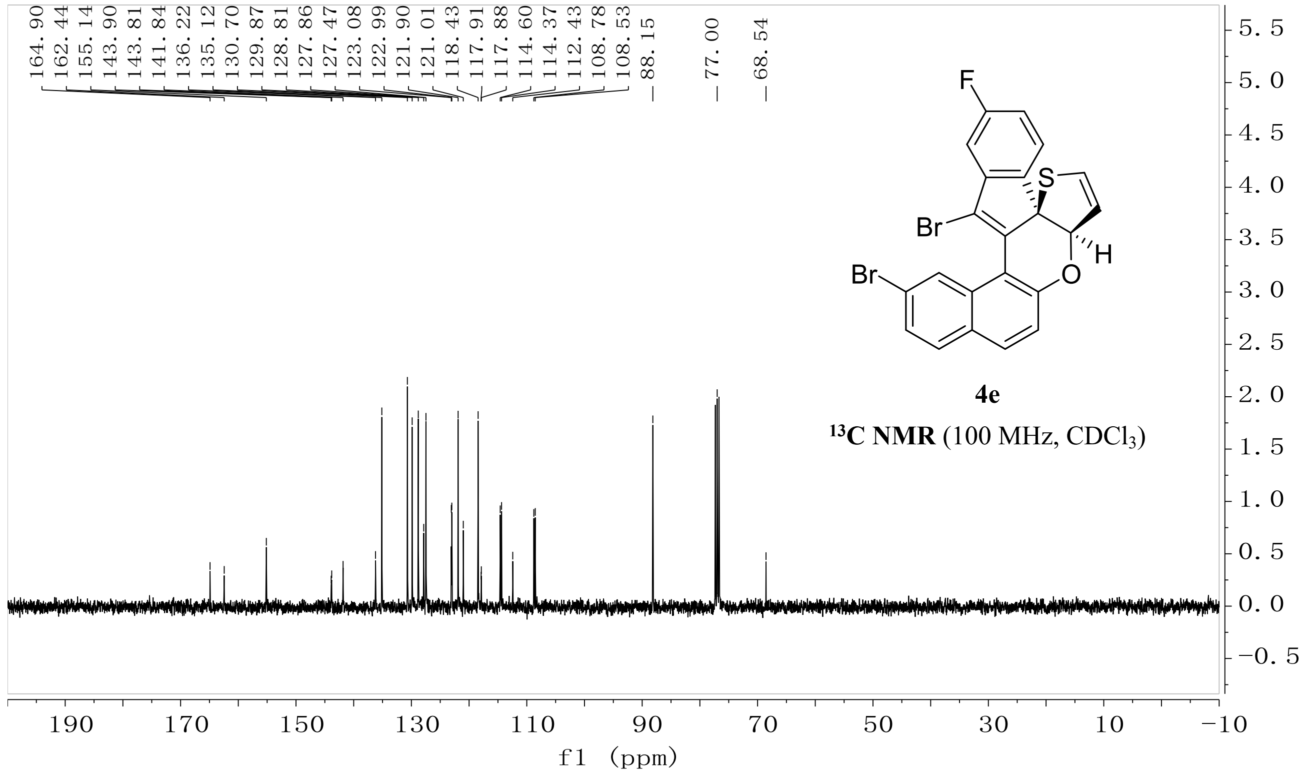
164.90 162.44 155.14 143.90 143.81 141.84 136.22 135.12 130.70 129.87 128.81 127.86 127.47 123.08 122.99 121.90 121.01 118.43 117.91 117.88 114.60 114.37 112.43 108.78 108.53
88.15
77.00
68.54
F
S
Br
H
Br
O
4e
13C NMR (100 MHz, CDCl3)
f1 (ppm)

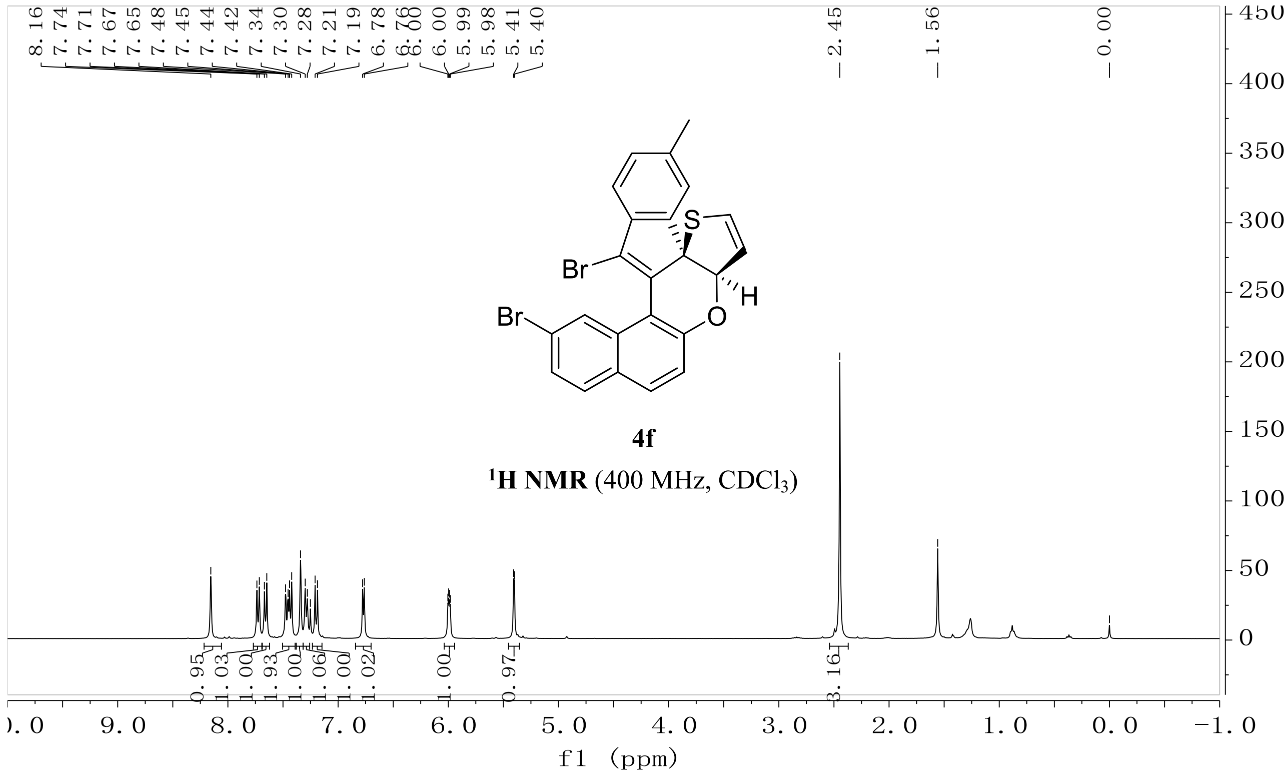

8.16 7.74 7.71 7.67 7.65 7.48 7.45 7.44 7.42 7.34 7.30 7.28 7.21 7.19 6.78 6.76 6.00 6.00 5.99 5.98 5.41 5.40
2.45
1.56
0.00
Br
Br
S
H
O
4f
¹H NMR (400 MHz, CDCl₃)
0.95 1.03 1.00 1.93 1.00 1.06 1.00 1.02 1.00 0.97 3.16
f1 (ppm)

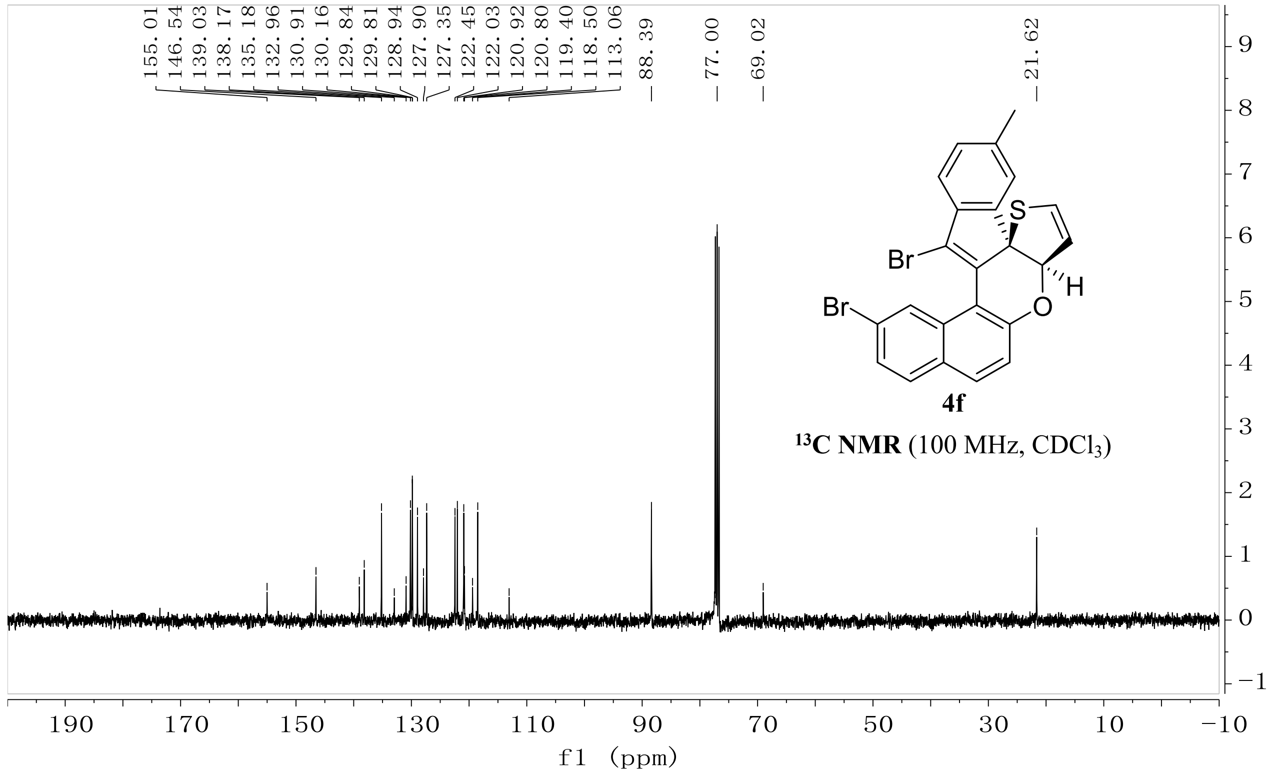

155.01 146.54 139.03 138.17 135.18 132.96 130.91 130.16 129.84 129.81 128.94 127.90 127.35 122.45 122.03 120.92 120.80 119.40 118.50 113.06
88.39
77.00
69.02
21.62
Br
Br
S
H
O
4f
¹³C NMR (100 MHz, CDCl₃)
f1 (ppm)

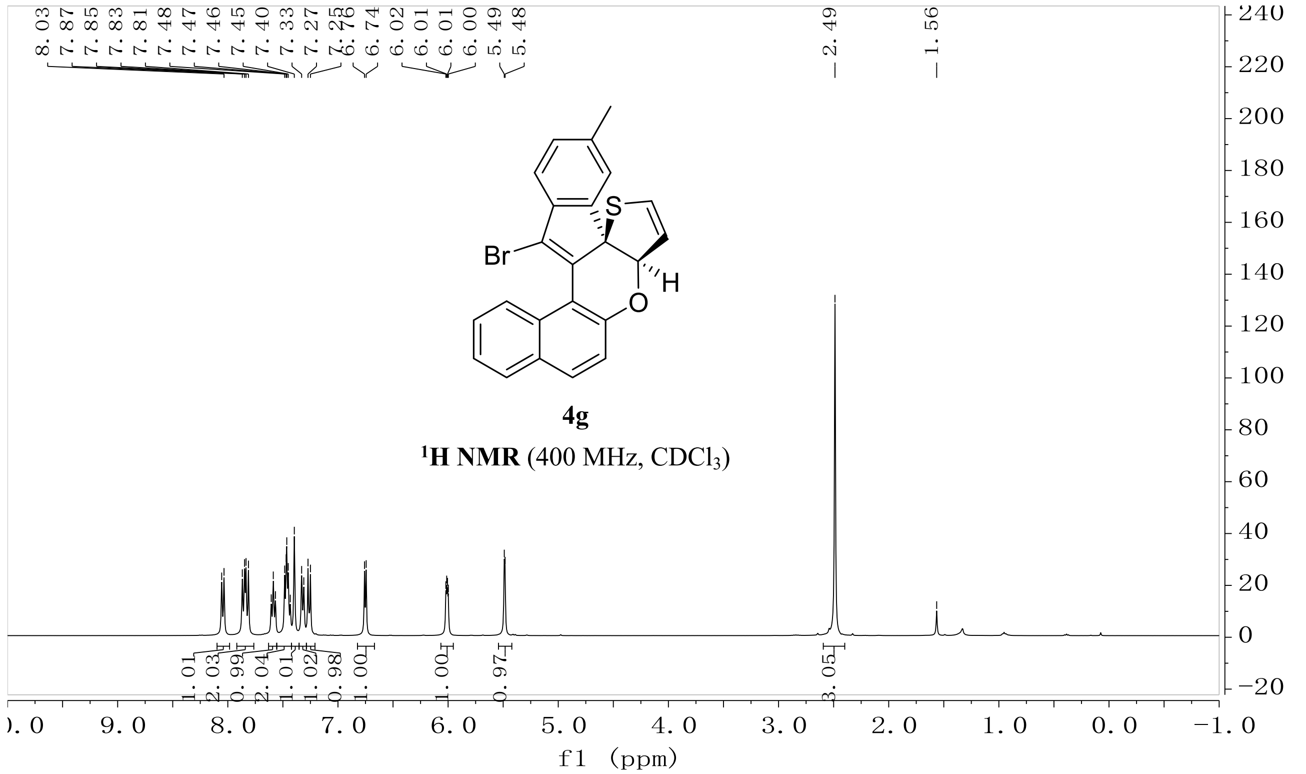
8.03
7.87
7.85
7.83
7.81
7.48
7.47
7.46
7.45
7.40
7.33
7.27
7.25
6.76
6.74
6.02
6.01
6.01
6.00
5.49
5.48
2.49
1.56
Br
S
H
O
4g
1H NMR (400 MHz, CDCl3)
1.01
2.03
0.99
2.04
1.01
1.02
0.98
1.00
1.00
0.97
3.05
f1 (ppm)

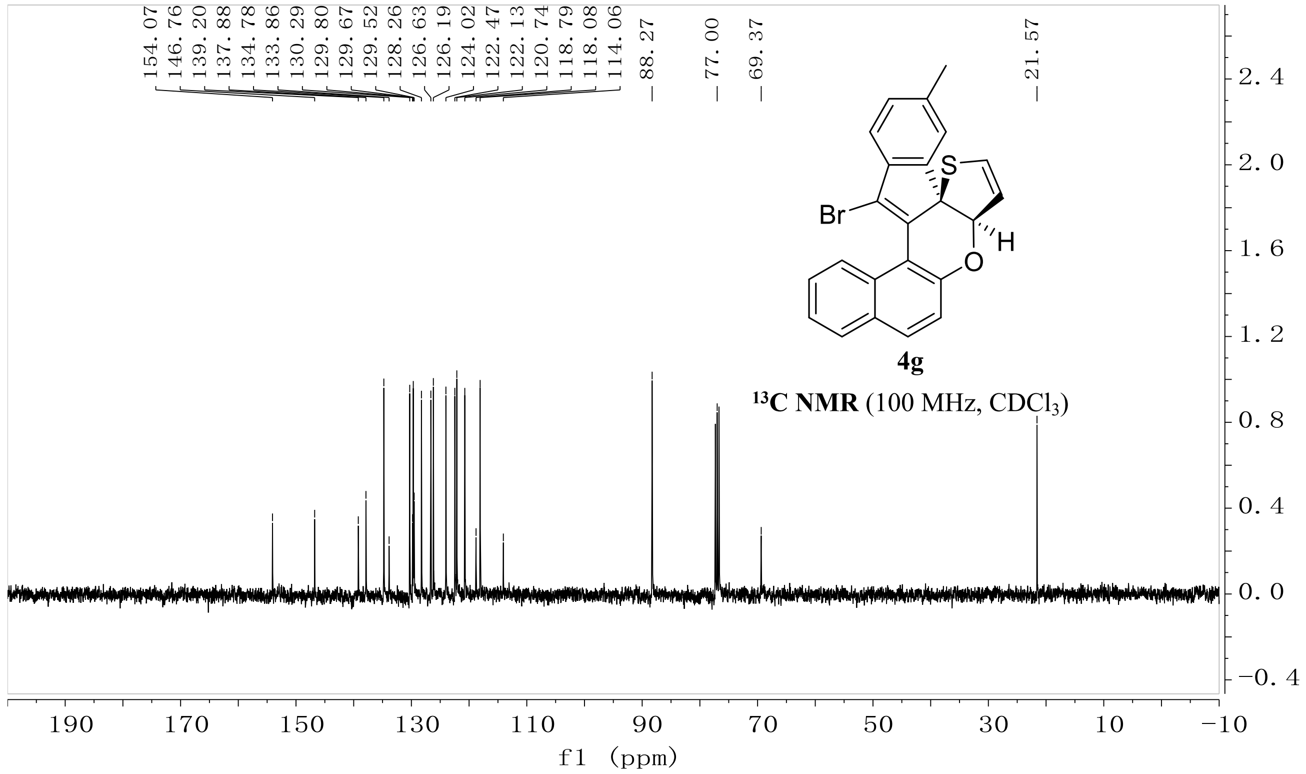
154.07
146.76
139.20
137.88
134.78
133.86
130.29
129.80
129.67
129.52
128.26
126.63
126.19
124.02
122.47
122.13
120.74
118.79
118.08
114.06
88.27
77.00
69.37
21.57
Br
S
H
O
4g
13C NMR (100 MHz, CDCl3)
f1 (ppm)

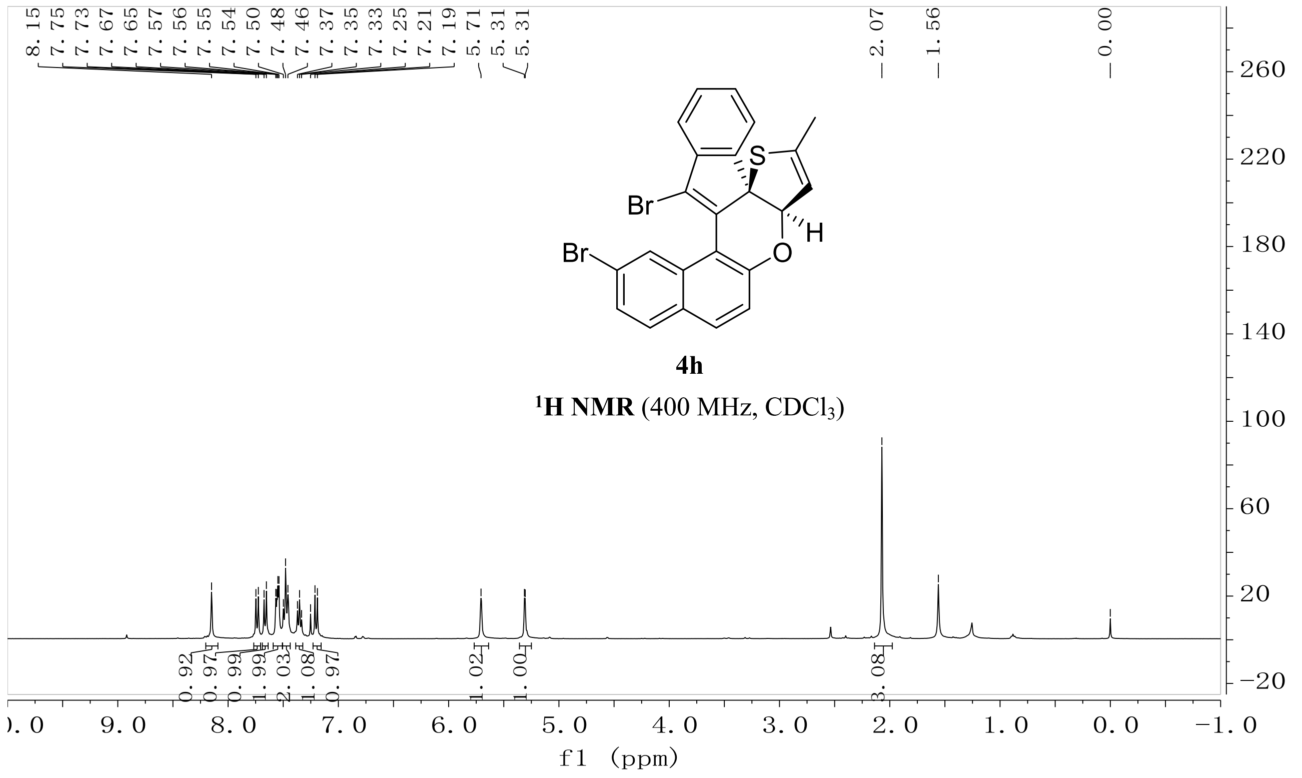

8.15 7.75 7.73 7.67 7.65 7.57 7.56 7.55 7.54 7.50 7.48 7.46 7.37 7.35 7.33 7.25 7.21 7.19 5.71 5.31 5.31 2.07 1.56 0.00
Br
Br
S
O
H
4h
¹H NMR (400 MHz, CDCl₃)
0.92 0.97 0.99 1.99 2.03 1.08 0.97 1.02 1.00 3.08
f1 (ppm)

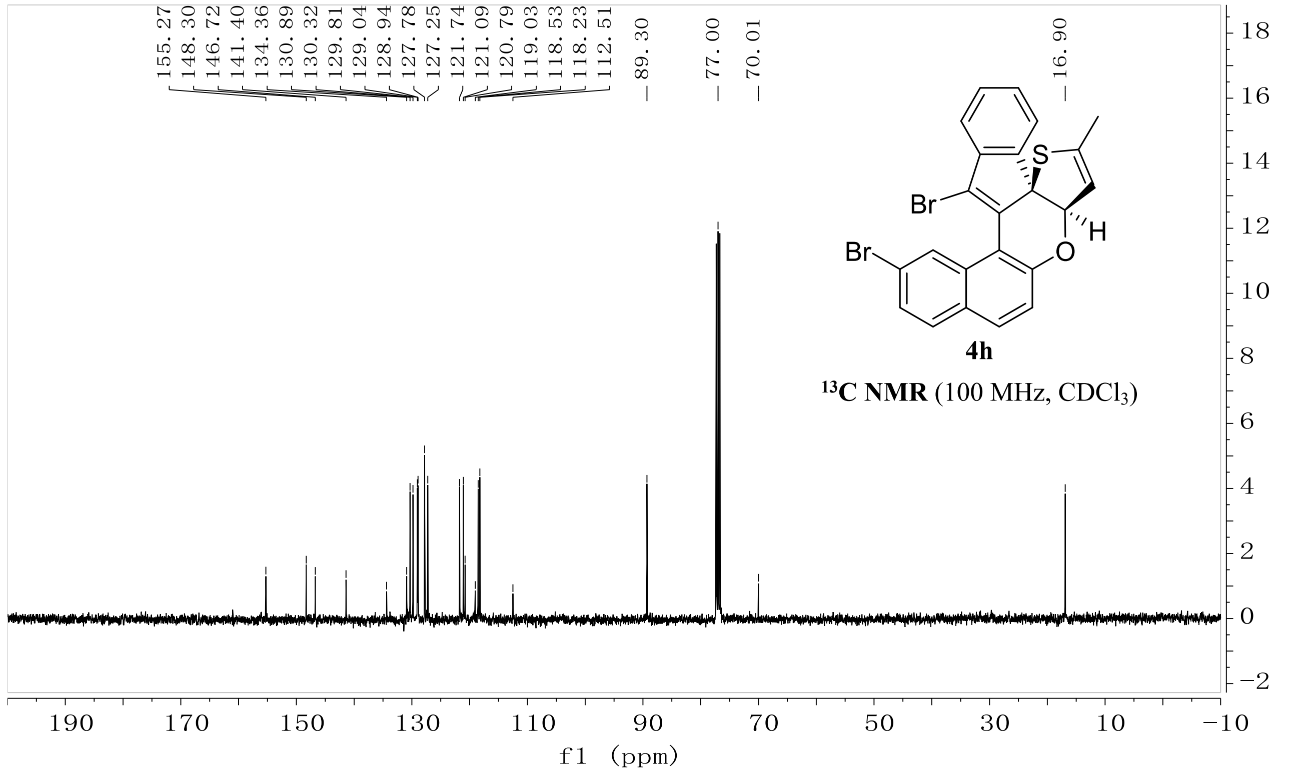

155.27 148.30 146.72 141.40 134.36 130.89 130.32 129.81 129.04 128.94 127.78 127.25 121.74 121.09 120.79 119.03 118.53 118.23 112.51 89.30 77.00 70.01 16.90
Br
Br
S
O
H
4h
¹³C NMR (100 MHz, CDCl₃)
f1 (ppm)

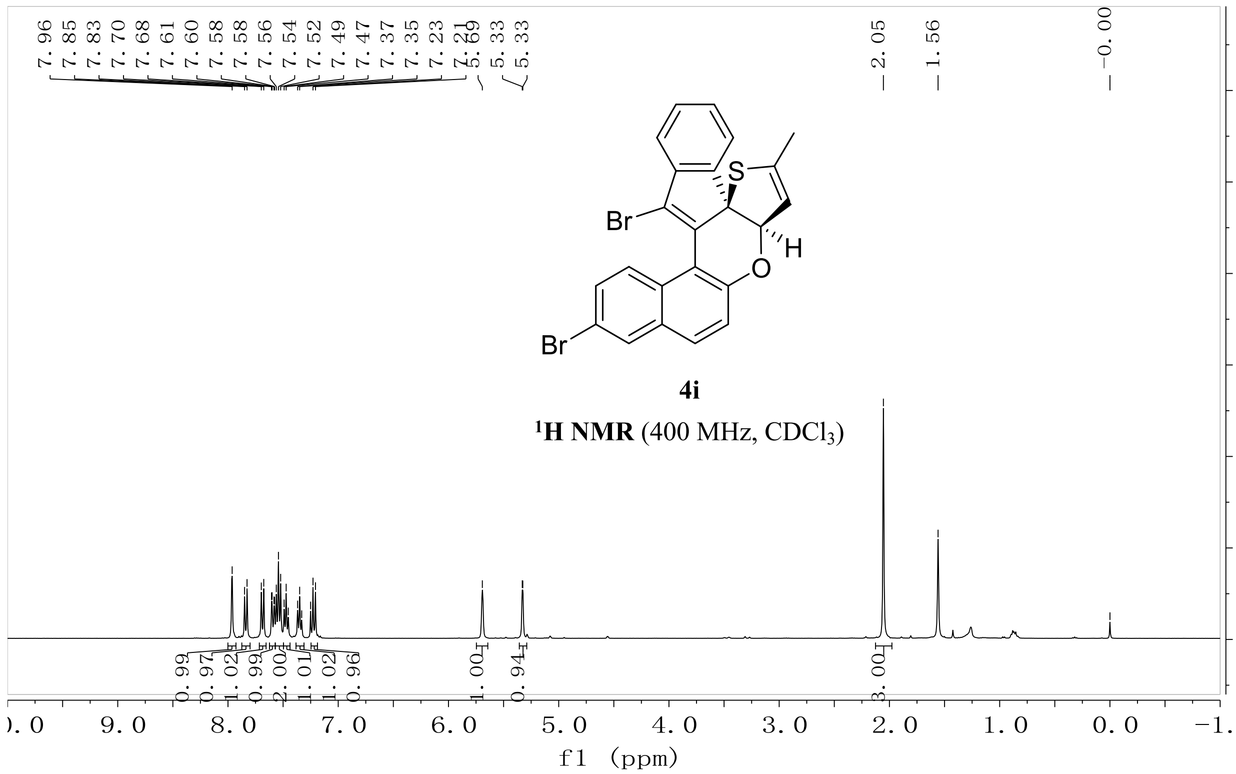

4i

^{1}H NMR (400 MHz, $CDCl_3$)

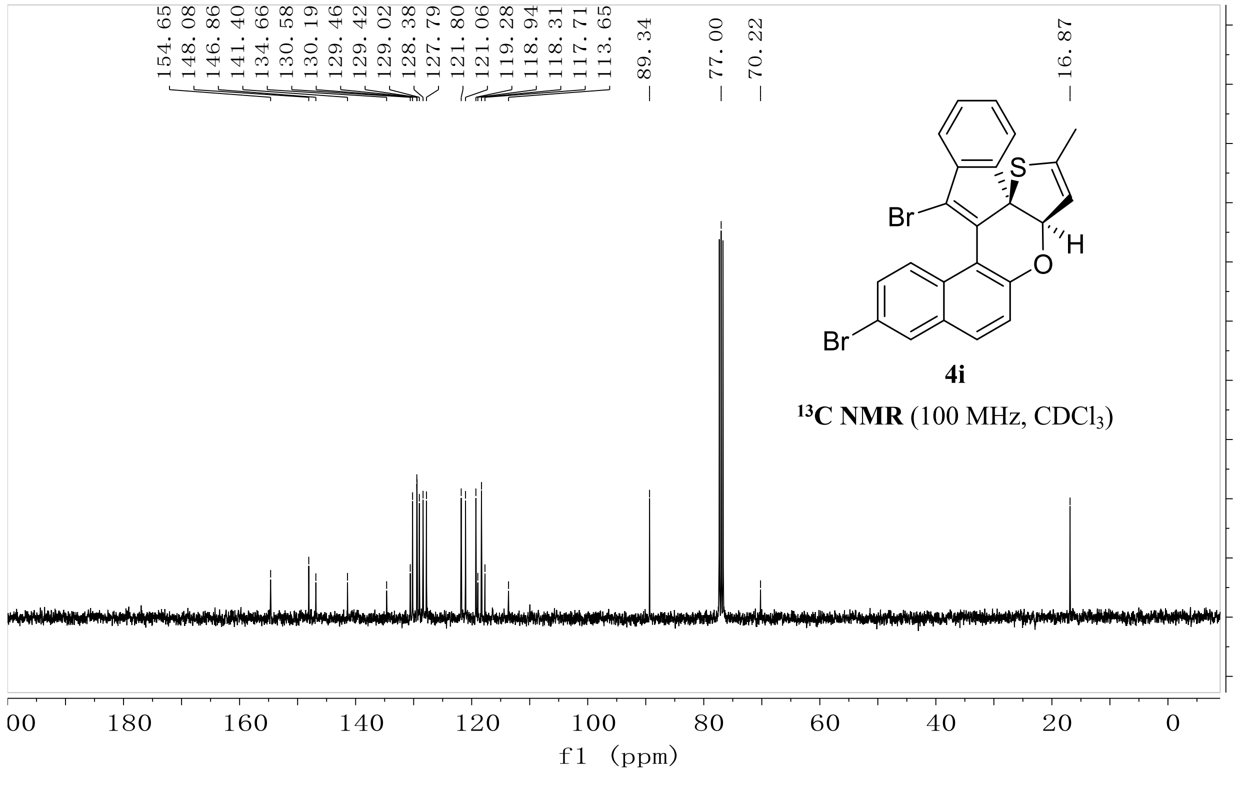

4i

^{13}C NMR (100 MHz, $CDCl_3$)

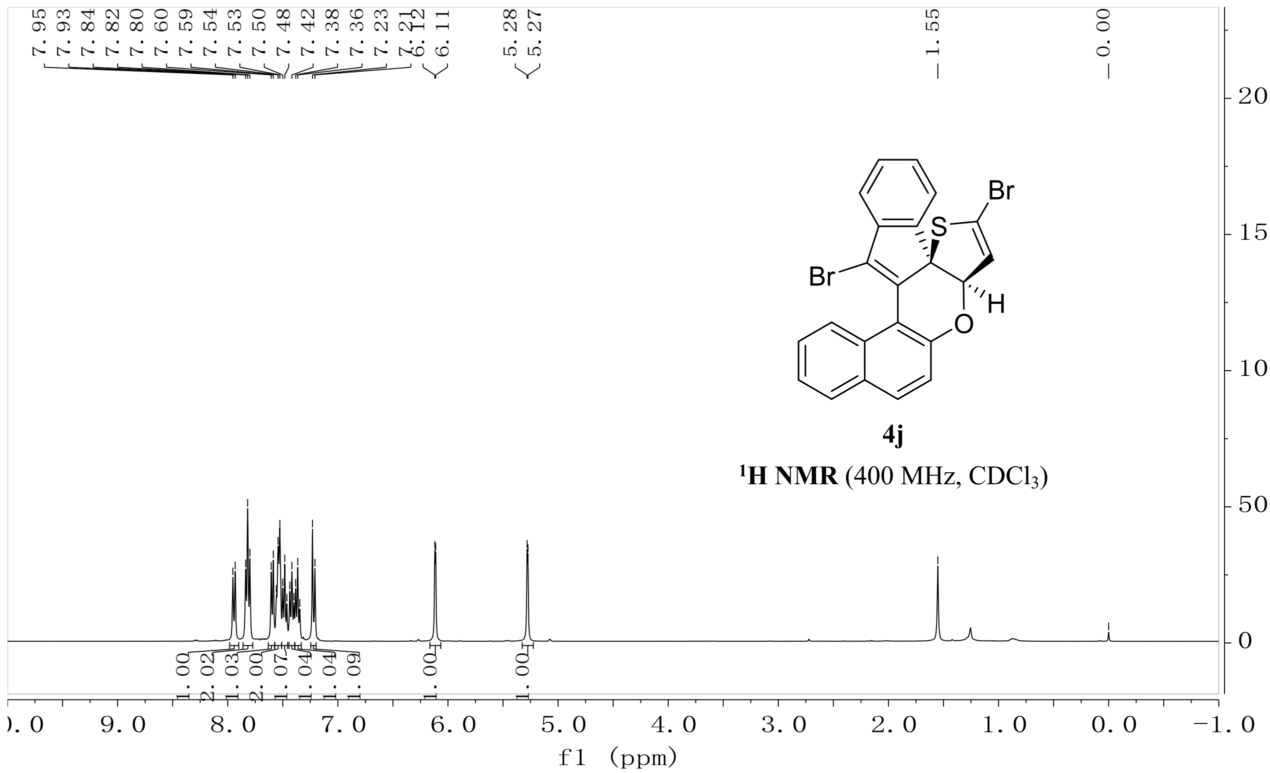
7.95
7.93
7.84
7.82
7.80
7.60
7.59
7.54
7.53
7.50
7.48
7.42
7.38
7.36
7.23
7.21
6.12
6.11
5.28
5.27
1.55
0.00
Br
S
Br
H
O
4j
1H NMR (400 MHz, CDCl3)
f1 (ppm)

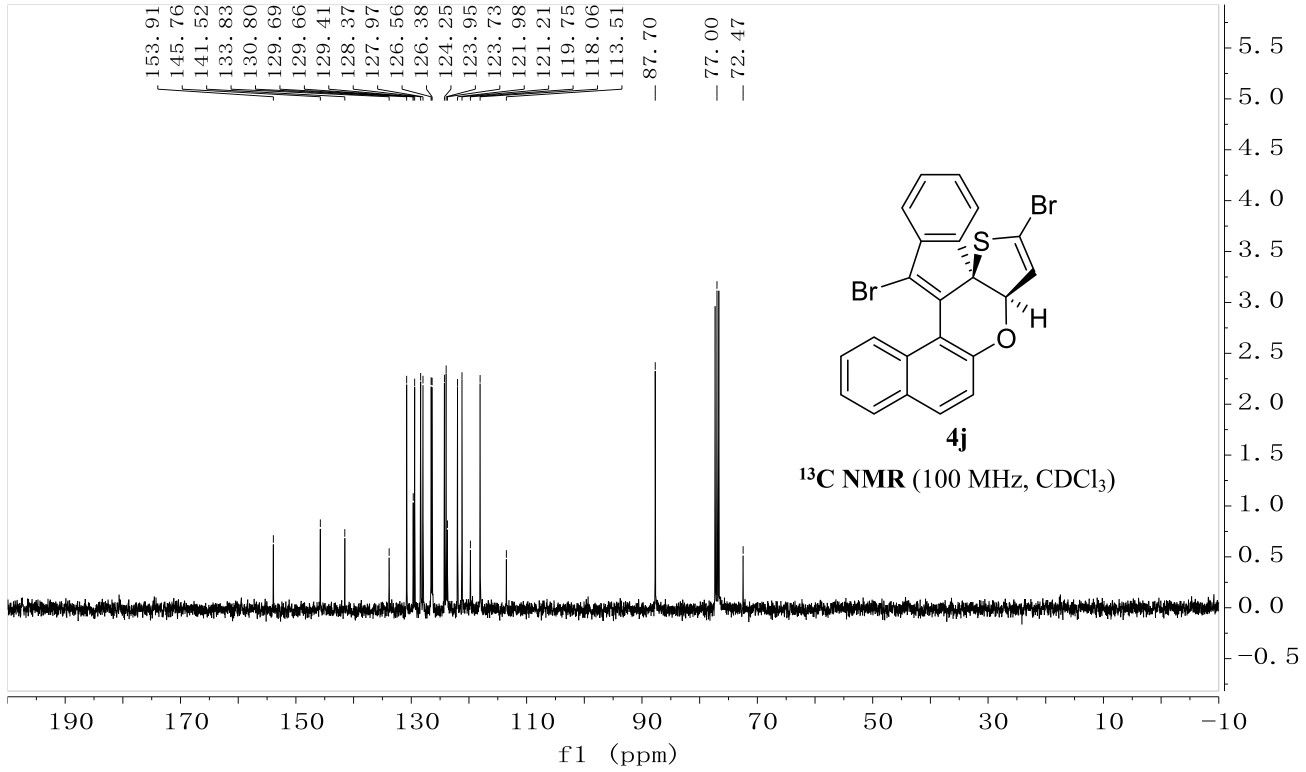
153.91
145.76
141.52
133.83
130.80
129.69
129.66
129.41
128.37
127.97
126.56
126.38
124.25
123.95
123.73
121.98
121.21
119.75
118.06
113.51
87.70
77.00
72.47
Br
S
Br
H
O
4j
13C NMR (100 MHz, CDCl3)
f1 (ppm)

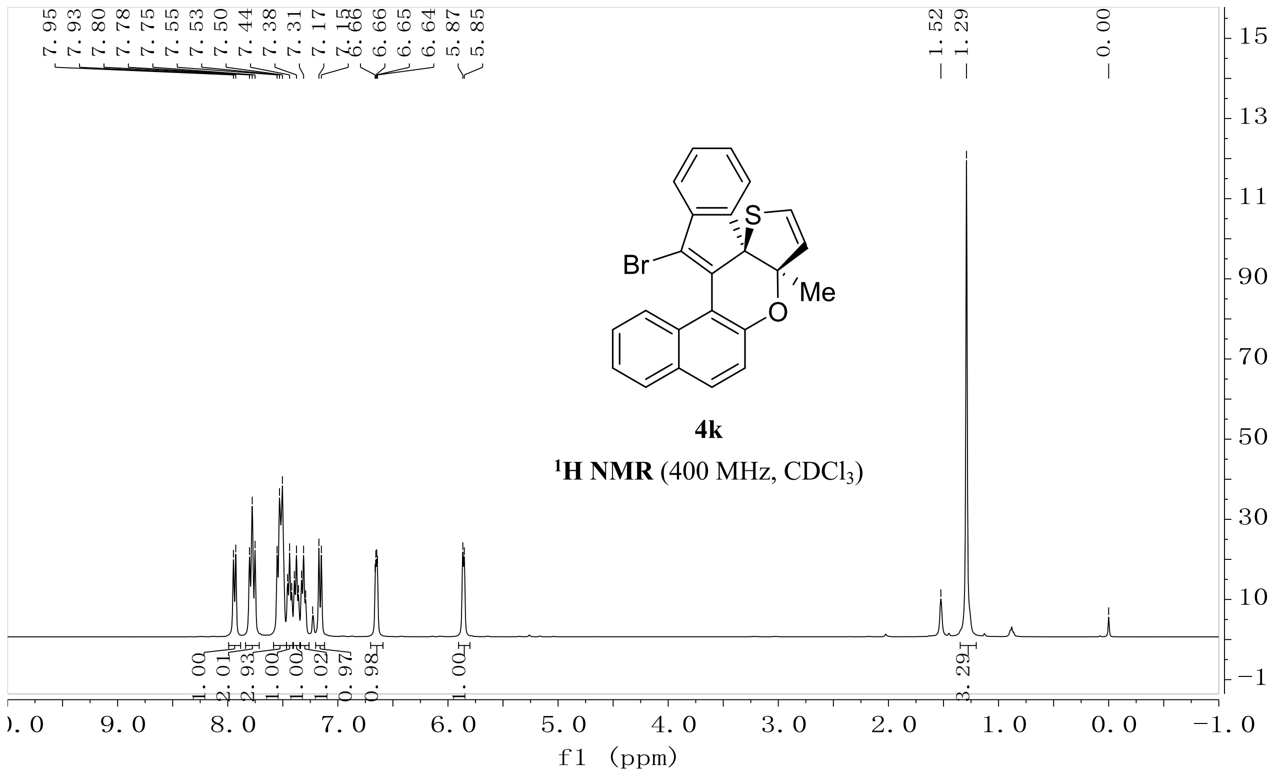
7.95 7.93 7.80 7.78 7.75 7.55 7.53 7.50 7.44 7.38 7.31 7.17 7.15 6.66 6.65 6.64 5.87 5.85
1.52 1.29
0.00
Br
S
Me
O
4k
1H NMR (400 MHz, CDCl3)
1.00 2.01 2.93 1.00 1.00 1.02 0.97 0.98
1.00
3.29
f1 (ppm)

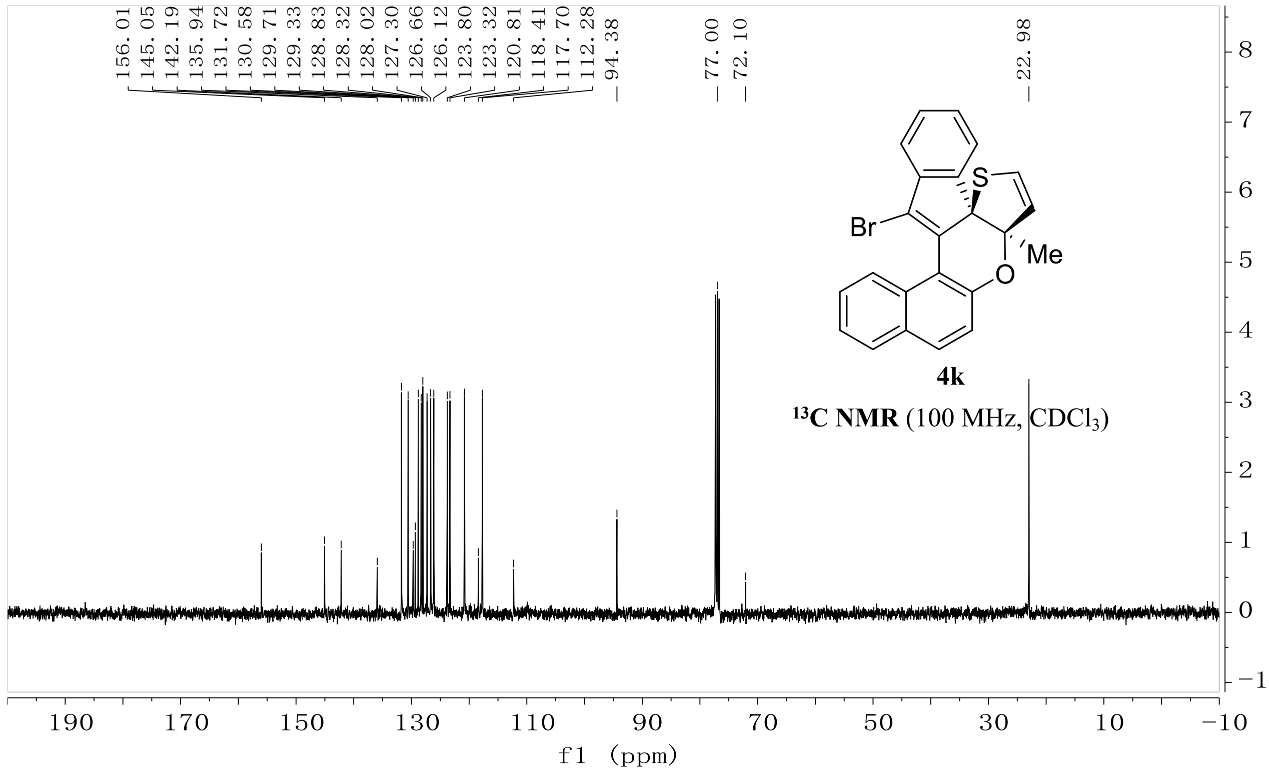
156.01 145.05 142.19 135.94 131.72 130.58 129.71 129.33 128.83 128.32 128.02 127.30 126.66 126.12 123.80 123.32 120.81 118.41 117.70 112.28
94.38
77.00 72.10
22.98
Br
S
Me
O
4k
13C NMR (100 MHz, CDCl3)
f1 (ppm)

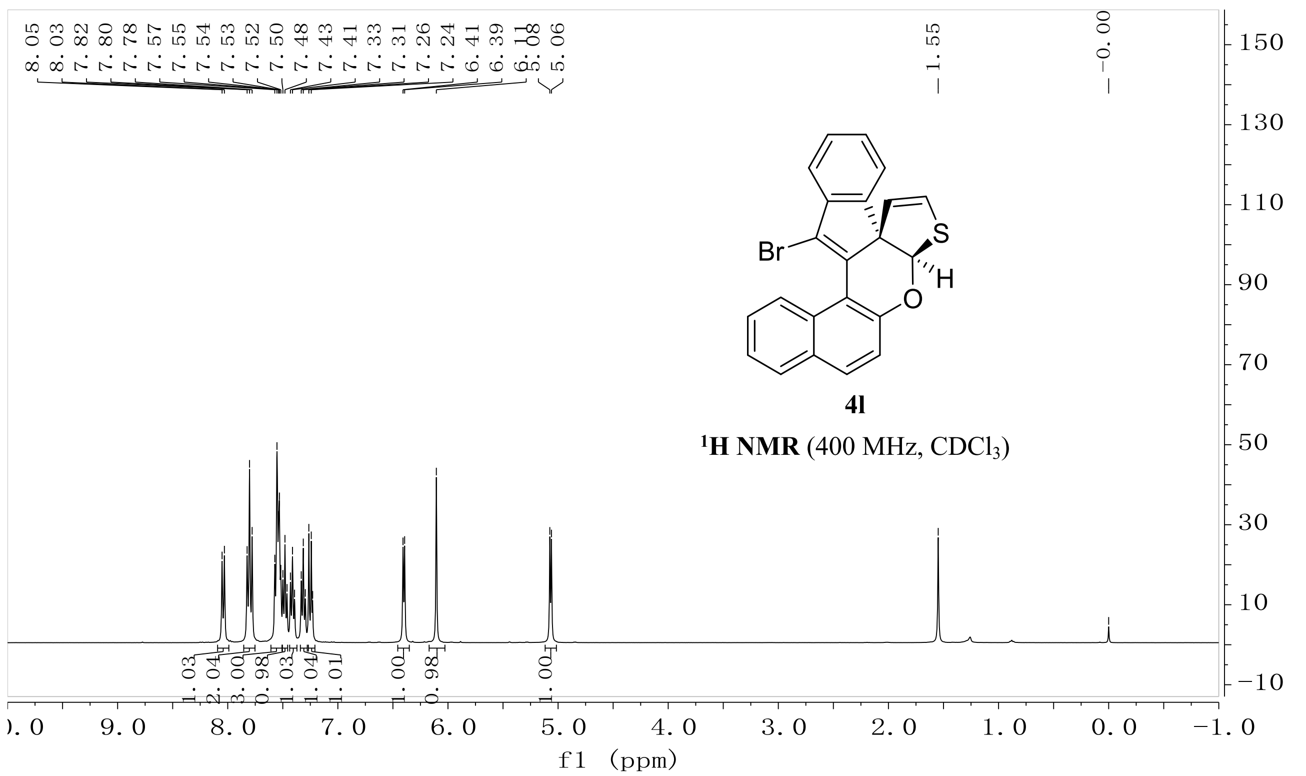

4l
¹H NMR (400 MHz, CDCl₃)
f1 (ppm)

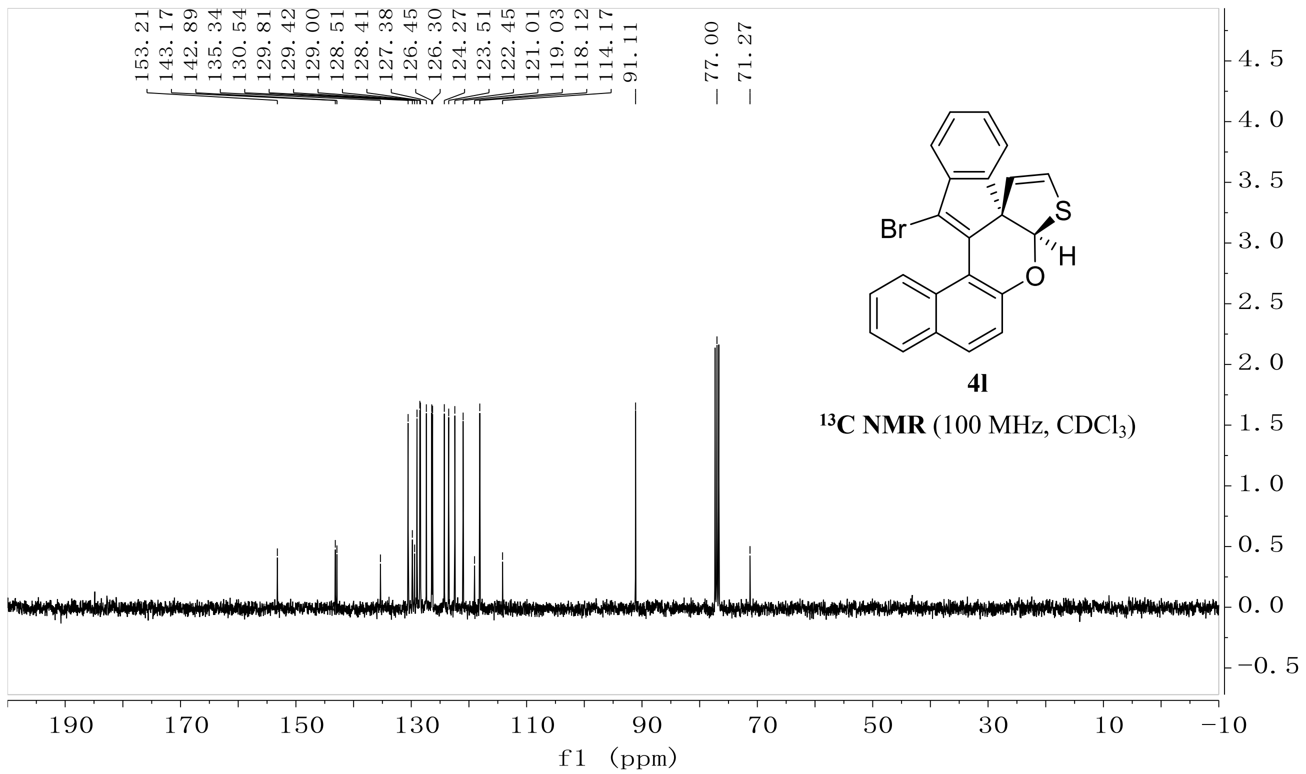

4l
¹³C NMR (100 MHz, CDCl₃)
f1 (ppm)

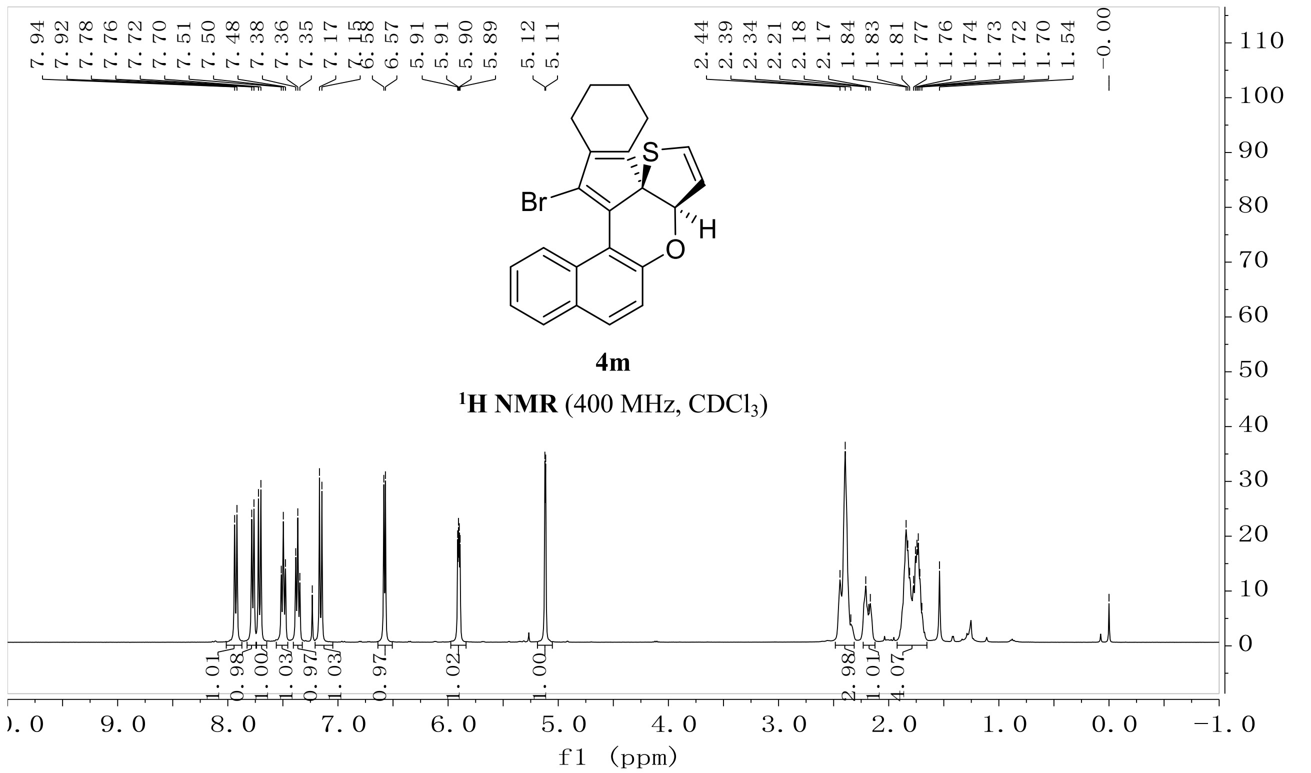

4m

^{1}H NMR (400 MHz, $CDCl_3$)

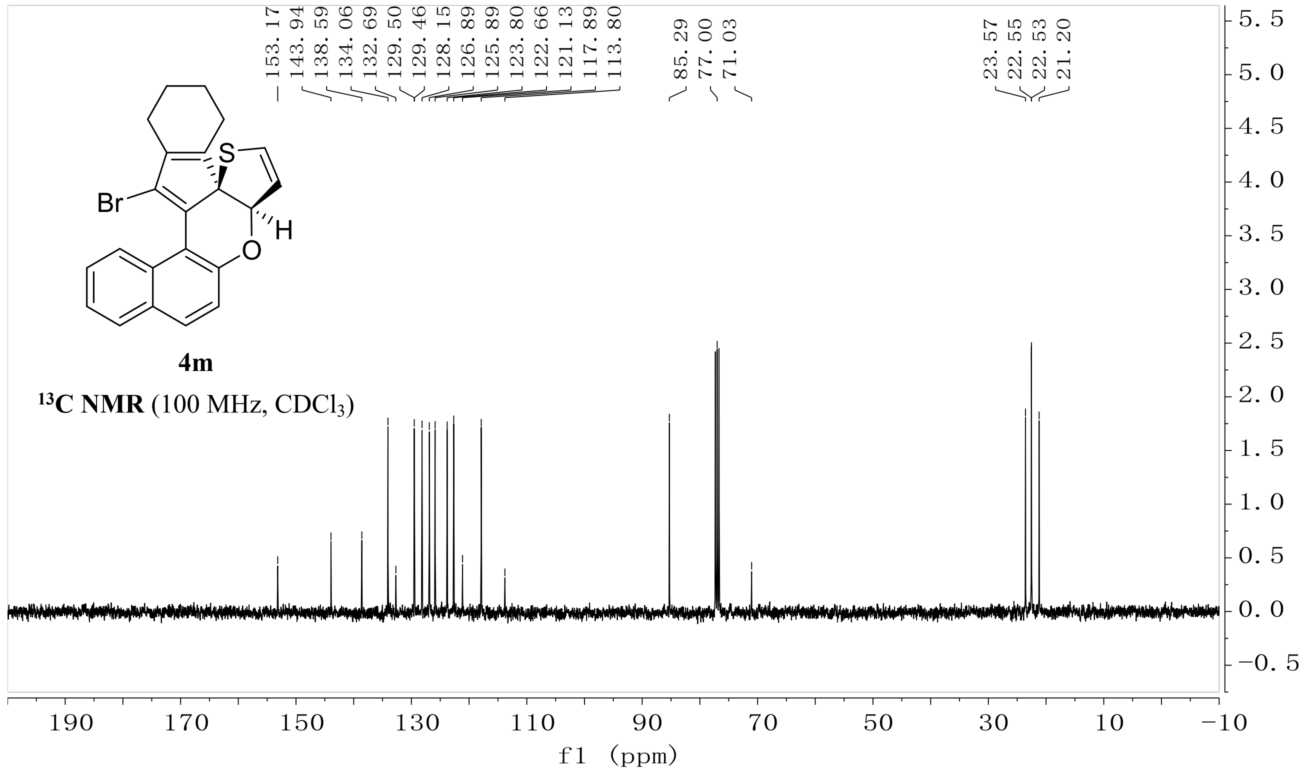

4m

^{13}C NMR (100 MHz, $CDCl_3$)

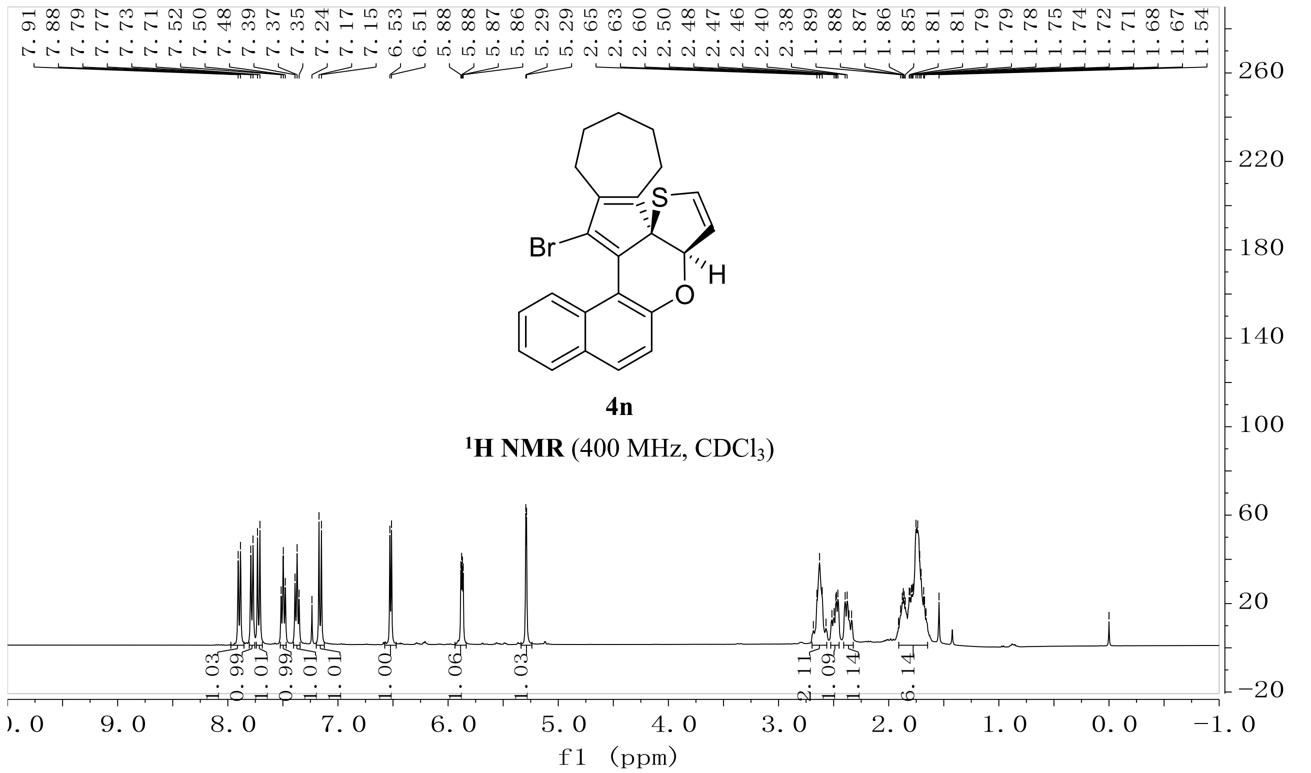
Br
S
H
O
4n
¹H NMR (400 MHz, CDCl3)
f1 (ppm)

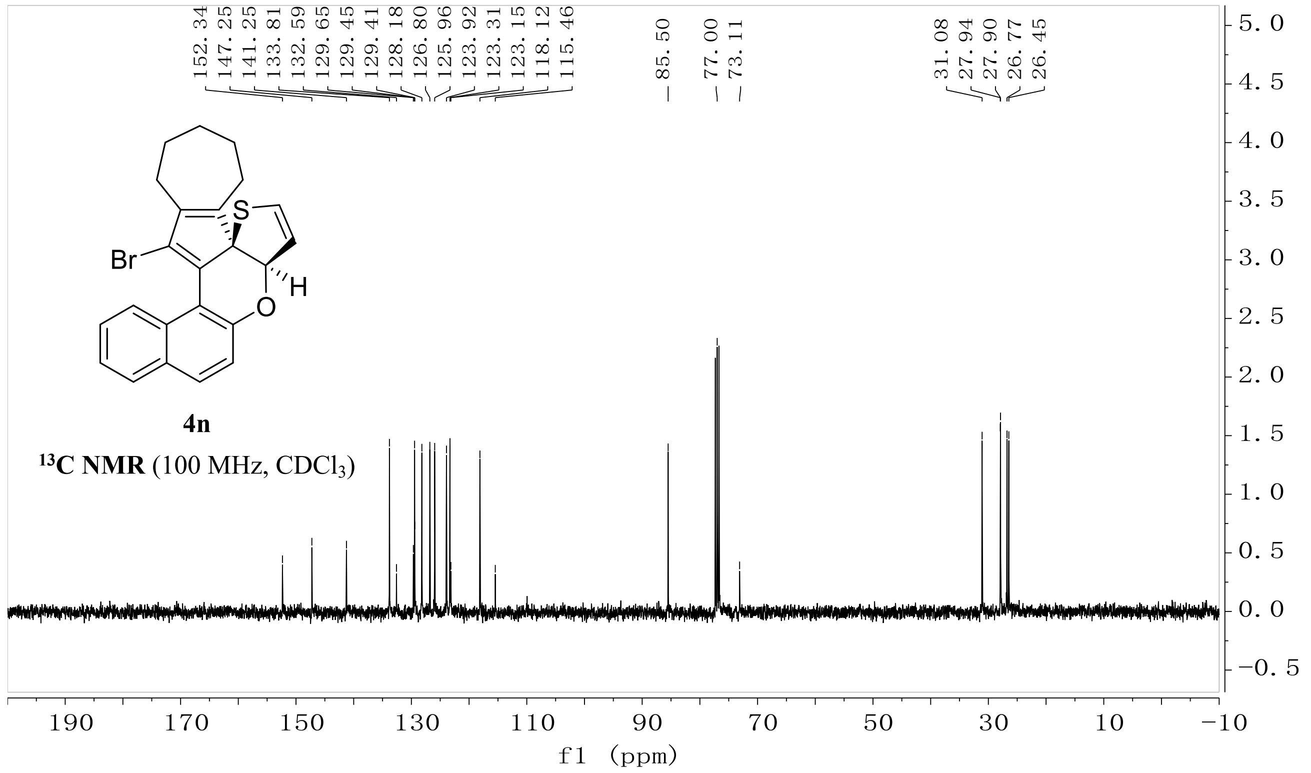
Br
S
H
O
4n
¹³C NMR (100 MHz, CDCl3)
f1 (ppm)

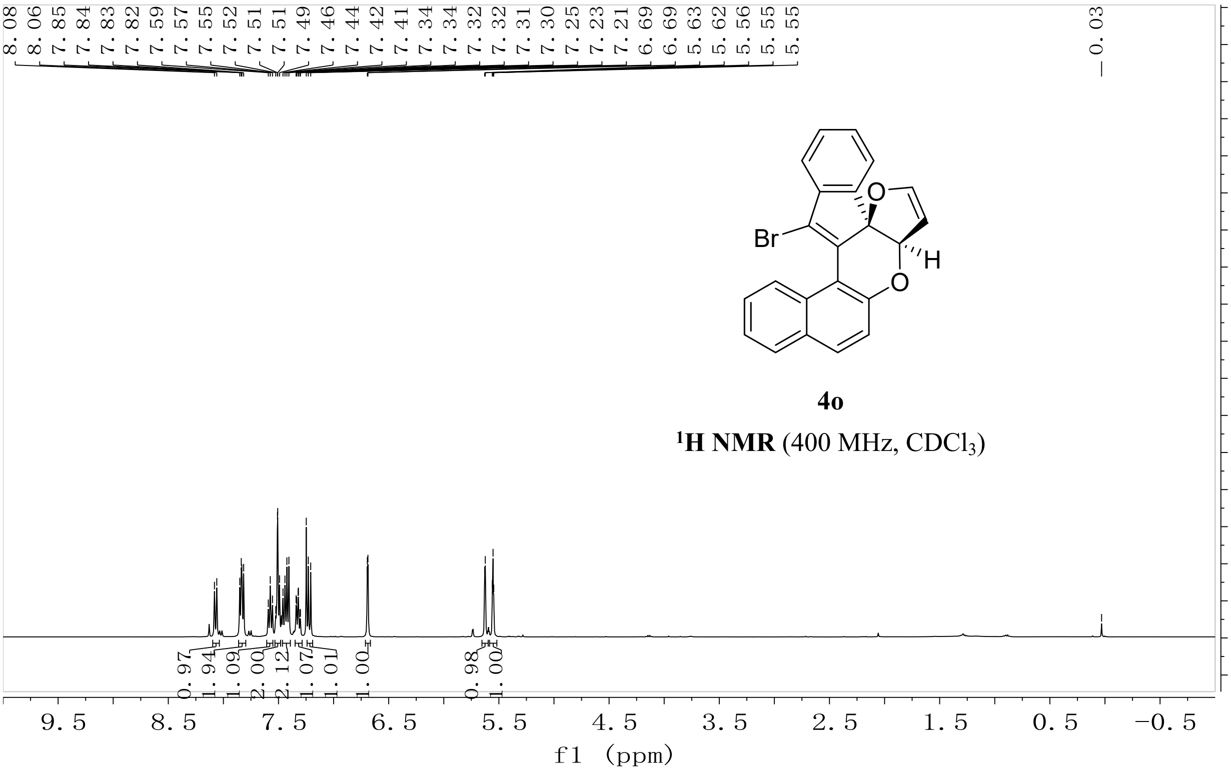

8.08 8.06 7.85 7.84 7.83 7.82 7.59 7.57 7.55 7.52 7.51 7.51 7.49 7.46 7.44 7.42 7.41 7.34 7.34 7.32 7.32 7.31 7.30 7.25 7.23 7.21 6.69 6.69 5.63 5.62 5.56 5.55 5.55
0.03
Br
O
O
H
4o
^{1}H NMR (400 MHz, $CDCl_3$)
0.97 1.94 1.09 2.00 2.12 1.07 1.01 1.00 0.98 1.00
f1 (ppm)

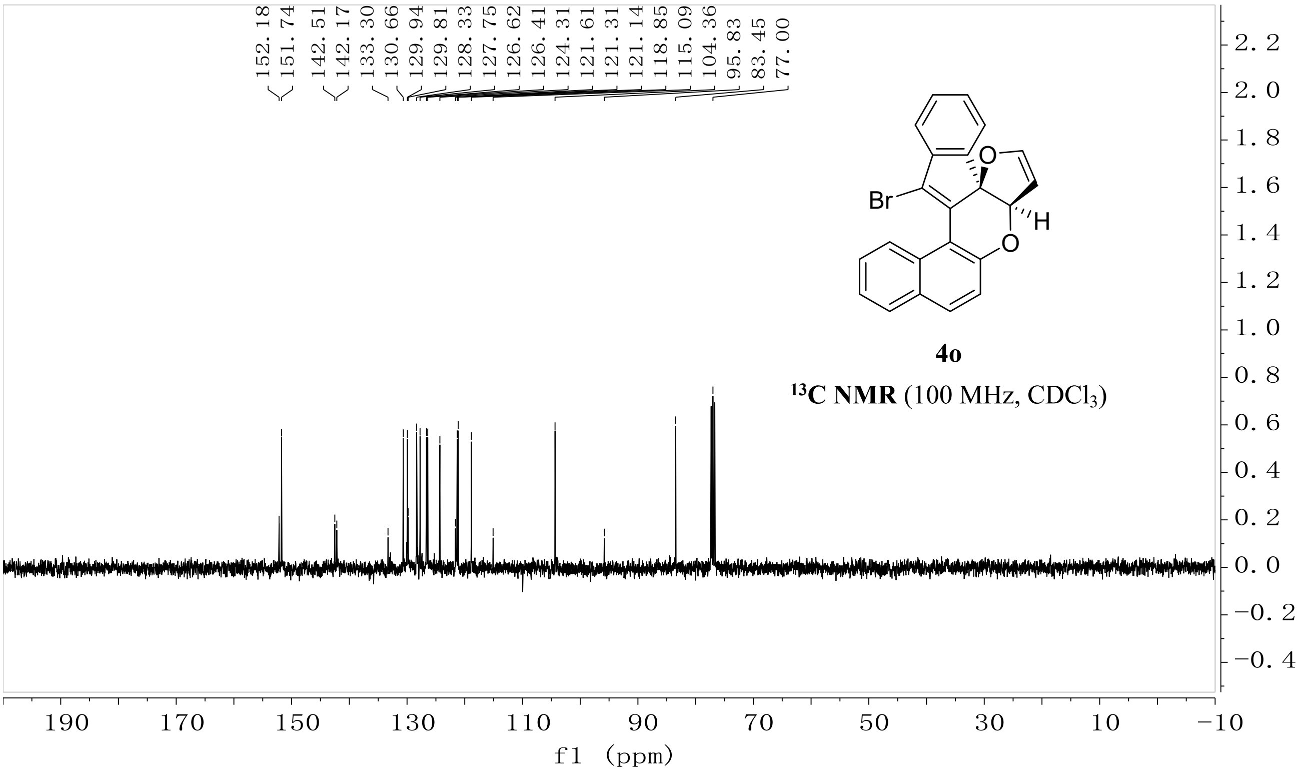

152.18 151.74 142.51 142.17 133.30 130.66 129.94 129.81 128.33 127.75 126.62 126.41 124.31 121.61 121.31 121.14 118.85 115.09 104.36 95.83 83.45 77.00
Br
O
O
H
4o
^{13}C NMR (100 MHz, $CDCl_3$)
f1 (ppm)

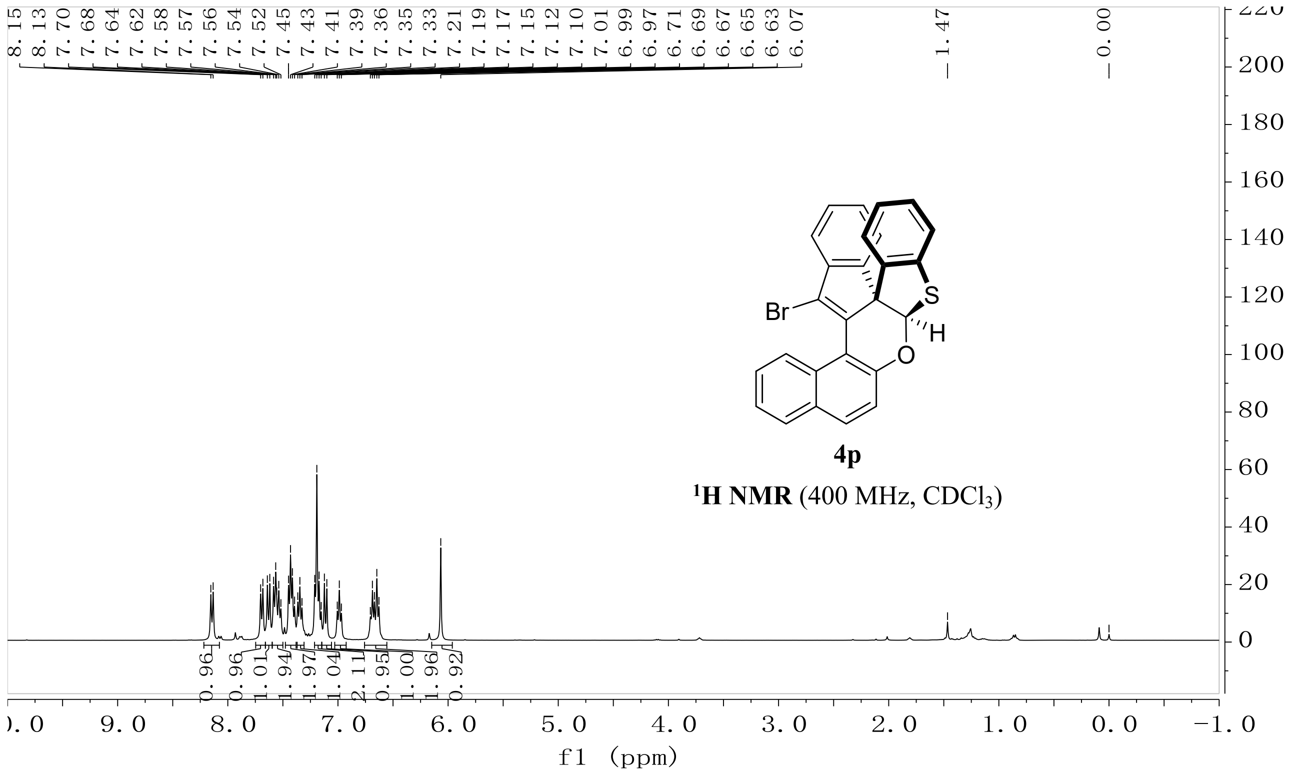

8.15 8.13 7.70 7.68 7.64 7.62 7.58 7.57 7.56 7.54 7.52 7.45 7.43 7.41 7.39 7.36 7.35 7.33 7.21 7.19 7.17 7.15 7.12 7.10 7.01 6.99 6.97 6.71 6.69 6.67 6.65 6.63 6.07
1.47
0.00
0.96 0.96 1.01 1.94 1.97 1.04 2.11 0.95 1.00 1.96 0.92
Br
S
H
O
4p
^{1}H NMR (400 MHz, CDCl$_3$)
f1 (ppm)

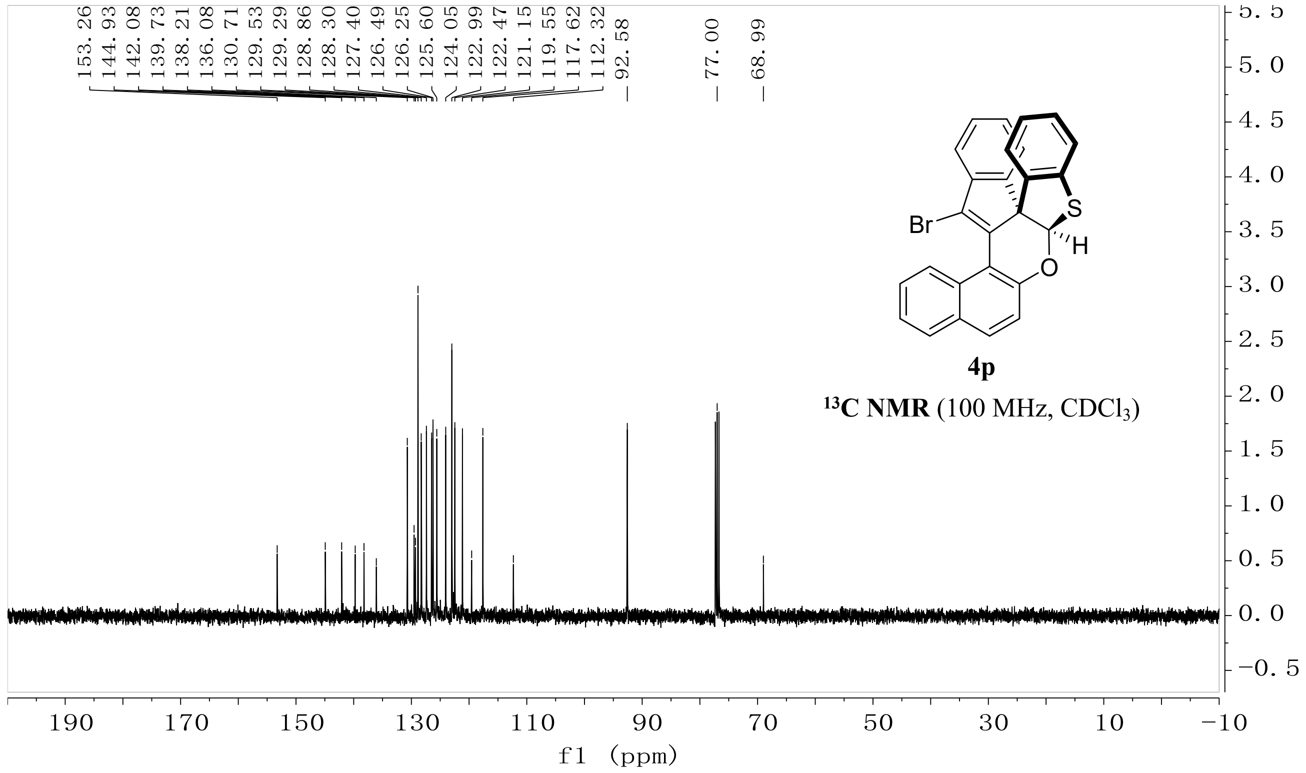

153.26 144.93 142.08 139.73 138.21 136.08 130.71 129.53 129.29 128.86 128.30 127.40 126.49 126.25 125.60 124.05 122.99 122.47 121.15 119.55 117.62 112.32
92.58
77.00
68.99
Br
S
H
O
4p
^{13}C NMR (100 MHz, CDCl$_3$)
f1 (ppm)

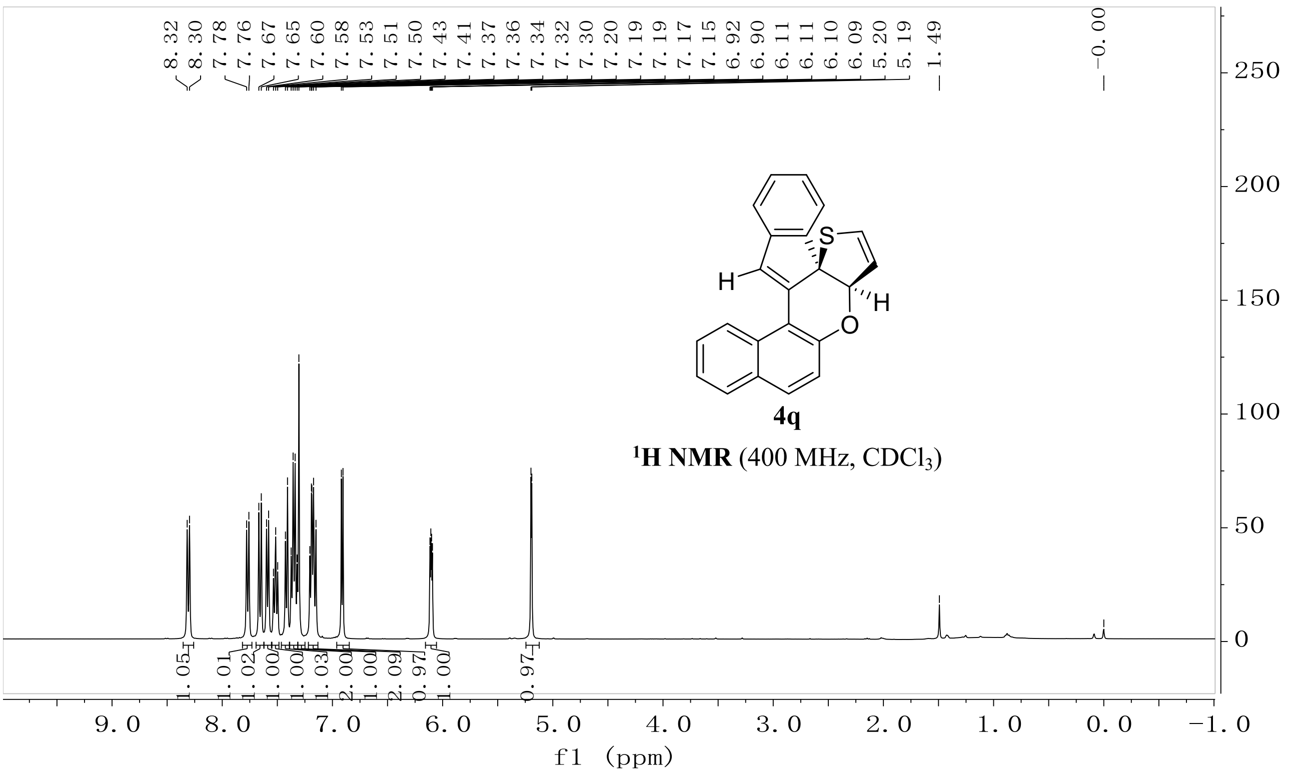
8.32
8.30
7.78
7.76
7.67
7.65
7.60
7.58
7.53
7.51
7.50
7.43
7.41
7.37
7.36
7.34
7.32
7.30
7.20
7.19
7.19
7.17
7.15
6.92
6.90
6.11
6.11
6.10
6.09
5.20
5.19
1.49
-0.00
H
S
H
O
4q
1H NMR (400 MHz, CDCl3)
1.05
1.01
1.02
1.00
1.00
1.00
1.03
2.00
1.00
2.09
0.97
1.00
0.97
9.0
8.0
7.0
6.0
5.0
4.0
3.0
2.0
1.0
0.0
-1.0
f1 (ppm)
250
200
150
100
50
0

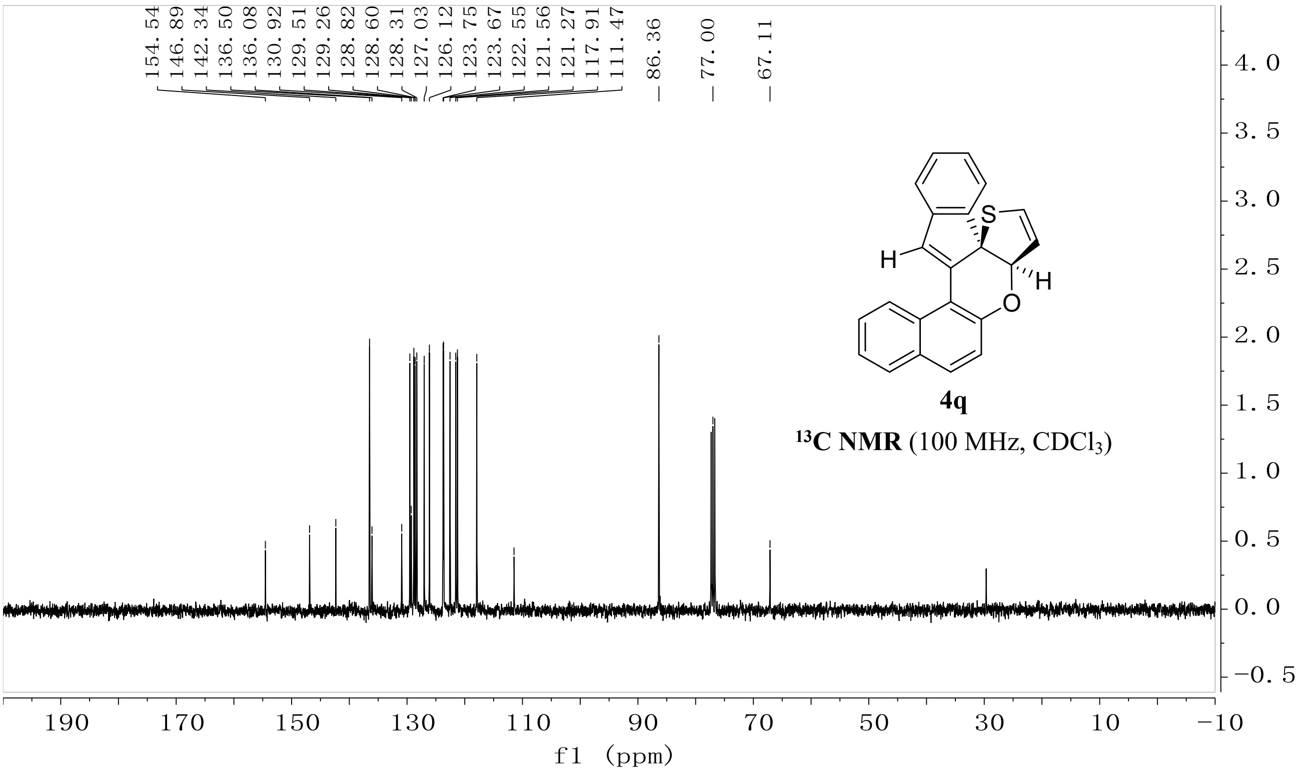
154.54
146.89
142.34
136.50
136.08
130.92
129.51
129.26
128.82
128.60
128.31
127.03
126.12
123.75
123.67
122.55
121.56
121.27
117.91
111.47
86.36
77.00
67.11
H
S
H
O
4q
13C NMR (100 MHz, CDCl3)
190
170
150
130
110
90
70
50
30
10
-10
f1 (ppm)
4.0
3.5
3.0
2.5
2.0
1.5
1.0
0.5
0.0
-0.5

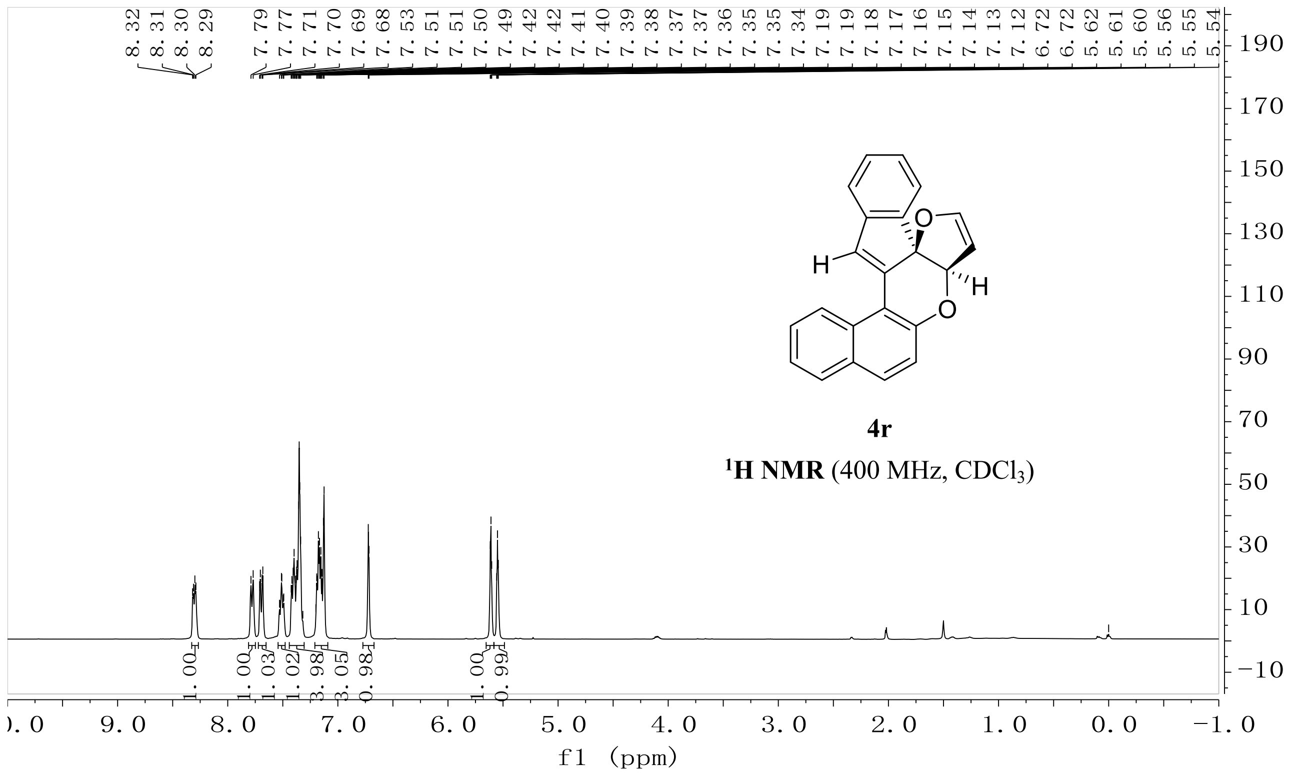
8.32 8.31 8.30 8.29 7.79 7.77 7.71 7.70 7.69 7.68 7.53 7.51 7.51 7.50 7.49 7.42 7.42 7.41 7.40 7.39 7.38 7.37 7.37 7.36 7.35 7.35 7.34 7.19 7.19 7.18 7.17 7.16 7.15 7.14 7.13 7.12 6.72 6.72 5.62 5.61 5.60 5.56 5.55 5.54
H
O
H
O
4r
1H NMR (400 MHz, CDCl3)
1.00 1.00 1.03 1.02 3.98 3.05 0.98 1.00 0.99
f1 (ppm)

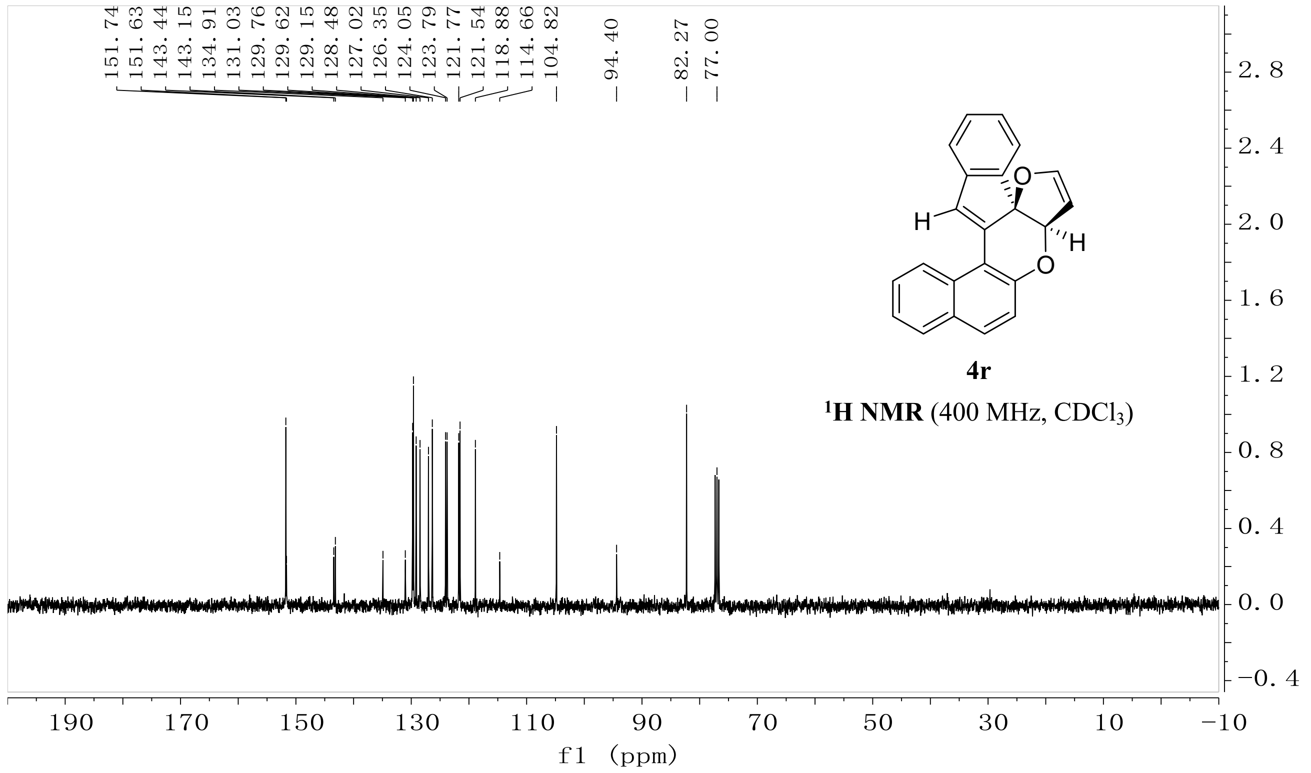
151.74 151.63 143.44 143.15 134.91 131.03 129.76 129.62 129.15 128.48 127.02 126.35 124.05 123.79 121.77 121.54 118.88 114.66 104.82 94.40 82.27 77.00
H
O
H
O
4r
1H NMR (400 MHz, CDCl3)
f1 (ppm)

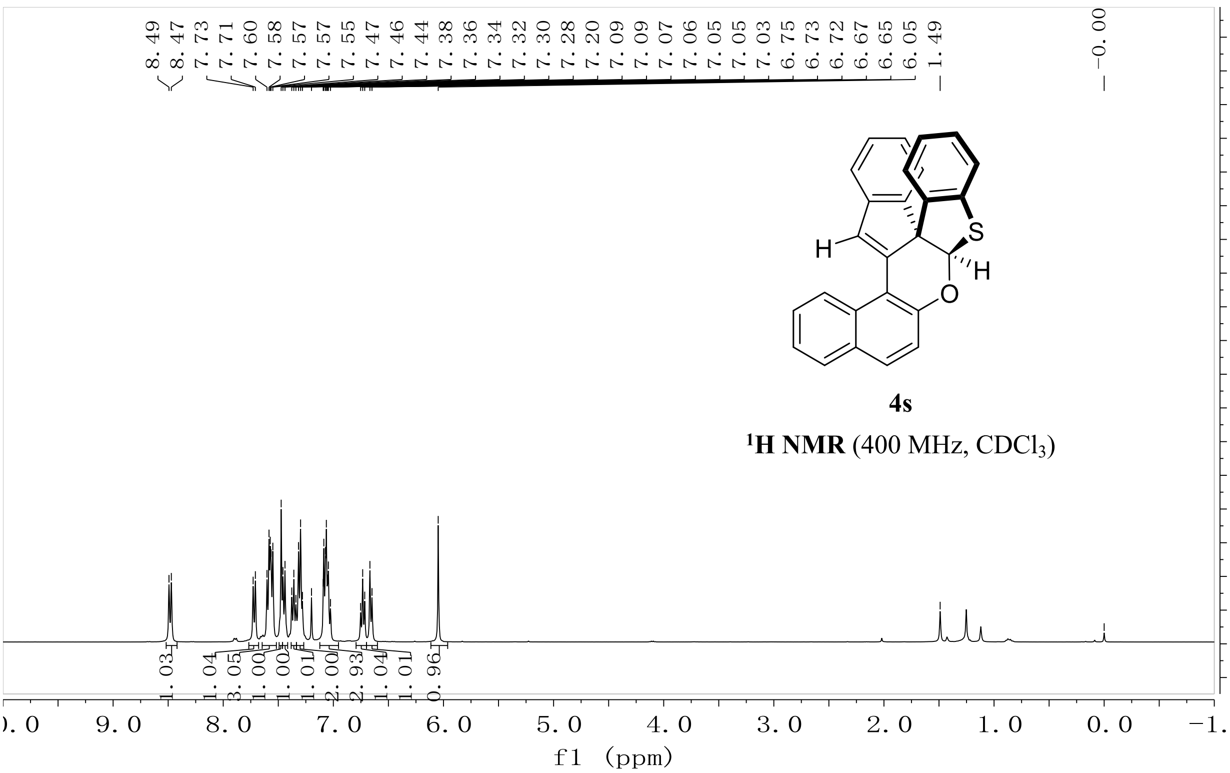

8.49
8.47
7.73
7.71
7.60
7.58
7.57
7.57
7.55
7.47
7.46
7.44
7.38
7.36
7.34
7.32
7.30
7.28
7.20
7.09
7.09
7.07
7.06
7.05
7.05
7.03
6.75
6.73
6.72
6.67
6.65
6.05
1.49
-0.00
S
H
H
O
4s
1H NMR (400 MHz, CDCl3)
1.03
1.04
3.05
1.00
1.00
1.01
2.00
2.93
1.04
1.01
0.96
f1 (ppm)

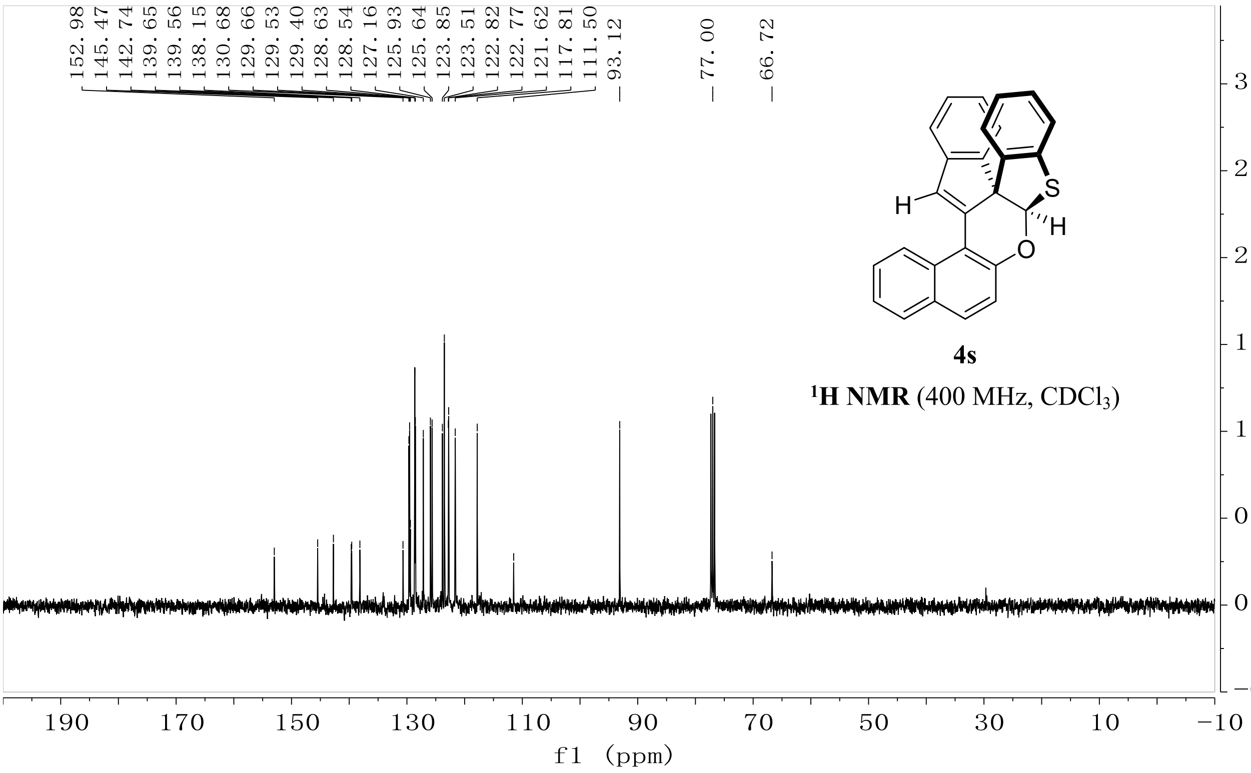

152.98
145.47
142.74
139.65
139.56
138.15
130.68
129.66
129.53
129.40
128.63
128.54
127.16
125.93
125.64
123.85
123.51
122.82
122.77
121.62
117.81
111.50
93.12
77.00
66.72
S
H
H
O
4s
1H NMR (400 MHz, CDCl3)
f1 (ppm)

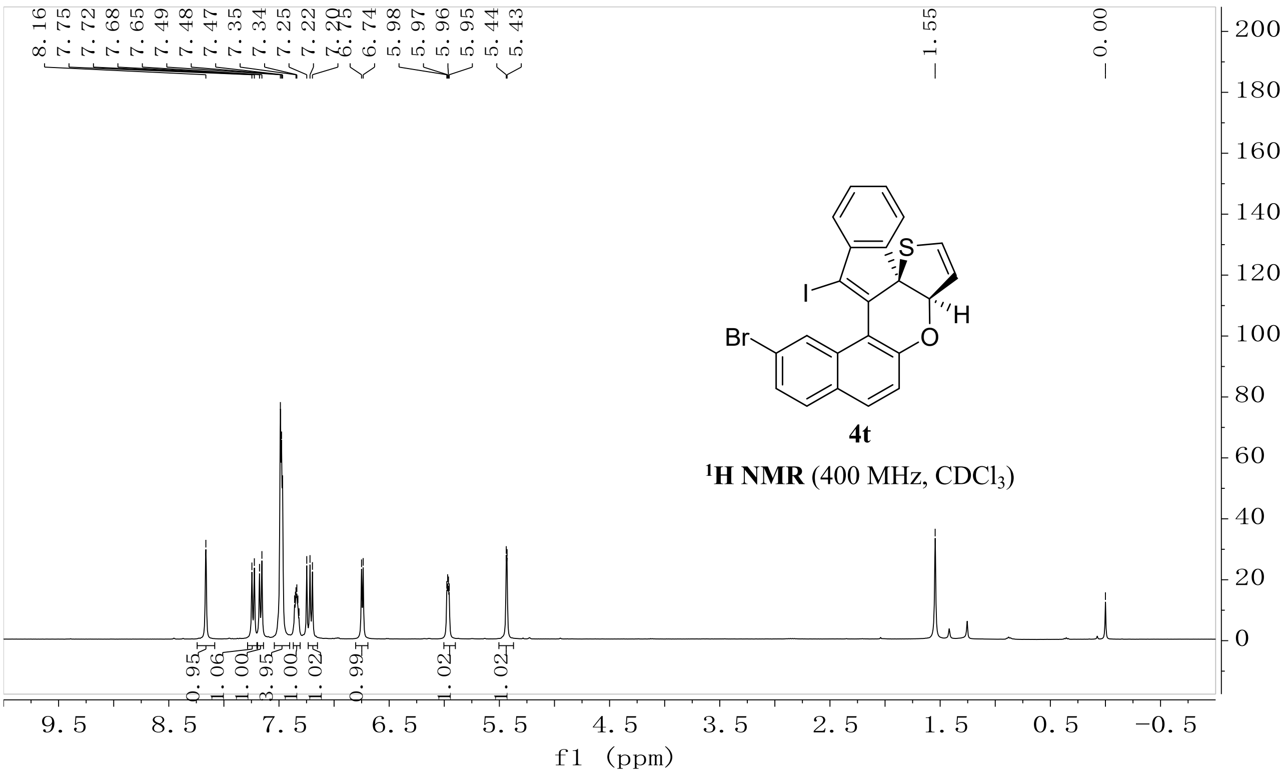
8.16
7.75
7.72
7.68
7.65
7.49
7.48
7.47
7.35
7.34
7.25
7.22
7.20
6.75
6.74
5.98
5.97
5.96
5.95
5.44
5.43
1.55
0.00
0.95
1.06
1.00
3.95
1.00
1.02
0.99
1.02
1.02
Br
I
S
O
H
4t
1H NMR (400 MHz, CDCl3)
f1 (ppm)

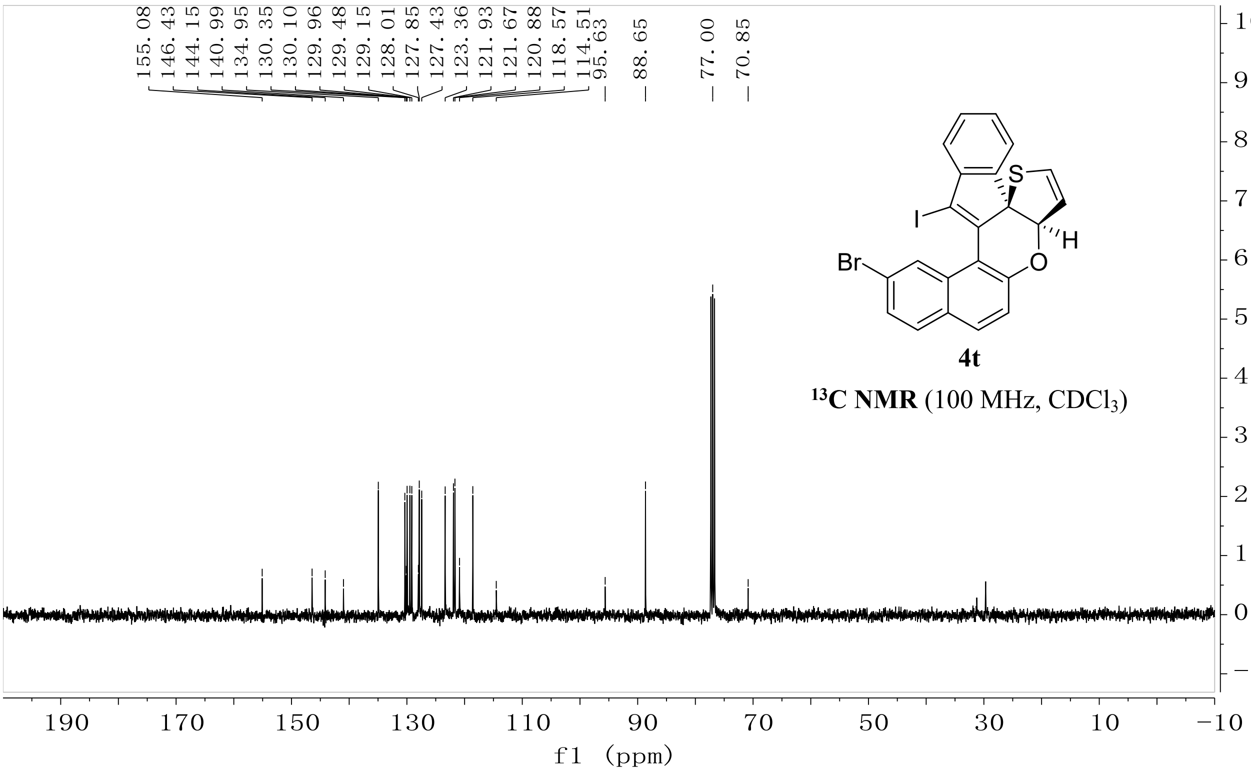
155.08
146.43
144.15
140.99
134.95
130.35
130.10
129.96
129.48
129.15
128.01
127.85
127.43
123.36
121.93
121.67
120.88
118.57
114.51
95.63
88.65
77.00
70.85
Br
I
S
O
H
4t
13C NMR (100 MHz, CDCl3)
f1 (ppm)

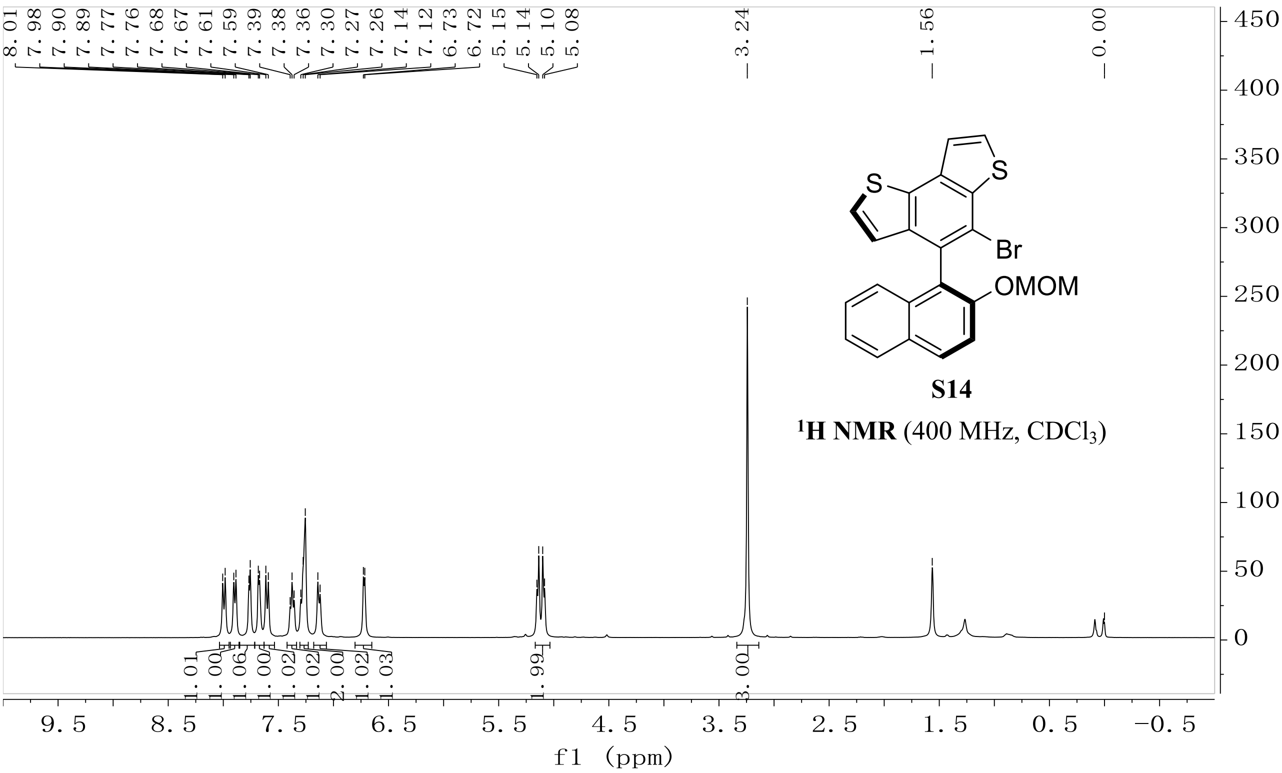

S14

^{1}H NMR (400 MHz, $CDCl_3$)

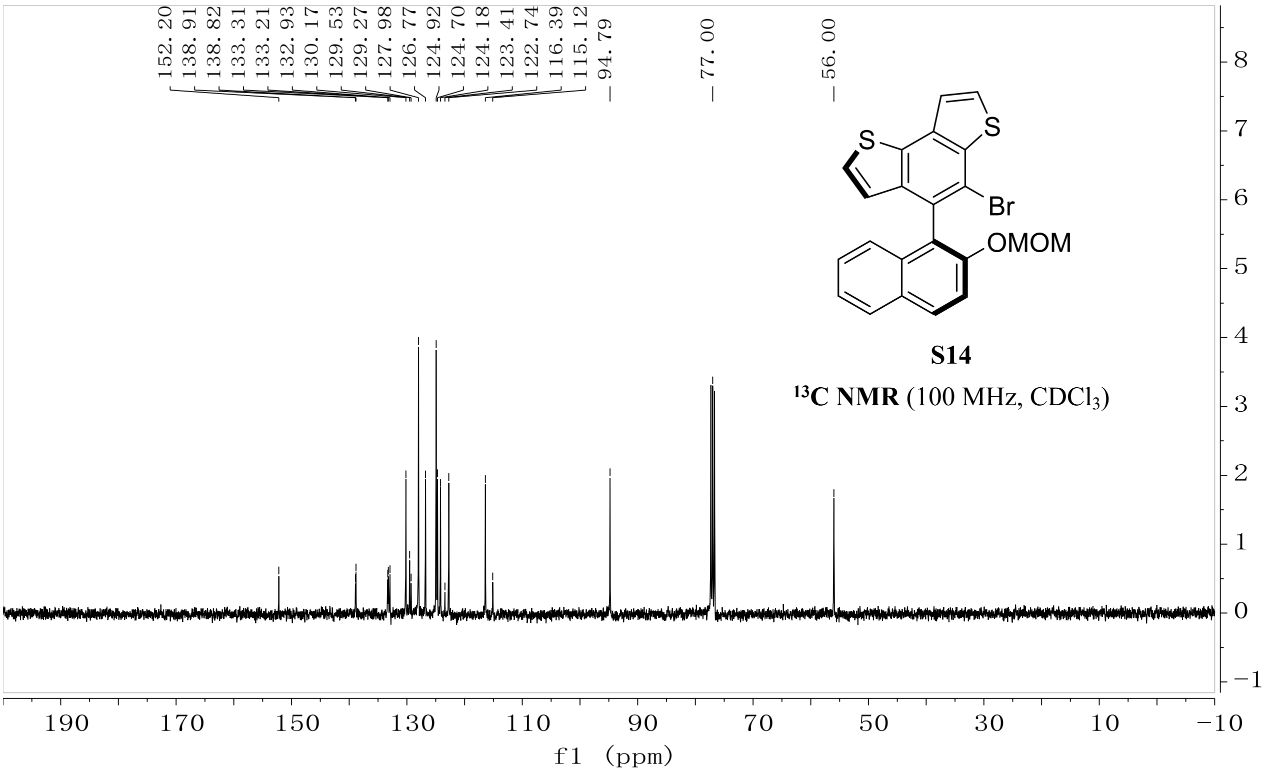

S14

^{13}C NMR (100 MHz, $CDCl_3$)

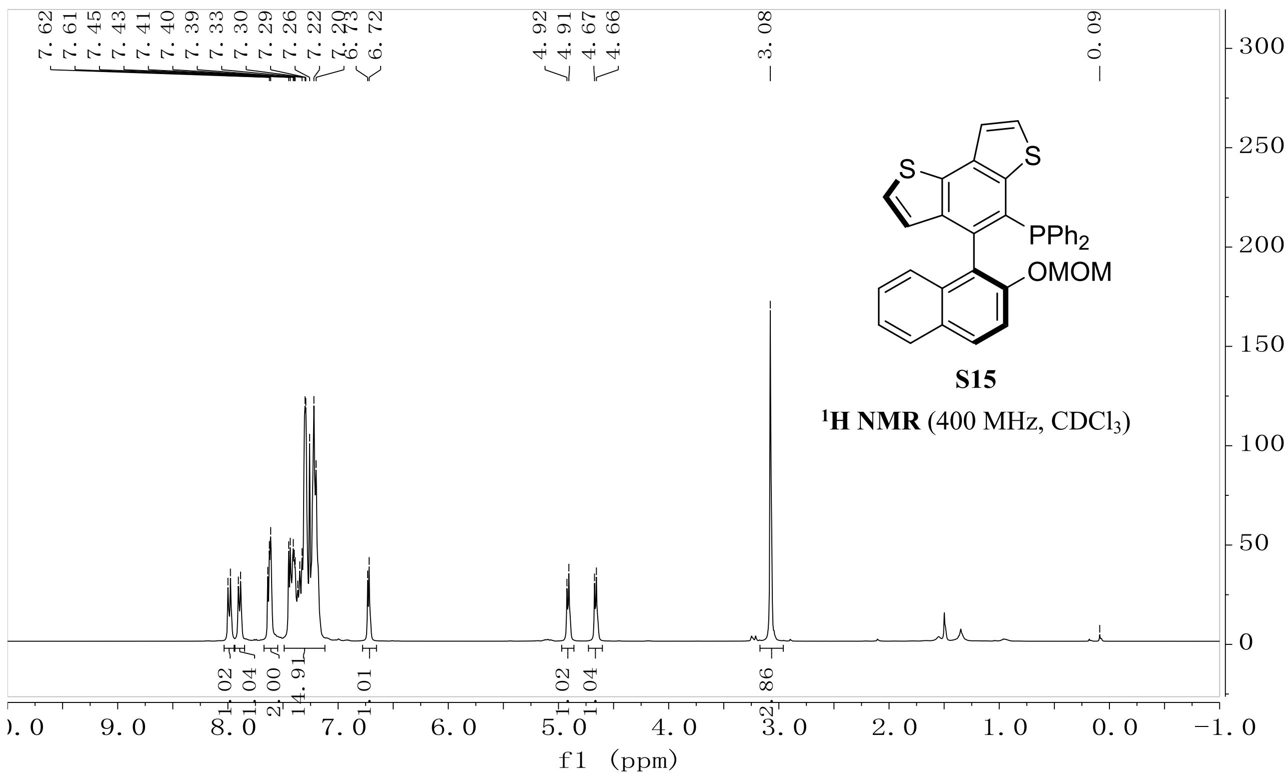
S15
¹H NMR (400 MHz, CDCl₃)
f1 (ppm)

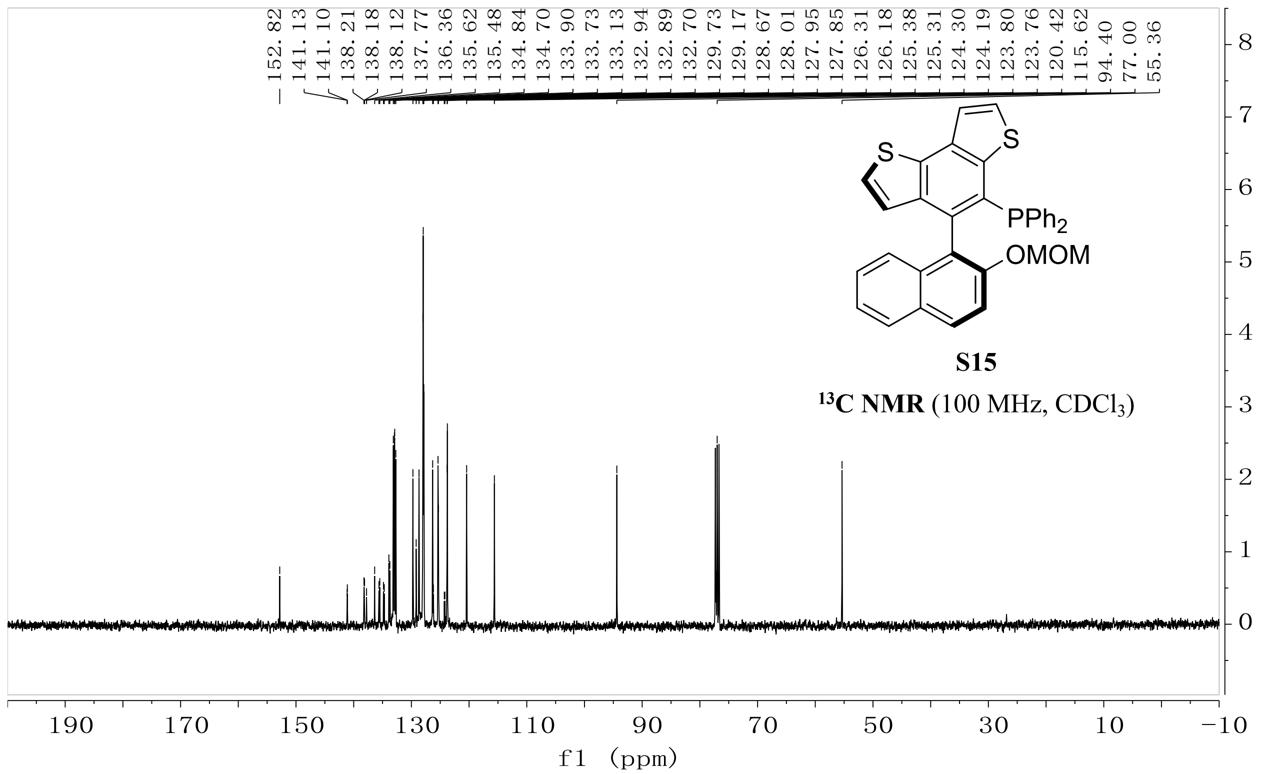
S15
¹³C NMR (100 MHz, CDCl₃)
f1 (ppm)

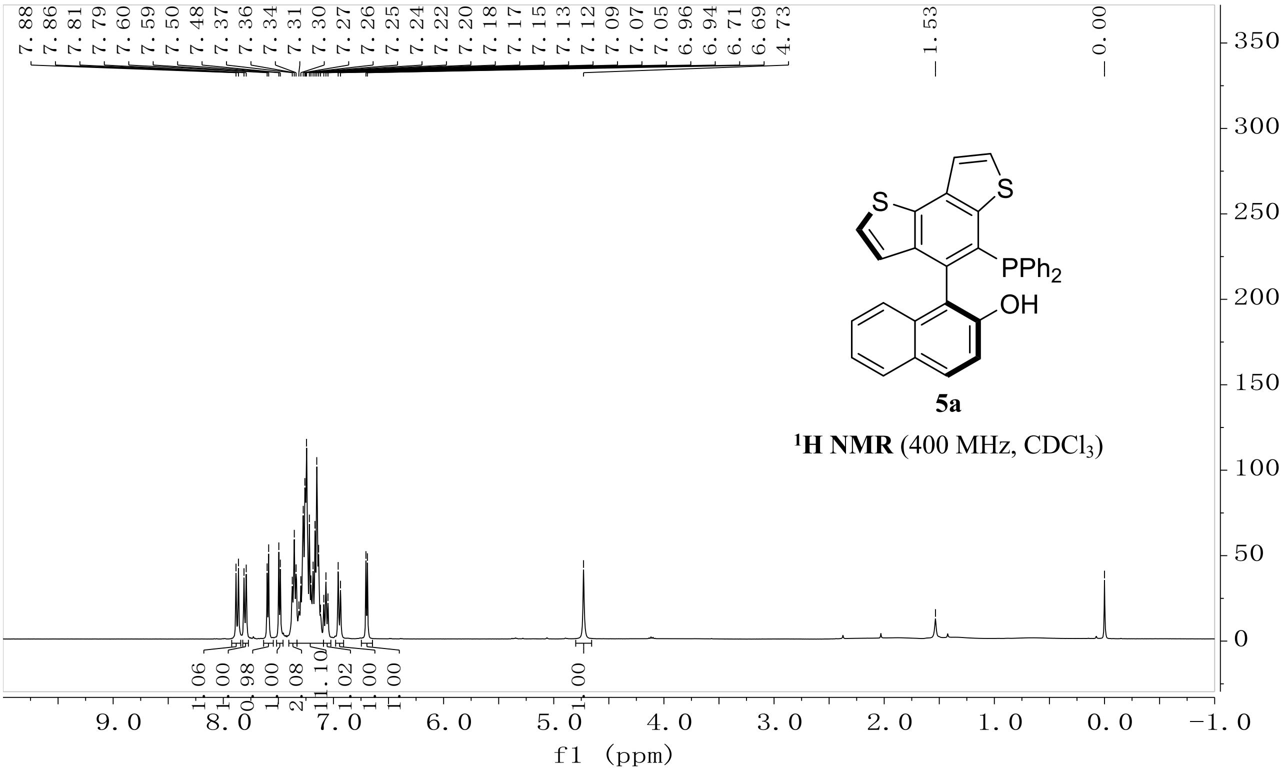
S
S
PPh2
OH
5a
1H NMR (400 MHz, CDCl3)
f1 (ppm)

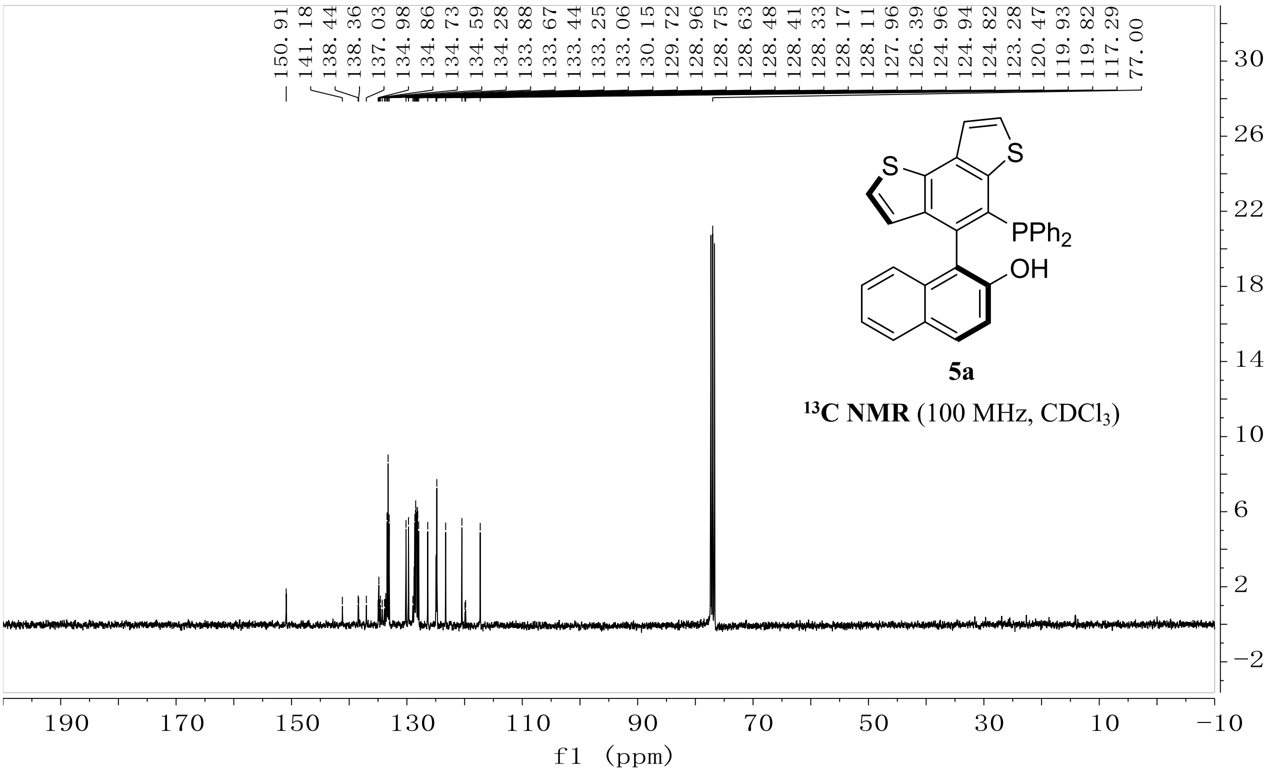
S
S
PPh2
OH
5a
13C NMR (100 MHz, CDCl3)
f1 (ppm)

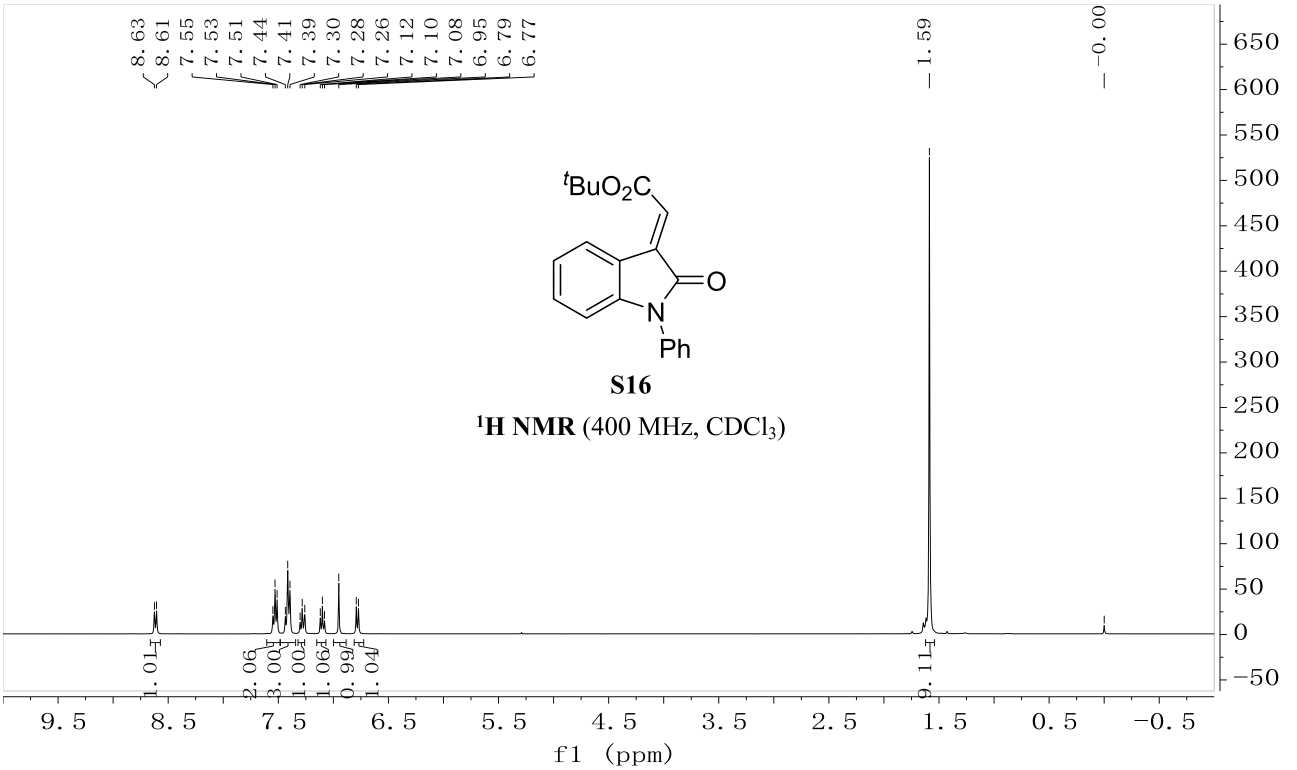
8.63
8.61
7.55
7.53
7.51
7.44
7.41
7.39
7.30
7.28
7.26
7.12
7.10
7.08
6.95
6.79
6.77
1.59
-0.00
tBuO2C
O
N
Ph
S16
1H NMR (400 MHz, CDCl3)
1.01
2.06
3.00
1.00
1.06
0.99
1.04
9.11
f1 (ppm)

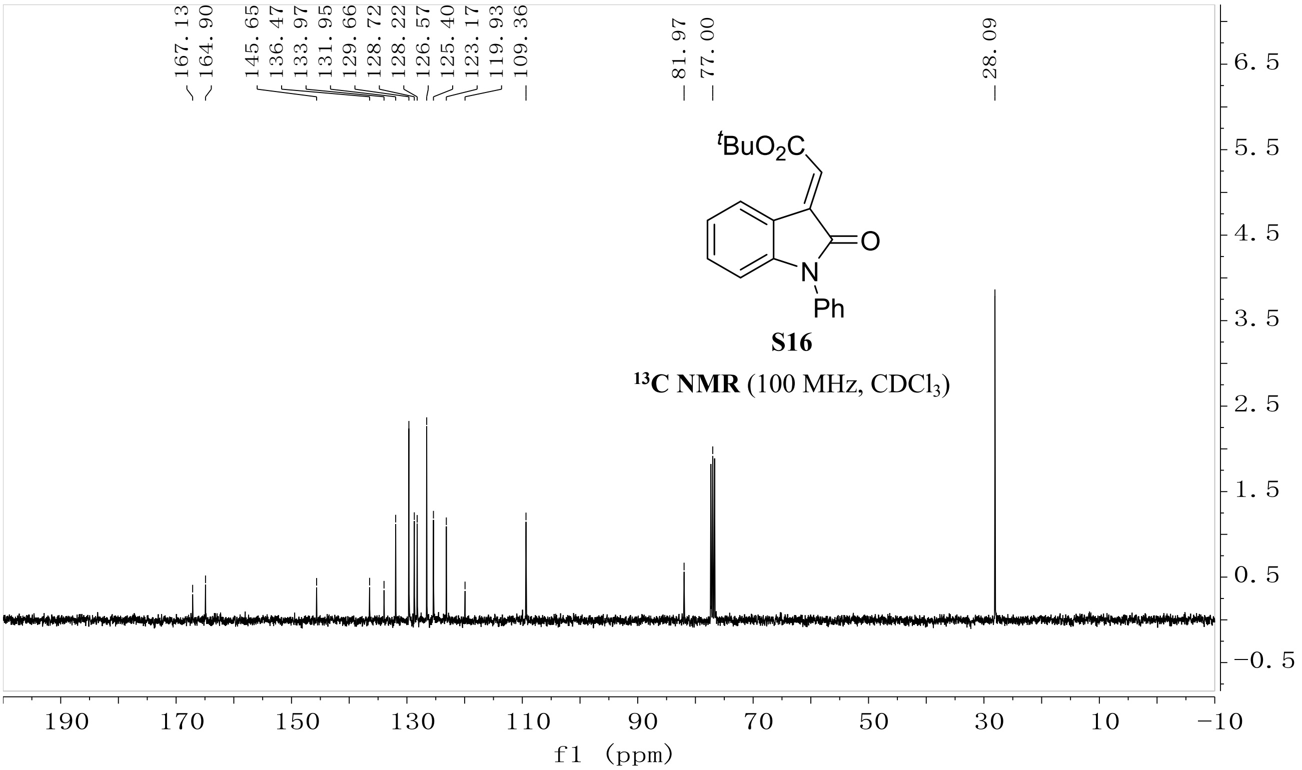
167.13
164.90
145.65
136.47
133.97
131.95
129.66
128.72
128.22
126.57
125.40
123.17
119.93
109.36
81.97
77.00
28.09
tBuO2C
O
N
Ph
S16
13C NMR (100 MHz, CDCl3)
f1 (ppm)

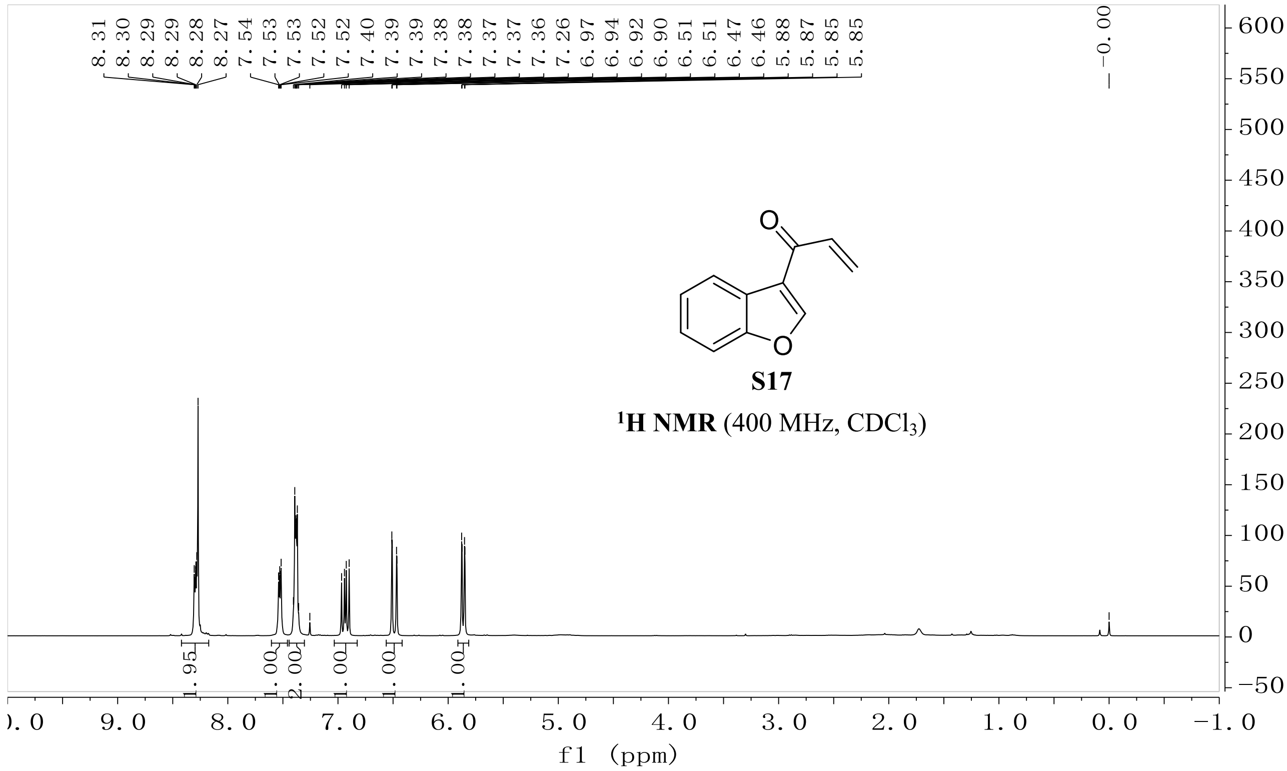
8.31 8.30 8.29 8.29 8.28 8.27 7.54 7.53 7.53 7.52 7.52 7.40 7.39 7.39 7.38 7.38 7.37 7.37 7.36 7.26 6.97 6.94 6.92 6.90 6.51 6.51 6.47 6.46 5.88 5.87 5.85 5.85
-0.00
O
O
S17
1H NMR (400 MHz, CDCl3)
1.95 1.00 2.00 1.00 1.00 1.00
f1 (ppm)

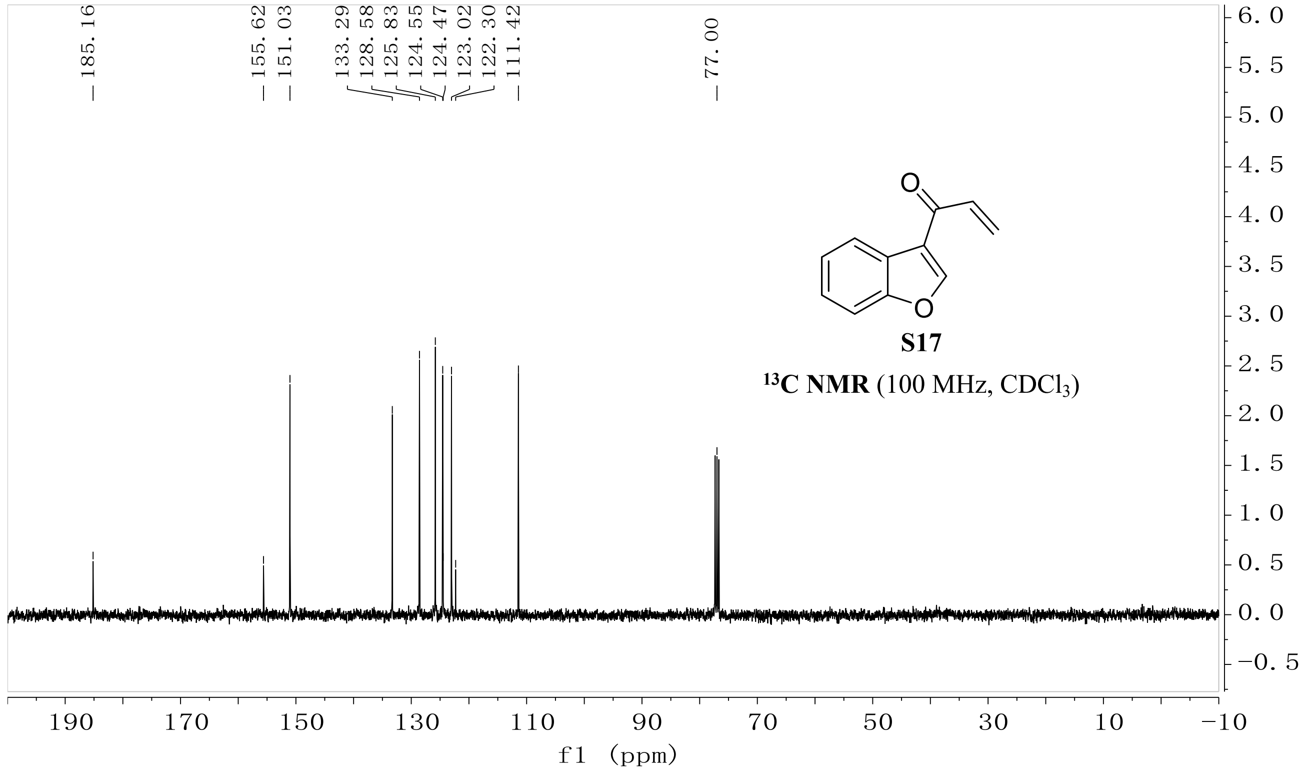
185.16
155.62
151.03
133.29 128.58 125.83 124.55 124.47 123.02 122.30 111.42
77.00
O
O
S17
13C NMR (100 MHz, CDCl3)
f1 (ppm)

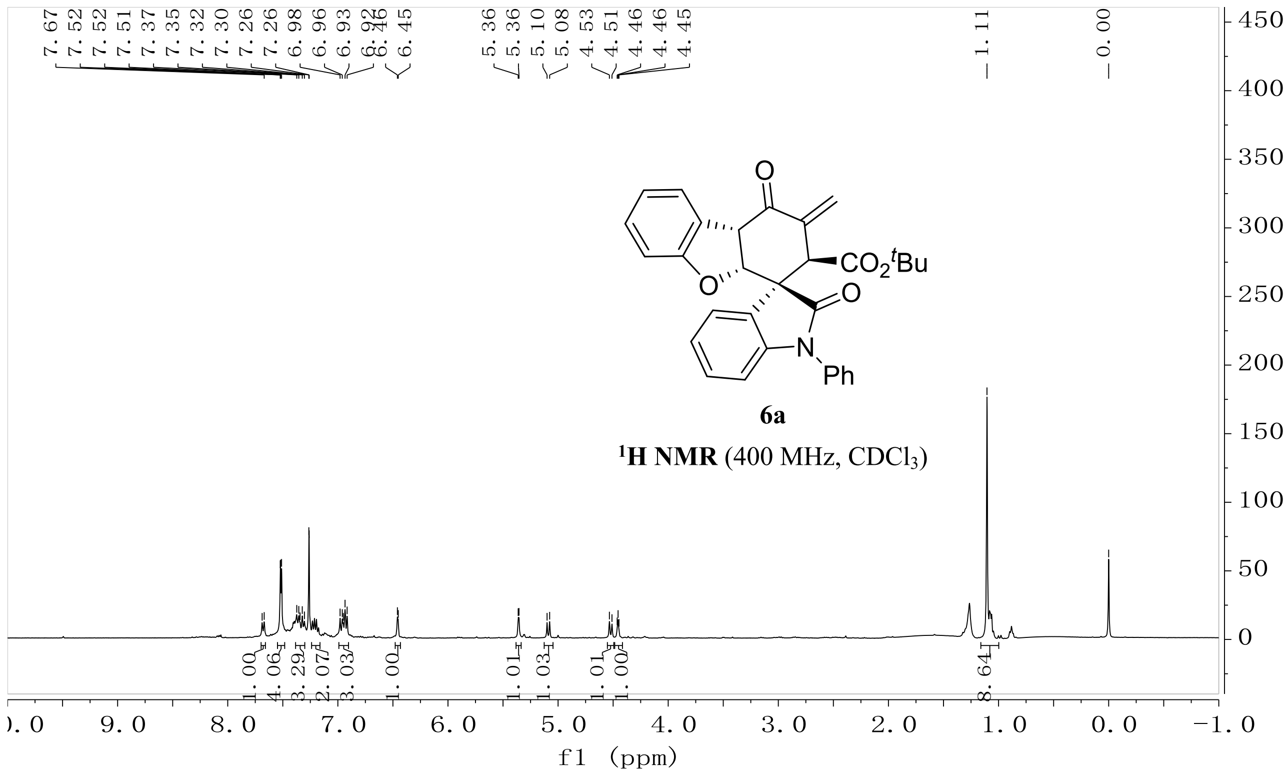
6a
^{1}H NMR (400 MHz, CDCl$_3$)
f1 (ppm)

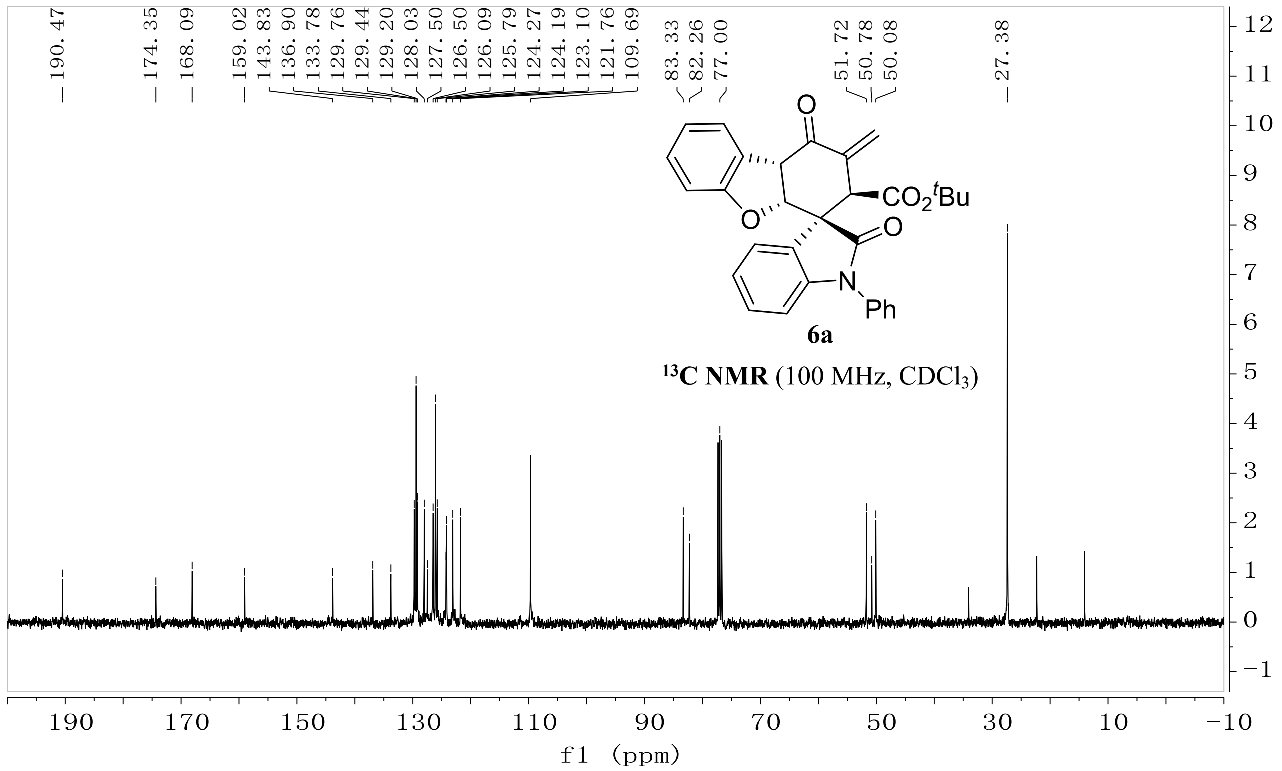
6a
^{13}C NMR (100 MHz, CDCl$_3$)
f1 (ppm)

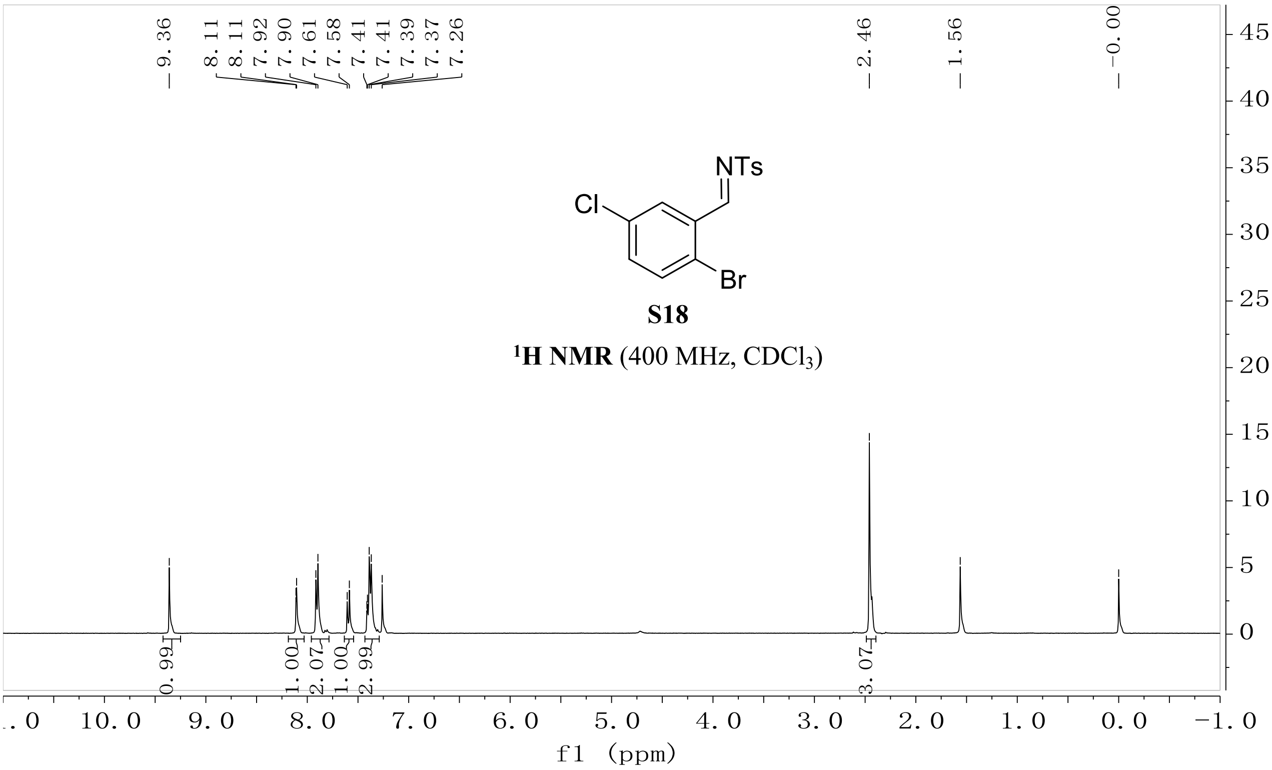
9.36
8.11
8.11
7.92
7.90
7.61
7.58
7.41
7.41
7.39
7.37
7.26
2.46
1.56
-0.00
NTs
Cl
Br
S18
¹H NMR (400 MHz, CDCl3)
0.99
1.00
2.07
1.00
2.99
3.07
f1 (ppm)

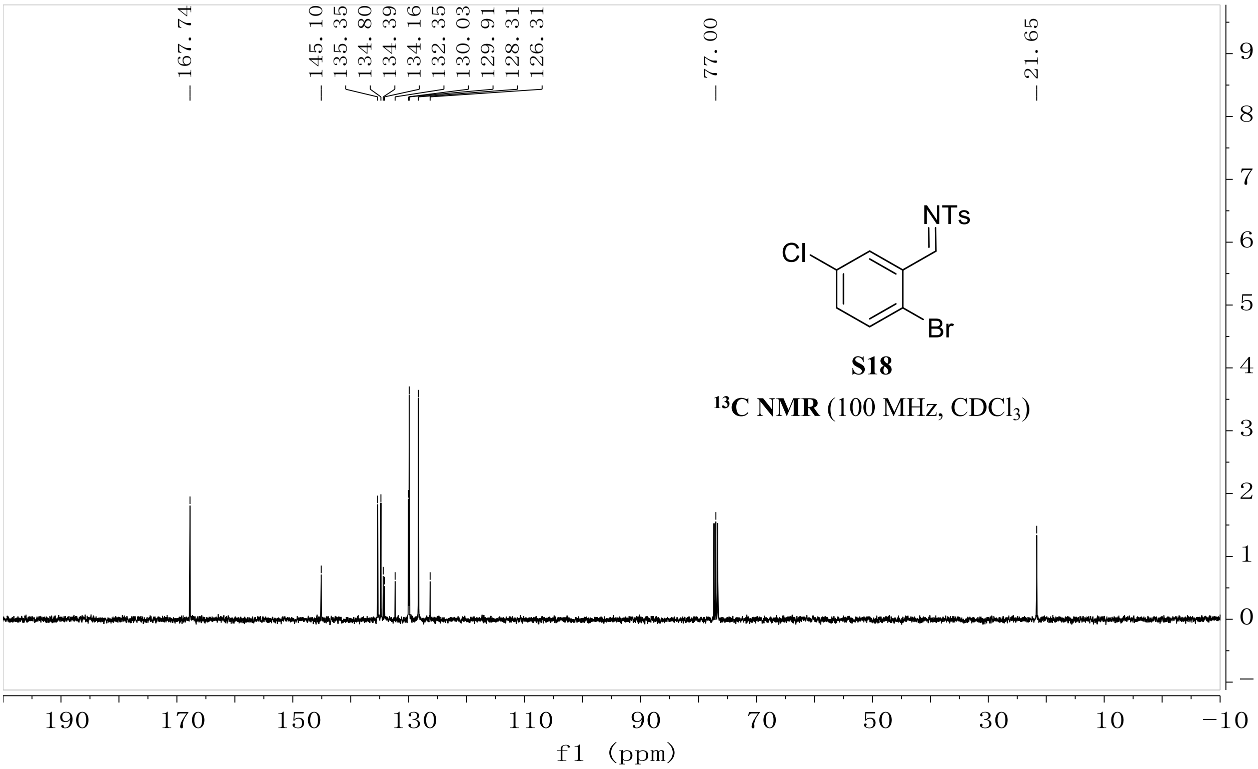
167.74
145.10
135.35
134.80
134.39
134.16
132.35
130.03
129.91
128.31
126.31
77.00
21.65
NTs
Cl
Br
S18
¹³C NMR (100 MHz, CDCl3)
f1 (ppm)

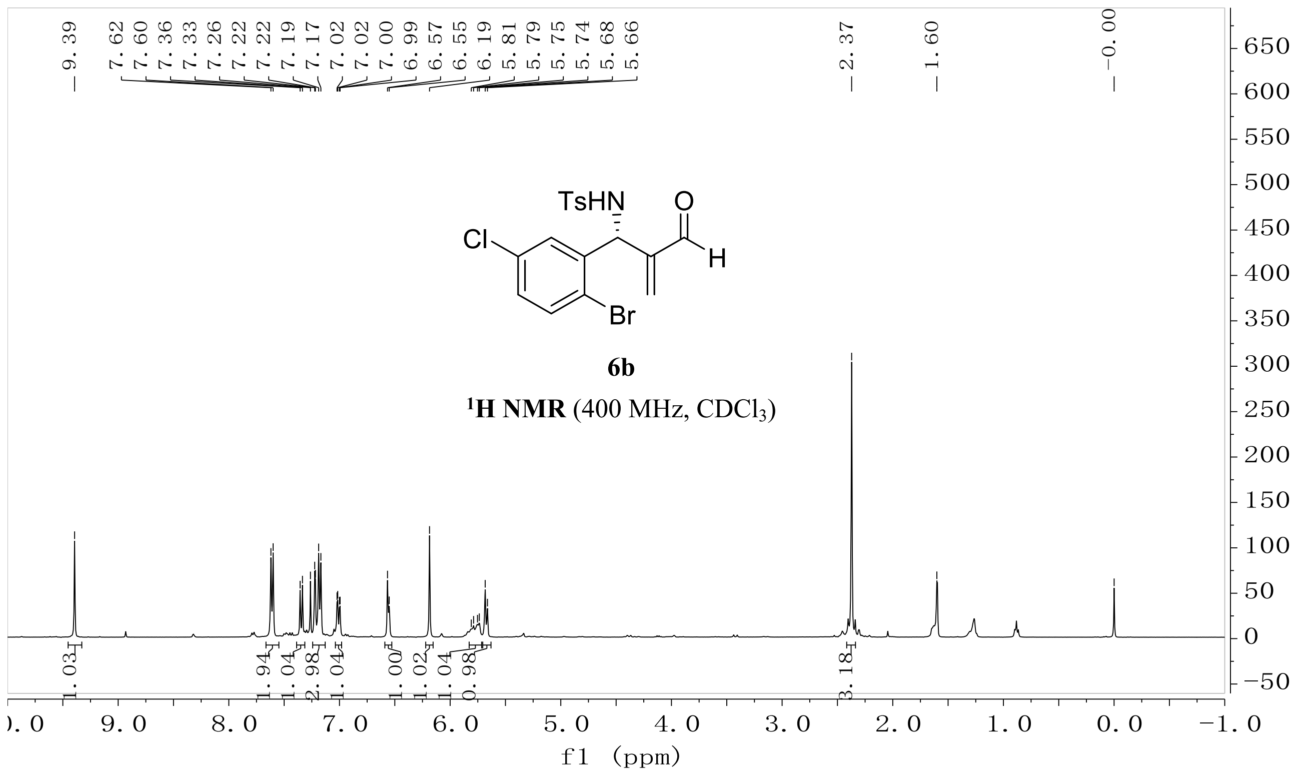

6b

^{1}H NMR (400 MHz, $CDCl_3$)

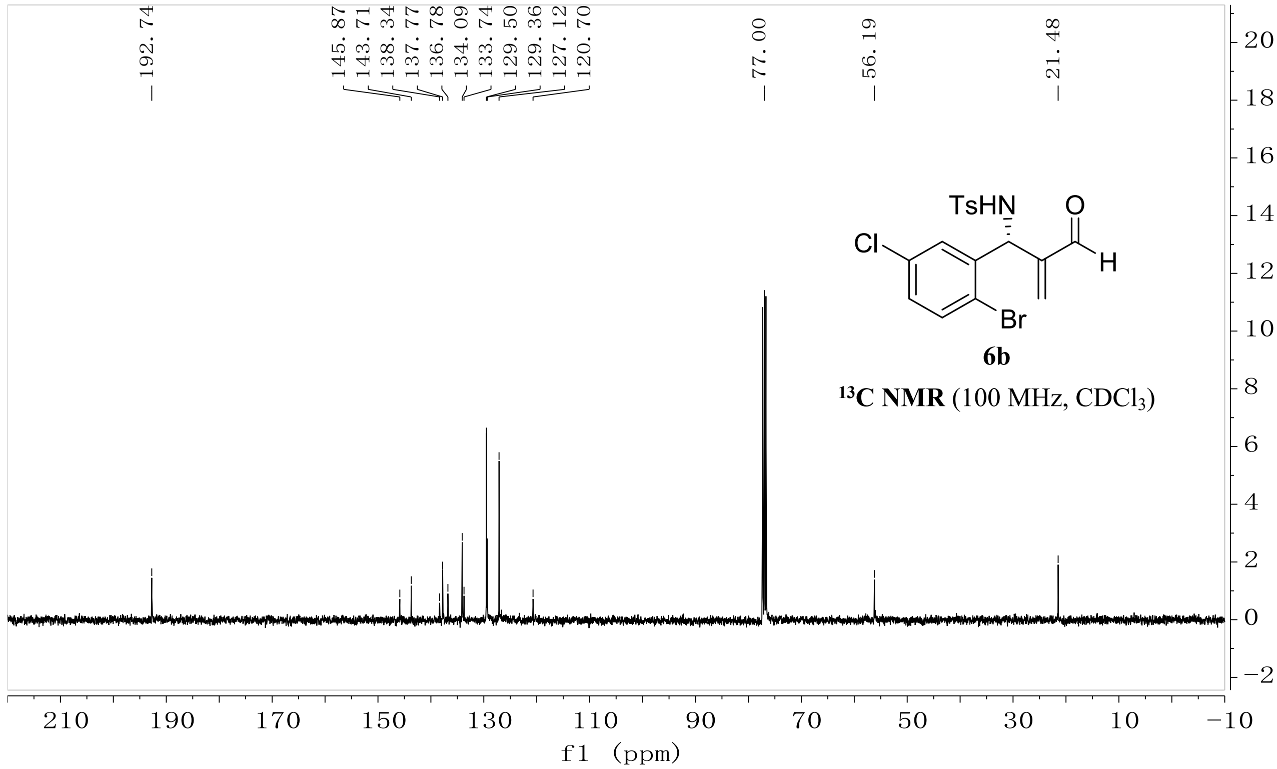

6b

^{13}C NMR (100 MHz, $CDCl_3$)

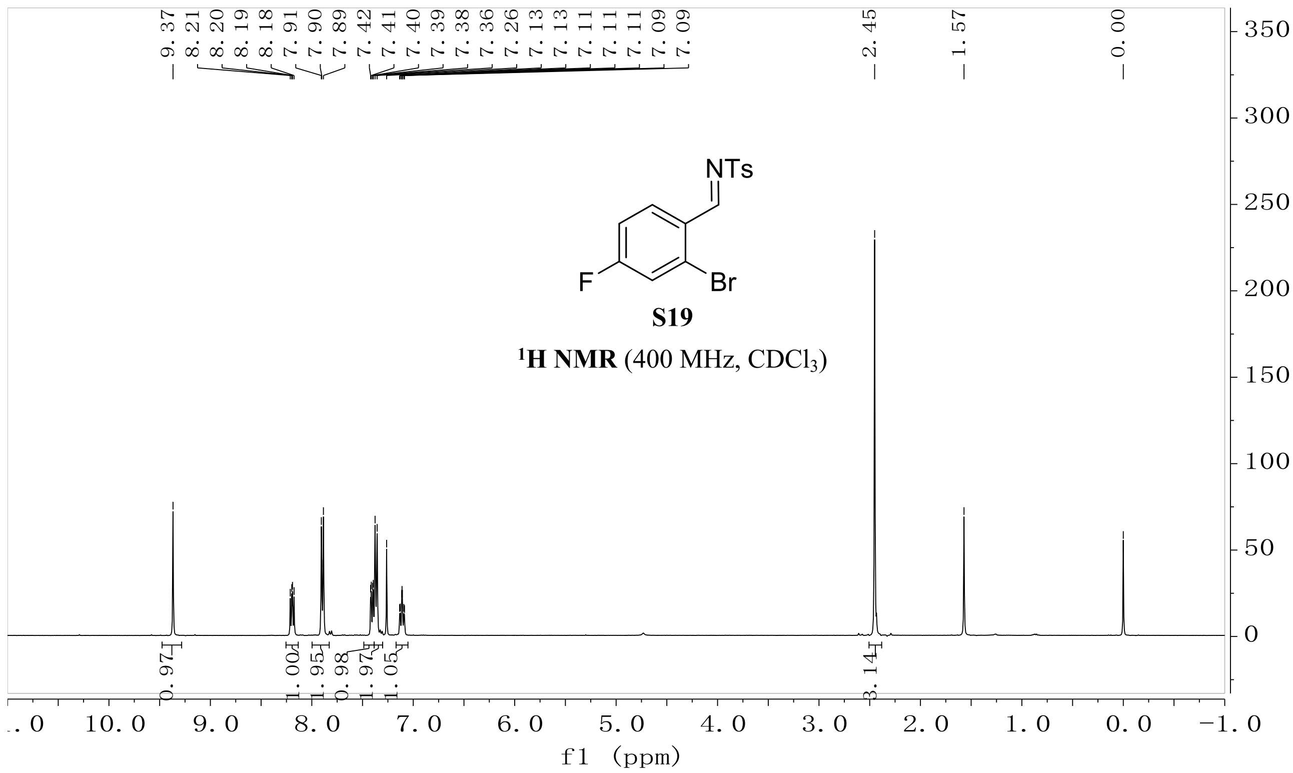
NTs
F
Br
S19
1H NMR (400 MHz, CDCl3)
f1 (ppm)

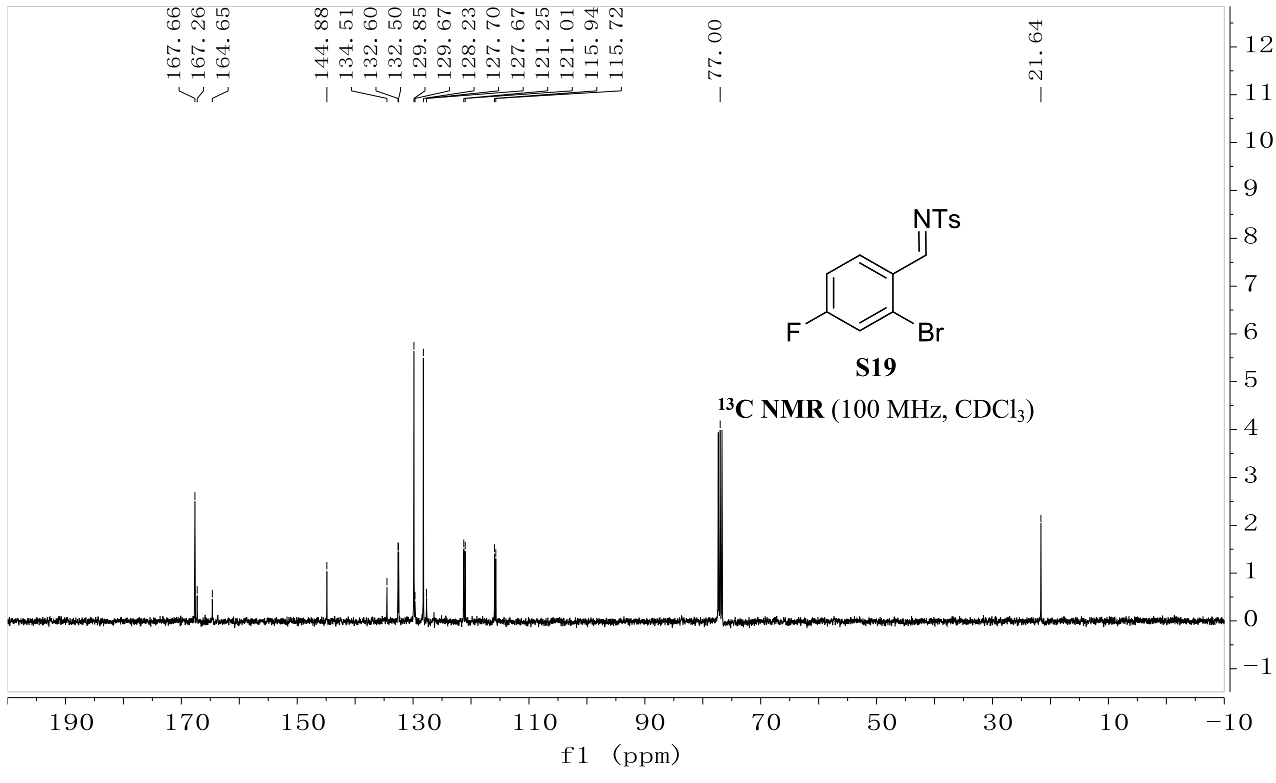
NTs
F
Br
S19
13C NMR (100 MHz, CDCl3)
f1 (ppm)

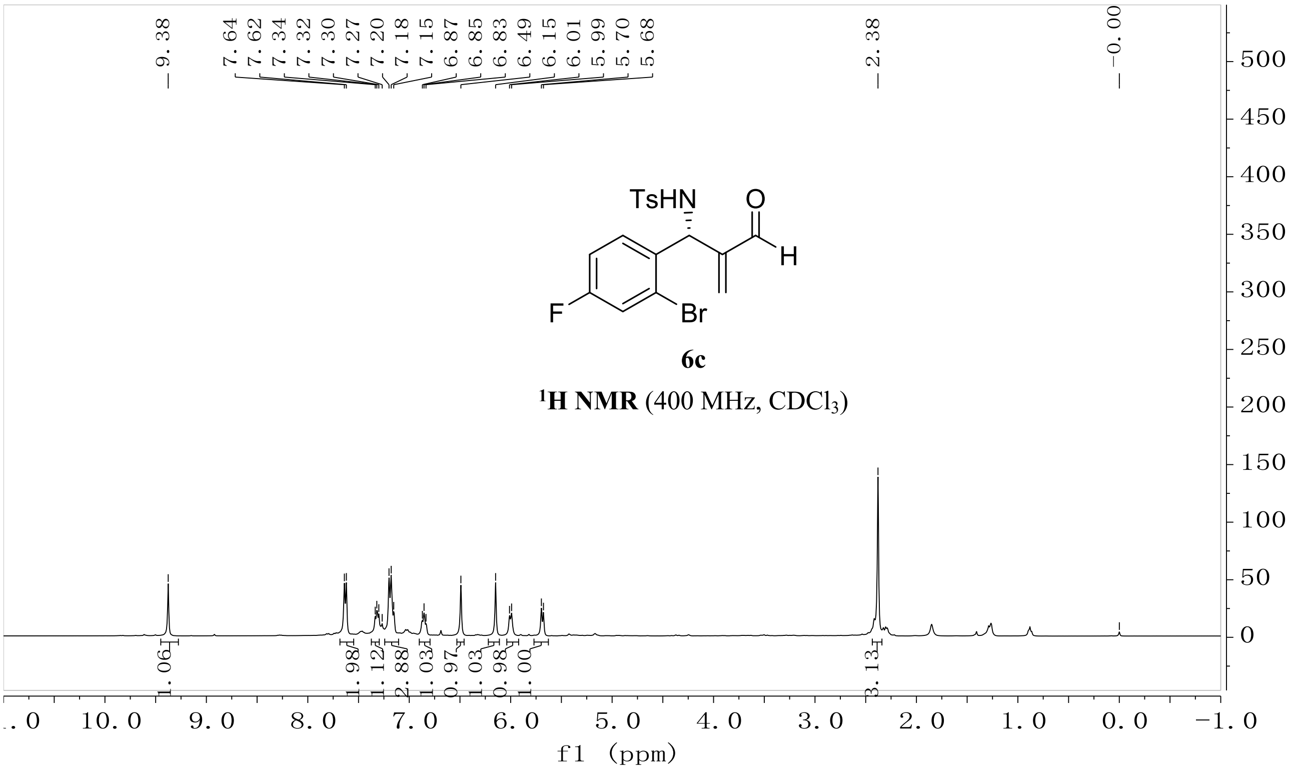

6c

^{1}H NMR (400 MHz, $CDCl_3$)

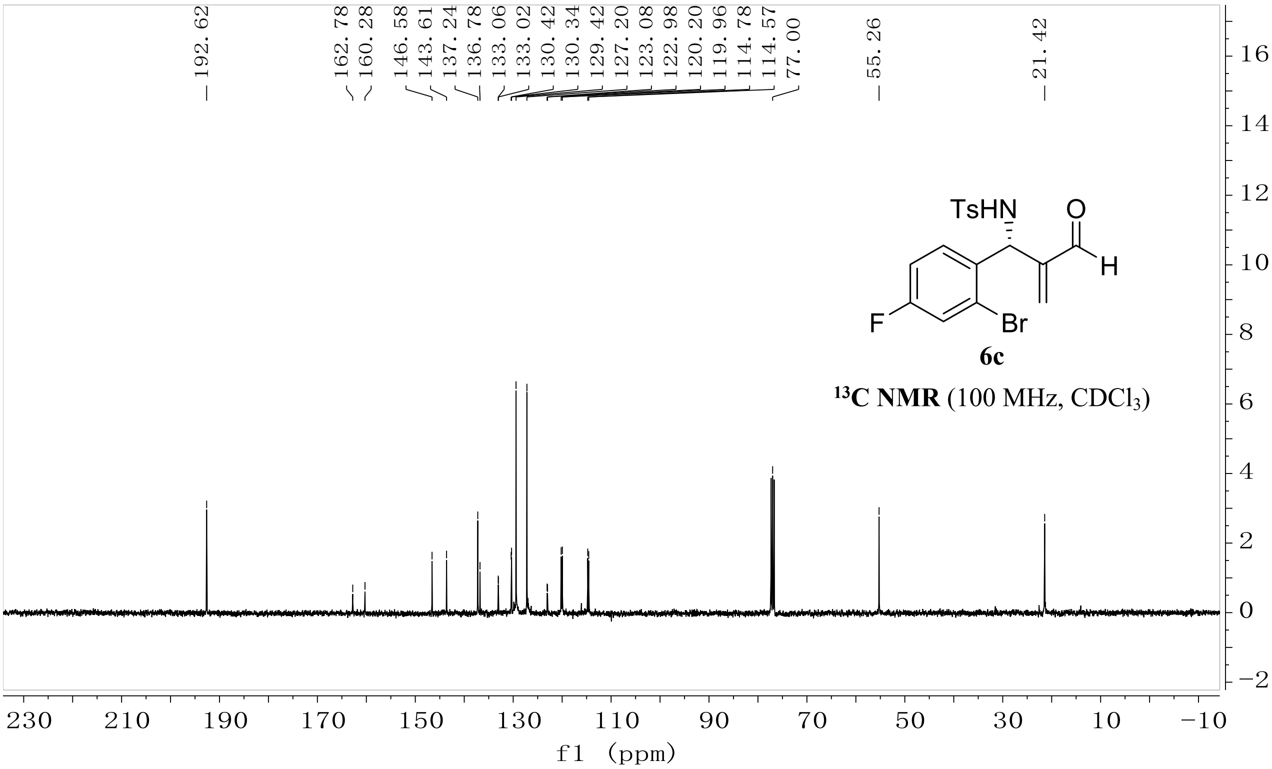

6c

^{13}C NMR (100 MHz, $CDCl_3$)

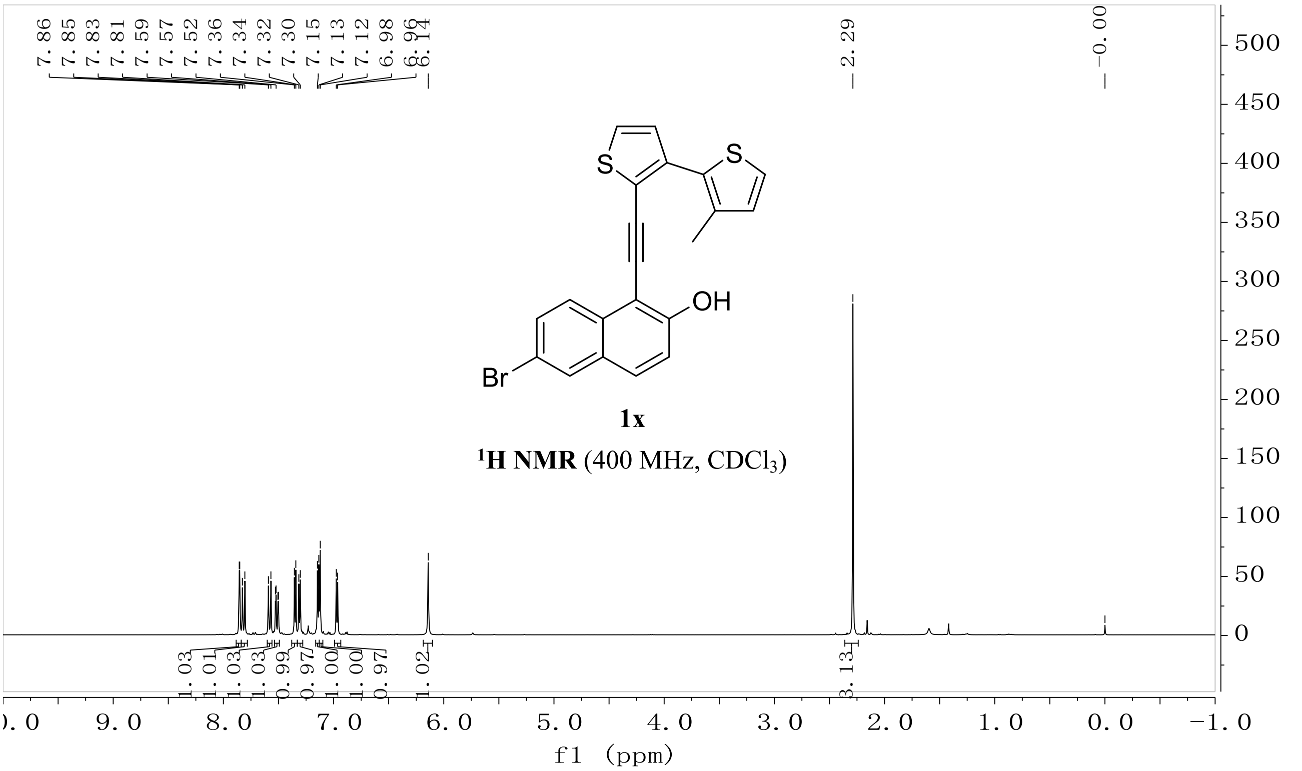
7.86 7.85 7.83 7.81 7.59 7.57 7.52 7.36 7.34 7.32 7.30 7.15 7.13 7.12 6.98 6.96 6.14
2.29
-0.00
1x
¹H NMR (400 MHz, CDCl₃)
1.03 1.01 1.03 1.03 0.99 0.97 1.00 1.00 0.97 1.02
3.13
f1 (ppm)

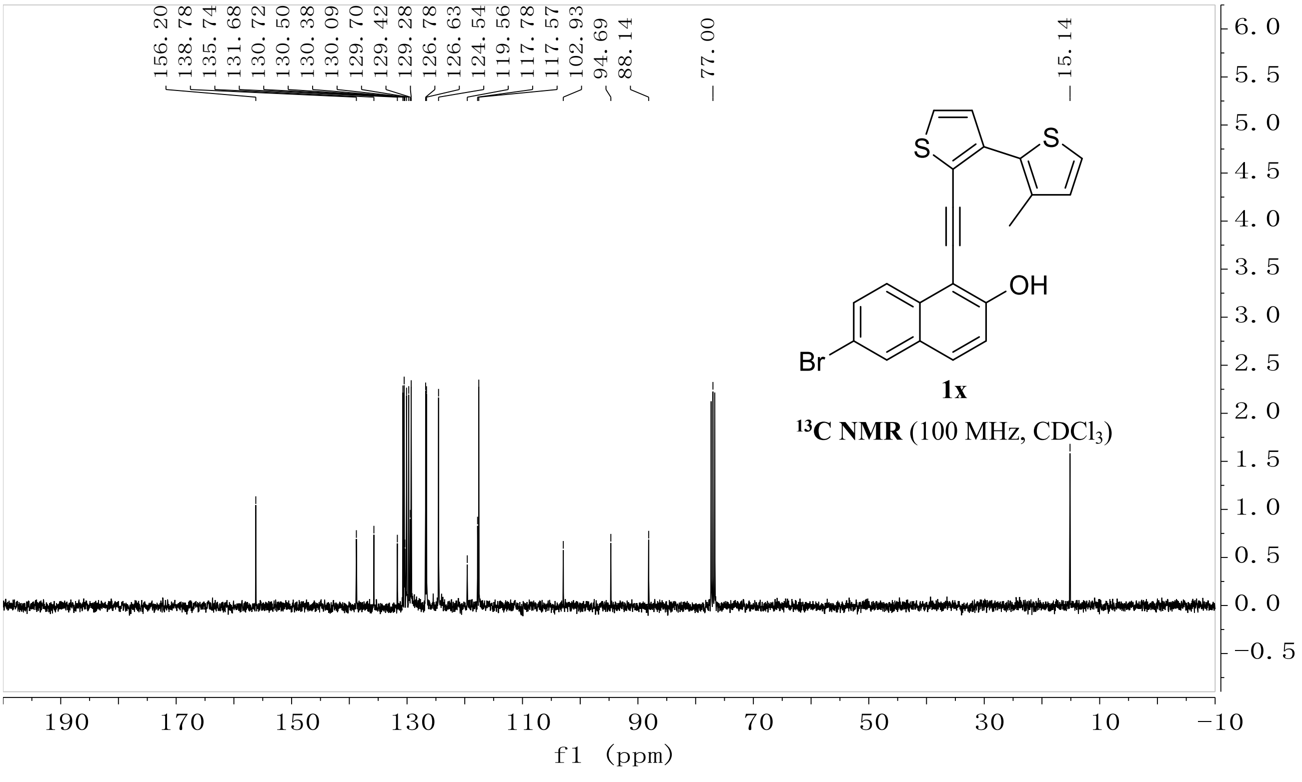
156.20 138.78 135.74 131.68 130.72 130.50 130.38 130.09 129.70 129.42 129.28 126.78 126.63 124.54 119.56 117.78 117.57 102.93 94.69 88.14
77.00
15.14
1x
¹³C NMR (100 MHz, CDCl₃)
f1 (ppm)

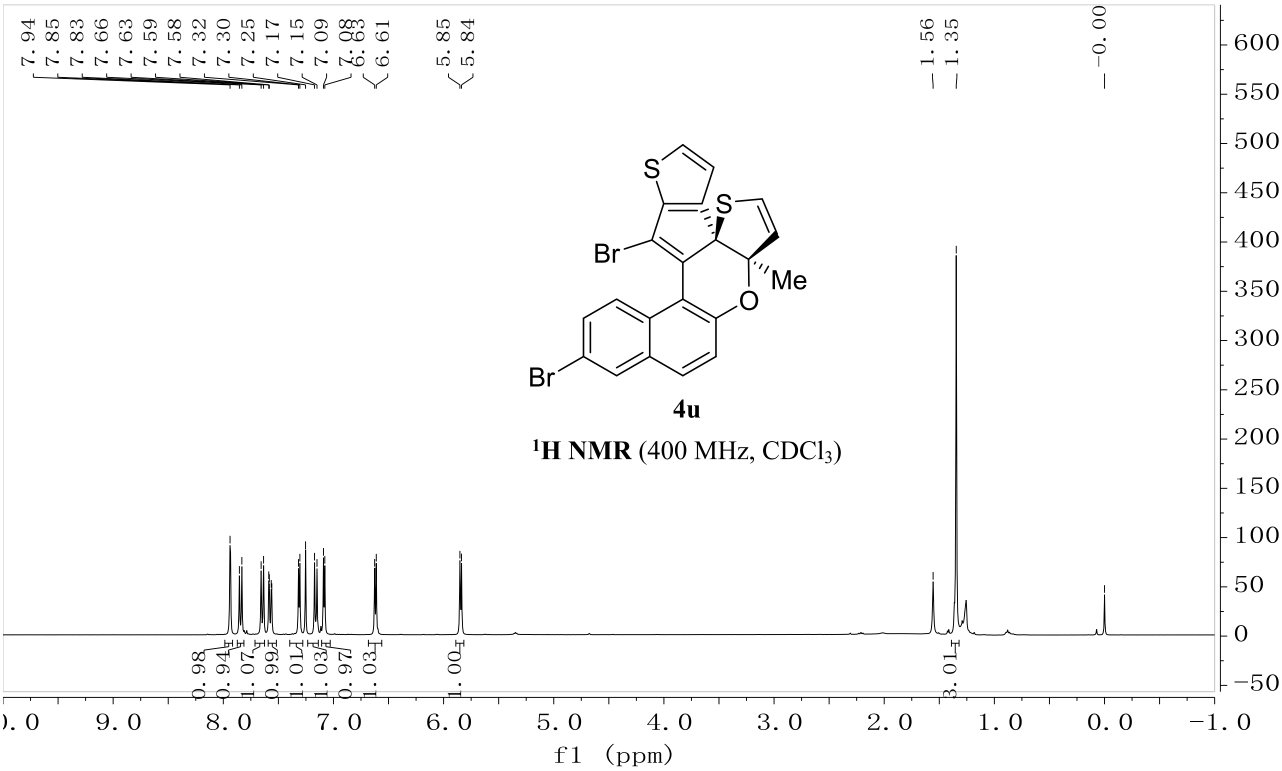

4u

^{1}H NMR (400 MHz, $CDCl_3$)

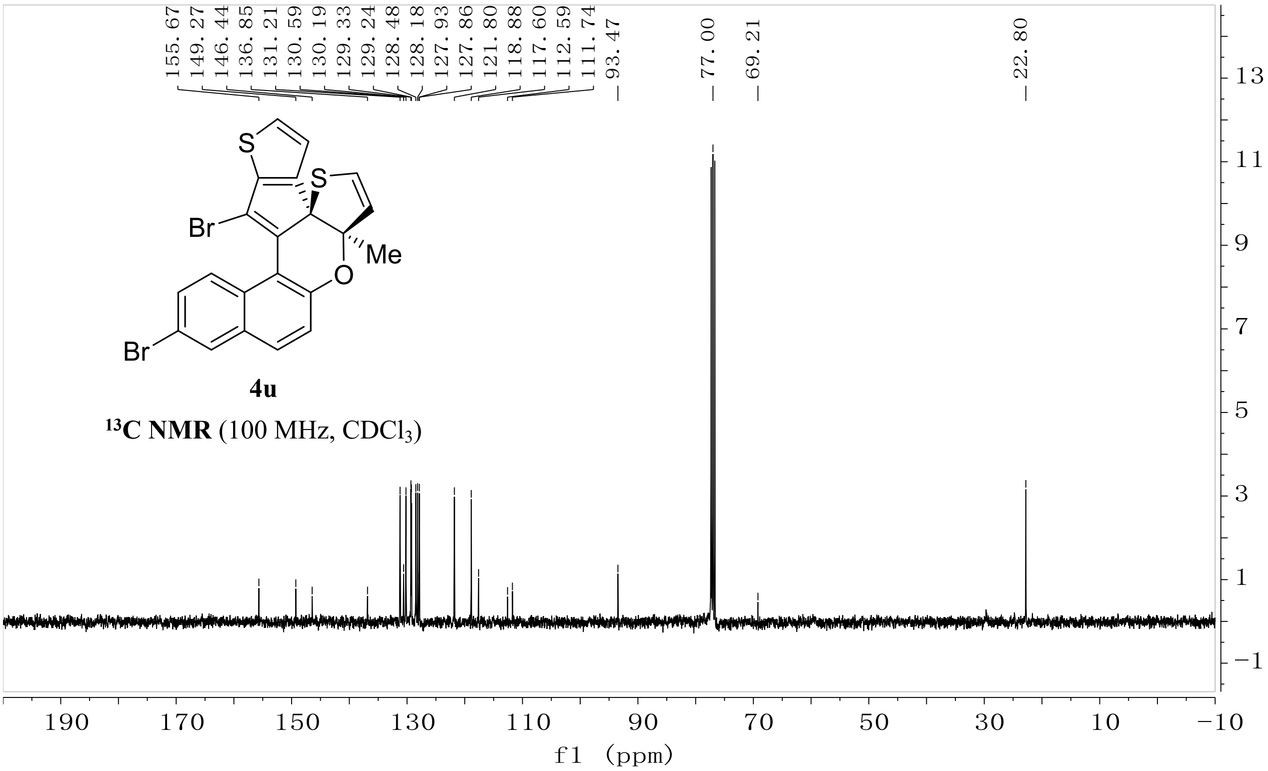

4u

^{13}C NMR (100 MHz, $CDCl_3$)

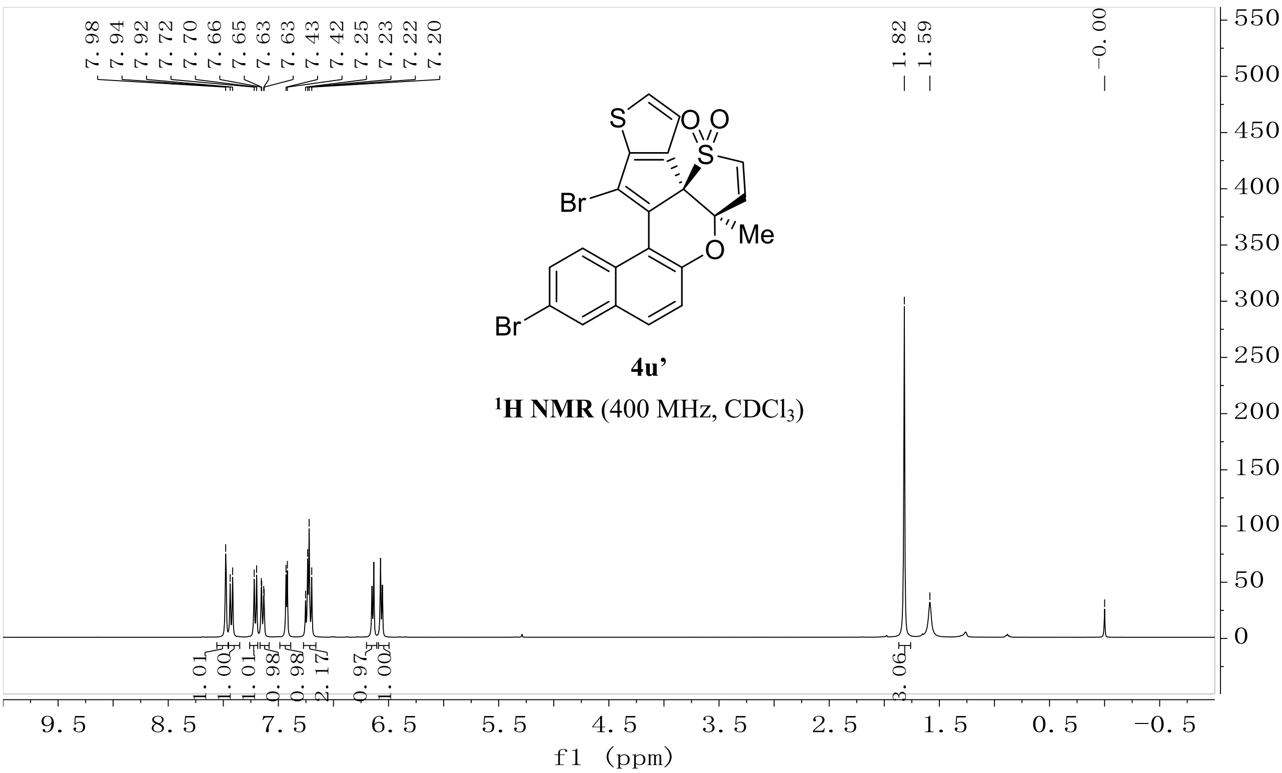
7.98
7.94
7.92
7.72
7.70
7.66
7.65
7.63
7.63
7.43
7.42
7.25
7.23
7.22
7.20
1.82
1.59
-0.00
S
O
O
S
Br
Me
O
Br
4u'
1H NMR (400 MHz, CDCl3)
1.01
1.00
1.01
0.98
0.98
2.17
0.97
1.00
3.06
f1 (ppm)

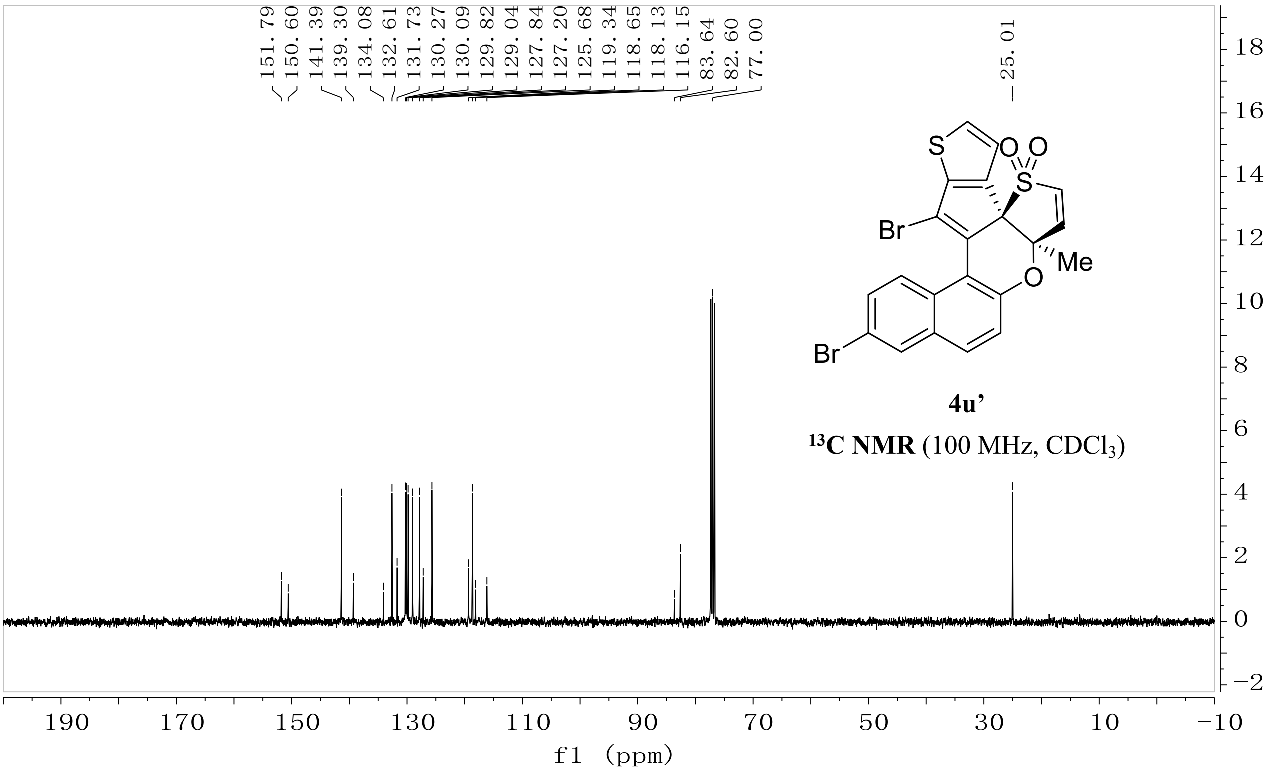
151.79
150.60
141.39
139.30
134.08
132.61
131.73
130.27
130.09
129.82
129.04
127.84
127.20
125.68
119.34
118.65
118.13
116.15
83.64
82.60
77.00
25.01
S
O
O
S
Br
Me
O
Br
4u'
13C NMR (100 MHz, CDCl3)
f1 (ppm)

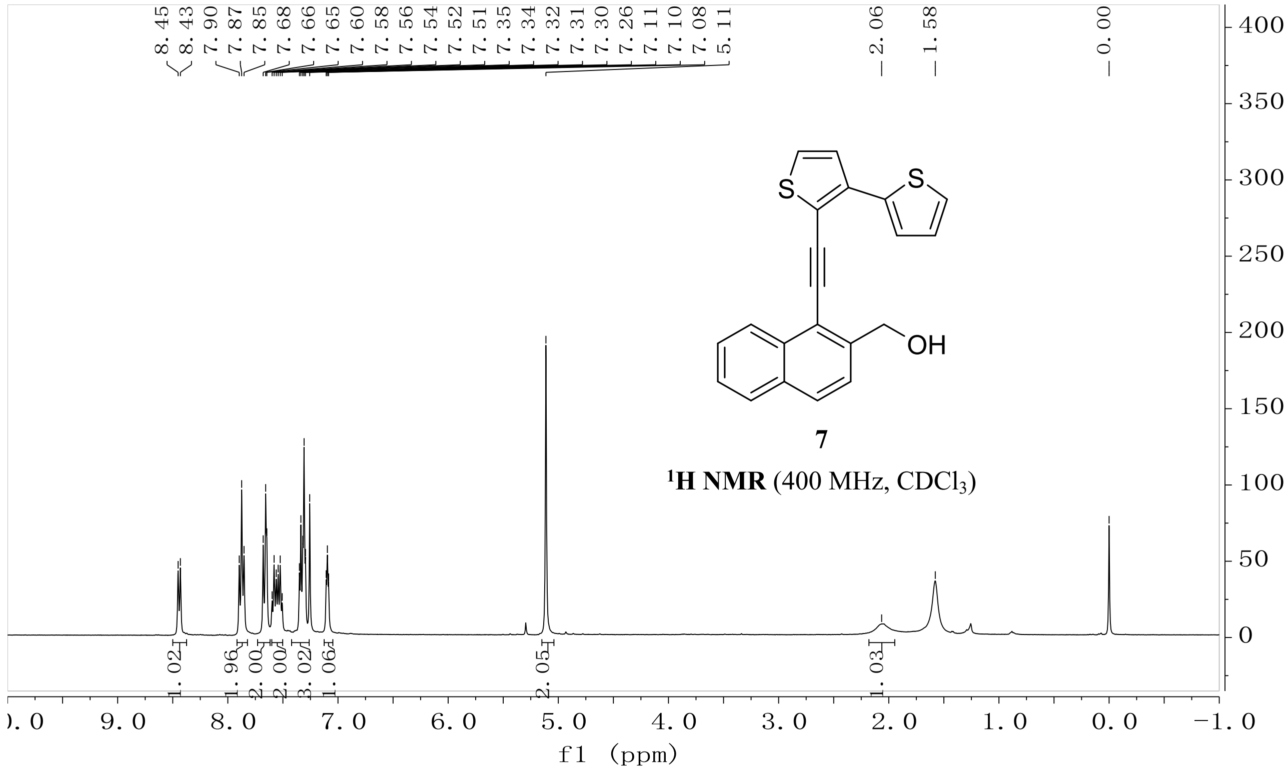
8.45
8.43
7.90
7.87
7.85
7.68
7.66
7.65
7.60
7.58
7.56
7.54
7.52
7.51
7.35
7.34
7.32
7.31
7.30
7.26
7.11
7.10
7.08
5.11
2.06
1.58
0.00
1.02
1.96
2.00
2.00
3.02
1.06
2.05
1.03
S
S
OH
7
1H NMR (400 MHz, CDCl3)
f1 (ppm)

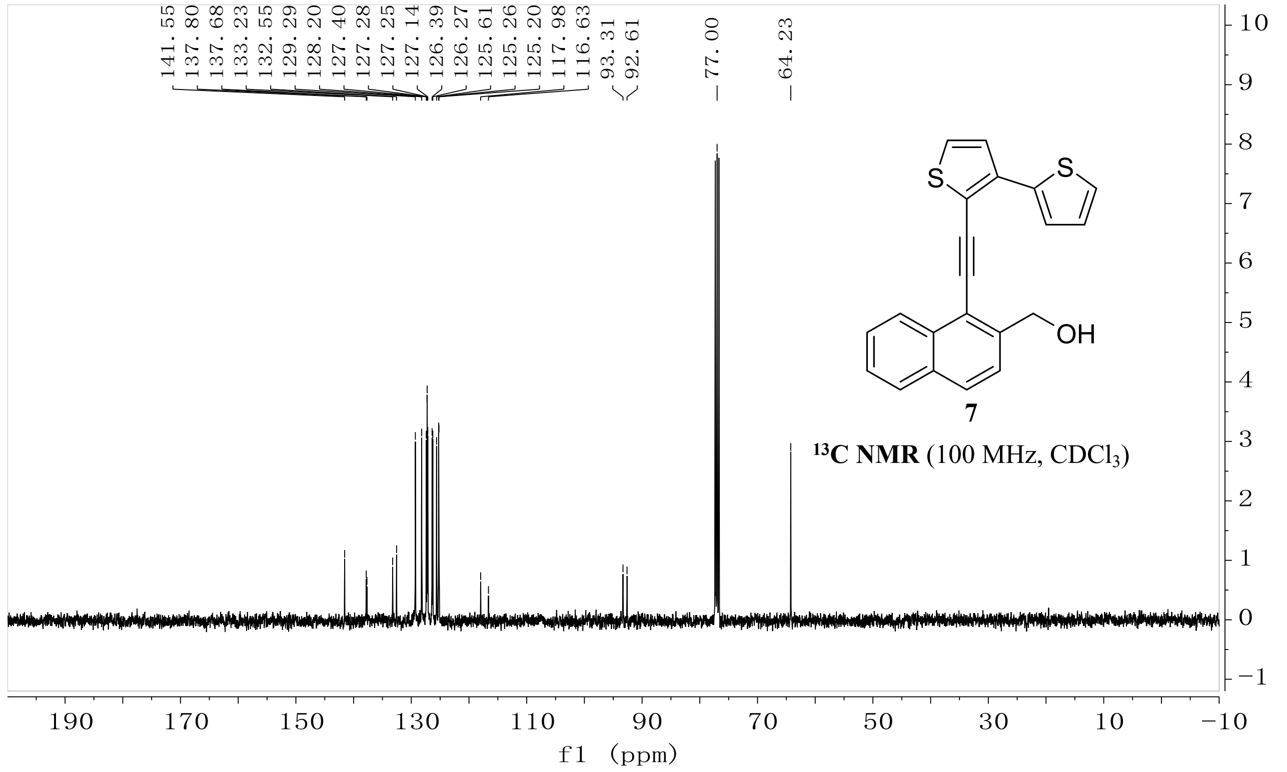
141.55
137.80
137.68
133.23
132.55
129.29
128.20
127.40
127.28
127.25
127.14
126.39
126.27
125.61
125.26
125.20
117.98
116.63
93.31
92.61
77.00
64.23
S
S
OH
7
13C NMR (100 MHz, CDCl3)
f1 (ppm)

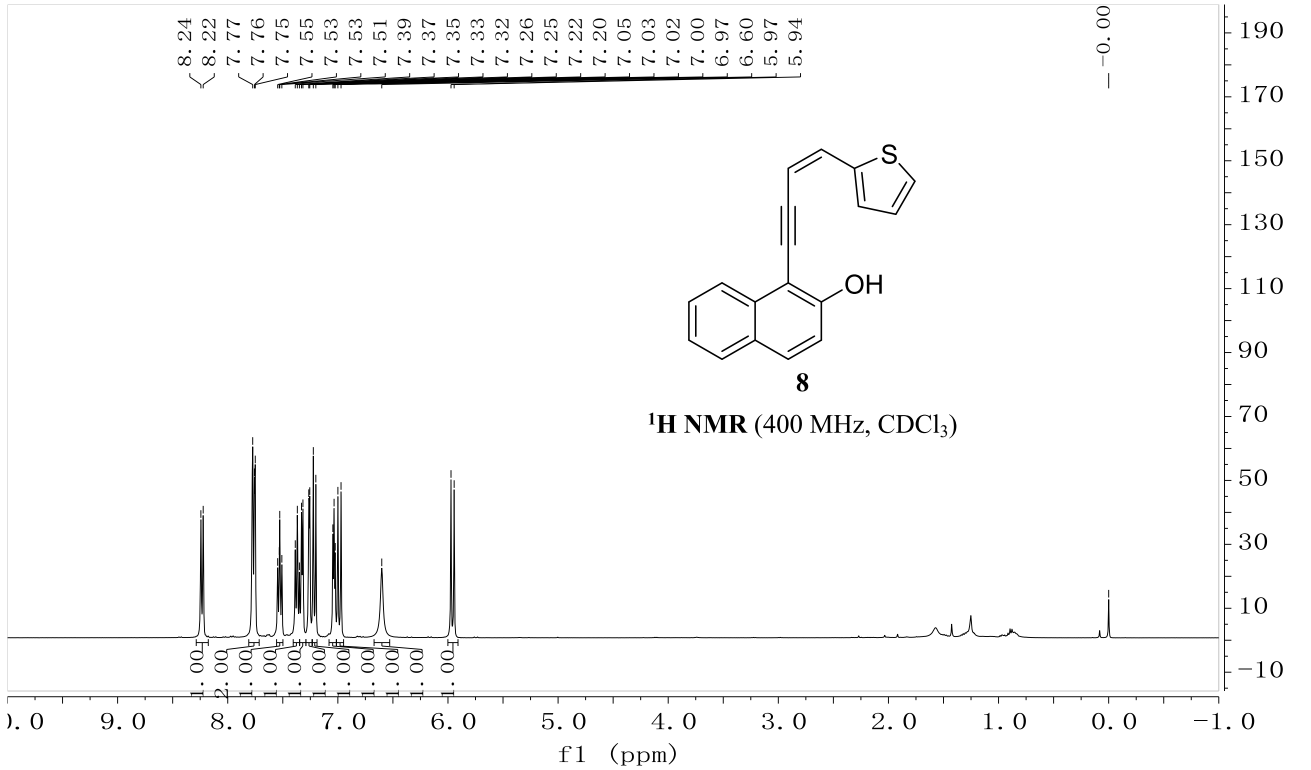
8.24
8.22
7.77
7.76
7.75
7.55
7.53
7.53
7.51
7.39
7.37
7.35
7.33
7.32
7.26
7.25
7.22
7.20
7.05
7.03
7.02
7.00
6.97
6.60
5.97
5.94
-0.00
OH
S
8
^{1}H NMR (400 MHz, CDCl$_3$)
1.00
2.00
1.00
1.00
1.00
1.00
1.00
1.00
1.00
1.00
1.00
f1 (ppm)

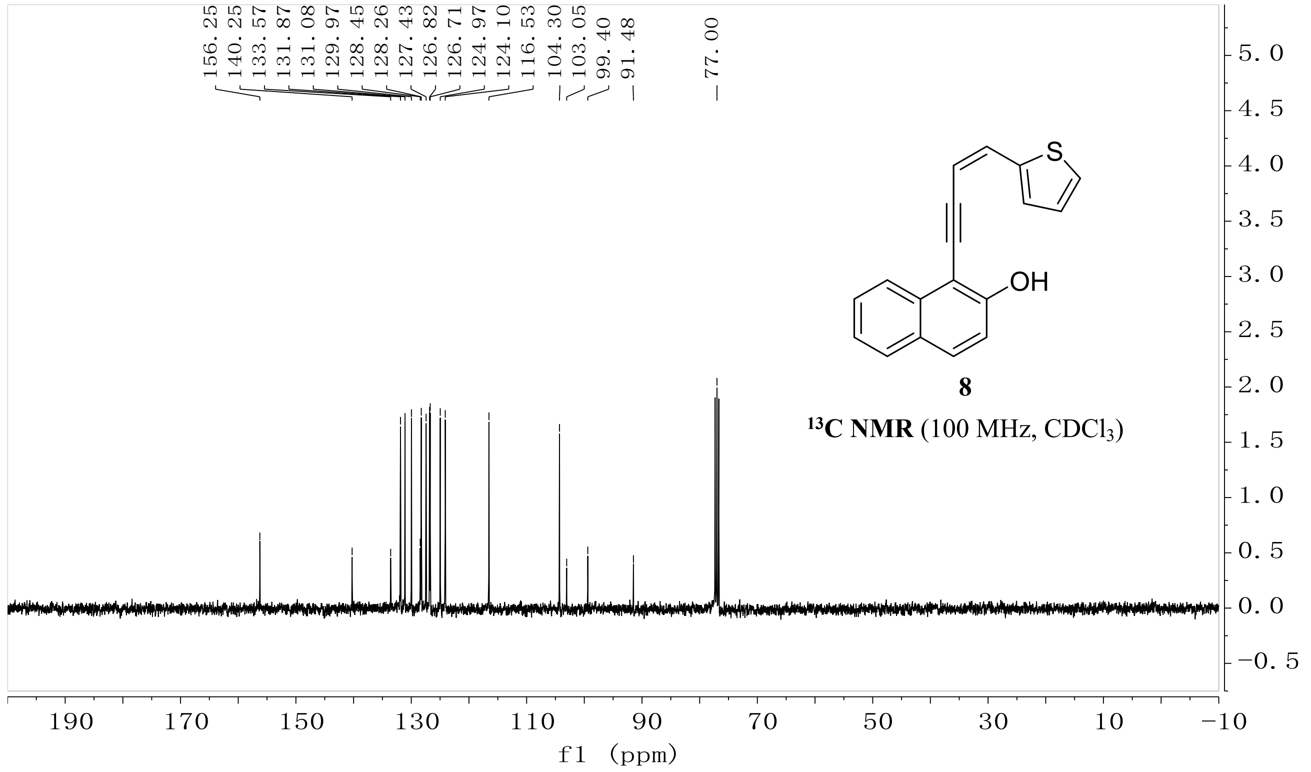
156.25
140.25
133.57
131.87
131.08
129.97
128.45
128.26
127.43
126.82
126.71
124.97
124.10
116.53
104.30
103.05
99.40
91.48
77.00
OH
S
8
^{13}C NMR (100 MHz, CDCl$_3$)
f1 (ppm)

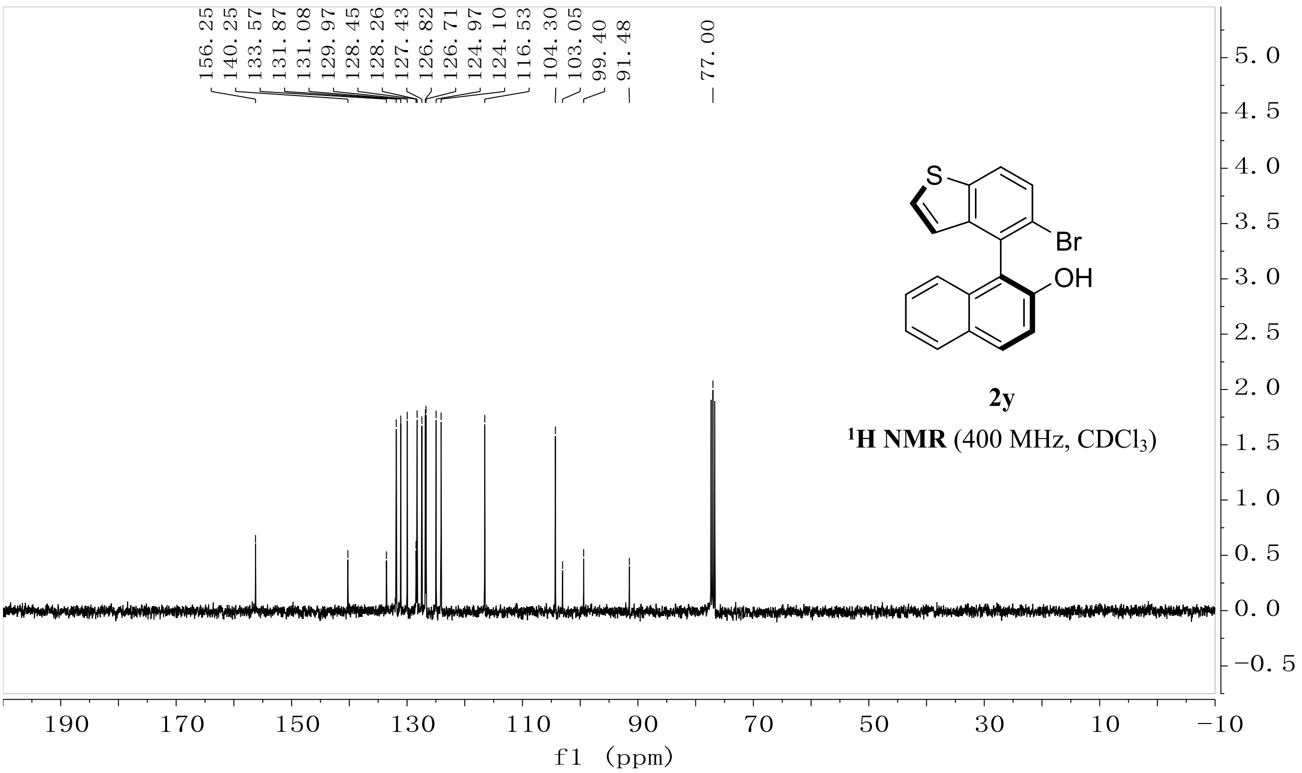

156.25
140.25
133.57
131.87
131.08
129.97
128.45
128.26
127.43
126.82
126.71
124.97
124.10
116.53
104.30
103.05
99.40
91.48
77.00
S
Br
OH
2y
1H NMR (400 MHz, CDCl3)
5.0
4.5
4.0
3.5
3.0
2.5
2.0
1.5
1.0
0.5
0.0
−0.5
190 170 150 130 110 90 70 50 30 10 −10
f1 (ppm)

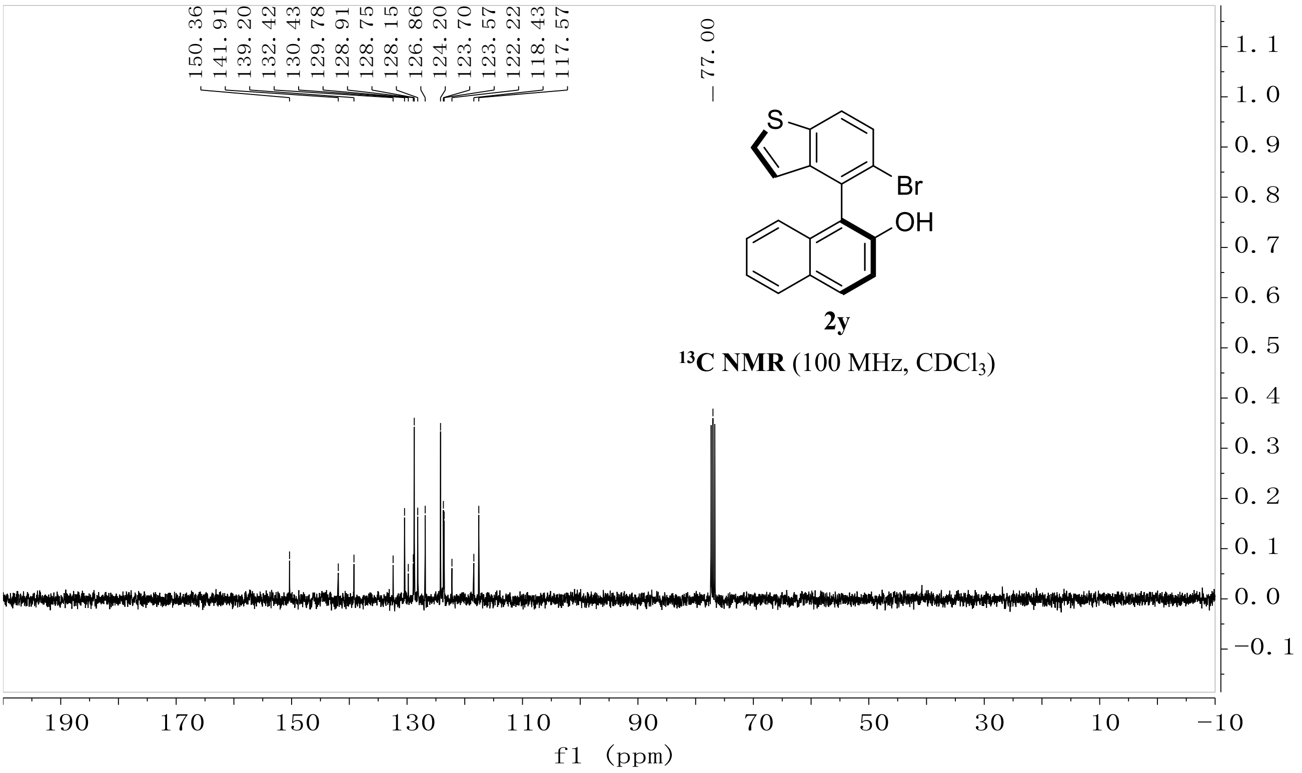

150.36
141.91
139.20
132.42
130.43
129.78
128.91
128.75
128.15
126.86
124.20
123.70
123.57
122.22
118.43
117.57
77.00
S
Br
OH
2y
13C NMR (100 MHz, CDCl3)
1.1
1.0
0.9
0.8
0.7
0.6
0.5
0.4
0.3
0.2
0.1
0.0
−0.1
190 170 150 130 110 90 70 50 30 10 −10
f1 (ppm)

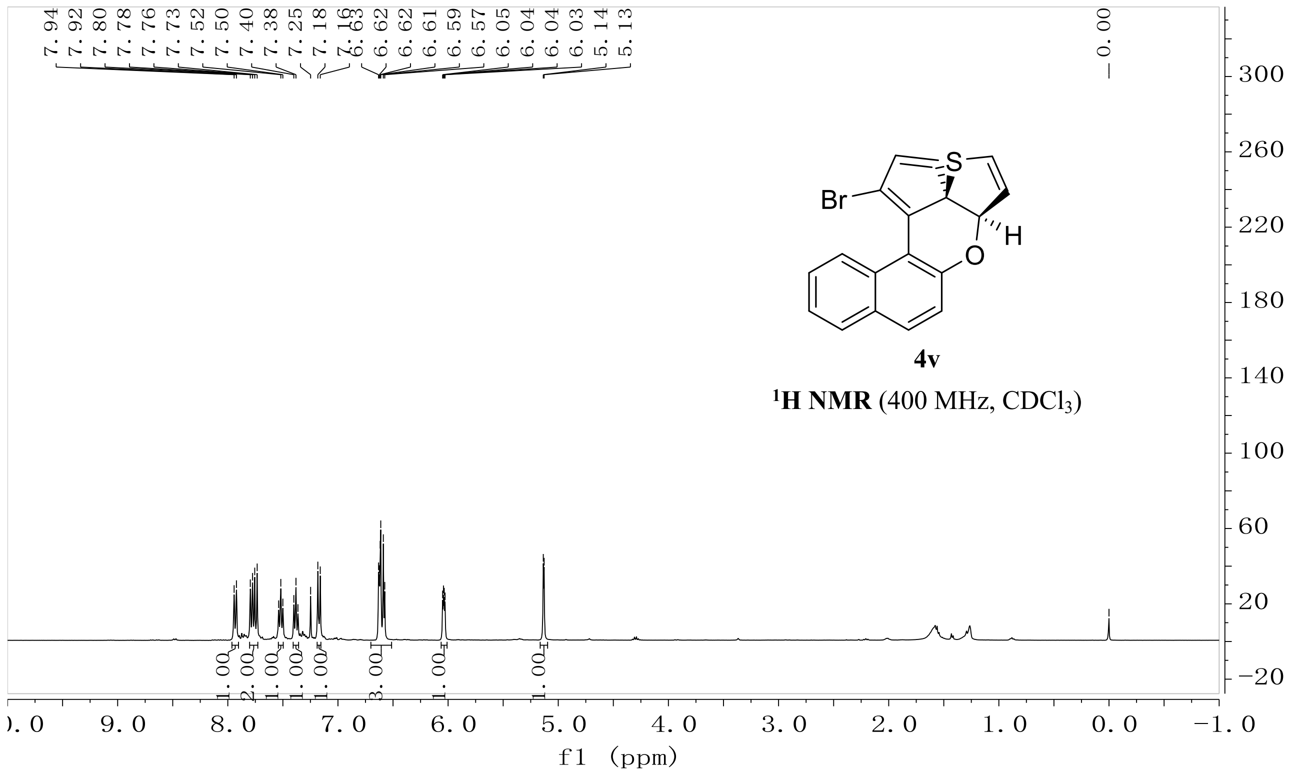
7.94 7.92 7.80 7.78 7.76 7.73 7.52 7.50 7.40 7.38 7.25 7.18 7.16 6.63 6.62 6.62 6.61 6.59 6.57 6.05 6.04 6.04 6.03 5.14 5.13
0.00
Br
S
O
H
4v
^{1}H NMR (400 MHz, CDCl$_3$)
1.00 2.00 1.00 1.00 1.00 3.00 1.00 1.00
f1 (ppm)

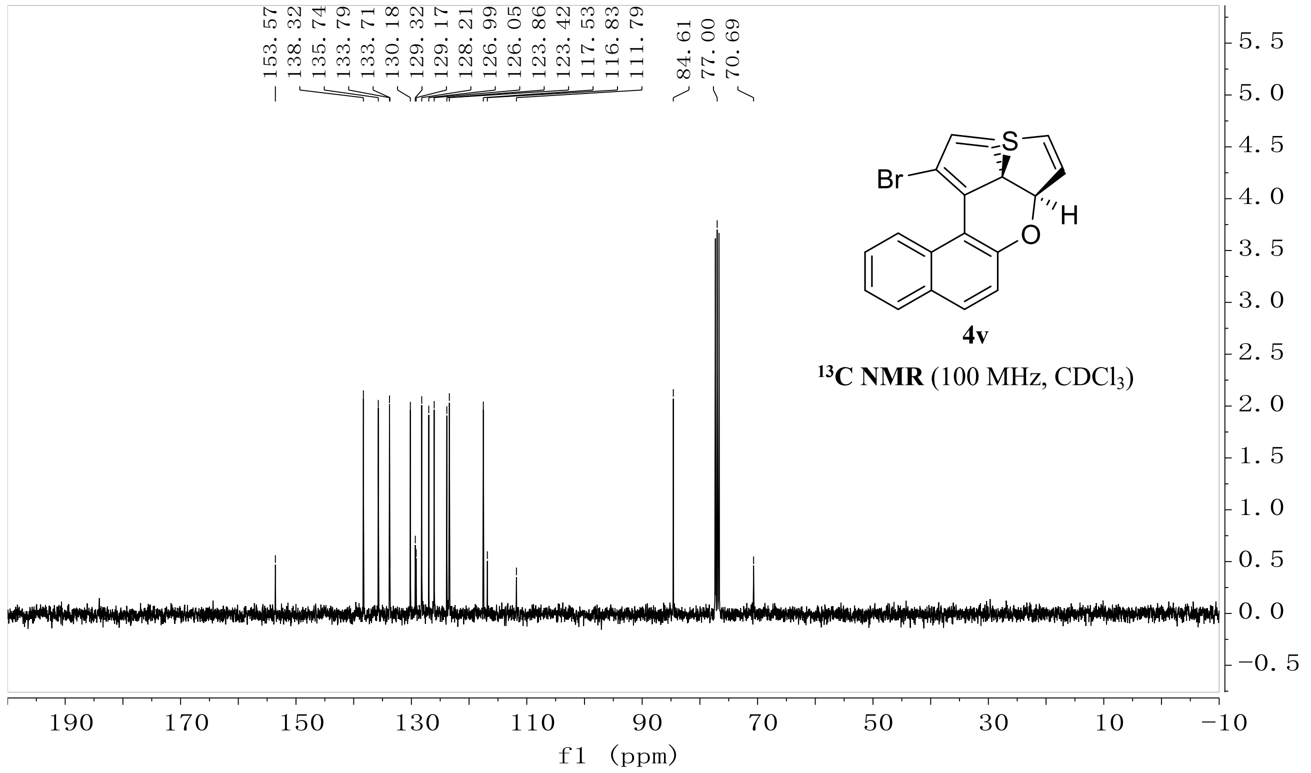
153.57 138.32 135.74 133.79 133.71 130.18 129.32 129.17 128.21 126.99 126.05 123.86 123.42 117.53 116.83 111.79
84.61 77.00 70.69
Br
S
O
H
4v
^{13}C NMR (100 MHz, CDCl$_3$)
f1 (ppm)

XIV. X-ray crystallographic information

The authors thank Mr. Xiangnan Gong (Analytical and Testing Center of Chongqing University) for spectroscopic measurements.

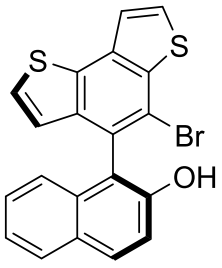

2a
CCDC 2203136

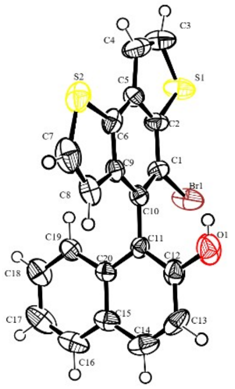

Supplementary Figure 2. ORTEP-style illustration of 2a with ellipsoids at 50% probability

Identification code	20220411-ZZX-0411-200K
Empirical formula	C20H11BrOS2
Formula weight	411.32
Temperature/K	200.00(10)
Crystal system	trigonal
Space group	P32
a/Å	12.4762(8)
b/Å	12.4762(8)
c/Å	9.5066(6)
α/°	90
β/°	90
γ/°	120
Volume/Å3	1281.50(18)
Z	3
ρcalcg/cm3	1.599
μ/mm-1	2.653
F(000)	618.0
Crystal size/mm3	0.36 × 0.35 × 0.32
Radiation	Mo Kα (λ = 0.71073)
2Θ range for data collection/°	3.77 to 58.032
Index ranges	-15 ≤ h ≤ 15, -15 ≤ k ≤ 15, -12 ≤ l ≤ 12
Reflections collected	6071
Independent reflections	3885 [Rint = 0.0325, Rsigma = 0.0753]
Data/restraints/parameters	3885/1/218
Goodness-of-fit on F2	0.997
Final R indexes [I>=2σ (I)]	R1 = 0.0458, wR2 = 0.0653
Final R indexes [all data]	R1 = 0.0704, wR2 = 0.0771
Largest diff. peak/hole / e Å-3	0.40/-0.62
Flack parameter	-0.007(5)

The CIF file is available on CCDC (2203136). The author response to the check CIF alert is shown below:

Alert level B: PLAT420_ALERT_2_B D-H Without Acceptor O1---H1.   Please Check

RESPONSE: This alert is from the existence of naphthol bearing an O-H bond forming no hydrogen bonds. The probable hydrogen bond is the O1—H1---S2. But in the molecular structure, the distance between S005 and H007 is far greater than the critical value of hydrogen bond formation. The angle and distance between O1—H1---S2 are inappropriate. In this case, there is no potential hydrogen bond acceptor near the naphthol molecule.

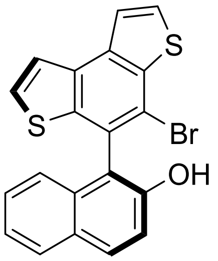

2l
CCDC 2203144

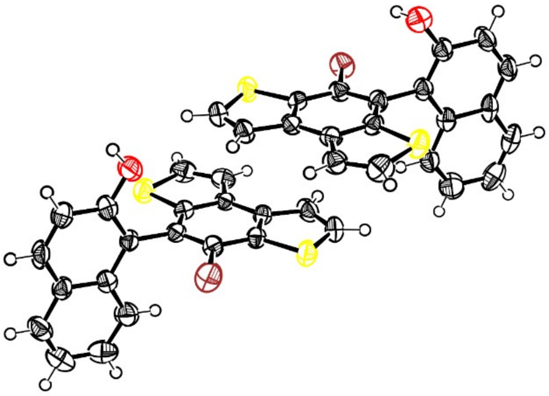

Supplementary Figure 3. ORTEP-style illustration of 2l with ellipsoids at 50% probability

Identification code	20220613-ZZX-1291
Empirical formula	C20H11BrOS2
Formula weight	411.32
Temperature/K	293(2)
Crystal system	monoclinic
Space group	P21
a/Å	7.4965(3)
b/Å	19.1120(7)
c/Å	11.7078(4)
α/°	90
β/°	96.131(4)
γ/°	90
Volume/Å3	1667.82(11)
Z	4
ρcalcg/cm3	1.638
μ/mm-1	2.718
F(000)	824.0
Crystal size/mm3	0.38 × 0.36 × 0.31
Radiation	Mo Kα (λ = 0.71073)
2Θ range for data collection/°	3.498 to 58.136
Index ranges	-9 ≤ h ≤ 10, -24 ≤ k ≤ 24, -15 ≤ l ≤ 15
Reflections collected	24487
Independent reflections	7929 [Rint = 0.0384, Rsigma = 0.0493]
Data/restraints/parameters	7929/1/435
Goodness-of-fit on F2	1.030
Final R indexes [I>=2σ (I)]	R1 = 0.0440, wR2 = 0.0946
Final R indexes [all data]	R1 = 0.0708, wR2 = 0.1082
Largest diff. peak/hole / e Å-3	1.31/-0.40

Flack parameter	0.001(5)

The CIF file is available on CCDC (2203144). The author response to the check CIF alert is shown below:

PLAT420_ALERT_2_B D-H Bond Without Acceptor O007---H007 Please Check

RESPONSE: This alert is from the existence of naphthol bearing an O-H bond forming no hydrogen bonds. The probable hydrogen bond is the O007—H007---S005. But in the molecular structure, the distance between S005 and H007 is far greater than the critical value of hydrogen bond formation. The angle and distance between O007—H007---S005 are inappropriate. In this case, there is no potential hydrogen bond acceptor near the naphthol molecule.

PLAT919_ALERT_3_B Reflection # Likely Affected by the Beamstop 1 Check

RESPONSE: This alert reports the number of reflections with intensities is affected by the beamstop. The beamstop is a electronic component used to protect the detector from the strong X-Ray, it may may affect the signal at certain angles.

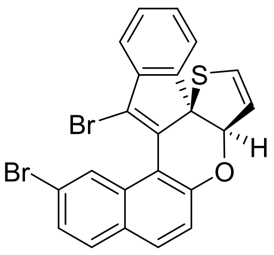

4b
CCDC 2203132

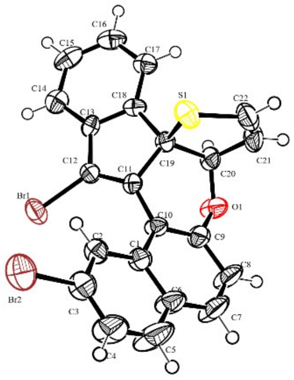

Supplementary Figure 4. ORTEP-style illustration of 4b with ellipsoids at 50% probability

Identification code	20201230-Yan HL-PL-ZZA-53
Empirical formula	C22H12Br2OS
Formula weight	484.20
Temperature/K	295
Crystal system	orthorhombic
Space group	P212121
a/Å	8.0277(5)
b/Å	10.4982(4)
c/Å	22.0586(11)
α/°	90
β/°	90
γ/°	90
Volume/Å3	1859.02(16)
Z	4
ρcalcg/cm3	1.730
μ/mm-1	4.481
F(000)	952.0
Crystal size/mm3	? × ? × ?
Radiation	MoKα (λ = 0.71073)
2Θ range for data collection/°	6.766 to 58.222
Index ranges	-10 ≤ h ≤ 9, -13 ≤ k ≤ 14, -30 ≤ l ≤ 16
Reflections collected	7547
Independent reflections	4232 [Rint = 0.0374, Rsigma = 0.0728]
Data/restraints/parameters	4232/0/235
Goodness-of-fit on F2	1.071
Final R indexes [I>=2σ (I)]	R1 = 0.0529, wR2 = 0.1060
Final R indexes [all data]	R1 = 0.0856, wR2 = 0.1236
Largest diff. peak/hole / e Å-3	0.59/-0.71
Flack parameter	0.000(11)

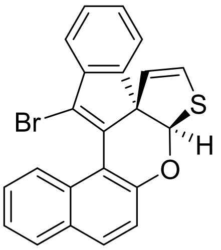

4l
CCDC 2203141

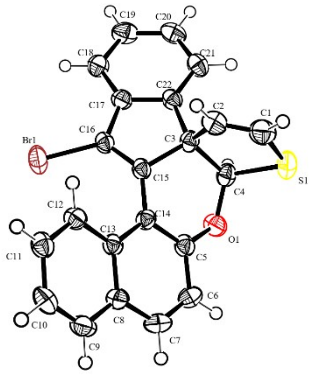

Figure 5. ORTEP-style illustration of 4l with ellipsoids at 50% probability

Identification code	20220613-ZZX-1290
Empirical formula	$C_{22}H_{13}BrOS$
Formula weight	405.29
Temperature/K	293(2)
Crystal system	monoclinic
Space group	$P2_1$
a/Å	9.0211(4)
b/Å	8.9321(3)
c/Å	11.4528(6)
α/°	90
β/°	113.132(6)
γ/°	90
Volume/Å^3	848.64(7)
Z	2
ρ_{calc}g/cm^3	1.586
μ/mm^{-1}	2.551
F(000)	408.0
Crystal size/mm^3	0.43 × 0.39 × 0.35
Radiation	Mo Kα (λ = 0.71073)
2Θ range for data collection/°	3.868 to 58.68
Index ranges	$-11 \le h \le 12$, $-11 \le k \le 12$, $-14 \le l \le 14$
Reflections collected	13415
Independent reflections	4043 [R_{int} = 0.0359, R_{sigma} = 0.0356]
Data/restraints/parameters	4043/1/226
Goodness-of-fit on F^2	1.077
Final R indexes [I>=2σ (I)]	R_1 = 0.0347, wR_2 = 0.0781
Final R indexes [all data]	R_1 = 0.0431, wR_2 = 0.0834
Largest diff. peak/hole / e Å^{-3}	0.30/-0.82
Flack parameter	-0.006(4)

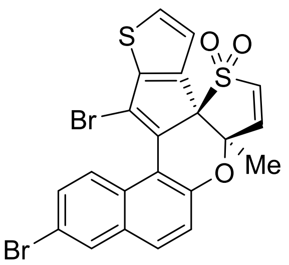

4u'

CCDC 2203146

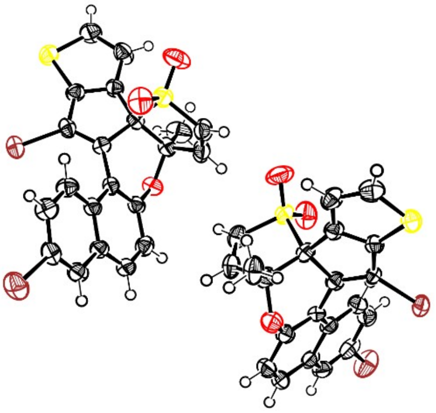

Supplementary Figure 6. ORTEP-style illustration of 4u' with ellipsoids at 50% probability

Identification code	20220708-ZZX-1312
Empirical formula	$C_{42}H_{24}Br_4O_6S_4$
Formula weight	1072.49
Temperature/K	293(2)
Crystal system	monoclinic
Space group	$P2_1$
a/Å	13.1561(6)
b/Å	10.0689(3)
c/Å	15.4836(7)
α/°	90
β/°	109.024(5)
γ/°	90
Volume/Å^3	1939.05(15)
Z	2
ρ_{calc}g/cm^3	1.837
μ/mm^{-1}	4.417
F(000)	1056.0
Crystal size/mm^3	0.35 × 0.34 × 0.25
Radiation	Mo Kα (λ = 0.71073)
2Θ range for data collection/°	3.274 to 58.174
Index ranges	-17 ≤ h ≤ 17, -13 ≤ k ≤ 13, -15 ≤ l ≤ 21
Reflections collected	15893
Independent reflections	8839 [R_{int} = 0.0314, R_{sigma} = 0.0650]
Data/restraints/parameters	8839/1/507
Goodness-of-fit on F^2	1.023
Final R indexes [I>=2σ (I)]	R_1 = 0.0405, wR_2 = 0.0608
Final R indexes [all data]	R_1 = 0.0687, wR_2 = 0.0734
Largest diff. peak/hole / e Å^{-3}	0.39/-0.44
Flack parameter	-0.002(4)

The CIF file is available on CCDC (2203146). The author response to the check CIF alert is shown below:

PLAT919_ALERT_3_B Reflection # Likely Affected by the Beamstop 1 Check

RESPONSE: This alert reports the number of reflections with intensities is affected by the beamstop. The beamstop is a electronic component used to protect the detector from the strong X-Ray, it may may affect the signal at certain angles.

References

1. Jia, S.; Chen, Z.; Zhang, N.; Tan, Y.; Liu, Y.; Deng, J.; Yan, H. *J. Am. Chem. Soc.* **2018**, *140*, 7056-7060.
2. 9 Gaussian 09, Revision D.01, M. J. Frisch, G. W. Trucks, H. B. Schlegel, G. E.Scuseria, M. A. Robb, J. R. Cheeseman, G. Scalmani, V. Barone, G. A. Petersson, H. Nakatsuji, X. Li, M. Caricato, A. Marenich, J. Bloino, B. G. Janesko, R. Gomperts, B. Mennucci, H. P. Hratchian, J. V. Ortiz, A. F. Izmaylov,J. L. Sonnenberg, D. Williams-Young, F. Ding, F. Lipparini, F. Egidi, J.Goings, B. Peng, A. Petrone, T. Henderson, D. Ranasinghe, V. G. Zakrzewski, J. Gao, N. Rega, G. Zheng, W. Liang, M. Hada, M. Ehara, K. Toyota, R.Fukuda, J. Hasegawa, M. Ishida, T. Nakajima, Y. Honda, O. Kitao, H.Nakai, T. Vreven, K. Throssell, J. A. Montgomery, Jr., J. E. Peralta, F.Ogliaro, M. Bearpark, J. J. Heyd, E. Brothers, K. N. Kudin, V. N. Staroverov,T. Keith, R. Kobayashi, J. Normand, K. Raghavachari, A. Rendell, J. C.Burant, S. S. Iyengar, J. Tomasi, M. Cossi, J. M. Millam, M. Klene, C.Adamo, R. Cammi, J. W. Ochterski, R. L. Martin, K. Morokuma, O. Farkas,J. B. Foresman, D. J. Fox, Gaussian, Inc., Wallingford CT, **2016**.
3. A. V. Marenich, C. J. Cramer, D. G. Truhlar, *J. Phys. Chem. B.* **2009**, *113*, 6378–6396.
4. (a) A. D. Becke, *J. Chem. Phys.* **1993**, *98*, 5648 – 5652; (b) C. Lee, W. Yang,R. G. Parr, *Phys. Rev. B* **1988**, *37*, 785–789.
5. (a) Ditchfield, R.; Hehre, W. J.; Pople, J. A. *J. Chem. Phys.* **1971**, *54*, 724-728. (b) Hariharan, P. C.; Pople, J. A. *Theor. Chim. Acta* **1973**, *28*, 213-222. (c) Hehre, W. J.; Ditchfield, R.; Pople, J. A. *J. Chem. Phys.* **1972**, *56*, 2257-2261.
6. Krishnan, R., Binkley, J. S., Seeger, R. & Pople, J. A. *J. Chem. Phys.* **1980**, *72*, 650-654.
7. He, T.; Peng, L.; Li, S.; Hu, F.; Xie, C.; Huang, S.; Jia, S.; Qin, W.; Yan, H. *Org. Lett.* **2020**, *22*, 6966−6971.
8. Love, B. E.; Raje, P. S. *Synlett*. **1994**, 493.
9. Schmidt, Y.; Breit, B. *Chem. Eur. J.* **2011**, *17*, 11789 – 11796.
